# Supplementary material for: Menagerie: A text-mining tool to support animal-human translation in neurodegeneration research
Source: PLoS One. 2019 Dec 17;14(12):e0226176. doi: 10.1371/journal.pone.0226176 (PMC6917268; doi:10.1371/journal.pone.0226176)
Supplement: S1 Table — Primary data sources included clinical trials and journal articles. Meta-analyses were included in this category as they yield new insights through statistical analysis of multiple studies. Reviews, case reports, editorials and letters comprised secondary data. In general, approximately 70% of studies report a promising outcome (POS) with proportions in primary data sources being slightly lower than in all data sources. In both datasets, a slightly increasing trend in promising reports across 10 years was noted. (DOCX) [file pone.0226176.s002.docx]

**S1 Table:** Proportions of outcome reporting by outcome type in primary and secondary data sources.

|  | **Total (% primary)** | **% POS** | **% MIXED** | **% NEG** | **% OTHER** |
| --- | --- | --- | --- | --- | --- |
| 2008 all | 3433 | 0.71 | 0.18 | 0.05 | 0.06 |
| 2008 primary | 2408 (0.7) | 0.69 | 0.18 | 0.06 | 0.06 |
| 2012 all | 4727 | 0.72 | 0.17 | 0.05 | 0.05 |
| 2012 primary | 3554 (0.75) | 0.70 | 0.17 | 0.06 | 0.06 |
| 2017 all | 6321 | 0.73 | 0.17 | 0.05 | 0.05 |
| 2017 primary | 4546 (0.72) | 0.72 | 0.18 | 0.05 | 0.05 |

Primary data sources included clinical trials and journal articles. Meta-analyses were included in this category as they yield new insights through statistical analysis of multiple studies. Reviews, case reports, editorials and letters comprised secondary data. In general, approximately 70% of studies report a promising outcome (POS) with proportions in primary data sources being slightly lower than in all data sources. In both datasets, a slightly increasing trend in promising reports across 10 years was noted.
