# Supplementary material for: Menagerie: A text-mining tool to support animal-human translation in neurodegeneration research
Source: PLoS One. 2019 Dec 17;14(12):e0226176. doi: 10.1371/journal.pone.0226176 (PMC6917268; doi:10.1371/journal.pone.0226176)
Supplement: S2 Table — This module extracts pharmacologic and non-pharmacologic interventions, as well as genes or co-morbidities that influence the Parkinson’s disease phenotype. These are extracted from the title of the abstract only. (DOCX) [file pone.0226176.s003.docx]

**S2 Table:** Interventions/disease modifiers extracted over 10 years. This module extracts pharmacologic and non-pharmacologic interventions, as well as genes or co-morbidities that influence the Parkinson’s disease phenotype. These are extracted from the title of the abstract only.

| **Text Term** | **UMLS/MeSH term** | **2008** | **2012** | **2017** | **2017/2012** |
| --- | --- | --- | --- | --- | --- |
| alpha synuclein | D051844 | 138 | 165 | 213 | 1.29 |
| Deep brain stimulations | D046690 | 69 | 152 | 156 | 1.03 |
| levodopa | D007980 | 92 | 121 | 140 | 1.16 |
| parkin | C084820 | 34 | 43 | 48 | 1.12 |
| physical therapy | C0949766 | 10 | 30 | 47 | 1.57 |
| exercise | D015444 | 9 | 30 | 46 | 1.53 |
| stem cell therapy | D013234 | 31 | 61 | 41 | 0.67 |
| stimulation | C1292856 | 52 | 48 | 37 | 0.77 |
| iron | D007501 | 7 | 15 | 29 | 1.93 |
| Adenosine A2A | C0255998 | 27 | 17 | 24 | 1.41 |
| amyloid | D000682 | 7 | 20 | 21 | 1.05 |
| transplantation | D014180 | 18 | 28 | 20 | 0.71 |
| medication | C0013227 | 9 | 18 | 20 | 1.11 |
| carbidopa | D002230 | 0 | 4 | 18 | 4.50 |
| Dopamine transporter | D050483 | 4 | 10 | 16 | 1.60 |
| neurotrophic factor | D009414 | 9 | 11 | 16 | 1.45 |
| corticosterone | D003345 | 5 | 4 | 16 | 4.00 |
| noparticles | D053758 | 2 | 1 | 15 | 15.00 |
| balance | C0179199 | 4 | 11 | 15 | 1.36 |
| peptide | D010455 | 8 | 11 | 14 | 1.27 |
| CIT | C016308 | 10 | 13 | 14 | 1.08 |
| device | D004864 | 6 | 5 | 13 | 2.60 |
| implantation | C0021102 | 13 | 5 | 13 | 2.60 |
| transcranial direct current stimulation | D065908 | 0 | 3 | 12 | 4.00 |
| monoamine oxidase | D008995 | 6 | 6 | 12 | 2.00 |
| traumatic brain injury | D001930 | 1 | 3 | 12 | 4.00 |
| pramipexole | C061333 | 11 | 12 | 12 | 1.00 |
| rotigotine | C047508 | 8 | 6 | 12 | 2.00 |
| ropinirole | C046649 | 6 | 6 | 12 | 2.00 |
| sensors | C0183210 | 2 | 3 | 12 | 4.00 |
| carbidopa and levodopa | C009265 | 4 | 8 | 12 | 1.50 |
| gene therapy | D015316 | 8 | 12 | 11 | 0.92 |
| paraquat | D010269 | 8 | 3 | 11 | 3.67 |
| dance | D003614 | 1 | 1 | 11 | 11.00 |
| deprenyl | D012642 | 7 | 2 | 10 | 5.00 |
| glutathione | D005978 | 10 | 13 | 10 | 0.77 |
| apomorphine | D001058 | 7 | 4 | 10 | 2.50 |
| antioxidant | D000975 | 7 | 5 | 10 | 2.00 |
| Nrf2 | C0118522 | 1 | 6 | 10 | 1.67 |
| dopamine medication | C0013036 | 3 | 3 | 10 | 3.33 |
| synucleins | D051843 | 5 | 8 | 9 | 1.13 |
| serotonin | D012701 | 6 | 4 | 9 | 2.25 |
| pesticides | D010575 | 4 | 7 | 9 | 1.29 |
| lipopolysaccharide | D008070 | 3 | 5 | 9 | 1.80 |
| curcumin | D003474 | 4 | 7 | 9 | 1.29 |
| Caffeine | D002110 | 10 | 7 | 9 | 1.29 |
| acupuncture | D026881 | 5 | 5 | 8 | 1.60 |
| amyloid beta | C3484390 | 3 | 8 | 8 | 1.00 |
| microbiota | D064307 | 0 | 1 | 7 | 7.00 |
| transcranial magnetic stimulation | D050781 | 5 | 5 | 7 | 1.40 |
| glutamate | D018698 | 4 | 1 | 7 | 7.00 |
| vitamin d | D014807 | 1 | 3 | 7 | 2.33 |
| urate | D014527 | 3 | 8 | 7 | 0.88 |
| prions | D011328 | 1 | 7 | 7 | 1.00 |
| metformin | D008687 | 0 | 1 | 7 | 7.00 |
| lipids | D008055 | 2 | 9 | 7 | 0.78 |
| injection | D007267 | 3 | 2 | 7 | 3.50 |
| neurotoxic | C528652 | 4 | 6 | 7 | 1.17 |
| vanilloid | C528652 | 4 | 6 | 7 | 1.17 |
| statins | C086276 | 5 | 2 | 7 | 3.50 |
| lactacystin | C067713 | 6 | 1 | 7 | 7.00 |
| rasagiline | C031967 | 8 | 6 | 7 | 1.17 |
| PARP1 | C0032405 | 1 | 2 | 7 | 3.50 |
| paraquat | C0030493 | 2 | 3 | 7 | 2.33 |
| beta synuclein | D051846 | 2 | 3 | 6 | 2.00 |
| GDNF | D051100 | 5 | 6 | 6 | 1.00 |
| proteasome | D046988 | 5 | 8 | 6 | 0.75 |
| Interleukin6 | D015850 | 1 | 2 | 6 | 3 |
| ligands | D008024 | 4 | 4 | 6 | 1.50 |
| acetylcholine | D000109 | 6 | 10 | 6 | 0.60 |
| safimide | C092797 |  | 2 | 6 | 3.00 |
| coenzyme Q10 | C024989 | 6 | 3 | 6 | 2.00 |
| thalamotomy | C0195894 | 1 | 1 | 6 | 6.00 |
| Akt | C0164786 | 2 | 6 | 6 | 1.00 |
| baicalein | C006680 | 2 | 3 | 6 | 2.00 |
| PI3 kise | C0044602 | 1 | 4 | 6 | 1.50 |
| LRRK2 | C0031727 | 6 | 3 | 6 | 2.00 |
| PINK1 | C0031727 | 6 | 3 | 6 | 2.00 |
| cellbased therapies | D064987 | 1 | 5 | 5 | 1.00 |
| grafts | D019737 | 3 | 8 | 5 | 0.63 |
| BDNF | D019208 | 0 | 1 | 5 | 5.00 |
| dopamine agonists | D018491 | 8 | 9 | 5 | 0.56 |
| neurotransmitters | D018377 | 1 | 2 | 5 | 2.50 |
| electroacupuncture | D015671 |  | 4 | 5 | 1.25 |
| quercetin | D011794 | 3 | 4 | 5 | 1.25 |
| denervation | D003714 | 4 | 4 | 5 | 1.25 |
| ioflupane | C519528 | 3 | 8 | 5 | 0.63 |
| Pimavanserin | C510793 | 0 | 0 | 5 |  |
| CSF | C3540512 | 1 | 1 | 5 | 5.00 |
| istradefylline | C111599 | 4 | 2 | 5 | 2.50 |
| exendin4 | C074031 | 1 | 1 | 5 | 5.00 |
| Resveratrol | C059514 | 3 | 5 | 5 | 1 |
| MPP | C044202 | 6 | 1 | 5 | 5.00 |
| chemical | C0220806 | 1 | 4 | 5 | 1.25 |
| PDSS | C0175659 | 3 | 3 | 5 | 1.67 |
| neuromelanin | C014121 | 4 | 2 | 5 | 2.5 |
| stress | C0038435 | 1 | 0 | 5 |  |
| Long Noncoding R | D062085 |  |  | 4 |  |
| strength training | D055070 |  | 1 | 4 | 4 |
| miRs | D035683 |  | 1 | 4 | 4 |
| Dexmedetomidine | D020927 |  |  | 4 |  |
| program | D019542 | 2 | 2 | 4 | 2 |
| neuroprotective agents | D018696 |  | 1 | 4 | 4 |
| NFkappaB | D016328 | 2 | 7 | 4 | 0.57142857 |
| Nuclear factor kappaB | D016328 | 2 | 7 | 4 | 0.57142857 |
| nuclear factorkappa B | D016328 | 2 | 7 | 4 | 0.57142857 |
| nuclear factorkappaB | D016328 | 2 | 7 | 4 | 0.57142857 |
| valproate | D014635 | 1 | 1 | 4 | 4 |
| valproic acid | D014635 | 1 | 1 | 4 | 4 |
| TNF | D014409 | 3 | 4 | 4 | 1 |
| TNF alpha | D014409 | 3 | 4 | 4 | 1 |
| TNFalpha | D014409 | 3 | 4 | 4 | 1 |
| TNFalpha | D014409 | 3 | 4 | 4 | 1 |
| Tumor Necrosis Factor | D014409 | 3 | 4 | 4 | 1 |
| tumor necrosis factor alpha | D014409 | 3 | 4 | 4 | 1 |
| therapeutics | D013812 | 2 | 3 | 4 | 1.33333333 |
| task performance | D013647 | 3 | 2 | 4 | 2 |
| light therapy | D010789 |  |  | 4 |  |
| PSP | D010637 | 3 | 1 | 4 | 4 |
| excitotoxins | D009498 | 5 | 8 | 4 | 0.5 |
| methamphetamine | D008694 | 10 | 7 | 4 | 0.57142857 |
| metals | D008670 | 7 | 1 | 4 | 4 |
| Heme | D006418 | 3 | 4 | 4 | 1 |
| drugs | D004364 |  | 5 | 4 | 0.8 |
| pharmaceutical products | D004364 |  | 5 | 4 | 0.8 |
| copper | D003300 | 4 | 1 | 4 | 4 |
| cholesterol | D002784 | 1 | 1 | 4 | 4 |
| calcium | D002118 | 1 | 4 | 4 | 1 |
| Botulinum toxin | D001905 |  | 2 | 4 | 2 |
| amantadine | D000547 | 1 | 5 | 4 | 0.8 |
| Parkinson | C1021144 |  | 5 | 4 | 0.8 |
| Parkinsons | C1021144 |  | 5 | 4 | 0.8 |
| PD | C1021144 |  | 5 | 4 | 0.8 |
| PPMI | C1021144 |  | 5 | 4 | 0.8 |
| entacapone | C071192 | 12 | 2 | 4 | 2 |
| proteincoupled receptors | C0597357 |  |  | 4 |  |
| RXRalpha | C0597357 |  |  | 4 |  |
| TrkB | C0597357 |  |  | 4 |  |
| TRPC6 | C0597357 |  |  | 4 |  |
| enriched | C0359583 | 2 |  | 4 |  |
| chaperones | C0243041 | 6 | 7 | 4 | 0.57142857 |
| zonisamide | C022189 | 1 | 4 | 4 | 1 |
| accelerometer | C0178951 | 1 |  | 4 |  |
| game | C0150593 |  | 1 | 4 | 4 |
| D2 Receptors | C0058698 |  |  | 4 |  |
| D2R | C0058698 |  |  | 4 |  |
| dopamine D2/D3 receptors | C0058698 |  |  | 4 |  |
| DRD2 | C0058698 |  |  | 4 |  |
| insertion | C0021107 |  |  | 4 |  |
| polyphenols | D059808 | 3 |  | 3 |  |
| AMPK | D055372 |  |  | 3 |  |
| Ghrelin | D054439 | 1 | 1 | 3 | 3 |
| caspase3 | D053148 | 1 | 1 | 3 | 3 |
| Mucu | D031299 | 1 | 1 | 3 | 3 |
| calorie restriction | D031204 |  |  | 3 |  |
| Centella asiatica | D028041 |  | 1 | 3 | 3 |
| ubiquitin | D025801 | 4 | 5 | 3 | 0.6 |
| dopamine D2 receptor | D017448 | 1 | 1 | 3 | 3 |
| dopamine D2 receptors | D017448 | 1 | 1 | 3 | 3 |
| Isradipine | D017275 |  |  | 3 |  |
| tau protein | D016875 |  | 1 | 3 | 3 |
| tau proteins | D016875 |  | 1 | 3 | 3 |
| Gamma knife radiosurgery | D016634 |  |  | 3 |  |
| radiosurgery | D016634 |  |  | 3 |  |
| Stereotactic radiosurgery | D016634 |  |  | 3 |  |
| TGFbeta | D016212 | 1 |  | 3 |  |
| TGFbeta | D016212 | 1 |  | 3 |  |
| Transforming growth factor beta | D016212 | 1 |  | 3 |  |
| Transforming Growth Factor beta2 | D016212 | 1 |  | 3 |  |
| cytokine | D016207 | 2 | 8 | 3 | 0.375 |
| Cytokines | D016207 | 2 | 8 | 3 | 0.375 |
| NMDA | D016202 | 4 | 10 | 3 | 0.3 |
| Droxidopa | D015103 |  |  | 3 |  |
| ATRA | D014212 |  | 1 | 3 | 3 |
| RA | D014212 |  | 1 | 3 | 3 |
| retinoic acid | D014212 |  | 1 | 3 | 3 |
| Trehalose | D014199 |  | 2 | 3 | 1.5 |
| Ser | D012694 | 3 | 6 | 3 | 0.5 |
| serine | D012694 | 3 | 6 | 3 | 0.5 |
| SAR | D012521 | 1 | 2 | 3 | 1.5 |
| Rifampicin | D012293 | 1 |  | 3 |  |
| Rifampin | D012293 | 1 |  | 3 |  |
| nicotine | D009538 | 4 | 3 | 3 | 1 |
| acid amide | D009536 | 2 | 2 | 3 | 1.5 |
| nicotimide | D009536 | 2 | 2 | 3 | 1.5 |
| vitamin B | D009536 | 2 | 2 | 3 | 1.5 |
| Vitamin B3 | D009536 | 2 | 2 | 3 | 1.5 |
| microparticles | D008863 | 1 | 4 | 3 | 0.75 |
| microspheres | D008863 | 1 | 4 | 3 | 0.75 |
| Melatonin | D008550 | 3 | 6 | 3 | 0.5 |
| insulin | D007328 | 1 |  | 3 |  |
| Immunotherapies | D007167 |  |  | 3 |  |
| Immunotherapy | D007167 |  |  | 3 |  |
| Hydrogen | D006859 |  | 1 | 3 | 3 |
| hydrogen gas | D006859 |  | 1 | 3 | 3 |
| Molecular hydrogen | D006859 |  | 1 | 3 | 3 |
| GTP | D006160 |  | 1 | 3 | 3 |
| gold noparticles | D006046 |  | 1 | 3 | 3 |
| glycogen | D006003 | 2 | 2 | 3 | 1.5 |
| buccal | D005996 | 1 | 2 | 3 | 1.5 |
| nitroglycerin | D005996 | 1 | 2 | 3 | 1.5 |
| fluoro | D005461 | 1 | 1 | 3 | 3 |
| docosahexaenoic acid | D004281 | 1 | 3 | 3 | 1 |
| Mitochondrial D | D004272 | 2 | 4 | 3 | 0.75 |
| mtD | D004272 | 2 | 4 | 3 | 0.75 |
| deferoxamine | D003676 |  | 2 | 3 | 1.5 |
| desferrioxamine | D003676 |  | 2 | 3 | 1.5 |
| desferrioxamine B | D003676 |  | 2 | 3 | 1.5 |
| DFOB | D003676 |  | 2 | 3 | 1.5 |
| cysteamine | D003543 |  |  | 3 |  |
| creatine | D003401 | 4 | 1 | 3 | 3 |
| Ceftriaxone | D002443 |  | 1 | 3 | 3 |
| catecholamine | D002395 | 2 | 2 | 3 | 1.5 |
| catecholamines | D002395 | 2 | 2 | 3 | 1.5 |
| catechol Omethyltransferase | D002394 | 1 | 3 | 3 | 1 |
| CatecholOmethyltransferase | D002394 | 1 | 3 | 3 | 1 |
| butyrate | D002087 |  | 1 | 3 | 3 |
| Benserazide | D001545 |  | 4 | 3 | 0.75 |
| autoantibodies | D001323 |  | 1 | 3 | 3 |
| aspartate | D001224 | 2 | 3 | 3 | 1 |
| ascorbate | D001205 | 3 | 3 | 3 | 1 |
| ascorbic acid | D001205 | 3 | 3 | 3 | 1 |
| Vitamin C | D001205 | 3 | 3 | 3 | 1 |
| antibodies | D000906 | 2 |  | 3 |  |
| Intrasal administration | D000281 |  | 1 | 3 | 3 |
| adenosine triphosphate | D000255 | 3 | 7 | 3 | 0.42857143 |
| ATP | D000255 | 3 | 7 | 3 | 0.42857143 |
| preladent | C539997 |  | 1 | 3 | 3 |
| MK 3814 | C539997 |  | 1 | 3 | 3 |
| SCH420814 | C539997 |  | 1 | 3 | 3 |
| Photobiomodulation | C4019433 |  | 1 | 3 | 3 |
| NET | C3853572 |  | 1 | 3 | 3 |
| NETPD | C3853572 |  | 1 | 3 | 3 |
| nets | C3853572 |  | 1 | 3 | 3 |
| MAOB inhibitors | C3653412 | 1 | 3 | 3 | 1 |
| monoamine oxidase B inhibitors | C3653412 | 1 | 3 | 3 | 1 |
| monoamine oxidase type B inhibitors | C3653412 | 1 | 3 | 3 | 1 |
| transport | C3495449 | 2 | 2 | 3 | 1.5 |
| lncRs | C3494264 |  |  | 3 |  |
| Long Noncoding Rs | C3494264 |  |  | 3 |  |
| long noncoding Rs | C3494264 |  |  | 3 |  |
| level | C2946261 | 1 |  | 3 |  |
| basis | C1874451 | 2 | 1 | 3 | 3 |
| stabilisation | C1293130 |  |  | 3 |  |
| stabilization | C1293130 |  |  | 3 |  |
| DMT1 | C0596902 | 2 | 5 | 3 | 0.6 |
| SERT | C0596902 | 2 | 5 | 3 | 0.6 |
| transporter | C0596902 | 2 | 5 | 3 | 0.6 |
| transporters | C0596902 | 2 | 5 | 3 | 0.6 |
| MAOB Inhibitor | C0595265 |  |  | 3 |  |
| Murine | C0591833 |  | 4 | 3 | 0.75 |
| hydroxyl | C031356 | 5 | 6 | 3 | 0.5 |
| Abl | C030358 |  |  | 3 |  |
| The C | C023665 |  | 2 | 3 | 1.5 |
| A 7 | C020846 |  |  | 3 |  |
| ground | C0185051 |  | 1 | 3 | 3 |
| Tetramethylpyrazine | C017953 |  |  | 3 |  |
| band | C0175723 | 1 | 2 | 3 | 1.5 |
| bands | C0175723 | 1 | 2 | 3 | 1.5 |
| dopamine transporters | C0114838 | 1 |  | 3 |  |
| DAT SPECT | C0114838 | 1 |  | 3 |  |
| DATSPECT | C0114838 | 1 |  | 3 |  |
| piperine | C008922 |  | 1 | 3 | 3 |
| GBA | C0017768 |  |  | 3 |  |
| lysosomal glucocerebrosidase | C0017768 |  |  | 3 |  |
| chi | C000536 | 2 | 1 | 3 | 3 |
| medical canbis | D064086 |  |  | 2 |  |
| Spil cord stimulation | D062187 |  | 1 | 2 | 2 |
| persolized medicine | D057285 |  | 1 | 2 | 2 |
| Sirtuin 2 | D056565 |  | 2 | 2 | 1 |
| inducible nitric oxide synthase | D052247 |  |  | 2 |  |
| Dopamine D3 receptor | D050637 |  |  | 2 |  |
| 1433 protein | D048948 |  |  | 2 |  |
| Luteolin | D047311 | 1 | 1 | 2 | 2 |
| apigenin | D047310 |  |  | 2 |  |
| notechnology | D036103 |  |  | 2 |  |
| flowers | D035264 |  |  | 2 |  |
| HMGB1 | D024243 |  |  | 2 |  |
| mitochondrial protein | D024101 |  | 1 | 2 | 2 |
| Ttype calcium channel | D020747 | 1 |  | 2 |  |
| caspase | D020169 |  | 1 | 2 | 2 |
| hydrogel | D020136 | 1 |  | 2 |  |
| Simvastatin | D019821 | 1 | 1 | 2 | 2 |
| Prosthesis | D019736 |  |  | 2 |  |
| Botulinum toxin type A | D019274 |  |  | 2 |  |
| DPH oxidase | D019255 |  | 2 | 2 | 1 |
| IL13 | D018793 |  |  | 2 |  |
| sodium selenite | D018038 |  |  | 2 |  |
| Lithium chloride | D018021 |  |  | 2 |  |
| protein kise A | D017868 |  |  | 2 |  |
| Dopamine D1 Receptor | D017447 |  |  | 2 |  |
| IL10 | D016753 | 1 | 1 | 2 | 2 |
| Interleukin10 | D016753 | 1 | 1 | 2 | 2 |
| Fluvoxamine maleate | D016666 |  |  | 2 |  |
| cognitive behavior therapy | D015928 |  | 4 | 2 | 0.5 |
| Cognitive behavioral therapy | D015928 |  | 4 | 2 | 0.5 |
| cognitivebehavioral therapy | D015928 |  | 4 | 2 | 0.5 |
| Cognitivebehavioural therapy | D015928 |  | 4 | 2 | 0.5 |
| Computerized Cognitive behavioral therapy | D015928 |  | 4 | 2 | 0.5 |
| IL4 | D015847 | 1 |  | 2 |  |
| Interleukin 4 | D015847 | 1 |  | 2 |  |
| Interleukin4 | D015847 | 1 |  | 2 |  |
| propofol | D015742 |  |  | 2 |  |
| water | D014867 |  |  | 2 |  |
| retinol | D014801 |  |  | 2 |  |
| Vitamin A | D014801 |  |  | 2 |  |
| Rab | D014740 | 1 |  | 2 |  |
| tubulin | D014404 |  | 1 | 2 | 2 |
| transcription factor | D014157 | 1 | 4 | 2 | 0.5 |
| transcription factors | D014157 | 1 | 4 | 2 | 0.5 |
| antipsychotic drugs | D014150 |  | 3 | 2 | 0.66666667 |
| antipsychotics | D014150 |  | 3 | 2 | 0.66666667 |
| neuroleptic drugs | D014150 |  | 3 | 2 | 0.66666667 |
| Thiamine | D013831 |  |  | 2 |  |
| streptozotocin | D013311 |  |  | 2 |  |
| SDS | D012967 |  |  | 2 |  |
| reserpine | D012110 | 3 | 2 | 2 | 1 |
| RAGE | D011889 |  |  | 2 |  |
| QSM | D011803 |  |  | 2 |  |
| Fenofibrate | D011345 |  |  | 2 |  |
| probenecid | D011339 | 1 | 2 | 2 | 1 |
| Polyvinylpyrrolidone | D011205 |  |  | 2 |  |
| PVP | D011205 |  |  | 2 |  |
| PEI | D011094 |  |  | 2 |  |
| polyethylenimine | D011094 |  |  | 2 |  |
| medicil herbs | D010946 |  |  | 2 |  |
| Medicil plants | D010946 |  |  | 2 |  |
| piribedil | D010891 | 1 |  | 2 |  |
| phospholipid | D010743 | 3 | 2 | 2 | 1 |
| phospholipids | D010743 | 3 | 2 | 2 | 1 |
| APAs | D010131 |  |  | 2 |  |
| PAS | D010131 |  |  | 2 |  |
| noradreline | D009638 | 1 | 7 | 2 | 0.28571429 |
| norepinephrine | D009638 | 1 | 7 | 2 | 0.28571429 |
| NFL | D009544 |  |  | 2 |  |
| DP | D009249 | 1 | 1 | 2 | 2 |
| DPH | D009249 | 1 | 1 | 2 | 2 |
| myeloperoxidase | D009195 | 1 | 1 | 2 | 2 |
| peroxidase | D009195 | 1 | 1 | 2 | 2 |
| minocycline | D008911 | 1 | 2 | 2 | 1 |
| microelectrode | D008839 | 2 | 3 | 2 | 0.66666667 |
| Maneb | D008344 | 3 | 4 | 2 | 0.5 |
| lipoic acid | D008063 |  | 1 | 2 | 2 |
| Laminin | D007797 | 1 | 1 | 2 | 2 |
| kynurenine | D007737 |  |  | 2 |  |
| Kinetin | D007701 |  |  | 2 |  |
| KA | D007608 | 2 |  | 2 |  |
| kainic acid | D007608 | 2 |  | 2 |  |
| Isatin | D007510 |  |  | 2 |  |
| praxis | D007216 |  |  | 2 |  |
| H2S | D006862 |  | 3 | 2 | 0.66666667 |
| hydrogen sulfide | D006862 |  | 3 | 2 | 0.66666667 |
| hydrogen sulphide | D006862 |  | 3 | 2 | 0.66666667 |
| Homocysteine | D006710 | 2 | 7 | 2 | 0.28571429 |
| hemoglobin | D006454 | 1 | 1 | 2 | 2 |
| harmine | D006247 |  | 1 | 2 | 2 |
| harmaline | D006246 |  | 1 | 2 | 2 |
| Graphene | D006108 |  |  | 2 |  |
| Aldose | D005985 |  | 1 | 2 | 2 |
| glyceraldehyde | D005985 |  | 1 | 2 | 2 |
| Glutamine | D005973 |  |  | 2 |  |
| betaglucocerebrosidase | D005962 |  | 2 | 2 | 1 |
| glucocerebrosidase | D005962 |  | 2 | 2 | 1 |
| Genetic counseling | D005817 |  |  | 2 |  |
| gastrostomies | D005774 |  |  | 2 |  |
| gastrostomy | D005774 |  |  | 2 |  |
| freshwater | D005618 |  |  | 2 |  |
| food | D005502 | 1 |  | 2 |  |
| Folate | D005492 | 1 | 2 | 2 | 1 |
| folic acid | D005492 | 1 | 2 | 2 | 1 |
| flavonoid | D005419 |  | 2 | 2 | 1 |
| flavonoids | D005419 |  | 2 | 2 | 1 |
| Glasses | D005139 |  |  | 2 |  |
| exon | D005091 |  | 3 | 2 | 0.66666667 |
| estradiol | D004958 |  | 1 | 2 | 2 |
| Epidermal Growth Factor | D004815 |  |  | 2 |  |
| Ellagic acid | D004610 | 1 | 1 | 2 | 2 |
| polyphenolic | D004610 | 1 | 1 | 2 | 2 |
| electrode | D004566 | 2 | 2 | 2 | 1 |
| electrodes | D004566 | 2 | 2 | 2 | 1 |
| electrical stimulation | D004558 | 3 | 5 | 2 | 0.4 |
| Electrical stimulation therapies | D004558 | 3 | 5 | 2 | 0.4 |
| dihydroxyphenylalanine | D004295 | 1 | 3 | 2 | 0.66666667 |
| diet | D004032 |  | 1 | 2 | 2 |
| Diets | D004032 |  | 1 | 2 | 2 |
| dieldrin | D004026 | 3 | 1 | 2 | 2 |
| cysteine | D003545 | 1 | 2 | 2 | 1 |
| Lcysteine | D003545 | 1 | 2 | 2 | 1 |
| coffee | D003069 | 3 | 2 | 2 | 1 |
| Chlorpromazine | D002746 |  |  | 2 |  |
| ceruloplasmin | D002570 | 2 | 2 | 2 | 1 |
| Carboline | D002243 |  |  | 2 |  |
| Capsaicin | D002211 |  |  | 2 |  |
| Canbis | D002188 |  |  | 2 |  |
| Marijua | D002188 |  |  | 2 |  |
| Aspirin | D001241 |  | 1 | 2 | 2 |
| ECM | D001241 |  | 1 | 2 | 2 |
| ApoE | D001057 | 2 | 2 | 2 | 1 |
| Local Anesthesia | D000772 |  |  | 2 |  |
| aminopyridine | D000631 |  |  | 2 |  |
| aminobutyric acid | D000613 |  | 2 | 2 | 1 |
| alcohol | D000438 |  | 2 | 2 | 1 |
| ethanol | D000431 | 1 |  | 2 |  |
| ATPase | D000251 | 1 | 3 | 2 | 0.66666667 |
| Acrolein | D000171 |  |  | 2 |  |
| Glcc | D000117 |  | 1 | 2 | 2 |
| acetylcysteine | D000111 | 3 | 1 | 2 | 2 |
| C | D000111 | 3 | 1 | 2 | 2 |
| Opicapone | C549349 |  |  | 2 |  |
| DOPAL | C539838 | 1 |  | 2 |  |
| Morroniside | C488401 |  |  | 2 |  |
| GSK3beta | C448280 |  |  | 2 |  |
| liraglutide | C439759 |  |  | 2 |  |
| Nexus | C433206 |  |  | 2 |  |
| TO901317 | C423915 |  |  | 2 |  |
| tozadent | C3886388 |  |  | 2 |  |
| PGK1 | C3814452 |  |  | 2 |  |
| Phosphoglycerate Kise 1 | C3814452 |  |  | 2 |  |
| tonic | C3543842 | 1 |  | 2 |  |
| Blockade | C3540676 |  | 2 | 2 | 1 |
| braincomputer interfacing | C3494288 |  |  | 2 |  |
| Brain Computer Interfacing | C3494288 |  |  | 2 |  |
| Adapted | C2937289 |  |  | 2 |  |
| sirtuin2 | C2720168 |  | 1 | 2 | 2 |
| cortex | C2700441 | 4 | 2 | 2 | 1 |
| tural product | C1566558 |  | 1 | 2 | 2 |
| injected | C1533685 |  |  | 2 |  |
| p300 | C1530358 |  | 1 | 2 | 2 |
| JAK2STAT3 | C1527617 |  |  | 2 |  |
| RBD | C1522002 |  |  | 2 |  |
| RBD | C1522002 |  |  | 2 |  |
| slice | C1519355 | 3 | 2 | 2 | 1 |
| slices | C1519355 | 3 | 2 | 2 | 1 |
| botulinum neurotoxin | C1295507 |  | 1 | 2 | 2 |
| exploration | C1280903 | 2 | 1 | 2 | 2 |
| explorations | C1280903 | 2 | 1 | 2 | 2 |
| Care Delivery | C1171257 |  |  | 2 |  |
| Klotho | C1144173 |  |  | 2 |  |
| Hypocretin | C110857 |  | 1 | 2 | 2 |
| orexin | C110857 |  | 1 | 2 | 2 |
| Orexin a | C110857 |  | 1 | 2 | 2 |
| OrexinA | C110857 |  | 1 | 2 | 2 |
| PLK2 | C1100824 |  |  | 2 |  |
| Pololike kise 2 | C1100824 |  |  | 2 |  |
| metabotropic glutamate receptor 4 | C108823 | 1 | 1 | 2 | 2 |
| metabotropic glutamate receptors 4 | C108823 | 1 | 1 | 2 | 2 |
| Mayo | C1077578 |  |  | 2 |  |
| desmethoxyfallypride | C100958 |  | 1 | 2 | 2 |
| DMFP | C100958 |  | 1 | 2 | 2 |
| fingolimod | C098720 |  |  | 2 |  |
| FTY720 | C098720 |  |  | 2 |  |
| HDAC6 | C0969119 |  |  | 2 |  |
| migalastat | C090092 |  |  | 2 |  |
| HCl | C089399 | 1 |  | 2 |  |
| Cognitive rehabilitation | C0870303 |  | 2 | 2 | 1 |
| brain stimulation | C0870227 | 1 | 1 | 2 | 2 |
| molybdenum disulfide | C082964 |  |  | 2 |  |
| MoS2 | C082964 |  |  | 2 |  |
| donepezil | C076946 | 1 | 3 | 2 | 0.66666667 |
| ultra | C0728475 | 1 | 1 | 2 | 2 |
| ultra | C0728475 | 1 | 1 | 2 | 2 |
| Ultra high | C0728475 | 1 | 1 | 2 | 2 |
| rivastigmine | C072506 | 3 | 1 | 2 | 2 |
| treatment option | C0683525 | 2 | 2 | 2 | 1 |
| Treatment options | C0683525 | 2 | 2 | 2 | 1 |
| Psychosocial interventions | C0683471 |  |  | 2 |  |
| Psychosocial therapy | C0683471 |  |  | 2 |  |
| adjunct therapy | C0677850 |  |  | 2 |  |
| adjunctive therapy | C0677850 |  |  | 2 |  |
| Adjuvant Therapy | C0677850 |  |  | 2 |  |
| Held | C0675390 |  |  | 2 |  |
| eltoprazine | C063828 |  |  | 2 |  |
| echicoside | C060297 |  | 1 | 2 | 2 |
| isoform | C0597298 |  | 3 | 2 | 0.66666667 |
| protein kise C isoforms | C0597298 |  | 3 | 2 | 0.66666667 |
| Hip Fracture Surgery | C0596706 |  |  | 2 |  |
| Loganin | C059516 |  |  | 2 |  |
| Tangeretin | C059006 |  |  | 2 |  |
| tangeritin | C059006 |  |  | 2 |  |
| duloxetine | C058218 |  | 1 | 2 | 2 |
| structural changes | C0581602 |  | 1 | 2 | 2 |
| Glucagonlike Peptide1 Receptor | C053432 |  |  | 2 |  |
| YKL40 | C0528649 |  |  | 2 |  |
| Isorhynchophylline | C052714 |  | 1 | 2 | 2 |
| reconstruction | C0524865 |  | 1 | 2 | 2 |
| Astragaloside IV | C052064 |  | 1 | 2 | 2 |
| deferiprone | C044919 | 1 |  | 2 |  |
| electroencephalographic | C0443310 |  |  | 2 |  |
| sponging | C0441126 |  |  | 2 |  |
| ultrafine | C043595 | 1 |  | 2 |  |
| oxyresveratrol | C034912 | 1 |  | 2 |  |
| Tetrahydroxystilbene | C034912 | 1 |  | 2 |  |
| CRS | C032538 |  |  | 2 |  |
| Tauroursodeoxycholic acid | C031655 |  | 1 | 2 | 2 |
| manganese chloride | C025340 |  | 1 | 2 | 2 |
| MnCl2 | C025340 |  | 1 | 2 | 2 |
| Cdk5 | C0249586 |  | 2 | 2 | 1 |
| cyclindependent kise5 | C0249586 |  | 2 | 2 | 1 |
| Hsp70 | C0243043 | 5 |  | 2 |  |
| Nutritiol Supplements | C0242295 |  |  | 2 |  |
| Supplement | C0242295 |  |  | 2 |  |
| Prevention | C0199176 |  | 5 | 2 | 0.4 |
| Prophylaxis | C0199176 |  | 5 | 2 | 0.4 |
| 34a | C018691 |  |  | 2 |  |
| Spil Surgery | C0185908 |  |  | 2 |  |
| extraction | C0185115 | 1 | 1 | 2 | 2 |
| removal | C0185115 | 1 | 1 | 2 | 2 |
| folding | C0185026 | 1 | 1 | 2 | 2 |
| carnosic acid | C018381 | 1 | 1 | 2 | 2 |
| asiatic acid | C017032 |  |  | 2 |  |
| apraclonidine | C016986 |  |  | 2 |  |
| CpG | C015772 |  |  | 2 |  |
| Asarone | C012195 |  |  | 2 |  |
| algite | C008970 |  |  | 2 |  |
| drug target | C0085104 | 1 | 1 | 2 | 2 |
| drug targets | C0085104 | 1 | 1 | 2 | 2 |
| targeteddrug | C0085104 | 1 | 1 | 2 | 2 |
| walk | C0080331 | 1 | 1 | 2 | 2 |
| Dityrosine | C007543 |  |  | 2 |  |
| + /H+ Exchanger | C0074785 |  |  | 2 |  |
| +/H+ Exchanger | C0074785 |  |  | 2 |  |
| linked protein | C0065011 |  |  | 2 |  |
| HMGCR | C0063164 |  |  | 2 |  |
| sulfoxide | C005746 | 1 |  | 2 |  |
| sham | C005703 | 1 | 3 | 2 | 0.66666667 |
| Ursolic acid | C005466 |  |  | 2 |  |
| brilliant blue G | C004692 |  |  | 2 |  |
| CCL2 | C004260 | 1 |  | 2 |  |
| STAT3 | C0040661 |  |  | 2 |  |
| antiParkinson agents | C0040616 |  |  | 2 |  |
| AntiParkinsonian Agents | C0040616 |  |  | 2 |  |
| thiol | C0038734 | 1 | 1 | 2 | 2 |
| thiol | C0038734 | 1 | 1 | 2 | 2 |
| Sulfated | C0038720 |  |  | 2 |  |
| Sulphated | C0038720 |  |  | 2 |  |
| mRs | C0035696 |  |  | 2 |  |
| Dopamine D2L receptorinteracting proteins | C0034798 |  |  | 2 |  |
| Dopamine receptor interacting proteins | C0034798 |  |  | 2 |  |
| dopaminergic D2 receptors | C0034798 |  |  | 2 |  |
| dopaminergic D3 receptors | C0034798 |  |  | 2 |  |
| dopaminergic receptors | C0034798 |  |  | 2 |  |
| Thymoquinone | C003466 |  |  | 2 |  |
| gene product | C0033684 | 1 |  | 2 |  |
| LRRK2 | C0033684 | 1 |  | 2 |  |
| VPS35 | C0033684 | 1 |  | 2 |  |
| PLA2G6 | C0031667 |  |  | 2 |  |
| protease | C0030946 |  | 1 | 2 | 2 |
| Physical Behavior | C0026606 |  | 1 | 2 | 2 |
| motor & reward seeking behavior | C0026606 |  | 1 | 2 | 2 |
| motor coordition behaviors | C0026606 |  | 1 | 2 | 2 |
| intronic | C0021920 | 1 | 1 | 2 | 2 |
| HLA | C0019629 |  |  | 2 |  |
| human leukocyte antigen | C0019629 |  |  | 2 |  |
| Hsp70 | C0018850 | 2 |  | 2 |  |
| stress response protein | C0018850 | 2 |  | 2 |  |
| stress response proteins | C0018850 | 2 |  | 2 |  |
| stress sigling protein | C0018850 | 2 |  | 2 |  |
| betaNmethylaminoLalanine | C001824 | 2 | 1 | 2 | 2 |
| BMAA | C001824 | 2 | 1 | 2 | 2 |
| GP78 | C0017968 |  |  | 2 |  |
| GPNMB | C0017968 |  |  | 2 |  |
| GFAP | C0017626 | 1 | 1 | 2 | 2 |
| GPER | C0014939 |  | 1 | 2 | 2 |
| oestrogen | C0014939 |  | 1 | 2 | 2 |
| dietary | C0012155 | 3 | 3 | 2 | 0.66666667 |
| combined SSRI antipsychotic treatment | C0009429 |  |  | 2 |  |
| combined treatment | C0009429 |  |  | 2 |  |
| deutetrabezine | C000609690 |  |  | 2 |  |
| Electronic Cigarette | D066300 |  |  | 1 |  |
| Ammonium | D064751 |  |  | 1 |  |
| alpha7ChR | D064569 |  |  | 1 |  |
| hepcidin | D064451 |  |  | 1 |  |
| unstructured proteins | D064267 | 1 | 1 | 1 | 1 |
| endocanbinoid | D063388 |  | 3 | 1 | 0.33333333 |
| transdermal nicotine | D061485 |  |  | 1 |  |
| neurofeedback | D058765 |  |  | 1 |  |
| islet amyloid polypeptide | D058228 |  | 2 | 1 | 0.5 |
| amyloid proteins | D058227 |  | 1 | 1 | 1 |
| transdermal patch | D057968 | 1 |  | 1 |  |
| fiducial markers | D057918 |  |  | 1 |  |
| Cathepsin L | D056668 |  |  | 1 |  |
| Sirtuin 3 | D056566 |  |  | 1 |  |
| chaperonin CCT | D056404 |  |  | 1 |  |
| prescription drugs | D055553 |  |  | 1 |  |
| volatile organic compounds | D055549 |  |  | 1 |  |
| vagus nerve stimulation | D055536 |  |  | 1 |  |
| growth Differentiation Factor 15 | D055436 |  |  | 1 |  |
| Incretin | D054795 |  |  | 1 |  |
| CaMKII | D054732 |  | 1 | 1 | 1 |
| phosphodiesterase 4 | D054703 |  |  | 1 |  |
| Protein Tyrosine Phosphatase 1B | D054562 |  |  | 1 |  |
| Secretory Phospholipase A2 | D054497 |  |  | 1 |  |
| rho kise | D054460 |  | 2 | 1 | 0.5 |
| perforin | D054353 |  |  | 1 |  |
| pallidotomy | D053860 | 4 |  | 1 |  |
| TGFbeta1 | D053773 | 1 |  | 1 |  |
| syndecans | D053667 |  |  | 1 |  |
| Caspase7 | D053179 |  |  | 1 |  |
| particulate matter | D052638 |  |  | 1 |  |
| Benzothiazoles | D052160 |  |  | 1 |  |
| gammasynuclein | D051845 | 1 | 2 | 1 | 0.5 |
| Olfactory Marker Protein | D051842 |  |  | 1 |  |
| aquaporin 4 | D051401 |  | 1 | 1 | 1 |
| Aquaporin 1 | D051398 |  |  | 1 |  |
| Cyclindependent kise 5 | D051360 |  |  | 1 |  |
| Toll Like Receptor 4 | D051197 |  | 1 | 1 | 1 |
| Tolllike receptor 2 | D051195 |  |  | 1 |  |
| betacatenin | D051176 |  |  | 1 |  |
| SRE | D050600 |  | 1 | 1 | 1 |
| Vesicular monoamine transporter | D050493 | 2 | 3 | 1 | 0.33333333 |
| serotonin transporter | D050486 |  | 1 | 1 | 1 |
| Lon protease | D049070 |  |  | 1 |  |
| Chitosan | D048271 |  |  | 1 |  |
| p38 MAPK | D048051 | 2 | 1 | 1 | 1 |
| cJun Ntermil kise | D048031 |  |  | 1 |  |
| Peroxisome proliferatoractivated receptors | D047492 | 2 |  | 1 |  |
| cytochrome c | D045304 | 1 | 2 | 1 | 0.5 |
| triuretic Peptide | D045265 |  |  | 1 |  |
| Fbox Protein | D044783 |  |  | 1 |  |
| tetrahydroisoquinoline | D044005 | 4 | 1 | 1 | 1 |
| Lipoproteinassociated phospholipase A2 | D043203 |  |  | 1 |  |
| Mediterranean diet | D038441 | 1 | 1 | 1 | 1 |
| GSK3 | D038362 |  |  | 1 |  |
| growth factors | D036341 | 1 |  | 1 |  |
| auxilin | D034021 |  |  | 1 |  |
| Withania somnifera | D032341 |  |  | 1 |  |
| Veronica | D032264 |  |  | 1 |  |
| Morinda citrifolia | D032066 |  |  | 1 |  |
| olive | D031658 |  |  | 1 |  |
| teucrium | D031367 |  |  | 1 |  |
| Scutellaria | D031363 |  |  | 1 |  |
| perilla | D031343 |  |  | 1 |  |
| safflower | D031187 |  |  | 1 |  |
| Curcuma | D030024 |  |  | 1 |  |
| Pueraria lobata | D029908 |  |  | 1 |  |
| Activin | D028341 |  |  | 1 |  |
| Avocado | D027421 |  |  | 1 |  |
| glutamate transporter | D027322 | 1 |  | 1 |  |
| Urtica dioica | D026982 |  |  | 1 |  |
| Osteopathic manipulation | D026301 |  |  | 1 |  |
| manipulation therapy | D026201 |  |  | 1 |  |
| singlet oxygen | D026082 |  |  | 1 |  |
| permethrin | D026023 |  |  | 1 |  |
| Merlin | D025581 |  |  | 1 |  |
| tocopherol | D024505 |  |  | 1 |  |
| tissue engineering | D023822 |  |  | 1 |  |
| rolipram | D020889 | 1 |  | 1 |  |
| Matrix Metalloproteise2 | D020778 |  |  | 1 |  |
| proteome | D020543 | 5 | 5 | 1 | 0.2 |
| Pluronic F68 | D020442 | 1 |  | 1 |  |
| Poloxamer 188 | D020442 | 1 |  | 1 |  |
| IL7 receptor | D020395 |  |  | 1 |  |
| Pglycoprotein | D020168 |  |  | 1 |  |
| betahydroxybutyrate | D020155 |  | 1 | 1 | 1 |
| Rapamycin | D020123 | 2 | 3 | 1 | 0.33333333 |
| Nitric Oxide Donors | D020030 |  |  | 1 |  |
| calcineurin | D019703 |  | 1 | 1 | 1 |
| maps | D019532 |  |  | 1 |  |
| CYP2D6 | D019389 | 1 | 1 | 1 | 1 |
| Cytochrome P450 2D6 | D019389 | 1 | 1 | 1 | 1 |
| Copper sulfate | D019327 |  |  | 1 |  |
| DNP | D019297 |  |  | 1 |  |
| pyruvate | D019289 |  |  | 1 |  |
| Gprotein | D019204 | 1 |  | 1 |  |
| NCAM | D019006 |  |  | 1 |  |
| chemokines | D018925 |  | 1 | 1 | 1 |
| Heat Shock Protein 70 | D018840 |  |  | 1 |  |
| molecular chaperones | D018832 |  | 3 | 1 | 0.33333333 |
| intercellular adhesion molecule1 | D018799 |  |  | 1 |  |
| Nutritiol Support | D018529 |  |  | 1 |  |
| early intervention | D018479 |  | 1 | 1 | 1 |
| AMPA | D018350 | 1 | 2 | 1 | 0.5 |
| Fisteride | D018120 |  |  | 1 |  |
| LTD | D017998 |  | 2 | 1 | 0.5 |
| Parasympathetic Denervation | D017764 |  |  | 1 |  |
| Duvoglustat | D017485 |  |  | 1 |  |
| Delta Opioid Receptor | D017465 | 1 |  | 1 |  |
| delta opioid receptors | D017465 | 1 |  | 1 |  |
| delta receptors | D017465 | 1 |  | 1 |  |
| Advanced glycation end products | D017127 | 1 |  | 1 |  |
| Interferonbeta | D016899 |  |  | 1 |  |
| apolipoprotein A1 | D016632 |  |  | 1 |  |
| Astemizole | D016589 |  |  | 1 |  |
| Antisense Oligonucleotides | D016376 |  |  | 1 |  |
| Dizocilpine | D016291 |  | 1 | 1 | 1 |
| MK801 | D016291 |  | 1 | 1 | 1 |
| endothelial growth factor | D016228 |  | 1 | 1 | 1 |
| benzoquinones | D016227 |  |  | 1 |  |
| IL8 | D016209 |  |  | 1 |  |
| Colony Stimulating Factor Receptor | D016184 |  |  | 1 |  |
| GCSF | D016179 |  |  | 1 |  |
| Granulocyte ColonyStimulating Factor | D016179 |  |  | 1 |  |
| PCR | D016133 | 1 | 1 | 1 | 1 |
| Dideoxycytidine | D016047 |  |  | 1 |  |
| integrins | D016023 | 1 |  | 1 |  |
| cystatin | D015891 |  |  | 1 |  |
| ionomycin | D015759 |  |  | 1 |  |
| Calcitonin GeneRelated Peptide | D015740 |  |  | 1 |  |
| 1methyl4phenylpyridine | D015655 | 1 | 1 | 1 | 1 |
| MPP+ | D015655 | 1 | 1 | 1 | 1 |
| MPP+ | D015655 | 1 | 1 | 1 | 1 |
| Biosensors | D015374 |  |  | 1 |  |
| quinolinones | D015363 |  |  | 1 |  |
| citalopram | D015283 | 2 |  | 1 |  |
| Escitalopram | D015283 | 2 |  | 1 |  |
| dopaminergic agents | D015259 |  |  | 1 |  |
| dopaminergic drugs | D015259 |  |  | 1 |  |
| Sulfotransferase | D015238 |  |  | 1 |  |
| PGE2 | D015232 |  | 1 | 1 | 1 |
| prostaglandin E2 | D015232 |  | 1 | 1 | 1 |
| sodium channel | D015222 |  | 2 | 1 | 0.5 |
| sodium channels | D015222 |  | 2 | 1 | 0.5 |
| Potassium channel | D015221 | 1 |  | 1 |  |
| potassium channels | D015221 | 1 |  | 1 |  |
| calcium channels | D015220 | 1 | 1 | 1 | 1 |
| butyrolactone | D015107 |  |  | 1 |  |
| yohimbine | D015016 |  |  | 1 |  |
| yoga | D015013 |  |  | 1 |  |
| Walker | D014853 |  |  | 1 |  |
| vitamin E | D014810 | 1 |  | 1 |  |
| vitamin B12 | D014805 |  |  | 1 |  |
| Vasoactive intestil peptide | D014660 |  | 1 | 1 | 1 |
| vasoactive intestil polypeptide | D014660 |  | 1 | 1 | 1 |
| vagotomy | D014628 |  |  | 1 |  |
| uridine | D014529 | 1 |  | 1 |  |
| ubiquinone | D014451 |  |  | 1 |  |
| Tunicamycin | D014415 |  |  | 1 |  |
| Wolfram | D014414 |  |  | 1 |  |
| TRP | D014364 |  | 1 | 1 | 1 |
| tryptophan | D014364 |  | 1 | 1 | 1 |
| Triterpene | D014315 |  |  | 1 |  |
| transketolase | D014174 |  |  | 1 |  |
| transferase | D014166 |  |  | 1 |  |
| Biometals | D014131 |  | 1 | 1 | 1 |
| trace element | D014131 |  | 1 | 1 | 1 |
| toluene | D014050 |  |  | 1 |  |
| smokeless tobacco | D014030 |  |  | 1 |  |
| Titate | D014025 |  |  | 1 |  |
| thrombin | D013917 | 1 |  | 1 |  |
| threonine | D013912 |  |  | 1 |  |
| Cardiac Surgery | D013903 |  |  | 1 |  |
| TCDD | D013749 |  |  | 1 |  |
| tea | D013662 |  | 1 | 1 | 1 |
| taurine | D013654 |  | 1 | 1 | 1 |
| tannin | D013634 |  |  | 1 |  |
| tamoxifen | D013629 |  |  | 1 |  |
| sympathetic denervation | D013562 | 2 | 1 | 1 | 1 |
| Ronin | D013427 |  |  | 1 |  |
| steroid | D013256 |  | 1 | 1 | 1 |
| steroids | D013256 |  | 1 | 1 | 1 |
| Sport | D013177 |  |  | 1 |  |
| sphingosine | D013110 |  |  | 1 |  |
| somatostatin | D013004 |  | 2 | 1 | 0.5 |
| solution | D012996 |  | 1 | 1 | 1 |
| solutions | D012996 |  | 1 | 1 | 1 |
| soil | D012987 |  |  | 1 |  |
| Sodium oxybate | D012978 | 1 |  | 1 |  |
| Nighttime | D012890 | 1 |  | 1 |  |
| Nighttime sleep | D012890 | 1 |  | 1 |  |
| sex hormones | D012739 |  |  | 1 |  |
| serum albumin | D012709 |  |  | 1 |  |
| MFI | D012524 |  | 1 | 1 | 1 |
| Sarin | D012524 |  | 1 | 1 | 1 |
| saponins | D012503 |  |  | 1 |  |
| Rubber | D012408 |  |  | 1 |  |
| ribose | D012266 |  |  | 1 |  |
| nicotinic receptor | D011978 | 2 | 1 | 1 | 1 |
| nicotinic receptors | D011978 | 2 | 1 | 1 | 1 |
| muscarinic receptor | D011976 |  | 1 | 1 | 1 |
| Muscarinic receptors | D011976 |  | 1 | 1 | 1 |
| dopamine receptor | D011954 | 4 | 2 | 1 | 0.5 |
| dopamine receptors | D011954 | 4 | 2 | 1 | 0.5 |
| Raf | D011887 |  | 1 | 1 | 1 |
| quinidine | D011802 |  |  | 1 |  |
| Pyridostigmine bromide | D011729 |  |  | 1 |  |
| pyridines | D011725 |  |  | 1 |  |
| pteridine | D011621 |  |  | 1 |  |
| Transglutamise | D011503 |  | 1 | 1 | 1 |
| propan | D011407 |  | 1 | 1 | 1 |
| promoter region | D011401 |  |  | 1 |  |
| promethazine | D011398 |  |  | 1 |  |
| progesterone | D011374 |  |  | 1 |  |
| diol | D011276 |  |  | 1 |  |
| Polysaccharides | D011134 |  |  | 1 |  |
| Polylysine | D011107 |  |  | 1 |  |
| Pluronic P85 | D011060 |  |  | 1 |  |
| Plastic | D010969 |  |  | 1 |  |
| Plasmalogen | D010955 |  |  | 1 |  |
| plant | D010944 |  |  | 1 |  |
| plants | D010944 |  |  | 1 |  |
| Placebo | D010919 | 2 | 3 | 1 | 0.33333333 |
| a 65 | D010892 |  |  | 1 |  |
| PACA | D010842 |  |  | 1 |  |
| organophosphate | D010755 |  |  | 1 |  |
| PRPP | D010754 |  |  | 1 |  |
| Phospholipase D | D010739 |  |  | 1 |  |
| phosphoglycerate kise | D010735 |  |  | 1 |  |
| phosphate | D010710 |  |  | 1 |  |
| phloretin | D010693 |  |  | 1 |  |
| proteases | D010447 |  |  | 1 |  |
| Pentazocine | D010423 |  |  | 1 |  |
| linear accelerator | D010315 |  |  | 1 |  |
| Total Parenteral Nutrition | D010289 |  |  | 1 |  |
| pantothete | D010205 |  |  | 1 |  |
| Pancreatic Polypeptide | D010191 |  |  | 1 |  |
| green tea | D010095 | 3 |  | 1 |  |
| oxotremorine | D010095 | 3 |  | 1 |  |
| Oxide | D010087 | 1 |  | 1 |  |
| oxides | D010087 | 1 |  | 1 |  |
| Oxazepam | D010076 |  |  | 1 |  |
| Nortriptyline | D009661 |  |  | 1 |  |
| nitroprusside | D009599 |  | 1 | 1 | 1 |
| nimodipine | D009553 |  |  | 1 |  |
| neutron | D009502 |  | 1 | 1 | 1 |
| neurosurgery | D009493 | 2 |  | 1 |  |
| Neuropeptide | D009479 | 1 | 1 | 1 | 1 |
| neuropeptides | D009479 | 1 | 1 | 1 | 1 |
| neuropeptide Y | D009478 |  | 1 | 1 | 1 |
| Atomizer | D009330 |  |  | 1 |  |
| D | D009243 | 3 | 3 | 1 | 0.33333333 |
| DH | D009243 | 3 | 3 | 1 | 0.33333333 |
| nicotimide adenine dinucleotide | D009243 | 3 | 3 | 1 | 0.33333333 |
| molsidomine | D008981 |  |  | 1 |  |
| micelle | D008823 | 1 | 1 | 1 | 1 |
| micelles | D008823 | 1 | 1 | 1 | 1 |
| mianserin | D008803 |  |  | 1 |  |
| methylmalonic acid | D008764 |  |  | 1 |  |
| MoCA | D008753 |  |  | 1 |  |
| methylene blue | D008751 |  | 2 | 1 | 0.5 |
| Methylthioninium chloride | D008751 |  | 2 | 1 | 0.5 |
| methionine | D008715 |  |  | 1 |  |
| metalloporphyrin | D008665 | 2 |  | 1 |  |
| metalloporphyrins | D008665 | 2 |  | 1 |  |
| Masks | D008397 |  |  | 1 |  |
| MgSO4 | D008278 |  |  | 1 |  |
| lysophosphatidylcholine | D008244 |  | 1 | 1 | 1 |
| Lysine | D008239 |  |  | 1 |  |
| liposomes | D008081 | 1 | 1 | 1 | 1 |
| Lipoproteins | D008074 |  |  | 1 |  |
| lipid A | D008050 |  |  | 1 |  |
| lead | D007854 | 1 | 2 | 1 | 0.5 |
| lead compounds | D007854 | 1 | 2 | 1 | 0.5 |
| lactulose | D007792 |  | 1 | 1 | 1 |
| ketocozole | D007654 | 1 |  | 1 |  |
| ketamine | D007649 |  |  | 1 |  |
| isoflurane | D007530 |  |  | 1 |  |
| ion channel | D007473 | 2 |  | 1 |  |
| ion channels | D007473 | 2 |  | 1 |  |
| intron | D007438 |  |  | 1 |  |
| IL1 | D007375 | 1 |  | 1 |  |
| inositol | D007294 | 1 |  | 1 |  |
| Intraperitoneal Injections | D007274 |  |  | 1 |  |
| Immunization | D007114 |  | 1 | 1 | 1 |
| immunoglobulin G | D007074 |  |  | 1 |  |
| IgA | D007070 |  |  | 1 |  |
| Hydrocortisone | D006854 |  |  | 1 |  |
| hydrazone | D006835 |  |  | 1 |  |
| Homovanillic acid | D006719 |  |  | 1 |  |
| Histones | D006657 |  |  | 1 |  |
| histamine | D006632 | 1 | 2 | 1 | 0.5 |
| heptachlor | D006533 | 1 |  | 1 |  |
| tetrahydro | D006533 | 1 |  | 1 |  |
| heparan sulfate | D006497 |  |  | 1 |  |
| haloperidol | D006220 | 2 | 4 | 1 | 0.25 |
| guanosine | D006151 |  | 1 | 1 | 1 |
| glucosylceramide | D005963 |  |  | 1 |  |
| Glucose6phosphatase | D005952 |  |  | 1 |  |
| glucose | D005947 | 1 |  | 1 |  |
| glucocorticoid | D005938 | 1 | 2 | 1 | 0.5 |
| glucocorticoids | D005938 | 1 | 2 | 1 | 0.5 |
| GVS | D005840 |  |  | 1 |  |
| ganglioside | D005732 |  | 1 | 1 | 1 |
| gangliosides | D005732 |  | 1 | 1 | 1 |
| gammaglutamyltransferase | D005723 |  |  | 1 |  |
| fostering | D005581 |  |  | 1 |  |
| football | D005538 |  | 2 | 1 | 0.5 |
| fludrocortisone | D005438 |  |  | 1 |  |
| oxo | D004987 |  |  | 1 |  |
| estrogen | D004967 | 10 |  | 1 |  |
| estrogens | D004967 | 10 |  | 1 |  |
| Erythropoietin | D004921 |  | 1 | 1 | 1 |
| electroconvulsive therapy | D004565 | 1 | 2 | 1 | 0.5 |
| ECG | D004562 |  |  | 1 |  |
| transcutaneous electrical nerve stimulation | D004561 |  |  | 1 |  |
| dynorphin | D004399 |  | 1 | 1 | 1 |
| Drug therapy | D004358 | 1 | 2 | 1 | 0.5 |
| pharmacotherapies | D004358 | 1 | 2 | 1 | 0.5 |
| pharmacotherapy | D004358 | 1 | 2 | 1 | 0.5 |
| doxycycline | D004318 |  |  | 1 |  |
| D binding protein | D004268 |  |  | 1 |  |
| disaccharides | D004187 |  |  | 1 |  |
| diquat | D004178 |  |  | 1 |  |
| dinitrophenol | D004140 |  |  | 1 |  |
| Des | D004054 |  |  | 1 |  |
| DEN | D004052 |  |  | 1 |  |
| dietary fat | D004041 |  |  | 1 |  |
| diazepam | D003975 |  |  | 1 |  |
| dextromethorphan | D003915 |  | 1 | 1 | 1 |
| dextran | D003911 |  |  | 1 |  |
| Dexamethasone | D003907 |  |  | 1 |  |
| desipramine | D003891 | 1 |  | 1 |  |
| Citicoline | D003566 |  |  | 1 |  |
| cystathionine | D003540 |  |  | 1 |  |
| Complement | D003165 |  |  | 1 |  |
| Cocaine | D003042 | 1 |  | 1 |  |
| clozapine | D003024 | 1 | 1 | 1 | 1 |
| clathrin | D002966 |  |  | 1 |  |
| chromone | D002867 |  | 2 | 1 | 0.5 |
| Chromones | D002867 |  | 2 | 1 | 0.5 |
| Chromatin | D002843 |  |  | 1 |  |
| chondroitin | D002807 |  |  | 1 |  |
| Cholinesterase | D002802 |  | 1 | 1 | 1 |
| cholinesterase inhibitor | D002800 | 1 | 1 | 1 | 1 |
| cholinesterase inhibitors | D002800 | 1 | 1 | 1 | 1 |
| Vitamin D3 | D002762 |  |  | 1 |  |
| Chloroquine | D002738 |  |  | 1 |  |
| Chloride | D002712 |  |  | 1 |  |
| Chlamydomos | D002696 |  |  | 1 |  |
| Cerium | D002563 |  |  | 1 |  |
| grain | D002523 |  |  | 1 |  |
| grains | D002523 |  |  | 1 |  |
| Ceramide | D002518 |  | 1 | 1 | 1 |
| cathepsin D | D002402 | 1 |  | 1 |  |
| Carnosine | D002336 | 1 |  | 1 |  |
| carnitine | D002331 |  | 1 | 1 | 1 |
| cardiolipin | D002308 |  |  | 1 |  |
| Carbon Dioxide | D002245 | 1 |  | 1 |  |
| CO2 | D002245 | 1 |  | 1 |  |
| Methyl Group | D002244 |  |  | 1 |  |
| canbinoid | D002186 | 2 | 2 | 1 | 0.5 |
| canbinoids | D002186 | 2 | 2 | 1 | 0.5 |
| Canbidiol | D002185 |  |  | 1 |  |
| CBD | D002185 |  |  | 1 |  |
| camptothecin | D002166 |  |  | 1 |  |
| calpain | D002154 | 3 |  | 1 |  |
| calcitriol | D002117 |  |  | 1 |  |
| Biperiden | D001712 |  |  | 1 |  |
| bibenzyl | D001632 |  |  | 1 |  |
| berberine | D001599 |  |  | 1 |  |
| Benzoxazoles | D001583 |  |  | 1 |  |
| benzoate | D001565 |  |  | 1 |  |
| atrazine | D001280 |  |  | 1 |  |
| aspartame | D001218 |  |  | 1 |  |
| Asparagine | D001216 |  |  | 1 |  |
| arthroplasty | D001178 |  | 1 | 1 | 1 |
| Appendectomy | D001062 |  |  | 1 |  |
| antidepressants | D000928 | 1 | 2 | 1 | 0.5 |
| antiepileptic drugs | D000927 | 1 |  | 1 |  |
| anthocyanins | D000872 |  |  | 1 |  |
| animals | D000818 | 2 | 1 | 1 | 1 |
| general anesthesia | D000768 |  | 1 | 1 | 1 |
| algesic drugs | D000700 | 1 |  | 1 |  |
| algesics | D000700 | 1 |  | 1 |  |
| amphetamine | D000661 | 1 | 1 | 1 | 1 |
| amodiaquine | D000655 |  |  | 1 |  |
| ND4 | D000644 |  |  | 1 |  |
| Aminolevulite | D000622 |  |  | 1 |  |
| amino acid | D000596 | 2 | 1 | 1 | 1 |
| amino acids | D000596 | 2 | 1 | 1 | 1 |
| ambroxol | D000551 |  |  | 1 |  |
| aldehyde | D000447 |  | 1 | 1 | 1 |
| air | D000388 |  | 1 | 1 | 1 |
| sodiumpotassium pump | D000254 |  |  | 1 |  |
| adenylate | D000249 | 1 |  | 1 |  |
| ADP | D000244 |  |  | 1 |  |
| daily living activities | D000203 |  |  | 1 |  |
| actin | D000199 | 1 |  | 1 |  |
| acetylcholinesterase | D000110 | 1 | 1 | 1 | 1 |
| ALCAR | D000108 |  |  | 1 |  |
| Salvianolic acid | C568740 | 1 |  | 1 |  |
| idalopirdine | C568612 |  |  | 1 |  |
| SU4312 | C549541 |  |  | 1 |  |
| 4RCembranoid | C544693 |  |  | 1 |  |
| abobotulinumtoxi | C542869 |  |  | 1 |  |
| Dalfampridine | C542865 |  |  | 1 |  |
| a cysteine | C538813 | 1 | 3 | 1 | 0.33333333 |
| Cys | C538813 | 1 | 3 | 1 | 0.33333333 |
| cysteite | C538813 | 1 | 3 | 1 | 0.33333333 |
| prM | C533957 |  |  | 1 |  |
| MEK Inhibitor | C533206 |  |  | 1 |  |
| Azilsartan | C521273 |  |  | 1 |  |
| carboxamide | C521013 |  |  | 1 |  |
| Docosahexaenoyl Dopamine | C520236 |  |  | 1 |  |
| Resolvin D1 | C518399 |  |  | 1 |  |
| Aspartate Glutamate | C514131 |  | 2 | 1 | 0.5 |
| GLT | C514131 |  | 2 | 1 | 0.5 |
| PMN | C510909 |  |  | 1 |  |
| 5Hydroxytriptolide | C509074 |  |  | 1 |  |
| dieckol | C503840 |  |  | 1 |  |
| varenicline | C500838 |  |  | 1 |  |
| necrostatin | C500233 |  |  | 1 |  |
| Sitagliptin | C496398 |  |  | 1 |  |
| Noleoyldopamine | C488796 |  |  | 1 |  |
| Phosphatidylethanolamine | C483858 |  |  | 1 |  |
| FAUC 329 | C477900 |  |  | 1 |  |
| erastin | C477224 |  |  | 1 |  |
| Dihydromyricetin | C472036 |  |  | 1 |  |
| Succinobucol | C471047 |  |  | 1 |  |
| Purmorphamine | C470893 |  |  | 1 |  |
| Isobavachalcone | C468754 |  |  | 1 |  |
| Dabigatran Etexilate | C453962 |  |  | 1 |  |
| FDDNP | C444520 |  |  | 1 |  |
| SCL | C441521 |  |  | 1 |  |
| AM1241 | C439263 |  |  | 1 |  |
| histone deacetylase 3 | C438596 |  |  | 1 |  |
| Humanin | C427211 |  |  | 1 |  |
| AL108 | C425904 | 2 |  | 1 |  |
| AL208 | C425904 | 2 |  | 1 |  |
| davunetide | C425904 | 2 |  | 1 |  |
| PVSIPQ | C425904 | 2 |  | 1 |  |
| rosuvastatin | C422923 |  |  | 1 |  |
| PDX | C418863 |  |  | 1 |  |
| Imidazo | C416932 |  |  | 1 |  |
| BGP15 | C405586 |  |  | 1 |  |
| Temsirolimus | C401859 |  |  | 1 |  |
| SYN | C3886793 |  | 1 | 1 | 1 |
| SYN115 | C3886793 |  | 1 | 1 | 1 |
| Anchor | C3854239 |  |  | 1 |  |
| TrkB | C3853697 |  |  | 1 |  |
| SRC | C3853644 |  | 1 | 1 | 1 |
| tACS | C3852966 |  |  | 1 |  |
| dopaminergic compensatory mechanisms | C3825331 |  | 1 | 1 | 1 |
| dopaminergic mechanisms | C3825331 |  | 1 | 1 | 1 |
| BAG3 | C3816451 |  |  | 1 |  |
| Mfn2 | C3816215 |  | 1 | 1 | 1 |
| Mitofusin 2 | C3816215 |  | 1 | 1 | 1 |
| delta | C3815527 |  | 2 | 1 | 0.5 |
| oxygen therapy | C3665674 |  |  | 1 |  |
| DiHuangYinZi | C3658712 |  |  | 1 |  |
| apelin | C3542402 |  |  | 1 |  |
| AlphaSynucleinInteracting Protein | C3541909 | 3 | 2 | 1 | 0.5 |
| synphilin 1 | C3541909 | 3 | 2 | 1 | 0.5 |
| Synphilin1 | C3541909 | 3 | 2 | 1 | 0.5 |
| PARK7 | C3538910 | 1 |  | 1 |  |
| protein DJ1 | C3538910 | 1 |  | 1 |  |
| NMDAR2B | C3537346 |  |  | 1 |  |
| ARE | C3494205 |  | 2 | 1 | 0.5 |
| Bactris guineensis | C3418646 |  |  | 1 |  |
| Boerhaavia diffusa | C3099352 |  |  | 1 |  |
| targeted antioxidant therapy | C2985566 |  |  | 1 |  |
| DaTscans | C2980484 |  |  | 1 |  |
| Crest | C2948667 |  |  | 1 |  |
| GABAa receptor antagonists | C2936801 |  |  | 1 |  |
| Purinergic Antagonism | C2936568 |  |  | 1 |  |
| duration | C2926735 |  | 2 | 1 | 0.5 |
| GLP1/GIP dual receptor agonist | C2917359 |  |  | 1 |  |
| contamints | C2827365 | 1 |  | 1 |  |
| programme | C2728259 |  | 1 | 1 | 1 |
| manometric | C2720530 |  |  | 1 |  |
| Sirt3 | C2720169 |  |  | 1 |  |
| highfrequency oscillations | C2718088 |  |  | 1 |  |
| depth electrode EEG | C2711277 |  |  | 1 |  |
| MAPT | C2700455 | 2 | 1 | 1 | 1 |
| microtubuleassociated protein tau | C2700455 | 2 | 1 | 1 | 1 |
| Cassia tora | C2614189 |  |  | 1 |  |
| voice treatment | C2459996 |  | 2 | 1 | 0.5 |
| MitoNEET | C2003363 |  |  | 1 |  |
| methyltransferase inhibitors | C1997663 |  |  | 1 |  |
| Epimedium koreanum kai extract | C1950830 |  |  | 1 |  |
| Intervention | C1948041 |  |  | 1 |  |
| Whey protein isolate | C1883555 |  |  | 1 |  |
| PARP inhibitors | C1882413 |  |  | 1 |  |
| bloodbrain barrier disruption | C1831732 |  |  | 1 |  |
| Conveyer | C1740548 |  |  | 1 |  |
| New Drug | C1718097 |  |  | 1 |  |
| New drugs | C1718097 |  |  | 1 |  |
| barriers | C1706912 |  | 1 | 1 | 1 |
| IL1alpha | C1702299 |  |  | 1 |  |
| Bacopa monniera | C1653429 |  | 2 | 1 | 0.5 |
| Bacopa monnieri | C1653429 |  | 2 | 1 | 0.5 |
| surgical specimens | C1647891 |  |  | 1 |  |
| LXR | C1612060 |  |  | 1 |  |
| syptopodin | C1611526 |  |  | 1 |  |
| CREB/VMAT2 | C1566322 |  |  | 1 |  |
| TRPC Channels | C1563722 |  |  | 1 |  |
| Transforming growth factorbeta1 | C1515406 |  |  | 1 |  |
| metabolic biomarkers | C1513159 |  | 1 | 1 | 1 |
| Metabolic markers | C1513159 |  | 1 | 1 | 1 |
| matrix metalloproteise8 inhibitor | C1513016 |  |  | 1 |  |
| Trx | C1504637 |  |  | 1 |  |
| Veronica jacquinii | C1483275 |  |  | 1 |  |
| Passiflora cincinta | C1459698 |  |  | 1 |  |
| 1433/phosphoTau | C1458135 |  |  | 1 |  |
| MAP Kise SWIP13 | C1456416 |  |  | 1 |  |
| human Tau protein | C1454489 |  |  | 1 |  |
| Thioredoxininteracting protein | C1450344 |  |  | 1 |  |
| homeodomaininteracting protein kise 2 | C1447702 |  |  | 1 |  |
| Rip2 | C1447644 |  |  | 1 |  |
| CIB1 | C1447592 |  |  | 1 |  |
| Protein Phosphatase 2A Catalytic Subunit Activity in Cell Free Assays. alpha | C1335539 |  |  | 1 |  |
| GPR17 | C1333731 |  |  | 1 |  |
| BAG5 | C1332393 |  | 1 | 1 | 1 |
| Apoptosis Inhibitors | C1332320 |  |  | 1 |  |
| Small Molecular | C1328819 |  |  | 1 |  |
| antipsychotic therapy | C1319421 |  |  | 1 |  |
| fatty acid binding protein 3 | C1312696 |  |  | 1 |  |
| Dicer | C1309483 |  |  | 1 |  |
| stroke prevention | C1277289 |  |  | 1 |  |
| Atypical antipsychotic | C1276996 | 1 | 1 | 1 | 1 |
| atypical antipsychotic drugs | C1276996 | 1 | 1 | 1 | 1 |
| atypical antipsychotics | C1276996 | 1 | 1 | 1 | 1 |
| Glitazone | C1257987 |  |  | 1 |  |
| MPEP | C121465 | 1 |  | 1 |  |
| painfree | C121034 |  |  | 1 |  |
| Tectorigenin | C120039 |  |  | 1 |  |
| Spatholobi | C1189810 |  |  | 1 |  |
| pyrazolopyridine | C118531 |  |  | 1 |  |
| protein complexes | C1180347 |  |  | 1 |  |
| Cu2 | C1177210 | 1 |  | 1 |  |
| committed | C1171947 |  |  | 1 |  |
| isochronous | C1170358 |  |  | 1 |  |
| Iminoquinone | C116897 |  |  | 1 |  |
| Human Proteins | C1142652 |  |  | 1 |  |
| Sirts | C1136177 |  |  | 1 |  |
| Olea europaea | C1122969 |  |  | 1 |  |
| dutasteride | C108373 |  |  | 1 |  |
| Angelica dahurica | C1083049 |  |  | 1 |  |
| Glabridin | C107601 |  |  | 1 |  |
| Bougainvillea spectabilis | C1070355 |  |  | 1 |  |
| ALTHEA | C1070218 |  |  | 1 |  |
| Aldehyde dehydrogese 1 | C105537 |  |  | 1 |  |
| Selol | C105370 |  |  | 1 |  |
| 5HT7 Receptor | C104425 |  |  | 1 |  |
| mGluR1 | C104077 |  |  | 1 |  |
| Blue Lotus | C1014691 |  |  | 1 |  |
| Nymphea caerulea | C1014691 |  |  | 1 |  |
| decursin | C101278 |  |  | 1 |  |
| Bupleurum falcatum | C1012569 |  |  | 1 |  |
| Aloe arborescens | C1011943 |  |  | 1 |  |
| Paeonia suffruticosa | C1011804 |  |  | 1 |  |
| selank | C100827 |  |  | 1 |  |
| Sorbus alnifolia | C1006302 |  |  | 1 |  |
| neuroserpin | C100531 |  |  | 1 |  |
| Gracilaria cornea | C1003664 |  |  | 1 |  |
| SHR | C100169 |  |  | 1 |  |
| CB1 | C100099 |  | 1 | 1 | 1 |
| Vanillin | C100058 |  |  | 1 |  |
| P2Y6 receptor | C099845 |  |  | 1 |  |
| Bougainvillea glabra | C0996705 |  |  | 1 |  |
| Humulus japonicus | C0996683 |  |  | 1 |  |
| Alaria esculenta | C0996325 |  |  | 1 |  |
| elders | C0994408 |  | 1 | 1 | 1 |
| sal gel | C0991526 |  |  | 1 |  |
| syptojanin | C097702 |  |  | 1 |  |
| Board | C0972401 |  |  | 1 |  |
| Piloty's Acid | C097220 |  |  | 1 |  |
| polyglutamine | C097188 | 4 | 7 | 1 | 0.14285714 |
| polyQ | C097188 | 4 | 7 | 1 | 0.14285714 |
| p10 | C0966870 |  | 1 | 1 | 1 |
| p11 | C0966870 |  | 1 | 1 | 1 |
| AtractylenolideI | C0965735 |  |  | 1 |  |
| HIF1alpha | C0965644 | 1 |  | 1 |  |
| rhTFAM | C0965025 |  |  | 1 |  |
| of nuclear receptorrelated 1 protein | C0961411 |  |  | 1 |  |
| Rhizoma | C0949790 |  |  | 1 |  |
| Manipulated | C0947647 |  | 3 | 1 | 0.33333333 |
| manipulation | C0947647 |  | 3 | 1 | 0.33333333 |
| Montelukast | C093875 |  |  | 1 |  |
| controlled conditions | C0920467 |  | 2 | 1 | 0.5 |
| disease locomotion control | C0920467 |  | 2 | 1 | 0.5 |
| PD controls | C0920467 |  | 2 | 1 | 0.5 |
| speech rehabilitation | C0919925 |  |  | 1 |  |
| alpha4beta2 ChR | C0911771 |  | 2 | 1 | 0.5 |
| alpha4beta2 neurol nicotinic receptors | C0911771 |  | 2 | 1 | 0.5 |
| alpha4beta2 Nicotinic Receptors | C0911771 |  | 2 | 1 | 0.5 |
| Protosappanin A | C090723 |  | 1 | 1 | 1 |
| Thiazolidinedione | C089946 |  |  | 1 |  |
| NOMI | C089750 |  |  | 1 |  |
| human urine | C0885847 |  |  | 1 |  |
| FP CIT | C087552 |  |  | 1 |  |
| wogonin | C085514 |  |  | 1 |  |
| Hydroxysafflor Yellow A | C085278 |  |  | 1 |  |
| Radiofrequency ablation | C0850292 |  |  | 1 |  |
| Squamosamide | C084439 | 1 |  | 1 |  |
| Rikkunshito | C084136 |  |  | 1 |  |
| Isoflavonoids | C0815051 |  |  | 1 |  |
| noncompetitive muscarinic antagonists | C0815027 |  |  | 1 |  |
| toxin 6 | C081494 |  |  | 1 |  |
| Checkpoint Kise 1 | C081350 |  |  | 1 |  |
| sargramostim | C081222 |  |  | 1 |  |
| pacemakers | C0810633 |  |  | 1 |  |
| brain stimulator | C0810489 |  |  | 1 |  |
| brain stimulators | C0810489 |  |  | 1 |  |
| Mortalin | C080352 | 1 |  | 1 |  |
| fruit extracts | C0772257 |  |  | 1 |  |
| aromatic | C0772162 | 1 | 1 | 1 | 1 |
| Calnuc | C076066 |  |  | 1 |  |
| Perilipin2 | C0760488 |  |  | 1 |  |
| gammaSynucleins | C0753208 |  |  | 1 |  |
| EKO | C075270 |  |  | 1 |  |
| Granulins | C074957 |  |  | 1 |  |
| Kamikihito | C073747 |  |  | 1 |  |
| Bilobalide | C073710 | 1 |  | 1 |  |
| Carvacrol | C073316 |  |  | 1 |  |
| stopping | C0723457 |  | 1 | 1 | 1 |
| Arctigenin | C071942 |  |  | 1 |  |
| remifentanil | C071741 |  |  | 1 |  |
| Licochalcone A | C070840 |  |  | 1 |  |
| legumain | C069586 |  |  | 1 |  |
| CA1 | C069373 | 1 |  | 1 |  |
| NTS | C068445 |  | 1 | 1 | 1 |
| novel neuroprotective therapy | C0683465 | 1 | 1 | 1 | 1 |
| novel therapies | C0683465 | 1 | 1 | 1 | 1 |
| Novel Treatments | C0683465 | 1 | 1 | 1 | 1 |
| Roc | C0683187 | 3 |  | 1 |  |
| carboxylic acid derivatives | C0682936 |  |  | 1 |  |
| Olympic | C0681662 |  |  | 1 |  |
| nutrient | C0678695 |  |  | 1 |  |
| SMPD1 | C0676458 |  |  | 1 |  |
| polypyrrole | C067635 | 1 | 1 | 1 | 1 |
| pyrrolo | C067635 | 1 | 1 | 1 | 1 |
| Prohibitin | C067438 |  |  | 1 |  |
| TaClo | C067372 |  |  | 1 |  |
| iNOS | C0669372 |  | 1 | 1 | 1 |
| neurol nitric oxide synthases | C0669368 |  |  | 1 |  |
| pleiotrophin | C066845 |  | 1 | 1 | 1 |
| AF6 | C0667316 |  |  | 1 |  |
| tolcapone | C066340 | 4 | 1 | 1 | 1 |
| HuanglianJieDuTang | C061676 |  |  | 1 |  |
| 26S proteasome | C061553 |  |  | 1 |  |
| diphenyl diselenide | C061132 |  |  | 1 |  |
| pioglitazone | C060836 | 2 | 1 | 1 | 1 |
| eckol | C060311 |  |  | 1 |  |
| ERK | C0600388 | 3 | 4 | 1 | 0.25 |
| ERK1 | C0600388 | 3 | 4 | 1 | 0.25 |
| extracellular siglregulated kise | C0600388 | 3 | 4 | 1 | 0.25 |
| stretch | C0600080 |  | 1 | 1 | 1 |
| Stretch | C0600080 |  | 1 | 1 | 1 |
| adapter protein | C0599697 |  | 1 | 1 | 1 |
| JAK | C0597721 |  |  | 1 |  |
| Pyrethroid | C0597329 |  |  | 1 |  |
| Unkeito | C058415 |  |  | 1 |  |
| infusion | C0574032 | 1 |  | 1 |  |
| infusions | C0574032 | 1 |  | 1 |  |
| molecule | C0567416 | 1 | 3 | 1 | 0.33333333 |
| molecules | C0567416 | 1 | 3 | 1 | 0.33333333 |
| icariin | C056599 |  |  | 1 |  |
| exemestane | C056516 |  |  | 1 |  |
| Omega3 Fatty Acids Supplementation | C0561929 | 1 |  | 1 |  |
| omega3 fattyacid supplementation | C0561929 | 1 |  | 1 |  |
| apocynin | C056165 |  | 1 | 1 | 1 |
| Dimethyl Fumarate | C056020 |  |  | 1 |  |
| Amino Acid Supplementation | C0556082 |  |  | 1 |  |
| Calcipotriol | C055085 |  |  | 1 |  |
| ablation | C0547070 | 1 | 4 | 1 | 0.25 |
| human plasma | C0544357 | 1 |  | 1 |  |
| Sesamin | C054125 | 1 | 1 | 1 | 1 |
| Syptotagmin11 | C0539651 |  |  | 1 |  |
| HMOX1 | C0538674 |  | 1 | 1 | 1 |
| HO1 | C0538674 |  | 1 | 1 | 1 |
| caspase3/7 | C0537969 |  |  | 1 |  |
| 5HT1F Receptor | C0534690 |  |  | 1 |  |
| FAAH | C0531004 |  |  | 1 |  |
| metabotropic glutamate receptor subtype 5 | C0530778 | 2 | 1 | 1 | 1 |
| metabotropic glutamate receptor type 5 | C0530778 | 2 | 1 | 1 | 1 |
| mGluR5 | C0530778 | 2 | 1 | 1 | 1 |
| BMP2 | C0527443 |  |  | 1 |  |
| neurostimulation | C0521307 |  | 2 | 1 | 0.5 |
| CSF Replacement | C0521302 |  |  | 1 |  |
| CalciumDependent Protein Kise | C052123 |  |  | 1 |  |
| a 10 | C052091 |  | 2 | 1 | 0.5 |
| thioperamide | C052075 |  |  | 1 |  |
| Eudragit RS | C050528 |  |  | 1 |  |
| 1MeTIQ | C050386 |  |  | 1 |  |
| Demethoxycurcumin | C050229 |  |  | 1 |  |
| Fasudil | C049347 |  |  | 1 |  |
| TOF | C049232 | 1 |  | 1 |  |
| modafinil | C048833 | 3 |  | 1 |  |
| semax | C048487 |  |  | 1 |  |
| cabergoline | C047047 | 7 | 7 | 1 | 0.14285714 |
| Trimethyltin | C046488 |  |  | 1 |  |
| Angiogenin | C046366 |  | 1 | 1 | 1 |
| Discoidin domain receptor | C046287 |  |  | 1 |  |
| Ginkgolide B | C045856 |  |  | 1 |  |
| epigallocatechin gallate | C045651 |  |  | 1 |  |
| movement therapy | C0454279 |  |  | 1 |  |
| Gastrodin | C045345 |  | 1 | 1 | 1 |
| Dripping | C0452837 |  |  | 1 |  |
| pinhole | C0450042 |  |  | 1 |  |
| Continuous intranigral infusion | C0444889 |  |  | 1 |  |
| Wiring | C0441650 |  |  | 1 |  |
| debris | C0440266 |  |  | 1 |  |
| mixture | C0439962 | 1 | 2 | 1 | 0.5 |
| mixtures | C0439962 | 1 | 2 | 1 | 0.5 |
| substance | C0439861 | 1 | 2 | 1 | 0.5 |
| substances | C0439861 | 1 | 2 | 1 | 0.5 |
| transcranial static magnetic field stimulation | C0436548 |  |  | 1 |  |
| chrysin | C043561 |  |  | 1 |  |
| PLGA | C043435 |  |  | 1 |  |
| pressure body weightsupport | C0419008 |  |  | 1 |  |
| nitazoxanide | C041747 |  |  | 1 |  |
| GIP receptor | C041086 |  |  | 1 |  |
| Lumbar Spine Surgery | C0408578 |  |  | 1 |  |
| ToL | C040550 |  |  | 1 |  |
| caffeic acid | C040048 |  |  | 1 |  |
| ventricular surgery | C0397255 |  |  | 1 |  |
| Syptojanin 1 | C0385927 |  |  | 1 |  |
| ibudilast | C038366 |  |  | 1 |  |
| Nramp1 | C0380924 |  |  | 1 |  |
| Decompression Surgery | C0376530 |  |  | 1 |  |
| Isothiocyate | C037152 |  |  | 1 |  |
| samples | C0370003 |  |  | 1 |  |
| acylcarnitines | C0368608 |  |  | 1 |  |
| salsolinol | C036617 | 2 | 3 | 1 | 0.33333333 |
| Organophosphate pesticides | C0360429 |  |  | 1 |  |
| dauricine | C035934 |  |  | 1 |  |
| Glucosylsphingosine | C035742 |  |  | 1 |  |
| mirtazapine | C035133 |  |  | 1 |  |
| mitochondrial calcium uniporter | C034703 |  |  | 1 |  |
| matrine | C034244 |  |  | 1 |  |
| Isocitrate | C034219 |  |  | 1 |  |
| puerarin | C033607 |  | 1 | 1 | 1 |
| elder | C0331055 |  |  | 1 |  |
| Rhus typhi | C0330956 |  |  | 1 |  |
| dihydrotetrabezine | C032811 | 4 | 1 | 1 | 1 |
| Black and White | C031927 |  |  | 1 |  |
| Phenothiazine | C031637 |  |  | 1 |  |
| pyrazole | C031280 |  |  | 1 |  |
| carboxamides | C031066 |  |  | 1 |  |
| surviving | C0310255 |  |  | 1 |  |
| benzimidazole | C031000 |  |  | 1 |  |
| pyrimidin | C030986 | 3 |  | 1 |  |
| pyrimidine | C030986 | 3 |  | 1 |  |
| ceria | C030583 |  |  | 1 |  |
| Psychostimulant | C0304403 |  |  | 1 |  |
| Indol | C030374 |  | 1 | 1 | 1 |
| Indole | C030374 |  | 1 | 1 | 1 |
| MLi | C030290 |  |  | 1 |  |
| Peripheral Myelin | C0301710 |  |  | 1 |  |
| Benzopyrone | C030123 |  |  | 1 |  |
| imidazole | C029899 |  |  | 1 |  |
| tartrate | C029768 |  |  | 1 |  |
| aquaporin4 | C0292777 |  |  | 1 |  |
| AEG | C028791 |  |  | 1 |  |
| POPC | C028694 |  |  | 1 |  |
| Mat | C028526 |  |  | 1 |  |
| mPEG | C028210 |  |  | 1 |  |
| Androgen deprivation therapy | C0279492 |  |  | 1 |  |
| potentiation | C0279023 |  |  | 1 |  |
| hydroxyephedrine | C026055 |  | 2 | 1 | 0.5 |
| DAS | C025953 |  |  | 1 |  |
| Hederagenin | C025763 |  |  | 1 |  |
| Trichloromethyl | C025280 |  |  | 1 |  |
| MEF2D | C0250373 |  |  | 1 |  |
| A factor | C025035 |  |  | 1 |  |
| Caryophyllene | C024714 |  |  | 1 |  |
| anticholinergic medications | C0242896 |  |  | 1 |  |
| Caulis | C0242767 |  |  | 1 |  |
| leaf | C0242724 |  |  | 1 |  |
| dopamine receptor blockers | C0242702 |  |  | 1 |  |
| daurisoline | C023968 |  |  | 1 |  |
| Ferrireductase | C023903 |  |  | 1 |  |
| Acacetin | C023717 |  | 2 | 1 | 0.5 |
| indolinone | C022960 |  |  | 1 |  |
| fiber | C0225326 | 1 |  | 1 |  |
| Prolyl oligopeptidase | C022432 | 1 | 1 | 1 | 1 |
| QM protein | C0218544 |  |  | 1 |  |
| Hyperoside | C021304 |  |  | 1 |  |
| mangostin | C021053 |  |  | 1 |  |
| C/EBPbeta | C0209548 |  | 1 | 1 | 1 |
| CCAAT/Enhancer binding protein beta | C0209548 |  | 1 | 1 | 1 |
| GHK | C020905 |  |  | 1 |  |
| AAV9GDNF | C0207072 |  |  | 1 |  |
| nuclear receptor | C0206588 |  | 2 | 1 | 0.5 |
| Nurr1 | C0206588 |  | 2 | 1 | 0.5 |
| Elective | C0206058 |  |  | 1 |  |
| Structural ConnectomeBased Alysis | C0204514 |  |  | 1 |  |
| Pinealectomy | C0193735 |  |  | 1 |  |
| Mobilization | C0185112 |  |  | 1 |  |
| manipulations | C0185111 | 1 | 1 | 1 | 1 |
| Rollator | C0184339 |  |  | 1 |  |
| tweezer | C0184187 |  | 1 | 1 | 1 |
| support | C0183683 |  | 1 | 1 | 1 |
| Spreader | C0183479 |  |  | 1 |  |
| Probe | C0182400 |  | 1 | 1 | 1 |
| probes | C0182400 |  | 1 | 1 | 1 |
| Alterriol Monomethyl Ether | C018206 |  |  | 1 |  |
| manifolds | C0181722 |  |  | 1 |  |
| Lift | C0181620 |  |  | 1 |  |
| Cups | C0180231 |  |  | 1 |  |
| Cover | C0180153 |  |  | 1 |  |
| controller | C0180112 |  |  | 1 |  |
| PAMs | C017915 |  |  | 1 |  |
| alyser | C0179038 |  |  | 1 |  |
| Adapter | C0178959 |  |  | 1 |  |
| hypo | C017717 | 1 |  | 1 |  |
| stimulators | C0175727 |  |  | 1 |  |
| cypermethrin | C017160 |  | 1 | 1 | 1 |
| sepiapterin | C016727 | 1 |  | 1 |  |
| MMP9 | C0165519 |  |  | 1 |  |
| Synuclein's | C0165073 |  |  | 1 |  |
| paeoniflorin | C015423 |  | 1 | 1 | 1 |
| postural exercises | C0150716 |  |  | 1 |  |
| arsenite | C015001 |  |  | 1 |  |
| plumbagin | C014758 |  |  | 1 |  |
| DAC | C014347 |  |  | 1 |  |
| Paeonol | C013638 |  | 1 | 1 | 1 |
| Leonurine | C013587 |  |  | 1 |  |
| DEG | C013484 |  |  | 1 |  |
| neurotrophin receptor | C0132173 |  |  | 1 |  |
| Alpha Lipoamide | C013091 | 1 |  | 1 |  |
| lipoamide | C013091 | 1 |  | 1 |  |
| MAG | C0129439 |  |  | 1 |  |
| benzotriazole | C012771 |  |  | 1 |  |
| Adenylosuccite | C012168 |  |  | 1 |  |
| Amentoflavone | C011164 |  |  | 1 |  |
| glyphosate | C010974 |  | 2 | 1 | 0.5 |
| daf | C0108793 |  |  | 1 |  |
| biphenyl | C010574 |  | 1 | 1 | 1 |
| diphenyl | C010574 |  | 1 | 1 | 1 |
| Angiotensin 17 | C0103306 |  |  | 1 |  |
| titanium dioxide | C009495 |  |  | 1 |  |
| Salidroside | C009172 |  | 1 | 1 | 1 |
| protocatechuic acid | C009091 |  |  | 1 |  |
| 143 | C0090388 | 1 |  | 1 |  |
| amyloid precursor protein | C0085151 | 2 | 2 | 1 | 0.5 |
| APP | C0085151 | 2 | 2 | 1 | 0.5 |
| betaAPP | C0085151 | 2 | 2 | 1 | 0.5 |
| PDQ | C008418 | 4 | 3 | 1 | 0.33333333 |
| Nur77 | C0083740 |  |  | 1 |  |
| MANF | C0083735 |  |  | 1 |  |
| Ginsenosides Rd | C0082699 |  |  | 1 |  |
| 25Hydroxycholesterol | C007997 |  |  | 1 |  |
| Complement receptor 3 | C0079785 |  |  | 1 |  |
| Colony Stimulating Factor1 | C0079784 |  |  | 1 |  |
| CSFPR 2 | C0079460 |  |  | 1 |  |
| E3 Ubiquitin Ligases | C0077678 |  |  | 1 |  |
| thrombinPAR1 | C0076552 |  |  | 1 |  |
| ethyl ester | C007650 |  |  | 1 |  |
| Tauroursodeoxycholic bile acid | C0075857 |  |  | 1 |  |
| DOPAL | C007430 |  |  | 1 |  |
| CNQ | C007332 |  |  | 1 |  |
| PoreForming Proteins | C0071728 |  |  | 1 |  |
| Cerebrolysin | C006952 |  |  | 1 |  |
| Growth Associated Protein43 | C0068563 |  |  | 1 |  |
| Brilliant Blue | C006796 |  |  | 1 |  |
| Bisphenol A | C006780 |  |  | 1 |  |
| MTHFR | C0066357 |  |  | 1 |  |
| Liuwei dihuang | C0065103 |  |  | 1 |  |
| U18666A | C006261 |  |  | 1 |  |
| glu | C0061472 |  |  | 1 |  |
| glutamate transporters | C0061467 | 1 |  | 1 |  |
| 2,4DAB | C005959 |  |  | 1 |  |
| palmitoylethanolamide | C005958 |  | 1 | 1 | 1 |
| Troxerutin | C005865 |  |  | 1 |  |
| Magnolol | C005498 |  | 1 | 1 | 1 |
| Benzothiazole | C005465 |  |  | 1 |  |
| boswellic acids | C0053959 |  |  | 1 |  |
| ringenin | C005273 |  |  | 1 |  |
| MEK | C005222 |  |  | 1 |  |
| metiapine | C005179 |  |  | 1 |  |
| CEP | C005073 |  |  | 1 |  |
| CEPA | C005073 |  |  | 1 |  |
| harmane | C005010 |  |  | 1 |  |
| ferulic acid | C004999 |  |  | 1 |  |
| paxatriol | C004563 |  |  | 1 |  |
| MES | C004550 |  |  | 1 |  |
| Biochanin | C004541 |  |  | 1 |  |
| AGU | C004479 |  |  | 1 |  |
| Traveled | C0040802 |  |  | 1 |  |
| TAT | C0040627 |  |  | 1 |  |
| antipsychotic drug | C0040615 |  | 1 | 1 | 1 |
| Neuroleptic | C0040615 |  | 1 | 1 | 1 |
| neuroleptic | C0040615 |  | 1 | 1 | 1 |
| toxin | C0040549 | 4 | 4 | 1 | 0.25 |
| toxin | C0040549 | 4 | 4 | 1 | 0.25 |
| Total Hip Arthroplasty | C0040508 |  |  | 1 |  |
| SOD1 | C0038838 | 1 |  | 1 |  |
| Resin | C0035191 |  |  | 1 |  |
| rehabilitation program | C0034991 | 1 | 3 | 1 | 0.33333333 |
| rehabilitation treatment | C0034991 | 1 | 3 | 1 | 0.33333333 |
| rehabilitative | C0034991 | 1 | 3 | 1 | 0.33333333 |
| Regulatory Elements | C0034987 |  |  | 1 |  |
| SREBF1 | C0034987 |  |  | 1 |  |
| recreatiol | C0034872 | 2 |  | 1 |  |
| Recreatiol physical activity | C0034872 | 2 |  | 1 |  |
| Prostaglandin EP2 Receptors | C0034835 |  |  | 1 |  |
| Reading Activity | C0034754 |  |  | 1 |  |
| PDHA1 | C0034343 |  |  | 1 |  |
| Pdxk | C0034265 |  |  | 1 |  |
| tetrahydrobiopterin | C003402 | 1 | 1 | 1 | 1 |
| PrPC | C0033164 |  |  | 1 |  |
| phosphodiesterase | C0031640 |  |  | 1 |  |
| 7ketocholesterol | C003001 |  |  | 1 |  |
| monoamine oxidases | C0026454 |  |  | 1 |  |
| MAGL | C0026452 |  |  | 1 |  |
| MAPT | C0026045 |  |  | 1 |  |
| Betulin | C002503 |  |  | 1 |  |
| ligand | C0023688 |  | 1 | 1 | 1 |
| Ketone Body | C0022631 |  |  | 1 |  |
| IL | C0021764 |  |  | 1 |  |
| IL1beta | C0021753 | 1 |  | 1 |  |
| TypeI interferons | C0021743 |  |  | 1 |  |
| IGF2 | C0021666 |  |  | 1 |  |
| IGF1 | C0021665 | 1 |  | 1 |  |
| insulinlike growth factor1 | C0021665 | 1 |  | 1 |  |
| Diabetes drug | C0020616 |  |  | 1 |  |
| hypoglycemic | C0020616 |  |  | 1 |  |
| Triptolide | C001899 | 1 | 1 | 1 | 1 |
| GIP | C0017132 |  |  | 1 |  |
| omega3 polyunsaturated fatty acid | C0015689 | 1 |  | 1 |  |
| omega3 polyunsaturated fatty acids | C0015689 | 1 |  | 1 |  |
| Ape1 | C0014230 |  |  | 1 |  |
| electrical muscle stimulation therapy | C0013787 |  |  | 1 |  |
| electrical brain stimulation | C0013786 |  | 1 | 1 | 1 |
| electrical forepaw stimulation | C0013786 |  | 1 | 1 | 1 |
| pharmacologic treatments | C0013216 |  |  | 1 |  |
| pharmacological treatment | C0013216 |  |  | 1 |  |
| pharmacological treatments | C0013216 |  |  | 1 |  |
| Pharmacotherapy treatment | C0013216 |  |  | 1 |  |
| Drain | C0013103 |  |  | 1 |  |
| DIETARY BIOACTIVE LIPIDS | C0012171 |  |  | 1 |  |
| dairy foods | C0010947 |  |  | 1 |  |
| PKA | C0010531 |  |  | 1 |  |
| Chromen2One | C0010206 |  |  | 1 |  |
| Securinine | C000785 |  |  | 1 |  |
| carboted | C0007026 |  |  | 1 |  |
| Calciumactivated SK channels | C0006685 |  |  | 1 |  |
| Creactive proteins | C0006560 |  |  | 1 |  |
| Botulinum neurotoxin type A | C0006050 |  |  | 1 |  |
| kuromanin | C000596494 |  |  | 1 |  |
| relamorelin | C000593860 |  |  | 1 |  |
| Tetrachlorobenzoquinone | C000591600 |  |  | 1 |  |
| autoantibody | C0004358 |  | 1 | 1 | 1 |
| Autoimmune antibody | C0004358 |  | 1 | 1 | 1 |
| Antiparkinson drug | C0003405 | 4 | 2 | 1 | 0.5 |
| antiParkinson drugs | C0003405 | 4 | 2 | 1 | 0.5 |
| antiparkinsonian drugs | C0003405 | 4 | 2 | 1 | 0.5 |
| Antiparkinsonian medication | C0003405 | 4 | 2 | 1 | 0.5 |
| antiParkinson's disease candidate drug | C0003405 | 4 | 2 | 1 | 0.5 |
| PRX002 | C0003250 |  |  | 1 |  |
| Antiarrhythmics | C0003195 |  |  | 1 |  |
| ACEinhibitors | C0003015 |  |  | 1 |  |
| Mito | C0002475 |  |  | 1 |  |
| Ethanolic | C0001962 |  | 1 | 1 | 1 |
| betablockers | C0001645 |  |  | 1 |  |
| alpha1adrenoceptors | C0001638 |  |  | 1 |  |
| intrasal stem cell administration | C0001560 |  |  | 1 |  |
| acidic | C0001128 |  | 1 | 1 | 1 |
| INTERVENTION TERM | INTERVENTION ID |  |  |  |  |
| CYP2B6 | D065702 |  | 1 |  | 0 |
| prolyl hydroxylase | D064828 |  | 1 |  | 0 |
| Phytochemical | D064209 |  | 1 |  | 0 |
| claudin3 | D062465 |  | 1 |  | 0 |
| Brassinosteroids | D060406 |  | 1 |  | 0 |
| HLADRB1 | D059811 |  | 1 |  | 0 |
| Highfat diet | D059305 |  | 1 |  | 0 |
| Corepressors | D056970 |  | 1 |  | 0 |
| grape seed extract | D056604 |  | 1 |  | 0 |
| Histone deacetylase inhibitors | D056572 |  | 2 |  | 0 |
| tural cytotoxicity triggering receptor 1 | D055627 |  | 1 |  | 0 |
| heat shock protein 27 | D055551 |  | 1 |  | 0 |
| growth differentiation factor 5 | D055428 |  | 1 |  | 0 |
| glutaredoxin 1 | D054477 |  | 1 |  | 0 |
| peroxiredoxin | D054464 |  | 1 |  | 0 |
| presenilin1 | D053764 |  | 1 |  | 0 |
| Pax notoginseng | D053723 |  | 1 |  | 0 |
| IL13Ralpha1 | D053661 |  | 1 |  | 0 |
| interleukin1beta | D053583 |  | 3 |  | 0 |
| osteopontin | D053495 |  | 2 |  | 0 |
| Apolipoprotein E4 | D053327 |  | 1 |  | 0 |
| caspase8 | D053181 |  | 1 |  | 0 |
| caspase 6 | D053178 |  | 1 |  | 0 |
| Xlinked inhibitor of apoptosis protein | D051636 |  | 1 |  | 0 |
| hippocalcin | D051597 |  | 1 |  | 0 |
| Tolllike receptors | D051193 |  | 2 |  | 0 |
| selenoprotein P | D051149 |  | 1 |  | 0 |
| scavenger receptor | D051116 |  | 1 |  | 0 |
| neurturin | D051101 |  | 1 |  | 0 |
| Hsc70 protein | D050883 |  | 1 |  | 0 |
| syptobrevin | D050683 |  | 1 |  | 0 |
| Mutant protein | D050505 |  | 1 |  | 0 |
| antiresorptive agents | D050071 |  | 1 |  | 0 |
| Apoptosis siglregulating kise 1 | D048848 |  | 1 |  | 0 |
| TRAF6 | D048029 |  | 2 |  | 0 |
| peroxisome proliferatoractivated receptor gamma | D047495 |  | 1 |  | 0 |
| thiazolidinediones | D045162 |  | 1 |  | 0 |
| E3 ubiquitin ligase | D044767 |  | 2 |  | 0 |
| canbinoid receptor | D043882 |  | 1 |  | 0 |
| Paraoxose1 | D043303 |  | 1 |  | 0 |
| Laccase | D042845 |  | 1 |  | 0 |
| VEGFA | D042461 |  | 1 |  | 0 |
| Iridoid | D039823 |  | 1 |  | 0 |
| sirtuins | D037761 |  | 1 |  | 0 |
| EphrinA1 | D036382 |  | 1 |  | 0 |
| iron regulatory protein 1 | D035941 |  | 1 |  | 0 |
| Stereospermum | D029402 |  | 1 |  | 0 |
| Acorus gramineus | D029023 |  | 1 |  | 0 |
| herbal preparation | D028321 |  | 1 |  | 0 |
| grape | D027843 |  | 1 |  | 0 |
| serum response factor | D026362 |  | 1 |  | 0 |
| vacuolar ATPase | D025262 |  | 1 |  | 0 |
| Vitamin K2 | D024482 |  | 1 |  | 0 |
| cyclophilin | D021983 |  | 1 |  | 0 |
| nerve growth factor | D020932 |  | 1 |  | 0 |
| mitogenactivated protein kise | D020928 |  | 1 |  | 0 |
| conotoxin | D020916 |  | 1 |  | 0 |
| raclopride | D020891 |  | 2 |  | 0 |
| Neuregulin1 | D020890 |  | 1 |  | 0 |
| Rho GTPases | D020741 |  | 1 |  | 0 |
| glucuronide | D020719 |  | 1 |  | 0 |
| RGS protein | D020710 |  | 2 |  | 0 |
| RGS proteins | D020710 |  | 2 |  | 0 |
| GTPase activating protein | D020690 |  | 1 |  | 0 |
| GTPase | D020558 |  | 1 |  | 0 |
| 3'UTR | D020413 |  | 1 |  | 0 |
| Hormone replacement therapy | D020249 |  | 1 |  | 0 |
| Sodium benzoate | D020160 |  | 1 |  | 0 |
| styrol | D020058 |  | 1 |  | 0 |
| diacylglycerol kise | D019852 |  | 1 |  | 0 |
| Clavulanic acid | D019818 |  | 1 |  | 0 |
| riluzole | D019782 |  | 3 |  | 0 |
| protein disulfide isomerase | D019704 |  | 1 |  | 0 |
| Protein disulfide isomerases | D019704 |  | 1 |  | 0 |
| Cytochrome P450 2E1 | D019392 |  | 1 |  | 0 |
| CYP1A2 | D019388 |  | 1 |  | 0 |
| CYP1A1 | D019363 |  | 1 |  | 0 |
| staurosporine | D019311 |  | 1 |  | 0 |
| MDMA | D018817 |  | 2 |  | 0 |
| glutamate receptor antagonists | D018691 |  | 1 |  | 0 |
| Anticholinergics | D018680 |  | 1 |  | 0 |
| ABC transporters | D018528 |  | 1 |  | 0 |
| hyperoxia | D018496 |  | 1 |  | 0 |
| metabotropic glutamate receptor | D018094 |  | 5 |  | 0 |
| metabotropic glutamate receptors | D018094 |  | 5 |  | 0 |
| GABA receptors | D018079 |  | 1 |  | 0 |
| cD | D018076 |  | 1 |  | 0 |
| substance P receptor | D018040 |  | 1 |  | 0 |
| glycine receptors | D018009 |  | 1 |  | 0 |
| Neurotransmitter receptors | D017981 |  | 1 |  | 0 |
| selective serotonin reuptake inhibitors | D017367 |  | 1 |  | 0 |
| Serotonin reuptake inhibitors | D017367 |  | 1 |  | 0 |
| neurofilament proteins | D016900 |  | 1 |  | 0 |
| arachidote | D016718 |  | 1 |  | 0 |
| bupropion | D016642 |  | 1 |  | 0 |
| amyloid beta peptide | D016229 |  | 2 |  | 0 |
| amyloid betapeptide | D016229 |  | 2 |  | 0 |
| FGF2 | D016222 |  | 2 |  | 0 |
| Fibroblast growth factor 2 | D016222 |  | 2 |  | 0 |
| Mifepristone | D015735 |  | 1 |  | 0 |
| cis retinoic acid | D015474 |  | 1 |  | 0 |
| eicosapentaenoate | D015118 |  | 1 |  | 0 |
| EPA | D015118 |  | 1 |  | 0 |
| OMe | D014934 |  | 1 |  | 0 |
| venom | D014688 |  | 1 |  | 0 |
| Ursodeoxycholic acid | D014580 |  | 1 |  | 0 |
| UDP | D014530 |  | 1 |  | 0 |
| tyrosise | D014442 |  | 1 |  | 0 |
| tyramine | D014439 |  | 1 |  | 0 |
| Tri | D014241 |  | 2 |  | 0 |
| Trichloroethylene | D014241 |  | 2 |  | 0 |
| thioredoxin | D013879 |  | 2 |  | 0 |
| thioredoxin 1 | D013879 |  | 2 |  | 0 |
| thiazole | D013844 |  | 1 |  | 0 |
| Delta(9)THC | D013759 |  | 1 |  | 0 |
| tetrahydrocanbinol | D013759 |  | 1 |  | 0 |
| THC | D013759 |  | 1 |  | 0 |
| Tetracyclines | D013754 |  | 1 |  | 0 |
| tetracycline | D013752 |  | 1 |  | 0 |
| tetrabezine | D013747 |  | 2 |  | 0 |
| Tef | D013721 |  | 2 |  | 0 |
| strychnine | D013331 |  | 1 |  | 0 |
| SRY | D013307 |  | 1 |  | 0 |
| sphingomyelin | D013109 |  | 1 |  | 0 |
| sphingolipid | D013107 |  | 1 |  | 0 |
| Sphingolipids | D013107 |  | 1 |  | 0 |
| sound | D013016 |  | 1 |  | 0 |
| accent | D012970 |  | 1 |  | 0 |
| MSG | D012970 |  | 1 |  | 0 |
| cigarette smoking | D012907 |  | 1 |  | 0 |
| serotonin antagonist | D012702 |  | 1 |  | 0 |
| Rutin | D012431 |  | 1 |  | 0 |
| ruthenium | D012428 |  | 1 |  | 0 |
| serotonin receptors | D011985 |  | 1 |  | 0 |
| Fc receptors | D011961 |  | 1 |  | 0 |
| Estrogen receptors | D011960 |  | 1 |  | 0 |
| quinoxaline | D011810 |  | 1 |  | 0 |
| quizoline | D011799 |  | 1 |  | 0 |
| pyroglutamate | D011761 |  | 1 |  | 0 |
| PRL | D011370 |  | 1 |  | 0 |
| Allopregnolone | D011280 |  | 1 |  | 0 |
| transthyretin | D011228 |  | 1 |  | 0 |
| polychlorited biphenyls | D011078 |  | 2 |  | 0 |
| poly I | D011069 |  | 1 |  | 0 |
| piroxicam | D010894 |  | 1 |  | 0 |
| Piracetam | D010889 |  | 1 |  | 0 |
| phosphocholine | D010767 |  | 1 |  | 0 |
| phosphatases | D010744 |  | 1 |  | 0 |
| phenols | D010636 |  | 1 |  | 0 |
| pergolide | D010479 |  | 1 |  | 0 |
| peptide fragments | D010446 |  | 1 |  | 0 |
| parvalbumin | D010320 |  | 1 |  | 0 |
| oxytocin | D010121 |  | 1 |  | 0 |
| oxidases | D010088 |  | 2 |  | 0 |
| reductase | D010088 |  | 2 |  | 0 |
| Oro | D009963 |  | 1 |  | 0 |
| NDGA | D009637 |  | 1 |  | 0 |
| NO | D009569 |  | 1 |  | 0 |
| phthoquinone | D009285 |  | 1 |  | 0 |
| music therapy | D009147 |  | 1 |  | 0 |
| monocrotophos | D008999 |  | 1 |  | 0 |
| MAO inhibitors | D008996 |  | 1 |  | 0 |
| monoamine oxidase inhibitors | D008996 |  | 1 |  | 0 |
| Methylphenidate | D008774 |  | 5 |  | 0 |
| methyldopa | D008750 |  | 1 |  | 0 |
| memantine | D008559 |  | 1 |  | 0 |
| akatinol | D008559 |  | 1 |  | 0 |
| medicine | D008511 |  | 2 |  | 0 |
| meat | D008460 |  | 2 |  | 0 |
| massage therapy | D008405 |  | 1 |  | 0 |
| Lanosterol | D007810 |  | 1 |  | 0 |
| isoxazole | D007555 |  | 1 |  | 0 |
| Interferongamma | D007371 |  | 1 |  | 0 |
| IGF1 | D007334 |  | 1 |  | 0 |
| immobilization | D007103 |  | 1 |  | 0 |
| Ibuprofen | D007052 |  | 1 |  | 0 |
| honey | D006722 |  | 1 |  | 0 |
| histidine | D006639 |  | 1 |  | 0 |
| histamine Nmethyltransferase | D006637 |  | 1 |  | 0 |
| herbicide | D006540 |  | 1 |  | 0 |
| heparin | D006493 |  | 1 |  | 0 |
| cGMP | D006152 |  | 2 |  | 0 |
| cyclic GMP | D006152 |  | 2 |  | 0 |
| glycoprotein | D006023 |  | 1 |  | 0 |
| glycoproteins | D006023 |  | 1 |  | 0 |
| glycopeptide | D006020 |  | 1 |  | 0 |
| Ginkgo biloba extract | D005976 |  | 1 |  | 0 |
| glial fibrillary acidic protein | D005904 |  | 1 |  | 0 |
| Pax ginseng | D005894 |  | 1 |  | 0 |
| gallate | D005707 |  | 1 |  | 0 |
| Berry | D005638 |  | 1 |  | 0 |
| fruit | D005638 |  | 1 |  | 0 |
| Fruits | D005638 |  | 1 |  | 0 |
| forskolin | D005576 |  | 1 |  | 0 |
| Fluoxetine | D005473 |  | 8 |  | 0 |
| plasmin | D005341 |  | 1 |  | 0 |
| Ferritin | D005293 |  | 1 |  | 0 |
| europium | D005063 |  | 1 |  | 0 |
| dimethyl | D004980 |  | 1 |  | 0 |
| Ergotamine | D004878 |  | 1 |  | 0 |
| ERM | D004878 |  | 1 |  | 0 |
| eggs | D004531 |  | 1 |  | 0 |
| Dopamine betahydroxylase | D004299 |  | 1 |  | 0 |
| domperidone | D004294 |  | 1 |  | 0 |
| diethyldithiocarbamate | D004050 |  | 1 |  | 0 |
| dietary carbohydrate | D004040 |  | 1 |  | 0 |
| diamine | D003959 |  | 1 |  | 0 |
| deoxyribose | D003855 |  | 1 |  | 0 |
| CTP | D003570 |  | 1 |  | 0 |
| Color | D003116 |  | 1 |  | 0 |
| colchicine | D003078 |  | 1 |  | 0 |
| CoA | D003065 |  | 1 |  | 0 |
| clozepam | D002998 |  | 1 |  | 0 |
| cholecystokinin | D002766 |  | 1 |  | 0 |
| chlorogenic acid | D002726 |  | 1 |  | 0 |
| catalase | D002374 |  | 1 |  | 0 |
| cardiac glycosides | D002301 |  | 1 |  | 0 |
| carbazoles | D002227 |  | 2 |  | 0 |
| cane | D002183 |  | 1 |  | 0 |
| calcium channel antagonists | D002121 |  | 3 |  | 0 |
| calcium channel blockers | D002121 |  | 3 |  | 0 |
| chocolate | D002099 |  | 1 |  | 0 |
| Creactive protein | D002097 |  | 1 |  | 0 |
| Buspirone | D002065 |  | 1 |  | 0 |
| bromocriptine | D001971 |  | 5 |  | 0 |
| bromoergocryptine | D001971 |  | 5 |  | 0 |
| Bradykinin | D001920 |  | 1 |  | 0 |
| serum proteins | D001798 |  | 1 |  | 0 |
| betaine | D001622 |  | 1 |  | 0 |
| lindane | D001556 |  | 2 |  | 0 |
| PLK | D001556 |  | 2 |  | 0 |
| benzazepine | D001552 |  | 1 |  | 0 |
| benzamides | D001549 |  | 1 |  | 0 |
| bee venom | D001514 |  | 2 |  | 0 |
| Aroclor | D001140 |  | 1 |  | 0 |
| arginine vasopressin | D001127 |  | 2 |  | 0 |
| tricyclic antidepressants | D000929 |  | 1 |  | 0 |
| angiotensin | D000809 |  | 5 |  | 0 |
| anesthetic | D000777 |  | 1 |  | 0 |
| serpin A1 | D000515 |  | 1 |  | 0 |
| methanol | D000432 |  | 1 |  | 0 |
| albumin | D000418 |  | 2 |  | 0 |
| agmatine | D000376 |  | 2 |  | 0 |
| AMP kise | D000263 |  | 1 |  | 0 |
| cAMP | D000242 |  | 1 |  | 0 |
| cyclic AMP | D000242 |  | 1 |  | 0 |
| auditory stimulation | D000161 |  | 1 |  | 0 |
| acid | D000143 |  | 1 |  | 0 |
| acetyltransferase | D000123 |  | 1 |  | 0 |
| acetyltransferases | D000123 |  | 1 |  | 0 |
| UWA101 | C579241 |  | 1 |  | 0 |
| LLDT67 | C577754 |  | 1 |  | 0 |
| Moracenin D | C574050 |  | 1 |  | 0 |
| VU0364770 | C571150 |  | 1 |  | 0 |
| 11dehydrosinulariolide | C569844 |  | 1 |  | 0 |
| CyPPA | C552889 |  | 1 |  | 0 |
| KYP2047 | C551540 |  | 1 |  | 0 |
| Perampanel | C551441 |  | 2 |  | 0 |
| AV133 | C549477 |  | 1 |  | 0 |
| Obotulinumtoxi | C542870 |  | 1 |  | 0 |
| pyrazolo | C533694 |  | 1 |  | 0 |
| MMP2200 | C531935 |  | 1 |  | 0 |
| Xin | C530716 |  | 1 |  | 0 |
| Bushen Huoxue | C527851 |  | 1 |  | 0 |
| florbetaben | C527756 |  | 1 |  | 0 |
| Gly | C527686 |  | 1 |  | 0 |
| teriflunomide | C527525 |  | 1 |  | 0 |
| hemantane | C526127 |  | 1 |  | 0 |
| HE3286 | C524733 |  | 1 |  | 0 |
| yokukansan | C524644 |  | 1 |  | 0 |
| pyridine | C521421 |  | 1 |  | 0 |
| carboxy | C519986 |  | 4 |  | 0 |
| pitolisant | C516975 |  | 1 |  | 0 |
| TAE684 | C516714 |  | 1 |  | 0 |
| doxanthrine | C516224 |  | 1 |  | 0 |
| first step | C511239 |  | 1 |  | 0 |
| lubiprostone | C506401 |  | 1 |  | 0 |
| secretogranin III | C506167 |  | 1 |  | 0 |
| Thr | C498005 |  | 1 |  | 0 |
| DAT1 | C496402 |  | 1 |  | 0 |
| Tenuigenin | C493220 |  | 1 |  | 0 |
| thiazolyl | C489828 |  | 1 |  | 0 |
| GW5074 | C489251 |  | 1 |  | 0 |
| Stalevo | C481098 |  | 1 |  | 0 |
| fipamezole | C480818 |  | 1 |  | 0 |
| Pardoprunox | C480679 |  | 1 |  | 0 |
| GW0742 | C479979 |  | 1 |  | 0 |
| liver X receptor | C469720 |  | 1 |  | 0 |
| Liver X receptor beta | C469720 |  | 1 |  | 0 |
| dipyridophezine | C443808 |  | 1 |  | 0 |
| Pegylated granulocyte colonystimulating factor | C423652 |  | 1 |  | 0 |
| hydroxyphenyl | C414204 |  | 2 |  | 0 |
| tea polyphenol | C414204 |  | 2 |  | 0 |
| DASB | C412822 |  | 2 |  | 0 |
| ceramide transfer protein | C3853747 |  | 1 |  | 0 |
| granules | C3853628 |  | 1 |  | 0 |
| ErbB4 | C3853568 |  | 1 |  | 0 |
| Selaginella delicatula | C3749808 |  | 1 |  | 0 |
| 5HT1A | C3712806 |  | 1 |  | 0 |
| calcium homeostasis | C3653518 |  | 2 |  | 0 |
| VMAT | C3641892 |  | 1 |  | 0 |
| combitions | C3539957 |  | 1 |  | 0 |
| ABCA5 | C3539865 |  | 1 |  | 0 |
| Silent information regulator 2 | C3539589 |  | 1 |  | 0 |
| others | C3539125 |  | 1 |  | 0 |
| ZFPM2 | C3537374 |  | 1 |  | 0 |
| dihydroxybenzoate | C3535821 |  | 1 |  | 0 |
| Garcinia indica | C3460451 |  | 1 |  | 0 |
| Melissa officilis aqueous extract | C3256424 |  | 1 |  | 0 |
| Mori Cortex | C3152925 |  | 1 |  | 0 |
| HSPB8BAG3 | C2987147 |  | 1 |  | 0 |
| Dickkopf1 | C2987134 |  | 1 |  | 0 |
| Neuromuscular electrical stimulation | C2985393 |  | 1 |  | 0 |
| mitochondrial aldehyde dehydrogeses | C2827577 |  | 1 |  | 0 |
| aversion | C2825146 |  | 1 |  | 0 |
| Xbox binding protein1 | C2825104 |  | 1 |  | 0 |
| XBP1 | C2825104 |  | 1 |  | 0 |
| Glucose regulated protein | C2599779 |  | 1 |  | 0 |
| gene promoter | C2350877 |  | 1 |  | 0 |
| dietary flavonoids | C2348265 |  | 1 |  | 0 |
| infrared laser therapy | C1881207 |  | 1 |  | 0 |
| xCT | C1880211 |  | 1 |  | 0 |
| Flanders | C1875156 |  | 2 |  | 0 |
| Ltype Ca | C1870006 |  | 1 |  | 0 |
| Crerecombise | C1744318 |  | 1 |  | 0 |
| Stereotactic operations | C1735594 |  | 1 |  | 0 |
| antialphasynuclein antibodies | C1624602 |  | 1 |  | 0 |
| ovo | C1623788 |  | 1 |  | 0 |
| PEP1metallothioneinIII protein | C1608757 |  | 1 |  | 0 |
| NRF1 | C1565068 |  | 1 |  | 0 |
| Nramp | C1528325 |  | 1 |  | 0 |
| revised | C1527075 |  | 1 |  | 0 |
| Derivation | C1524082 |  | 1 |  | 0 |
| sequential intranigral administration | C1519252 |  | 1 |  | 0 |
| LINE1 | C1517938 |  | 1 |  | 0 |
| curcuminoids | C1511563 |  | 1 |  | 0 |
| Top IIbeta | C1504636 |  | 1 |  | 0 |
| Fitness | C1456706 |  | 2 |  | 0 |
| TFEB | C1448629 |  | 1 |  | 0 |
| MEK1 | C1434639 |  | 1 |  | 0 |
| AAV2hAADC | C1413943 |  | 1 |  | 0 |
| POLG1 | C1335439 |  | 1 |  | 0 |
| PEP1 | C1335057 |  | 3 |  | 0 |
| PEPHD | C1335057 |  | 3 |  | 0 |
| pharmacological evaluation | C1320678 |  | 1 |  | 0 |
| Ogg1 | C1313359 |  | 1 |  | 0 |
| RING finger protein 11 | C1312851 |  | 1 |  | 0 |
| mTOR | C1307407 |  | 2 |  | 0 |
| polypeptide | C1305923 |  | 1 |  | 0 |
| Chinese herbal medicine | C1273412 |  | 2 |  | 0 |
| cytoprotective agents | C1268903 |  | 1 |  | 0 |
| DPH oxidase 1 | C121788 |  | 2 |  | 0 |
| caprolactone | C121056 |  | 1 |  | 0 |
| Mitochondrial permeability transition pore | C120771 |  | 3 |  | 0 |
| protein component | C1179435 |  | 1 |  | 0 |
| motor proteins | C1179106 |  | 1 |  | 0 |
| HFS | C116938 |  | 1 |  | 0 |
| Complementary and altertive medicine | C1148475 |  | 1 |  | 0 |
| plates | C1139930 |  | 1 |  | 0 |
| eukaryotic translation initiation factor | C1136317 |  | 1 |  | 0 |
| U0126 | C113580 |  | 1 |  | 0 |
| compound K | C112772 |  | 1 |  | 0 |
| Suberoylanilide hydroxamic acid | C111237 |  | 1 |  | 0 |
| Alpinia oxyphylla | C1089993 |  | 1 |  | 0 |
| Gardenia jasminoides | C1089114 |  | 1 |  | 0 |
| Sigma1 receptor | C106106 |  | 2 |  | 0 |
| sigma1 receptors | C106106 |  | 2 |  | 0 |
| 25Hydroxyvitamin D | C104450 |  | 1 |  | 0 |
| D polymerase gamma | C104307 |  | 1 |  | 0 |
| Acanthopax senticosus | C1035215 |  | 1 |  | 0 |
| Retigabine | C101866 |  | 1 |  | 0 |
| Tissue transglutamise | C101201 |  | 1 |  | 0 |
| pellet | C0993610 |  | 1 |  | 0 |
| Frataxin | C098527 |  | 1 |  | 0 |
| flibanserin | C098107 |  | 1 |  | 0 |
| HDM2 | C0971008 |  | 1 |  | 0 |
| SOD2 | C0968147 |  | 1 |  | 0 |
| heat shock cogte 70 interacting protein ( | C0964863 |  | 1 |  | 0 |
| botulinum toxin type B | C096323 |  | 1 |  | 0 |
| RimabotulinumtoxinB | C096323 |  | 1 |  | 0 |
| fibroblast growth factor 20 | C0962069 |  | 2 |  | 0 |
| Fibroblast growth factor20 | C0962069 |  | 2 |  | 0 |
| TFAM | C0960756 |  | 1 |  | 0 |
| Nociceptin | C096012 |  | 1 |  | 0 |
| NOP | C096012 |  | 1 |  | 0 |
| orphanin FQ | C096012 |  | 1 |  | 0 |
| mitochondrial chaperone protein | C0949610 |  | 2 |  | 0 |
| Mitochondrial CHCHDContaining Proteins | C0949610 |  | 2 |  | 0 |
| Mitochondrial fission proteins | C0949610 |  | 2 |  | 0 |
| Ranbp2 | C094493 |  | 1 |  | 0 |
| Ziprasidone | C092292 |  | 1 |  | 0 |
| phenserine | C092280 |  | 1 |  | 0 |
| posiphen | C092280 |  | 1 |  | 0 |
| Dopaminochrome | C091584 |  | 1 |  | 0 |
| Tollip | C0915585 |  | 1 |  | 0 |
| Chlorotoxin | C091539 |  | 1 |  | 0 |
| alpha3beta4 ChRs | C0911770 |  | 1 |  | 0 |
| focal ultra | C0910498 |  | 1 |  | 0 |
| Rosiglitazone | C089730 |  | 3 |  | 0 |
| CuATSM | C089548 |  | 1 |  | 0 |
| Altertive oxidase | C088813 |  | 1 |  | 0 |
| single molecule | C0872367 |  | 1 |  | 0 |
| singlemolecule | C0872367 |  | 1 |  | 0 |
| G proteincoupled receptor kises | C0872043 |  | 1 |  | 0 |
| Bright light therapy | C0870230 |  | 1 |  | 0 |
| MAP1B | C085284 |  | 1 |  | 0 |
| telmisartan | C084178 |  | 1 |  | 0 |
| heat shock protein 70.1 | C081884 |  | 1 |  | 0 |
| toxic proteins | C0815048 |  | 1 |  | 0 |
| Protein Function | C0815043 |  | 1 |  | 0 |
| val | C081489 |  | 2 |  | 0 |
| Environmental tobacco smoke | C0813971 |  | 1 |  | 0 |
| 7nitroindazole | C080122 |  | 1 |  | 0 |
| Pyrazolopyridines | C0766039 |  | 1 |  | 0 |
| LRP6 | C0765067 |  | 1 |  | 0 |
| Fluazim | C075780 |  | 1 |  | 0 |
| Sodium phenylbutyrate | C075773 |  | 1 |  | 0 |
| PBP | C0755397 |  | 1 |  | 0 |
| erucin | C073539 |  | 1 |  | 0 |
| constituents | C0729650 |  | 1 |  | 0 |
| templates | C0728990 |  | 1 |  | 0 |
| MG132 | C072553 |  | 1 |  | 0 |
| MG132 | C072553 |  | 1 |  | 0 |
| carbamic acid | C070766 |  | 1 |  | 0 |
| quetiapine | C069541 |  | 1 |  | 0 |
| Receptor for advanced glycation endproducts | C068348 |  | 1 |  | 0 |
| CAE | C067796 |  | 1 |  | 0 |
| Operatiol plasticity | C0677616 |  | 1 |  | 0 |
| metabotropic glutamate receptor subtype 4 | C0667286 |  | 1 |  | 0 |
| Torsi | C0665823 |  | 1 |  | 0 |
| nephrocizin | C066408 |  | 1 |  | 0 |
| IkappaB kise family | C0663914 |  | 2 |  | 0 |
| IKK | C0663914 |  | 2 |  | 0 |
| Salvianolic acid A | C066201 |  | 1 |  | 0 |
| Meloxicam | C065757 |  | 1 |  | 0 |
| atorvastatin | C065179 |  | 1 |  | 0 |
| FLB457 | C065065 |  | 1 |  | 0 |
| C2ceramide | C064769 |  | 1 |  | 0 |
| MMP | C0623362 |  | 1 |  | 0 |
| dihydrexidine | C061532 |  | 1 |  | 0 |
| Abeta peptide | C0611285 |  | 1 |  | 0 |
| toxicants | C0599787 |  | 2 |  | 0 |
| monoamine | C0599682 |  | 2 |  | 0 |
| magnesium ions | C0596876 |  | 1 |  | 0 |
| tissueengineered | C0596171 |  | 1 |  | 0 |
| aiding speech therapy | C0596052 |  | 1 |  | 0 |
| GSM | C059554 |  | 1 |  | 0 |
| Posterior spil fusion | C0582026 |  | 1 |  | 0 |
| revascularization | C0581603 |  | 1 |  | 0 |
| bafilomycin A | C057620 |  | 1 |  | 0 |
| epigallocatechin | C057580 |  | 1 |  | 0 |
| Theaflavin | C056068 |  | 2 |  | 0 |
| IBZM | C055743 |  | 1 |  | 0 |
| iodobenzamide | C055743 |  | 1 |  | 0 |
| boswellic acid | C054625 |  | 1 |  | 0 |
| metalloproteises 1 | C0539420 |  | 1 |  | 0 |
| uncoupling protein 2 | C0536847 |  | 1 |  | 0 |
| dopamine quinones | C0536449 |  | 1 |  | 0 |
| onestep | C0534010 |  | 1 |  | 0 |
| angiotensin type 1 receptors | C0529330 |  | 1 |  | 0 |
| material | C0520510 |  | 1 |  | 0 |
| materials | C0520510 |  | 1 |  | 0 |
| ginsenoside Re | C049864 |  | 1 |  | 0 |
| Lamotrigine | C047781 |  | 1 |  | 0 |
| NPF | C047034 |  | 1 |  | 0 |
| PTIQ | C046152 |  | 1 |  | 0 |
| Automated method | C0456979 |  | 1 |  | 0 |
| leflunomide | C045463 |  | 1 |  | 0 |
| white chocolate | C0453439 |  | 1 |  | 0 |
| lemon juice | C0452457 |  | 1 |  | 0 |
| general population sample | C0444062 |  | 1 |  | 0 |
| specific antibodies | C0443640 |  | 1 |  | 0 |
| ApoE isoform | C0443526 |  | 1 |  | 0 |
| octacosanol | C044309 |  | 1 |  | 0 |
| prenyl diphosphate | C043060 |  | 1 |  | 0 |
| olive oil | C042763 |  | 1 |  | 0 |
| medical therapy | C0418981 |  | 1 |  | 0 |
| Eumelanin | C041877 |  | 1 |  | 0 |
| rosmarinic acid | C041376 |  | 1 |  | 0 |
| Isoliquiritigenin | C040920 |  | 1 |  | 0 |
| arterial spin labeled perfusion | C0398345 |  | 1 |  | 0 |
| Calpain inhibitor | C039304 |  | 1 |  | 0 |
| calpain inhibitors | C039304 |  | 1 |  | 0 |
| medin | C0388561 |  | 1 |  | 0 |
| Dihydropyridine | C038806 |  | 1 |  | 0 |
| Baicalin | C038044 |  | 1 |  | 0 |
| 5HT1A receptors | C0379900 |  | 1 |  | 0 |
| marc | C0379885 |  | 1 |  | 0 |
| recombint human erythropoietin | C0376541 |  | 1 |  | 0 |
| dichlorofluorescein | C037631 |  | 1 |  | 0 |
| lung volume reduction surgery | C0375931 |  | 1 |  | 0 |
| PFS | C037145 |  | 1 |  | 0 |
| obovatol | C036795 |  | 1 |  | 0 |
| norfluoxetine | C036139 |  | 1 |  | 0 |
| levodopa methyl ester | C035420 |  | 1 |  | 0 |
| piperazin | C034930 |  | 1 |  | 0 |
| instruments | C0348000 |  | 1 |  | 0 |
| catechol | C034221 |  | 1 |  | 0 |
| Harpagoside | C033249 |  | 1 |  | 0 |
| Oxicam | C032801 |  | 1 |  | 0 |
| DFT | C032788 |  | 1 |  | 0 |
| fumaric acid | C032005 |  | 1 |  | 0 |
| MRF | C0312443 |  | 1 |  | 0 |
| Human albumin | C0304925 |  | 1 |  | 0 |
| fluid | C0302908 |  | 1 |  | 0 |
| Fluids | C0302908 |  | 1 |  | 0 |
| DCFDA | C029569 |  | 1 |  | 0 |
| mTOR | C0293060 |  | 2 |  | 0 |
| pololike kise | C0290178 |  | 1 |  | 0 |
| Nrf2 | C0289507 |  | 1 |  | 0 |
| Nuclear factor erythroid 2related factor | C0289507 |  | 1 |  | 0 |
| pace | C0287990 |  | 1 |  | 0 |
| CFT | C0280462 |  | 1 |  | 0 |
| replacement therapy | C0279033 |  | 4 |  | 0 |
| ligustilide | C027820 |  | 1 |  | 0 |
| 4hydroxynonel | C027576 |  | 2 |  | 0 |
| HNE | C027576 |  | 2 |  | 0 |
| butylphthalide | C027125 |  | 1 |  | 0 |
| 4EBP1 | C0254260 |  | 1 |  | 0 |
| growth/differentiation factor 5 | C0253373 |  | 1 |  | 0 |
| huntingtin | C0252274 |  | 2 |  | 0 |
| DMG | C025138 |  | 1 |  | 0 |
| GSK3B | C0244988 |  | 1 |  | 0 |
| ADP | C024376 |  | 1 |  | 0 |
| opioid | C0242402 |  | 1 |  | 0 |
| flavin | C024132 |  | 1 |  | 0 |
| glutamate | C0220839 |  | 1 |  | 0 |
| protein kise Cdelta | C0217250 |  | 1 |  | 0 |
| p25 | C0215955 |  | 3 |  | 0 |
| TPPP/p25 | C0215955 |  | 3 |  | 0 |
| SFPQ | C0214345 |  | 1 |  | 0 |
| PEDF | C0213321 |  | 1 |  | 0 |
| betaCFT | C021279 |  | 1 |  | 0 |
| mGlu receptor | C0206529 |  | 2 |  | 0 |
| mGluRs | C0206529 |  | 2 |  | 0 |
| receptor tyrosine kise | C0206364 |  | 1 |  | 0 |
| general practice partnership. Short nursing care | C0204650 |  | 1 |  | 0 |
| Stroke rehabilitation | C0204097 |  | 1 |  | 0 |
| packing | C0184967 |  | 2 |  | 0 |
| pheomelanin | C018362 |  | 1 |  | 0 |
| simulator | C0183309 |  | 1 |  | 0 |
| gamma knife | C0182639 |  | 1 |  | 0 |
| fisetin | C017875 |  | 1 |  | 0 |
| dopamine D2 partial agonist | C0178601 |  | 2 |  | 0 |
| dopamine D3 agonist | C0178601 |  | 2 |  | 0 |
| Dopamine D3 receptorpreferring agonists | C0178601 |  | 2 |  | 0 |
| Protein structure | C0175168 |  | 1 |  | 0 |
| MAPK1 | C0170168 |  | 1 |  | 0 |
| Sulforaphane | C016766 |  | 2 |  | 0 |
| methylmercury | C0166979 |  | 1 |  | 0 |
| diacetate | C016635 |  | 1 |  | 0 |
| matrix metalloproteise3 | C0164371 |  | 1 |  | 0 |
| ubiquitin carboxyltermil hydrolase | C0164005 |  | 2 |  | 0 |
| UCH | C0164005 |  | 2 |  | 0 |
| UCHL1 | C0164005 |  | 2 |  | 0 |
| chimeric protein | C0162768 |  | 1 |  | 0 |
| fusion protein | C0162768 |  | 1 |  | 0 |
| Schisandrin B | C015499 |  | 1 |  | 0 |
| Nitrones | C015388 |  | 1 |  | 0 |
| Cutting | C0152060 |  | 3 |  | 0 |
| cutting | C0152060 |  | 3 |  | 0 |
| cognitive stimulation | C0150174 |  | 1 |  | 0 |
| decreased anxiety | C0150135 |  | 1 |  | 0 |
| TIMPs 1 | C0145947 |  | 1 |  | 0 |
| vinpocetine | C013983 |  | 1 |  | 0 |
| pyrroline | C013231 |  | 1 |  | 0 |
| nimesulide | C012655 |  | 1 |  | 0 |
| GFP | C0120285 |  | 2 |  | 0 |
| ifenprodil | C010739 |  | 1 |  | 0 |
| norharman | C010262 |  | 1 |  | 0 |
| NRH | C010262 |  | 1 |  | 0 |
| Dimebon | C010119 |  | 1 |  | 0 |
| REM | C0086964 |  | 1 |  | 0 |
| intravenous immunoglobulin | C0085297 |  | 2 |  | 0 |
| trkB Receptors | C0084873 |  | 1 |  | 0 |
| vascular factors | C0078057 |  | 1 |  | 0 |
| Fucoidan | C007789 |  | 1 |  | 0 |
| tumor necrosis factor alpha death receptor | C0077503 |  | 1 |  | 0 |
| theaflavins | C0076379 |  | 1 |  | 0 |
| Neurokinin receptor | C0075372 |  | 1 |  | 0 |
| Deoxy | C0069851 |  | 1 |  | 0 |
| DPH oxidases | C0068355 |  | 1 |  | 0 |
| NOX | C0068355 |  | 1 |  | 0 |
| Mitochondrial neurol uncoupling proteins | C0066604 |  | 1 |  | 0 |
| kaempferol | C006552 |  | 1 |  | 0 |
| LRP8 | C0065059 |  | 1 |  | 0 |
| IAPP | C0063684 |  | 1 |  | 0 |
| Imidazoleacetic acid | C005954 |  | 1 |  | 0 |
| astaxanthin | C005948 |  | 1 |  | 0 |
| DARPP32 | C0057146 |  | 1 |  | 0 |
| dopamine and cAMPregulated phosphoprotein32 | C0057146 |  | 1 |  | 0 |
| cofilin | C0056080 |  | 1 |  | 0 |
| phthazarin | C005503 |  | 1 |  | 0 |
| honokiol | C005499 |  | 1 |  | 0 |
| ftazone | C005272 |  | 1 |  | 0 |
| GABA(B) receptor | C0051610 |  | 1 |  | 0 |
| waters | C0043047 |  | 1 |  | 0 |
| vehicles | C0042444 |  | 1 |  | 0 |
| inoculation | C0042196 |  | 1 |  | 0 |
| uPA | C0042071 |  | 1 |  | 0 |
| Transposition | C0040674 |  | 1 |  | 0 |
| Environmental toxins | C0040538 |  | 1 |  | 0 |
| tame | C0040494 |  | 1 |  | 0 |
| MS# | C0039676 |  | 1 |  | 0 |
| TatDJ1 protein | C0039341 |  | 1 |  | 0 |
| swims | C0039003 |  | 1 |  | 0 |
| lung reduction surgery | C0038903 |  | 1 |  | 0 |
| mitochondrial complex II | C0038615 |  | 1 |  | 0 |
| sesame seed oil | C0036845 |  | 1 |  | 0 |
| S129 | C0036720 |  | 2 |  | 0 |
| serines | C0036720 |  | 2 |  | 0 |
| GABA(A) receptors | C0034807 |  | 1 |  | 0 |
| estrogen receptor | C0034804 |  | 1 |  | 0 |
| Oestrogen receptors | C0034804 |  | 1 |  | 0 |
| group singing therapy | C0033971 |  | 1 |  | 0 |
| prostaglandin J( | C0033554 |  | 1 |  | 0 |
| Polymeric | C0032521 |  | 1 |  | 0 |
| placebo | C0032042 |  | 2 |  | 0 |
| Phosphodiesterase 10 inhibitors | C0031638 |  | 1 |  | 0 |
| phosphodiesterase 7 inhibitors | C0031638 |  | 1 |  | 0 |
| WINCS | C0027908 |  | 1 |  | 0 |
| DTdiaphorase | C0027280 |  | 1 |  | 0 |
| monoamine oxidaseB | C0026456 |  | 1 |  | 0 |
| costunolide | C002602 |  | 1 |  | 0 |
| LRRK2 | C0023401 |  | 1 |  | 0 |
| laminins | C0022984 |  | 1 |  | 0 |
| glyoxalase | C0022956 |  | 1 |  | 0 |
| glyoxalases | C0022956 |  | 1 |  | 0 |
| interferon gamma | C0021740 |  | 1 |  | 0 |
| transmembrane protein | C0021699 |  | 1 |  | 0 |
| organochlorines | C0020247 |  | 1 |  | 0 |
| ganglioside GM1 | C0016895 |  | 1 |  | 0 |
| fructus | C0016767 |  | 1 |  | 0 |
| fruitful | C0016767 |  | 1 |  | 0 |
| SPCA | C0015502 |  | 1 |  | 0 |
| exonic | C0015295 |  | 1 |  | 0 |
| eIF2alpha | C0013733 |  | 1 |  | 0 |
| dolichols | C0012990 |  | 1 |  | 0 |
| ENS | C001195 |  | 1 |  | 0 |
| FDG | C0011501 |  | 1 |  | 0 |
| CAG | C0010843 |  | 1 |  | 0 |
| pill | C0009905 |  | 1 |  | 0 |
| CART | C0009170 |  | 1 |  | 0 |
| CNS Drug | C0007680 |  | 1 |  | 0 |
| catechol0methyltransferase | C0007407 |  | 2 |  | 0 |
| COMT | C0007407 |  | 2 |  | 0 |
| GSK2578215A | C000600930 |  | 1 |  | 0 |
| Behavioral Treatment | C0004933 |  | 1 |  | 0 |
| antibody | C0003241 |  | 3 |  | 0 |
| base | C0002055 |  | 1 |  | 0 |
| methanolic | C0001963 |  | 1 |  | 0 |
| Nacetyl cysteine | C0001047 |  | 2 |  | 0 |
