# Supplementary material for: Menagerie: A text-mining tool to support animal-human translation in neurodegeneration research
Source: PLoS One. 2019 Dec 17;14(12):e0226176. doi: 10.1371/journal.pone.0226176 (PMC6917268; doi:10.1371/journal.pone.0226176)
Supplement: S3 Table — These are extracted from the entire abstract. Multiple synonyms describing the same entity map to a common UMLS or NCBI identifier. (DOCX) [file pone.0226176.s004.docx]

**S3 Table:** Gene mentions extracted over 10 years. These are extracted from the entire abstract. Multiple synonyms describing the same entity map to a common UMLS or NCBI identifier.

| **Text term** | **NCBI/UMLS identifier** | **2008** | **2012** | **2017** | **2017/2012** |
| --- | --- | --- | --- | --- | --- |
| Alpha-synuclein | 6622 | 263 | 368 | 522 | 1.41847826086957 |
| alpha-synuclein gene | 6622 | 263 | 368 | 522 | 1.41847826086957 |
| SNCA | 6622 | 263 | 368 | 522 | 1.41847826086957 |
| SNCA gene | 6622 | 263 | 368 | 522 | 1.41847826086957 |
| alpha-synuclein (alphaSYN) gene | 6622 | 263 | 368 | 522 | 1.41847826086957 |
| alphaSYN | 6622 | 263 | 368 | 522 | 1.41847826086957 |
| AS | 6622 | 263 | 368 | 522 | 1.41847826086957 |
| alpha-syn | 6622 | 263 | 368 | 522 | 1.41847826086957 |
| alphaS | 6622 | 263 | 368 | 522 | 1.41847826086957 |
| alpha-synuclein A53T mutant gene | 6622 | 263 | 368 | 522 | 1.41847826086957 |
| alpha Syn | 6622 | 263 | 368 | 522 | 1.41847826086957 |
| NACP | 6622 | 263 | 368 | 522 | 1.41847826086957 |
| alpha-syn gene | 6622 | 263 | 368 | 522 | 1.41847826086957 |
| alpha- synuclein | 6622 | 263 | 368 | 522 | 1.41847826086957 |
| NAC | 6622 | 263 | 368 | 522 | 1.41847826086957 |
| PARK1-13 | 6622 | 263 | 368 | 522 | 1.41847826086957 |
| PARK1 | 6622 | 263 | 368 | 522 | 1.41847826086957 |
| aSyn | 6622 | 263 | 368 | 522 | 1.41847826086957 |
| PARK4 | 6622 | 263 | 368 | 522 | 1.41847826086957 |
| PD1 | 6622 | 263 | 368 | 522 | 1.41847826086957 |
| alpha-synuclein-encoding SNCA gene | 6622 | 263 | 368 | 522 | 1.41847826086957 |
| hSNCA | 6622 | 263 | 368 | 522 | 1.41847826086957 |
| SNCA-140 | 6622 | 263 | 368 | 522 | 1.41847826086957 |
| SNCA-126 | 6622 | 263 | 368 | 522 | 1.41847826086957 |
| Alpha synuclein | 6622 | 263 | 368 | 522 | 1.41847826086957 |
| alphaSynuclein | 6622 | 263 | 368 | 522 | 1.41847826086957 |
| alpha-synuclein (SNCA) gene | 6622 | 263 | 368 | 522 | 1.41847826086957 |
| SNCA genes | 6622 | 263 | 368 | 522 | 1.41847826086957 |
| SNCA's | 6622 | 263 | 368 | 522 | 1.41847826086957 |
| a-syn | 6622 | 263 | 368 | 522 | 1.41847826086957 |
| alpha-synuclein encoding gene | 6622 | 263 | 368 | 522 | 1.41847826086957 |
| alpha-synuclein-mediated genetic | 6622 | 263 | 368 | 522 | 1.41847826086957 |
| alpha-Synuclein genetic | 6622 | 263 | 368 | 522 | 1.41847826086957 |
| PARK1-15 | 6622 | 263 | 368 | 522 | 1.41847826086957 |
| alpha-synuclein (SNCA) genes | 6622 | 263 | 368 | 522 | 1.41847826086957 |
| alpha-synuclein genes | 6622 | 263 | 368 | 522 | 1.41847826086957 |
| alpha synuclein gene | 6622 | 263 | 368 | 522 | 1.41847826086957 |
| alpha-S | 6622 | 263 | 368 | 522 | 1.41847826086957 |
| aSN | 6622 | 263 | 368 | 522 | 1.41847826086957 |
| alpha-synuclein's | 6622 | 263 | 368 | 522 | 1.41847826086957 |
| synuclein, alpha | 6622 | 263 | 368 | 522 | 1.41847826086957 |
| alpha-synuclein A53T gene | 6622 | 263 | 368 | 522 | 1.41847826086957 |
| alpha- Syn | 6622 | 263 | 368 | 522 | 1.41847826086957 |
| synuclein alpha | 6622 | 263 | 368 | 522 | 1.41847826086957 |
| SNCA126 | 6622 | 263 | 368 | 522 | 1.41847826086957 |
| ASP | 6622 | 263 | 368 | 522 | 1.41847826086957 |
| A53T-SNCA | 6622 | 263 | 368 | 522 | 1.41847826086957 |
| alpha-synuclein propagation | 6622 | 263 | 368 | 522 | 1.41847826086957 |
| Alpha - synuclein | 6622 | 263 | 368 | 522 | 1.41847826086957 |
| alpha-synuclein pathology propagation | 6622 | 263 | 368 | 522 | 1.41847826086957 |
| alpha-synuclein cell-to-cell propagation | 6622 | 263 | 368 | 522 | 1.41847826086957 |
| alpha-synuclein intercellular propagation | 6622 | 263 | 368 | 522 | 1.41847826086957 |
| alpha-SYN encoding gene | 6622 | 263 | 368 | 522 | 1.41847826086957 |
| a-synuclein | 6622 | 263 | 368 | 522 | 1.41847826086957 |
| SNCAA53T | 6622 | 263 | 368 | 522 | 1.41847826086957 |
| PASH | 6622 | 263 | 368 | 522 | 1.41847826086957 |
| Synuclein Alpha Non A4 Component of Amyloid Precursor | 6622 | 263 | 368 | 522 | 1.41847826086957 |
| alpha-SN | 6622 | 263 | 368 | 522 | 1.41847826086957 |
| SNCA-3 | 6622 | 263 | 368 | 522 | 1.41847826086957 |
| tyrosine hydroxylase | 7054 | 134 | 129 | 162 | 1.25581395348837 |
| TH | 7054 | 134 | 129 | 162 | 1.25581395348837 |
| TH(+) | 7054 | 134 | 129 | 162 | 1.25581395348837 |
| TH(+ | 7054 | 134 | 129 | 162 | 1.25581395348837 |
| TH+ | 7054 | 134 | 129 | 162 | 1.25581395348837 |
| TH-IR | 7054 | 134 | 129 | 162 | 1.25581395348837 |
| THir | 7054 | 134 | 129 | 162 | 1.25581395348837 |
| INS-TH | 7054 | 134 | 129 | 162 | 1.25581395348837 |
| bovine growth hormone | 7054 | 134 | 129 | 162 | 1.25581395348837 |
| thrombin | 7054 | 134 | 129 | 162 | 1.25581395348837 |
| neuronal nitric oxide synthase | 7054 | 134 | 129 | 162 | 1.25581395348837 |
| nitric oxide synthase | 7054 | 134 | 129 | 162 | 1.25581395348837 |
| glycogen synthase kinase-3 | 7054 | 134 | 129 | 162 | 1.25581395348837 |
| TH gene | 7054 | 134 | 129 | 162 | 1.25581395348837 |
| TH+] | 7054 | 134 | 129 | 162 | 1.25581395348837 |
| glutathione-S-transferase | 7054 | 134 | 129 | 162 | 1.25581395348837 |
| alpha-methyl-p-tyrosine | 7054 | 134 | 129 | 162 | 1.25581395348837 |
| alphaMT | 7054 | 134 | 129 | 162 | 1.25581395348837 |
| erythropoietin | 7054 | 134 | 129 | 162 | 1.25581395348837 |
| inducible nitric oxide synthase (iNOS) protein | 7054 | 134 | 129 | 162 | 1.25581395348837 |
| hTH | 7054 | 134 | 129 | 162 | 1.25581395348837 |
| TH-SDHD | 7054 | 134 | 129 | 162 | 1.25581395348837 |
| the SAC | 7054 | 134 | 129 | 162 | 1.25581395348837 |
| TH-NTN | 7054 | 134 | 129 | 162 | 1.25581395348837 |
| nuclear factor erythroid 2-related factor | 7054 | 134 | 129 | 162 | 1.25581395348837 |
| DTH | 7054 | 134 | 129 | 162 | 1.25581395348837 |
| TH genes | 7054 | 134 | 129 | 162 | 1.25581395348837 |
| glutathione peroxidase | 7054 | 134 | 129 | 162 | 1.25581395348837 |
| LRRK2 | 120892 | 69 | 123 | 117 | 0.951219512195122 |
| Leucine-rich repeat kinase 2 (LRRK2) gene | 120892 | 69 | 123 | 117 | 0.951219512195122 |
| LRRK2 gene | 120892 | 69 | 123 | 117 | 0.951219512195122 |
| Leucine-rich repeat kinase 2 | 120892 | 69 | 123 | 117 | 0.951219512195122 |
| Leucine-Rich Repeat Kinase 2 gene | 120892 | 69 | 123 | 117 | 0.951219512195122 |
| leucine rich repeat kinase 2 | 120892 | 69 | 123 | 117 | 0.951219512195122 |
| dardarin | 120892 | 69 | 123 | 117 | 0.951219512195122 |
| PARK8 | 120892 | 69 | 123 | 117 | 0.951219512195122 |
| leucine-rich repeat kinase-2 | 120892 | 69 | 123 | 117 | 0.951219512195122 |
| leucine-rich-repeat kinase 2 gene | 120892 | 69 | 123 | 117 | 0.951219512195122 |
| LRRK2 genes | 120892 | 69 | 123 | 117 | 0.951219512195122 |
| LRRK2 dardarin gene | 120892 | 69 | 123 | 117 | 0.951219512195122 |
| human LRRK2 | 120892 | 69 | 123 | 117 | 0.951219512195122 |
| leucine-rich repeat kinase (LRRK)2 genes | 120892 | 69 | 123 | 117 | 0.951219512195122 |
| leucine-rich repeat kinase2 | 120892 | 69 | 123 | 117 | 0.951219512195122 |
| LRRK2-regulated genes | 120892 | 69 | 123 | 117 | 0.951219512195122 |
| leucine-rich repeat kinase-2 (LRRK2) gene | 120892 | 69 | 123 | 117 | 0.951219512195122 |
| LRRK2 p | 120892 | 69 | 123 | 117 | 0.951219512195122 |
| LRRK-2 genes | 120892 | 69 | 123 | 117 | 0.951219512195122 |
| LRRK-2 | 120892 | 69 | 123 | 117 | 0.951219512195122 |
| LRRK2's | 120892 | 69 | 123 | 117 | 0.951219512195122 |
| G2019S-LRRK2 | 120892 | 69 | 123 | 117 | 0.951219512195122 |
| Leucine rich repeat kinase type 2 | 120892 | 69 | 123 | 117 | 0.951219512195122 |
| Lrrk2-KD | 120892 | 69 | 123 | 117 | 0.951219512195122 |
| LRRK.2 | 120892 | 69 | 123 | 117 | 0.951219512195122 |
| LRRK2-PD | 120892 | 69 | 123 | 117 | 0.951219512195122 |
| LRRK2 genetic | 120892 | 69 | 123 | 117 | 0.951219512195122 |
| leucine-rich-repeat kinase 2 | 120892 | 69 | 123 | 117 | 0.951219512195122 |
| human leucine-rich-repeat kinase 2 | 120892 | 69 | 123 | 117 | 0.951219512195122 |
| LRRK2-WT | 120892 | 69 | 123 | 117 | 0.951219512195122 |
| LRRK2-R1441G | 120892 | 69 | 123 | 117 | 0.951219512195122 |
| hLRRK2 | 120892 | 69 | 123 | 117 | 0.951219512195122 |
| leucine-rich repeat protein kinase 2 | 120892 | 69 | 123 | 117 | 0.951219512195122 |
| pLRRK2 | 120892 | 69 | 123 | 117 | 0.951219512195122 |
| LRRK2-G2019S | 120892 | 69 | 123 | 117 | 0.951219512195122 |
| LRRK2-R1441C | 120892 | 69 | 123 | 117 | 0.951219512195122 |
| LRRK2-encoding genes | 120892 | 69 | 123 | 117 | 0.951219512195122 |
| leucine-rich repeat kinase 2 (LRRK2)/glucocerebrosidase gene | 120892 | 69 | 123 | 117 | 0.951219512195122 |
| Leucine-rich repeat kinase 2 (LRRK2)-gene | 120892 | 69 | 123 | 117 | 0.951219512195122 |
| LRRK2 G2019S gene | 120892 | 69 | 123 | 117 | 0.951219512195122 |
| LRRK2G2019S | 120892 | 69 | 123 | 117 | 0.951219512195122 |
| human G2019S-LRRK2 | 120892 | 69 | 123 | 117 | 0.951219512195122 |
| parkin | 5071 | 60 | 79 | 99 | 1.25316455696203 |
| PARK 2 | 5071 | 60 | 79 | 99 | 1.25316455696203 |
| parkin gene | 5071 | 60 | 79 | 99 | 1.25316455696203 |
| E3 ubiquitin ligases | 5071 | 60 | 79 | 99 | 1.25316455696203 |
| PARK2 | 5071 | 60 | 79 | 99 | 1.25316455696203 |
| E3 ubiquitin ligase | 5071 | 60 | 79 | 99 | 1.25316455696203 |
| PRKN | 5071 | 60 | 79 | 99 | 1.25316455696203 |
| E3 ubiquitin-protein ligase parkin | 5071 | 60 | 79 | 99 | 1.25316455696203 |
| parkin genes | 5071 | 60 | 79 | 99 | 1.25316455696203 |
| Parkin Co-Regulated Gene | 5071 | 60 | 79 | 99 | 1.25316455696203 |
| Parkin-co-regulated gene | 5071 | 60 | 79 | 99 | 1.25316455696203 |
| Park2 gene | 5071 | 60 | 79 | 99 | 1.25316455696203 |
| AR-JP | 5071 | 60 | 79 | 99 | 1.25316455696203 |
| parkin (PARK2) gene- | 5071 | 60 | 79 | 99 | 1.25316455696203 |
| E3 ubiquitin-ligase | 5071 | 60 | 79 | 99 | 1.25316455696203 |
| PARK2 protein | 5071 | 60 | 79 | 99 | 1.25316455696203 |
| PARK2 proteins | 5071 | 60 | 79 | 99 | 1.25316455696203 |
| parkin's | 5071 | 60 | 79 | 99 | 1.25316455696203 |
| PARK | 5071 | 60 | 79 | 99 | 1.25316455696203 |
| PARK2 genes | 5071 | 60 | 79 | 99 | 1.25316455696203 |
| PARK2 genetic | 5071 | 60 | 79 | 99 | 1.25316455696203 |
| PARK2's | 5071 | 60 | 79 | 99 | 1.25316455696203 |
| E3 ubiquitin protein ligase parkin | 5071 | 60 | 79 | 99 | 1.25316455696203 |
| pdr-1 | 5071 | 60 | 79 | 99 | 1.25316455696203 |
| tau | 4137 | 28 | 88 | 92 | 1.04545454545455 |
| microtubule-associated protein tau | 4137 | 28 | 88 | 92 | 1.04545454545455 |
| tau protein | 4137 | 28 | 88 | 92 | 1.04545454545455 |
| MAPT | 4137 | 28 | 88 | 92 | 1.04545454545455 |
| microtubule-associated protein tau gene | 4137 | 28 | 88 | 92 | 1.04545454545455 |
| MAPT gene | 4137 | 28 | 88 | 92 | 1.04545454545455 |
| Saitohin | 4137 | 28 | 88 | 92 | 1.04545454545455 |
| STH | 4137 | 28 | 88 | 92 | 1.04545454545455 |
| PPND | 4137 | 28 | 88 | 92 | 1.04545454545455 |
| tau40 | 4137 | 28 | 88 | 92 | 1.04545454545455 |
| MAPT genes | 4137 | 28 | 88 | 92 | 1.04545454545455 |
| FTDP-17 | 4137 | 28 | 88 | 92 | 1.04545454545455 |
| microtubule-associated protein tau (MAPT) gene | 4137 | 28 | 88 | 92 | 1.04545454545455 |
| microtubule-binding protein tau | 4137 | 28 | 88 | 92 | 1.04545454545455 |
| microtubule associated protein tau | 4137 | 28 | 88 | 92 | 1.04545454545455 |
| human Tau protein | 4137 | 28 | 88 | 92 | 1.04545454545455 |
| p-Tau | 4137 | 28 | 88 | 92 | 1.04545454545455 |
| tau proteins | 4137 | 28 | 88 | 92 | 1.04545454545455 |
| MAPT genetic | 4137 | 28 | 88 | 92 | 1.04545454545455 |
| H1-MAPT | 4137 | 28 | 88 | 92 | 1.04545454545455 |
| t-tau | 4137 | 28 | 88 | 92 | 1.04545454545455 |
| MAPT-N296H | 4137 | 28 | 88 | 92 | 1.04545454545455 |
| pTau | 4137 | 28 | 88 | 92 | 1.04545454545455 |
| microtubule (MT)-associated protein tau | 4137 | 28 | 88 | 92 | 1.04545454545455 |
| tau/ | 4137 | 28 | 88 | 92 | 1.04545454545455 |
| alpha-synuclein's | C0285890 | 41 | 56 | 83 | 1.48214285714286 |
| alpha-synWT | C0285890 | 41 | 56 | 83 | 1.48214285714286 |
| AS | C0285890 | 41 | 56 | 83 | 1.48214285714286 |
| alphaS | C0285890 | 41 | 56 | 83 | 1.48214285714286 |
| alpha-syn | C0285890 | 41 | 56 | 83 | 1.48214285714286 |
| alpha synuclein | C0285890 | 41 | 56 | 83 | 1.48214285714286 |
| alpha Syn | C0285890 | 41 | 56 | 83 | 1.48214285714286 |
| N-alpha-Syn | C0285890 | 41 | 56 | 83 | 1.48214285714286 |
| alpha-synucleins | C0285890 | 41 | 56 | 83 | 1.48214285714286 |
| aSyn | C0285890 | 41 | 56 | 83 | 1.48214285714286 |
| alpha-gamma-synuclein | C0285890 | 41 | 56 | 83 | 1.48214285714286 |
| alpha-synuclein | C0285890 | 41 | 56 | 83 | 1.48214285714286 |
| alphaSN | C0285890 | 41 | 56 | 83 | 1.48214285714286 |
| alpha- synuclein | C0285890 | 41 | 56 | 83 | 1.48214285714286 |
| alfa-synuclein | C0285890 | 41 | 56 | 83 | 1.48214285714286 |
| alpha-S | C0285890 | 41 | 56 | 83 | 1.48214285714286 |
| alpha- > beta-synuclein | C0285890 | 41 | 56 | 83 | 1.48214285714286 |
| A53T-SNCA | C0285890 | 41 | 56 | 83 | 1.48214285714286 |
| palphaSyn | C0285890 | 41 | 56 | 83 | 1.48214285714286 |
| a-syn | C0285890 | 41 | 56 | 83 | 1.48214285714286 |
| aSN | C0285890 | 41 | 56 | 83 | 1.48214285714286 |
| h-alphas | C0285890 | 41 | 56 | 83 | 1.48214285714286 |
| ASOX | C0285890 | 41 | 56 | 83 | 1.48214285714286 |
| A53T AS cells | C0285890 | 41 | 56 | 83 | 1.48214285714286 |
| alpha-synP | C0285890 | 41 | 56 | 83 | 1.48214285714286 |
| SNCA-OVX | C0285890 | 41 | 56 | 83 | 1.48214285714286 |
| alpha-SN | C0285890 | 41 | 56 | 83 | 1.48214285714286 |
| alpha)-synuclein | C0285890 | 41 | 56 | 83 | 1.48214285714286 |
| dopamine transporter | C0114838 | 49 | 43 | 81 | 1.88372093023256 |
| dopamine transporters | C0114838 | 49 | 43 | 81 | 1.88372093023256 |
| DAT | C0114838 | 49 | 43 | 81 | 1.88372093023256 |
| DA transporters | C0114838 | 49 | 43 | 81 | 1.88372093023256 |
| DA transporter | C0114838 | 49 | 43 | 81 | 1.88372093023256 |
| dopamine (DA) transporter | C0114838 | 49 | 43 | 81 | 1.88372093023256 |
| dopamine-transporter | C0114838 | 49 | 43 | 81 | 1.88372093023256 |
| dopamine active transporter | C0114838 | 49 | 43 | 81 | 1.88372093023256 |
| dopamine reuptake transporter | C0114838 | 49 | 43 | 81 | 1.88372093023256 |
| DAT-SPECT | C0114838 | 49 | 43 | 81 | 1.88372093023256 |
| DATs | C0114838 | 49 | 43 | 81 | 1.88372093023256 |
| DAT SPECT | C0114838 | 49 | 43 | 81 | 1.88372093023256 |
| DAT | 6531 | 24 | 43 | 75 | 1.74418604651163 |
| dopamine transporter | 6531 | 24 | 43 | 75 | 1.74418604651163 |
| DA transporter | 6531 | 24 | 43 | 75 | 1.74418604651163 |
| SLC6A3 | 6531 | 24 | 43 | 75 | 1.74418604651163 |
| hDAT | 6531 | 24 | 43 | 75 | 1.74418604651163 |
| DAT1 | 6531 | 24 | 43 | 75 | 1.74418604651163 |
| DAT-SPECT | 6531 | 24 | 43 | 75 | 1.74418604651163 |
| TRODAT-1 | 6531 | 24 | 43 | 75 | 1.74418604651163 |
| DAT-KO | 6531 | 24 | 43 | 75 | 1.74418604651163 |
| dopamine-transporter | 6531 | 24 | 43 | 75 | 1.74418604651163 |
| amyloid | C0002716 | 29 | 53 | 72 | 1.35849056603774 |
| amyloids | C0002716 | 29 | 53 | 72 | 1.35849056603774 |
| amyloid ( | C0002716 | 29 | 53 | 72 | 1.35849056603774 |
| Abeta42 | C0002716 | 29 | 53 | 72 | 1.35849056603774 |
| amyloid- | C0002716 | 29 | 53 | 72 | 1.35849056603774 |
| AA | C0002716 | 29 | 53 | 72 | 1.35849056603774 |
| amyloid-beta1 | C0002716 | 29 | 53 | 72 | 1.35849056603774 |
| ABSMs | C0002716 | 29 | 53 | 72 | 1.35849056603774 |
| Amyloid beta1 | C0002716 | 29 | 53 | 72 | 1.35849056603774 |
| PINK1 | 65018 | 37 | 59 | 61 | 1.03389830508475 |
| PARK6 | 65018 | 37 | 59 | 61 | 1.03389830508475 |
| PINK 1 | 65018 | 37 | 59 | 61 | 1.03389830508475 |
| PTEN-induced kinase 1 | 65018 | 37 | 59 | 61 | 1.03389830508475 |
| PTEN-induced putative kinase 1 | 65018 | 37 | 59 | 61 | 1.03389830508475 |
| PINK1 genes | 65018 | 37 | 59 | 61 | 1.03389830508475 |
| PINK1 gene | 65018 | 37 | 59 | 61 | 1.03389830508475 |
| PINK | 65018 | 37 | 59 | 61 | 1.03389830508475 |
| G309D-PINK1 | 65018 | 37 | 59 | 61 | 1.03389830508475 |
| PINK-1 | 65018 | 37 | 59 | 61 | 1.03389830508475 |
| hPINK1 | 65018 | 37 | 59 | 61 | 1.03389830508475 |
| PINK1-PARKIN | 65018 | 37 | 59 | 61 | 1.03389830508475 |
| PTEN-induced putative kinase1 | 65018 | 37 | 59 | 61 | 1.03389830508475 |
| PINK1 p | 65018 | 37 | 59 | 61 | 1.03389830508475 |
| pink1Y431 | 65018 | 37 | 59 | 61 | 1.03389830508475 |
| PINK1-PARK2 | 65018 | 37 | 59 | 61 | 1.03389830508475 |
| PINK1's | 65018 | 37 | 59 | 61 | 1.03389830508475 |
| PINK)1 | 65018 | 37 | 59 | 61 | 1.03389830508475 |
| TcPINK1 | 65018 | 37 | 59 | 61 | 1.03389830508475 |
| glucocerebrosidase | 2629 | 7 | 32 | 52 | 1.625 |
| GBA | 2629 | 7 | 32 | 52 | 1.625 |
| beta-glucocerebrosidase | 2629 | 7 | 32 | 52 | 1.625 |
| imiglucerase | 2629 | 7 | 32 | 52 | 1.625 |
| GC | 2629 | 7 | 32 | 52 | 1.625 |
| GCase | 2629 | 7 | 32 | 52 | 1.625 |
| GBA1 | 2629 | 7 | 32 | 52 | 1.625 |
| GBA gene | 2629 | 7 | 32 | 52 | 1.625 |
| GBA-SYT11 | 2629 | 7 | 32 | 52 | 1.625 |
| GBA-GBAP1 | 2629 | 7 | 32 | 52 | 1.625 |
| lysosomal glucocerebrosidase | 2629 | 7 | 32 | 52 | 1.625 |
| GBA genes | 2629 | 7 | 32 | 52 | 1.625 |
| N370S-GBA1 | 2629 | 7 | 32 | 52 | 1.625 |
| GBA1-PD | 2629 | 7 | 32 | 52 | 1.625 |
| GBA-PD | 2629 | 7 | 32 | 52 | 1.625 |
| acid-beta-glucosidase | 2629 | 7 | 32 | 52 | 1.625 |
| ubiquitin | C0041538 | 41 | 55 | 43 | 0.781818181818182 |
| Ubiquitin- | C0041538 | 41 | 55 | 43 | 0.781818181818182 |
| UPS | C0041538 | 41 | 55 | 43 | 0.781818181818182 |
| ubiquitinated | C0041538 | 41 | 55 | 43 | 0.781818181818182 |
| ubiquitin/ | C0041538 | 41 | 55 | 43 | 0.781818181818182 |
| Ub | C0041538 | 41 | 55 | 43 | 0.781818181818182 |
| UP | C0041538 | 41 | 55 | 43 | 0.781818181818182 |
| Akt | 207 | 13 | 37 | 43 | 1.16216216216216 |
| AKT1 gene | 207 | 13 | 37 | 43 | 1.16216216216216 |
| AKT1 | 207 | 13 | 37 | 43 | 1.16216216216216 |
| Rac | 207 | 13 | 37 | 43 | 1.16216216216216 |
| PKB | 207 | 13 | 37 | 43 | 1.16216216216216 |
| pAkt | 207 | 13 | 37 | 43 | 1.16216216216216 |
| ubiquitin's | C0041538 | 41 | 55 | 43 | 0.781818181818182 |
| pAkt473 | 207 | 13 | 37 | 43 | 1.16216216216216 |
| p-Akt | 207 | 13 | 37 | 43 | 1.16216216216216 |
| ubiquitin (Ub)- | C0041538 | 41 | 55 | 43 | 0.781818181818182 |
| pUb | C0041538 | 41 | 55 | 43 | 0.781818181818182 |
| DJ-1 | 11315 | 30 | 53 | 40 | 0.754716981132076 |
| PARK7 | 11315 | 30 | 53 | 40 | 0.754716981132076 |
| DJ1 | 11315 | 30 | 53 | 40 | 0.754716981132076 |
| protein DJ-1 | 11315 | 30 | 53 | 40 | 0.754716981132076 |
| L166P DJ-1 | 11315 | 30 | 53 | 40 | 0.754716981132076 |
| E163K DJ-1 | 11315 | 30 | 53 | 40 | 0.754716981132076 |
| human DJ-1 protein | 11315 | 30 | 53 | 40 | 0.754716981132076 |
| SY5Y DJ-1 | 11315 | 30 | 53 | 40 | 0.754716981132076 |
| PARK7/DJ-1 gene | 11315 | 30 | 53 | 40 | 0.754716981132076 |
| PARK7 gene | 11315 | 30 | 53 | 40 | 0.754716981132076 |
| oncogene DJ1 | 11315 | 30 | 53 | 40 | 0.754716981132076 |
| Parkinson protein 7 | 11315 | 30 | 53 | 40 | 0.754716981132076 |
| DJ1-C57 | 11315 | 30 | 53 | 40 | 0.754716981132076 |
| 7 DJ-1 | 11315 | 30 | 53 | 40 | 0.754716981132076 |
| Parkinson disease (autosomal recessive, early onset) 7 | 11315 | 30 | 53 | 40 | 0.754716981132076 |
| TgDJ-1 | 11315 | 30 | 53 | 40 | 0.754716981132076 |
| Protein deglycase DJ-1 | 11315 | 30 | 53 | 40 | 0.754716981132076 |
| TNF-alpha | 7124 | 22 | 22 | 39 | 1.77272727272727 |
| tumor necrosis factor-alpha | 7124 | 22 | 22 | 39 | 1.77272727272727 |
| TNF | 7124 | 22 | 22 | 39 | 1.77272727272727 |
| tumor necrosis factor (TNF)-alpha | 7124 | 22 | 22 | 39 | 1.77272727272727 |
| tumor necrosis factor alpha | 7124 | 22 | 22 | 39 | 1.77272727272727 |
| TNFa | 7124 | 22 | 22 | 39 | 1.77272727272727 |
| TNFalpha | 7124 | 22 | 22 | 39 | 1.77272727272727 |
| transforming growth factor alpha | 7124 | 22 | 22 | 39 | 1.77272727272727 |
| DIF | 7124 | 22 | 22 | 39 | 1.77272727272727 |
| tumor necrosis factor | 7124 | 22 | 22 | 39 | 1.77272727272727 |
| solTNF | 7124 | 22 | 22 | 39 | 1.77272727272727 |
| tmTNF | 7124 | 22 | 22 | 39 | 1.77272727272727 |
| caspase-3 | 836 | 22 | 17 | 39 | 2.29411764705882 |
| caspase-3 gene | 836 | 22 | 17 | 39 | 2.29411764705882 |
| caspase 3 | 836 | 22 | 17 | 39 | 2.29411764705882 |
| procaspase-3 | 836 | 22 | 17 | 39 | 2.29411764705882 |
| CASP3 | 836 | 22 | 17 | 39 | 2.29411764705882 |
| TNF receptor-associated factor 6 | 7124 | 22 | 22 | 39 | 1.77272727272727 |
| Caspase3 | 836 | 22 | 17 | 39 | 2.29411764705882 |
| TNF-a | 7124 | 22 | 22 | 39 | 1.77272727272727 |
| pro-inflammatory TNF-alpha | 7124 | 22 | 22 | 39 | 1.77272727272727 |
| pro-inflammatory cytokines TNF- alpha | 7124 | 22 | 22 | 39 | 1.77272727272727 |
| TNF alpha | 7124 | 22 | 22 | 39 | 1.77272727272727 |
| FOG | 161882 | 8 | 24 | 37 | 1.54166666666667 |
| monoamine oxidase type B | 4129 | 20 | 25 | 37 | 1.48 |
| MAO-B | 4129 | 20 | 25 | 37 | 1.48 |
| MAOB | 4129 | 20 | 25 | 37 | 1.48 |
| monoamine oxidase B | 4129 | 20 | 25 | 37 | 1.48 |
| monoamine oxidase-B | 4129 | 20 | 25 | 37 | 1.48 |
| MAOB-G | 4129 | 20 | 25 | 37 | 1.48 |
| MAO B | 4129 | 20 | 25 | 37 | 1.48 |
| MAO(B) | 4129 | 20 | 25 | 37 | 1.48 |
| hMAO-B | 4129 | 20 | 25 | 37 | 1.48 |
| MAO-B gene | 4129 | 20 | 25 | 37 | 1.48 |
| nFOG | 161882 | 8 | 24 | 37 | 1.54166666666667 |
| amyloid-beta | C3484390 | 12 | 25 | 36 | 1.44 |
| amyloid beta | C3484390 | 12 | 25 | 36 | 1.44 |
| Abeta | C3484390 | 12 | 25 | 36 | 1.44 |
| amyloid beta- | C3484390 | 12 | 25 | 36 | 1.44 |
| hIAPP-induced beta- | C3484390 | 12 | 25 | 36 | 1.44 |
| Abeta42 | C3484390 | 12 | 25 | 36 | 1.44 |
| amyloid peptides beta | C3484390 | 12 | 25 | 36 | 1.44 |
| nuclear factor-kappaB | C0079904 | 11 | 25 | 35 | 1.4 |
| NF-kappaB | C0079904 | 11 | 25 | 35 | 1.4 |
| nuclear factor-kappa B | C0079904 | 11 | 25 | 35 | 1.4 |
| Nuclear factor kappa-B | C0079904 | 11 | 25 | 35 | 1.4 |
| nuclear factor kappa B | C0079904 | 11 | 25 | 35 | 1.4 |
| MoCA | 1795 | 3 | 7 | 35 | 5 |
| nuclear factor (NF)-kappaB | C0079904 | 11 | 25 | 35 | 1.4 |
| PBP | 1795 | 3 | 7 | 35 | 5 |
| NFkB | C0079904 | 11 | 25 | 35 | 1.4 |
| NF-kB | C0079904 | 11 | 25 | 35 | 1.4 |
| nuclear factor kappaB | C0079904 | 11 | 25 | 35 | 1.4 |
| nuclear transcription factor kappa B ( | C0079904 | 11 | 25 | 35 | 1.4 |
| nuclear factor NF-kappa B | C0079904 | 11 | 25 | 35 | 1.4 |
| MoCA's | 1795 | 3 | 7 | 35 | 5 |
| nuclear factor- kappa B | C0079904 | 11 | 25 | 35 | 1.4 |
| nuclear factor kappa beta | C0079904 | 11 | 25 | 35 | 1.4 |
| transcription factor NF-kB | C0079904 | 11 | 25 | 35 | 1.4 |
| tyrosine hydroxylase | C0041491 | 37 | 28 | 34 | 1.21428571428571 |
| TH | C0041491 | 37 | 28 | 34 | 1.21428571428571 |
| TH-ir | C0041491 | 37 | 28 | 34 | 1.21428571428571 |
| tyrosine-hydroxylase | C0041491 | 37 | 28 | 34 | 1.21428571428571 |
| tyrosine-3-monooxygenase | C0041491 | 37 | 28 | 34 | 1.21428571428571 |
| top | 7064 | 9 | 17 | 34 | 2 |
| tyrosine 3-monooxygenase | C0041491 | 37 | 28 | 34 | 1.21428571428571 |
| k-TSP | 7064 | 9 | 17 | 34 | 2 |
| thyrosine hydroxylase | C0041491 | 37 | 28 | 34 | 1.21428571428571 |
| LEDD | C0023570 | 11 | 21 | 33 | 1.57142857142857 |
| LC | C0023570 | 11 | 21 | 33 | 1.57142857142857 |
| LD | C0023570 | 11 | 21 | 33 | 1.57142857142857 |
| LDID | C0023570 | 11 | 21 | 33 | 1.57142857142857 |
| LD/DDI | C0023570 | 11 | 21 | 33 | 1.57142857142857 |
| L: -dopa | C0023570 | 11 | 21 | 33 | 1.57142857142857 |
| DLI | C0023570 | 11 | 21 | 33 | 1.57142857142857 |
| levodopa-equivalent | C0023570 | 11 | 21 | 33 | 1.57142857142857 |
| L-dopa preparations | C0023570 | 11 | 21 | 33 | 1.57142857142857 |
| FDOPA | C0023570 | 11 | 21 | 33 | 1.57142857142857 |
| LDOPA | C0023570 | 11 | 21 | 33 | 1.57142857142857 |
| LC-100 | C0023570 | 11 | 21 | 33 | 1.57142857142857 |
| LED | C0023570 | 11 | 21 | 33 | 1.57142857142857 |
| L -DOPA | C0023570 | 11 | 21 | 33 | 1.57142857142857 |
| L DOPA | C0023570 | 11 | 21 | 33 | 1.57142857142857 |
| F-DOPA | C0023570 | 11 | 21 | 33 | 1.57142857142857 |
| levodopa- | C0023570 | 11 | 21 | 33 | 1.57142857142857 |
| LCIIG | C0023570 | 11 | 21 | 33 | 1.57142857142857 |
| LDD | C0023570 | 11 | 21 | 33 | 1.57142857142857 |
| LEDDs | C0023570 | 11 | 21 | 33 | 1.57142857142857 |
| Levodopa's | C0023570 | 11 | 21 | 33 | 1.57142857142857 |
| LR | C0023570 | 11 | 21 | 33 | 1.57142857142857 |
| glial fibrillary acidic protein | C0017626 | 18 | 17 | 32 | 1.88235294117647 |
| glial fibrillary acid protein | C0017626 | 18 | 17 | 32 | 1.88235294117647 |
| glial fibrillary acidic proteins | C0017626 | 18 | 17 | 32 | 1.88235294117647 |
| glial fibrillar acidic protein | C0017626 | 18 | 17 | 32 | 1.88235294117647 |
| GFAP | C0017626 | 18 | 17 | 32 | 1.88235294117647 |
| Amyloid-beta (Abeta) peptide | 351 | 17 | 24 | 30 | 1.25 |
| Abeta | 351 | 17 | 24 | 30 | 1.25 |
| amyloid beta peptide | 351 | 17 | 24 | 30 | 1.25 |
| amyloid-beta peptide | 351 | 17 | 24 | 30 | 1.25 |
| amyloid- | 351 | 17 | 24 | 30 | 1.25 |
| beta-amyloid peptide | 351 | 17 | 24 | 30 | 1.25 |
| APP gene | 351 | 17 | 24 | 30 | 1.25 |
| amyloid | 351 | 17 | 24 | 30 | 1.25 |
| amyloid beta-peptide | 351 | 17 | 24 | 30 | 1.25 |
| amyloid precursor protein | 351 | 17 | 24 | 30 | 1.25 |
| STN- | 1917 | 12 | 22 | 30 | 1.36363636363636 |
| STN's | 1917 | 12 | 22 | 30 | 1.36363636363636 |
| STN ( | 1917 | 12 | 22 | 30 | 1.36363636363636 |
| APP | 351 | 17 | 24 | 30 | 1.25 |
| amyloid beta (Abeta) peptide | 351 | 17 | 24 | 30 | 1.25 |
| Abeta- | 351 | 17 | 24 | 30 | 1.25 |
| AAA | 351 | 17 | 24 | 30 | 1.25 |
| Abeta( | 351 | 17 | 24 | 30 | 1.25 |
| amyloid beta (Abeta42) peptide | 351 | 17 | 24 | 30 | 1.25 |
| ABPP | 351 | 17 | 24 | 30 | 1.25 |
| AD-specific amyloid precursor protein | 351 | 17 | 24 | 30 | 1.25 |
| Abeta peptide | 351 | 17 | 24 | 30 | 1.25 |
| STN-) | 1917 | 12 | 22 | 30 | 1.36363636363636 |
| DBS-STN | 1917 | 12 | 22 | 30 | 1.36363636363636 |
| IL-1beta | 3553 | 9 | 14 | 29 | 2.07142857142857 |
| interleukin-1beta | 3553 | 9 | 14 | 29 | 2.07142857142857 |
| IL-1 | 3553 | 9 | 14 | 29 | 2.07142857142857 |
| dopamine receptor | C0034798 | 28 | 32 | 29 | 0.90625 |
| dopamine receptors | C0034798 | 28 | 32 | 29 | 0.90625 |
| dopamine (DA) receptors | C0034798 | 28 | 32 | 29 | 0.90625 |
| DA receptors | C0034798 | 28 | 32 | 29 | 0.90625 |
| dopamine D2High receptors | C0034798 | 28 | 32 | 29 | 0.90625 |
| DAR | C0034798 | 28 | 32 | 29 | 0.90625 |
| dopaminergic D2 receptors | C0034798 | 28 | 32 | 29 | 0.90625 |
| dopaminergic receptors | C0034798 | 28 | 32 | 29 | 0.90625 |
| Dopamine receptor interacting proteins | C0034798 | 28 | 32 | 29 | 0.90625 |
| DRIPs | C0034798 | 28 | 32 | 29 | 0.90625 |
| DA receptor | C0034798 | 28 | 32 | 29 | 0.90625 |
| theta | 55879 | 8 | 17 | 29 | 1.70588235294118 |
| prion proteins | C0033164 | 9 | 24 | 29 | 1.20833333333333 |
| prion | C0033164 | 9 | 24 | 29 | 1.20833333333333 |
| prions | C0033164 | 9 | 24 | 29 | 1.20833333333333 |
| prion protein | C0033164 | 9 | 24 | 29 | 1.20833333333333 |
| cTBS | 55879 | 8 | 17 | 29 | 1.70588235294118 |
| iTBS | 55879 | 8 | 17 | 29 | 1.70588235294118 |
| DR | C0034798 | 28 | 32 | 29 | 0.90625 |
| DAT striatal specific binding sites | C0034798 | 28 | 32 | 29 | 0.90625 |
| Theta- | 55879 | 8 | 17 | 29 | 1.70588235294118 |
| IL1B | 3553 | 9 | 14 | 29 | 2.07142857142857 |
| interleukin 1beta | 3553 | 9 | 14 | 29 | 2.07142857142857 |
| D2R | C0034798 | 28 | 32 | 29 | 0.90625 |
| theta ( | 55879 | 8 | 17 | 29 | 1.70588235294118 |
| theta] | 55879 | 8 | 17 | 29 | 1.70588235294118 |
| dopamine receptor family | C0034798 | 28 | 32 | 29 | 0.90625 |
| interleukin-1-beta | 3553 | 9 | 14 | 29 | 2.07142857142857 |
| TBS | 55879 | 8 | 17 | 29 | 1.70588235294118 |
| PrPSc | C0033164 | 9 | 24 | 29 | 1.20833333333333 |
| PrPC | C0033164 | 9 | 24 | 29 | 1.20833333333333 |
| dopamine type 2 receptor | C0034798 | 28 | 32 | 29 | 0.90625 |
| interleukin-1 beta | 3553 | 9 | 14 | 29 | 2.07142857142857 |
| dopamine 1-like receptor | C0034798 | 28 | 32 | 29 | 0.90625 |
| dopamine 3-like receptor | C0034798 | 28 | 32 | 29 | 0.90625 |
| IL-1 beta | 3553 | 9 | 14 | 29 | 2.07142857142857 |
| dopamine 1 receptors | C0034798 | 28 | 32 | 29 | 0.90625 |
| IL-1b | 3553 | 9 | 14 | 29 | 2.07142857142857 |
| dopaminergic D3 receptors | C0034798 | 28 | 32 | 29 | 0.90625 |
| pro-IL-1beta | 3553 | 9 | 14 | 29 | 2.07142857142857 |
| Dopamine D2L receptor-interacting proteins | C0034798 | 28 | 32 | 29 | 0.90625 |
| alphaSYN | 20617 | 13 | 23 | 27 | 1.17391304347826 |
| alpha-syn | 20617 | 13 | 23 | 27 | 1.17391304347826 |
| SNCA | 20617 | 13 | 23 | 27 | 1.17391304347826 |
| iNOS | 4843 | 10 | 21 | 27 | 1.28571428571429 |
| inducible nitric oxide synthase | 4843 | 10 | 21 | 27 | 1.28571428571429 |
| inducible NO synthase | 4843 | 10 | 21 | 27 | 1.28571428571429 |
| NOS | 4843 | 10 | 21 | 27 | 1.28571428571429 |
| NOS2A | 4843 | 10 | 21 | 27 | 1.28571428571429 |
| inducible nitric-oxide synthase | 4843 | 10 | 21 | 27 | 1.28571428571429 |
| inducible nitric oxide synthases | 4843 | 10 | 21 | 27 | 1.28571428571429 |
| Nos2 | 4843 | 10 | 21 | 27 | 1.28571428571429 |
| inducible nitric oxide (NO) synthase | 4843 | 10 | 21 | 27 | 1.28571428571429 |
| D2 receptors | C0058698 | 23 | 11 | 26 | 2.36363636363636 |
| dopamine D2 receptors | C0058698 | 23 | 11 | 26 | 2.36363636363636 |
| D2-dopamine receptors | C0058698 | 23 | 11 | 26 | 2.36363636363636 |
| dopamine D2/D3 receptors | C0058698 | 23 | 11 | 26 | 2.36363636363636 |
| dopamine D2 receptor | C0058698 | 23 | 11 | 26 | 2.36363636363636 |
| D2R | C0058698 | 23 | 11 | 26 | 2.36363636363636 |
| dopamine receptor-D2 | C0058698 | 23 | 11 | 26 | 2.36363636363636 |
| D2 dopamine (DA) receptors | C0058698 | 23 | 11 | 26 | 2.36363636363636 |
| D2/D3 receptors | C0058698 | 23 | 11 | 26 | 2.36363636363636 |
| DA D2 receptors | C0058698 | 23 | 11 | 26 | 2.36363636363636 |
| DA D2/3 receptors | C0058698 | 23 | 11 | 26 | 2.36363636363636 |
| Da-d2r | C0058698 | 23 | 11 | 26 | 2.36363636363636 |
| dopamine-D2/3 receptor | C0058698 | 23 | 11 | 26 | 2.36363636363636 |
| dopamine/D2 receptor | C0058698 | 23 | 11 | 26 | 2.36363636363636 |
| D2 subfamily receptors | C0058698 | 23 | 11 | 26 | 2.36363636363636 |
| D2 Rs | C0058698 | 23 | 11 | 26 | 2.36363636363636 |
| dopamine D2/3 receptors | C0058698 | 23 | 11 | 26 | 2.36363636363636 |
| dopamine (DA) D2/D3 receptors | C0058698 | 23 | 11 | 26 | 2.36363636363636 |
| D2-like dopamine receptors | C0058698 | 23 | 11 | 26 | 2.36363636363636 |
| DA-D2 receptor | C0058698 | 23 | 11 | 26 | 2.36363636363636 |
| D2-receptors | C0058698 | 23 | 11 | 26 | 2.36363636363636 |
| GSH | C0017817 | 17 | 31 | 25 | 0.806451612903226 |
| GSH) | C0017817 | 17 | 31 | 25 | 0.806451612903226 |
| leucine-rich repeat kinase 2 | 66725 | 3 | 27 | 25 | 0.925925925925926 |
| LRRK2 | 66725 | 3 | 27 | 25 | 0.925925925925926 |
| Leucine-rich-repeat-kinase 2 | 66725 | 3 | 27 | 25 | 0.925925925925926 |
| leucine rich repeat kinase 2 | 66725 | 3 | 27 | 25 | 0.925925925925926 |
| GSH- | C0017817 | 17 | 31 | 25 | 0.806451612903226 |
| leucine-rich-repeat-kinase-2 | 66725 | 3 | 27 | 25 | 0.925925925925926 |
| in | C0017817 | 17 | 31 | 25 | 0.806451612903226 |
| GPx | C0017817 | 17 | 31 | 25 | 0.806451612903226 |
| sHSP | C0033684 | 23 | 33 | 24 | 0.727272727272727 |
| Proteinaceous | C0033684 | 23 | 33 | 24 | 0.727272727272727 |
| gene products | C0033684 | 23 | 33 | 24 | 0.727272727272727 |
| sHsps | C0033684 | 23 | 33 | 24 | 0.727272727272727 |
| PAF | C0033684 | 23 | 33 | 24 | 0.727272727272727 |
| gene product | C0033684 | 23 | 33 | 24 | 0.727272727272727 |
| PMs | C0033684 | 23 | 33 | 24 | 0.727272727272727 |
| PKC | C0033684 | 23 | 33 | 24 | 0.727272727272727 |
| protein's | C0033684 | 23 | 33 | 24 | 0.727272727272727 |
| GDNF | 2668 | 19 | 20 | 24 | 1.2 |
| glial cell-derived neurotrophic factor | 2668 | 19 | 20 | 24 | 1.2 |
| Glial-derived neurotrophic factor | 2668 | 19 | 20 | 24 | 1.2 |
| glial-derived neurotrophic factor gene | 2668 | 19 | 20 | 24 | 1.2 |
| GDNF gene | 2668 | 19 | 20 | 24 | 1.2 |
| GDNF plasmid DNA gene | 2668 | 19 | 20 | 24 | 1.2 |
| Glial derived neurotrophic factor | 2668 | 19 | 20 | 24 | 1.2 |
| GDNF' | 2668 | 19 | 20 | 24 | 1.2 |
| gdnf(-/- | 2668 | 19 | 20 | 24 | 1.2 |
| APOE | 348 | 15 | 14 | 24 | 1.71428571428571 |
| apolipoprotein E gene | 348 | 15 | 14 | 24 | 1.71428571428571 |
| apolipoprotein E (APOE) epsilon2 | 348 | 15 | 14 | 24 | 1.71428571428571 |
| apolipoprotein E | 348 | 15 | 14 | 24 | 1.71428571428571 |
| apolipoprotein E (APOE) 3 | 348 | 15 | 14 | 24 | 1.71428571428571 |
| APOE 4 | 348 | 15 | 14 | 24 | 1.71428571428571 |
| APOE 4 allele | 348 | 15 | 14 | 24 | 1.71428571428571 |
| Apolipoprotein E4 | 348 | 15 | 14 | 24 | 1.71428571428571 |
| APOE- | 348 | 15 | 14 | 24 | 1.71428571428571 |
| amyloid fibrils | C1449651 | 11 | 25 | 24 | 0.96 |
| amyloid-like fibrillar | C1449651 | 11 | 25 | 24 | 0.96 |
| Abeta fibrils | C1449651 | 11 | 25 | 24 | 0.96 |
| Synuclein | C0165073 | 13 | 21 | 24 | 1.14285714285714 |
| SIAH | C0165073 | 13 | 21 | 24 | 1.14285714285714 |
| synucleins | C0165073 | 13 | 21 | 24 | 1.14285714285714 |
| SYN | C0165073 | 13 | 21 | 24 | 1.14285714285714 |
| LC3 | 84557 | 1 | 12 | 24 | 2 |
| ERK1 | 5595 | 4 | 10 | 24 | 2.4 |
| Bax | 581 | 8 | 15 | 24 | 1.6 |
| Bax protein | 581 | 8 | 15 | 24 | 1.6 |
| amyloid-like fibrils | C1449651 | 11 | 25 | 24 | 0.96 |
| amyloid fibrillar | C1449651 | 11 | 25 | 24 | 0.96 |
| GDNFs | 2668 | 19 | 20 | 24 | 1.2 |
| protein- | C0033684 | 23 | 33 | 24 | 0.727272727272727 |
| LC3-II | 84557 | 1 | 12 | 24 | 2 |
| Gdnf(+/-) | 2668 | 19 | 20 | 24 | 1.2 |
| Gdnf(+/- | 2668 | 19 | 20 | 24 | 1.2 |
| amyloid-beta fibrils | C1449651 | 11 | 25 | 24 | 0.96 |
| apoE receptors | 348 | 15 | 14 | 24 | 1.71428571428571 |
| LC3-I/II | 84557 | 1 | 12 | 24 | 2 |
| APOE epsilon4 allele | 348 | 15 | 14 | 24 | 1.71428571428571 |
| protein ( | C0033684 | 23 | 33 | 24 | 0.727272727272727 |
| APOE epsilon4 | 348 | 15 | 14 | 24 | 1.71428571428571 |
| APOE epsilon2 | 348 | 15 | 14 | 24 | 1.71428571428571 |
| DLP1 | C0033684 | 23 | 33 | 24 | 0.727272727272727 |
| Glia-Derived Neurotrophic Factor | 2668 | 19 | 20 | 24 | 1.2 |
| SREBP | C0033684 | 23 | 33 | 24 | 0.727272727272727 |
| APO E | 348 | 15 | 14 | 24 | 1.71428571428571 |
| APO E4 | 348 | 15 | 14 | 24 | 1.71428571428571 |
| EYFP | C0033684 | 23 | 33 | 24 | 0.727272727272727 |
| GDNF) | 2668 | 19 | 20 | 24 | 1.2 |
| apoE2 | 348 | 15 | 14 | 24 | 1.71428571428571 |
| apoE3 | 348 | 15 | 14 | 24 | 1.71428571428571 |
| apoE4 | 348 | 15 | 14 | 24 | 1.71428571428571 |
| UCPs | C0033684 | 23 | 33 | 24 | 0.727272727272727 |
| RAFs | C1449651 | 11 | 25 | 24 | 0.96 |
| SNAPP | C0033684 | 23 | 33 | 24 | 0.727272727272727 |
| apolipoprotein E (APOE) epsilon4 | 348 | 15 | 14 | 24 | 1.71428571428571 |
| MCPs | C0033684 | 23 | 33 | 24 | 0.727272727272727 |
| PMCA | C0033684 | 23 | 33 | 24 | 0.727272727272727 |
| LC3- | 84557 | 1 | 12 | 24 | 2 |
| YAP | C0033684 | 23 | 33 | 24 | 0.727272727272727 |
| prions | C0033684 | 23 | 33 | 24 | 0.727272727272727 |
| glial-cell derived neurotrophic factor (hGDNF) gene | 2668 | 19 | 20 | 24 | 1.2 |
| hGDNF | 2668 | 19 | 20 | 24 | 1.2 |
| Synuclein's | C0165073 | 13 | 21 | 24 | 1.14285714285714 |
| AAV9-GDNF | 2668 | 19 | 20 | 24 | 1.2 |
| APOE4 allele | 348 | 15 | 14 | 24 | 1.71428571428571 |
| PQC | C0033684 | 23 | 33 | 24 | 0.727272727272727 |
| PDIs | C0033684 | 23 | 33 | 24 | 0.727272727272727 |
| LC3-I to LC3- | 84557 | 1 | 12 | 24 | 2 |
| LC-3 | 84557 | 1 | 12 | 24 | 2 |
| alphaS | C0165073 | 13 | 21 | 24 | 1.14285714285714 |
| AMPK | C0033684 | 23 | 33 | 24 | 0.727272727272727 |
| MAPK3 | 5595 | 4 | 10 | 24 | 2.4 |
| GFP-LC3 | 84557 | 1 | 12 | 24 | 2 |
| autophagy-related proteins LC3- | 84557 | 1 | 12 | 24 | 2 |
| LC3II | 84557 | 1 | 12 | 24 | 2 |
| LC3I | 84557 | 1 | 12 | 24 | 2 |
| Rare | C1514917 | 25 | 28 | 23 | 0.821428571428571 |
| brain-derived neurotrophic factor | 627 | 11 | 15 | 23 | 1.53333333333333 |
| BDNF | 627 | 11 | 15 | 23 | 1.53333333333333 |
| BDNF gene | 627 | 11 | 15 | 23 | 1.53333333333333 |
| BDNF' | 627 | 11 | 15 | 23 | 1.53333333333333 |
| brain derived neurotrophic factor | 627 | 11 | 15 | 23 | 1.53333333333333 |
| superoxide dismutase | C0038838 | 8 | 15 | 23 | 1.53333333333333 |
| superoxide-dismutase | C0038838 | 8 | 15 | 23 | 1.53333333333333 |
| SOD | C0038838 | 8 | 15 | 23 | 1.53333333333333 |
| super oxide dismutase | C0038838 | 8 | 15 | 23 | 1.53333333333333 |
| Brain-derived neurotrophic factor (BDNF) genetic | 627 | 11 | 15 | 23 | 1.53333333333333 |
| rarer | C1514917 | 25 | 28 | 23 | 0.821428571428571 |
| proBDNF | 627 | 11 | 15 | 23 | 1.53333333333333 |
| rarity | C1514917 | 25 | 28 | 23 | 0.821428571428571 |
| superoxide dismutases | C0038838 | 8 | 15 | 23 | 1.53333333333333 |
| wise | 25928 | 1 | 8 | 22 | 2.75 |
| Bcl-2 | 596 | 8 | 8 | 22 | 2.75 |
| Bcl-2 gene | 596 | 8 | 8 | 22 | 2.75 |
| bcl2 protein | 596 | 8 | 8 | 22 | 2.75 |
| BCL2 | 596 | 8 | 8 | 22 | 2.75 |
| mTOR | 21977 | 2 | 15 | 22 | 1.46666666666667 |
| tumor necrosis factor alpha | 21926 | 5 | 15 | 22 | 1.46666666666667 |
| TNF-alpha | 21926 | 5 | 15 | 22 | 1.46666666666667 |
| tumor necrosis factor-alpha | 21926 | 5 | 15 | 22 | 1.46666666666667 |
| tumor necrosis factor | 21926 | 5 | 15 | 22 | 1.46666666666667 |
| TNF | 21926 | 5 | 15 | 22 | 1.46666666666667 |
| interleukin 6 | 3569 | 4 | 11 | 22 | 2 |
| IL-6 | 3569 | 4 | 11 | 22 | 2 |
| interleukin-6 | 3569 | 4 | 11 | 22 | 2 |
| IL6 | 3569 | 4 | 11 | 22 | 2 |
| Bcl-2/Bax gene | 596 | 8 | 8 | 22 | 2.75 |
| TNFalpha | 21926 | 5 | 15 | 22 | 1.46666666666667 |
| HSF | 3569 | 4 | 11 | 22 | 2 |
| Bcl-2 genes | 596 | 8 | 8 | 22 | 2.75 |
| TOR | 21977 | 2 | 15 | 22 | 1.46666666666667 |
| Bcl2associated | 596 | 8 | 8 | 22 | 2.75 |
| lysine | C0024337 | 6 | 6 | 21 | 3.5 |
| lysines | C0024337 | 6 | 6 | 21 | 3.5 |
| green fluorescent protein | C0120285 | 14 | 25 | 21 | 0.84 |
| GFP | C0120285 | 14 | 25 | 21 | 0.84 |
| GFP(+ | C0120285 | 14 | 25 | 21 | 0.84 |
| GFP(- | C0120285 | 14 | 25 | 21 | 0.84 |
| tumor necrosis factor-alpha | 24835 | 6 | 12 | 21 | 1.75 |
| TNF | 24835 | 6 | 12 | 21 | 1.75 |
| TNF-alpha | 24835 | 6 | 12 | 21 | 1.75 |
| TNF]-alpha | 24835 | 6 | 12 | 21 | 1.75 |
| Lys | C0024337 | 6 | 6 | 21 | 3.5 |
| tumor necrosis factor alpha | 24835 | 6 | 12 | 21 | 1.75 |
| green fluorescence protein | C0120285 | 14 | 25 | 21 | 0.84 |
| TNFalpha | 24835 | 6 | 12 | 21 | 1.75 |
| green fluorescent proteins | C0120285 | 14 | 25 | 21 | 0.84 |
| GFP-IRES | C0120285 | 14 | 25 | 21 | 0.84 |
| mtPAGFP | C0120285 | 14 | 25 | 21 | 0.84 |
| green-fluorescent protein | C0120285 | 14 | 25 | 21 | 0.84 |
| 5-GFP | C0120285 | 14 | 25 | 21 | 0.84 |
| TNF- alpha | 24835 | 6 | 12 | 21 | 1.75 |
| Tnfa | 24835 | 6 | 12 | 21 | 1.75 |
| TNF-a | 24835 | 6 | 12 | 21 | 1.75 |
| HSV-GFP | C0120285 | 14 | 25 | 21 | 0.84 |
| p38 MAP kinase | 1398 | 17 | 18 | 20 | 1.11111111111111 |
| p38 MAPK | 1398 | 17 | 18 | 20 | 1.11111111111111 |
| p38 mitogen-activated protein kinase | 1398 | 17 | 18 | 20 | 1.11111111111111 |
| p38 | 1398 | 17 | 18 | 20 | 1.11111111111111 |
| p38/MAPK | 1398 | 17 | 18 | 20 | 1.11111111111111 |
| p-P38 | 1398 | 17 | 18 | 20 | 1.11111111111111 |
| caspase-3 | 25402 | 12 | 8 | 20 | 2.5 |
| caspase 3 | 25402 | 12 | 8 | 20 | 2.5 |
| MAO-A | 4128 | 3 | 9 | 20 | 2.22222222222222 |
| MAOA | 4128 | 3 | 9 | 20 | 2.22222222222222 |
| Nrf2 | 2551 | 5 | 11 | 20 | 1.81818181818182 |
| monoamine oxidase type A | 4128 | 3 | 9 | 20 | 2.22222222222222 |
| monoamine oxidase A | 4128 | 3 | 9 | 20 | 2.22222222222222 |
| IL-1beta | 16176 |  | 12 | 20 | 1.66666666666667 |
| interleukin-1-beta | 16176 |  | 12 | 20 | 1.66666666666667 |
| p38 mitogen activated protein kinase | 1398 | 17 | 18 | 20 | 1.11111111111111 |
| interleukin-1beta | 16176 |  | 12 | 20 | 1.66666666666667 |
| p38 mitogen-activated protein (MAP) kinase | 1398 | 17 | 18 | 20 | 1.11111111111111 |
| mitogen-activated protein kinase p38 | 1398 | 17 | 18 | 20 | 1.11111111111111 |
| p38 mitogen-activated protein kinases | 1398 | 17 | 18 | 20 | 1.11111111111111 |
| p38K | 1398 | 17 | 18 | 20 | 1.11111111111111 |
| interleukin 1-beta | 16176 |  | 12 | 20 | 1.66666666666667 |
| interleukin 1beta | 16176 |  | 12 | 20 | 1.66666666666667 |
| human MAO-A | 4128 | 3 | 9 | 20 | 2.22222222222222 |
| p38 mitogenactivated protein kinase | 1398 | 17 | 18 | 20 | 1.11111111111111 |
| MAO A | 4128 | 3 | 9 | 20 | 2.22222222222222 |
| hMAO-A | 4128 | 3 | 9 | 20 | 2.22222222222222 |
| interleukin 1 beta | 16176 |  | 12 | 20 | 1.66666666666667 |
| monoamine oxidase-A | 4128 | 3 | 9 | 20 | 2.22222222222222 |
| P38/p-ERK | 1398 | 17 | 18 | 20 | 1.11111111111111 |
| Il1b | 16176 |  | 12 | 20 | 1.66666666666667 |
| NRF2-KEAP1 | 2551 | 5 | 11 | 20 | 1.81818181818182 |
| caspase3 | 25402 | 12 | 8 | 20 | 2.5 |
| GSK3beta | 2932 | 2 | 7 | 19 | 2.71428571428571 |
| Trails | 8743 | 4 | 4 | 19 | 4.75 |
| Trail | 8743 | 4 | 4 | 19 | 4.75 |
| Trail making | 8743 | 4 | 4 | 19 | 4.75 |
| GSK3B | 2932 | 2 | 7 | 19 | 2.71428571428571 |
| Glycogen synthase kinase-3beta | 2932 | 2 | 7 | 19 | 2.71428571428571 |
| GSK-3beta | 2932 | 2 | 7 | 19 | 2.71428571428571 |
| glycogen synthase kinase 3beta | 2932 | 2 | 7 | 19 | 2.71428571428571 |
| Glycogen synthase kinase-3 beta | 2932 | 2 | 7 | 19 | 2.71428571428571 |
| proteasome | C0208355 | 34 | 40 | 18 | 0.45 |
| 20S proteasome | C0208355 | 34 | 40 | 18 | 0.45 |
| proteosome | C0208355 | 34 | 40 | 18 | 0.45 |
| proteasomes | C0208355 | 34 | 40 | 18 | 0.45 |
| D2R | 1813 | 11 | 14 | 18 | 1.28571428571429 |
| dopamine D2 receptor | 1813 | 11 | 14 | 18 | 1.28571428571429 |
| DRD2 | 1813 | 11 | 14 | 18 | 1.28571428571429 |
| D2 dopamine receptor gene | 1813 | 11 | 14 | 18 | 1.28571428571429 |
| hD2R | 1813 | 11 | 14 | 18 | 1.28571428571429 |
| dopamine D(2) receptor | 1813 | 11 | 14 | 18 | 1.28571428571429 |
| dopamine D2-receptor | 1813 | 11 | 14 | 18 | 1.28571428571429 |
| human dopamine D2 receptor | 1813 | 11 | 14 | 18 | 1.28571428571429 |
| AKT | 11651 | 1 | 10 | 18 | 1.8 |
| D(2)DR | 1813 | 11 | 14 | 18 | 1.28571428571429 |
| D2Rs | 1813 | 11 | 14 | 18 | 1.28571428571429 |
| Akt1 | 11651 | 1 | 10 | 18 | 1.8 |
| A2AR-D2R | 1813 | 11 | 14 | 18 | 1.28571428571429 |
| D2R-5 | 1813 | 11 | 14 | 18 | 1.28571428571429 |
| D2R-MSN | 1813 | 11 | 14 | 18 | 1.28571428571429 |
| dopamine receptor D2 | 1813 | 11 | 14 | 18 | 1.28571428571429 |
| ERK)1/2 | 2048 | 4 | 9 | 17 | 1.88888888888889 |
| ERK | 2048 | 4 | 9 | 17 | 1.88888888888889 |
| MAPK/ERK Kinase 2 | 2048 | 4 | 9 | 17 | 1.88888888888889 |
| NMDA) receptor | C0080093 | 8 | 20 | 17 | 0.85 |
| NMDA receptors | C0080093 | 8 | 20 | 17 | 0.85 |
| NMDA) receptors | C0080093 | 8 | 20 | 17 | 0.85 |
| NMDA receptor | C0080093 | 8 | 20 | 17 | 0.85 |
| NMDA-receptors | C0080093 | 8 | 20 | 17 | 0.85 |
| NMDA glutamate receptors | C0080093 | 8 | 20 | 17 | 0.85 |
| glial cell line-derived neurotrophic factor | C0207072 | 10 | 13 | 17 | 1.30769230769231 |
| glial cell-line derived neurotrophic factor | C0207072 | 10 | 13 | 17 | 1.30769230769231 |
| monoamine oxidase | C0026454 | 7 | 12 | 17 | 1.41666666666667 |
| monoamine oxidases | C0026454 | 7 | 12 | 17 | 1.41666666666667 |
| MAOs | C0026454 | 7 | 12 | 17 | 1.41666666666667 |
| MAO | C0026454 | 7 | 12 | 17 | 1.41666666666667 |
| SYN(- | 2534 | 5 | 5 | 17 | 3.4 |
| syn | 2534 | 5 | 5 | 17 | 3.4 |
| syn's | 2534 | 5 | 5 | 17 | 3.4 |
| ERK1 | 2048 | 4 | 9 | 17 | 1.88888888888889 |
| glial cell line derived neurotrophic factor | C0207072 | 10 | 13 | 17 | 1.30769230769231 |
| NMDA-receptor | C0080093 | 8 | 20 | 17 | 0.85 |
| NMDA/AMPA receptor | C0080093 | 8 | 20 | 17 | 0.85 |
| glial-cell line derived neurotrophic factor | C0207072 | 10 | 13 | 17 | 1.30769230769231 |
| N-methyl-D-aspartate receptors | C0080093 | 8 | 20 | 17 | 0.85 |
| NMDARs | C0080093 | 8 | 20 | 17 | 0.85 |
| PFFs | 2534 | 5 | 5 | 17 | 3.4 |
| alpha-SYOs | 2534 | 5 | 5 | 17 | 3.4 |
| Fyn | 2534 | 5 | 5 | 17 | 3.4 |
| ERK 1 | 2048 | 4 | 9 | 17 | 1.88888888888889 |
| ubiquitin protein ligases | C0077678 | 8 | 21 | 16 | 0.761904761904762 |
| E3 ubiquitin-ligases | C0077678 | 8 | 21 | 16 | 0.761904761904762 |
| ubiquitin-protein ligase | C0077678 | 8 | 21 | 16 | 0.761904761904762 |
| ubiquitin-protein isopeptide ligase | C0077678 | 8 | 21 | 16 | 0.761904761904762 |
| E3 ubiquitin-ligase | C0077678 | 8 | 21 | 16 | 0.761904761904762 |
| E3 ubiquitin ligase | C0077678 | 8 | 21 | 16 | 0.761904761904762 |
| E3 ligase | C0077678 | 8 | 21 | 16 | 0.761904761904762 |
| human Haptoglobin (Hp) protein | 4929 | 11 | 20 | 16 | 0.8 |
| Nurr1 | 4929 | 11 | 20 | 16 | 0.8 |
| human protein | 4929 | 11 | 20 | 16 | 0.8 |
| Nurr 1 | 4929 | 11 | 20 | 16 | 0.8 |
| human proteins | 4929 | 11 | 20 | 16 | 0.8 |
| extracellular signal-regulated kinase | C0600388 | 9 | 7 | 16 | 2.28571428571429 |
| signal-regulated kinase | C0600388 | 9 | 7 | 16 | 2.28571428571429 |
| ERK | C0600388 | 9 | 7 | 16 | 2.28571428571429 |
| extracellular signal-regulated kinase 1/2 (ERK1/2) mitogen-activated protein kinase | C0600388 | 9 | 7 | 16 | 2.28571428571429 |
| amyloid precursor protein | C0085151 | 6 | 14 | 16 | 1.14285714285714 |
| betaAPP | C0085151 | 6 | 14 | 16 | 1.14285714285714 |
| beta-amyloid precursor protein | C0085151 | 6 | 14 | 16 | 1.14285714285714 |
| APPsw | C0085151 | 6 | 14 | 16 | 1.14285714285714 |
| amyloid-beta protein precursor | C0085151 | 6 | 14 | 16 | 1.14285714285714 |
| PI3K | 5294 | 3 | 6 | 16 | 2.66666666666667 |
| Ferritin | C0015879 | 4 | 6 | 16 | 2.66666666666667 |
| caspase-3 | 12367 | 4 | 10 | 16 | 1.6 |
| caspase 3 | 12367 | 4 | 10 | 16 | 1.6 |
| caspase3 | 12367 | 4 | 10 | 16 | 1.6 |
| Abeta precursor protein | C0085151 | 6 | 14 | 16 | 1.14285714285714 |
| PI3K-AKT | 5294 | 3 | 6 | 16 | 2.66666666666667 |
| amyloid precursor proteins | C0085151 | 6 | 14 | 16 | 1.14285714285714 |
| APP | C0085151 | 6 | 14 | 16 | 1.14285714285714 |
| nuclear receptor related-1 | 4929 | 11 | 20 | 16 | 0.8 |
| E3 ligases | C0077678 | 8 | 21 | 16 | 0.761904761904762 |
| E3-ubiquitin ligase | C0077678 | 8 | 21 | 16 | 0.761904761904762 |
| human PD-associated protein | 4929 | 11 | 20 | 16 | 0.8 |
| human gene products | 4929 | 11 | 20 | 16 | 0.8 |
| human alpha-synuclein protein | 4929 | 11 | 20 | 16 | 0.8 |
| extracellular signal-regulated kinases | C0600388 | 9 | 7 | 16 | 2.28571428571429 |
| nuclear receptor related 1 | 4929 | 11 | 20 | 16 | 0.8 |
| human brain proteins | 4929 | 11 | 20 | 16 | 0.8 |
| E3-ligase | C0077678 | 8 | 21 | 16 | 0.761904761904762 |
| human ROCO proteins | 4929 | 11 | 20 | 16 | 0.8 |
| p-ERK | C0600388 | 9 | 7 | 16 | 2.28571428571429 |
| human alpha synuclein protein | 4929 | 11 | 20 | 16 | 0.8 |
| NR4A2 | 4929 | 11 | 20 | 16 | 0.8 |
| amyloid protein precursor | C0085151 | 6 | 14 | 16 | 1.14285714285714 |
| human neuronal protein | 4929 | 11 | 20 | 16 | 0.8 |
| extracellular signal regulated kinase | C0600388 | 9 | 7 | 16 | 2.28571428571429 |
| Nuclear receptor-related 1 | 4929 | 11 | 20 | 16 | 0.8 |
| FL-APP | C0085151 | 6 | 14 | 16 | 1.14285714285714 |
| PI3K- | 5294 | 3 | 6 | 16 | 2.66666666666667 |
| TrkB-PI3K | 5294 | 3 | 6 | 16 | 2.66666666666667 |
| E3 Ubiquitin Ligases | C0077678 | 8 | 21 | 16 | 0.761904761904762 |
| CASP3 | 12367 | 4 | 10 | 16 | 1.6 |
| E3 ubiquitin-protein ligase | C0077678 | 8 | 21 | 16 | 0.761904761904762 |
| NR4A2 gene | 4929 | 11 | 20 | 16 | 0.8 |
| amyloid-beta precursor protein | C0085151 | 6 | 14 | 16 | 1.14285714285714 |
| extracellular signal-regulated protein kinase | C0600388 | 9 | 7 | 16 | 2.28571428571429 |
| extracellular signal regulated protein kinase | C0600388 | 9 | 7 | 16 | 2.28571428571429 |
| SF | C0015879 | 4 | 6 | 16 | 2.66666666666667 |
| extracellular signal-regulated protein kinases | C0600388 | 9 | 7 | 16 | 2.28571428571429 |
| Nurr-1 | 4929 | 11 | 20 | 16 | 0.8 |
| cytochrome C | 54205 | 15 | 11 | 15 | 1.36363636363636 |
| IL-1 | null | 3 | 21 | 15 | 0.714285714285714 |
| GPCR | null | 3 | 21 | 15 | 0.714285714285714 |
| ERK | null | 3 | 21 | 15 | 0.714285714285714 |
| PSP | null | 3 | 21 | 15 | 0.714285714285714 |
| luciferase | C0024075 | 5 | 9 | 15 | 1.66666666666667 |
| ALS | 3483 | 4 | 4 | 15 | 3.75 |
| DJ-1 | 57320 | 5 | 11 | 15 | 1.36363636363636 |
| interleukin-1beta | 24494 | 2 | 7 | 15 | 2.14285714285714 |
| IL-1beta | 24494 | 2 | 7 | 15 | 2.14285714285714 |
| Brain-derived neurotrophic factor | 12064 | 3 | 11 | 15 | 1.36363636363636 |
| BDNF | 12064 | 3 | 11 | 15 | 1.36363636363636 |
| brain derived neurotrophic factor | 12064 | 3 | 11 | 15 | 1.36363636363636 |
| PI3K | null | 3 | 21 | 15 | 0.714285714285714 |
| PRP | null | 3 | 21 | 15 | 0.714285714285714 |
| RAMP | null | 3 | 21 | 15 | 0.714285714285714 |
| PARK7 | 57320 | 5 | 11 | 15 | 1.36363636363636 |
| ALF | null | 3 | 21 | 15 | 0.714285714285714 |
| NDR | null | 3 | 21 | 15 | 0.714285714285714 |
| microtubule-associated protein | null | 3 | 21 | 15 | 0.714285714285714 |
| PI 3-K | null | 3 | 21 | 15 | 0.714285714285714 |
| T1 | null | 3 | 21 | 15 | 0.714285714285714 |
| GSTs | null | 3 | 21 | 15 | 0.714285714285714 |
| YAP | null | 3 | 21 | 15 | 0.714285714285714 |
| Rab7 | null | 3 | 21 | 15 | 0.714285714285714 |
| Dj1 | 57320 | 5 | 11 | 15 | 1.36363636363636 |
| cytochromec | 54205 | 15 | 11 | 15 | 1.36363636363636 |
| AMPK | null | 3 | 21 | 15 | 0.714285714285714 |
| IL-1 beta | 24494 | 2 | 7 | 15 | 2.14285714285714 |
| DAn | null | 3 | 21 | 15 | 0.714285714285714 |
| receptor- | C0597357 | 10 | 6 | 14 | 2.33333333333333 |
| receptor-modifying proteins | C0597357 | 10 | 6 | 14 | 2.33333333333333 |
| receptor protein | C0597357 | 10 | 6 | 14 | 2.33333333333333 |
| receptor (D1R)- | C0597357 | 10 | 6 | 14 | 2.33333333333333 |
| D1R | C0597357 | 10 | 6 | 14 | 2.33333333333333 |
| molecular chaperone | C0243041 | 23 | 25 | 14 | 0.56 |
| molecular chaperones | C0243041 | 23 | 25 | 14 | 0.56 |
| chaperones | C0243041 | 23 | 25 | 14 | 0.56 |
| CMA | C0243041 | 23 | 25 | 14 | 0.56 |
| chaperone- | C0243041 | 23 | 25 | 14 | 0.56 |
| proteome | C0751973 | 14 | 7 | 14 | 2 |
| HBPP | C0751973 | 14 | 7 | 14 | 2 |
| proteomes | C0751973 | 14 | 7 | 14 | 2 |
| cysteine | C0010654 | 14 | 23 | 14 | 0.608695652173913 |
| l-cysteine | C0010654 | 14 | 23 | 14 | 0.608695652173913 |
| Cys | C0010654 | 14 | 23 | 14 | 0.608695652173913 |
| C106 | C0010654 | 14 | 23 | 14 | 0.608695652173913 |
| protein kinase | C0033640 | 10 | 9 | 14 | 1.55555555555556 |
| protein kinases | C0033640 | 10 | 9 | 14 | 1.55555555555556 |
| PINK1 protein | C0033640 | 10 | 9 | 14 | 1.55555555555556 |
| Abeta | C1705543 | 9 | 18 | 14 | 0.777777777777778 |
| Abeta( | C1705543 | 9 | 18 | 14 | 0.777777777777778 |
| glutathione peroxidase | C0017822 | 5 | 11 | 14 | 1.27272727272727 |
| GFAP | 2670 | 4 | 13 | 14 | 1.07692307692308 |
| GFAP gene | 2670 | 4 | 13 | 14 | 1.07692307692308 |
| Bcl-2 | 12043 | 5 | 4 | 14 | 3.5 |
| Bcl2 | 12043 | 5 | 4 | 14 | 3.5 |
| interleukin-6 | 24498 | 1 | 3 | 14 | 4.66666666666667 |
| chaperone's | C0243041 | 23 | 25 | 14 | 0.56 |
| molecular co-chaperone | C0243041 | 23 | 25 | 14 | 0.56 |
| Beclin 1 | 8678 |  | 7 | 14 | 2 |
| cysteines | C0010654 | 14 | 23 | 14 | 0.608695652173913 |
| IL-6 | 24498 | 1 | 3 | 14 | 4.66666666666667 |
| Beclin-1 | 8678 |  | 7 | 14 | 2 |
| AMPK | C0033640 | 10 | 9 | 14 | 1.55555555555556 |
| chaperon | C0243041 | 23 | 25 | 14 | 0.56 |
| D2R | C0597357 | 10 | 6 | 14 | 2.33333333333333 |
| kinase-associated protein | C0033640 | 10 | 9 | 14 | 1.55555555555556 |
| GPx | C0017822 | 5 | 11 | 14 | 1.27272727272727 |
| chaperoning | C0243041 | 23 | 25 | 14 | 0.56 |
| beclin1 | 8678 |  | 7 | 14 | 2 |
| FcgammaR | C0597357 | 10 | 6 | 14 | 2.33333333333333 |
| ATG6 | 8678 |  | 7 | 14 | 2 |
| glial fibrillary acidic protein (GFAP) gene | 2670 | 4 | 13 | 14 | 1.07692307692308 |
| protein-coupled receptors | C0597357 | 10 | 6 | 14 | 2.33333333333333 |
| protein coupled receptors | C0597357 | 10 | 6 | 14 | 2.33333333333333 |
| GSH-Px | C0017822 | 5 | 11 | 14 | 1.27272727272727 |
| PINK1 kinase | C0033640 | 10 | 9 | 14 | 1.55555555555556 |
| molecular chaperone- | C0243041 | 23 | 25 | 14 | 0.56 |
| NLRs | C0597357 | 10 | 6 | 14 | 2.33333333333333 |
| APP | C1705543 | 9 | 18 | 14 | 0.777777777777778 |
| Il6 | 24498 | 1 | 3 | 14 | 4.66666666666667 |
| D1R- | C0597357 | 10 | 6 | 14 | 2.33333333333333 |
| BECN1 | 8678 |  | 7 | 14 | 2 |
| A1 Rs | C0597357 | 10 | 6 | 14 | 2.33333333333333 |
| A2A Rs | C0597357 | 10 | 6 | 14 | 2.33333333333333 |
| A2AR- | C0597357 | 10 | 6 | 14 | 2.33333333333333 |
| Abeta(1 | C1705543 | 9 | 18 | 14 | 0.777777777777778 |
| TrkB | C0597357 | 10 | 6 | 14 | 2.33333333333333 |
| interleukin 6 | 24498 | 1 | 3 | 14 | 4.66666666666667 |
| RAGE | C0597357 | 10 | 6 | 14 | 2.33333333333333 |
| glial fibrillary acidic protein | 2670 | 4 | 13 | 14 | 1.07692307692308 |
| A2A R | C0597357 | 10 | 6 | 14 | 2.33333333333333 |
| mitogen-activated protein (MAP) kinase | C0752312 | 5 | 10 | 13 | 1.3 |
| mitogen-activated protein kinases | C0752312 | 5 | 10 | 13 | 1.3 |
| mitogen-activated protein kinase | C0752312 | 5 | 10 | 13 | 1.3 |
| MAPK/ERK | C0752312 | 5 | 10 | 13 | 1.3 |
| p53 | 7157 | 17 | 14 | 13 | 0.928571428571429 |
| p53 protein | 7157 | 17 | 14 | 13 | 0.928571428571429 |
| COMT | 1312 | 16 | 19 | 13 | 0.684210526315789 |
| catechol O-methyltransferase (COMT) gene | 1312 | 16 | 19 | 13 | 0.684210526315789 |
| catechol O-methyltransferase | 1312 | 16 | 19 | 13 | 0.684210526315789 |
| catechol-O-methyltransferase | 1312 | 16 | 19 | 13 | 0.684210526315789 |
| COMT gene | 1312 | 16 | 19 | 13 | 0.684210526315789 |
| Adenosine A2A receptor | 135 | 14 | 5 | 13 | 2.6 |
| adenosine A(2A) receptor | 135 | 14 | 5 | 13 | 2.6 |
| ADORA2A | 135 | 14 | 5 | 13 | 2.6 |
| pace | C0287990 | 9 | 15 | 13 | 0.866666666666667 |
| paced | C0287990 | 9 | 15 | 13 | 0.866666666666667 |
| MSA | 7173 | 7 | 4 | 13 | 3.25 |
| caspase-3 | C0291573 | 2 | 6 | 13 | 2.16666666666667 |
| hub | 1993 | 1 | 1 | 13 | 13 |
| glial cell line-derived neurotrophic factor | 25453 | 5 | 6 | 13 | 2.16666666666667 |
| GDNF | 25453 | 5 | 6 | 13 | 2.16666666666667 |
| catechol-O-methyl transferase | 1312 | 16 | 19 | 13 | 0.684210526315789 |
| adenosine A 2A receptor | 135 | 14 | 5 | 13 | 2.6 |
| MAP kinases | C0752312 | 5 | 10 | 13 | 1.3 |
| adenosine receptors | 135 | 14 | 5 | 13 | 2.6 |
| Adenosine A2A-receptor | 135 | 14 | 5 | 13 | 2.6 |
| Adenosine A(2A)-receptor | 135 | 14 | 5 | 13 | 2.6 |
| mitogen-activated protein kinase 3 family | C0752312 | 5 | 10 | 13 | 1.3 |
| caspases-3 | C0291573 | 2 | 6 | 13 | 2.16666666666667 |
| Adenosine A2A receptor gene | 135 | 14 | 5 | 13 | 2.6 |
| proteins p53 | 7157 | 17 | 14 | 13 | 0.928571428571429 |
| p53 target proteins | 7157 | 17 | 14 | 13 | 0.928571428571429 |
| CC3 | C0291573 | 2 | 6 | 13 | 2.16666666666667 |
| MAPK | C0752312 | 5 | 10 | 13 | 1.3 |
| catechol-O-methyltransferase (COMT) gene | 1312 | 16 | 19 | 13 | 0.684210526315789 |
| pace- | C0287990 | 9 | 15 | 13 | 0.866666666666667 |
| mitogen activated protein kinase | C0752312 | 5 | 10 | 13 | 1.3 |
| #NAME? | C0752312 | 5 | 10 | 13 | 1.3 |
| COMT genetic | 1312 | 16 | 19 | 13 | 0.684210526315789 |
| COMT genes | 1312 | 16 | 19 | 13 | 0.684210526315789 |
| A2AR | 135 | 14 | 5 | 13 | 2.6 |
| caspase 3 | C0291573 | 2 | 6 | 13 | 2.16666666666667 |
| hubs | 1993 | 1 | 1 | 13 | 13 |
| A2AAR | 135 | 14 | 5 | 13 | 2.6 |
| TP53 | 7157 | 17 | 14 | 13 | 0.928571428571429 |
| A2ARs | 135 | 14 | 5 | 13 | 2.6 |
| glial cell-line derived neurotrophic factor | 25453 | 5 | 6 | 13 | 2.16666666666667 |
| A2AR gene | 135 | 14 | 5 | 13 | 2.6 |
| catechol Omethyltransferase | 1312 | 16 | 19 | 13 | 0.684210526315789 |
| mitogenactivated protein kinase | C0752312 | 5 | 10 | 13 | 1.3 |
| Catechol-O-methyltransferase (COMT) genetic | 1312 | 16 | 19 | 13 | 0.684210526315789 |
| p53 tumor suppressor | 7157 | 17 | 14 | 13 | 0.928571428571429 |
| Tp53 gene | 7157 | 17 | 14 | 13 | 0.928571428571429 |
| Tp53 protein | 7157 | 17 | 14 | 13 | 0.928571428571429 |
| amyloid fibril forming protein | C1456454 | 6 | 17 | 12 | 0.705882352941177 |
| amyloid protein | C1456454 | 6 | 17 | 12 | 0.705882352941177 |
| amyloidogenic proteins | C1456454 | 6 | 17 | 12 | 0.705882352941177 |
| amyloid proteins | C1456454 | 6 | 17 | 12 | 0.705882352941177 |
| amyloid forming proteins | C1456454 | 6 | 17 | 12 | 0.705882352941177 |
| ortholog | C1335144 | 8 | 10 | 12 | 1.2 |
| ortholog gene | C1335144 | 8 | 10 | 12 | 1.2 |
| orthologs | C1335144 | 8 | 10 | 12 | 1.2 |
| orthologue | C1335144 | 8 | 10 | 12 | 1.2 |
| Bcl-2 | 24224 | 3 | 12 | 12 | 1 |
| Bax | 12028 | 7 | 3 | 12 | 4 |
| BCL2-associated X protein | 12028 | 7 | 3 | 12 | 4 |
| Bax | 24887 | 2 | 10 | 12 | 1.2 |
| Akt | 24185 | 4 | 6 | 12 | 2 |
| Maps | C0026045 | 1 | 9 | 12 | 1.33333333333333 |
| cAMP response element-binding protein | 820 | 3 | 1 | 12 | 12 |
| cAMP | 820 | 3 | 1 | 12 | 12 |
| dopamine- and cAMP-regulated phosphoprotein-32 | 820 | 3 | 1 | 12 | 12 |
| GSK-3beta | 84027 | 1 | 2 | 12 | 6 |
| BDNF | 24225 | 3 | 8 | 12 | 1.5 |
| brain-derived neurotrophic factor | 24225 | 3 | 8 | 12 | 1.5 |
| disease-associated genes | 925 | 3 | 8 | 12 | 1.5 |
| disease-modifying genes | 925 | 3 | 8 | 12 | 1.5 |
| human disease proteins | 925 | 3 | 8 | 12 | 1.5 |
| Amyloidogenic metal-binding proteins | C1456454 | 6 | 17 | 12 | 0.705882352941177 |
| disease-related genes | 925 | 3 | 8 | 12 | 1.5 |
| Nrf2 | 18024 | 1 | 4 | 12 | 3 |
| NF-E2-related factor 2 | 18024 | 1 | 4 | 12 | 3 |
| disease genes | 925 | 3 | 8 | 12 | 1.5 |
| p62 | 2965 |  | 9 | 12 | 1.33333333333333 |
| amyloidogenic peptides/proteins | C1456454 | 6 | 17 | 12 | 0.705882352941177 |
| disease gene | 925 | 3 | 8 | 12 | 1.5 |
| Bcl(2 | 24224 | 3 | 12 | 12 | 1 |
| amyloid-forming protein | C1456454 | 6 | 17 | 12 | 0.705882352941177 |
| microtubule-associated tau protein | C0026045 | 1 | 9 | 12 | 1.33333333333333 |
| brain derived neurotrophic factor | 24225 | 3 | 8 | 12 | 1.5 |
| disease provoking genes | 925 | 3 | 8 | 12 | 1.5 |
| amyloid-like proteins | C1456454 | 6 | 17 | 12 | 0.705882352941177 |
| disease associated genes | 925 | 3 | 8 | 12 | 1.5 |
| amyloid-protein | C1456454 | 6 | 17 | 12 | 0.705882352941177 |
| glycogen synthase kinase-3beta | 84027 | 1 | 2 | 12 | 6 |
| microtubule-associated protein | C0026045 | 1 | 9 | 12 | 1.33333333333333 |
| CD8 | 925 | 3 | 8 | 12 | 1.5 |
| GSK3beta | 84027 | 1 | 2 | 12 | 6 |
| glycogen synthase kinase 3 beta | 84027 | 1 | 2 | 12 | 6 |
| Bcl2 | 24224 | 3 | 12 | 12 | 1 |
| microtubule associated proteins | C0026045 | 1 | 9 | 12 | 1.33333333333333 |
| amyloid peptide/protein | C1456454 | 6 | 17 | 12 | 0.705882352941177 |
| Nrf-2 | 18024 | 1 | 4 | 12 | 3 |
| disease-gene | 925 | 3 | 8 | 12 | 1.5 |
| amyloid binding proteins | C1456454 | 6 | 17 | 12 | 0.705882352941177 |
| Orthologous gene | C1335144 | 8 | 10 | 12 | 1.2 |
| amyloid-forming proteins | C1456454 | 6 | 17 | 12 | 0.705882352941177 |
| cAMP response-element binding protein | 820 | 3 | 1 | 12 | 12 |
| cAMP response element binding (CREB) protein | 820 | 3 | 1 | 12 | 12 |
| cAMP response element-binding (CREB) protein | 820 | 3 | 1 | 12 | 12 |
| glycogen synthase kinase 3beta | 84027 | 1 | 2 | 12 | 6 |
| #NAME? | 820 | 3 | 1 | 12 | 12 |
| cramp | 820 | 3 | 1 | 12 | 12 |
| cAMP response element binding protein | 820 | 3 | 1 | 12 | 12 |
| cAMP-responsive element binding protein | 820 | 3 | 1 | 12 | 12 |
| vesicular monoamine transporter 2 | C0246932 | 15 | 8 | 11 | 1.375 |
| vesicular monoamine transporter-2 | C0246932 | 15 | 8 | 11 | 1.375 |
| VMAT | C0246932 | 15 | 8 | 11 | 1.375 |
| NMDA | C0079883 | 10 | 13 | 11 | 0.846153846153846 |
| GTPase | C0018296 | 12 | 14 | 11 | 0.785714285714286 |
| GTPases | C0018296 | 12 | 14 | 11 | 0.785714285714286 |
| alpha1 | 146 | 8 | 3 | 11 | 3.66666666666667 |
| DAR | 146 | 8 | 3 | 11 | 3.66666666666667 |
| Vim | 7431 | 1 | 12 | 11 | 0.916666666666667 |
| GFAP | 14580 | 7 | 6 | 11 | 1.83333333333333 |
| interleukin-6 | 16193 | 1 | 5 | 11 | 2.2 |
| allelic | C0002085 | 2 | 8 | 11 | 1.375 |
| GSK3beta | 56637 |  | 3 | 11 | 3.66666666666667 |
| PRNP | 5621 | 2 | 5 | 11 | 2.2 |
| prion protein | 5621 | 2 | 5 | 11 | 2.2 |
| CJD | 5621 | 2 | 5 | 11 | 2.2 |
| PrP c | 5621 | 2 | 5 | 11 | 2.2 |
| PrPc | 5621 | 2 | 5 | 11 | 2.2 |
| PrP(c) | 5621 | 2 | 5 | 11 | 2.2 |
| Creutzfeldt-Jakob disease | 5621 | 2 | 5 | 11 | 2.2 |
| acetylcholinesterase | C0001044 | 2 | 7 | 11 | 1.57142857142857 |
| neurotrophins | C0027754 | 3 | 3 | 11 | 3.66666666666667 |
| NTFs | C0027754 | 3 | 3 | 11 | 3.66666666666667 |
| neural growth factors | C0027754 | 3 | 3 | 11 | 3.66666666666667 |
| PrP | 5621 | 2 | 5 | 11 | 2.2 |
| glial fibrillary acidic protein | 14580 | 7 | 6 | 11 | 1.83333333333333 |
| IL-6 | 16193 | 1 | 5 | 11 | 2.2 |
| vimentin | 7431 | 1 | 12 | 11 | 0.916666666666667 |
| PrP (C) | 5621 | 2 | 5 | 11 | 2.2 |
| glycogen synthase kinase 3beta | 56637 |  | 3 | 11 | 3.66666666666667 |
| NMDA) | C0079883 | 10 | 13 | 11 | 0.846153846153846 |
| GSK-3beta | 56637 |  | 3 | 11 | 3.66666666666667 |
| interleukin 6 | 16193 | 1 | 5 | 11 | 2.2 |
| neurotrophic proteins | C0027754 | 3 | 3 | 11 | 3.66666666666667 |
| neurotrophic protein | C0027754 | 3 | 3 | 11 | 3.66666666666667 |
| Na+/K+ -ATPase alpha1 subunit | 146 | 8 | 3 | 11 | 3.66666666666667 |
| neurotrophic factor (MANF) protein | C0027754 | 3 | 3 | 11 | 3.66666666666667 |
| alpha1 adrenergic receptor | 146 | 8 | 3 | 11 | 3.66666666666667 |
| Fatal Familial Insomnia | 5621 | 2 | 5 | 11 | 2.2 |
| alpha 1 | 146 | 8 | 3 | 11 | 3.66666666666667 |
| alpha1- | 146 | 8 | 3 | 11 | 3.66666666666667 |
| KASP | C0002085 | 2 | 8 | 11 | 1.375 |
| alpha1-adrenoceptors | 146 | 8 | 3 | 11 | 3.66666666666667 |
| glycogen synthase kinase-3beta | 56637 |  | 3 | 11 | 3.66666666666667 |
| Il6 | 16193 | 1 | 5 | 11 | 2.2 |
| MAs | C0002085 | 2 | 8 | 11 | 1.375 |
| Allele- | C0002085 | 2 | 8 | 11 | 1.375 |
| Neurotrophic growth factors | C0027754 | 3 | 3 | 11 | 3.66666666666667 |
| NTs | C0027754 | 3 | 3 | 11 | 3.66666666666667 |
| PRNP-D178N | 5621 | 2 | 5 | 11 | 2.2 |
| PRNP-E200K | 5621 | 2 | 5 | 11 | 2.2 |
| homology | C1334043 | 4 | 19 | 10 | 0.526315789473684 |
| homologs | C1334043 | 4 | 19 | 10 | 0.526315789473684 |
| homologues | C1334043 | 4 | 19 | 10 | 0.526315789473684 |
| lactate dehydrogenase | C0022917 | 9 | 5 | 10 | 2 |
| LDH | C0022917 | 9 | 5 | 10 | 2 |
| lactic acid dehydrogenase | C0022917 | 9 | 5 | 10 | 2 |
| NADPH oxidase | C0068355 | 7 | 14 | 10 | 0.714285714285714 |
| NADPH-oxidase | C0068355 | 7 | 14 | 10 | 0.714285714285714 |
| NADPH oxidases | C0068355 | 7 | 14 | 10 | 0.714285714285714 |
| ligases | C0023689 | 8 | 10 | 10 | 1 |
| ligase | C0023689 | 8 | 10 | 10 | 1 |
| binding protein | C0242210 | 7 | 1 | 10 | 10 |
| BiP | C0242210 | 7 | 1 | 10 | 10 |
| DAT | 13162 | 7 | 3 | 10 | 3.33333333333333 |
| DA transporter | 13162 | 7 | 3 | 10 | 3.33333333333333 |
| parkin | 40336 | 7 | 11 | 10 | 0.909090909090909 |
| clock | 9575 | 6 | 8 | 10 | 1.25 |
| PARP | 142 | 6 | 5 | 10 | 2 |
| poly(ADP-ribose) polymerase | 142 | 6 | 5 | 10 | 2 |
| DAT | C0596902 | 5 | 4 | 10 | 2.5 |
| membrane dopamine transporter | C0596902 | 5 | 4 | 10 | 2.5 |
| DMT1 | C0596902 | 5 | 4 | 10 | 2.5 |
| membrane transporter | C0596902 | 5 | 4 | 10 | 2.5 |
| PINK1 | 68943 | 3 | 10 | 10 | 1 |
| catalase | C0007367 | 3 | 9 | 10 | 1.11111111111111 |
| GFAP | 24387 | 1 | 5 | 10 | 2 |
| adenosine receptor | C0001471 | 3 | 7 | 10 | 1.42857142857143 |
| AR | C0001471 | 3 | 7 | 10 | 1.42857142857143 |
| adenosine receptors | C0001471 | 3 | 7 | 10 | 1.42857142857143 |
| adenosine 2A receptor | C0001471 | 3 | 7 | 10 | 1.42857142857143 |
| Fahn | 79152 | 3 | 3 | 10 | 3.33333333333333 |
| BFMDS | 79152 | 3 | 3 | 10 | 3.33333333333333 |
| AADC | 1644 | 4 | 7 | 10 | 1.42857142857143 |
| dopa decarboxylase | 1644 | 4 | 7 | 10 | 1.42857142857143 |
| hAADC | 1644 | 4 | 7 | 10 | 1.42857142857143 |
| aromatic l-amino acid decarboxylase | 1644 | 4 | 7 | 10 | 1.42857142857143 |
| Heme oxygenase-1 | 3162 | 3 | 6 | 10 | 1.66666666666667 |
| HO-1 | 3162 | 3 | 6 | 10 | 1.66666666666667 |
| HO-1 gene | 3162 | 3 | 6 | 10 | 1.66666666666667 |
| Poly(ADP-ribose) polymerase-1 | 142 | 6 | 5 | 10 | 2 |
| PARP-1 | 142 | 6 | 5 | 10 | 2 |
| nicotinamide adenine dinucleotide phosphate (NADPH) oxidase | C0068355 | 7 | 14 | 10 | 0.714285714285714 |
| CAT | C0007367 | 3 | 9 | 10 | 1.11111111111111 |
| NOX | C0068355 | 7 | 14 | 10 | 0.714285714285714 |
| GPBP | C0242210 | 7 | 1 | 10 | 10 |
| PHOX | C0068355 | 7 | 14 | 10 | 0.714285714285714 |
| VPS35 | 55737 |  | 11 | 10 | 0.909090909090909 |
| gene 'vacuolar protein sorting 35 homolog | C1334043 | 4 | 19 | 10 | 0.526315789473684 |
| vacuolar protein sorting 35 | 55737 |  | 11 | 10 | 0.909090909090909 |
| clock genes | 9575 | 6 | 8 | 10 | 1.25 |
| PARK17 | 55737 |  | 11 | 10 | 0.909090909090909 |
| VPS35 genes | 55737 |  | 11 | 10 | 0.909090909090909 |
| membrane transporters | C0596902 | 5 | 4 | 10 | 2.5 |
| VPS35 genetic | 55737 |  | 11 | 10 | 0.909090909090909 |
| EC 2.1.1.6 | C0023689 | 8 | 10 | 10 | 1 |
| poly(ADP-ribose) polymerase-1 gene | 142 | 6 | 5 | 10 | 2 |
| dParkin | 40336 | 7 | 11 | 10 | 0.909090909090909 |
| clocks | 9575 | 6 | 8 | 10 | 1.25 |
| CST | 9575 | 6 | 8 | 10 | 1.25 |
| CRT | 9575 | 6 | 8 | 10 | 1.25 |
| membrane transport proteins | C0596902 | 5 | 4 | 10 | 2.5 |
| adenosine 2A receptors | C0001471 | 3 | 7 | 10 | 1.42857142857143 |
| VPS35 gene | 55737 |  | 11 | 10 | 0.909090909090909 |
| aromatic (L)-amino acid decarboxylase | 1644 | 4 | 7 | 10 | 1.42857142857143 |
| PTEN-induced putative kinase 1 | 68943 | 3 | 10 | 10 | 1 |
| NADPH-dependent oxidases | C0068355 | 7 | 14 | 10 | 0.714285714285714 |
| poly-ADP ribose polymerase | 142 | 6 | 5 | 10 | 2 |
| poly (ADP-ribose) polymerase 1 | 142 | 6 | 5 | 10 | 2 |
| PARP1 | 142 | 6 | 5 | 10 | 2 |
| SLC6A3 | 13162 | 7 | 3 | 10 | 3.33333333333333 |
| binding proteins | C0242210 | 7 | 1 | 10 | 10 |
| adenosine) receptor | C0001471 | 3 | 7 | 10 | 1.42857142857143 |
| Poly(ADP-ribose)polymerase-1 | 142 | 6 | 5 | 10 | 2 |
| PARP-1's | 142 | 6 | 5 | 10 | 2 |
| poly (ADP-ribose) polymerase | 142 | 6 | 5 | 10 | 2 |
| BFMDRS | 79152 | 3 | 3 | 10 | 3.33333333333333 |
| binding neuronal protein | C0242210 | 7 | 1 | 10 | 10 |
| dopa-decarboxylase | 1644 | 4 | 7 | 10 | 1.42857142857143 |
| VPS35-DLP1 | 55737 |  | 11 | 10 | 0.909090909090909 |
| heme oxygenase 1 | 3162 | 3 | 6 | 10 | 1.66666666666667 |
| Ddc | 1644 | 4 | 7 | 10 | 1.42857142857143 |
| HMOX1 | 3162 | 3 | 6 | 10 | 1.66666666666667 |
| AMT | C0596902 | 5 | 4 | 10 | 2.5 |
| ENAH | C1334043 | 4 | 19 | 10 | 0.526315789473684 |
| Vacuolar protein sorting-associated protein 35 | 55737 |  | 11 | 10 | 0.909090909090909 |
| FTM | 79152 | 3 | 3 | 10 | 3.33333333333333 |
| SREBP | C0242210 | 7 | 1 | 10 | 10 |
| scVPS35 | 55737 |  | 11 | 10 | 0.909090909090909 |
| binding immunoglobulin protein | C0242210 | 7 | 1 | 10 | 10 |
| clock feedback gene | 9575 | 6 | 8 | 10 | 1.25 |
| clock-targeted genes | 9575 | 6 | 8 | 10 | 1.25 |
| A2A AR | C0001471 | 3 | 7 | 10 | 1.42857142857143 |
| nicotinamide adenine dinucleotide phosphate-oxidase | C0068355 | 7 | 14 | 10 | 0.714285714285714 |
| clock- | 9575 | 6 | 8 | 10 | 1.25 |
| Poly (ADP-ribose)-polymerase1 | 142 | 6 | 5 | 10 | 2 |
| heme-oxygenase-1 | 3162 | 3 | 6 | 10 | 1.66666666666667 |
| homologies | C1334043 | 4 | 19 | 10 | 0.526315789473684 |
| membrane transport protein | C0596902 | 5 | 4 | 10 | 2.5 |
| stress signaling protein | C0018850 | 6 | 7 | 9 | 1.28571428571429 |
| stress shock proteins | C0018850 | 6 | 7 | 9 | 1.28571428571429 |
| heat shock proteins | C0018850 | 6 | 7 | 9 | 1.28571428571429 |
| HSPs | C0018850 | 6 | 7 | 9 | 1.28571428571429 |
| stress proteins | C0018850 | 6 | 7 | 9 | 1.28571428571429 |
| stress-responsive protein | C0018850 | 6 | 7 | 9 | 1.28571428571429 |
| heat shock protein | C0018850 | 6 | 7 | 9 | 1.28571428571429 |
| stress response protein | C0018850 | 6 | 7 | 9 | 1.28571428571429 |
| stress protein | C0018850 | 6 | 7 | 9 | 1.28571428571429 |
| mutant cDNA genes | C0678941 | 3 | 6 | 9 | 1.5 |
| gene variation | C0678941 | 3 | 6 | 9 | 1.5 |
| gene variants | C0678941 | 3 | 6 | 9 | 1.5 |
| mutant genes | C0678941 | 3 | 6 | 9 | 1.5 |
| htt | 3064 | 8 | 14 | 9 | 0.642857142857143 |
| huntingtin | 3064 | 8 | 14 | 9 | 0.642857142857143 |
| UCHL1 | 7345 | 16 | 13 | 9 | 0.692307692307692 |
| UCH-L1 | 7345 | 16 | 13 | 9 | 0.692307692307692 |
| ubiquitin C-terminal hydrolase L1 | 7345 | 16 | 13 | 9 | 0.692307692307692 |
| PARK5 | 7345 | 16 | 13 | 9 | 0.692307692307692 |
| gad | 7345 | 16 | 13 | 9 | 0.692307692307692 |
| ubiquitin-C-terminal hydrolase-L1 | 7345 | 16 | 13 | 9 | 0.692307692307692 |
| JNKs | 5599 | 13 | 11 | 9 | 0.818181818181818 |
| Jun N-terminal protein kinase | 5599 | 13 | 11 | 9 | 0.818181818181818 |
| JNK | 5599 | 13 | 11 | 9 | 0.818181818181818 |
| JNK1 | 5599 | 13 | 11 | 9 | 0.818181818181818 |
| N-terminal kinase | 5599 | 13 | 11 | 9 | 0.818181818181818 |
| Jun-N-terminal kinase | 5599 | 13 | 11 | 9 | 0.818181818181818 |
| Jun N-terminal kinase | 5599 | 13 | 11 | 9 | 0.818181818181818 |
| caspase | C0010656 | 11 | 8 | 9 | 1.125 |
| caspases | C0010656 | 11 | 8 | 9 | 1.125 |
| coding region | C0079941 | 9 | 19 | 9 | 0.473684210526316 |
| coding sequence | C0079941 | 9 | 19 | 9 | 0.473684210526316 |
| coding regions | C0079941 | 9 | 19 | 9 | 0.473684210526316 |
| open reading-frames | C0079941 | 9 | 19 | 9 | 0.473684210526316 |
| Tumor Necrosis Factor | C0041368 | 3 | 17 | 9 | 0.529411764705882 |
| tumor necrosis factor-alpha | C0041368 | 3 | 17 | 9 | 0.529411764705882 |
| TNFalpha inversely reduced alpha- | C0041368 | 3 | 17 | 9 | 0.529411764705882 |
| TNF | C0041368 | 3 | 17 | 9 | 0.529411764705882 |
| tumour necrosis factor-alpha | C0041368 | 3 | 17 | 9 | 0.529411764705882 |
| tumour necrosis factor | C0041368 | 3 | 17 | 9 | 0.529411764705882 |
| coenzyme Q-10 | C0077666 | 9 | 3 | 9 | 3 |
| coenzyme Q10 | C0077666 | 9 | 3 | 9 | 3 |
| CoQ10 | C0077666 | 9 | 3 | 9 | 3 |
| coenzyme Q(10 | C0077666 | 9 | 3 | 9 | 3 |
| human Spr gene | C0314604 | 8 | 14 | 9 | 0.642857142857143 |
| human aromatic l-amino acid decarboxylase gene | C0314604 | 8 | 14 | 9 | 0.642857142857143 |
| human Roco genes | C0314604 | 8 | 14 | 9 | 0.642857142857143 |
| human Parkinson disease gene | C0314604 | 8 | 14 | 9 | 0.642857142857143 |
| Gene encoding human | C0314604 | 8 | 14 | 9 | 0.642857142857143 |
| human DJ-1 gene | C0314604 | 8 | 14 | 9 | 0.642857142857143 |
| amyloid (beta-sheeted proteins | C0078939 | 7 | 7 | 9 | 1.28571428571429 |
| amyloid beta-protein | C0078939 | 7 | 7 | 9 | 1.28571428571429 |
| amyloid-beta protein | C0078939 | 7 | 7 | 9 | 1.28571428571429 |
| amyloid-beta peptide | C0078939 | 7 | 7 | 9 | 1.28571428571429 |
| beta-amyloid protein | C0078939 | 7 | 7 | 9 | 1.28571428571429 |
| amyloid beta protein | C0078939 | 7 | 7 | 9 | 1.28571428571429 |
| beta-amyloid-induced tau protein | C0078939 | 7 | 7 | 9 | 1.28571428571429 |
| amyloid-beta peptides | C0078939 | 7 | 7 | 9 | 1.28571428571429 |
| beta amyloid protein | C0078939 | 7 | 7 | 9 | 1.28571428571429 |
| monoamine transporter | 214084 | 7 | 2 | 9 | 4.5 |
| vesicular monoamine transporter-2 | 214084 | 7 | 2 | 9 | 4.5 |
| VMAT-2 | 214084 | 7 | 2 | 9 | 4.5 |
| vesicular monoamine transporter 2 | 214084 | 7 | 2 | 9 | 4.5 |
| VMAT2 | 214084 | 7 | 2 | 9 | 4.5 |
| NF-kappa B | 4790 | 2 | 11 | 9 | 0.818181818181818 |
| NF-kappaB | 4790 | 2 | 11 | 9 | 0.818181818181818 |
| beta-synuclein | C0245467 | 6 | 6 | 9 | 1.5 |
| BS | C0245467 | 6 | 6 | 9 | 1.5 |
| phosphatases | C0031678 | 4 | 10 | 9 | 0.9 |
| phosphatase | C0031678 | 4 | 10 | 9 | 0.9 |
| insulin | C0021641 | 4 | 9 | 9 | 1 |
| aromatic l-amino acid decarboxylase | C1412028 | 2 | 8 | 9 | 1.125 |
| aromatic amino acid decarboxylase | C1412028 | 2 | 8 | 9 | 1.125 |
| isoenzyme | C0022173 | 2 | 1 | 9 | 9 |
| isoenzymes | C0022173 | 2 | 1 | 9 | 9 |
| armadillo | 1499 | 1 | 3 | 9 | 3 |
| SOD1 | 6647 | 4 | 5 | 9 | 1.8 |
| Cu,Zn-superoxide dismutase | 6647 | 4 | 5 | 9 | 1.8 |
| Cu/Zn-superoxide-dismutase | 6647 | 4 | 5 | 9 | 1.8 |
| Cu/Zn-superoxide dismutase | 6647 | 4 | 5 | 9 | 1.8 |
| spin | 2969 | 4 | 4 | 9 | 2.25 |
| ESR | 2969 | 4 | 4 | 9 | 2.25 |
| transforming growth factor beta | C0040690 | 1 | 4 | 9 | 2.25 |
| TGF-beta | C0040690 | 1 | 4 | 9 | 2.25 |
| CREB | 12912 | 1 | 1 | 9 | 9 |
| Toll-like receptor (TLR)4 | 7099 | 1 | 5 | 9 | 1.8 |
| kit | C3853650 | 2 | 1 | 9 | 9 |
| alanine | C0001898 | 3 | 7 | 9 | 1.28571428571429 |
| S129A | C0001898 | 3 | 7 | 9 | 1.28571428571429 |
| amyloid beta-peptide | C0078939 | 7 | 7 | 9 | 1.28571428571429 |
| human genes | C0314604 | 8 | 14 | 9 | 0.642857142857143 |
| human gene | C0314604 | 8 | 14 | 9 | 0.642857142857143 |
| human disease genes | C0314604 | 8 | 14 | 9 | 0.642857142857143 |
| ubiquitin carboxyl-terminal hydrolase L1 | 7345 | 16 | 13 | 9 | 0.692307692307692 |
| gracile axonal dystrophy | 7345 | 16 | 13 | 9 | 0.692307692307692 |
| tumor necrosis factor (TNF)-alpha | C0041368 | 3 | 17 | 9 | 0.529411764705882 |
| UCHL1 gene | 7345 | 16 | 13 | 9 | 0.692307692307692 |
| human FBXO7 gene | C0314604 | 8 | 14 | 9 | 0.642857142857143 |
| JNK/SAP kinase | 5599 | 13 | 11 | 9 | 0.818181818181818 |
| NFkappaB | 4790 | 2 | 11 | 9 | 0.818181818181818 |
| Huntingtin gene | 3064 | 8 | 14 | 9 | 0.642857142857143 |
| isozymes | C0022173 | 2 | 1 | 9 | 9 |
| Abeta peptide | C0078939 | 7 | 7 | 9 | 1.28571428571429 |
| GCH1 | 2643 |  | 3 | 9 | 3 |
| human SLC41A1 gene | C0314604 | 8 | 14 | 9 | 0.642857142857143 |
| N-mutHtt | 3064 | 8 | 14 | 9 | 0.642857142857143 |
| nodal | 4838 |  |  | 9 |  |
| soluble insulin | C0021641 | 4 | 9 | 9 | 1 |
| ATF4 | 468 |  | 2 | 9 | 4.5 |
| open reading frame | C0079941 | 9 | 19 | 9 | 0.473684210526316 |
| uORF2 | C0079941 | 9 | 19 | 9 | 0.473684210526316 |
| TNFalpha | C0041368 | 3 | 17 | 9 | 0.529411764705882 |
| human clock genes | C0314604 | 8 | 14 | 9 | 0.642857142857143 |
| GTP-cyclohydrolase 1 | 2643 |  | 3 | 9 | 3 |
| aromatic l-amino-acid decarboxylase | C1412028 | 2 | 8 | 9 | 1.125 |
| human ATP13A2 gene | C0314604 | 8 | 14 | 9 | 0.642857142857143 |
| transforming growth factor-beta | C0040690 | 1 | 4 | 9 | 2.25 |
| beta-catenin | 1499 | 1 | 3 | 9 | 3 |
| Toll- | 7099 | 1 | 5 | 9 | 1.8 |
| ubiquitin carboxy-terminal hydrolase L1 | 7345 | 16 | 13 | 9 | 0.692307692307692 |
| GCH1 gene | 2643 |  | 3 | 9 | 3 |
| toll | 7099 | 1 | 5 | 9 | 1.8 |
| homodimer | 6647 | 4 | 5 | 9 | 1.8 |
| aromatic amino-acid decarboxylase | C1412028 | 2 | 8 | 9 | 1.125 |
| toll-like receptor 4 | 7099 | 1 | 5 | 9 | 1.8 |
| superoxide dismutase 1 | 6647 | 4 | 5 | 9 | 1.8 |
| heat-shock proteins | C0018850 | 6 | 7 | 9 | 1.28571428571429 |
| heat-shock protein | C0018850 | 6 | 7 | 9 | 1.28571428571429 |
| copper/zinc superoxide dismutase | 6647 | 4 | 5 | 9 | 1.8 |
| human ABCB1 gene | C0314604 | 8 | 14 | 9 | 0.642857142857143 |
| human brain gene | C0314604 | 8 | 14 | 9 | 0.642857142857143 |
| ubiquitin carboxyl-terminal esterase L1 | 7345 | 16 | 13 | 9 | 0.692307692307692 |
| human SNCA gene | C0314604 | 8 | 14 | 9 | 0.642857142857143 |
| TLR4 | 7099 | 1 | 5 | 9 | 1.8 |
| human MT-III gene | C0314604 | 8 | 14 | 9 | 0.642857142857143 |
| human tissue-specific genes | C0314604 | 8 | 14 | 9 | 0.642857142857143 |
| amyloid beta-peptides | C0078939 | 7 | 7 | 9 | 1.28571428571429 |
| human inflammation genes | C0314604 | 8 | 14 | 9 | 0.642857142857143 |
| genetic mutants | C0678941 | 3 | 6 | 9 | 1.5 |
| Amyloid beta peptides | C0078939 | 7 | 7 | 9 | 1.28571428571429 |
| PGP 9.5 | 7345 | 16 | 13 | 9 | 0.692307692307692 |
| amyloid beta (Abeta) peptides | C0078939 | 7 | 7 | 9 | 1.28571428571429 |
| Abeta peptides | C0078939 | 7 | 7 | 9 | 1.28571428571429 |
| beta-amyloid precuror protein | C0078939 | 7 | 7 | 9 | 1.28571428571429 |
| aromatic-amino-acid decarboxylase | C1412028 | 2 | 8 | 9 | 1.125 |
| human wild-type SNCA gene | C0314604 | 8 | 14 | 9 | 0.642857142857143 |
| betaS | C0245467 | 6 | 6 | 9 | 1.5 |
| LAAD | C1412028 | 2 | 8 | 9 | 1.125 |
| Toll-like receptor-4 | 7099 | 1 | 5 | 9 | 1.8 |
| P123H betaS | C0245467 | 6 | 6 | 9 | 1.5 |
| G93A-SOD1 | 6647 | 4 | 5 | 9 | 1.8 |
| human tropoelastin gene | C0314604 | 8 | 14 | 9 | 0.642857142857143 |
| human elastin gene | C0314604 | 8 | 14 | 9 | 0.642857142857143 |
| human PD genes | C0314604 | 8 | 14 | 9 | 0.642857142857143 |
| ATF4-dependent pro-apoptotic gene | 468 |  | 2 | 9 | 4.5 |
| genetic knockout mutants | C0678941 | 3 | 6 | 9 | 1.5 |
| mutant gene | C0678941 | 3 | 6 | 9 | 1.5 |
| Huntingtin (HTT) gene | 3064 | 8 | 14 | 9 | 0.642857142857143 |
| human Microtubule Actin Cross-linking Factor 1 gene | C0314604 | 8 | 14 | 9 | 0.642857142857143 |
| PI3k | 18708 |  |  | 9 |  |
| SLC18A2 | 214084 | 7 | 2 | 9 | 4.5 |
| dopa-responsive dystonia | 2643 |  | 3 | 9 | 3 |
| DYT5a | 2643 |  | 3 | 9 | 3 |
| tumor necrosis factor alpha | C0041368 | 3 | 17 | 9 | 0.529411764705882 |
| GTP cyclohydrolase 1 | 2643 |  | 3 | 9 | 3 |
| Gene Variant | C0678941 | 3 | 6 | 9 | 1.5 |
| beta-catenin-dependent myelin gene | 1499 | 1 | 3 | 9 | 3 |
| beta synuclein | C0245467 | 6 | 6 | 9 | 1.5 |
| stress causing protein | C0018850 | 6 | 7 | 9 | 1.28571428571429 |
| TMEM230 | 29058 |  |  | 9 |  |
| tolls | 7099 | 1 | 5 | 9 | 1.8 |
| h-bsyn | C0245467 | 6 | 6 | 9 | 1.5 |
| transforming growth factor (TGF)-beta | C0040690 | 1 | 4 | 9 | 2.25 |
| CoQ | C0077666 | 9 | 3 | 9 | 3 |
| TLR-4 | 7099 | 1 | 5 | 9 | 1.8 |
| human TAF1 gene | C0314604 | 8 | 14 | 9 | 0.642857142857143 |
| TMEM230 gene | 29058 |  |  | 9 |  |
| stress response proteins | C0018850 | 6 | 7 | 9 | 1.28571428571429 |
| Cu-Zn superoxide dismutase | 6647 | 4 | 5 | 9 | 1.8 |
| Toll Like Receptor 4 | 7099 | 1 | 5 | 9 | 1.8 |
| ASL | 2969 | 4 | 4 | 9 | 2.25 |
| transforming growth factor beta superfamily | C0040690 | 1 | 4 | 9 | 2.25 |
| activating transcription factor 4 | 468 |  | 2 | 9 | 4.5 |
| human GDNF gene | C0314604 | 8 | 14 | 9 | 0.642857142857143 |
| Toll-like-receptor-4 | 7099 | 1 | 5 | 9 | 1.8 |
| GCH | 2643 |  | 3 | 9 | 3 |
| amyloid cross-beta protein | C0078939 | 7 | 7 | 9 | 1.28571428571429 |
| tumor necrosis factors | C0041368 | 3 | 17 | 9 | 0.529411764705882 |
| GTP cyclohydrolase I | 2643 |  | 3 | 9 | 3 |
| human ataxin 3 gene | C0314604 | 8 | 14 | 9 | 0.642857142857143 |
| gene R1398H variant | C0678941 | 3 | 6 | 9 | 1.5 |
| TGFbeta | C0040690 | 1 | 4 | 9 | 2.25 |
| Ubiquitin C-terminal hydrolase-L1 | 7345 | 16 | 13 | 9 | 0.692307692307692 |
| transmembrane protein 230 | 29058 |  |  | 9 |  |
| transforming growth factor-beta (TGF-beta) superfamily | C0040690 | 1 | 4 | 9 | 2.25 |
| rhTFAM-stimulated gene | C0314604 | 8 | 14 | 9 | 0.642857142857143 |
| superoxide dismutase-1 | 6647 | 4 | 5 | 9 | 1.8 |
| ATF-4 | 468 |  | 2 | 9 | 4.5 |
| human AD genes | C0314604 | 8 | 14 | 9 | 0.642857142857143 |
| JNK)/p-P38 mitogen-activated protein kinase | 5599 | 13 | 11 | 9 | 0.818181818181818 |
| protein gene product 9.5 | 7345 | 16 | 13 | 9 | 0.692307692307692 |
| gene coding variant | C0678941 | 3 | 6 | 9 | 1.5 |
| mutant HTT | C0678941 | 3 | 6 | 9 | 1.5 |
| CREB/VMAT2 | 468 |  | 2 | 9 | 4.5 |
| HTT gene | 3064 | 8 | 14 | 9 | 0.642857142857143 |
| beta amyloid [a protein | C0078939 | 7 | 7 | 9 | 1.28571428571429 |
| beta-amyloid/PrPC | C0078939 | 7 | 7 | 9 | 1.28571428571429 |
| ATF4-CHOP | 468 |  | 2 | 9 | 4.5 |
| Mapk8 | 5599 | 13 | 11 | 9 | 0.818181818181818 |
| isozyme | C0022173 | 2 | 1 | 9 | 9 |
| Abeta proteins | C0078939 | 7 | 7 | 9 | 1.28571428571429 |
| activating transcription factor-4 | 468 |  | 2 | 9 | 4.5 |
| PGP9.5 | 7345 | 16 | 13 | 9 | 0.692307692307692 |
| human atg5 gene | C0314604 | 8 | 14 | 9 | 0.642857142857143 |
| p-JNK | 5599 | 13 | 11 | 9 | 0.818181818181818 |
| amyloid beta peptide | C0078939 | 7 | 7 | 9 | 1.28571428571429 |
| CREB1 | 12912 | 1 | 1 | 9 | 9 |
| SOD | 6647 | 4 | 5 | 9 | 1.8 |
| SOD1 protein | 6647 | 4 | 5 | 9 | 1.8 |
| SOD1 gene | 6647 | 4 | 5 | 9 | 1.8 |
| MnSOD | 6647 | 4 | 5 | 9 | 1.8 |
| CuZn superoxide dismutase | 6647 | 4 | 5 | 9 | 1.8 |
| SOD1-PRDX5 | 6647 | 4 | 5 | 9 | 1.8 |
| c-Jun-N-terminal kinase | 3725 | 14 | 11 | 8 | 0.727272727272727 |
| c-Jun-N-terminal kinases | 3725 | 14 | 11 | 8 | 0.727272727272727 |
| c-Jun | 3725 | 14 | 11 | 8 | 0.727272727272727 |
| c-Jun N-terminal kinase | 3725 | 14 | 11 | 8 | 0.727272727272727 |
| c-Jun N-terminal kinases | 3725 | 14 | 11 | 8 | 0.727272727272727 |
| NF-kappaB/p53/c-Jun | 3725 | 14 | 11 | 8 | 0.727272727272727 |
| c-JUN kinase | 3725 | 14 | 11 | 8 | 0.727272727272727 |
| c-Jun-N terminal kinase | 3725 | 14 | 11 | 8 | 0.727272727272727 |
| activator protein 1 | 3725 | 14 | 11 | 8 | 0.727272727272727 |
| Jun | 3725 | 14 | 11 | 8 | 0.727272727272727 |
| Botulinum toxin | C0006055 | 2 | 3 | 8 | 2.66666666666667 |
| BTX | C0006055 | 2 | 3 | 8 | 2.66666666666667 |
| BTX-A | C0006055 | 2 | 3 | 8 | 2.66666666666667 |
| Botulinum | C0006055 | 2 | 3 | 8 | 2.66666666666667 |
| glial cell line-derived neurotrophic factor | 14573 | 8 | 11 | 8 | 0.727272727272727 |
| GDNF | 14573 | 8 | 11 | 8 | 0.727272727272727 |
| nicotinic acetylcholine receptor | C0034830 | 9 | 13 | 8 | 0.615384615384615 |
| Nicotinic acetylcholine receptors | C0034830 | 9 | 13 | 8 | 0.615384615384615 |
| nAChRs | C0034830 | 9 | 13 | 8 | 0.615384615384615 |
| nicotinic receptors | C0034830 | 9 | 13 | 8 | 0.615384615384615 |
| nicotinic acetylcholinergic receptors | C0034830 | 9 | 13 | 8 | 0.615384615384615 |
| nicotinic acetycholine receptors | C0034830 | 9 | 13 | 8 | 0.615384615384615 |
| nicotinic receptor | C0034830 | 9 | 13 | 8 | 0.615384615384615 |
| Nestin | 10763 | 8 | 4 | 8 | 2 |
| protein complex | C1180347 | 8 | 8 | 8 | 1 |
| complex proteins | C1180347 | 8 | 8 | 8 | 1 |
| complex (TSC) gene products | C1180347 | 8 | 8 | 8 | 1 |
| protein complexes | C1180347 | 8 | 8 | 8 | 1 |
| complex protein | C1180347 | 8 | 8 | 8 | 1 |
| Pitx3 | 5309 | 6 | 12 | 8 | 0.666666666666667 |
| Pitx3 gene | 5309 | 6 | 12 | 8 | 0.666666666666667 |
| Pitx3-gene | 5309 | 6 | 12 | 8 | 0.666666666666667 |
| PTEN-induced kinase 1 | 31607 | 7 | 12 | 8 | 0.666666666666667 |
| PINK1 | 31607 | 7 | 12 | 8 | 0.666666666666667 |
| type | 6445 | 4 | 11 | 8 | 0.727272727272727 |
| Homocysteine | C0019878 | 5 | 8 | 8 | 1 |
| tHcy | C0019878 | 5 | 8 | 8 | 1 |
| Hcy | C0019878 | 5 | 8 | 8 | 1 |
| recombinant proteins | C0034861 | 6 | 6 | 8 | 1.33333333333333 |
| recombinant gene products | C0034861 | 6 | 6 | 8 | 1.33333333333333 |
| recombinant alpha-syn proteins | C0034861 | 6 | 6 | 8 | 1.33333333333333 |
| recombinant protein preparations | C0034861 | 6 | 6 | 8 | 1.33333333333333 |
| recombinant protein | C0034861 | 6 | 6 | 8 | 1.33333333333333 |
| recombinant human proteins | C0034861 | 6 | 6 | 8 | 1.33333333333333 |
| recombinant alphaS protein | C0034861 | 6 | 6 | 8 | 1.33333333333333 |
| hemoglobin | C0019046 | 3 | 3 | 8 | 2.66666666666667 |
| Smn-/- | 3925 | 4 | 9 | 8 | 0.888888888888889 |
| lag | 3925 | 4 | 9 | 8 | 0.888888888888889 |
| lags | 3925 | 4 | 9 | 8 | 0.888888888888889 |
| SMN | 3925 | 4 | 9 | 8 | 0.888888888888889 |
| protease K | C0059256 | 5 | 5 | 8 | 1.6 |
| proteinase K | C0059256 | 5 | 5 | 8 | 1.6 |
| protease-K | C0059256 | 5 | 5 | 8 | 1.6 |
| caspase 9 | 842 | 5 | 2 | 8 | 4 |
| caspase-9 | 842 | 5 | 2 | 8 | 4 |
| dopamine receptor D2 | 13489 | 4 | 8 | 8 | 1 |
| D2 receptor | 13489 | 4 | 8 | 8 | 1 |
| D(2) receptor | 13489 | 4 | 8 | 8 | 1 |
| FAB | 2187 | 4 | 8 | 8 | 1 |
| cytochrome c | C0010754 | 4 | 4 | 8 | 2 |
| calcium-permeable ion channels | C0006685 | 1 | 3 | 8 | 2.66666666666667 |
| calcium channels | C0006685 | 1 | 3 | 8 | 2.66666666666667 |
| dopamine receptor D1 | 13488 | 4 | 2 | 8 | 4 |
| D(1) receptor | 13488 | 4 | 2 | 8 | 4 |
| dopamine D(1) receptor | 13488 | 4 | 2 | 8 | 4 |
| Drd1a | 13488 | 4 | 2 | 8 | 4 |
| HO-1 | C0538674 | 3 | 5 | 8 | 1.6 |
| heme oxygenase-1 | C0538674 | 3 | 5 | 8 | 1.6 |
| Cox-2 | 4513 | 4 | 6 | 8 | 1.33333333333333 |
| TAT-PAX6 protein | 1385 | 1 | 2 | 8 | 4 |
| transactivator of transcription (TAT) protein | 1385 | 1 | 2 | 8 | 4 |
| AChE | 43 | 1 | 1 | 8 | 8 |
| IL-10 | 3586 | 4 | 4 | 8 | 2 |
| Interleukin-10 | 3586 | 4 | 4 | 8 | 2 |
| IL10 | 3586 | 4 | 4 | 8 | 2 |
| hIL-10 | 3586 | 4 | 4 | 8 | 2 |
| nuclear factor-E2-related factor-2 | C0289507 | 3 | 3 | 8 | 2.66666666666667 |
| NF-E2-related factor 2 | C0289507 | 3 | 3 | 8 | 2.66666666666667 |
| nuclear factor-E2-related factor 2 | C0289507 | 3 | 3 | 8 | 2.66666666666667 |
| nuclear factor E2-related factor 2 | C0289507 | 3 | 3 | 8 | 2.66666666666667 |
| NGF | C0027752 | 3 | 6 | 8 | 1.33333333333333 |
| nerve growth factor | C0027752 | 3 | 6 | 8 | 1.33333333333333 |
| RBD- | C1522002 | 1 | 1 | 8 | 8 |
| RBDs | C1522002 | 1 | 1 | 8 | 8 |
| c-jun NH(2)-terminal kinase 1/ | 3725 | 14 | 11 | 8 | 0.727272727272727 |
| jun NH(2)-terminal kinase | 3725 | 14 | 11 | 8 | 0.727272727272727 |
| PTEN-induced putative kinase 1 | 31607 | 7 | 12 | 8 | 0.666666666666667 |
| proNGF | C0027752 | 3 | 6 | 8 | 1.33333333333333 |
| Hb | C0019046 | 3 | 3 | 8 | 2.66666666666667 |
| haemoglobin | C0019046 | 3 | 3 | 8 | 2.66666666666667 |
| CREB | 1385 | 1 | 2 | 8 | 4 |
| 3'UTR | 2837 |  | 4 | 8 | 2 |
| botulinum toxins | C0006055 | 2 | 3 | 8 | 2.66666666666667 |
| NF-kappaB | 18033 |  | 6 | 8 | 1.33333333333333 |
| calcium release channel | C0006685 | 1 | 3 | 8 | 2.66666666666667 |
| Ca(2+) release channels | C0006685 | 1 | 3 | 8 | 2.66666666666667 |
| lag- | 3925 | 4 | 9 | 8 | 0.888888888888889 |
| 3' UTR | 2837 |  | 4 | 8 | 2 |
| NF-E2-related factor-2 | C0289507 | 3 | 3 | 8 | 2.66666666666667 |
| BoNT/A | C0006055 | 2 | 3 | 8 | 2.66666666666667 |
| RBD [pRBD](+) | C1522002 | 1 | 1 | 8 | 8 |
| pRBD(-) | C1522002 | 1 | 1 | 8 | 8 |
| pRBD(+ | C1522002 | 1 | 1 | 8 | 8 |
| pRBD | C1522002 | 1 | 1 | 8 | 8 |
| pRBD(- | C1522002 | 1 | 1 | 8 | 8 |
| CB1 receptor | 6445 | 4 | 11 | 8 | 0.727272727272727 |
| IL-10 gene | 3586 | 4 | 4 | 8 | 2 |
| dPINK1 | 31607 | 7 | 12 | 8 | 0.666666666666667 |
| exome | C3178814 |  | 5 | 8 | 1.6 |
| dopamine receptor 2 | 13489 | 4 | 8 | 8 | 1 |
| AMPK | 5562 |  | 1 | 8 | 8 |
| FAB's | 2187 | 4 | 8 | 8 | 1 |
| calcium channel | C0006685 | 1 | 3 | 8 | 2.66666666666667 |
| proteinase-K | C0059256 | 5 | 5 | 8 | 1.6 |
| recombinant prion protein | C0034861 | 6 | 6 | 8 | 1.33333333333333 |
| dopamine D2 receptor | 13489 | 4 | 8 | 8 | 1 |
| DRD2 | 13489 | 4 | 8 | 8 | 1 |
| dopamine D2-receptor | 13489 | 4 | 8 | 8 | 1 |
| Nrf2 | C0289507 | 3 | 3 | 8 | 2.66666666666667 |
| NF-E2 related factor 2 | C0289507 | 3 | 3 | 8 | 2.66666666666667 |
| nuclear factor-erythroid 2 (NF-E2) related factor | C0289507 | 3 | 3 | 8 | 2.66666666666667 |
| D1 receptor | 13488 | 4 | 2 | 8 | 4 |
| VDCCs | C0006685 | 1 | 3 | 8 | 2.66666666666667 |
| c-Jun NH(2)-terminal kinase | 3725 | 14 | 11 | 8 | 0.727272727272727 |
| NFkappaB | 18033 |  | 6 | 8 | 1.33333333333333 |
| recombinant Hc-TeTx protein | C0034861 | 6 | 6 | 8 | 1.33333333333333 |
| recombinant LRRK2 protein | C0034861 | 6 | 6 | 8 | 1.33333333333333 |
| recombinant DjDJ-1 protein | C0034861 | 6 | 6 | 8 | 1.33333333333333 |
| Cc | C0010754 | 4 | 4 | 8 | 2 |
| hemeoxygenase-1 | C0538674 | 3 | 5 | 8 | 1.6 |
| protein null-specific mitochondrial complex | C1180347 | 8 | 8 | 8 | 1 |
| BoNT | C0006055 | 2 | 3 | 8 | 2.66666666666667 |
| nicotinic acthylcholine receptors | C0034830 | 9 | 13 | 8 | 0.615384615384615 |
| neural tissue spheres in medium containing epidermal growth factor | C0027752 | 3 | 6 | 8 | 1.33333333333333 |
| neural growth factor | C0027752 | 3 | 6 | 8 | 1.33333333333333 |
| recombinant mouse KAT-2 protein | C0034861 | 6 | 6 | 8 | 1.33333333333333 |
| CHCHD2 | 51142 |  |  | 8 |  |
| BT | C0006055 | 2 | 3 | 8 | 2.66666666666667 |
| nuclear factor erythroid 2-related factor | C0289507 | 3 | 3 | 8 | 2.66666666666667 |
| CHCHD2 gene | 51142 |  |  | 8 |  |
| lagged | 3925 | 4 | 9 | 8 | 0.888888888888889 |
| exomes | C3178814 |  | 5 | 8 | 1.6 |
| calcium release channels | C0006685 | 1 | 3 | 8 | 2.66666666666667 |
| Recombinant ORM2 protein | C0034861 | 6 | 6 | 8 | 1.33333333333333 |
| complex signalling protein | C1180347 | 8 | 8 | 8 | 1 |
| dopamine D1 receptor | 13488 | 4 | 2 | 8 | 4 |
| NF kappa B | 18033 |  | 6 | 8 | 1.33333333333333 |
| D2-receptor | 13489 | 4 | 8 | 8 | 1 |
| transactivator protein | 1385 | 1 | 2 | 8 | 4 |
| tTA | 1385 | 1 | 2 | 8 | 4 |
| glial cell-line derived neurotrophic factor | 14573 | 8 | 11 | 8 | 0.727272727272727 |
| HB ( | C0019046 | 3 | 3 | 8 | 2.66666666666667 |
| COX2 | 4513 | 4 | 6 | 8 | 1.33333333333333 |
| RBD) | C1522002 | 1 | 1 | 8 | 8 |
| MAM | 6445 | 4 | 11 | 8 | 0.727272727272727 |
| recombinant alpha-syn protein | C0034861 | 6 | 6 | 8 | 1.33333333333333 |
| coiled-coil-helix-coiled-coil-helix domain containing 2 | 51142 |  |  | 8 |  |
| PD-RBD | C1522002 | 1 | 1 | 8 | 8 |
| recombinant wild-type DJ-1 protein | C0034861 | 6 | 6 | 8 | 1.33333333333333 |
| Hg | C0019046 | 3 | 3 | 8 | 2.66666666666667 |
| Calcium-activated SK channels | C0006685 | 1 | 3 | 8 | 2.66666666666667 |
| biogenesis proteins | C0034861 | 6 | 6 | 8 | 1.33333333333333 |
| haem oxygenase-1 | C0538674 | 3 | 5 | 8 | 1.6 |
| 3-'UTR | 2837 |  | 4 | 8 | 2 |
| cpRBD | C1522002 | 1 | 1 | 8 | 8 |
| RRMS | C1522002 | 1 | 1 | 8 | 8 |
| HC | C0019878 | 5 | 8 | 8 | 1 |
| recombinant PP2Ac protein | C0034861 | 6 | 6 | 8 | 1.33333333333333 |
| RBDSQ-J | C1522002 | 1 | 1 | 8 | 8 |
| PITX3-EGFP | 5309 | 6 | 12 | 8 | 0.666666666666667 |
| nicotinic acetylcholine alpha4beta2* receptors | C0034830 | 9 | 13 | 8 | 0.615384615384615 |
| 3'' UTR | 2837 |  | 4 | 8 | 2 |
| caspase9 | 842 | 5 | 2 | 8 | 4 |
| interleukin 10 | 3586 | 4 | 4 | 8 | 2 |
| D1-receptor | 13488 | 4 | 2 | 8 | 4 |
| AMPKalpha1 | 5562 |  | 1 | 8 | 8 |
| cJun | 3725 | 14 | 11 | 8 | 0.727272727272727 |
| RBD-- | C1522002 | 1 | 1 | 8 | 8 |
| pAMPK | 5562 |  | 1 | 8 | 8 |
| TH | C0020364 | 9 | 6 | 7 | 1.16666666666667 |
| hydroxylase | C0020364 | 9 | 6 | 7 | 1.16666666666667 |
| SCOPA-MS | C0039676 | 8 | 10 | 7 | 0.7 |
| ms) | C0039676 | 8 | 10 | 7 | 0.7 |
| ms (- | C0039676 | 8 | 10 | 7 | 0.7 |
| SNAPP-MS | C0039676 | 8 | 10 | 7 | 0.7 |
| ms ( | C0039676 | 8 | 10 | 7 | 0.7 |
| insulin | 3630 | 6 | 5 | 7 | 1.4 |
| Ins | 3630 | 6 | 5 | 7 | 1.4 |
| SNr | C0032405 | 6 | 9 | 7 | 0.777777777777778 |
| mammalian target of rapamycin | 2475 | 3 | 9 | 7 | 0.777777777777778 |
| MnSOD | 6648 | 5 | 1 | 7 | 7 |
| N-acetylcysteine | C0001047 | 4 | 9 | 7 | 0.777777777777778 |
| Delta FosB | 2354 | 4 | 3 | 7 | 2.33333333333333 |
| FosB | 2354 | 4 | 3 | 7 | 2.33333333333333 |
| DeltaFosB | 2354 | 4 | 3 | 7 | 2.33333333333333 |
| PGC-1alpha | 10891 | 1 | 8 | 7 | 0.875 |
| peroxisome proliferator-activated receptor-gamma co-activator 1alpha | 10891 | 1 | 8 | 7 | 0.875 |
| DA D1 receptors | C0058697 | 4 | 3 | 7 | 2.33333333333333 |
| dopamine D1 receptors | C0058697 | 4 | 3 | 7 | 2.33333333333333 |
| dopamine D1/D5 receptor | C0058697 | 4 | 3 | 7 | 2.33333333333333 |
| Toll-like receptors | C0670896 |  | 6 | 7 | 1.16666666666667 |
| glutamine | C0017797 | 3 | 6 | 7 | 1.16666666666667 |
| Gln | C0017797 | 3 | 6 | 7 | 1.16666666666667 |
| grid | 9402 | 1 | 6 | 7 | 1.16666666666667 |
| P300 | 2033 | 1 | 3 | 7 | 2.33333333333333 |
| inducible NO synthase | 24599 | 2 | 4 | 7 | 1.75 |
| iNOS | 24599 | 2 | 4 | 7 | 1.75 |
| poly (ADP-ribose) polymerase | 23038 | 1 | 2 | 7 | 3.5 |
| parkin-interacting proteins | C0673396 | 3 | 3 | 7 | 2.33333333333333 |
| Parkin (PARK2) interacting protein | C0673396 | 3 | 3 | 7 | 2.33333333333333 |
| Parkin-co-regulated gene (PACRG) product | C0673396 | 3 | 3 | 7 | 2.33333333333333 |
| transmembrane accessory proteins | C0021699 | 2 | 6 | 7 | 1.16666666666667 |
| inner membrane protein | C0021699 | 2 | 6 | 7 | 1.16666666666667 |
| GAL4 | 3960 | 1 | 1 | 7 | 7 |
| autoantibodies | C0004358 | 1 | 3 | 7 | 2.33333333333333 |
| glycine | C0017890 | 2 | 4 | 7 | 1.75 |
| Gly | C0017890 | 2 | 4 | 7 | 1.75 |
| NeuN | 52897 | 2 |  | 7 |  |
| ATPase | 1769 | 2 | 5 | 7 | 1.4 |
| ATPase gene | 1769 | 2 | 5 | 7 | 1.4 |
| E3 ubiquitin ligase Parkin | C2983548 |  | 5 | 7 | 1.4 |
| MAO B | 25750 | 3 | 3 | 7 | 2.33333333333333 |
| MAO-B | 25750 | 3 | 3 | 7 | 2.33333333333333 |
| FRAP | 2475 | 3 | 9 | 7 | 0.777777777777778 |
| hydroxylases | C0020364 | 9 | 6 | 7 | 1.16666666666667 |
| Mn SOD | 6648 | 5 | 1 | 7 | 7 |
| PGC-1 | 10891 | 1 | 8 | 7 | 0.875 |
| Peroxisome proliferator-activated receptor-gamma coactivator-1alpha | 10891 | 1 | 8 | 7 | 0.875 |
| mTOR | 2475 | 3 | 9 | 7 | 0.777777777777778 |
| toll-like receptor | C0670896 |  | 6 | 7 | 1.16666666666667 |
| glutamines | C0017797 | 3 | 6 | 7 | 1.16666666666667 |
| PERK | 9451 |  | 1 | 7 | 7 |
| interleukin-6 | C0021760 |  | 7 | 7 | 1 |
| grids | 9402 | 1 | 6 | 7 | 1.16666666666667 |
| protein kinase B | C0164786 |  | 8 | 7 | 0.875 |
| MS( | C0039676 | 8 | 10 | 7 | 0.7 |
| IL-6 | C0021760 |  | 7 | 7 | 1 |
| transmembrane protein | C0021699 | 2 | 6 | 7 | 1.16666666666667 |
| E3 ubiquitin-protein ligase parkin PD gene | C2983548 |  | 5 | 7 | 1.4 |
| N-acetyl cysteine | C0001047 | 4 | 9 | 7 | 0.777777777777778 |
| GBA | 14466 |  | 1 | 7 | 7 |
| GBA1 | 14466 |  | 1 | 7 | 7 |
| N-acetyl-cysteine | C0001047 | 4 | 9 | 7 | 0.777777777777778 |
| autoantibody | C0004358 | 1 | 3 | 7 | 2.33333333333333 |
| transmembrane proteins | C0021699 | 2 | 6 | 7 | 1.16666666666667 |
| NAC | C0001047 | 4 | 9 | 7 | 0.777777777777778 |
| acetyl-cysteine | C0001047 | 4 | 9 | 7 | 0.777777777777778 |
| PPARGC1A | 10891 | 1 | 8 | 7 | 0.875 |
| integral membrane proteins | C0021699 | 2 | 6 | 7 | 1.16666666666667 |
| SNpr | C0032405 | 6 | 9 | 7 | 0.777777777777778 |
| acetyl cysteine | C0001047 | 4 | 9 | 7 | 0.777777777777778 |
| MDs | 5048 |  |  | 7 |  |
| PGC1alpha | 10891 | 1 | 8 | 7 | 0.875 |
| MS# | C0039676 | 8 | 10 | 7 | 0.7 |
| dopamine D1 receptor | C0058697 | 4 | 3 | 7 | 2.33333333333333 |
| SOD2 | 6648 | 5 | 1 | 7 | 7 |
| TLR adaptor protein | C0670896 |  | 6 | 7 | 1.16666666666667 |
| TLR | C0670896 |  | 6 | 7 | 1.16666666666667 |
| TLRs | C0670896 |  | 6 | 7 | 1.16666666666667 |
| recombinant IL6 | C0021760 |  | 7 | 7 | 1 |
| parkin protein | C0673396 | 3 | 3 | 7 | 2.33333333333333 |
| peroxisome proliferator-activated receptor-gamma coactivator 1alpha | 10891 | 1 | 8 | 7 | 0.875 |
| glutamine- | C0017797 | 3 | 6 | 7 | 1.16666666666667 |
| poly(ADP-ribose) polymerase | 23038 | 1 | 2 | 7 | 3.5 |
| D1R | C0058697 | 4 | 3 | 7 | 2.33333333333333 |
| Parkin-associated proteins | C0673396 | 3 | 3 | 7 | 2.33333333333333 |
| transmembrane-anchored protein | C0021699 | 2 | 6 | 7 | 1.16666666666667 |
| interleukin (IL)-6 | C0021760 |  | 7 | 7 | 1 |
| N-acetyl-l-cysteine | C0001047 | 4 | 9 | 7 | 0.777777777777778 |
| PGC-1alpha's | 10891 | 1 | 8 | 7 | 0.875 |
| monoamine oxidase-B | 25750 | 3 | 3 | 7 | 2.33333333333333 |
| monoamine oxidase B | 25750 | 3 | 3 | 7 | 2.33333333333333 |
| mechanistic target of rapamycin | 2475 | 3 | 9 | 7 | 0.777777777777778 |
| PGC-1 alpha | 10891 | 1 | 8 | 7 | 0.875 |
| GAL4-UAS | 3960 | 1 | 1 | 7 | 7 |
| poly(ADP-ribose) polymerases | 23038 | 1 | 2 | 7 | 3.5 |
| ms/ | C0039676 | 8 | 10 | 7 | 0.7 |
| EP300 | 2033 | 1 | 3 | 7 | 2.33333333333333 |
| protein kinase B (p | C0164786 |  | 8 | 7 | 0.875 |
| MDS-UPDRS | 5048 |  |  | 7 |  |
| ms (+/- | C0039676 | 8 | 10 | 7 | 0.7 |
| glucocerebrosidase | 14466 |  | 1 | 7 | 7 |
| GCase | 14466 |  | 1 | 7 | 7 |
| Parkin overexpression selectively degraded Syt IV protein | C0673396 | 3 | 3 | 7 | 2.33333333333333 |
| ms (* | C0039676 | 8 | 10 | 7 | 0.7 |
| poly(ADP-ribose)polymerase | 23038 | 1 | 2 | 7 | 3.5 |
| p-ERK | 9451 |  | 1 | 7 | 7 |
| integral membrane protein | C0021699 | 2 | 6 | 7 | 1.16666666666667 |
| E3 Ub ligase PARKIN | C2983548 |  | 5 | 7 | 1.4 |
| partes | C0032405 | 6 | 9 | 7 | 0.777777777777778 |
| dopamine D1-class receptors | C0058697 | 4 | 3 | 7 | 2.33333333333333 |
| Autoimmune antibody | C0004358 | 1 | 3 | 7 | 2.33333333333333 |
| NAbs | C0004358 | 1 | 3 | 7 | 2.33333333333333 |
| Human peroxisome proliferator-activated receptor gamma coactivator-1alpha | 10891 | 1 | 8 | 7 | 0.875 |
| L-glutamine | C0017797 | 3 | 6 | 7 | 1.16666666666667 |
| parkin proteins | C0673396 | 3 | 3 | 7 | 2.33333333333333 |
| GRID-HAMD | 9402 | 1 | 6 | 7 | 1.16666666666667 |
| Parkin-interacting protein | C0673396 | 3 | 3 | 7 | 2.33333333333333 |
| N200-P300 | 2033 | 1 | 3 | 7 | 2.33333333333333 |
| PRM-MS | C0039676 | 8 | 10 | 7 | 0.7 |
| peroxisome proliferator-activated receptor gamma coactivator 1-alpha | 10891 | 1 | 8 | 7 | 0.875 |
| interleukin 6 | C0021760 |  | 7 | 7 | 1 |
| D1Rs | C0058697 | 4 | 3 | 7 | 2.33333333333333 |
| Poly (ADP-ribose) polymerase-1 | 23038 | 1 | 2 | 7 | 3.5 |
| eukaryotic translation initiation factor 2-alpha kinase 3 | 9451 |  | 1 | 7 | 7 |
| protein-serine kinase | C0072402 | 2 | 8 | 6 | 0.75 |
| serine threonine kinase | C0072402 | 2 | 8 | 6 | 0.75 |
| metabotropic glutamate receptor 5 | C0530778 | 3 | 4 | 6 | 1.5 |
| metabotropic glutamate receptor type 5 | C0530778 | 3 | 4 | 6 | 1.5 |
| mGluR type 5 | C0530778 | 3 | 4 | 6 | 1.5 |
| metabotropic glutamate receptor subtype 5 | C0530778 | 3 | 4 | 6 | 1.5 |
| Hsp70 | 3308 | 13 | 4 | 6 | 1.5 |
| COP | 114769 | 5 | 6 | 6 | 1 |
| complement | C0009498 | 2 | 5 | 6 | 1.2 |
| glycogen synthase kinase 3beta | C0244988 | 2 | 4 | 6 | 1.5 |
| glycogen synthase kinase-3 beta | C0244988 | 2 | 4 | 6 | 1.5 |
| glycogen synthase kinase-3beta | C0244988 | 2 | 4 | 6 | 1.5 |
| serotonin transporter | 6532 | 8 | 3 | 6 | 2 |
| SERT | 6532 | 8 | 3 | 6 | 2 |
| 5-HTT | 6532 | 8 | 3 | 6 | 2 |
| VMAT2 | 6571 | 8 | 11 | 6 | 0.545454545454545 |
| ETvmat2 | 6571 | 8 | 11 | 6 | 0.545454545454545 |
| vesicular monoamine transporter 2 | 6571 | 8 | 11 | 6 | 0.545454545454545 |
| genesis | 27022 | 5 | 13 | 6 | 0.461538461538462 |
| microtubule-associated protein 2 | C0024773 | 2 |  | 6 |  |
| wave | 8936 | 8 | 8 | 6 | 0.75 |
| monoamine oxidase-B | 109731 | 7 | 3 | 6 | 2 |
| MAO-B | 109731 | 7 | 3 | 6 | 2 |
| monoamine oxidase B | 109731 | 7 | 3 | 6 | 2 |
| CIs | 1154 | 7 | 11 | 6 | 0.545454545454545 |
| tubulin | C0041348 | 6 | 8 | 6 | 0.75 |
| NAc | 7504 | 6 | 6 | 6 | 1 |
| NAC32 | 7504 | 6 | 6 | 6 | 1 |
| N-acetylaspartate | C0067684 | 5 | 3 | 6 | 2 |
| NAA | C0067684 | 5 | 3 | 6 | 2 |
| NAA/Cr | C0067684 | 5 | 3 | 6 | 2 |
| N-acetyl aspartate | C0067684 | 5 | 3 | 6 | 2 |
| candidate genetic | C1332838 | 5 | 4 | 6 | 1.5 |
| candidate susceptibility genes | C1332838 | 5 | 4 | 6 | 1.5 |
| candidate reference genes | C1332838 | 5 | 4 | 6 | 1.5 |
| Nurr1 | 18227 | 4 | 8 | 6 | 0.75 |
| G-protein coupled receptors | C0682972 | 4 | 8 | 6 | 0.75 |
| GPCRs | C0682972 | 4 | 8 | 6 | 0.75 |
| G protein-coupled receptors | C0682972 | 4 | 8 | 6 | 0.75 |
| G-protein-coupled receptor | C0682972 | 4 | 8 | 6 | 0.75 |
| G protein-coupled receptor | C0682972 | 4 | 8 | 6 | 0.75 |
| G protein-coupled protease-activated receptors | C0682972 | 4 | 8 | 6 | 0.75 |
| glutamate transporter | C0061467 | 4 | 4 | 6 | 1.5 |
| glutamate transporters | C0061467 | 4 | 4 | 6 | 1.5 |
| PI 3-kinase | C0044602 | 4 | 2 | 6 | 3 |
| phosphatidyl-inositol-3-kinase | C0044602 | 4 | 2 | 6 | 3 |
| phosphatidylinositol 3-kinase | C0044602 | 4 | 2 | 6 | 3 |
| PI3K | C0044602 | 4 | 2 | 6 | 3 |
| protein kinase A | C0010531 | 4 | 6 | 6 | 1 |
| cyclic AMP dependent protein kinase | C0010531 | 4 | 6 | 6 | 1 |
| multidomain protein | C2326433 | 4 | 7 | 6 | 0.857142857142857 |
| multi-WD-domain protein | C2326433 | 4 | 7 | 6 | 0.857142857142857 |
| DAT | 24898 | 4 | 2 | 6 | 3 |
| Nurr1 | 54278 | 4 | 2 | 6 | 3 |
| Nurr 1 | 54278 | 4 | 2 | 6 | 3 |
| Task | 3777 | 1 | 6 | 6 | 1 |
| APAs | 2028 | 3 | 2 | 6 | 3 |
| APA | 2028 | 3 | 2 | 6 | 3 |
| silent information regulator 2 | 22933 |  | 6 | 6 | 1 |
| SIRT2 | 22933 |  | 6 | 6 | 1 |
| Sir2 | 22933 |  | 6 | 6 | 1 |
| muscarinic receptor | C0034826 | 3 | 3 | 6 | 2 |
| Muscarinic acetylcholine receptors | C0034826 | 3 | 3 | 6 | 2 |
| muscarinic acetylcholine receptor | C0034826 | 3 | 3 | 6 | 2 |
| LFP | 4000 | 3 | 5 | 6 | 1.2 |
| adenosine A(2A) receptor | 11540 | 1 |  | 6 |  |
| Nrf2 | 83619 | 1 | 5 | 6 | 1.2 |
| apolipoprotein (apo) AII | 114548 | 1 | 1 | 6 | 6 |
| NR2B | 2904 | 3 | 2 | 6 | 3 |
| ACE | 1636 | 3 | 2 | 6 | 3 |
| TANGO | 405 |  | 1 | 6 | 6 |
| IL-8 | 3576 | 3 | 2 | 6 | 3 |
| interleukin 8 | 3576 | 3 | 2 | 6 | 3 |
| CXCL8 | 3576 | 3 | 2 | 6 | 3 |
| 18)F]-dopa | C0244672 | 1 | 1 | 6 | 6 |
| genetic susceptibility loci | C0678933 | 2 | 4 | 6 | 1.5 |
| genetic loci | C0678933 | 2 | 4 | 6 | 1.5 |
| proapoptotic proteins | C1565115 |  | 1 | 6 | 6 |
| iNOS | 18126 | 2 | 4 | 6 | 1.5 |
| CCL2 | 20296 | 1 |  | 6 |  |
| interleukin | C0021764 | 1 | 2 | 6 | 3 |
| mitochondrial genes | C1563761 | 2 | 3 | 6 | 2 |
| mitochondrial respiratory genes | C1563761 | 2 | 3 | 6 | 2 |
| ret | 5979 | 2 | 2 | 6 | 3 |
| mitochondria-specific proteins | C0949610 | 2 | 4 | 6 | 1.5 |
| mitochondrial import proteins | C0949610 | 2 | 4 | 6 | 1.5 |
| CHOP | 1649 | 1 | 2 | 6 | 3 |
| Gadd153 | 1649 | 1 | 2 | 6 | 3 |
| cannabinoid CB(1) receptors | C0054594 | 2 | 4 | 6 | 1.5 |
| cannabinoid receptor | C0054594 | 2 | 4 | 6 | 1.5 |
| cannabinoid receptor- | C0054594 | 2 | 4 | 6 | 1.5 |
| cannabinoid receptors | C0054594 | 2 | 4 | 6 | 1.5 |
| purinergic receptor | C0034836 | 1 | 1 | 6 | 6 |
| mitochondrial ubiquitin-dependent protein | C0949610 | 2 | 4 | 6 | 1.5 |
| complement proteins | C0009498 | 2 | 5 | 6 | 1.2 |
| candidate PD-related genes | C1332838 | 5 | 4 | 6 | 1.5 |
| cis-acting element | 1154 | 7 | 11 | 6 | 0.545454545454545 |
| serine/threonine kinase | C0072402 | 2 | 8 | 6 | 0.75 |
| complements | C0009498 | 2 | 5 | 6 | 1.2 |
| angiotensin converting enzyme | 1636 | 3 | 2 | 6 | 3 |
| MCP-1 | 6347 | 1 | 2 | 6 | 3 |
| peptidyl-prolyl cis/trans isomerase | 1154 | 7 | 11 | 6 | 0.545454545454545 |
| DCP | 1636 | 3 | 2 | 6 | 3 |
| G protein-coupled oestrogen receptor | C0682972 | 4 | 8 | 6 | 0.75 |
| 18)F]DOPA | C0244672 | 1 | 1 | 6 | 6 |
| GPCR | C0682972 | 4 | 8 | 6 | 0.75 |
| Nrf-2 | 83619 | 1 | 5 | 6 | 1.2 |
| complementarity | C0009498 | 2 | 5 | 6 | 1.2 |
| spot | 387357 |  | 10 | 6 | 0.6 |
| mGlu5 | C0530778 | 3 | 4 | 6 | 1.5 |
| 5HTTLPR | 6532 | 8 | 3 | 6 | 2 |
| genetic locus | C0678933 | 2 | 4 | 6 | 1.5 |
| PARKIN protein | 50873 |  | 2 | 6 | 3 |
| oxidized glutathione | C0061516 |  | 4 | 6 | 1.5 |
| GSSG | C0061516 |  | 4 | 6 | 1.5 |
| phosphoinositide 3-kinases | C0089793 |  | 6 | 6 | 1 |
| CCL2 | 6347 | 1 | 2 | 6 | 3 |
| cis-acting elements | 1154 | 7 | 11 | 6 | 0.545454545454545 |
| cannabinoid 1 (CB1) receptor | C0054594 | 2 | 4 | 6 | 1.5 |
| T1-MPRAGE | 921 |  | 5 | 6 | 1.2 |
| phosphatidylinositiol-3-kinase | C0089793 |  | 6 | 6 | 1 |
| PI3K | C0089793 |  | 6 | 6 | 1 |
| PKA | C0010531 | 4 | 6 | 6 | 1 |
| heme oxygenase-1 | 15368 |  | 5 | 6 | 1.2 |
| Iba1 | 114737 |  | 2 | 6 | 3 |
| Muscarinic acetylcholine (ACh) receptors | C0034826 | 3 | 3 | 6 | 2 |
| muscarinic receptors | C0034826 | 3 | 3 | 6 | 2 |
| candidate-gene | C1332838 | 5 | 4 | 6 | 1.5 |
| G-protein-coupled receptors | C0682972 | 4 | 8 | 6 | 0.75 |
| GSK-3beta | C0244988 | 2 | 4 | 6 | 1.5 |
| NR4A2 | 18227 | 4 | 8 | 6 | 0.75 |
| AMP-activated protein kinase | C2350345 |  | 4 | 6 | 1.5 |
| interleukin-8 | 3576 | 3 | 2 | 6 | 3 |
| mitochondrial chaperone protein | C0949610 | 2 | 4 | 6 | 1.5 |
| 18F-FDOPA | C0244672 | 1 | 1 | 6 | 6 |
| VMAT-2 | 6571 | 8 | 11 | 6 | 0.545454545454545 |
| Mitochondrial fission proteins | C0949610 | 2 | 4 | 6 | 1.5 |
| cis-acting regulatory element | 1154 | 7 | 11 | 6 | 0.545454545454545 |
| multi-domain protein | C2326433 | 4 | 7 | 6 | 0.857142857142857 |
| RSP-T1 | 921 |  | 5 | 6 | 1.2 |
| serine-threonine kinase | C0072402 | 2 | 8 | 6 | 0.75 |
| phosphatidylinositol-3 kinase | C0044602 | 4 | 2 | 6 | 3 |
| Cannabinoid type-1 receptors | C0054594 | 2 | 4 | 6 | 1.5 |
| eQTLs | C3826857 |  | 2 | 6 | 3 |
| expression quantitative trait loci | C3826857 |  | 2 | 6 | 3 |
| eQTL | C3826857 |  | 2 | 6 | 3 |
| mitochondrial fission protein | C0949610 | 2 | 4 | 6 | 1.5 |
| sirtuin type 2 | 22933 |  | 6 | 6 | 1 |
| inducible NO synthase | 18126 | 2 | 4 | 6 | 1.5 |
| T1-T2 | 921 |  | 5 | 6 | 1.2 |
| glutathione disulfide | C0061516 |  | 4 | 6 | 1.5 |
| monocyte chemoattractant protein 1 | 6347 | 1 | 2 | 6 | 3 |
| serine-threonine protein kinase | C0072402 | 2 | 8 | 6 | 0.75 |
| spots | 387357 |  | 10 | 6 | 0.6 |
| pro-apoptotic proteins | C1565115 |  | 1 | 6 | 6 |
| NF-E2-related factor-2 | 83619 | 1 | 5 | 6 | 1.2 |
| sirtuin 2 | 22933 |  | 6 | 6 | 1 |
| LFPs | 4000 | 3 | 5 | 6 | 1.2 |
| Slc18a2 | 6571 | 8 | 11 | 6 | 0.545454545454545 |
| candidate PGC1alpha target genes | C1332838 | 5 | 4 | 6 | 1.5 |
| cis-genetic | 1154 | 7 | 11 | 6 | 0.545454545454545 |
| Ddit3 | 1649 | 1 | 2 | 6 | 3 |
| mitochondrial fission gene | C1563761 | 2 | 3 | 6 | 2 |
| mitochondrial pro-apoptotic ARTS protein | C0949610 | 2 | 4 | 6 | 1.5 |
| Nuclear receptor subfamily 4, group A, member 2 | 18227 | 4 | 8 | 6 | 0.75 |
| genetic risk loci | C0678933 | 2 | 4 | 6 | 1.5 |
| serine/threonine kinase family | C0072402 | 2 | 8 | 6 | 0.75 |
| HMOX1 | 15368 |  | 5 | 6 | 1.2 |
| HO-1 | 15368 |  | 5 | 6 | 1.2 |
| phosphoinositide 3-kinase | C0089793 |  | 6 | 6 | 1 |
| G-protein-coupled glutamate receptors | C0682972 | 4 | 8 | 6 | 0.75 |
| Task 1 | 3777 | 1 | 6 | 6 | 1 |
| CW | 8936 | 8 | 8 | 6 | 0.75 |
| multidomain proteins | C2326433 | 4 | 7 | 6 | 0.857142857142857 |
| mitochondrial DNA-polymerase gamma (POLG1) gene | C1563761 | 2 | 3 | 6 | 2 |
| Deltasir2 | 22933 |  | 6 | 6 | 1 |
| serine/threonine protein kinase | C0072402 | 2 | 8 | 6 | 0.75 |
| candidate modifying genes | C1332838 | 5 | 4 | 6 | 1.5 |
| PARK2 | 50873 |  | 2 | 6 | 3 |
| cis- | 1154 | 7 | 11 | 6 | 0.545454545454545 |
| targeting genes | C1332838 | 5 | 4 | 6 | 1.5 |
| PI 3-K | C0089793 |  | 6 | 6 | 1 |
| metabotropic glutamate (mGlu) 5 receptors | C0530778 | 3 | 4 | 6 | 1.5 |
| MWs | 114548 | 1 | 1 | 6 | 6 |
| IL-1RI | C0021764 | 1 | 2 | 6 | 3 |
| serine/threonine-protein kinase | C0072402 | 2 | 8 | 6 | 0.75 |
| interleukins | C0021764 | 1 | 2 | 6 | 3 |
| A2AR | 11540 | 1 |  | 6 |  |
| c-Abl | 25 |  |  | 6 |  |
| phosphatidylinositol-3-kinase | C0044602 | 4 | 2 | 6 | 3 |
| NLRP3 | 114548 | 1 | 1 | 6 | 6 |
| G-protein coupled receptor | C0682972 | 4 | 8 | 6 | 0.75 |
| t1/ | 921 |  | 5 | 6 | 1.2 |
| VMAT2-KO | 6571 | 8 | 11 | 6 | 0.545454545454545 |
| VMAT2-HI | 6571 | 8 | 11 | 6 | 0.545454545454545 |
| pNR2B | 2904 | 3 | 2 | 6 | 3 |
| Sirt2 gene | 22933 |  | 6 | 6 | 1 |
| ace-1 | 1636 | 3 | 2 | 6 | 3 |
| 5-HTTLPR | 6532 | 8 | 3 | 6 | 2 |
| GRIN2B | 2904 | 3 | 2 | 6 | 3 |
| Mitochondrial CHCHD-Containing Proteins | C0949610 | 2 | 4 | 6 | 1.5 |
| G protein-coupled nucleotide receptor | C0682972 | 4 | 8 | 6 | 0.75 |
| Abl | 25 |  |  | 6 |  |
| 6-fluoro-l-DOPA | C0244672 | 1 | 1 | 6 | 6 |
| DA transporter | 24898 | 4 | 2 | 6 | 3 |
| cannabinoid 1 receptors | C0054594 | 2 | 4 | 6 | 1.5 |
| G protein-coupled estrogen receptor | C0682972 | 4 | 8 | 6 | 0.75 |
| NFE2L2 | 83619 | 1 | 5 | 6 | 1.2 |
| cis-genes | 1154 | 7 | 11 | 6 | 0.545454545454545 |
| targeted gene | C1332838 | 5 | 4 | 6 | 1.5 |
| metabotropic glutamate receptor 1/5 | C0530778 | 3 | 4 | 6 | 1.5 |
| IL | C0021764 | 1 | 2 | 6 | 3 |
| mitochondrial chaperone proteins | C0949610 | 2 | 4 | 6 | 1.5 |
| mitochondrial DNA genes | C1563761 | 2 | 3 | 6 | 2 |
| Nurr-1 | 54278 | 4 | 2 | 6 | 3 |
| inducible nitric oxide synthase | 18126 | 2 | 4 | 6 | 1.5 |
| mitochondrial import machinery proteins | C0949610 | 2 | 4 | 6 | 1.5 |
| mitochondrial complex I protein | C0949610 | 2 | 4 | 6 | 1.5 |
| Abl1 | 25 |  |  | 6 |  |
| IL- | C0021764 | 1 | 2 | 6 | 3 |
| candidate PDassociated genes | C1332838 | 5 | 4 | 6 | 1.5 |
| monocyte chemotactic protein 1 | 6347 | 1 | 2 | 6 | 3 |
| cyclic AMP-dependent protein kinase | C0010531 | 4 | 6 | 6 | 1 |
| Sirtuin-2 | 22933 |  | 6 | 6 | 1 |
| multi domain protein | C2326433 | 4 | 7 | 6 | 0.857142857142857 |
| mitochondrial one-carbon cycle metabolism genes | C1563761 | 2 | 3 | 6 | 2 |
| phosphoinositide-3-kinase | C0089793 |  | 6 | 6 | 1 |
| Muscarinic acetylcholine M4 receptors | C0034826 | 3 | 3 | 6 | 2 |
| monocyte chemoattractant protein-1 | 20296 | 1 |  | 6 |  |
| MCP-1 | 20296 | 1 |  | 6 |  |
| 18 F]fluoro-L-dopa | C0244672 | 1 | 1 | 6 | 6 |
| MAO B | 109731 | 7 | 3 | 6 | 2 |
| chemokine profiling revealed early CCL2 | 6347 | 1 | 2 | 6 | 3 |
| aryl hydrocarbon receptor nuclear translocator (Arnt) genes | 405 |  | 1 | 6 | 6 |
| mitochondria-related genes | C1563761 | 2 | 3 | 6 | 2 |
| signal transducer and activator of transcription 3 | 6774 |  |  | 6 |  |
| STAT3 | 6774 |  |  | 6 |  |
| adenosine A2A receptor | 11540 | 1 |  | 6 |  |
| purinergic receptors | C0034836 | 1 | 1 | 6 | 6 |
| purinergic P2X7 receptor | C0034836 | 1 | 1 | 6 | 6 |
| expression quantitative trait locus | C3826857 |  | 2 | 6 | 3 |
| glycogen synthase kinase (GSK)-3beta | C0244988 | 2 | 4 | 6 | 1.5 |
| a/p-a- | 2028 | 3 | 2 | 6 | 3 |
| 5'-AMP-activated protein kinase | C2350345 |  | 4 | 6 | 1.5 |
| ACE' | 1636 | 3 | 2 | 6 | 3 |
| G protein-coupled melatonin receptors | C0682972 | 4 | 8 | 6 | 0.75 |
| WAVE1 | 8936 | 8 | 8 | 6 | 0.75 |
| serine/threonine protein kinases | C0072402 | 2 | 8 | 6 | 0.75 |
| like | 405 |  | 1 | 6 | 6 |
| mitochondrial DNA-encoded respiratory genes | C1563761 | 2 | 3 | 6 | 2 |
| mitochondrial dynamics-associated protein | C0949610 | 2 | 4 | 6 | 1.5 |
| monocyte chemoattractant protein-1 | 6347 | 1 | 2 | 6 | 3 |
| candidate target genes | C1332838 | 5 | 4 | 6 | 1.5 |
| serine/threonine kinases | C0072402 | 2 | 8 | 6 | 0.75 |
| CB2R | C0054594 | 2 | 4 | 6 | 1.5 |
| mitochondrial marker proteins | C0949610 | 2 | 4 | 6 | 1.5 |
| 3D-T1 | 921 |  | 5 | 6 | 1.2 |
| complementing | C0009498 | 2 | 5 | 6 | 1.2 |
| cannabinoid type 2 receptor | C0054594 | 2 | 4 | 6 | 1.5 |
| 5'-adenosine monophosphate-activated protein kinase | C2350345 |  | 4 | 6 | 1.5 |
| AMP activated protein kinase | C2350345 |  | 4 | 6 | 1.5 |
| angiotensin I converting enzyme | 1636 | 3 | 2 | 6 | 3 |
| N-acetyl-aspartate | C0067684 | 5 | 3 | 6 | 2 |
| complement control protein | C0009498 | 2 | 5 | 6 | 1.2 |
| cannabinoid type 1 receptors | C0054594 | 2 | 4 | 6 | 1.5 |
| Purinoceptors | C0034836 | 1 | 1 | 6 | 6 |
| DNA Damage Inducible Transcript 3 | 1649 | 1 | 2 | 6 | 3 |
| multidomain-containing scaffolding protein | C2326433 | 4 | 7 | 6 | 0.857142857142857 |
| candidate disease genes | C1332838 | 5 | 4 | 6 | 1.5 |
| t1) | 921 |  | 5 | 6 | 1.2 |
| candidate risk genes | C1332838 | 5 | 4 | 6 | 1.5 |
| mitochondria-localized genes | C1563761 | 2 | 3 | 6 | 2 |
| GluN2B | 2904 | 3 | 2 | 6 | 3 |
| NMDAR2B | 2904 | 3 | 2 | 6 | 3 |
| metabotropic glutamate receptors 5 | C0530778 | 3 | 4 | 6 | 1.5 |
| microtubule-associated protein-2 | C0024773 | 2 |  | 6 |  |
| VMAT2's | 6571 | 8 | 11 | 6 | 0.545454545454545 |
| PtdIns3K | C0044602 | 4 | 2 | 6 | 3 |
| targeting different genes | C1332838 | 5 | 4 | 6 | 1.5 |
| multi-domain Roco protein | C2326433 | 4 | 7 | 6 | 0.857142857142857 |
| microtubule associated proteins-2 | C0024773 | 2 |  | 6 |  |
| 18 F-Dopa | C0244672 | 1 | 1 | 6 | 6 |
| Bcr-Abl | 25 |  |  | 6 |  |
| Hsp70participated | 3308 | 13 | 4 | 6 | 1.5 |
| mitochondrial twin CX9C protein | C0949610 | 2 | 4 | 6 | 1.5 |
| MP | C0949610 | 2 | 4 | 6 | 1.5 |
| IFN-gamma | 3458 | 2 | 6 | 5 | 0.833333333333333 |
| interferon gamma | 3458 | 2 | 6 | 5 | 0.833333333333333 |
| mGluR5 | 14805 | 4 | 4 | 5 | 1.25 |
| max | 4149 | 3 | 4 | 5 | 1.25 |
| disease susceptibility gene | C0919453 | 10 | 9 | 5 | 0.555555555555556 |
| susceptibility genes | C0919453 | 10 | 9 | 5 | 0.555555555555556 |
| susceptibility gene | C0919453 | 10 | 9 | 5 | 0.555555555555556 |
| susceptibility gene's | C0919453 | 10 | 9 | 5 | 0.555555555555556 |
| PD susceptibility gene | C0919453 | 10 | 9 | 5 | 0.555555555555556 |
| HTRA2 | 27429 | 9 | 10 | 5 | 0.5 |
| serine protease HtrA2/Omi | 27429 | 9 | 10 | 5 | 0.5 |
| HtrA2/Omi | 27429 | 9 | 10 | 5 | 0.5 |
| serine protease OMI | 27429 | 9 | 10 | 5 | 0.5 |
| OMI | 27429 | 9 | 10 | 5 | 0.5 |
| serine protease HTRA2 | 27429 | 9 | 10 | 5 | 0.5 |
| HtrA2 gene | 27429 | 9 | 10 | 5 | 0.5 |
| polyglutamine | C0384782 | 10 | 16 | 5 | 0.3125 |
| poly-glutamine | C0384782 | 10 | 16 | 5 | 0.3125 |
| polyQ | C0384782 | 10 | 16 | 5 | 0.3125 |
| glutamate receptors | C0061465 | 6 | 7 | 5 | 0.714285714285714 |
| Glu receptors | C0061465 | 6 | 7 | 5 | 0.714285714285714 |
| glutamate metabotropic receptors | C0061465 | 6 | 7 | 5 | 0.714285714285714 |
| glutamate receptor | C0061465 | 6 | 7 | 5 | 0.714285714285714 |
| tyrosine hydroxylase | 25085 | 6 | 2 | 5 | 2.5 |
| nitrotyrosine | C0047645 | 8 | 4 | 5 | 1.25 |
| 3-nitrotyrosine | C0047645 | 8 | 4 | 5 | 1.25 |
| NT | C0047645 | 8 | 4 | 5 | 1.25 |
| caspase-9 | 58918 | 3 | 1 | 5 | 5 |
| sonic hedgehog | 6469 | 7 | 7 | 5 | 0.714285714285714 |
| Shh | 6469 | 7 | 7 | 5 | 0.714285714285714 |
| per 1 | C3273591 | 1 | 2 | 5 | 2.5 |
| April | C3715025 |  | 3 | 5 | 1.66666666666667 |
| ion channels | C0022009 | 6 | 5 | 5 | 1 |
| ion channel | C0022009 | 6 | 5 | 5 | 1 |
| membrane ion channels | C0022009 | 6 | 5 | 5 | 1 |
| membrane channel | C0022009 | 6 | 5 | 5 | 1 |
| arginine | C0003765 | 6 | 3 | 5 | 1.66666666666667 |
| L-arginine | C0003765 | 6 | 3 | 5 | 1.66666666666667 |
| glycoprotein | C0017968 | 5 | 1 | 5 | 5 |
| vesicular monoamine transporter-type 2 | C3539788 | 5 | 8 | 5 | 0.625 |
| monoamine neurotransmitter transporter | C3539788 | 5 | 8 | 5 | 0.625 |
| monoamine transporters | C3539788 | 5 | 8 | 5 | 0.625 |
| MATs | C3539788 | 5 | 8 | 5 | 0.625 |
| cyclooxygenase-2 | 5743 | 5 | 9 | 5 | 0.555555555555556 |
| GABA(A) receptor | C0034807 | 5 | 7 | 5 | 0.714285714285714 |
| GABA(A) receptors | C0034807 | 5 | 7 | 5 | 0.714285714285714 |
| aspartate receptor | C0052536 | 5 | 6 | 5 | 0.833333333333333 |
| aspartic acid receptor | C0052536 | 5 | 6 | 5 | 0.833333333333333 |
| aspartate NR2B receptor | C0052536 | 5 | 6 | 5 | 0.833333333333333 |
| aspartate receptors | C0052536 | 5 | 6 | 5 | 0.833333333333333 |
| pro-apoptotic protein | C0379214 | 4 | 4 | 5 | 1.25 |
| pro-cell death protein | C0379214 | 4 | 4 | 5 | 1.25 |
| beta2 | 4760 | 5 | 8 | 5 | 0.625 |
| beta2* nAChR | 4760 | 5 | 8 | 5 | 0.625 |
| beta2(*) nAChR | 4760 | 5 | 8 | 5 | 0.625 |
| alpha6beta2 | 4760 | 5 | 8 | 5 | 0.625 |
| alpha4beta2(*) ( | 4760 | 5 | 8 | 5 | 0.625 |
| alpha4beta2 | 4760 | 5 | 8 | 5 | 0.625 |
| beta2- | 4760 | 5 | 8 | 5 | 0.625 |
| SMA | 6606 | 5 | 5 | 5 | 1 |
| ICD | 79158 | 2 | 8 | 5 | 0.625 |
| Brain-derived neurotrophic factor | C0107103 | 4 | 2 | 5 | 2.5 |
| BDNF | C0107103 | 4 | 2 | 5 | 2.5 |
| methionine | C0025646 | 4 | 4 | 5 | 1.25 |
| SIR2 | 23411 | 3 | 7 | 5 | 0.714285714285714 |
| SIRT1 | 23411 | 3 | 7 | 5 | 0.714285714285714 |
| SIRT1-7 | 23411 | 3 | 7 | 5 | 0.714285714285714 |
| tagging | 404663 | 4 | 4 | 5 | 1.25 |
| cdk5 | 1020 | 1 | 6 | 5 | 0.833333333333333 |
| adenosine A2A receptor | 25369 | 4 | 4 | 5 | 1.25 |
| adenosine A(2A) receptor | 25369 | 4 | 4 | 5 | 1.25 |
| A2AR | 25369 | 4 | 4 | 5 | 1.25 |
| cyclooxygenase-2 | 19225 | 4 | 3 | 5 | 1.66666666666667 |
| gene- | C0017337 | 2 | 1 | 5 | 5 |
| cytochrome c | C0010749 | 2 | 6 | 5 | 0.833333333333333 |
| anti-phosphorylated alpha-synuclein (alphaS) antibody | C0003242 | 3 | 3 | 5 | 1.66666666666667 |
| anti-tyrosine hydroxylase antibody | C0003242 | 3 | 3 | 5 | 1.66666666666667 |
| anti-neuronal nuclei antibody | C0003242 | 3 | 3 | 5 | 1.66666666666667 |
| anti-glial fibrillary acidic protein antibody | C0003242 | 3 | 3 | 5 | 1.66666666666667 |
| anti-S 100beta antibody | C0003242 | 3 | 3 | 5 | 1.66666666666667 |
| anti-nestin antibody | C0003242 | 3 | 3 | 5 | 1.66666666666667 |
| Anti-oligomeric Abeta single-chain variable domain antibody | C0003242 | 3 | 3 | 5 | 1.66666666666667 |
| nuclear receptor | C0206588 | 3 | 5 | 5 | 1 |
| LPD | 65059 | 3 | 1 | 5 | 5 |
| chromatin | C0008546 | 3 | 3 | 5 | 1.66666666666667 |
| PI3 kinase | 5266 | 3 |  | 5 |  |
| PI3- | 5266 | 3 |  | 5 |  |
| COX-2 | 26198 | 1 | 4 | 5 | 1.25 |
| NFL | 4747 |  | 1 | 5 | 5 |
| ACh | 2261 | 2 | 1 | 5 | 5 |
| Botulinum toxin type A | C0006050 |  | 1 | 5 | 5 |
| Drp1 | 1400 | 1 | 3 | 5 | 1.66666666666667 |
| atg5 gene | 9474 | 1 | 3 | 5 | 1.66666666666667 |
| Go-No | 92344 | 2 | 1 | 5 | 5 |
| PKC | 112476 | 1 | 3 | 5 | 1.66666666666667 |
| PINK1 | 494085 | 1 | 1 | 5 | 5 |
| Asn( | C0003995 | 1 | 2 | 5 | 2.5 |
| ICF | 1789 | 1 |  | 5 |  |
| SIRT3 | 23410 |  |  | 5 |  |
| sirtuin 3 | 23410 |  |  | 5 |  |
| receptor tyrosine kinase | C0206364 | 1 | 1 | 5 | 5 |
| XBP-1 | 7494 | 1 | 2 | 5 | 2.5 |
| Go/ | 92344 | 2 | 1 | 5 | 5 |
| D1-4 | 25802 |  | 2 | 5 | 2.5 |
| disease susceptibility genes | C0919453 | 10 | 9 | 5 | 0.555555555555556 |
| HtrA2 genes | 27429 | 9 | 10 | 5 | 0.5 |
| PARK13 | 27429 | 9 | 10 | 5 | 0.5 |
| monoamine transporter | C3539788 | 5 | 8 | 5 | 0.625 |
| cyclin-dependent kinase 5 | 1020 | 1 | 6 | 5 | 0.833333333333333 |
| Interferon-gamma | 3458 | 2 | 6 | 5 | 0.833333333333333 |
| asparagine | C0003995 | 1 | 2 | 5 | 2.5 |
| cyclin-dependent kinase-5 | 1020 | 1 | 6 | 5 | 0.833333333333333 |
| cyclooxygenase (COX)-2 | 5743 | 5 | 9 | 5 | 0.555555555555556 |
| DSR | C0036720 |  | 3 | 5 | 1.66666666666667 |
| A(2A) R | 25369 | 4 | 4 | 5 | 1.25 |
| neurofilament triplet protein | C0085418 |  | 2 | 5 | 2.5 |
| NF-L | 4747 |  | 1 | 5 | 5 |
| polyQ>/ | C0384782 | 10 | 16 | 5 | 0.3125 |
| polyQ</ | C0384782 | 10 | 16 | 5 | 0.3125 |
| BTX type A | C0006050 |  | 1 | 5 | 5 |
| Cerebral dopamine neurotrophic factor | 441549 |  | 3 | 5 | 1.66666666666667 |
| serines | C0036720 |  | 3 | 5 | 1.66666666666667 |
| X-box binding protein-1 | 7494 | 1 | 2 | 5 | 2.5 |
| XBP1 | 7494 | 1 | 2 | 5 | 2.5 |
| vesicular monoamine transporter type 2 | C3539788 | 5 | 8 | 5 | 0.625 |
| heme oxygenase 1 | 24451 |  | 5 | 5 | 1 |
| nuclear receptors | C0206588 | 3 | 5 | 5 | 1 |
| anti-alpha-synuclein antibody | C0003242 | 3 | 3 | 5 | 1.66666666666667 |
| PD susceptibility genes | C0919453 | 10 | 9 | 5 | 0.555555555555556 |
| ionic channels | C0022009 | 6 | 5 | 5 | 1 |
| glycoproteins | C0017968 | 5 | 1 | 5 | 5 |
| pro-inflammatory proteins | C0379214 | 4 | 4 | 5 | 1.25 |
| collapsin response mediator protein 1 | 1400 | 1 | 3 | 5 | 1.66666666666667 |
| CRMP-1 | 1400 | 1 | 3 | 5 | 1.66666666666667 |
| CDNF | 441549 |  | 3 | 5 | 1.66666666666667 |
| Go-/No | 92344 | 2 | 1 | 5 | 5 |
| BST1 | 683 |  | 5 | 5 | 1 |
| PARK16 | 100359403 |  | 3 | 5 | 1.66666666666667 |
| taurine | C0039350 |  | 2 | 5 | 2.5 |
| neurofilament proteins | C0085418 |  | 2 | 5 | 2.5 |
| C9ORF72 | 203228 |  | 7 | 5 | 0.714285714285714 |
| CA3 | 761 |  | 2 | 5 | 2.5 |
| D1R | 25802 |  | 2 | 5 | 2.5 |
| miR-7 | 10859 |  | 2 | 5 | 2.5 |
| heme-oxygenase 1 | 24451 |  | 5 | 5 | 1 |
| HO-1 | 24451 |  | 5 | 5 | 1 |
| anti-BDNF antibody | C0003242 | 3 | 3 | 5 | 1.66666666666667 |
| tag | 404663 | 4 | 4 | 5 | 1.25 |
| vascular endothelial growth factor | 7422 |  | 2 | 5 | 2.5 |
| VEGF | 7422 |  | 2 | 5 | 2.5 |
| Asn/ | C0003995 | 1 | 2 | 5 | 2.5 |
| Asn | C0003995 | 1 | 2 | 5 | 2.5 |
| beta-catenin | 12387 |  | 1 | 5 | 5 |
| botulinum neurotoxin type A | C0006050 |  | 1 | 5 | 5 |
| Atg5 | 9474 | 1 | 3 | 5 | 1.66666666666667 |
| Gelb | 4318 |  | 3 | 5 | 1.66666666666667 |
| S129 | C0036720 |  | 3 | 5 | 1.66666666666667 |
| chromosome 9 open reading frame 72 | 203228 |  | 7 | 5 | 0.714285714285714 |
| mGluR(5 | 14805 | 4 | 4 | 5 | 1.25 |
| glucagon-like peptide-1 | C0061355 |  | 1 | 5 | 5 |
| lysosomal enzyme glucocerebrosidase | C0017768 |  | 3 | 5 | 1.66666666666667 |
| CASP1 | 834 |  | 1 | 5 | 5 |
| MMP9 | 4318 |  | 3 | 5 | 1.66666666666667 |
| GABAA receptor | C0034807 | 5 | 7 | 5 | 0.714285714285714 |
| BST1 genes | 683 |  | 5 | 5 | 1 |
| SPSS | C3813609 |  | 1 | 5 | 5 |
| serum albumin | C0036773 |  | 1 | 5 | 5 |
| VEGF-A | 7422 |  | 2 | 5 | 2.5 |
| vascular endothelial growth factor-A | 7422 |  | 2 | 5 | 2.5 |
| aspartic acid receptors | C0052536 | 5 | 6 | 5 | 0.833333333333333 |
| SIRT1 gene | 23411 | 3 | 7 | 5 | 0.714285714285714 |
| heme oxygenase-1 | 24451 |  | 5 | 5 | 1 |
| caspase 9 | 58918 | 3 | 1 | 5 | 5 |
| IFNgamma | 3458 | 2 | 6 | 5 | 0.833333333333333 |
| anti-glutamic acid decarboxylase (GAD) antibody | C0003242 | 3 | 3 | 5 | 1.66666666666667 |
| PRRT2 | 112476 | 1 | 3 | 5 | 1.66666666666667 |
| DYT10 | 112476 | 1 | 3 | 5 | 1.66666666666667 |
| GABAA benzodiazepine receptors | C0034807 | 5 | 7 | 5 | 0.714285714285714 |
| C9orf72 gene | 203228 |  | 7 | 5 | 0.714285714285714 |
| CD4 | 920 |  | 2 | 5 | 2.5 |
| alpha3beta2beta3 | 4760 | 5 | 8 | 5 | 0.625 |
| GLP-1 | C0061355 |  | 1 | 5 | 5 |
| anti-phospho-Ser935 antibody | C0003242 | 3 | 3 | 5 | 1.66666666666667 |
| BST1 gene | 683 |  | 5 | 5 | 1 |
| D1-D5 | 25802 |  | 2 | 5 | 2.5 |
| pro-autophagy protein | C0379214 | 4 | 4 | 5 | 1.25 |
| SMN1 | 6606 | 5 | 5 | 5 | 1 |
| go/no | 92344 | 2 | 1 | 5 | 5 |
| ATG) 5 | 9474 | 1 | 3 | 5 | 1.66666666666667 |
| DCGs | C0017337 | 2 | 1 | 5 | 5 |
| Atg-5 | 9474 | 1 | 3 | 5 | 1.66666666666667 |
| Gelb's | 4318 |  | 3 | 5 | 1.66666666666667 |
| GABA A/glycine receptors | C0034807 | 5 | 7 | 5 | 0.714285714285714 |
| X-box-binding protein 1 | 7494 | 1 | 2 | 5 | 2.5 |
| anti-parkin antibody | C0003242 | 3 | 3 | 5 | 1.66666666666667 |
| BNT-A | C0006050 |  | 1 | 5 | 5 |
| caspase-1 | 834 |  | 1 | 5 | 5 |
| D1-H3 | 25802 |  | 2 | 5 | 2.5 |
| membrane channels | C0022009 | 6 | 5 | 5 | 1 |
| COX2 | 26198 | 1 | 4 | 5 | 1.25 |
| membrane Cav1 channels | C0022009 | 6 | 5 | 5 | 1 |
| nuclear receptor-related 1 protein | C0206588 | 3 | 5 | 5 | 1 |
| PI3K | 5266 | 3 |  | 5 |  |
| HCs | 3141 |  |  | 5 |  |
| PTEN-induced putative kinase 1 | 494085 | 1 | 1 | 5 | 5 |
| aSN's | C0003995 | 1 | 2 | 5 | 2.5 |
| aHR | 196 |  |  | 5 |  |
| SIRT1-SIRT7 | 23411 | 3 | 7 | 5 | 0.714285714285714 |
| anti-alpha-synuclein monoclonal antibody | C0003242 | 3 | 3 | 5 | 1.66666666666667 |
| Drp1significantly | 1400 | 1 | 3 | 5 | 1.66666666666667 |
| DA D1 receptor | 25802 |  | 2 | 5 | 2.5 |
| D1-MSN | 25802 |  | 2 | 5 | 2.5 |
| D1DR | 25802 |  | 2 | 5 | 2.5 |
| cyclooxygenase 2 | 19225 | 4 | 3 | 5 | 1.66666666666667 |
| nuclear mineralocorticoid receptor | C0206588 | 3 | 5 | 5 | 1 |
| DNMT3B | 1789 | 1 |  | 5 |  |
| Intramembrane Proteases | 81502 |  |  | 5 |  |
| intramembrane protease | 81502 |  |  | 5 |  |
| intramembrane-cleaving proteases | 81502 |  |  | 5 |  |
| I-CLiPs | 81502 |  |  | 5 |  |
| receptor tyrosine kinase ( | C0206364 | 1 | 1 | 5 | 5 |
| pro-autophagic proteins | C0379214 | 4 | 4 | 5 | 1.25 |
| proline-rich transmembrane protein 2 | 112476 | 1 | 3 | 5 | 1.66666666666667 |
| anti-CD200R1 antibody | C0003242 | 3 | 3 | 5 | 1.66666666666667 |
| anti-LRRK2 antibody | C0003242 | 3 | 3 | 5 | 1.66666666666667 |
| neurofilament light chain (NfL) protein | C0085418 |  | 2 | 5 | 2.5 |
| tyrosine kinase receptors | C0206364 | 1 | 1 | 5 | 5 |
| neurofilament light chain protein | C0085418 |  | 2 | 5 | 2.5 |
| BTX-A | C0006050 |  | 1 | 5 | 5 |
| methionines | C0025646 | 4 | 4 | 5 | 1.25 |
| nicotinic receptor beta2 | 4760 | 5 | 8 | 5 | 0.625 |
| transforming growth factor-beta2 | 4760 | 5 | 8 | 5 | 0.625 |
| DEGs | C0017337 | 2 | 1 | 5 | 5 |
| human CDNF | 441549 |  | 3 | 5 | 1.66666666666667 |
| rhCDNF | 441549 |  | 3 | 5 | 1.66666666666667 |
| glutamate-receptor | C0061465 | 6 | 7 | 5 | 0.714285714285714 |
| glucagon-like peptide (GLP)-1 | C0061355 |  | 1 | 5 | 5 |
| CD157 | 683 |  | 5 | 5 | 1 |
| BST-1 | 683 |  | 5 | 5 | 1 |
| MMP-9 | 4318 |  | 3 | 5 | 1.66666666666667 |
| GABAa receptors | C0034807 | 5 | 7 | 5 | 0.714285714285714 |
| NMDARs | C0052536 | 5 | 6 | 5 | 0.833333333333333 |
| pro-inflammatory protein | C0379214 | 4 | 4 | 5 | 1.25 |
| NEUROD1 | 4760 | 5 | 8 | 5 | 0.625 |
| abobotulinumtoxinA | C0006050 |  | 1 | 5 | 5 |
| cytochrome-c | C0010749 | 2 | 6 | 5 | 0.833333333333333 |
| D1-3 | 25802 |  | 2 | 5 | 2.5 |
| anti-phosphoTrkA antibody | C0003242 | 3 | 3 | 5 | 1.66666666666667 |
| CRMP1 | 1400 | 1 | 3 | 5 | 1.66666666666667 |
| CD157 KO | 683 |  | 5 | 5 | 1 |
| polyQ-Htt | C0384782 | 10 | 16 | 5 | 0.3125 |
| BoNT-A | C0006050 |  | 1 | 5 | 5 |
| glucocerebrosidases | C0017768 |  | 3 | 5 | 1.66666666666667 |
| L-serine | C0036720 |  | 3 | 5 | 1.66666666666667 |
| human cerebral dopamine neurotrophic factor | 441549 |  | 3 | 5 | 1.66666666666667 |
| cyclin-dependent protein kinase 5 | 1020 | 1 | 6 | 5 | 0.833333333333333 |
| incobotulinumtoxinA | C0006050 |  | 1 | 5 | 5 |
| SMN | 6606 | 5 | 5 | 5 | 1 |
| S185 | C0036720 |  | 3 | 5 | 1.66666666666667 |
| S232 | C0036720 |  | 3 | 5 | 1.66666666666667 |
| X-Box binding protein 1 | 7494 | 1 | 2 | 5 | 2.5 |
| spp | 81502 |  |  | 5 |  |
| lysosomal enzyme acid beta-glucosidase | C0017768 |  | 3 | 5 | 1.66666666666667 |
| RING | C0017337 | 2 | 1 | 5 | 5 |
| IncoA | C0006050 |  | 1 | 5 | 5 |
| iTRAQ | 404663 | 4 | 4 | 5 | 1.25 |
| FuG-E | C0017968 | 5 | 1 | 5 | 5 |
| S129P | C0036720 |  | 3 | 5 | 1.66666666666667 |
| sirtuin 1 | 23411 | 3 | 7 | 5 | 0.714285714285714 |
| Sirt 1 | 23411 | 3 | 7 | 5 | 0.714285714285714 |
| spp. | 81502 |  |  | 5 |  |
| PI3 | 5266 | 3 |  | 5 |  |
| conserved dopamine neurotrophic factor | 441549 |  | 3 | 5 | 1.66666666666667 |
| beta2AR | 4760 | 5 | 8 | 5 | 0.625 |
| tyrosine kinase collagen-activated receptors | C0206364 | 1 | 1 | 5 | 5 |
| caspase 1 | 834 |  | 1 | 5 | 5 |
| Asn- | C0003995 | 1 | 2 | 5 | 2.5 |
| neurofilament protein | C0085418 |  | 2 | 5 | 2.5 |
| NPNFP | C0085418 |  | 2 | 5 | 2.5 |
| aryl hydrocarbon receptor | 196 |  |  | 5 |  |
| 1D-IR | 25802 |  | 2 | 5 | 2.5 |
| Cytc | C0010749 | 2 | 6 | 5 | 0.833333333333333 |
| DNA methyltransferase 3b | 1789 | 1 |  | 5 |  |
| glucagon like peptide-1 | C0061355 |  | 1 | 5 | 5 |
| GABAA rho1 receptors | C0034807 | 5 | 7 | 5 | 0.714285714285714 |
| GABAA rho1 receptor- | C0034807 | 5 | 7 | 5 | 0.714285714285714 |
| NFP | C0085418 |  | 2 | 5 | 2.5 |
| nuclear RAS receptors | C0206588 | 3 | 5 | 5 | 1 |
| Syn | 23336 | 6 | 3 | 4 | 1.33333333333333 |
| specific p53 antibody | C0443640 | 7 | 5 | 4 | 0.8 |
| specific antibodies | C0443640 | 7 | 5 | 4 | 0.8 |
| specific A11 antibody | C0443640 | 7 | 5 | 4 | 0.8 |
| specific 'M2M6' antibodies | C0443640 | 7 | 5 | 4 | 0.8 |
| specific primary antibody | C0443640 | 7 | 5 | 4 | 0.8 |
| fusion protein | C0162768 | 11 | 8 | 4 | 0.5 |
| fusion proteins | C0162768 | 11 | 8 | 4 | 0.5 |
| chimeric protein | C0162768 | 11 | 8 | 4 | 0.5 |
| reductase | C0030016 | 3 | 6 | 4 | 0.666666666666667 |
| oxido-reductase | C0030016 | 3 | 6 | 4 | 0.666666666666667 |
| PARK9 | 23400 | 2 | 16 | 4 | 0.25 |
| ATP13A2 | 23400 | 2 | 16 | 4 | 0.25 |
| ATP13A2 genes | 23400 | 2 | 16 | 4 | 0.25 |
| PS) | 653247 | 2 | 1 | 4 | 4 |
| SCOPA-PS | 653247 | 2 | 1 | 4 | 4 |
| serotonin transporter | C0170657 | 8 | 5 | 4 | 0.8 |
| serotonin transporters | C0170657 | 8 | 5 | 4 | 0.8 |
| 5HT transporter | C0170657 | 8 | 5 | 4 | 0.8 |
| beta-III-tubulin | C0005290 | 4 | 5 | 4 | 0.8 |
| beta-tubulin | C0005290 | 4 | 5 | 4 | 0.8 |
| doublecortin | 1641 | 5 | 1 | 4 | 4 |
| E14 | 4863 | 7 | 3 | 4 | 1.33333333333333 |
| E-14 | 4863 | 7 | 3 | 4 | 1.33333333333333 |
| DJ-1 | 117287 | 1 | 4 | 4 | 1 |
| tyrosinase | 7299 | 6 | 7 | 4 | 0.571428571428571 |
| Tyr | 7299 | 6 | 7 | 4 | 0.571428571428571 |
| natively unfolded proteins | C3658247 | 6 | 3 | 4 | 1.33333333333333 |
| unstructured proteins | C3658247 | 6 | 3 | 4 | 1.33333333333333 |
| protein structure | C0175168 | 5 | 9 | 4 | 0.444444444444444 |
| protein structures | C0175168 | 5 | 9 | 4 | 0.444444444444444 |
| SAR | 1757 | 5 | 3 | 4 | 1.33333333333333 |
| actin | C0001271 | 4 | 3 | 4 | 1.33333333333333 |
| peroxisome proliferator activated receptor-gamma | 5468 | 2 | 8 | 4 | 0.5 |
| PPAR-gamma | 5468 | 2 | 8 | 4 | 0.5 |
| peroxisome proliferator-activated receptor gamma | 5468 | 2 | 8 | 4 | 0.5 |
| PPARgamma | 5468 | 2 | 8 | 4 | 0.5 |
| SERT protein | C0007292 | 2 | 4 | 4 | 1 |
| transporter protein | C0007292 | 2 | 4 | 4 | 1 |
| carrier protein | C0007292 | 2 | 4 | 4 | 1 |
| interleukin-1beta | C0021753 | 4 | 6 | 4 | 0.666666666666667 |
| interleukin (IL)-1 beta | C0021753 | 4 | 6 | 4 | 0.666666666666667 |
| interleukin-1 (IL-1) beta | C0021753 | 4 | 6 | 4 | 0.666666666666667 |
| dynamin-related protein 1 | 10059 | 1 | 8 | 4 | 0.5 |
| DLP1 | 10059 | 1 | 8 | 4 | 0.5 |
| Drp1 | 10059 | 1 | 8 | 4 | 0.5 |
| neural cell adhesion molecule | C0282587 | 1 |  | 4 |  |
| PSA-NCAM | C0282587 | 1 |  | 4 |  |
| PTEN | 5728 | 4 | 2 | 4 | 2 |
| PTEN-induced | 5728 | 4 | 2 | 4 | 2 |
| anti-keratin monoclonal antibodies | C1624602 | 4 | 5 | 4 | 0.8 |
| anti-sonic hedgehog antibodies | C1624602 | 4 | 5 | 4 | 0.8 |
| anti-acetylcholinesterase antibodies | C1624602 | 4 | 5 | 4 | 0.8 |
| anti-dopamine/vesicular monoamine transporter 2 (VMAT2) antibodies | C1624602 | 4 | 5 | 4 | 0.8 |
| anti-nitrotyrosine antibodies | C1624602 | 4 | 5 | 4 | 0.8 |
| gamma-synuclein | C0753208 | 4 | 3 | 4 | 1.33333333333333 |
| HSPA5 | 3309 | 4 | 2 | 4 | 2 |
| HSPA5 -415 | 3309 | 4 | 2 | 4 | 2 |
| immunoglobulin heavy chain binding protein | 3309 | 4 | 2 | 4 | 2 |
| BiP | 3309 | 4 | 2 | 4 | 2 |
| Grp78 | 3309 | 4 | 2 | 4 | 2 |
| COX-2 | 17709 | 4 | 3 | 4 | 1.33333333333333 |
| traits | 55840 | 2 | 2 | 4 | 2 |
| neuronal nitric oxide synthase | 18125 | 3 | 3 | 4 | 1.33333333333333 |
| nNOS | 18125 | 3 | 3 | 4 | 1.33333333333333 |
| P21 | 1026 | 2 | 5 | 4 | 0.8 |
| WAF1/CIP1 | 1026 | 2 | 5 | 4 | 0.8 |
| protein function | C0815043 | 3 | 3 | 4 | 1.33333333333333 |
| oncogene | C0029016 | 3 | 2 | 4 | 2 |
| catalase | 847 | 3 | 3 | 4 | 1.33333333333333 |
| membrane associated proteins | C0025252 |  | 1 | 4 | 4 |
| membrane type-1 protein | C0025252 |  | 1 | 4 | 4 |
| progranulin | 2896 | 3 | 2 | 4 | 2 |
| PGRN | 2896 | 3 | 2 | 4 | 2 |
| genetic features | C1517495 | 3 | 1 | 4 | 4 |
| genetic characteristics | C1517495 | 3 | 1 | 4 | 4 |
| MER | 2852 | 2 | 5 | 4 | 0.8 |
| C-reactive protein | 1401 | 1 | 4 | 4 | 1 |
| CA2 | 760 | 3 | 1 | 4 | 4 |
| Ca2+-activated K+ channels | 760 | 3 | 1 | 4 | 4 |
| EC 1.6.5.3 | C0020289 | 3 | 2 | 4 | 2 |
| hydrolases | C0020289 | 3 | 2 | 4 | 2 |
| Mitochondrial transcription factor A | 7019 | 1 | 4 | 4 | 1 |
| TFAM | 7019 | 1 | 4 | 4 | 1 |
| PLA2 | 8398 | 2 | 4 | 4 | 1 |
| phospholipase A(2) group VI | 8398 | 2 | 4 | 4 | 1 |
| PLA2G6 | 8398 | 2 | 4 | 4 | 1 |
| dPD | 1806 | 2 | 1 | 4 | 4 |
| dopamine D3 receptor | 13490 | 1 |  | 4 |  |
| adenosine A2A receptors | C0255998 | 2 | 1 | 4 | 4 |
| trans-activator | C0040627 | 2 | 2 | 4 | 2 |
| TAT | C0040627 | 2 | 2 | 4 | 2 |
| APOE epsilon4 allele | C3642141 | 2 | 1 | 4 | 4 |
| BH4 | C0048897 | 2 | 1 | 4 | 4 |
| Marf | 9927 | 2 | 3 | 4 | 1.33333333333333 |
| PSEN1 | 5663 | 1 | 1 | 4 | 4 |
| transferrin | 7018 | 2 | 3 | 4 | 1.33333333333333 |
| kynurenine | C0022818 | 1 |  | 4 |  |
| p25alpha | 11076 | 2 | 1 | 4 | 4 |
| p25alpha's | 11076 | 2 | 1 | 4 | 4 |
| tubulin polymerization promoting protein | 11076 | 2 | 1 | 4 | 4 |
| TPPP | 11076 | 2 | 1 | 4 | 4 |
| MEP | 1514 | 2 |  | 4 |  |
| p38 | 26416 | 2 | 2 | 4 | 2 |
| p38 MAPK | 26416 | 2 | 2 | 4 | 2 |
| p38MAPK | 26416 | 2 | 2 | 4 | 2 |
| Na(+)/K(+) ATPase | C0001479 | 2 | 1 | 4 | 4 |
| Na+,K+-ATPase | C0001479 | 2 | 1 | 4 | 4 |
| AFM | 173 | 1 | 2 | 4 | 2 |
| MSNs | 4478 | 1 | 3 | 4 | 1.33333333333333 |
| i) altered protein | C0087044 |  | 2 | 4 | 2 |
| alpha7 nAChR | C0051334 | 2 | 2 | 4 | 2 |
| CCK | 885 | 1 | 1 | 4 | 4 |
| cholecystokinin | 885 | 1 | 1 | 4 | 4 |
| 26 S proteasome | C0286330 | 2 | 2 | 4 | 2 |
| 26 S proteasomes | C0286330 | 2 | 2 | 4 | 2 |
| 26 S proteasomal | C0286330 | 2 | 2 | 4 | 2 |
| 26S proteasomes | C0286330 | 2 | 2 | 4 | 2 |
| 26S proteasomal | C0286330 | 2 | 2 | 4 | 2 |
| 26S proteasome | C0286330 | 2 | 2 | 4 | 2 |
| NoGo | 57142 | 2 | 1 | 4 | 4 |
| CHIP | 10273 | 2 | 3 | 4 | 1.33333333333333 |
| at 2 | 186 | 2 | 1 | 4 | 4 |
| Ghrelin | 58991 | 1 |  | 4 |  |
| mitochondrial genome | C1819716 | 1 | 2 | 4 | 2 |
| SNAP-25 | 6616 |  |  | 4 |  |
| IL-4 | 3565 | 1 | 1 | 4 | 4 |
| ghrelin | C0911014 |  | 3 | 4 | 1.33333333333333 |
| Hsp70-interacting protein | 6767 | 1 |  | 4 |  |
| human leukocyte antigen | C0019629 |  | 2 | 4 | 2 |
| IL-2 | 3558 | 1 |  | 4 |  |
| MTHFR | 4524 |  | 2 | 4 | 2 |
| APOE epsilon4 alleles | C3642141 | 2 | 1 | 4 | 4 |
| oxidoreductase | C0030016 | 3 | 6 | 4 | 0.666666666666667 |
| mitofusin-2 | 9927 | 2 | 3 | 4 | 1.33333333333333 |
| peroxisome proliferator-activated receptor-gamma | C0166417 | 1 | 3 | 4 | 1.33333333333333 |
| sodium-potassium pump | C0001479 | 2 | 1 | 4 | 4 |
| cancer genes | C0029016 | 3 | 2 | 4 | 2 |
| PEPI | 2896 | 3 | 2 | 4 | 2 |
| interleukin (IL)-1beta | C0021753 | 4 | 6 | 4 | 0.666666666666667 |
| AT2 | 186 | 2 | 1 | 4 | 4 |
| anti-melanocytes antibodies | C1624602 | 4 | 5 | 4 | 0.8 |
| CRP | 1401 | 1 | 4 | 4 | 1 |
| Prdx2 | 7001 |  | 2 | 4 | 2 |
| light | 8740 |  | 3 | 4 | 1.33333333333333 |
| intrinsically disordered proteins | C3658247 | 6 | 3 | 4 | 1.33333333333333 |
| Kynurenines | C0022818 | 1 |  | 4 |  |
| LC3 | 8740 |  | 3 | 4 | 1.33333333333333 |
| neuronal NOS | 18125 | 3 | 3 | 4 | 1.33333333333333 |
| peroxisome proliferator-activated receptor gamma2 | 5468 | 2 | 8 | 4 | 0.5 |
| PPARgamma | C0166417 | 1 | 3 | 4 | 1.33333333333333 |
| TUG | 79058 |  | 3 | 4 | 1.33333333333333 |
| MANF | 7873 |  | 2 | 4 | 2 |
| physiological full-length protein | C0815043 | 3 | 3 | 4 | 1.33333333333333 |
| nuclear factor erythroid 2-related factor 2 | 4780 |  | 5 | 4 | 0.8 |
| DMN | 23336 | 6 | 3 | 4 | 1.33333333333333 |
| NBIA2 | 8398 | 2 | 4 | 4 | 1 |
| p600 | 3596 |  | 1 | 4 | 4 |
| cfp | 5199 |  | 2 | 4 | 2 |
| lysosome-associated membrane protein 2 | 3920 |  | 3 | 4 | 1.33333333333333 |
| LAMP2 | 3920 |  | 3 | 4 | 1.33333333333333 |
| Parkinson disease (autosomal recessive, early onset) 7 | 117287 | 1 | 4 | 4 | 1 |
| Park7 | 117287 | 1 | 4 | 4 | 1 |
| Cox2 | 17709 | 4 | 3 | 4 | 1.33333333333333 |
| Hsp70 interacting protein | 6767 | 1 |  | 4 |  |
| alpha7 nAChRs | C0051334 | 2 | 2 | 4 | 2 |
| inositol-requiring enzyme 1 | 2081 |  |  | 4 |  |
| IRE1 | 2081 |  |  | 4 |  |
| transferrins | 7018 | 2 | 3 | 4 | 1.33333333333333 |
| caspase-1 | 12362 |  | 1 | 4 | 4 |
| FUS | 2521 |  | 3 | 4 | 1.33333333333333 |
| PARK14 | 8398 | 2 | 4 | 4 | 1 |
| Mfn2 | 9927 | 2 | 3 | 4 | 1.33333333333333 |
| Opa1 | 4976 |  |  | 4 |  |
| synapsin-1 | C0087044 |  | 2 | 4 | 2 |
| IFNgamma | 15978 |  | 2 | 4 | 2 |
| ATP13A2 gene | 23400 | 2 | 16 | 4 | 0.25 |
| M83 | 58986 |  | 1 | 4 | 4 |
| SD 4.2 | C0019737 |  | 4 | 4 | 1 |
| DAT protein | C0007292 | 2 | 4 | 4 | 1 |
| PLA2G6 gene | 8398 | 2 | 4 | 4 | 1 |
| chimeric proteins | C0162768 | 11 | 8 | 4 | 0.5 |
| RAB7L1 | 8934 |  | 1 | 4 | 4 |
| peroxisome proliferator-activated receptor-gamma | 5468 | 2 | 8 | 4 | 0.5 |
| transport proteins | C0007292 | 2 | 4 | 4 | 1 |
| PTEN)- | 5728 | 4 | 2 | 4 | 2 |
| anti-phosphorylated alpha-synuclein (palphaSyn) antibodies | C1624602 | 4 | 5 | 4 | 0.8 |
| GRN | 2896 | 3 | 2 | 4 | 2 |
| ATPase type 13A2 | 23400 | 2 | 16 | 4 | 0.25 |
| GLT-1 | 6506 |  | 2 | 4 | 2 |
| MEK | 17242 |  | 1 | 4 | 4 |
| alpha7 nicotinic acetylcholine receptor | C0051334 | 2 | 2 | 4 | 2 |
| Moesin | 4478 | 1 | 3 | 4 | 1.33333333333333 |
| transforming growth factor-beta1 | C1515406 |  | 1 | 4 | 4 |
| TGF-beta1 | C1515406 |  | 1 | 4 | 4 |
| Nrf2 | 4780 |  | 5 | 4 | 0.8 |
| Iba1 | 199 |  | 2 | 4 | 2 |
| Rab5 | 5868 |  | 1 | 4 | 4 |
| dehydrogenase | C0030016 | 3 | 6 | 4 | 0.666666666666667 |
| Ca(II) | 760 | 3 | 1 | 4 | 4 |
| incretin hormone | C0017132 |  | 1 | 4 | 4 |
| L-type voltage-sensitive calcium channels | C0288263 |  | 2 | 4 | 2 |
| GPER1 | 2852 | 2 | 5 | 4 | 0.8 |
| peroxisome-proliferator-activated receptor gamma co-activator | C0166417 | 1 | 3 | 4 | 1.33333333333333 |
| dynamin-like protein 1 | 10059 | 1 | 8 | 4 | 0.5 |
| protein 1 | C0087044 |  | 2 | 4 | 2 |
| HLA | C0019629 |  | 2 | 4 | 2 |
| programmed cell death proteins | C1564881 |  | 3 | 4 | 1.33333333333333 |
| BPH4 | C0048897 | 2 | 1 | 4 | 4 |
| TPPP/p25 | 11076 | 2 | 1 | 4 | 4 |
| p25 | 11076 | 2 | 1 | 4 | 4 |
| NeuN | 146713 |  | 2 | 4 | 2 |
| Nuclear factor erythroid-2 related factor 2 | 4780 |  | 5 | 4 | 0.8 |
| NOS | 18125 | 3 | 3 | 4 | 1.33333333333333 |
| anti-CD68 antibodies | C1624602 | 4 | 5 | 4 | 0.8 |
| p38MAPK | C1120843 |  | 3 | 4 | 1.33333333333333 |
| p38 MAPK | C1120843 |  | 3 | 4 | 1.33333333333333 |
| PDSS)-2 | 57107 |  | 1 | 4 | 4 |
| PDSS-2 | 57107 |  | 1 | 4 | 4 |
| lysosome-associated membrane protein-2 | 3920 |  | 3 | 4 | 1.33333333333333 |
| LAMP2B | 3920 |  | 3 | 4 | 1.33333333333333 |
| P2X7 | 5027 |  | 1 | 4 | 4 |
| C/EBP homologous protein | 29467 |  | 2 | 4 | 2 |
| sPD) | 3239 |  | 2 | 4 | 2 |
| trans-acting factor | C0040627 | 2 | 2 | 4 | 2 |
| apoptotic proteins | C1564881 |  | 3 | 4 | 1.33333333333333 |
| Dynamin like protein 1 | 10059 | 1 | 8 | 4 | 0.5 |
| SD 7.2 | C0019737 |  | 4 | 4 | 1 |
| Peroxisome proliferator-activated receptor-gamma coactivator | C0166417 | 1 | 3 | 4 | 1.33333333333333 |
| Ps < . | 653247 | 2 | 1 | 4 | 4 |
| protein's structure | C0175168 | 5 | 9 | 4 | 0.444444444444444 |
| p21-activated kinases | 1026 | 2 | 5 | 4 | 0.8 |
| PAK | 1026 | 2 | 5 | 4 | 0.8 |
| proteins' spatial structure | C0175168 | 5 | 9 | 4 | 0.444444444444444 |
| DJ1 | 117287 | 1 | 4 | 4 | 1 |
| anti TH antibodies | C1624602 | 4 | 5 | 4 | 0.8 |
| p21(ras | 1026 | 2 | 5 | 4 | 0.8 |
| mitochondrial genomes | C1819716 | 1 | 2 | 4 | 2 |
| CD200 | 4345 |  | 1 | 4 | 4 |
| FOXO3a | 2309 |  | 1 | 4 | 4 |
| glucagon-like peptide 1 receptor | 2740 |  |  | 4 |  |
| SD 6.2 | C0019737 |  | 4 | 4 | 1 |
| PLA2G6 genes | 8398 | 2 | 4 | 4 | 1 |
| Mitofusin 2 | 9927 | 2 | 3 | 4 | 1.33333333333333 |
| L-type voltage-dependent calcium channels | C0288263 |  | 2 | 4 | 2 |
| transactivator | C0040627 | 2 | 2 | 4 | 2 |
| TTA | C0040627 | 2 | 2 | 4 | 2 |
| LAMP-2 | 3920 |  | 3 | 4 | 1.33333333333333 |
| GEP | 2896 | 3 | 2 | 4 | 2 |
| anti-C9orf72 antibodies | C1624602 | 4 | 5 | 4 | 0.8 |
| forkhead box O3a | 2309 |  | 1 | 4 | 4 |
| IFN-gamma | 15978 |  | 2 | 4 | 2 |
| GPER-1 | 2852 | 2 | 5 | 4 | 0.8 |
| glucagon-like peptide-1 receptor | 2740 |  |  | 4 |  |
| GLP-1R | 2740 |  |  | 4 |  |
| gammaS | C0753208 | 4 | 3 | 4 | 1.33333333333333 |
| TFEB | 7942 |  | 1 | 4 | 4 |
| transcription factor EB | 7942 |  | 1 | 4 | 4 |
| KP | C0022818 | 1 |  | 4 |  |
| CHOP | 29467 |  | 2 | 4 | 2 |
| C/EBP, homologous protein 10 | 29467 |  | 2 | 4 | 2 |
| Apolipoprotein E 4 allele | C3642141 | 2 | 1 | 4 | 4 |
| GMR | 1438 |  | 1 | 4 | 4 |
| proteins impact mitochondrial function | C0815043 | 3 | 3 | 4 | 1.33333333333333 |
| CCK-8 | 885 | 1 | 1 | 4 | 4 |
| CCK-B cholecystokinin receptors | 885 | 1 | 1 | 4 | 4 |
| SPD | 3239 |  | 2 | 4 | 2 |
| vascular cell adhesion molecule-1 | 7412 |  | 1 | 4 | 4 |
| VCAM-1 | 7412 |  | 1 | 4 | 4 |
| Alba | 173 | 1 | 2 | 4 | 2 |
| apoptosis-related proteins | C1564881 |  | 3 | 4 | 1.33333333333333 |
| CAT | 847 | 3 | 3 | 4 | 1.33333333333333 |
| sequence features | C1517495 | 3 | 1 | 4 | 4 |
| ionized calcium-binding adapter molecule-1 | 199 |  | 2 | 4 | 2 |
| L-type calcium channels | C0288263 |  | 2 | 4 | 2 |
| PPARgamma coactivator | C0166417 | 1 | 3 | 4 | 1.33333333333333 |
| beta-III tubulin | C0005290 | 4 | 5 | 4 | 0.8 |
| membrane-associated cytoplasmic proteins | C0025252 |  | 1 | 4 | 4 |
| Peroxisome proliferator-activated receptor gamma co- | C0166417 | 1 | 3 | 4 | 1.33333333333333 |
| peroxiredoxin 2 | 7001 |  | 2 | 4 | 2 |
| Prx2 | 7001 |  | 2 | 4 | 2 |
| specific domain antibody | C0443640 | 7 | 5 | 4 | 0.8 |
| IDPs | C3658247 | 6 | 3 | 4 | 1.33333333333333 |
| IL-4 | 16189 |  | 1 | 4 | 4 |
| hSYN | 23336 | 6 | 3 | 4 | 1.33333333333333 |
| transport protein | C0007292 | 2 | 4 | 4 | 1 |
| anti-alpha-synuclein antibodies | C1624602 | 4 | 5 | 4 | 0.8 |
| gamma synuclein | C0753208 | 4 | 3 | 4 | 1.33333333333333 |
| RAB7L1-NUCKS1 | 8934 |  | 1 | 4 | 4 |
| neuronal nuclei | 146713 |  | 2 | 4 | 2 |
| IRR = | 3645 |  |  | 4 |  |
| Iba1 | 29427 |  |  | 4 |  |
| mtTFA | 7019 | 1 | 4 | 4 | 1 |
| cancer-related genes | C0029016 | 3 | 2 | 4 | 2 |
| casp1 | 12362 |  | 1 | 4 | 4 |
| PS1 | 5663 | 1 | 1 | 4 | 4 |
| p.G2019 LRRK2 proteins | C0082731 |  |  | 4 |  |
| p.R1441H protein | C0082731 |  |  | 4 |  |
| transporter proteins | C0007292 | 2 | 4 | 4 | 1 |
| KYN | C0022818 | 1 |  | 4 |  |
| neurotrophic factor NT-3 | C0083735 |  |  | 4 |  |
| CHCHD10 | 400916 |  |  | 4 |  |
| P2X7 receptor | 5027 |  | 1 | 4 | 4 |
| CF-PC | 5420 |  |  | 4 |  |
| Synaptosomal-associated protein 25 kDa | 6616 |  |  | 4 |  |
| anti-PLP antibodies | C1624602 | 4 | 5 | 4 | 0.8 |
| presenilin 1 | 5663 | 1 | 1 | 4 | 4 |
| ps </= | 653247 | 2 | 1 | 4 | 4 |
| sarco/endoplasmic reticulum Ca2+-ATPase | 760 | 3 | 1 | 4 | 4 |
| IRR | 3645 |  |  | 4 |  |
| GPR30 | 2852 | 2 | 5 | 4 | 0.8 |
| G protein-coupled receptor 30 | 2852 | 2 | 5 | 4 | 0.8 |
| protein-function | C0815043 | 3 | 3 | 4 | 1.33333333333333 |
| nuclear factor erythroid 2 related factor 2 | 4780 |  | 5 | 4 | 0.8 |
| interferon-gamma | 15978 |  | 2 | 4 | 2 |
| CD200-CD200R | 4345 |  | 1 | 4 | 4 |
| Buffy | 36251 |  |  | 4 |  |
| PC+ | 5420 |  |  | 4 |  |
| surface proteins | C0025252 |  | 1 | 4 | 4 |
| TGFbeta1 | C1515406 |  | 1 | 4 | 4 |
| MSN-- | 4478 | 1 | 3 | 4 | 1.33333333333333 |
| Ps < | 653247 | 2 | 1 | 4 | 4 |
| VPS13C | 54832 |  |  | 4 |  |
| purinergic receptor subtype P2X7 | 5027 |  | 1 | 4 | 4 |
| apoptotic regulator | C1564881 |  | 3 | 4 | 1.33333333333333 |
| oxidoreductases | C0030016 | 3 | 6 | 4 | 0.666666666666667 |
| reductases | C0030016 | 3 | 6 | 4 | 0.666666666666667 |
| vascular cell adhesion molecule 1 | 7412 |  | 1 | 4 | 4 |
| membrane-embedded proteins | C0025252 |  | 1 | 4 | 4 |
| alpha7nAChR | C0051334 | 2 | 2 | 4 | 2 |
| DRD3 | 13490 | 1 |  | 4 |  |
| TrkB | 18212 |  |  | 4 |  |
| Mesencephalic astrocyte-derived neurotrophic factor | 7873 |  | 2 | 4 | 2 |
| CTX-MSN | 4478 | 1 | 3 | 4 | 1.33333333333333 |
| MSN | 4478 | 1 | 3 | 4 | 1.33333333333333 |
| MSN-MSN | 4478 | 1 | 3 | 4 | 1.33333333333333 |
| TA-MSN | 4478 | 1 | 3 | 4 | 1.33333333333333 |
| LV-PINK1 | 5728 | 4 | 2 | 4 | 2 |
| FOXO3 | 2309 |  | 1 | 4 | 4 |
| Na+/K+ -ATPase | C0001479 | 2 | 1 | 4 | 4 |
| hMANF) | 7873 |  | 2 | 4 | 2 |
| hMANF | 7873 |  | 2 | 4 | 2 |
| torin | 7001 |  | 2 | 4 | 2 |
| anti-p62 antibodies | C1624602 | 4 | 5 | 4 | 0.8 |
| CD200-CD200R1 | 4345 |  | 1 | 4 | 4 |
| SNAP25 | 6616 |  |  | 4 |  |
| PFC | 5199 |  | 2 | 4 | 2 |
| specific antibody-antigen | C0443640 | 7 | 5 | 4 | 0.8 |
| IL-13 | 3596 |  | 1 | 4 | 4 |
| Phospholipase A2, group VI | 8398 | 2 | 4 | 4 | 1 |
| L-type voltage-gated calcium channels | C0288263 |  | 2 | 4 | 2 |
| hMfn2 | 9927 | 2 | 3 | 4 | 1.33333333333333 |
| GIP | C0017132 |  | 1 | 4 | 4 |
| incretins | C1562292 |  |  | 4 |  |
| CREB | 81646 |  |  | 4 |  |
| apoptotic-related proteins | C1564881 |  | 3 | 4 | 1.33333333333333 |
| SD 20.2 | C0019737 |  | 4 | 4 | 1 |
| Tf | 7018 | 2 | 3 | 4 | 1.33333333333333 |
| RAB7L1 gene | 8934 |  | 1 | 4 | 4 |
| APOE*epsilon4 allele | C3642141 | 2 | 1 | 4 | 4 |
| transforming growth factor (TGF)-beta1 | C1515406 |  | 1 | 4 | 4 |
| protein functions | C0815043 | 3 | 3 | 4 | 1.33333333333333 |
| C reactive protein | 1401 | 1 | 4 | 4 | 1 |
| membrane-protein | C0025252 |  | 1 | 4 | 4 |
| CDNF | 361276 |  |  | 4 |  |
| Hsc70-interacting protein ( | 6767 | 1 |  | 4 |  |
| NT-3 | C0083735 |  |  | 4 |  |
| Protein secondary structures | C0175168 | 5 | 9 | 4 | 0.444444444444444 |
| Incretin hormones | C0017132 |  | 1 | 4 | 4 |
| GLP-1 receptor | 2740 |  |  | 4 |  |
| Interleukin-4 | 3565 | 1 | 1 | 4 | 4 |
| Interleukin 4 | 16189 |  | 1 | 4 | 4 |
| IL4 | 16189 |  | 1 | 4 | 4 |
| Hsc70-interacting protein | 6767 | 1 |  | 4 |  |
| genetic organization | C1517495 | 3 | 1 | 4 | 4 |
| anti-EPOR antibodies | C1624602 | 4 | 5 | 4 | 0.8 |
| P2X7R | 5027 |  | 1 | 4 | 4 |
| JAK2-STAT3 | 3717 |  |  | 4 |  |
| GVI | 8398 | 2 | 4 | 4 | 1 |
| SD 13.2 | C0019737 |  | 4 | 4 | 1 |
| GPER | 2852 | 2 | 5 | 4 | 0.8 |
| EC 3.1 | C0030016 | 3 | 6 | 4 | 0.666666666666667 |
| Tropomyosin receptor kinase | C0077397 |  |  | 4 |  |
| NLRP3 | 216799 |  |  | 4 |  |
| Na+/K+-ATPase | C0001479 | 2 | 1 | 4 | 4 |
| interleukin (IL)1beta | C0021753 | 4 | 6 | 4 | 0.666666666666667 |
| neurotrophin-3 | C0083735 |  |  | 4 |  |
| TF-regulated gene | 7018 | 2 | 3 | 4 | 1.33333333333333 |
| genetic feature | C1517495 | 3 | 1 | 4 | 4 |
| rhTFAM | 7019 | 1 | 4 | 4 | 1 |
| SD 9.2 | C0019737 |  | 4 | 4 | 1 |
| LTCCs | C0288263 |  | 2 | 4 | 2 |
| ionized calcium-binding adapter molecule 1 | 199 |  | 2 | 4 | 2 |
| Nuclear factor erythroid 2-like 2 | 4780 |  | 5 | 4 | 0.8 |
| SOCE | 760 | 3 | 1 | 4 | 4 |
| interleukin-2 | 3558 | 1 |  | 4 |  |
| alpha7 nicotinic acetylcholine receptors | C0051334 | 2 | 2 | 4 | 2 |
| APOE epsilon4 risky allele | C3642141 | 2 | 1 | 4 | 4 |
| IL-1beta | C0021753 | 4 | 6 | 4 | 0.666666666666667 |
| anti-Toxoplasma antibodies | C1624602 | 4 | 5 | 4 | 0.8 |
| allergic rhinitis | 3596 |  | 1 | 4 | 4 |
| JAK2 | 3717 |  |  | 4 |  |
| glutamate transporter 1 | 6506 |  | 2 | 4 | 2 |
| Peroxisome proliferator-activated receptor gamma | C0166417 | 1 | 3 | 4 | 1.33333333333333 |
| Cathepsin L | 1514 | 2 |  | 4 |  |
| CtsL | 1514 | 2 |  | 4 |  |
| LAMP2 gene | 3920 |  | 3 | 4 | 1.33333333333333 |
| neurotrophins 3 | C0083735 |  |  | 4 |  |
| SNAP | 6616 |  |  | 4 |  |
| incretin | C1562292 |  |  | 4 |  |
| surface protein | C0025252 |  | 1 | 4 | 4 |
| peroxisome proliferator-activated receptor gamma coactivator | C0166417 | 1 | 3 | 4 | 1.33333333333333 |
| VCAM1 | 7412 |  | 1 | 4 | 4 |
| Peroxiredoxin-2 | 7001 |  | 2 | 4 | 2 |
| fusion-fission protein | C0162768 | 11 | 8 | 4 | 0.5 |
| AC-PC | 5420 |  |  | 4 |  |
| IRE1alpha | 2081 |  |  | 4 |  |
| PODXL | 5420 |  |  | 4 |  |
| human leukocyte-antigen | C0019629 |  | 2 | 4 | 2 |
| RAB29 | 8934 |  | 1 | 4 | 4 |
| intrinsically disordered alpha-synuclein proteins | C3658247 | 6 | 3 | 4 | 1.33333333333333 |
| p-ERK protein | C0082731 |  |  | 4 |  |
| SD 12.2 | C0019737 |  | 4 | 4 | 1 |
| hydrolase | C0020289 | 3 | 2 | 4 | 2 |
| ionized calcium binding adapter molecule 1 | 199 |  | 2 | 4 | 2 |
| methylenetetrahydrofolate reductase | 4524 |  | 2 | 4 | 2 |
| GLP-1) receptor | 2740 |  |  | 4 |  |
| Dynamin-Related Protein-1 | 10059 | 1 | 8 | 4 | 0.5 |
| DNM1L | 10059 | 1 | 8 | 4 | 0.5 |
| Nuclear factor (erythroid-derived 2)-like 2 | 4780 |  | 5 | 4 | 0.8 |
| membrane-associated estrogen receptor | 2852 | 2 | 5 | 4 | 0.8 |
| peroxiredoxin2 | 7001 |  | 2 | 4 | 2 |
| GRN gene | 2896 | 3 | 2 | 4 | 2 |
| glutamate transporter-1 | 6506 |  | 2 | 4 | 2 |
| gamma-Synucleins | C0753208 | 4 | 3 | 4 | 1.33333333333333 |
| LAMP 2 | 3920 |  | 3 | 4 | 1.33333333333333 |
| mitogenactivated protein kinase 14 | 26416 | 2 | 2 | 4 | 2 |
| apoptosis related proteins | C1564881 |  | 3 | 4 | 1.33333333333333 |
| Conserved dopamine neurotrophic factor | 361276 |  |  | 4 |  |
| p-GSK-3beta protein | C0082731 |  |  | 4 |  |
| VPS13C gene | 54832 |  |  | 4 |  |
| ATPase Na+/K+ | C0001479 | 2 | 1 | 4 | 4 |
| MTHFR gene | 4524 |  | 2 | 4 | 2 |
| CAT) | 847 | 3 | 3 | 4 | 1.33333333333333 |
| Tubulin polymerization promoting proteins | 11076 | 2 | 1 | 4 | 4 |
| TPPPs | 11076 | 2 | 1 | 4 | 4 |
| Jak 1/2 | 3717 |  |  | 4 |  |
| LAMP2 protein | 3920 |  | 3 | 4 | 1.33333333333333 |
| LAMP2 proteins | 3920 |  | 3 | 4 | 1.33333333333333 |
| LAMP2A | C0025252 |  | 1 | 4 | 4 |
| neurotrophic derived factor | C0083735 |  |  | 4 |  |
| granulin | 2896 | 3 | 2 | 4 | 2 |
| TSA | 7001 |  | 2 | 4 | 2 |
| trans-acting splicing factors | C0040627 | 2 | 2 | 4 | 2 |
| Interleukin 4 | 3565 | 1 | 1 | 4 | 4 |
| methylenetetrahydrofolate reductase (MTHFR) gene | 4524 |  | 2 | 4 | 2 |
| p)AMPK | C0082731 |  |  | 4 |  |
| CHCHD10-CHCHD2 | 400916 |  |  | 4 |  |
| Rab5A | 5868 |  | 1 | 4 | 4 |
| MP function | C0815043 | 3 | 3 | 4 | 1.33333333333333 |
| Cu/Zn superoxide dismutase | C0010461 | 2 |  | 3 |  |
| superoxide dismutase 1 | C0010461 | 2 |  | 3 |  |
| and 1 | 11169 | 2 | 1 | 3 | 3 |
| acetylcholinesterase | 83817 | 2 | 1 | 3 | 3 |
| AChE | 83817 | 2 | 1 | 3 | 3 |
| MPP( | 4359 | 6 | 8 | 3 | 0.375 |
| MPP(+ | 4359 | 6 | 8 | 3 | 0.375 |
| Hsp90 | 3320 | 8 | 5 | 3 | 0.6 |
| metabotropic glutamate receptors | C0206529 | 8 | 10 | 3 | 0.3 |
| mGluR2/3 | C0206529 | 8 | 10 | 3 | 0.3 |
| metabotropic glutamate receptor | C0206529 | 8 | 10 | 3 | 0.3 |
| mGluR | C0206529 | 8 | 10 | 3 | 0.3 |
| mGluRs | C0206529 | 8 | 10 | 3 | 0.3 |
| metabotropic glutamate mGlu5 receptors | C0206529 | 8 | 10 | 3 | 0.3 |
| metabotropic glutamate (mGlu) receptors | C0206529 | 8 | 10 | 3 | 0.3 |
| mGlu receptor | C0206529 | 8 | 10 | 3 | 0.3 |
| mGlu receptors | C0206529 | 8 | 10 | 3 | 0.3 |
| Fos protein | 2353 | 5 | 3 | 3 | 1 |
| Fos | 2353 | 5 | 3 | 3 | 1 |
| cFos | 2353 | 5 | 3 | 3 | 1 |
| Fos(+ | 2353 | 5 | 3 | 3 | 1 |
| Fos- | 2353 | 5 | 3 | 3 | 1 |
| FLI | 2353 | 5 | 3 | 3 | 1 |
| S100beta | 6285 | 2 | 3 | 3 | 1 |
| S100beta protein | 6285 | 2 | 3 | 3 | 1 |
| S100 beta protein | 6285 | 2 | 3 | 3 | 1 |
| 5-HT(1A) receptors | C0379900 | 5 | 11 | 3 | 0.272727272727273 |
| 5-HT(1A) receptor | C0379900 | 5 | 11 | 3 | 0.272727272727273 |
| Serotonin 1A receptor | C0379900 | 5 | 11 | 3 | 0.272727272727273 |
| 5-HT1A receptors | C0379900 | 5 | 11 | 3 | 0.272727272727273 |
| Serotonin 1A receptors | C0379900 | 5 | 11 | 3 | 0.272727272727273 |
| vesicular monoamine transporters | C1529240 | 7 | 2 | 3 | 1.5 |
| vesicular monoamine transporter | C1529240 | 7 | 2 | 3 | 1.5 |
| VMAT | C1529240 | 7 | 2 | 3 | 1.5 |
| glutathione S-transferase | C0017837 | 2 | 6 | 3 | 0.5 |
| GST-DJ-1 | C0017837 | 2 | 6 | 3 | 0.5 |
| GSH-ST | C0017837 | 2 | 6 | 3 | 0.5 |
| PI3K | C0031727 | 4 | 11 | 3 | 0.272727272727273 |
| kinase- | C0031727 | 4 | 11 | 3 | 0.272727272727273 |
| KD | C0031727 | 4 | 11 | 3 | 0.272727272727273 |
| Hsp70 | C0243043 | 6 | 4 | 3 | 0.75 |
| heat-shock protein 70 | C0243043 | 6 | 4 | 3 | 0.75 |
| heat shock protein 70 | C0243043 | 6 | 4 | 3 | 0.75 |
| VMAT2 | 25549 | 6 | 3 | 3 | 1 |
| VMAT(2) | 25549 | 6 | 3 | 3 | 1 |
| VMAT-2 | 25549 | 6 | 3 | 3 | 1 |
| deoxy | C0069851 | 2 | 5 | 3 | 0.6 |
| islet amyloid polypeptide | C0063684 | 1 | 9 | 3 | 0.333333333333333 |
| LRRK2 | 42447 | 4 | 9 | 3 | 0.333333333333333 |
| dLRRK | 42447 | 4 | 9 | 3 | 0.333333333333333 |
| LRRK | 42447 | 4 | 9 | 3 | 0.333333333333333 |
| FosB | 100360880 | 2 |  | 3 |  |
| TDP-43 | 23435 | 5 | 7 | 3 | 0.428571428571429 |
| DNA-binding protein | 23435 | 5 | 7 | 3 | 0.428571428571429 |
| phenylalanine | C0031453 | 4 | 3 | 3 | 1 |
| calpain | C0006784 | 4 | 2 | 3 | 1.5 |
| reporter genes | C0206414 | 4 | 2 | 3 | 1.5 |
| reporter gene | C0206414 | 4 | 2 | 3 | 1.5 |
| IGF-1 | 3479 | 4 | 1 | 3 | 3 |
| insulin-like growth factor-1 | 3479 | 4 | 1 | 3 | 3 |
| IGF-I | 3479 | 4 | 1 | 3 | 3 |
| Hsc70 | 3312 | 4 | 4 | 3 | 0.75 |
| P-glycoprotein | 5243 | 4 | 2 | 3 | 1.5 |
| MDR1 | 5243 | 4 | 2 | 3 | 1.5 |
| ABCB1 | 5243 | 4 | 2 | 3 | 1.5 |
| multi-drug resistance 1 | 5243 | 4 | 2 | 3 | 1.5 |
| HIF-1 alpha | 3091 | 3 | 1 | 3 | 3 |
| HIF-1alpha | 3091 | 3 | 1 | 3 | 3 |
| ataxin-3 | 4287 | 3 | 6 | 3 | 0.5 |
| SCA3 | 4287 | 3 | 6 | 3 | 0.5 |
| SCA-3 | 4287 | 3 | 6 | 3 | 0.5 |
| ATXN3 | 4287 | 3 | 6 | 3 | 0.5 |
| adenosine A1 receptors | C0001451 |  |  | 3 |  |
| PHDs | 5132 | 2 | 1 | 3 | 3 |
| PDC | 5132 | 2 | 1 | 3 | 3 |
| PhD | 5132 | 2 | 1 | 3 | 3 |
| collagen | C0009325 | 4 | 2 | 3 | 1.5 |
| albumin | C0001924 | 1 | 6 | 3 | 0.5 |
| TSPO | 706 | 1 | 1 | 3 | 3 |
| ATPase | C0001473 | 1 | 6 | 3 | 0.5 |
| genetic component | C0599770 | 1 | 5 | 3 | 0.6 |
| SCA2 | 6311 | 1 | 5 | 3 | 0.6 |
| ataxin-2 | 6311 | 1 | 5 | 3 | 0.6 |
| Rab | 3267 | 3 | 1 | 3 | 3 |
| Rabs | 3267 | 3 | 1 | 3 | 3 |
| prolactin | 5617 | 1 | 2 | 3 | 1.5 |
| apoptosis-inducing factor | C0763396 | 3 | 1 | 3 | 3 |
| leucine-rich repeat kinase 2 | 300160 | 3 | 3 | 3 | 1 |
| LRRK2 | 300160 | 3 | 3 | 3 | 1 |
| Thy1 | 7070 | 2 | 5 | 3 | 0.6 |
| Thy-1 | 7070 | 2 | 5 | 3 | 0.6 |
| sodium channel | C0037492 | 1 | 5 | 3 | 0.6 |
| complexes I-IV | C0010760 | 3 | 5 | 3 | 0.6 |
| complex IV | C0010760 | 3 | 5 | 3 | 0.6 |
| cytochrome oxidase | C0010760 | 3 | 5 | 3 | 0.6 |
| functional component | C1179435 | 3 | 2 | 3 | 1.5 |
| protein elements | C1179435 | 3 | 2 | 3 | 1.5 |
| protein component | C1179435 | 3 | 2 | 3 | 1.5 |
| decreased glutathione | C0034917 | 1 | 1 | 3 | 3 |
| HTR2A | 3356 | 1 | 4 | 3 | 0.75 |
| PPAR | 5465 | 3 | 1 | 3 | 3 |
| PPARalpha | 5465 | 3 | 1 | 3 | 3 |
| choline acetyltransferase | C0008407 | 2 | 4 | 3 | 0.75 |
| ChAT | C0008407 | 2 | 4 | 3 | 0.75 |
| neuronal nitric oxide synthase | C0669368 | 1 | 4 | 3 | 0.75 |
| neuronal NO synthase | C0669368 | 1 | 4 | 3 | 0.75 |
| histidine | C0019602 | 3 | 1 | 3 | 3 |
| beta3 | 27319 | 3 | 1 | 3 | 3 |
| vasoactive intestinal peptide | C0042395 |  | 4 | 3 | 0.75 |
| Presynaptic nicotinic receptors | C0206129 | 1 | 1 | 3 | 3 |
| Presynaptic receptors | C0206129 | 1 | 1 | 3 | 3 |
| presynaptic histamine receptors | C0206129 | 1 | 1 | 3 | 3 |
| IAPP | 3375 | 2 | 4 | 3 | 0.75 |
| hIAPP | 3375 | 2 | 4 | 3 | 0.75 |
| amylin | 3375 | 2 | 4 | 3 | 0.75 |
| Lmx1a | 4009 | 1 | 3 | 3 | 1 |
| human genome | C0017429 | 2 | 4 | 3 | 0.75 |
| link protein | C0065011 | 1 | 4 | 3 | 0.75 |
| NIR | 26155 | 1 | 4 | 3 | 0.75 |
| MEG | 5775 | 2 | 4 | 3 | 0.75 |
| Gpr37 | 2861 | 2 | 2 | 3 | 1.5 |
| parkin-associated endothelin receptor-like | 2861 | 2 | 2 | 3 | 1.5 |
| DARPP-32 | 360616 | 2 | 3 | 3 | 1 |
| FBXO7 | 25793 | 1 | 3 | 3 | 1 |
| pentraxin 1 | C0006560 | 2 | 3 | 3 | 1 |
| CRP | C0006560 | 2 | 3 | 3 | 1 |
| AT II | C0003009 | 2 | 1 | 3 | 3 |
| AII | C0003009 | 2 | 1 | 3 | 3 |
| Pick's | 9463 | 1 | 2 | 3 | 1.5 |
| TGF-beta1 | 7040 | 2 | 2 | 3 | 1.5 |
| TGF-beta | 7040 | 2 | 2 | 3 | 1.5 |
| Transforming growth factor beta | 7040 | 2 | 2 | 3 | 1.5 |
| sirtuins | C1136177 | 1 | 3 | 3 | 1 |
| potassium channels | C0032824 | 2 | 3 | 3 | 1 |
| dopamine D3 receptor | C0082341 | 2 | 3 | 3 | 1 |
| dopamine D2/D3 receptor | C0082341 | 2 | 3 | 3 | 1 |
| dopamine D3 receptors | C0082341 | 2 | 3 | 3 | 1 |
| G-protein | C0086376 | 2 | 3 | 3 | 1 |
| Divalent metal transporter 1 | C0288148 | 1 | 3 | 3 | 1 |
| dopamine D1 receptor | 1812 | 1 | 3 | 3 | 1 |
| Drd1a | 1812 | 1 | 3 | 3 | 1 |
| RAB3A | 5864 | 1 |  | 3 |  |
| repressor | C1336789 | 2 | 1 | 3 | 3 |
| transcriptional repressor | C1336789 | 2 | 1 | 3 | 3 |
| TLR4 | 21898 | 1 | 3 | 3 | 1 |
| STAT | 6646 | 1 |  | 3 |  |
| bone morphogenetic protein | C0053932 | 2 |  | 3 |  |
| BMP | C0053932 | 2 |  | 3 |  |
| HCN | 378938 | 1 | 1 | 3 | 3 |
| calmodulin | C0006772 | 2 |  | 3 |  |
| ApEn | 328 | 1 | 1 | 3 | 3 |
| Mol | 90527 | 2 | 2 | 3 | 1.5 |
| mole | 90527 | 2 | 2 | 3 | 1.5 |
| tumor suppressor | C0079427 | 1 | 1 | 3 | 3 |
| T-type calcium channels | C0752118 | 1 |  | 3 |  |
| T-type calcium channel | C0752118 | 1 |  | 3 |  |
| Fis1p | 51024 | 1 | 2 | 3 | 1.5 |
| ERK | 24338 | 1 | 3 | 3 | 1 |
| Cathepsin D | 1509 | 2 |  | 3 |  |
| bis[ | 9531 | 1 | 1 | 3 | 3 |
| GCSF | 1440 |  |  | 3 |  |
| G-CSF | 1440 |  |  | 3 |  |
| lipoprotein | C0023820 | 2 |  | 3 |  |
| lipoproteins | C0023820 | 2 |  | 3 |  |
| PLP) | 5354 | 1 | 2 | 3 | 1.5 |
| DRD3 | 1814 | 1 | 2 | 3 | 1.5 |
| beta-arrestin | C0167464 | 1 |  | 3 |  |
| Synaptophysin | 20977 | 1 | 1 | 3 | 3 |
| SWT | 219793 | 1 |  | 3 |  |
| peptidase | C0030940 | 1 | 1 | 3 | 3 |
| metalloproteases | C0025543 |  | 1 | 3 | 3 |
| ERK2 | 5594 | 1 | 1 | 3 | 3 |
| PACRG gene product | C0751455 | 1 | 1 | 3 | 3 |
| VIP | 7432 |  | 1 | 3 | 3 |
| histones | C0019652 | 1 | 1 | 3 | 3 |
| kit | 3815 | 1 |  | 3 |  |
| antiporter | C0052088 | 1 | 1 | 3 | 3 |
| precursor protein | C0033665 | 1 | 1 | 3 | 3 |
| gamma-secretases | C0379528 |  | 1 | 3 | 3 |
| Grp78 | 25617 | 1 | 1 | 3 | 3 |
| DCX | 13193 | 1 |  | 3 |  |
| Thioredoxin | C0039938 | 1 | 2 | 3 | 1.5 |
| Trx | C0039938 | 1 | 2 | 3 | 1.5 |
| MRIs | 78996 | 1 |  | 3 |  |
| held | 8289 |  |  | 3 |  |
| clathrin | C0008905 | 1 |  | 3 |  |
| ApoA-I | 335 | 1 | 1 | 3 | 3 |
| eIF2 alpha | 83939 | 1 | 2 | 3 | 1.5 |
| catalase | 12359 | 1 | 2 | 3 | 1.5 |
| 14-3-3 proteins | C0090388 | 1 | 1 | 3 | 3 |
| prostate specific antigen | 354 | 1 | 2 | 3 | 1.5 |
| APs | 354 | 1 | 2 | 3 | 1.5 |
| gp91PHOX | 13058 | 1 | 2 | 3 | 1.5 |
| Hrs | 1822 | 1 | 2 | 3 | 1.5 |
| gp130 | 3572 |  |  | 3 |  |
| ghrelin's receptor | C0391690 | 1 | 1 | 3 | 3 |
| growth hormone secretagogue receptor (GHSR)- | C0391690 | 1 | 1 | 3 | 3 |
| peptide fragments | C0030935 | 1 | 2 | 3 | 1.5 |
| peptide fragment | C0030935 | 1 | 2 | 3 | 1.5 |
| P-glycoprotein | C0069906 | 1 | 1 | 3 | 3 |
| glutathione peroxidase 1 | 2876 | 1 |  | 3 |  |
| VCP | 7415 |  | 1 | 3 | 3 |
| Msx1 | 17701 | 1 |  | 3 |  |
| A beta | 8803 | 1 | 1 | 3 | 3 |
| tight junction proteins | C3494363 | 1 |  | 3 |  |
| BoNT/A | C1295507 |  | 2 | 3 | 1.5 |
| blood plasma protein | C0032120 | 1 | 1 | 3 | 3 |
| Tyrosine hydroxylase | 21823 | 1 | 2 | 3 | 1.5 |
| Kennedy disease | 367 |  | 1 | 3 | 3 |
| apolipoprotein | C0003591 | 1 |  | 3 |  |
| DMT1 | 18174 | 1 | 1 | 3 | 3 |
| CBS | 875 | 1 | 2 | 3 | 1.5 |
| NAA | C0085845 | 1 | 1 | 3 | 3 |
| CD | 1509 | 2 |  | 3 |  |
| proteinases | C0030940 | 1 | 1 | 3 | 3 |
| peripheral benzodiazepine receptor | 706 | 1 | 1 | 3 | 3 |
| peripheral benzodiazepine receptors | 706 | 1 | 1 | 3 | 3 |
| PBR | 706 | 1 | 1 | 3 | 3 |
| cystatin C | 1471 | 1 | 1 | 3 | 3 |
| hypoxia-inducible factor-1alpha | 3091 | 3 | 1 | 3 | 3 |
| Pick | 9463 | 1 | 2 | 3 | 1.5 |
| 5-HT2A receptor | 3356 | 1 | 4 | 3 | 0.75 |
| 5-HT(2A) receptor | 3356 | 1 | 4 | 3 | 0.75 |
| Paraoxonase-1 | 5444 |  | 3 | 3 | 1 |
| PON1 | 5444 |  | 3 | 3 | 1 |
| Bcl-2 protein | C0597712 | 1 | 1 | 3 | 3 |
| cannabinoid receptor 1 | 1268 |  | 8 | 3 | 0.375 |
| CNR1 | 1268 |  | 8 | 3 | 0.375 |
| CB1 | 1268 |  | 8 | 3 | 0.375 |
| cyclic-AMP response element binding protein | C0056695 |  | 1 | 3 | 3 |
| FBX07 | 25793 | 1 | 3 | 3 | 1 |
| PKPS | 25793 | 1 | 3 | 3 | 1 |
| FBXO7 gene | 25793 | 1 | 3 | 3 | 1 |
| nNOS | C0669368 | 1 | 4 | 3 | 0.75 |
| BAG3 | 9531 | 1 | 1 | 3 | 3 |
| Valosin-containing protein | 7415 |  | 1 | 3 | 3 |
| nuclear respiratory factor-1 | 4899 |  | 2 | 3 | 1.5 |
| NRF-1 | 4899 |  | 2 | 3 | 1.5 |
| microtubule-associated protein 1 light chain 3 (LC3)-II | C3642279 |  | 3 | 3 | 1 |
| ATXN2 | 6311 | 1 | 5 | 3 | 0.6 |
| serotonin 2A receptor gene | 3356 | 1 | 4 | 3 | 0.75 |
| HTR2A c. | 3356 | 1 | 4 | 3 | 0.75 |
| RXR | 6256 |  | 1 | 3 | 3 |
| calcium-activated proteases | C0006784 | 4 | 2 | 3 | 1.5 |
| calpains | C0006784 | 4 | 2 | 3 | 1.5 |
| PD-c | 5132 | 2 | 1 | 3 | 3 |
| PGC-1alpha | 19017 |  | 5 | 3 | 0.6 |
| MJD | 4287 | 3 | 6 | 3 | 0.5 |
| protein components | C1179435 | 3 | 2 | 3 | 1.5 |
| SNCA | 29219 |  | 1 | 3 | 3 |
| histone deacetylase | C0019643 |  | 4 | 3 | 0.75 |
| SWI | 6594 |  | 3 | 3 | 1 |
| hint | 3094 |  | 2 | 3 | 1.5 |
| potassium channel | C0032824 | 2 | 3 | 3 | 1 |
| cyclooxygenase 2 | 29527 |  | 3 | 3 | 1 |
| Nox2 | 13058 | 1 | 2 | 3 | 1.5 |
| PPARgamma co-activator-1alpha | 19017 |  | 5 | 3 | 0.6 |
| glutamate-glutamine | C1718801 |  | 1 | 3 | 3 |
| apolipoprotein A-I | 335 | 1 | 1 | 3 | 3 |
| c-Fos | 2353 | 5 | 3 | 3 | 1 |
| glyceraldehyde-3-phosphate dehydrogenase | 2597 |  | 2 | 3 | 1.5 |
| GluR1 | 14799 |  | 3 | 3 | 1 |
| CB1Rs | 1268 |  | 8 | 3 | 0.375 |
| bone morphogenetic proteins | C0053932 | 2 |  | 3 |  |
| PARK15 | 25793 | 1 | 3 | 3 | 1 |
| CNR | 1268 |  | 8 | 3 | 0.375 |
| Fis1 | 51024 | 1 | 2 | 3 | 1.5 |
| FGA | 2243 |  | 2 | 3 | 1.5 |
| PLP | 5354 | 1 | 2 | 3 | 1.5 |
| islet amyloid polypeptides | C0063684 | 1 | 9 | 3 | 0.333333333333333 |
| rhodopsin | 6010 |  | 2 | 3 | 1.5 |
| GSTO | C0017837 | 2 | 6 | 3 | 0.5 |
| ERK | C0031727 | 4 | 11 | 3 | 0.272727272727273 |
| APE1 | 328 | 1 | 1 | 3 | 3 |
| Pael-R | 2861 | 2 | 2 | 3 | 1.5 |
| proteolytic enzymes | C0030940 | 1 | 1 | 3 | 3 |
| mAb | C0003250 |  | 1 | 3 | 3 |
| NMS | 129521 |  | 1 | 3 | 3 |
| granulocyte-colony stimulating factor | 1440 |  |  | 3 |  |
| ATPases | C0001473 | 1 | 6 | 3 | 0.5 |
| muM | 56925 |  | 5 | 3 | 0.6 |
| h-IAPP | C0063684 | 1 | 9 | 3 | 0.333333333333333 |
| hypoxia-inducible factor 1alpha | 3091 | 3 | 1 | 3 | 3 |
| LC3 II | C3642279 |  | 3 | 3 | 1 |
| toll-like receptor 4 | 29260 |  | 1 | 3 | 3 |
| TLR4 | 29260 |  | 1 | 3 | 3 |
| dopamine D(1) receptor | 1812 | 1 | 3 | 3 | 1 |
| C-reactive protein | C0006560 | 2 | 3 | 3 | 1 |
| histone deacetylases | C0019643 |  | 4 | 3 | 0.75 |
| TAR DNA binding protein 43 | 23435 | 5 | 7 | 3 | 0.428571428571429 |
| thioredoxins | C0039938 | 1 | 2 | 3 | 1.5 |
| sodium channels | C0037492 | 1 | 5 | 3 | 0.6 |
| eukaryotic initiation factor 2alpha | C0013733 |  | 2 | 3 | 1.5 |
| GLP-1 receptor | 14652 |  | 1 | 3 | 3 |
| TGF-beta(1) | 7040 | 2 | 2 | 3 | 1.5 |
| TGF-beta(1 | 7040 | 2 | 2 | 3 | 1.5 |
| metalloproteinases | C0025543 |  | 1 | 3 | 3 |
| hMiro1 | 55288 |  | 2 | 3 | 1.5 |
| TARDBP gene | 23435 | 5 | 7 | 3 | 0.428571428571429 |
| TAR)-DNA-binding protein 43 | 23435 | 5 | 7 | 3 | 0.428571428571429 |
| TDP43 | 23435 | 5 | 7 | 3 | 0.428571428571429 |
| eIF2alpha | 83939 | 1 | 2 | 3 | 1.5 |
| iPLEX | C1697533 |  | 4 | 3 | 0.75 |
| OGT | 8473 |  | 2 | 3 | 1.5 |
| Kennedy's disease | 367 |  | 1 | 3 | 3 |
| 5-HT(2A | 3356 | 1 | 4 | 3 | 0.75 |
| CB(1)R | 1268 |  | 8 | 3 | 0.375 |
| BiP | 25617 | 1 | 1 | 3 | 3 |
| SYT11 | 23208 |  | 2 | 3 | 1.5 |
| genetic components | C0599770 | 1 | 5 | 3 | 0.6 |
| sirtuin 2 | 64383 |  | 2 | 3 | 1.5 |
| SIRT2 | 64383 |  | 2 | 3 | 1.5 |
| DARPP32 | 360616 | 2 | 3 | 3 | 1 |
| RIT2 | 6014 |  | 2 | 3 | 1.5 |
| TDP)-43 | 23435 | 5 | 7 | 3 | 0.428571428571429 |
| gamma-secretase | C0379528 |  | 1 | 3 | 3 |
| Hsc70 protein | 3312 | 4 | 4 | 3 | 0.75 |
| TAR DNA-binding protein | 23435 | 5 | 7 | 3 | 0.428571428571429 |
| AIF | C0763396 | 3 | 1 | 3 | 3 |
| lysozyme | C0026794 |  | 1 | 3 | 3 |
| MIRO1 | 55288 |  | 2 | 3 | 1.5 |
| RHOT1 | 55288 |  | 2 | 3 | 1.5 |
| Rho | 6010 |  | 2 | 3 | 1.5 |
| KLK3 | 354 | 1 | 2 | 3 | 1.5 |
| PSA | 354 | 1 | 2 | 3 | 1.5 |
| glutathione-S-transferase | C0017837 | 2 | 6 | 3 | 0.5 |
| Paraoxonase 1 | 5444 |  | 3 | 3 | 1 |
| PON 1 | 5444 |  | 3 | 3 | 1 |
| tumour suppressor | C0079427 | 1 | 1 | 3 | 3 |
| TARDBP | 23435 | 5 | 7 | 3 | 0.428571428571429 |
| repressors | C1336789 | 2 | 1 | 3 | 3 |
| GluA1 | 14799 |  | 3 | 3 | 1 |
| angiotensin II | C0003009 | 2 | 1 | 3 | 3 |
| eukaryotic initiation factor-2alpha | C0013733 |  | 2 | 3 | 1.5 |
| cystathionine beta-synthase | 875 | 1 | 2 | 3 | 1.5 |
| 5-HT1A-receptor | C0379900 | 5 | 11 | 3 | 0.272727272727273 |
| 5-HT1A-receptors | C0379900 | 5 | 11 | 3 | 0.272727272727273 |
| HMGB1 | 3146 |  |  | 3 |  |
| High mobility group box 1 | 3146 |  |  | 3 |  |
| dopamine D2/D3-receptor | C0082341 | 2 | 3 | 3 | 1 |
| vasoactive intestinal polypeptide | C0042395 |  | 4 | 3 | 0.75 |
| CB1/CB2 receptor | 1268 |  | 8 | 3 | 0.375 |
| CB2 | 12802 |  | 1 | 3 | 3 |
| peptidases | C0030940 | 1 | 1 | 3 | 3 |
| heat shock 70-kDa protein 8 | 3312 | 4 | 4 | 3 | 0.75 |
| NR2B | 14812 |  | 1 | 3 | 3 |
| ABCB1 gene | 5243 | 4 | 2 | 3 | 1.5 |
| cytochrome c oxidase | C0010760 | 3 | 5 | 3 | 0.6 |
| functional components | C1179435 | 3 | 2 | 3 | 1.5 |
| trkB | 25054 |  | 1 | 3 | 3 |
| SCH-22390 | 4771 |  | 1 | 3 | 3 |
| botulinum neurotoxin | C1295507 |  | 2 | 3 | 1.5 |
| BoNT-A | C1295507 |  | 2 | 3 | 1.5 |
| GPX1 | 24404 |  | 1 | 3 | 3 |
| TLR 4 | 21898 | 1 | 3 | 3 | 1 |
| pChAT | C0008407 | 2 | 4 | 3 | 0.75 |
| VIP- | C0042395 |  | 4 | 3 | 0.75 |
| Hsp70s | C0243043 | 6 | 4 | 3 | 0.75 |
| glutathione peroxidase-1 | 14775 |  | 1 | 3 | 3 |
| GPX-1 | 14775 |  | 1 | 3 | 3 |
| HLA-DRB1 | 3123 |  | 2 | 3 | 1.5 |
| HLA-DRB1 gene | 3123 |  | 2 | 3 | 1.5 |
| GCL | 2729 |  | 1 | 3 | 3 |
| plasma protein | C0032120 | 1 | 1 | 3 | 3 |
| linked protein | C0065011 | 1 | 4 | 3 | 0.75 |
| cyclooxygenase-2 | 29527 |  | 3 | 3 | 1 |
| median 2. | 11043 |  | 1 | 3 | 3 |
| NAA/Cr | C0085845 | 1 | 1 | 3 | 3 |
| AR | C0001924 | 1 | 6 | 3 | 0.5 |
| dopamine receptor (DR) D3 | 1814 | 1 | 2 | 3 | 1.5 |
| via | C3812285 |  | 1 | 3 | 3 |
| kinase family | C0031727 | 4 | 11 | 3 | 0.272727272727273 |
| LC3II | C3642279 |  | 3 | 3 | 1 |
| S100B | 6285 | 2 | 3 | 3 | 1 |
| S100B protein | 6285 | 2 | 3 | 3 | 1 |
| dopamine D2/D3 receptors | C0082341 | 2 | 3 | 3 | 1 |
| divalent metal transporter 1 | 18174 | 1 | 1 | 3 | 3 |
| PPARGC1A | 19017 |  | 5 | 3 | 0.6 |
| Glucagon-like peptide-1 | 2641 |  |  | 3 |  |
| adenosine triphosphatases | C0001473 | 1 | 6 | 3 | 0.5 |
| gene/protein | C0751455 | 1 | 1 | 3 | 3 |
| gene/proteins | C0751455 | 1 | 1 | 3 | 3 |
| DSBs | C0069851 | 2 | 5 | 3 | 0.6 |
| glutathione transferase | C0017837 | 2 | 6 | 3 | 0.5 |
| impacting | 55364 |  | 1 | 3 | 3 |
| MPP(+)) | 4359 | 6 | 8 | 3 | 0.375 |
| Dopamine receptor D3 | 1814 | 1 | 2 | 3 | 1.5 |
| Glutathione transferases | C0017837 | 2 | 6 | 3 | 0.5 |
| Nramp-dependent Fe(2 | C0288148 | 1 | 3 | 3 | 1 |
| tumor suppressors | C0079427 | 1 | 1 | 3 | 3 |
| PGC-1 alpha | 19017 |  | 5 | 3 | 0.6 |
| rho kinase | 6010 |  | 2 | 3 | 1.5 |
| presynaptic CB(1) receptors | C0206129 | 1 | 1 | 3 | 3 |
| TGFbeta | 7040 | 2 | 2 | 3 | 1.5 |
| O-GlcNAc | 8473 |  | 2 | 3 | 1.5 |
| Parkin-associated endothelin receptor-like receptor | 2861 | 2 | 2 | 3 | 1.5 |
| SWI) | 6594 |  | 3 | 3 | 1 |
| growth hormone secretagogue hormone receptor | C0391690 | 1 | 1 | 3 | 3 |
| G-protein-coupled receptor 37 | 2861 | 2 | 2 | 3 | 1.5 |
| PaelR | 2861 | 2 | 2 | 3 | 1.5 |
| Stat3 | 25125 |  | 1 | 3 | 3 |
| VGLUT2 | 140919 |  | 1 | 3 | 3 |
| linked toxic protein | C0065011 | 1 | 4 | 3 | 0.75 |
| NRF1 | 4899 |  | 2 | 3 | 1.5 |
| 5-Hydroxytryptamine 1A Receptors | C0379900 | 5 | 11 | 3 | 0.272727272727273 |
| 5-HT1A) receptors | C0379900 | 5 | 11 | 3 | 0.272727272727273 |
| 5-HT1A receptor | C0379900 | 5 | 11 | 3 | 0.272727272727273 |
| Lmx1a gene | 4009 | 1 | 3 | 3 | 1 |
| X-box-binding protein 1 | 289754 |  | 1 | 3 | 3 |
| glucagon | 2641 |  |  | 3 |  |
| HIF1alpha | 3091 | 3 | 1 | 3 | 3 |
| GPX1 | 14775 |  | 1 | 3 | 3 |
| Matrigel | C0065749 |  |  | 3 |  |
| Mtg | C0065749 |  |  | 3 |  |
| growth hormone secretagogue receptor | C0391690 | 1 | 1 | 3 | 3 |
| GHS-R1a | C0391690 | 1 | 1 | 3 | 3 |
| RIT2 gene | 6014 |  | 2 | 3 | 1.5 |
| monoclonal oligomer/protofibril selective alpha-synuclein antibodies | C0003250 |  | 1 | 3 | 3 |
| monoclonal alpha-synuclein antibodies | C0003250 |  | 1 | 3 | 3 |
| SYNJ1 | 8867 |  |  | 3 |  |
| microtubule-associated protein 1 light chain 3-II | C3642279 |  | 3 | 3 | 1 |
| Malat1 | 378938 | 1 | 1 | 3 | 3 |
| Metabotropic glutamate (mGlu) receptor | C0206529 | 8 | 10 | 3 | 0.3 |
| apurinic/apyrimidinic endonuclease 1 | 328 | 1 | 1 | 3 | 3 |
| glucose transporters isoform 1 | 6513 |  |  | 3 |  |
| GLUT | 6513 |  |  | 3 |  |
| ss1-42-amyloid peptide | 3123 |  | 2 | 3 | 1.5 |
| median 9.2 | 11043 |  | 1 | 3 | 3 |
| mGluR2/3 protein | C0206529 | 8 | 10 | 3 | 0.3 |
| GCLc | 2729 |  | 1 | 3 | 3 |
| mGluR1 | 14799 |  | 3 | 3 | 1 |
| bis- | 9531 | 1 | 1 | 3 | 3 |
| cyclic AMP response element-binding protein | C0056695 |  | 1 | 3 | 3 |
| PMd | 5354 | 1 | 2 | 3 | 1.5 |
| alpha ( SNCA ) gene | 378938 | 1 | 1 | 3 | 3 |
| serotonin 2a receptor | 3356 | 1 | 4 | 3 | 0.75 |
| 5-HT2A | 3356 | 1 | 4 | 3 | 0.75 |
| adenosine A1/A2A receptor | C0001451 |  |  | 3 |  |
| Transforming growth factor-beta1 | 7040 | 2 | 2 | 3 | 1.5 |
| Glutathione-S-transferases | C0017837 | 2 | 6 | 3 | 0.5 |
| GSTs | C0017837 | 2 | 6 | 3 | 0.5 |
| QCSP | 219793 | 1 |  | 3 |  |
| cysteine peptidases | C2717970 |  |  | 3 |  |
| metalloproteinase | C0025543 |  | 1 | 3 | 3 |
| Bcl-2 | 53585 |  |  | 3 |  |
| GAPDH | 2597 |  | 2 | 3 | 1.5 |
| P41 | 5594 | 1 | 1 | 3 | 3 |
| genes/proteins | C0751455 | 1 | 1 | 3 | 3 |
| Toll-like receptor 2 | 7097 |  |  | 3 |  |
| TLR2 | 7097 |  |  | 3 |  |
| presynaptic dopamine receptors | C0206129 | 1 | 1 | 3 | 3 |
| Ras-like without CAAX 2 | 6014 |  | 2 | 3 | 1.5 |
| endo/ | 79694 |  |  | 3 |  |
| GLUT1 | 6513 |  |  | 3 |  |
| SLC2A1 | 6513 |  |  | 3 |  |
| GIP | 14607 |  |  | 3 |  |
| Glucose dependent insulinotropic polypeptide | 14607 |  |  | 3 |  |
| CoQ 2 | 27235 |  |  | 3 |  |
| all' | 613 |  |  | 3 |  |
| TBSS | 219793 | 1 |  | 3 |  |
| presynaptic dopamine (DA) receptors | C0206129 | 1 | 1 | 3 | 3 |
| presynaptic D1 receptor | C0206129 | 1 | 1 | 3 | 3 |
| presynaptic dopamine receptor | C0206129 | 1 | 1 | 3 | 3 |
| Granulocyte Colony-Stimulating Factor | 1440 |  |  | 3 |  |
| Aldose reductase | 231 |  |  | 3 |  |
| AR | 231 |  |  | 3 |  |
| LC3B | 67443 |  |  | 3 |  |
| TLR-4 | 21898 | 1 | 3 | 3 | 1 |
| CD206 | 17533 |  |  | 3 |  |
| GP-EX | C3811116 |  |  | 3 |  |
| cyclic AMP-response element binding protein | C0056695 |  | 1 | 3 | 3 |
| GPR37-KO | 2861 | 2 | 2 | 3 | 1.5 |
| purinergic P2X7 receptors | C0386482 |  |  | 3 |  |
| P2X7R | C0386482 |  |  | 3 |  |
| STAT3 | 20848 |  |  | 3 |  |
| Hsc-70 | 3312 | 4 | 4 | 3 | 0.75 |
| Heat- Shock Protein-70 | C0243043 | 6 | 4 | 3 | 0.75 |
| Sirts | C1136177 | 1 | 3 | 3 | 1 |
| Mmp2 | 4313 |  |  | 3 |  |
| dopamine/D1 receptor | 1812 | 1 | 3 | 3 | 1 |
| voltage-activated calcium channels | C0814022 |  |  | 3 |  |
| dopamine receptor D1 | 1812 | 1 | 3 | 3 | 1 |
| trio | 7204 |  |  | 3 |  |
| HSPA8 | 3312 | 4 | 4 | 3 | 0.75 |
| all ' | 613 |  |  | 3 |  |
| translocator protein | 706 | 1 | 1 | 3 | 3 |
| SCH | 4771 |  | 1 | 3 | 3 |
| Fyn | 14360 |  |  | 3 |  |
| TAR-DNA binding protein | 23435 | 5 | 7 | 3 | 0.428571428571429 |
| toll like receptor 4 | 21898 | 1 | 3 | 3 | 1 |
| FOXO3 | 294515 |  |  | 3 |  |
| CRN | 10699 |  |  | 3 |  |
| MRgFUS | 78996 | 1 |  | 3 |  |
| Merlin | 4771 |  | 1 | 3 | 3 |
| XBP-1 | 289754 |  | 1 | 3 | 3 |
| median 2 | 11043 |  | 1 | 3 | 3 |
| gene protein | C0751455 | 1 | 1 | 3 | 3 |
| DRPLA | 1822 | 1 | 2 | 3 | 1.5 |
| Sirtuin-2 | 64383 |  | 2 | 3 | 1.5 |
| ApoA-1 | 335 | 1 | 1 | 3 | 3 |
| signal transducer and activator of transcription 3 | 20848 |  |  | 3 |  |
| 14-3-3 protein | C0090388 | 1 | 1 | 3 | 3 |
| gene 3 protein | C0751455 | 1 | 1 | 3 | 3 |
| HDACs | C0019643 |  | 4 | 3 | 0.75 |
| islet-amyloid polypeptide | C0063684 | 1 | 9 | 3 | 0.333333333333333 |
| capped | 1483 |  |  | 3 |  |
| IGF1 | 3479 | 4 | 1 | 3 | 3 |
| insulin-like growth factor 1 | 3479 | 4 | 1 | 3 | 3 |
| transcriptional repressors | C1336789 | 2 | 1 | 3 | 3 |
| ATG12 gene | 9140 |  |  | 3 |  |
| ATG12 | 9140 |  |  | 3 |  |
| Synaptojanin 1 | 8867 |  |  | 3 |  |
| #NAME? | C0814022 |  |  | 3 |  |
| Atx3 | 4287 | 3 | 6 | 3 | 0.5 |
| Soc | 91544 |  |  | 3 |  |
| CB1 cannabinoid receptor | 1268 |  | 8 | 3 | 0.375 |
| arginase-1 | 383 |  |  | 3 |  |
| Arg-1 | 383 |  |  | 3 |  |
| synaptotagmin XI | 23208 |  | 2 | 3 | 1.5 |
| Syt XI | 23208 |  | 2 | 3 | 1.5 |
| DEGs | 8560 |  |  | 3 |  |
| interleukin 6 signal transducer | 3572 |  |  | 3 |  |
| IL6ST | 3572 |  |  | 3 |  |
| interleukin (IL)-10 | C0085295 |  |  | 3 |  |
| ghrelin receptor | C0391690 | 1 | 1 | 3 | 3 |
| Matrix Metalloproteinase-2 | 4313 |  |  | 3 |  |
| MMP-2 | 4313 |  |  | 3 |  |
| glutathione peroxidase-1 | 2876 | 1 |  | 3 |  |
| GPX1 | 2876 | 1 |  | 3 |  |
| Gpx1 genes | 2876 | 1 |  | 3 |  |
| endo-/ | 79694 |  |  | 3 |  |
| glucose-dependent insulinotropic polypeptide | 14607 |  |  | 3 |  |
| GIP receptor | C0061126 |  |  | 3 |  |
| GLP-1R | 14652 |  | 1 | 3 | 3 |
| glutathione peroxidase 1 | 14775 |  | 1 | 3 | 3 |
| COQ2 | 27235 |  |  | 3 |  |
| hs-CRP | C0006560 | 2 | 3 | 3 | 1 |
| RXRalpha | 6256 |  | 1 | 3 | 3 |
| Retinoid X receptor alpha | 6256 |  | 1 | 3 | 3 |
| nuclear respiratory factor 1 | 4899 |  | 2 | 3 | 1.5 |
| Gad2 | 14417 |  |  | 3 |  |
| Pax6 | 18508 |  |  | 3 |  |
| gastric inhibitory polypeptide (GIP) receptor | C0061126 |  |  | 3 |  |
| GAD65 | 14417 |  |  | 3 |  |
| antagonist Brilliant Blue-G | C0381385 |  |  | 3 |  |
| DRB1 | 3123 |  | 2 | 3 | 1.5 |
| GPx-1 | 2876 | 1 |  | 3 |  |
| mAbs | C0003250 |  | 1 | 3 | 3 |
| Translocator Protein-18 kDa | 706 | 1 | 1 | 3 | 3 |
| S-100b | 6285 | 2 | 3 | 3 | 1 |
| TAR DNA-binding protein 43 | 23435 | 5 | 7 | 3 | 0.428571428571429 |
| H1c | 3006 |  |  | 3 |  |
| antagonist Brilliant Blue G | C0381385 |  |  | 3 |  |
| GLUT-1 | 6513 |  |  | 3 |  |
| FoxO3a | 294515 |  |  | 3 |  |
| PON-1 | 5444 |  | 3 | 3 | 1 |
| GP's | C3811116 |  |  | 3 |  |
| Rho-Kinase | 6010 |  | 2 | 3 | 1.5 |
| ROCK | 6010 |  | 2 | 3 | 1.5 |
| PRKAG2 | 51422 |  |  | 3 |  |
| PRKAG2 gene | 51422 |  |  | 3 |  |
| Histone de-acetylase | C0062773 |  |  | 3 |  |
| PD-NC | C0067895 |  |  | 3 |  |
| adenosine A1 receptor | C0001451 |  |  | 3 |  |
| A1R | C0001451 |  |  | 3 |  |
| Cu/Zn-superoxide dismutase | C0010461 | 2 |  | 3 |  |
| L-Phe | C0031453 | 4 | 3 | 3 | 1 |
| Por | 5447 |  |  | 3 |  |
| Ang II | C0003009 | 2 | 1 | 3 | 3 |
| Ferrireductase | C0243839 |  |  | 3 |  |
| APEX1 | 328 | 1 | 1 | 3 | 3 |
| CI 95% 1.5-2. | C0067503 |  |  | 3 |  |
| hS100B | 6285 | 2 | 3 | 3 | 1 |
| S100B gene | 6285 | 2 | 3 | 3 | 1 |
| Xbox binding protein 1 | 289754 |  | 1 | 3 | 3 |
| 5-HT1A) receptor | C0379900 | 5 | 11 | 3 | 0.272727272727273 |
| ADRsA1 | 231 |  |  | 3 |  |
| ADRsA2A | 231 |  |  | 3 |  |
| IL-6-signal transducer | 3572 |  |  | 3 |  |
| dopamine (DA) D2/D3 receptor | C0082341 | 2 | 3 | 3 | 1 |
| P2X7 receptors | C0386482 |  |  | 3 |  |
| PBT-2 | 3815 | 1 |  | 3 |  |
| MARKs | C0031727 | 4 | 11 | 3 | 0.272727272727273 |
| functional DNA elements | C1179435 | 3 | 2 | 3 | 1.5 |
| GSH-Px | 24404 |  | 1 | 3 | 3 |
| hinting | 3094 |  | 2 | 3 | 1.5 |
| FRA2 | 100360880 | 2 |  | 3 |  |
| linking protein | C0065011 | 1 | 4 | 3 | 0.75 |
| CORIN | 10699 |  |  | 3 |  |
| DRD1 | 1812 | 1 | 3 | 3 | 1 |
| presynaptic dopaminergic receptors | C0206129 | 1 | 1 | 3 | 3 |
| Miro-1 | 55288 |  | 2 | 3 | 1.5 |
| Bcl-2/Bax protein | C0597712 | 1 | 1 | 3 | 3 |
| cannabinoid receptors type 1 | 1268 |  | 8 | 3 | 0.375 |
| CB1R | 1268 |  | 8 | 3 | 0.375 |
| CT-MRI | 78996 | 1 |  | 3 |  |
| L-aspartate | C0085845 | 1 | 1 | 3 | 3 |
| PBT | 3815 | 1 |  | 3 |  |
| ETBR-LP-1 | 2861 | 2 | 2 | 3 | 1.5 |
| 5-HT 2A | 3356 | 1 | 4 | 3 | 0.75 |
| decreased brain reduced glutathione | C0034917 | 1 | 1 | 3 | 3 |
| CAPS | 1483 |  |  | 3 |  |
| ON-GP | C3811116 |  |  | 3 |  |
| androgen receptor | 367 |  | 1 | 3 | 3 |
| vACC | C0814022 |  |  | 3 |  |
| DMT1 | C0288148 | 1 | 3 | 3 | 1 |
| IGFI | 3479 | 4 | 1 | 3 | 3 |
| sOGT | 8473 |  | 2 | 3 | 1.5 |
| CB2R | 12802 |  | 1 | 3 | 3 |
| Synaptotagmin-11 | 23208 |  | 2 | 3 | 1.5 |
| H12 | 3006 |  |  | 3 |  |
| CI=-2. | C0067503 |  |  | 3 |  |
| Apolipoproteins | C0003591 | 1 |  | 3 |  |
| G-proteins | C0086376 | 2 | 3 | 3 | 1 |
| CatD | 1509 | 2 |  | 3 |  |
| PrL | 5617 | 1 | 2 | 3 | 1.5 |
| MPP+ ) | 4359 | 6 | 8 | 3 | 0.375 |
| histone acetyltransferase | C0062773 |  |  | 3 |  |
| VCP Gene | 7415 |  | 1 | 3 | 3 |
| AIs | 367 |  | 1 | 3 | 3 |
| 292DEGs | 8560 |  |  | 3 |  |
| Mrc1 | 17533 |  |  | 3 |  |
| arginase 1 | 383 |  |  | 3 |  |
| Cbs gene | 875 | 1 | 2 | 3 | 1.5 |
| GluN2B | 14812 |  | 1 | 3 | 3 |
| transcription repressor | C1336789 | 2 | 1 | 3 | 3 |
| neuronal nitric oxide synthases | C0669368 | 1 | 4 | 3 | 0.75 |
| cysteine proteases | C2717970 |  |  | 3 |  |
| BoNT | C1295507 |  | 2 | 3 | 1.5 |
| 5HT2A receptor | 3356 | 1 | 4 | 3 | 0.75 |
| XBP1 | 289754 |  | 1 | 3 | 3 |
| HL | C0026794 |  | 1 | 3 | 3 |
| eIF2alpha | C0013733 |  | 2 | 3 | 1.5 |
| Cnr2 | 12802 |  | 1 | 3 | 3 |
| angiotensin (Ang) II | C0003009 | 2 | 1 | 3 | 3 |
| amylin's | C0063684 | 1 | 9 | 3 | 0.333333333333333 |
| SYT11-GBA | 23208 |  | 2 | 3 | 1.5 |
| P2X7 receptor | C0386482 |  |  | 3 |  |
| plasma tau protein | C0032120 | 1 | 1 | 3 | 3 |
| Atg 12 | 9140 |  |  | 3 |  |
| Rho- | 6010 |  | 2 | 3 | 1.5 |
| CB1-CB2 | 1268 |  | 8 | 3 | 0.375 |
| Bcl-2 proteins | C0597712 | 1 | 1 | 3 | 3 |
| plasma proteins | C0032120 | 1 | 1 | 3 | 3 |
| G-coupled protein | C0086376 | 2 | 3 | 3 | 1 |
| peptide hydrolase | C0030940 | 1 | 1 | 3 | 3 |
| Rab10 | 10890 |  |  | 3 |  |
| cannabinoid receptor type 1 | 1268 |  | 8 | 3 | 0.375 |
| high-mobility group box 1 | 3146 |  |  | 3 |  |
| apoA1 | 335 | 1 | 1 | 3 | 3 |
| p-TDP-43 | 23435 | 5 | 7 | 3 | 0.428571428571429 |
| CTSD | 1509 | 2 |  | 3 |  |
| ADR | 231 |  |  | 3 |  |
| decreased nigral glutathione | C0034917 | 1 | 1 | 3 | 3 |
| matrix metalloproteinase (MMP)2 | 4313 |  |  | 3 |  |
| PDC's | 5132 | 2 | 1 | 3 | 3 |
| COLL | C0009325 | 4 | 2 | 3 | 1.5 |
| cpr | 5447 |  |  | 3 |  |
| tumor-suppressor | C0079427 | 1 | 1 | 3 | 3 |
| glutamate-cysteine ligase catalytic subunit | 2729 |  | 1 | 3 | 3 |
| glutamate + glutamine | C1718801 |  | 1 | 3 | 3 |
| IL-6/JAK/STAT3 | 3572 |  |  | 3 |  |
| tight junction (TJ) proteins | C3494363 | 1 |  | 3 |  |
| Slc11a2 | 18174 | 1 | 1 | 3 | 3 |
| Endo- | 79694 |  |  | 3 |  |
| choline acetyl transferase | C0008407 | 2 | 4 | 3 | 0.75 |
| SCH-23390 | 4771 |  | 1 | 3 | 3 |
| PARK20 | 8867 |  |  | 3 |  |
| glutamate/glutamine | C1718801 |  | 1 | 3 | 3 |
| CI 95% 2. | C0067503 |  |  | 3 |  |
| IL-10 | C0085295 |  |  | 3 |  |
| Apo | C0003591 | 1 |  | 3 |  |
| GST | C0017837 | 2 | 6 | 3 | 0.5 |
| serotonin 5-HT2A receptor | 3356 | 1 | 4 | 3 | 0.75 |
| androgen receptor Xq12 gene | 367 |  | 1 | 3 | 3 |
| C-reactive proteins | C0006560 | 2 | 3 | 3 | 1 |
| COQ2 gene | 27235 |  |  | 3 |  |
| tAIF | C0763396 | 3 | 1 | 3 | 3 |
| GCL++ | 2729 |  | 1 | 3 | 3 |
| GCL + | 2729 |  | 1 | 3 | 3 |
| CST | 1471 | 1 | 1 | 3 | 3 |
| Rab GTPases | 3267 | 3 | 1 | 3 | 3 |
| monoclonal phospho-specific antibodies | C0003250 |  | 1 | 3 | 3 |
| L-phenylalanine | C0031453 | 4 | 3 | 3 | 1 |
| huntingtin | 15194 | 3 | 4 | 2 | 0.5 |
| SOD1 | 20655 | 3 | 1 | 2 | 2 |
| Aquaporin 4 | 361 | 1 | 2 | 2 | 1 |
| AQP4 | 361 | 1 | 2 | 2 | 1 |
| water channel | C0599635 | 1 | 1 | 2 | 2 |
| Erythropoietin | 2056 | 1 | 2 | 2 | 1 |
| EPO | 2056 | 1 | 2 | 2 | 1 |
| catalase | 24248 | 2 | 2 | 2 | 1 |
| excitatory amino acids | C0243046 | 3 | 1 | 2 | 2 |
| excitatory amino acid | C0243046 | 3 | 1 | 2 | 2 |
| metallothionein | C0025545 | 3 | 1 | 2 | 2 |
| synphilin-1 | C3541909 | 8 | 5 | 2 | 0.4 |
| synphilin 1 | C3541909 | 8 | 5 | 2 | 0.4 |
| GLP-1 receptor | 25051 | 2 |  | 2 |  |
| glucagon-like peptide 1 receptor | 25051 | 2 |  | 2 |  |
| GLP-1R | 25051 | 2 |  | 2 |  |
| neuron-specific enolase | C1880904 | 3 | 2 | 2 | 1 |
| neuron specific enolase | C1880904 | 3 | 2 | 2 | 1 |
| mutant disease proteins | C1564139 | 3 | 1 | 2 | 2 |
| mutant huntingtin protein | C1564139 | 3 | 1 | 2 | 2 |
| mutant alpha-synuclein proteins | C1564139 | 3 | 1 | 2 | 2 |
| mutant aggregate-prone proteins | C1564139 | 3 | 1 | 2 | 2 |
| mutant polyglutamine proteins | C1564139 | 3 | 1 | 2 | 2 |
| Roc | C0683187 | 7 | 4 | 2 | 0.5 |
| Bcl-x | 598 | 3 | 4 | 2 | 0.5 |
| Bcl-xL | 598 | 3 | 4 | 2 | 0.5 |
| Pitx3 | 18742 | 5 | 7 | 2 | 0.285714285714286 |
| GST | 133482 | 5 | 2 | 2 | 1 |
| Exp | 4154 | 1 | 1 | 2 | 2 |
| interleukin 1-alpha | 3552 | 4 | 2 | 2 | 1 |
| IL-1alpha | 3552 | 4 | 2 | 2 | 1 |
| interleukin-1alpha | 3552 | 4 | 2 | 2 | 1 |
| IL-1 | 3552 | 4 | 2 | 2 | 1 |
| IL-1 alpha | 3552 | 4 | 2 | 2 | 1 |
| CYP2D | 1565 | 5 | 3 | 2 | 0.666666666666667 |
| CYP2D6 | 1565 | 5 | 3 | 2 | 0.666666666666667 |
| Pitx3 | 29609 | 5 |  | 2 |  |
| paired-like homeodomain transcription factor 3 | 29609 | 5 |  | 2 |  |
| signal-regulated kinase 1/ | C0082529 | 5 | 2 | 2 | 1 |
| ERK1/2 | C0082529 | 5 | 2 | 2 | 1 |
| extracellular signal-regulated protein kinase 1/ | C0082529 | 5 | 2 | 2 | 1 |
| extracellular signal-regulated kinase 1 | C0082529 | 5 | 2 | 2 | 1 |
| OX-42 | C3811761 | 5 | 4 | 2 | 0.5 |
| OX-6 | C3811761 | 5 | 4 | 2 | 0.5 |
| tropomyosin-related kinase B | C3853697 | 1 |  | 2 |  |
| TrkB | 4915 | 1 |  | 2 |  |
| valine | C0042285 | 4 | 4 | 2 | 0.5 |
| val | C0042285 | 4 | 4 | 2 | 0.5 |
| toxic misfolded proteins | C0815048 | 3 | 2 | 2 | 1 |
| toxic proteins | C0815048 | 3 | 2 | 2 | 1 |
| histone deacetylase 6 | 10013 | 3 | 2 | 2 | 1 |
| P100 | 1523 | 1 |  | 2 |  |
| NCAM | 17967 | 1 |  | 2 |  |
| Trypsin | C0041236 |  | 3 | 2 | 0.666666666666667 |
| mitochondrial superoxide dismutase | C0968147 | 4 | 3 | 2 | 0.666666666666667 |
| superoxide dismutase 2 | C0968147 | 4 | 3 | 2 | 0.666666666666667 |
| mitochondrial manganese superoxide dismutase | C0968147 | 4 | 3 | 2 | 0.666666666666667 |
| epidermal growth factor | 1950 | 4 | 1 | 2 | 2 |
| EGF | 1950 | 4 | 1 | 2 | 2 |
| Manganese superoxide dismutase | C0024708 | 4 | 2 | 2 | 1 |
| manganese-superoxide dismutase | C0024708 | 4 | 2 | 2 | 1 |
| manganese-containing superoxide dismutase | C0024708 | 4 | 2 | 2 | 1 |
| polypeptides | C1305923 | 2 | 7 | 2 | 0.285714285714286 |
| FGF20 | 26281 | 4 | 2 | 2 | 1 |
| fibroblast growth factor 20 | 26281 | 4 | 2 | 2 | 1 |
| fibroblast growth factor-20 | 26281 | 4 | 2 | 2 | 1 |
| FGF-20 | 26281 | 4 | 2 | 2 | 1 |
| gamma-glutamylcysteine ligase | C0017037 | 4 | 1 | 2 | 2 |
| gamma-glutamyl cysteine ligase | C0017037 | 4 | 1 | 2 | 2 |
| p35 | 2220 | 1 | 1 | 2 | 2 |
| regulatory proteins | C0815047 | 3 | 2 | 2 | 1 |
| regulatory/targeting proteins | C0815047 | 3 | 2 | 2 | 1 |
| UPR regulators | C0815047 | 3 | 2 | 2 | 1 |
| HDL4 | 6908 | 4 | 1 | 2 | 2 |
| TATA box binding protein (TBP) gene | 6908 | 4 | 1 | 2 | 2 |
| TBP | 6908 | 4 | 1 | 2 | 2 |
| D40 | 6908 | 4 | 1 | 2 | 2 |
| TATA box binding protein | 6908 | 4 | 1 | 2 | 2 |
| NF-kappaB p65 | 5970 | 4 | 4 | 2 | 0.5 |
| p65 subunit of NF-kappaB | 5970 | 4 | 4 | 2 | 0.5 |
| protein 18 | C0054966 | 1 |  | 2 |  |
| c-Myc | 4609 | 1 | 6 | 2 | 0.333333333333333 |
| kinase 1 (PINK1) gene | C1334324 | 3 | 3 | 2 | 0.666666666666667 |
| kinase 1 gene | C1334324 | 3 | 3 | 2 | 0.666666666666667 |
| kinase (LRRK2) gene | C1334324 | 3 | 3 | 2 | 0.666666666666667 |
| complex II | C0038615 | 3 | 2 | 2 | 1 |
| Complexes II | C0038615 | 3 | 2 | 2 | 1 |
| gamma-aminobutyric acid (GABA)- | C0016904 | 3 | 3 | 2 | 0.666666666666667 |
| GABA(A | C0016904 | 3 | 3 | 2 | 0.666666666666667 |
| Fas | 355 | 2 | 5 | 2 | 0.4 |
| CD95 | 355 | 2 | 5 | 2 | 0.4 |
| human insulin receptor | 3643 | 3 | 2 | 2 | 1 |
| HIR | 3643 | 3 | 2 | 2 | 1 |
| IR) | 3643 | 3 | 2 | 2 | 1 |
| TH-IR | 3643 | 3 | 2 | 2 | 1 |
| synthetic peptides | C0597551 | 3 | 2 | 2 | 1 |
| synthetic phospho-WOX1 peptide | C0597551 | 3 | 2 | 2 | 1 |
| synthetic beta-peptides | C0597551 | 3 | 2 | 2 | 1 |
| protein or 'canonical' amino acids | C1874187 | 1 | 5 | 2 | 0.4 |
| protein amino acids | C1874187 | 1 | 5 | 2 | 0.4 |
| amino acid protein | C1874187 | 1 | 5 | 2 | 0.4 |
| pH=4. | 54681 | 3 | 1 | 2 | 2 |
| pH 7.4 | 54681 | 3 | 1 | 2 | 2 |
| pH 5.0-7.4 | 54681 | 3 | 1 | 2 | 2 |
| peroxidase | C0027021 | 1 | 2 | 2 | 1 |
| eNOS | 4846 | 3 | 2 | 2 | 1 |
| NOS3 | 4846 | 3 | 2 | 2 | 1 |
| Thy1 | 21838 | 3 | 5 | 2 | 0.4 |
| beta-strands | C1706935 | 3 | 5 | 2 | 0.4 |
| beta-strand | C1706935 | 3 | 5 | 2 | 0.4 |
| S100beta | 20203 | 2 | 1 | 2 | 2 |
| S 100beta | 20203 | 2 | 1 | 2 | 2 |
| glutathione reductase | 2936 | 1 | 4 | 2 | 0.5 |
| p50 | 958 | 3 | 2 | 2 | 1 |
| enhanced green fluorescent protein | C1258415 | 3 | 3 | 2 | 0.666666666666667 |
| EGFP | C1258415 | 3 | 3 | 2 | 0.666666666666667 |
| butyrylcholinesterase | 590 | 1 |  | 2 |  |
| phospholipase A2 | C0031667 | 3 | 1 | 2 | 2 |
| phospholipase A(2) | C0031667 | 3 | 1 | 2 | 2 |
| MAPK/ERK | C0169101 | 2 | 4 | 2 | 0.5 |
| mitogen-activated protein kinase kinases | C0169101 | 2 | 4 | 2 | 0.5 |
| transcriptional factors | C0040648 | 2 | 4 | 2 | 0.5 |
| transcriptional factor | C0040648 | 2 | 4 | 2 | 0.5 |
| poly(ADP-ribose) polymerase | 11545 | 2 | 1 | 2 | 2 |
| PARP | 11545 | 2 | 1 | 2 | 2 |
| IGF-1 | 24482 | 2 | 1 | 2 | 2 |
| Insulin-like growth factor 1 | 24482 | 2 | 1 | 2 | 2 |
| dynorphin | C0013355 | 2 | 4 | 2 | 0.5 |
| DYN | C0013355 | 2 | 4 | 2 | 0.5 |
| estrogen receptor | C0034804 | 2 | 4 | 2 | 0.5 |
| oestrogen receptors | C0034804 | 2 | 4 | 2 | 0.5 |
| estrogen receptors | C0034804 | 2 | 4 | 2 | 0.5 |
| ER | C0034804 | 2 | 4 | 2 | 0.5 |
| LacZ | C0022959 | 2 |  | 2 |  |
| lacZ fusion gene | C0022959 | 2 |  | 2 |  |
| thioredoxin | 7295 | 1 | 4 | 2 | 0.5 |
| MMPs | C0623362 | 2 | 4 | 2 | 0.5 |
| matrix metalloproteinases | C0623362 | 2 | 4 | 2 | 0.5 |
| extracellular signal-regulated kinases 1 and 2 | C0170168 | 1 | 4 | 2 | 0.5 |
| ERK1/2 | C0170168 | 1 | 4 | 2 | 0.5 |
| MCS | 4183 | 2 |  | 2 |  |
| MCs) | 4183 | 2 |  | 2 |  |
| synaptophysin | 6855 | 2 | 3 | 2 | 0.666666666666667 |
| SMN2 | 6607 | 1 | 1 | 2 | 2 |
| MEK | 5609 | 2 | 1 | 2 | 2 |
| HDAC | 9734 | 2 | 1 | 2 | 2 |
| HDAC inhibitors | 9734 | 2 | 1 | 2 | 2 |
| dityrosine | C0058505 | 2 | 1 | 2 | 2 |
| UPS proteins | C1956096 | 1 | 1 | 2 | 2 |
| neural protein | C0027759 | 2 | 2 | 2 | 1 |
| neural phenotypic proteins | C0027759 | 2 | 2 | 2 | 1 |
| proline | C0033382 | 2 | 1 | 2 | 2 |
| annexin V | 308 | 2 | 3 | 2 | 0.666666666666667 |
| enkephalin | C0014298 | 2 |  | 2 |  |
| thyroid-releasing hormone | C0040135 | 1 | 1 | 2 | 2 |
| Thyroid hormone | C0040135 | 1 | 1 | 2 | 2 |
| mGluR agonist L-(+)-2-amino-4 | C0667286 | 2 | 3 | 2 | 0.666666666666667 |
| metabotropic glutamate receptor 4 | C0667286 | 2 | 3 | 2 | 0.666666666666667 |
| PKCdelta | 5580 | 2 |  | 2 |  |
| protein kinase C delta | 5580 | 2 |  | 2 |  |
| laminin | C0022984 | 2 | 3 | 2 | 0.666666666666667 |
| liver-X-receptors | C1612060 | 2 |  | 2 |  |
| liver X receptor | C1612060 | 2 |  | 2 |  |
| LXR | C1612060 | 2 |  | 2 |  |
| tet | C1515333 | 2 | 3 | 2 | 0.666666666666667 |
| Delta3 | C3542919 | 2 |  | 2 |  |
| change 3. | C3542919 | 2 |  | 2 |  |
| calbindin | C0054444 | 2 |  | 2 |  |
| calbindin- | C0054444 | 2 |  | 2 |  |
| deacetylases | C1333261 |  | 3 | 2 | 0.666666666666667 |
| NR2B | 24410 | 2 | 3 | 2 | 0.666666666666667 |
| cyclin-dependent kinase 5 | 140908 | 1 | 1 | 2 | 2 |
| Cdk5 | 140908 | 1 | 1 | 2 | 2 |
| E12 | 64100 | 2 | 1 | 2 | 2 |
| COMT | 24267 | 2 |  | 2 |  |
| catechol-O-methyltransferase | 24267 | 2 |  | 2 |  |
| the SAM | 9652 | 2 | 2 | 2 | 1 |
| RAB8A | 4218 | 1 |  | 2 |  |
| p47PHOX | 17969 | 2 |  | 2 |  |
| i) group 1 | C0963236 | 2 | 2 | 2 | 1 |
| I(1 | C0963236 | 2 | 2 | 2 | 1 |
| PrP(c) | 19122 | 1 | 3 | 2 | 0.666666666666667 |
| PrP | 19122 | 1 | 3 | 2 | 0.666666666666667 |
| PrP(c | 19122 | 1 | 3 | 2 | 0.666666666666667 |
| PDEs | C0031640 | 2 |  | 2 |  |
| phosphodiesterase | C0031640 | 2 |  | 2 |  |
| PDE | C0031640 | 2 |  | 2 |  |
| oxidase | C0242417 | 1 | 3 | 2 | 0.666666666666667 |
| val | C0078011 | 1 | 1 | 2 | 2 |
| cystine | C0010682 | 2 | 2 | 2 | 1 |
| cell surface receptors | C0034800 | 2 | 2 | 2 | 1 |
| IL-1 | C0021755 | 2 | 1 | 2 | 2 |
| Interleukin-1 | C0021755 | 2 | 1 | 2 | 2 |
| phospholipases | C0031671 | 2 | 1 | 2 | 2 |
| Membrane-bound | 19 | 2 |  | 2 |  |
| ABCA1 | 19 | 2 |  | 2 |  |
| JNK | 26419 | 1 | 3 | 2 | 0.666666666666667 |
| Bim | 10018 | 1 | 3 | 2 | 0.666666666666667 |
| Atg7 | 10533 | 1 | 1 | 2 | 2 |
| CSF proteins | C0007810 | 1 | 3 | 2 | 0.666666666666667 |
| cyclin | C0072108 | 1 | 1 | 2 | 2 |
| proliferating cell nuclear antigen | C0072108 | 1 | 1 | 2 | 2 |
| SV2A | 9900 |  |  | 2 |  |
| synaptic vesicle glycoprotein 2A | 9900 |  |  | 2 |  |
| somatostatin | C0037659 |  | 2 | 2 | 1 |
| cathepsin | C0007428 | 1 |  | 2 |  |
| cathepsin B | 1508 | 1 |  | 2 |  |
| D2-D3 | 1734 | 1 | 2 | 2 | 1 |
| different disease-associated proteins | C0082213 |  | 1 | 2 | 2 |
| different polyglutamine proteins | C0082213 |  | 1 | 2 | 2 |
| aldehyde dehydrogenase 1 | C0538135 | 1 | 2 | 2 | 1 |
| Histone deacetylase inhibitors | C1512474 | 1 | 1 | 2 | 2 |
| histone deacetylase (HDAC) inhibitors | C1512474 | 1 | 1 | 2 | 2 |
| HDAC inhibitors | C1512474 | 1 | 1 | 2 | 2 |
| histone deacetylase (HDAC) inhibitor | C1512474 | 1 | 1 | 2 | 2 |
| IGF-1- | C0021665 | 1 | 2 | 2 | 1 |
| insulin-like growth factors-I | C0021665 | 1 | 2 | 2 | 1 |
| SIRT1 | 93759 | 1 | 2 | 2 | 1 |
| GTP cyclohydrolase I | C0205775 | 1 | 2 | 2 | 1 |
| Hsp | 7190 | 1 | 2 | 2 | 1 |
| integrins | C0021701 | 1 | 2 | 2 | 1 |
| integrin | C0021701 | 1 | 2 | 2 | 1 |
| endothelial growth factor | C0079280 |  | 1 | 2 | 2 |
| cell adhesion molecules | C0007578 | 1 | 2 | 2 | 1 |
| CHIP | 358 | 1 | 1 | 2 | 2 |
| IGF-1R | 25718 | 1 |  | 2 |  |
| hMSC | 9242 | 1 |  | 2 |  |
| hexokinase | C0019472 | 1 |  | 2 |  |
| 5-HT2A receptor | 22954 | 1 |  | 2 |  |
| 5-HT2A receptors | 22954 | 1 |  | 2 |  |
| CASP7 | 840 | 1 |  | 2 |  |
| ABC | 10058 | 1 | 2 | 2 | 1 |
| epsinR | 9685 | 1 |  | 2 |  |
| SHH | 20423 | 1 |  | 2 |  |
| MAP kinase | 27352 | 1 | 1 | 2 | 2 |
| p38 mitogen-activated protein kinase | 1432 | 1 |  | 2 |  |
| PCA | 28982 | 1 |  | 2 |  |
| alpha-D-galactosidase A | 2717 | 1 |  | 2 |  |
| angiotensin II | 183 | 1 |  | 2 |  |
| protein phosphatase 2A | C1704708 | 1 | 2 | 2 | 1 |
| caspase-4 | 837 |  |  | 2 |  |
| NTF | C0132298 | 1 |  | 2 |  |
| myelin basic protein | 4155 | 1 | 1 | 2 | 2 |
| NR2A | 24409 | 1 | 1 | 2 | 2 |
| glyoxalase | C0022956 | 1 | 1 | 2 | 2 |
| Prolyl oligopeptidase | 5550 | 1 | 1 | 2 | 2 |
| Kcnn2 | 3781 | 1 | 1 | 2 | 2 |
| SK2 | 3781 | 1 | 1 | 2 | 2 |
| Kcnn2 gene | 3781 | 1 | 1 | 2 | 2 |
| CFC | 5781 | 1 |  | 2 |  |
| des | 1674 |  | 1 | 2 | 2 |
| P200 | 10565 | 1 |  | 2 |  |
| glucose-regulated protein | C2599779 | 1 | 2 | 2 | 1 |
| NAA/Cr | C0010286 | 1 |  | 2 |  |
| polymerase | 100616102 | 1 |  | 2 |  |
| FACTT | 100616102 | 1 |  | 2 |  |
| Foxa2 | 3170 |  | 1 | 2 | 2 |
| ChAT | 1103 | 1 | 1 | 2 | 2 |
| basic fibroblastic growth factor | C0380603 | 1 |  | 2 |  |
| basic fibroblast growth factor | C0380603 | 1 |  | 2 |  |
| Pael-R | 14763 | 1 |  | 2 |  |
| enzyme's | C0014442 | 1 |  | 2 |  |
| EGFR | 1956 |  | 2 | 2 | 1 |
| IFN | 3439 | 1 |  | 2 |  |
| alpha-secretase | C0379526 |  |  | 2 |  |
| CaV1.3 | 776 | 1 | 1 | 2 | 2 |
| Ca(V)1.3 | 776 | 1 | 1 | 2 | 2 |
| lysosomal enzymes | C0391845 | 1 | 2 | 2 | 1 |
| Rho-associated kinase | C0389995 |  | 1 | 2 | 2 |
| Ndufs4 | 17993 | 1 | 1 | 2 | 2 |
| LDL-C | 22796 | 1 |  | 2 |  |
| secreted phosphoprotein 1 | 6696 |  | 2 | 2 | 1 |
| SPP1 | 6696 |  | 2 | 2 | 1 |
| osteopontin | 6696 |  | 2 | 2 | 1 |
| Paris | 155061 |  |  | 2 |  |
| calcium-binding protein | C0006732 | 1 | 1 | 2 | 2 |
| calcium binding proteins | C0006732 | 1 | 1 | 2 | 2 |
| protein 1 isoform | C0597298 | 1 | 2 | 2 | 1 |
| mES | 4199 | 1 |  | 2 |  |
| microtubule-associated protein 1 light chain 3 | C3540600 | 1 |  | 2 |  |
| AADC | 24311 | 1 | 1 | 2 | 2 |
| PSF | 3490 | 1 |  | 2 |  |
| RING1 | 6015 | 1 |  | 2 |  |
| IL-6Ralpha | 3570 |  |  | 2 |  |
| GLAST | 29483 | 1 | 1 | 2 | 2 |
| EAAT1 | 29483 | 1 | 1 | 2 | 2 |
| IL-1 beta converting enzyme | 25166 | 1 |  | 2 |  |
| ICE | 25166 | 1 |  | 2 |  |
| MTs | 1678 | 1 |  | 2 |  |
| beta-arrestin2 | 409 | 1 |  | 2 |  |
| Trp | 7306 | 1 | 1 | 2 | 2 |
| 3-hydroxy-3-methylglutaryl-CoA reductase | 3156 | 1 |  | 2 |  |
| Metallothionein-III | 4504 | 1 | 1 | 2 | 2 |
| MT-III | 4504 | 1 | 1 | 2 | 2 |
| Ngn2 | 11924 | 1 |  | 2 |  |
| IkappaBalpha | 4792 | 1 | 1 | 2 | 2 |
| mitochondrial transcription factor A | C0965025 |  | 1 | 2 | 2 |
| TFAM | 21780 |  |  | 2 |  |
| Glucagon-like peptide 1 receptor | C0378073 | 1 | 1 | 2 | 2 |
| ubiquitin C | 7316 | 1 |  | 2 |  |
| HMG-CoA reductase | C0020374 |  |  | 2 |  |
| Pleiotrophin | 5764 | 1 | 1 | 2 | 2 |
| HER2 | 2064 | 1 |  | 2 |  |
| interferon-gamma | C0021740 | 1 |  | 2 |  |
| voltage dependent anion channel | C1506024 | 1 | 1 | 2 | 2 |
| VDAC | C1506024 | 1 | 1 | 2 | 2 |
| occludin | 18260 | 1 |  | 2 |  |
| GM-CSF | 1437 |  |  | 2 |  |
| beta-N-oxalyl amino-L-alanine | C0000392 | 1 |  | 2 |  |
| MS) | 4397 | 1 |  | 2 |  |
| PDE4 | 5141 | 1 |  | 2 |  |
| Angelman's syndrome | 7337 |  |  | 2 |  |
| recessive genes | C0017361 |  | 2 | 2 | 1 |
| HDAC6 | 10013 | 3 | 2 | 2 | 1 |
| scaffolding protein | C1179132 |  | 1 | 2 | 2 |
| Metallothioneins | C0025545 | 3 | 1 | 2 | 2 |
| inhibitors 1 | 5502 | 1 | 2 | 2 | 1 |
| Cell-surface glutamate receptors | C0034800 | 2 | 2 | 2 | 1 |
| proopiomelanocortin | 5443 | 1 |  | 2 |  |
| POMC | 5443 | 1 |  | 2 |  |
| gamma aminobutyric acid | C0016904 | 3 | 3 | 2 | 0.666666666666667 |
| enkephalins | C0014298 | 2 |  | 2 |  |
| aromatic amino acid | C0301713 | 1 |  | 2 |  |
| guanosine triphosphate) cyclohydrolase I | C0205775 | 1 | 2 | 2 | 1 |
| dihydroxyphenylalanine)- | C0013023 | 1 |  | 2 |  |
| sortilin | C0536670 |  | 1 | 2 | 2 |
| Src | 6714 | 1 | 1 | 2 | 2 |
| cytochrome P450 2D6 | 1565 | 5 | 3 | 2 | 0.666666666666667 |
| RAGE | 177 |  |  | 2 |  |
| SKP1 | 6500 |  | 2 | 2 | 1 |
| 4-Oct | 5362 |  | 3 | 2 | 0.666666666666667 |
| extracellular signal-regulated kinase (ERK)1 | C0082529 | 5 | 2 | 2 | 1 |
| p42 | 6908 | 4 | 1 | 2 | 2 |
| HDAC inhibitor | C1512474 | 1 | 1 | 2 | 2 |
| Orexin a | C0671870 |  | 1 | 2 | 2 |
| Orexin Receptor-1 | 3061 |  | 1 | 2 | 2 |
| OXA | C0671870 |  | 1 | 2 | 2 |
| scaffold proteins | C1179132 |  | 1 | 2 | 2 |
| GPER1 | 76854 |  |  | 2 |  |
| CAT | 24248 | 2 | 2 | 2 | 1 |
| ferritin light chain | C0391764 |  | 1 | 2 | 2 |
| IDP | 3417 |  | 3 | 2 | 0.666666666666667 |
| dopa decarboxylase | C0003806 |  | 4 | 2 | 0.5 |
| ToM | 24148 |  | 2 | 2 | 1 |
| MAO-A | 17161 |  | 2 | 2 | 1 |
| Th17) | 51497 |  | 1 | 2 | 2 |
| enhanced green fluorescence protein | C1258415 | 3 | 3 | 2 | 0.666666666666667 |
| mitogen-activated protein kinase | C0169101 | 2 | 4 | 2 | 0.5 |
| D(2 | 1734 | 1 | 2 | 2 | 1 |
| Src family tyrosine kinases | C0282625 |  |  | 2 |  |
| c-Src | 6714 | 1 | 1 | 2 | 2 |
| Src family kinase | C0282625 |  |  | 2 |  |
| transcriptional coactivator | C1336776 |  | 3 | 2 | 0.666666666666667 |
| protein amino acid | C1874187 | 1 | 5 | 2 | 0.4 |
| PTN | 5764 | 1 | 1 | 2 | 2 |
| sequestosome 1 | 8878 |  | 3 | 2 | 0.666666666666667 |
| p62 | 8878 |  | 3 | 2 | 0.666666666666667 |
| SQSTM1 | 8878 |  | 3 | 2 | 0.666666666666667 |
| SLC41A1 | 254428 |  | 2 | 2 | 1 |
| hSLC41A1 | 254428 |  | 2 | 2 | 1 |
| ROC-COR | C0683187 | 7 | 4 | 2 | 0.5 |
| neural-specific protein | C0027759 | 2 | 2 | 2 | 1 |
| wise | C1427738 |  | 2 | 2 | 1 |
| laminins | C0022984 | 2 | 3 | 2 | 0.666666666666667 |
| calcineurin | C0054450 |  | 1 | 2 | 2 |
| CaN | C0054450 |  | 1 | 2 | 2 |
| UTR | 8170 |  | 1 | 2 | 2 |
| GPx | 384001 |  | 2 | 2 | 1 |
| gp91phox | 1536 |  | 2 | 2 | 1 |
| gp91(phox) | 1536 |  | 2 | 2 | 1 |
| E3 ubiquitin ligase complex | C1256818 |  |  | 2 |  |
| transcription co-activator | C1336776 |  | 3 | 2 | 0.666666666666667 |
| arm | 10327 |  | 1 | 2 | 2 |
| ATF6 | 22926 |  | 1 | 2 | 2 |
| GABA receptors | C0206518 |  | 4 | 2 | 0.5 |
| Nogo receptor | 65078 |  | 1 | 2 | 2 |
| Gamma-aminobutyric acid(B) receptor | C0206518 |  | 4 | 2 | 0.5 |
| IL-17 | 3605 |  | 1 | 2 | 2 |
| LRP | 3949 |  | 2 | 2 | 1 |
| transcriptional activator | C1336776 |  | 3 | 2 | 0.666666666666667 |
| the S | 9652 | 2 | 2 | 2 | 1 |
| calmodulin kinase | C0006776 |  | 1 | 2 | 2 |
| VEGF | 22339 |  | 1 | 2 | 2 |
| SKP1A | 6500 |  | 2 | 2 | 1 |
| glucocorticoid receptors | C0034809 |  | 1 | 2 | 2 |
| beta-carbomethoxy-3 | 1934 |  | 3 | 2 | 0.666666666666667 |
| Mfn1 | 55669 |  |  | 2 |  |
| glucocorticoid receptor | C0034809 |  | 1 | 2 | 2 |
| PER1 | 5187 |  | 1 | 2 | 2 |
| Ca(v)1.3 | 12289 |  | 1 | 2 | 2 |
| CACNA1D | 12289 |  | 1 | 2 | 2 |
| PTPs | 5805 |  | 1 | 2 | 2 |
| vitamin D receptor | 7421 |  | 3 | 2 | 0.666666666666667 |
| insulin-like growth factor-I | C0021665 | 1 | 2 | 2 | 1 |
| DA | C0013023 | 1 |  | 2 |  |
| transcriptions factors | C0040648 | 2 | 4 | 2 | 0.5 |
| Receptor for advanced glycation endproducts | C0101725 |  | 1 | 2 | 2 |
| RAGE | C0101725 |  | 1 | 2 | 2 |
| NF-kappaB subunit p65 | 5970 | 4 | 4 | 2 | 0.5 |
| prolyl oligopeptidase | C0072132 |  | 1 | 2 | 2 |
| PREP | 5550 | 1 | 1 | 2 | 2 |
| IkappaB-alpha | 4792 | 1 | 1 | 2 | 2 |
| PGC-1alpha | 83516 |  | 2 | 2 | 1 |
| peroxisome proliferator-activated receptor gamma coactivator-1 alpha | 83516 |  | 2 | 2 | 1 |
| PLK-2 | 10769 |  | 4 | 2 | 0.5 |
| PLK2 | 10769 |  | 4 | 2 | 0.5 |
| Apoptosis signal-regulating kinase 1 | 4217 |  | 1 | 2 | 2 |
| phospholipase | C0031671 | 2 | 1 | 2 | 2 |
| PI3K inhibitor | C3537035 |  | 1 | 2 | 2 |
| kinase (PI3K) inhibitor | C3537035 |  | 1 | 2 | 2 |
| PI3K/Akt inhibitor | C3537035 |  | 1 | 2 | 2 |
| guanosine triphosphate cyclohydrolase 1 | C0205775 | 1 | 2 | 2 | 1 |
| phospholipase-A2 | C0031667 | 3 | 1 | 2 | 2 |
| TfR1 | 7037 |  | 1 | 2 | 2 |
| ULK1 | 8408 |  | 1 | 2 | 2 |
| ferroportin | C0915115 |  | 1 | 2 | 2 |
| LC3 | 66734 |  | 2 | 2 | 1 |
| IkappaBalpha | 18035 |  | 2 | 2 | 1 |
| GTP cyclohydrolase-1 | C0205775 | 1 | 2 | 2 | 1 |
| MBP | 4155 | 1 | 1 | 2 | 2 |
| 4-EBP | 10682 |  | 5 | 2 | 0.4 |
| Iron Regulatory Protein 1 | 48 |  | 2 | 2 | 1 |
| intercellular adhesion molecule 1 | 3383 |  | 1 | 2 | 2 |
| VDR | 7421 |  | 3 | 2 | 0.666666666666667 |
| amino-acid protein | C1874187 | 1 | 5 | 2 | 0.4 |
| ATR | 545 |  | 1 | 2 | 2 |
| TRAP1 | 10131 |  | 1 | 2 | 2 |
| synthase 3 | 4846 | 3 | 2 | 2 | 1 |
| PP2A | C1704708 | 1 | 2 | 2 | 1 |
| Apolipoprotein B-100 | 338 |  | 1 | 2 | 2 |
| adhesion molecule | C0007578 | 1 | 2 | 2 | 1 |
| Cyp) 2D6 gene | 1565 | 5 | 3 | 2 | 0.666666666666667 |
| superoxide dismutase-2 | C0968147 | 4 | 3 | 2 | 0.666666666666667 |
| noradrenaline transporters | C0132800 |  | 1 | 2 | 2 |
| PS-129 | 2843875 |  | 1 | 2 | 2 |
| SPS | 6611 |  | 2 | 2 | 1 |
| pH 4. | 54681 | 3 | 1 | 2 | 2 |
| PSG | 5673 |  | 4 | 2 | 0.5 |
| Myc | 4609 | 1 | 6 | 2 | 0.333333333333333 |
| interleukin-1 alpha | 3552 | 4 | 2 | 2 | 1 |
| PrP(res) | C0074204 |  | 1 | 2 | 2 |
| DNA binding protein | C0012940 |  | 3 | 2 | 0.666666666666667 |
| dynorphins | C0013355 | 2 | 4 | 2 | 0.5 |
| dynorphin's | C0013355 | 2 | 4 | 2 | 0.5 |
| ERKO | C0034804 | 2 | 4 | 2 | 0.5 |
| ALDH1 | 216 |  | 2 | 2 | 1 |
| DDC | C0003806 |  | 4 | 2 | 0.5 |
| GLP-1 receptors | C0378073 | 1 | 1 | 2 | 2 |
| NCL | 4691 |  | 1 | 2 | 2 |
| i) 1 | C0963236 | 2 | 2 | 2 | 1 |
| monoamine oxidase A | 17161 |  | 2 | 2 | 1 |
| ICAM | C0007578 | 1 | 2 | 2 | 1 |
| recessive genetic | C0017361 |  | 2 | 2 | 1 |
| Glucose regulated protein | C2599779 | 1 | 2 | 2 | 1 |
| activating transcription factor-6 | 22926 |  | 1 | 2 | 2 |
| GRP78/BiP | C2599779 | 1 | 2 | 2 | 1 |
| MCCC1 | 56922 |  | 1 | 2 | 2 |
| STK39 | 27347 |  | 2 | 2 | 1 |
| transient receptor potential channel 1 | 7220 |  | 1 | 2 | 2 |
| TRPC1 | 7220 |  | 1 | 2 | 2 |
| DNA-binding protein | C0012940 |  | 3 | 2 | 0.666666666666667 |
| aid | 57379 |  | 2 | 2 | 1 |
| metabotropic glutamate receptor type 4 | C0667286 | 2 | 3 | 2 | 0.666666666666667 |
| fatty acid amide hydrolase | C0531004 |  | 1 | 2 | 2 |
| transferrin receptor | 7037 |  | 1 | 2 | 2 |
| water channel proteins | C0599635 | 1 | 1 | 2 | 2 |
| WCPs | C0599635 | 1 | 1 | 2 | 2 |
| PARP1 | 11545 | 2 | 1 | 2 | 2 |
| chondroitinase ABC | 10058 | 1 | 2 | 2 | 1 |
| ChABC | 10058 | 1 | 2 | 2 | 1 |
| alpha-Synuclein protein | C2362546 |  | 2 | 2 | 1 |
| Trx | 7295 | 1 | 4 | 2 | 0.5 |
| human Trx1 gene | 7295 | 1 | 4 | 2 | 0.5 |
| PDs | 5172 |  | 2 | 2 | 1 |
| cMyc | 4609 | 1 | 6 | 2 | 0.333333333333333 |
| MMP | C0623362 | 2 | 4 | 2 | 0.5 |
| TLR2 | 24088 |  | 2 | 2 | 1 |
| major histocompatibility complex | C0024518 |  |  | 2 |  |
| recessive Parkinson disease genes | C0017361 |  | 2 | 2 | 1 |
| recessive PD-linked familial genes | C0017361 |  | 2 | 2 | 1 |
| NQO1 | 18104 |  | 1 | 2 | 2 |
| storage protein | C0815045 |  | 1 | 2 | 2 |
| glyoxalases | C0022956 | 1 | 1 | 2 | 2 |
| P60 | 8878 |  | 3 | 2 | 0.666666666666667 |
| #NAME? | C0012940 |  | 3 | 2 | 0.666666666666667 |
| IgM | 959 |  | 1 | 2 | 2 |
| BCL-X(L) | 598 | 3 | 4 | 2 | 0.5 |
| Bcl-X( | 598 | 3 | 4 | 2 | 0.5 |
| MAPK kinase | C0169101 | 2 | 4 | 2 | 0.5 |
| c-Jun | 16476 |  | 2 | 2 | 1 |
| Jun | 16476 |  | 2 | 2 | 1 |
| caspase-9 | 12371 |  | 1 | 2 | 2 |
| Rho-kinase | C0389995 |  | 1 | 2 | 2 |
| ROCK | C0389995 |  | 1 | 2 | 2 |
| beta2-AR | 154 |  | 1 | 2 | 2 |
| epidermal growth factor receptor | 1956 |  | 2 | 2 | 1 |
| ALDH1A1 | 216 |  | 2 | 2 | 1 |
| Aldehyde dehydrogenase 1 family, member A1 | 216 |  | 2 | 2 | 1 |
| nitric oxide synthase 3 | 4846 | 3 | 2 | 2 | 1 |
| C/EBP homologous protein | 10682 |  | 5 | 2 | 0.4 |
| Kinase 2 (LRRK2) gene | C1334324 | 3 | 3 | 2 | 0.666666666666667 |
| cerebrospinal fluid (CSF) proteins | C0007810 | 1 | 3 | 2 | 0.666666666666667 |
| endonuclease | C0014230 |  | 1 | 2 | 2 |
| NOX2 | 1536 |  | 2 | 2 | 1 |
| metabotropic glutamate receptor subtype 4 | C0667286 | 2 | 3 | 2 | 0.666666666666667 |
| P1' ( | 5551 |  | 1 | 2 | 2 |
| extracellular signal-regulated kinases 1/ | C0170168 | 1 | 4 | 2 | 0.5 |
| arm | C3715044 |  | 2 | 2 | 1 |
| prion protein | 19122 | 1 | 3 | 2 | 0.666666666666667 |
| alpha7-nAChR | 11441 |  | 1 | 2 | 2 |
| HLA genetic | C0599797 |  | 1 | 2 | 2 |
| Polo-like kinase 2 | 10769 |  | 4 | 2 | 0.5 |
| aldehyde dehydrogenase family 1 | C0538135 | 1 | 2 | 2 | 1 |
| low-density lipoprotein receptor gene | 3949 |  | 2 | 2 | 1 |
| LDLR gene | 3949 |  | 2 | 2 | 1 |
| oestrogen receptor | C0034804 | 2 | 4 | 2 | 0.5 |
| protein isoforms | C0597298 | 1 | 2 | 2 | 1 |
| cerebrospinal fluid peptides/proteins | C0007810 | 1 | 3 | 2 | 0.666666666666667 |
| oxidases | C0242417 | 1 | 3 | 2 | 0.666666666666667 |
| Ubiquitin-conjugated proteins | C1956096 | 1 | 1 | 2 | 2 |
| Insulin-like growth factor-1 | 24482 | 2 | 1 | 2 | 2 |
| Insulin-like growth factor (IGF)-1 | C0021665 | 1 | 2 | 2 | 1 |
| transcription activator | C1336776 |  | 3 | 2 | 0.666666666666667 |
| succinate dehydrogenase | C0038615 | 3 | 2 | 2 | 1 |
| trkB Receptors | C0084873 |  | 1 | 2 | 2 |
| BDNF receptor | C0084873 |  | 1 | 2 | 2 |
| GR | 2936 | 1 | 4 | 2 | 0.5 |
| i) (>/=1 | C0963236 | 2 | 2 | 2 | 1 |
| beta-carbo-methoxy-3 | 1934 |  | 3 | 2 | 0.666666666666667 |
| CD206 | 4360 |  | 1 | 2 | 2 |
| SIRT1 | 309757 |  | 1 | 2 | 2 |
| MMP+ | C0623362 | 2 | 4 | 2 | 0.5 |
| Metallothioneins (MT)-III | 4504 | 1 | 1 | 2 | 2 |
| TLR 2 | 24088 |  | 2 | 2 | 1 |
| kinase (GAK) gene | C1334324 | 3 | 3 | 2 | 0.666666666666667 |
| amino acid presynaptic protein | C1874187 | 1 | 5 | 2 | 0.4 |
| spectrin | C0037799 |  | 1 | 2 | 2 |
| protein tyrosine phosphatase 1B | 5770 |  | 1 | 2 | 2 |
| OPN | 6696 |  | 2 | 2 | 1 |
| integrin receptors | C0021701 | 1 | 2 | 2 | 1 |
| MAGNA-IR | 3643 | 3 | 2 | 2 | 1 |
| 0 | C0170168 | 1 | 4 | 2 | 0.5 |
| HLA-DRB5 genes | 3127 |  | 1 | 2 | 2 |
| HLA-DRB5 | 3127 |  | 1 | 2 | 2 |
| superoxide dismutase (SOD)-1/-2 | C0968147 | 4 | 3 | 2 | 0.666666666666667 |
| inhibitor-1 | 5502 | 1 | 2 | 2 | 1 |
| mdivi-1 | 5502 | 1 | 2 | 2 | 1 |
| adenosine A(1) receptor | 134 |  | 1 | 2 | 2 |
| protein kinase C isoforms | C0597298 | 1 | 2 | 2 | 1 |
| dsRED | C0960939 |  | 1 | 2 | 2 |
| monoamine oxidase (MAO) B | C0026456 |  | 3 | 2 | 0.666666666666667 |
| glutamate transporter | 29483 | 1 | 1 | 2 | 2 |
| DARPP-32 | 84152 |  |  | 2 |  |
| changing LAMP-2 protein | C0082213 |  | 1 | 2 | 2 |
| diethyldithiocarbamate | C0556252 |  | 1 | 2 | 2 |
| DEDC | C0556252 |  | 1 | 2 | 2 |
| porins | C0071728 |  | 2 | 2 | 1 |
| MAO type B) | C0026456 |  | 3 | 2 | 0.666666666666667 |
| clusterin | 1191 |  | 1 | 2 | 2 |
| p62 | 117268 |  | 1 | 2 | 2 |
| Bim protein | 10018 | 1 | 3 | 2 | 0.666666666666667 |
| LRP6 | 4040 |  | 1 | 2 | 2 |
| OCT | 5362 |  | 3 | 2 | 0.666666666666667 |
| beta-catenin | 84353 |  | 1 | 2 | 2 |
| Tgfb1 | 59086 |  | 1 | 2 | 2 |
| transforming growth factor beta1 | 59086 |  | 1 | 2 | 2 |
| TGF-beta1 | 59086 |  | 1 | 2 | 2 |
| mitogen-activated protein kinases (MAPKs) extracellular signal-regulated kinase 1 and 2 | C0170168 | 1 | 4 | 2 | 0.5 |
| extracellular signal-regulated protein kinase kinase 1 | C0082529 | 5 | 2 | 2 | 1 |
| mutant huntingtin proteins | C1564139 | 3 | 1 | 2 | 2 |
| HTT | 15194 | 3 | 4 | 2 | 0.5 |
| TFEB | 21425 |  | 1 | 2 | 2 |
| GABA-receptors | C0206518 |  | 4 | 2 | 0.5 |
| CRHR1 | 1394 |  | 1 | 2 | 2 |
| recessive EOPD genes | C0017361 |  | 2 | 2 | 1 |
| Gly-Trp | 7306 | 1 | 1 | 2 | 2 |
| polo-like kinase | 5347 |  | 1 | 2 | 2 |
| polo-like kinases | 5347 |  | 1 | 2 | 2 |
| myeloperoxidase | 17523 |  | 1 | 2 | 2 |
| EBP | 10682 |  | 5 | 2 | 0.4 |
| scaffolding proteins | C1179132 |  | 1 | 2 | 2 |
| beta-carboxymethyoxy-3 | 1934 |  | 3 | 2 | 0.666666666666667 |
| angiogenin | 283 |  | 1 | 2 | 2 |
| ANG | 283 |  | 1 | 2 | 2 |
| opioid receptors | C0034801 |  |  | 2 |  |
| Voltage-dependent anion channels | C1506024 | 1 | 1 | 2 | 2 |
| VDACs | C1506024 | 1 | 1 | 2 | 2 |
| voltage-dependent anion channels 1 | C0753015 |  | 1 | 2 | 2 |
| VDACs 1 | C0753015 |  | 1 | 2 | 2 |
| pore-forming proteins | C0071728 |  | 2 | 2 | 1 |
| PGI-I | 633 |  | 1 | 2 | 2 |
| rhTFAM | C0965025 |  | 1 | 2 | 2 |
| ErbB | 1956 |  | 2 | 2 | 1 |
| isoform's | C0597298 | 1 | 2 | 2 | 1 |
| PERK | 13666 |  | 1 | 2 | 2 |
| GRP78 | 14828 |  | 1 | 2 | 2 |
| CHOP | 13198 |  | 1 | 2 | 2 |
| Phosphodiesterases | C0031640 | 2 |  | 2 |  |
| SRS | 6611 |  | 2 | 2 | 1 |
| large surface coat gene | 9215 |  | 1 | 2 | 2 |
| H2AX | 3014 |  | 1 | 2 | 2 |
| gamma-aminobutyric acid receptor | C0206518 |  | 4 | 2 | 0.5 |
| Rho kinases | C0389995 |  | 1 | 2 | 2 |
| IL-13 | 16163 |  | 1 | 2 | 2 |
| Il13ra1 | 3597 |  | 1 | 2 | 2 |
| S100B | 20203 | 2 | 1 | 2 | 2 |
| immunoglobulin G | C0020852 |  | 1 | 2 | 2 |
| nuances | 23224 |  |  | 2 |  |
| synaptosomal-associated protein of 25 | C0075689 |  | 1 | 2 | 2 |
| PrPSc | 19122 | 1 | 3 | 2 | 0.666666666666667 |
| Trx/Prx | 7295 | 1 | 4 | 2 | 0.5 |
| type B monoamine oxidase | C0026456 |  | 3 | 2 | 0.666666666666667 |
| SPES-SCOPA | C0764874 |  | 1 | 2 | 2 |
| alpha-synuclein/fluorescent-protein | C2362546 |  | 2 | 2 | 1 |
| daf-16 | 172981 |  | 1 | 2 | 2 |
| GPX4 | 2879 |  | 1 | 2 | 2 |
| DPI | 1832 |  | 1 | 2 | 2 |
| HDAC inhibitor | 9734 | 2 | 1 | 2 | 2 |
| EGR3 | 1960 |  | 1 | 2 | 2 |
| c-Src | 83805 |  | 1 | 2 | 2 |
| integrators | C0309311 |  | 1 | 2 | 2 |
| norepinephrine transporter (NET) protein | C0132800 |  | 1 | 2 | 2 |
| v-myc | 4609 | 1 | 6 | 2 | 0.333333333333333 |
| estrogen-related receptor | C0034804 | 2 | 4 | 2 | 0.5 |
| cholinesterase | C0008429 |  | 1 | 2 | 2 |
| FGF20 gene | 26281 | 4 | 2 | 2 | 1 |
| alpha-melanocyte-stimulating hormone | 5443 | 1 |  | 2 |  |
| alpha-MSH | 5443 | 1 |  | 2 |  |
| TMEM175-GAK | 84286 |  |  | 2 |  |
| Growth Associated Protein-43 | 2596 |  |  | 2 |  |
| GAP-43 | 2596 |  |  | 2 |  |
| ROCK2 | 9475 |  |  | 2 |  |
| Different proteins | C0082213 |  | 1 | 2 | 2 |
| adhesion molecules | C0007578 | 1 | 2 | 2 | 1 |
| Aquaporin 1 | 358 | 1 | 1 | 2 | 2 |
| AQP1 | 358 | 1 | 1 | 2 | 2 |
| SPR | 6697 |  |  | 2 |  |
| Mutant GBA proteins | C1564139 | 3 | 1 | 2 | 2 |
| ICER | 1390 |  |  | 2 |  |
| myc proto-oncogenes | 4609 | 1 | 6 | 2 | 0.333333333333333 |
| Rho-associated protein kinase | C0389995 |  | 1 | 2 | 2 |
| neutral amino acids | C0301715 |  |  | 2 |  |
| kinase genes | C1334324 | 3 | 3 | 2 | 0.666666666666667 |
| unc-51 like autophagy activating kinase 1 | 8408 |  | 1 | 2 | 2 |
| TRIM32 | 22954 | 1 |  | 2 |  |
| AIF | 9131 |  |  | 2 |  |
| caspase-1 inhibitor | C0168318 |  |  | 2 |  |
| Caspase-7 | 840 | 1 |  | 2 |  |
| CPIB | 1476 |  |  | 2 |  |
| cell receptors | C0034800 | 2 | 2 | 2 | 1 |
| CLint | 9685 | 1 |  | 2 |  |
| calmodulin-dependent protein kinases | C0006776 |  | 1 | 2 | 2 |
| prolyl endopeptidase | 5550 | 1 | 1 | 2 | 2 |
| signaling pathways. alpha-syn protein | C1335962 |  |  | 2 |  |
| transcriptional co-activator | C1336776 |  | 3 | 2 | 0.666666666666667 |
| TGF-beta | 59086 |  | 1 | 2 | 2 |
| IL-10 | 25325 |  |  | 2 |  |
| cha | 10732 |  |  | 2 |  |
| MPO | 17523 |  | 1 | 2 | 2 |
| alpha-galactosidase A | 2717 | 1 |  | 2 |  |
| ADCY5 | 111 |  |  | 2 |  |
| RAI | 2657 |  |  | 2 |  |
| CTLA4-Ig | 1493 |  |  | 2 |  |
| D3could | 1735 |  |  | 2 |  |
| extracellular signal-regulated kinase (ERK) 1 | C0082529 | 5 | 2 | 2 | 1 |
| synaptosomal-associated protein 25 | C0075689 |  | 1 | 2 | 2 |
| per -1 | 5187 |  | 1 | 2 | 2 |
| ICH, 2 | 837 |  |  | 2 |  |
| DBP | 24309 |  |  | 2 |  |
| beta-secretase | C1454853 |  |  | 2 |  |
| Clk1 | 1195 |  |  | 2 |  |
| COQ7 | 12850 |  |  | 2 |  |
| Clk1 | 12747 |  |  | 2 |  |
| KATP channels | C1955862 |  |  | 2 |  |
| NR1 | 24408 |  |  | 2 |  |
| alpha-galactosidase | C0002268 |  |  | 2 |  |
| Angiotensin-II | 183 | 1 |  | 2 |  |
| ASK1 | 4217 |  | 1 | 2 | 2 |
| ROR | 100885779 |  |  | 2 |  |
| recessive Parkinson's genes | C0017361 |  | 2 | 2 | 1 |
| pts | 5805 |  | 1 | 2 | 2 |
| Orexin-A | C0671870 |  | 1 | 2 | 2 |
| Orexin 1 receptor | 3061 |  | 1 | 2 | 2 |
| SMPD1 | 6609 |  |  | 2 |  |
| sphingomyelin phosphodiesterase 1 | 6609 |  |  | 2 |  |
| SMPD1and | 6609 |  |  | 2 |  |
| Toll like receptor 2 | 24088 |  | 2 | 2 | 1 |
| MyD88 | 17874 |  |  | 2 |  |
| different 3 | C3542919 | 2 |  | 2 |  |
| splash | 26279 |  |  | 2 |  |
| Nod1 | 10392 |  |  | 2 |  |
| DAP | 1611 |  |  | 2 |  |
| CD200R | 131450 |  |  | 2 |  |
| AGU | 175 |  |  | 2 |  |
| GLA | 2717 | 1 |  | 2 |  |
| alpha-Gal | C0002268 |  |  | 2 |  |
| alpha-Gal | 2717 | 1 |  | 2 |  |
| YAP | 10413 |  |  | 2 |  |
| D2-NTR1 | 1734 | 1 | 2 | 2 | 1 |
| alphaS protein | C2362546 |  | 2 | 2 | 1 |
| mitochondrial calcium uniporter | C0054487 |  |  | 2 |  |
| voltage dependent anion channel 1 | C0753015 |  | 1 | 2 | 2 |
| thyroid hormones | C0040135 | 1 | 1 | 2 | 2 |
| davunetide | C0966140 |  |  | 2 |  |
| guanosine triphosphate cyclohydrolase-1 | C0205775 | 1 | 2 | 2 | 1 |
| M3 receptors | C0289799 |  |  | 2 |  |
| ct2 | 85413 |  |  | 2 |  |
| Ape1 | C0014230 |  | 1 | 2 | 2 |
| A2A-D2 | 1734 | 1 | 2 | 2 | 1 |
| synthetic D2 peptide | C0597551 | 3 | 2 | 2 | 1 |
| Transient receptor potential canonical (TRPC) channels | C1563722 |  |  | 2 |  |
| PTP1B | 5770 |  | 1 | 2 | 2 |
| SLC1A4 | 6509 |  |  | 2 |  |
| mutant torsinADeltaGAG protein | C1564139 | 3 | 1 | 2 | 2 |
| blocking antibody | C0282682 |  |  | 2 |  |
| caspase 9 | 12371 |  | 1 | 2 | 2 |
| P = . | 4948 |  |  | 2 |  |
| D2-MSN | 1734 | 1 | 2 | 2 | 1 |
| Adenylate Cyclase 5 | 111 |  |  | 2 |  |
| CSF tau proteins | C0007810 | 1 | 3 | 2 | 0.666666666666667 |
| AADC | 13195 |  |  | 2 |  |
| IN-1 | 16258 |  |  | 2 |  |
| inhibitors at 1 | 5502 | 1 | 2 | 2 | 1 |
| Phox2b | 364152 |  |  | 2 |  |
| Wnt | 114487 |  |  | 2 |  |
| Wnt-3a | 303181 |  |  | 2 |  |
| Pitx-3 | 29609 | 5 |  | 2 |  |
| 1-Oct | 5451 |  |  | 2 |  |
| DNMT3a | 1788 |  |  | 2 |  |
| DNMTs | C0012873 |  |  | 2 |  |
| glyoxalase III | C0293930 |  |  | 2 |  |
| IL-10 | 16153 |  |  | 2 |  |
| ADORA1 | 134 |  | 1 | 2 | 2 |
| Calnuc | 4924 |  |  | 2 |  |
| alpha-crystallin | C0002257 |  |  | 2 |  |
| alcohol dehydrogenase | 10327 |  | 1 | 2 | 2 |
| pH4 | 54681 | 3 | 1 | 2 | 2 |
| GPR37 | 14763 | 1 |  | 2 |  |
| orphan G protein-coupled receptor | 55890 |  |  | 2 |  |
| vesicular glutamate transporter 2 | C0969321 |  |  | 2 |  |
| SIPA1L2 | 57568 |  |  | 2 |  |
| CDR | 862 |  |  | 2 |  |
| XDP | 6908 | 4 | 1 | 2 | 2 |
| NFkappaB p50 subunit | 958 | 3 | 2 | 2 | 1 |
| MC1R | 4157 |  |  | 2 |  |
| melanocortin 1 receptor | 4157 |  |  | 2 |  |
| VEGF | 83785 |  |  | 2 |  |
| HDL | 3290 |  |  | 2 |  |
| different misfolded proteins | C0082213 |  | 1 | 2 | 2 |
| GABA/tCr | C0016904 | 3 | 3 | 2 | 0.666666666666667 |
| BCL2L1 | 598 | 3 | 4 | 2 | 0.5 |
| DNA methyltransferase | 1786 |  |  | 2 |  |
| DNMT1 | 1786 |  |  | 2 |  |
| RelA | 5970 | 4 | 4 | 2 | 0.5 |
| Chrna7 | 11441 |  | 1 | 2 | 2 |
| CHRNA7 gene | 1139 |  |  | 2 |  |
| CHRNA7 | 1139 |  |  | 2 |  |
| alpha secretase | C0379526 |  |  | 2 |  |
| Mmp10 | 4319 |  |  | 2 |  |
| ubiquitinated MOM proteins | C1956096 | 1 | 1 | 2 | 2 |
| miR-16 | 51573 |  |  | 2 |  |
| miR-21 | 406991 |  |  | 2 |  |
| PINK1 gene | C1334324 | 3 | 3 | 2 | 0.666666666666667 |
| pS129 | 2843875 |  | 1 | 2 | 2 |
| zinc finger protein 746 | 155061 |  |  | 2 |  |
| AIMP2 | 7965 |  |  | 2 |  |
| Bip | 14828 |  | 1 | 2 | 2 |
| endothelial adhesion molecules | 90952 |  |  | 2 |  |
| CYLD | 1540 |  |  | 2 |  |
| IkappaBalpha | 25493 |  |  | 2 |  |
| CYLD | 312937 |  |  | 2 |  |
| FGF)-2 | C0380603 | 1 |  | 2 |  |
| Nestin | 18008 |  |  | 2 |  |
| task annually over 3 | 51305 |  |  | 2 |  |
| FOXA2 | 25099 |  |  | 2 |  |
| p-TrkB | C3853697 | 1 |  | 2 |  |
| tyrosinase-related protein-1 | 7306 | 1 | 1 | 2 | 2 |
| TRP-1 | 7306 | 1 | 1 | 2 | 2 |
| TRP-2 | 7306 | 1 | 1 | 2 | 2 |
| FAAH | 14073 |  |  | 2 |  |
| TRAP-1 | 10131 |  | 1 | 2 | 2 |
| MAO-A/B ( | C0026456 |  | 3 | 2 | 0.666666666666667 |
| MAO-A/B | C0026456 |  | 3 | 2 | 0.666666666666667 |
| LHb | 25329 |  |  | 2 |  |
| 2D-IR | 3643 | 3 | 2 | 2 | 1 |
| SPN | 6693 |  |  | 2 |  |
| muscarinic M3 receptor | C0289799 |  |  | 2 |  |
| calcium/depolarization-dependent presynaptic glutamate | C0556252 |  | 1 | 2 | 2 |
| GTP-cyclohydrolase I | C0205775 | 1 | 2 | 2 | 1 |
| parkin interacting substrate | 155061 |  |  | 2 |  |
| kappa-light-chain | C0021036 |  |  | 2 |  |
| aft | 55783 |  |  | 2 |  |
| PARP-1 | 25591 |  |  | 2 |  |
| PARP1 | 25591 |  |  | 2 |  |
| TfR | 7037 |  | 1 | 2 | 2 |
| TfRs | 7037 |  | 1 | 2 | 2 |
| serotonin 2A receptors | C0289174 |  |  | 2 |  |
| HLA-DRA/HLA-DRB5 | 3127 |  | 1 | 2 | 2 |
| calcium binding protein | C0006732 | 1 | 1 | 2 | 2 |
| VAPB-PTPIP51 | 9217 |  |  | 2 |  |
| VAPB | 9217 |  |  | 2 |  |
| PTPIP51 | 55177 |  |  | 2 |  |
| synthetic linker protein | C0597552 |  |  | 2 |  |
| pore-forming protein | C0071728 |  | 2 | 2 | 1 |
| perforin | 5551 |  | 1 | 2 | 2 |
| Thy-1 | 21838 | 3 | 5 | 2 | 0.4 |
| mouse protein | C1334805 |  |  | 2 |  |
| Syn | 41247 |  |  | 2 |  |
| task 3 | 51305 |  |  | 2 |  |
| Nedd4-2 | 83814 |  |  | 2 |  |
| RIC3 | 79608 |  |  | 2 |  |
| RIC3 gene | 79608 |  |  | 2 |  |
| mouse brain protein | C1334805 |  |  | 2 |  |
| PLK | 5347 |  | 1 | 2 | 2 |
| neural proteins | C0027759 | 2 | 2 | 2 | 1 |
| citrate synthase | 1431 |  |  | 2 |  |
| mitochondrial enzymes citrate synthase | 1431 |  |  | 2 |  |
| CS | 1431 |  |  | 2 |  |
| caspase9 | 12371 |  | 1 | 2 | 2 |
| RIL | 8572 |  |  | 2 |  |
| tryptophan hydroxylase 2 | 121278 |  |  | 2 |  |
| TPH2 | 121278 |  |  | 2 |  |
| norepinephrine transporter | C0132800 |  | 1 | 2 | 2 |
| IL-17A | 3605 |  | 1 | 2 | 2 |
| RNase 5 | 283 |  | 1 | 2 | 2 |
| heme oxygenase | C0018969 |  |  | 2 |  |
| alpha-syn protein | C2362546 |  | 2 | 2 | 1 |
| Rab7 | 7879 |  |  | 2 |  |
| TREM2 | 83433 |  |  | 2 |  |
| ATF-6 | 22926 |  | 1 | 2 | 2 |
| TMEM175 | 84286 |  |  | 2 |  |
| Transmembrane protein 175 | 84286 |  |  | 2 |  |
| CG) | 1511 |  |  | 2 |  |
| Vps34 | 5289 |  |  | 2 |  |
| tryptic | C0041236 |  | 3 | 2 | 0.666666666666667 |
| P2Y6 receptor | 5031 |  |  | 2 |  |
| P2Y6R | 5031 |  |  | 2 |  |
| Nucleobindin 1 | 4924 |  |  | 2 |  |
| NUCB1 | 4924 |  |  | 2 |  |
| Rab7A | 7879 |  |  | 2 |  |
| SPE | C0764874 |  | 1 | 2 | 2 |
| GCS | C0163410 |  |  | 2 |  |
| caspase 7 | 840 | 1 |  | 2 |  |
| UDP-glucose ceramide glucosyltransferase | C0163410 |  |  | 2 |  |
| ATG7 genes | 10533 | 1 | 1 | 2 | 2 |
| angiotensinogen | 183 | 1 |  | 2 |  |
| ERbeta | 25149 |  |  | 2 |  |
| interleukin 6 receptor | 3570 |  |  | 2 |  |
| 1-Mar | 55016 |  |  | 2 |  |
| TOMM40 gene | 10452 |  |  | 2 |  |
| TOMM40 | 10452 |  |  | 2 |  |
| Bax-inhibitor-1 | 38936 |  |  | 2 |  |
| Bax inhibitor-1 | 38936 |  |  | 2 |  |
| BI-1 | 38936 |  |  | 2 |  |
| Arc | 23237 |  |  | 2 |  |
| activity-regulated cytoskeleton-associated protein | 23237 |  |  | 2 |  |
| monoamine oxidase (MAO)-B | C0026456 |  | 3 | 2 | 0.666666666666667 |
| synaptic vesicle glycoprotein 2 | 9900 |  |  | 2 |  |
| SV2 | 9900 |  |  | 2 |  |
| chitinase | C0008145 |  |  | 2 |  |
| caspase-1 | 25166 | 1 |  | 2 |  |
| brain at 2 | 5454 |  |  | 2 |  |
| ATR-IR | 545 |  | 1 | 2 | 2 |
| RIP1 | 7386 |  |  | 2 |  |
| RIP3 | 11035 |  |  | 2 |  |
| Ag2S | 185 |  |  | 2 |  |
| beta-arrestin 2 | 409 | 1 |  | 2 |  |
| Syt | 6760 |  |  | 2 |  |
| Syt I | 20979 |  |  | 2 |  |
| I protein | 3283 |  |  | 2 |  |
| TRPV1 | 193034 |  |  | 2 |  |
| FP-(+) | C3541238 |  |  | 2 |  |
| CYP1B1 | 1545 |  |  | 2 |  |
| TRPC1-STIM1 | 7220 |  | 1 | 2 | 2 |
| STIM1 | 6786 |  |  | 2 |  |
| A170 | 8878 |  | 3 | 2 | 0.666666666666667 |
| CD47 | 961 |  |  | 2 |  |
| chymotrypsin | C0008742 |  |  | 2 |  |
| beta-lactoglobulin | C0005248 |  |  | 2 |  |
| beta-LG | C0005248 |  |  | 2 |  |
| RFP | 5987 |  |  | 2 |  |
| aquaporin-4 | 361 | 1 | 2 | 2 | 1 |
| aquaporins | C0599635 | 1 | 1 | 2 | 2 |
| aquaporin | C0599635 | 1 | 1 | 2 | 2 |
| MC1R gene | 4157 |  |  | 2 |  |
| brain sections at 2 | 5454 |  |  | 2 |  |
| guanosine triphosphate cyclohydrolase I | C0205775 | 1 | 2 | 2 | 1 |
| LRRK1 | 233328 |  |  | 2 |  |
| DNAJB6 | 10049 |  |  | 2 |  |
| Nix | 665 |  |  | 2 |  |
| Nip3-like protein X | 665 |  |  | 2 |  |
| MAC-1 receptor | C0079785 |  |  | 2 |  |
| orphan GPCRs | 55890 |  |  | 2 |  |
| DOPAL | C1517852 |  |  | 2 |  |
| platelet-derived growth factor | C0032200 |  |  | 2 |  |
| TPH2 gene | 121278 |  |  | 2 |  |
| Thyroid gland hormone | C0040135 | 1 | 1 | 2 | 2 |
| PGI | 633 |  | 1 | 2 | 2 |
| nuanced | 23224 |  |  | 2 |  |
| Spr gene | 6697 |  |  | 2 |  |
| Spr-/- | 6697 |  |  | 2 |  |
| Spr-/ | 6697 |  |  | 2 |  |
| aldehyde dehydrogenase-1 | C0538135 | 1 | 2 | 2 | 1 |
| Aldh1a1 genes | 216 |  | 2 | 2 | 1 |
| IgG | C0020852 |  | 1 | 2 | 2 |
| GIP | 2695 |  |  | 2 |  |
| Arg1 | 11846 |  |  | 2 |  |
| Ym1 | 12655 |  |  | 2 |  |
| mitochondrial membrane protein | C1180069 |  |  | 2 |  |
| protein tyrosine phosphatase-interacting protein 51 | 55177 |  |  | 2 |  |
| CD157 | 12182 |  |  | 2 |  |
| Recessive F-box protein 7 gene | C0017361 |  | 2 | 2 | 1 |
| ASA | C0007804 |  |  | 2 |  |
| b = 0.50 | 2596 |  |  | 2 |  |
| DRB1 | 129831 |  |  | 2 |  |
| DQB1 | 3119 |  |  | 2 |  |
| HLA genes | C0599797 |  | 1 | 2 | 2 |
| metallothionein-3 | 4504 | 1 | 1 | 2 | 2 |
| microtubule-associated protein 1A/1B | C0250759 |  |  | 2 |  |
| p70S6K | 6198 |  |  | 2 |  |
| ribosomal DNA | C0035899 |  |  | 2 |  |
| rDNA | C0035899 |  |  | 2 |  |
| Gad1 | 14415 |  |  | 2 |  |
| Ala2GIP | 2695 |  |  | 2 |  |
| Liver X receptors | C1612060 | 2 |  | 2 |  |
| HDACi | C1512474 | 1 | 1 | 2 | 2 |
| Sirtuin-3 | C2720169 |  |  | 2 |  |
| Indo- | 3620 |  |  | 2 |  |
| recombinant human EPO | 2056 | 1 | 2 | 2 | 1 |
| Sulfotransferase | C0038769 |  |  | 2 |  |
| SLP-2 | 30968 |  |  | 2 |  |
| Stomatin-like protein 2 | 30968 |  |  | 2 |  |
| MIR4697 | 100616119 |  |  | 2 |  |
| gamma-glutamylcysteine synthetase | C0017037 | 4 | 1 | 2 | 2 |
| HLA-DQB1 | 3119 |  |  | 2 |  |
| LPO | 76113 |  |  | 2 |  |
| JAK2 | 24514 |  |  | 2 |  |
| receptor for advanced glycation end products | 177 |  |  | 2 |  |
| glucagon-like peptide-1 (GLP-1) receptor | C0378073 | 1 | 1 | 2 | 2 |
| intercellular adhesion molecule-1 | 3383 |  | 1 | 2 | 2 |
| ICAM-1 | 3383 |  | 1 | 2 | 2 |
| regulatory protein | C0815047 | 3 | 2 | 2 | 1 |
| protein B | C0377800 |  |  | 2 |  |
| Integrator | C0309311 |  | 1 | 2 | 2 |
| N-methyl-d-aspartate glutamate receptor | 24408 |  |  | 2 |  |
| AM630 | 3171066 |  |  | 2 |  |
| caspase 1 | 25166 | 1 |  | 2 |  |
| mBP | 17196 |  |  | 2 |  |
| peroxisome proliferator-activated receptor-gamma coactivator 1alpha | 83516 |  | 2 | 2 | 1 |
| SANS | 124590 |  |  | 2 |  |
| HLA-MHC-II genes | C0599797 |  | 1 | 2 | 2 |
| SREBP-1 | 6720 |  |  | 2 |  |
| eta2 | 11201 |  |  | 2 |  |
| gamma-aminobutyric acid (GABA)-A receptor | C0206518 |  | 4 | 2 | 0.5 |
| TrkB receptor | C0084873 |  | 1 | 2 | 2 |
| MT-3 | 4504 | 1 | 1 | 2 | 2 |
| MTs | C0025545 | 3 | 1 | 2 | 2 |
| ENK | C0014298 | 2 |  | 2 |  |
| hemoglobin delta | 3045 |  |  | 2 |  |
| interleukin-13 receptor alpha1 | 3597 |  | 1 | 2 | 2 |
| interleukin-13 | 16163 |  | 1 | 2 | 2 |
| SQSTM-1 | 8878 |  | 3 | 2 | 0.666666666666667 |
| Sequestosome-1 | 8878 |  | 3 | 2 | 0.666666666666667 |
| Rad18 | 56852 |  |  | 2 |  |
| PINK1 | 298575 |  |  | 2 |  |
| TOM40 | 10452 |  |  | 2 |  |
| PCSK9 | 255738 |  |  | 2 |  |
| HMGCR | 3156 | 1 |  | 2 |  |
| 3-hydroxy-3-methylglutaryl-CoA reductase | C0020374 |  |  | 2 |  |
| recessive Parkinson's disease genes | C0017361 |  | 2 | 2 | 1 |
| RANTES | 20304 |  |  | 2 |  |
| metagenome | C2717745 |  |  | 2 |  |
| metagenomes | C2717745 |  |  | 2 |  |
| GPER | 76854 |  |  | 2 |  |
| NFM | 4741 |  |  | 2 |  |
| pilot | 1960 |  | 1 | 2 | 2 |
| IL-6R | 3570 |  |  | 2 |  |
| Glucagon-Like Peptide-1 Receptor | C0378073 | 1 | 1 | 2 | 2 |
| P1-P21 | 5551 |  | 1 | 2 | 2 |
| NGL | 2064 | 1 |  | 2 |  |
| TrkB inhibitor | C3537035 |  | 1 | 2 | 2 |
| NF-L | 18039 |  |  | 2 |  |
| Bmal1 | 29657 |  |  | 2 |  |
| Per2 | 63840 |  |  | 2 |  |
| SYN | 6855 | 2 | 3 | 2 | 0.666666666666667 |
| GR | C0034809 |  | 1 | 2 | 2 |
| Ccl5 | 20304 |  |  | 2 |  |
| LS-1 | 10103 |  |  | 2 |  |
| TMEM106B genes | 54664 |  |  | 2 |  |
| transmembrane Protein 106B | 54664 |  |  | 2 |  |
| TMEM106B | 54664 |  |  | 2 |  |
| heme-oxygenase | C0018969 |  |  | 2 |  |
| myelin basic protein | 17196 |  |  | 2 |  |
| synthetic protein | C0597552 |  |  | 2 |  |
| Phospholipase D | C0031670 |  |  | 2 |  |
| PLD | 2822 |  |  | 2 |  |
| HBD | 3045 |  |  | 2 |  |
| dopamine D3 receptor | 1735 |  |  | 2 |  |
| epsilon sarcoglycan | 8910 |  |  | 2 |  |
| rRNA gene | C0035899 |  |  | 2 |  |
| TRPC Channels | C1563722 |  |  | 2 |  |
| dream | 30818 |  |  | 2 |  |
| BST1 | 12182 |  |  | 2 |  |
| Cav1.2 | 12288 |  |  | 2 |  |
| Cav1.3 | 12289 |  | 1 | 2 | 2 |
| Angelman syndrome | 7337 |  |  | 2 |  |
| Drp1 | 114114 |  |  | 2 |  |
| dynamin-related protein-1 | 114114 |  |  | 2 |  |
| toll-like receptor 2 | 24088 |  | 2 | 2 | 1 |
| voltage-dependent anion channel | C1506024 | 1 | 1 | 2 | 2 |
| mutant polyQ Huntingtin (polyQ-Htt) protein | C1564139 | 3 | 1 | 2 | 2 |
| beta-N-acetylglucosaminidase | 10724 |  |  | 2 |  |
| OGA | 10724 |  |  | 2 |  |
| Nogo-receptor | 65078 |  | 1 | 2 | 2 |
| NEP1-40 | 10436 |  |  | 2 |  |
| MiR-124-3p | 406909 |  |  | 2 |  |
| annexinA5 | 308 | 2 | 3 | 2 | 0.666666666666667 |
| ANXA5 | 308 | 2 | 3 | 2 | 0.666666666666667 |
| Homer | 9456 |  |  | 2 |  |
| alpha7 nicotinic receptor | 11441 |  | 1 | 2 | 2 |
| 2 - HY | 8284 |  |  | 2 |  |
| MAO-B I | C0014442 | 1 |  | 2 |  |
| BGN | 633 |  | 1 | 2 | 2 |
| CLU | 1191 |  | 1 | 2 | 2 |
| enzyme- | C0014442 | 1 |  | 2 |  |
| endothelial nitric oxide synthase | 4846 | 3 | 2 | 2 | 1 |
| transcriptional activators | C1336776 |  | 3 | 2 | 0.666666666666667 |
| YKL-40 | 1116 |  |  | 2 |  |
| Dopa decarboxylase | 35190 |  |  | 2 |  |
| Ddc | 35190 |  |  | 2 |  |
| cholinesterase 3 gene | 590 | 1 |  | 2 |  |
| Mutant pathogenic proteins | C1564139 | 3 | 1 | 2 | 2 |
| L-proline | C0033382 | 2 | 1 | 2 | 2 |
| L-valine | C0042285 | 4 | 4 | 2 | 0.5 |
| glutathione peroxidase 4 | 2879 |  | 1 | 2 | 2 |
| IkappaB-alpha | 18035 |  | 2 | 2 | 1 |
| NF-M | 4741 |  |  | 2 |  |
| Phosphoglycerate Kinase 1 | 5230 |  |  | 2 |  |
| human phosphoglycerate kinase 1 | 5230 |  |  | 2 |  |
| hPgk1 | 5230 |  |  | 2 |  |
| Pgk1 | 5230 |  |  | 2 |  |
| TH1-S31A | 51497 |  | 1 | 2 | 2 |
| TH1-S31E | 51497 |  | 1 | 2 | 2 |
| storage proteins | C0815045 |  | 1 | 2 | 2 |
| IRP1 | 48 |  | 2 | 2 | 1 |
| SMN2 gene | 6607 | 1 | 1 | 2 | 2 |
| ACTH | 5443 | 1 |  | 2 |  |
| neurogranin | 4900 |  |  | 2 |  |
| cerebrospinal fluid synaptic proteins | C0007810 | 1 | 3 | 2 | 0.666666666666667 |
| granulocyte-macrophage colony-stimulating factor) | 1437 |  |  | 2 |  |
| CTLA-4 | 1493 |  |  | 2 |  |
| cytotoxic T-lymphocyte-associated protein 4 | 1493 |  |  | 2 |  |
| PGK-1 | 5230 |  |  | 2 |  |
| large genetic | 9215 |  | 1 | 2 | 2 |
| beta strand | C1706935 | 3 | 5 | 2 | 0.4 |
| nerve cell protein | C0027759 | 2 | 2 | 2 | 1 |
| Alpha-Synuclein-Interacting Protein | C3541909 | 8 | 5 | 2 | 0.4 |
| gamma-aminobutyric acid A receptor | C0206518 |  | 4 | 2 | 0.5 |
| sirtuin-1 | 93759 | 1 | 2 | 2 | 1 |
| Dickkopf-1 | 22943 |  |  | 2 |  |
| Dkk1 | 22943 |  |  | 2 |  |
| XIST | 7503 |  |  | 2 |  |
| DBS-STN | C0013023 | 1 |  | 2 |  |
| HY] | 8284 |  |  | 2 |  |
| AEP | 5641 |  |  | 2 |  |
| AEP's | 5641 |  |  | 2 |  |
| beta-alanine | C0000392 | 1 |  | 2 |  |
| Insulin-like growth factor I | C0021665 | 1 | 2 | 2 | 1 |
| IGF-I | 24482 | 2 | 1 | 2 | 2 |
| E-selectin | 6401 |  |  | 2 |  |
| CYP2D6 gene | 1565 | 5 | 3 | 2 | 0.666666666666667 |
| debrisoquine 4-hydroxylase | 1565 | 5 | 3 | 2 | 0.666666666666667 |
| Slc30a10 | 226781 |  |  | 2 |  |
| MS ( | 4397 | 1 |  | 2 |  |
| metabotropic glutamate receptors 4 | C0667286 | 2 | 3 | 2 | 0.666666666666667 |
| metabotropic glutamate receptors (mGluRs) 4 | C0667286 | 2 | 3 | 2 | 0.666666666666667 |
| Integrin-linked kinase | C0385469 |  |  | 2 |  |
| myeloperoxidase | C0027021 | 1 | 2 | 2 | 1 |
| orexin-1 receptor | 3061 |  | 1 | 2 | 2 |
| OX-A | C0671870 |  | 1 | 2 | 2 |
| orexin-1 receptors | 3061 |  | 1 | 2 | 2 |
| SK) | 22943 |  |  | 2 |  |
| synthetic regulatory peptides | C0597551 | 3 | 2 | 2 | 1 |
| D3 dopamine receptor-selective compounds | 1735 |  |  | 2 |  |
| MSC-CM | 9242 | 1 |  | 2 |  |
| retromer-complex protein | 51699 |  |  | 2 |  |
| Opioid K receptor | C0034801 |  |  | 2 |  |
| caspase 4 | 837 |  |  | 2 |  |
| pseudogene | C0033799 |  |  | 2 |  |
| GBAP1 | 2630 |  |  | 2 |  |
| PM2.5-10 | 1262040 |  |  | 2 |  |
| nucleotide-binding oligomerization domain (NOD)-like receptor protein 1 | 10392 |  |  | 2 |  |
| per 1 | 5187 |  | 1 | 2 | 2 |
| sulfotransferases | C0038769 |  |  | 2 |  |
| greater genetic | 9215 |  | 1 | 2 | 2 |
| protectins | C0054966 | 1 |  | 2 |  |
| P2Y6 | 5031 |  |  | 2 |  |
| P2Y6 purinergic receptor | 5031 |  |  | 2 |  |
| JAK | C0597721 |  |  | 2 |  |
| PDE 4 | 5141 | 1 |  | 2 |  |
| signaling phospho-proteins | C1335962 |  |  | 2 |  |
| PrPres | C0074204 |  | 1 | 2 | 2 |
| PrPC | 19122 | 1 | 3 | 2 | 0.666666666666667 |
| TOMs | 24148 |  | 2 | 2 | 1 |
| interleukin-18 | 16173 |  |  | 2 |  |
| IL-18 | 16173 |  |  | 2 |  |
| kinase two gene | C1334324 | 3 | 3 | 2 | 0.666666666666667 |
| RING1UBL | 6015 | 1 |  | 2 |  |
| Grp94 | 7184 |  |  | 2 |  |
| Ube3a | 7337 |  |  | 2 |  |
| Nfkbia | 4792 | 1 | 1 | 2 | 2 |
| chitinase-like 3 | 12655 |  |  | 2 |  |
| Arg 1 | 11846 |  |  | 2 |  |
| MRC1 | 4360 |  | 1 | 2 | 2 |
| NAD(P)H: quinone oxidoreductase 1 | 18104 |  | 1 | 2 | 2 |
| glutamylcysteine ligase | C0017037 | 4 | 1 | 2 | 2 |
| BCL2L11 | 10018 | 1 | 3 | 2 | 0.666666666666667 |
| BCL2L11were | 10018 | 1 | 3 | 2 | 0.666666666666667 |
| estrogen receptor-related receptors | C0034804 | 2 | 4 | 2 | 0.5 |
| interferon | C0021747 |  |  | 2 |  |
| gamma-aminobutyric acid type-A (GABAA) receptor | C0206518 |  | 4 | 2 | 0.5 |
| KATP) channels | C1955862 |  |  | 2 |  |
| gamma-aminobutyric (GABA) acid | C0016904 | 3 | 3 | 2 | 0.666666666666667 |
| CHAs | 10732 |  |  | 2 |  |
| dopa decarboxylase | 24311 | 1 | 1 | 2 | 2 |
| Klotho | 9365 |  |  | 2 |  |
| NMDAR | 14810 |  |  | 2 |  |
| FOXP2 | 93986 |  |  | 2 |  |
| FOXP2's | 93986 |  |  | 2 |  |
| TH17 | 51497 |  | 1 | 2 | 2 |
| Aromatic amino acids | C0301713 | 1 |  | 2 |  |
| Janus kinase | C0597721 |  |  | 2 |  |
| monoamine oxidase inhibitor-B | C0026456 |  | 3 | 2 | 0.666666666666667 |
| tCr | C0010286 | 1 |  | 2 |  |
| DNA methyltransferase (DNMT) 3a | 1788 |  |  | 2 |  |
| CSMD1 | 64478 |  |  | 2 |  |
| CSMD1 gene | 64478 |  |  | 2 |  |
| Std | 6822 |  |  | 2 |  |
| Src | 83805 |  | 1 | 2 | 2 |
| NF-kB p65 subunit | 5970 | 4 | 4 | 2 | 0.5 |
| caspase-12 | 156117 |  |  | 2 |  |
| caspase 12 | 156117 |  |  | 2 |  |
| HDAC6 inhibitor | C1512474 | 1 | 1 | 2 | 2 |
| GluN2A | 24409 | 1 | 1 | 2 | 2 |
| GluN2B | 24410 | 2 | 3 | 2 | 0.666666666666667 |
| IDH | 3417 |  | 3 | 2 | 0.666666666666667 |
| synthetic alphaS peptides | C0597551 | 3 | 2 | 2 | 1 |
| death-associated protein | 1611 |  |  | 2 |  |
| DnaJ protein | 10049 |  |  | 2 |  |
| hepcidin | C0966897 |  |  | 2 |  |
| alpha-synuclein | 102119369 |  |  | 2 |  |
| BChE | 590 | 1 |  | 2 |  |
| Src kinase family | C0282625 |  |  | 2 |  |
| Cerebrolysin | C0055108 |  |  | 2 |  |
| Ulk 1 | 8408 |  | 1 | 2 | 2 |
| L-ferritin | C0391764 |  | 1 | 2 | 2 |
| DDR | 780 |  |  | 2 |  |
| G protein-coupled receptor 37 | 14763 | 1 |  | 2 |  |
| beta-hexosaminidase | 10724 |  |  | 2 |  |
| IFN- | 3439 | 1 |  | 2 |  |
| ASAH1 | 427 |  |  | 2 |  |
| acid ceramidase 1 | 427 |  |  | 2 |  |
| voltage-dependent anion channel 1 | C0753015 |  | 1 | 2 | 2 |
| Colony Stimulating Factor-1 | C0079784 |  |  | 2 |  |
| Interleukin-34 | 146433 |  |  | 2 |  |
| IL-34 | 146433 |  |  | 2 |  |
| cJun | 16476 |  | 2 | 2 | 1 |
| NADPH oxidase 2 | 1536 |  | 2 | 2 | 1 |
| Synaptotagmin 1 | 20979 |  |  | 2 |  |
| beta2-Adrenoreceptor | 154 |  | 1 | 2 | 2 |
| beta2AR | 154 |  | 1 | 2 | 2 |
| Rab12 | 201475 |  |  | 2 |  |
| lncRNA human urothelial carcinoma associated 1 | 7503 |  |  | 2 |  |
| DDR1 | 780 |  |  | 2 |  |
| delta 0-3 | C3542919 | 2 |  | 2 |  |
| receptor interacting protein kinase (RIPK) 3 | 11035 |  |  | 2 |  |
| SGCE | 8910 |  |  | 2 |  |
| Apps | 1508 | 1 |  | 2 |  |
| adenosine A1 receptor | 134 |  | 1 | 2 | 2 |
| adenosine A1 receptors | 134 |  | 1 | 2 | 2 |
| PHP | 427 |  |  | 2 |  |
| POP | C0072132 |  | 1 | 2 | 2 |
| N100-P200 | 10565 | 1 |  | 2 |  |
| P200-N200 | 10565 | 1 |  | 2 |  |
| apoJ | 1191 |  | 1 | 2 | 2 |
| apoB | 338 |  | 1 | 2 | 2 |
| ASK1-MKK3 | 4217 |  | 1 | 2 | 2 |
| opioid-receptor | C0034801 |  |  | 2 |  |
| A1AR | 134 |  | 1 | 2 | 2 |
| gene Transcription Factor | C0040648 | 2 | 4 | 2 | 0.5 |
| mTF | C0040648 | 2 | 4 | 2 | 0.5 |
| SMN2 genes | 6607 | 1 | 1 | 2 | 2 |
| MR + | 4360 |  | 1 | 2 | 2 |
| TF | C0040648 | 2 | 4 | 2 | 0.5 |
| biocatalysts | C0014442 | 1 |  | 2 |  |
| blocking antibodies | C0282682 |  |  | 2 |  |
| polymerase- | 100616102 | 1 |  | 2 |  |
| COX4I1 | 1327 |  |  | 2 |  |
| adenosine monophosphate kinase | C0001464 |  |  | 2 |  |
| DiY | C0058505 | 2 | 1 | 2 | 2 |
| protein kinase isoforms | C0597298 | 1 | 2 | 2 | 1 |
| i.p | 3283 |  |  | 2 |  |
| alpha-Synuclein (alpha-Syn) protein | C2362546 |  | 2 | 2 | 1 |
| MDCs | C1332016 |  |  | 2 |  |
| cREM | 1390 |  |  | 2 |  |
| lysosomal hydrolase enzyme | C0391845 | 1 | 2 | 2 | 1 |
| HOMER1 | 9456 |  |  | 2 |  |
| UBC | 7316 | 1 |  | 2 |  |
| PLK1 | 5347 |  | 1 | 2 | 2 |
| RPS6KB1 | 6198 |  |  | 2 |  |
| polo-like kinase 1 | 5347 |  | 1 | 2 | 2 |
| IGF-1 | C0021665 | 1 | 2 | 2 | 1 |
| IIS downstream protein | C1335962 |  |  | 2 |  |
| LDLR | 3949 |  | 2 | 2 | 1 |
| ZNF746 | 155061 |  |  | 2 |  |
| protein C | C0033621 |  |  | 2 |  |
| integrin-associated protein | 961 |  |  | 2 |  |
| IAP | 961 |  |  | 2 |  |
| Serine/threonine kinase 39 | 27347 |  | 2 | 2 | 1 |
| AS | 7337 |  |  | 2 |  |
| calmodulin-dependent kinase | C0006776 |  | 1 | 2 | 2 |
| Prnp | 19122 | 1 | 3 | 2 | 0.666666666666667 |
| 15 - CG | 1511 |  |  | 2 |  |
| legumain | 5641 |  |  | 2 |  |
| PDE1 | C0031640 | 2 |  | 2 |  |
| macrophage colony-stimulating factor | C0079784 |  |  | 2 |  |
| interleukin (IL)-34 | 146433 |  |  | 2 |  |
| OFF-DOPA | C0013023 | 1 |  | 2 |  |
| Hexokinases | C0019472 | 1 |  | 2 |  |
| indoleamine-2,3-dioxygenase-1 | 3620 |  |  | 2 |  |
| IDO1 | 3620 |  |  | 2 |  |
| hub' proteins | C3179452 |  |  | 2 |  |
| NFL | 18039 |  |  | 2 |  |
| hub protein | C3179452 |  |  | 2 |  |
| sirtuin 3 | C2720169 |  |  | 2 |  |
| vesicular glutamate transporter-2 | C0969321 |  |  | 2 |  |
| interleukin 13 receptor alpha 1 | 3597 |  | 1 | 2 | 2 |
| aminoacyl tRNA synthetase complex interacting multifunctional protein 2 | 7965 |  |  | 2 |  |
| Complement receptor 3 | C0079785 |  |  | 2 |  |
| PIK3C3 | 5289 |  |  | 2 |  |
| janus kinase 2 | 24514 |  |  | 2 |  |
| DNA methyltransferases | C0012873 |  |  | 2 |  |
| Clk | 1195 |  |  | 2 |  |
| retromer protein | 51699 |  |  | 2 |  |
| sbs | 1540 |  |  | 2 |  |
| protein's C | C0033621 |  |  | 2 |  |
| HT2A | 22954 | 1 |  | 2 |  |
| angiotensin II type 1 receptor | 185 |  |  | 2 |  |
| interleukin-10 | 16153 |  |  | 2 |  |
| protein B ( | C0377800 |  |  | 2 |  |
| LPO | 4025 |  |  | 2 |  |
| neutral amino acid transporter | 6509 |  |  | 2 |  |
| PARP-1 | 11545 | 2 | 1 | 2 | 2 |
| MT | C0025545 | 3 | 1 | 2 | 2 |
| Wnt3a | 303181 |  |  | 2 |  |
| adipocyte fatty acid-binding protein | 2167 |  |  | 2 |  |
| aP2 | 2167 |  |  | 2 |  |
| I item-1. | C0963236 | 2 | 2 | 2 | 1 |
| Ang 1-7 | 283 |  | 1 | 2 | 2 |
| FABP4 | 2167 |  |  | 2 |  |
| Aga | 175 |  |  | 2 |  |
| serotonin 2A receptor | C0289174 |  |  | 2 |  |
| Klotho protein | 9365 |  |  | 2 |  |
| tAIF | 9131 |  |  | 2 |  |
| toxic dipeptide repeat proteins | C0815048 | 3 | 2 | 2 | 1 |
| adenosine monophosphateactivated protein kinase | C0001464 |  |  | 2 |  |
| miR16 | 51573 |  |  | 2 |  |
| Chitinase 3-like 1 | 1116 |  |  | 2 |  |
| LRRK2 gene | C1334324 | 3 | 3 | 2 | 0.666666666666667 |
| SREBF1 | 6720 |  |  | 2 |  |
| aquaporin 4 | 11829 | 1 |  | 1 |  |
| AQP4 | 11829 | 1 |  | 1 |  |
| MSA-P | 4507 | 2 | 1 | 1 | 1 |
| MSAP | 4507 | 2 | 1 | 1 | 1 |
| net) | C3853572 | 2 | 2 | 1 | 0.5 |
| nets | C3853572 | 2 | 2 | 1 | 0.5 |
| PNs | C3853572 | 2 | 2 | 1 | 0.5 |
| PANDA | 101154753 | 1 |  | 1 |  |
| 3-O-methyldopa | C0964304 | 4 | 1 | 1 | 1 |
| CO2 | 717 | 1 | 1 | 1 | 1 |
| lipoamide dehydrogenase | 1738 | 1 |  | 1 |  |
| Peptide hormone | C0597192 | 2 |  | 1 |  |
| Fos | 314322 | 7 | 4 | 1 | 0.25 |
| c-fos | 314322 | 7 | 4 | 1 | 0.25 |
| CTS | 7276 | 2 | 2 | 1 | 0.5 |
| ATTR | 7276 | 2 | 2 | 1 | 0.5 |
| Proteoglycans | C0033692 | 1 |  | 1 |  |
| PGs | C0033692 | 1 |  | 1 |  |
| heart-fatty acid binding protein | 2170 | 1 |  | 1 |  |
| H-FABP | 2170 | 1 |  | 1 |  |
| nitric oxide synthase | C0132555 | 7 | 5 | 1 | 0.2 |
| NOS | C0132555 | 7 | 5 | 1 | 0.2 |
| nitric oxide synthases | C0132555 | 7 | 5 | 1 | 0.2 |
| nitric oxide synthetase | C0132555 | 7 | 5 | 1 | 0.2 |
| nitric-oxide synthase | C0132555 | 7 | 5 | 1 | 0.2 |
| casein kinase II | C0108555 | 1 | 1 | 1 | 1 |
| Hypocretin | C1113688 | 1 | 1 | 1 | 1 |
| orexin | C1113688 | 1 | 1 | 1 | 1 |
| B12 | 4709 | 2 | 3 | 1 | 0.333333333333333 |
| fibroblast growth factor 2 | 2247 | 6 | 2 | 1 | 0.5 |
| FGF-2 | 2247 | 6 | 2 | 1 | 0.5 |
| bFGF | 2247 | 6 | 2 | 1 | 0.5 |
| FGF2 | 2247 | 6 | 2 | 1 | 0.5 |
| Hereditary hemochromatosis | 3077 | 3 | 2 | 1 | 0.5 |
| HFE | 3077 | 3 | 2 | 1 | 0.5 |
| HH | 3077 | 3 | 2 | 1 | 0.5 |
| HFE genes | 3077 | 3 | 2 | 1 | 0.5 |
| HFE gene | 3077 | 3 | 2 | 1 | 0.5 |
| haptoglobin | 3240 | 1 |  | 1 |  |
| Hp | 3240 | 1 |  | 1 |  |
| Bak | 578 | 3 |  | 1 |  |
| OBS | 3952 | 2 | 1 | 1 | 1 |
| Leptin | 3952 | 2 | 1 | 1 | 1 |
| Pol | 100616496 | 1 | 1 | 1 | 1 |
| TdT | 1791 | 1 | 1 | 1 | 1 |
| S-adenosylmethionine | C0036002 | 5 | 2 | 1 | 0.5 |
| SAM | C0036002 | 5 | 2 | 1 | 0.5 |
| ubiquinone oxidoreductase | C0171406 | 4 | 3 | 1 | 0.333333333333333 |
| NADH Coenzyme Q (CoQ) oxidoreductase | C0171406 | 4 | 3 | 1 | 0.333333333333333 |
| tryptophan hydroxylase | C0041252 | 3 | 1 | 1 | 1 |
| synthetic enzyme | C0597548 | 2 | 1 | 1 | 1 |
| adaptor protein | C1135629 | 3 | 3 | 1 | 0.333333333333333 |
| adaptor proteins | C1135629 | 3 | 3 | 1 | 0.333333333333333 |
| Neurosin | 5653 | 2 | 1 | 1 | 1 |
| Kallikrein 6 | 5653 | 2 | 1 | 1 | 1 |
| hk6 | 5653 | 2 | 1 | 1 | 1 |
| Leu | C0023401 | 2 | 7 | 1 | 0.142857142857143 |
| leucine | C0023401 | 2 | 7 | 1 | 0.142857142857143 |
| P22 | 11331 | 1 | 1 | 1 | 1 |
| bAP | 11331 | 1 | 1 | 1 | 1 |
| TH | C0041485 | 1 | 7 | 1 | 0.142857142857143 |
| mitogen | C0018284 | 4 | 3 | 1 | 0.333333333333333 |
| MAPK/ERK | C0018284 | 4 | 3 | 1 | 0.333333333333333 |
| p95 | 4683 | 1 |  | 1 |  |
| cytochrome CYP1A1 | 1543 | 1 | 1 | 1 | 1 |
| p25 | 3934 | 1 | 2 | 1 | 0.5 |
| insects | C0981916 | 1 | 1 | 1 | 1 |
| synphilin | 9627 | 3 | 3 | 1 | 0.333333333333333 |
| synphilin-1 | 9627 | 3 | 3 | 1 | 0.333333333333333 |
| synphilin 1 | 9627 | 3 | 3 | 1 | 0.333333333333333 |
| histamine N-methyltransferase | 3176 | 2 | 2 | 1 | 0.5 |
| HNMT | 3176 | 2 | 2 | 1 | 0.5 |
| fibroblast growth factors | C0016026 | 4 | 3 | 1 | 0.333333333333333 |
| FGF | C0016026 | 4 | 3 | 1 | 0.333333333333333 |
| fibroblast growth factor | C0016026 | 4 | 3 | 1 | 0.333333333333333 |
| FGF) | C0016026 | 4 | 3 | 1 | 0.333333333333333 |
| Fried | 10129 | 1 |  | 1 |  |
| MCP | 822 | 1 |  | 1 |  |
| SCP | 2571 | 1 | 1 | 1 | 1 |
| GRK | C0872383 | 1 | 1 | 1 | 1 |
| GRKs | C0872383 | 1 | 1 | 1 | 1 |
| GH | C0037663 | 3 | 1 | 1 | 1 |
| growth hormone | C0037663 | 3 | 1 | 1 | 1 |
| G protein-coupled receptor kinases | C0872043 | 1 | 1 | 1 | 1 |
| UCH-L1 | 22223 | 3 | 5 | 1 | 0.2 |
| chitotriosidase | C0253005 |  |  | 1 |  |
| MEK1 | 5604 | 3 | 1 | 1 | 1 |
| MEKK1-SEK1 | 5604 | 3 | 1 | 1 | 1 |
| MAPKK1 | 5604 | 3 | 1 | 1 | 1 |
| mGluR2 | 14800 | 2 |  | 1 |  |
| TOF | 2626 | 3 | 1 | 1 | 1 |
| TOF) | 2626 | 3 | 1 | 1 | 1 |
| Cdc37 | 11140 | 2 |  | 1 |  |
| ATP-dependent protease | 51761 | 3 | 4 | 1 | 0.25 |
| ATP-sensitive potassium channels | 51761 | 3 | 4 | 1 | 0.25 |
| nNOS | 4842 | 3 | 3 | 1 | 0.333333333333333 |
| NOS1 | 4842 | 3 | 3 | 1 | 0.333333333333333 |
| NOS | 4842 | 3 | 3 | 1 | 0.333333333333333 |
| ubiquitin carboxy-terminal esterase | C0164005 | 3 | 4 | 1 | 0.25 |
| ubiquitin carboxy-terminal hydrolase- | C0164005 | 3 | 4 | 1 | 0.25 |
| alpha/beta-tubulin | C0002318 | 3 | 2 | 1 | 0.5 |
| alpha-tubulin | C0002318 | 3 | 2 | 1 | 0.5 |
| drp1 | 33445 | 3 | 1 | 1 | 1 |
| NQO1 | 1728 | 3 | 1 | 1 | 1 |
| NAD(P)H:quinone oxidoreductase 1 | 1728 | 3 | 1 | 1 | 1 |
| quinone reductase 1 | 1728 | 3 | 1 | 1 | 1 |
| chymotrypsin-like | 1506 | 2 | 1 | 1 | 1 |
| p11 | 6281 | 3 | 1 | 1 | 1 |
| P11-II | 6281 | 3 | 1 | 1 | 1 |
| P10 | 6281 | 3 | 1 | 1 | 1 |
| cyclooxygenase | C0033551 | 3 | 3 | 1 | 0.333333333333333 |
| COX | C0033551 | 3 | 3 | 1 | 0.333333333333333 |
| DYT1 | 1861 | 2 | 1 | 1 | 1 |
| amino-4-phosphonobutyric acid | C0002520 | 3 | 2 | 1 | 0.5 |
| amino-acid | C0002520 | 3 | 2 | 1 | 0.5 |
| transferase | C0040676 | 2 | 5 | 1 | 0.2 |
| transferases | C0040676 | 2 | 5 | 1 | 0.2 |
| gamma-GTP | 92170 | 1 |  | 1 |  |
| pros | C3813209 | 1 | 3 | 1 | 0.333333333333333 |
| mGlu5 | 2915 | 3 | 1 | 1 | 1 |
| tyrosinase | C0012524 | 2 | 1 | 1 | 1 |
| neuron-specific enolase | 2026 | 3 | 1 | 1 | 1 |
| NSE | 2026 | 3 | 1 | 1 | 1 |
| Peroxisome proliferator-activated receptor | C0166418 | 3 | 3 | 1 | 0.333333333333333 |
| PPARs | C0166418 | 3 | 3 | 1 | 0.333333333333333 |
| Peroxisome proliferator-activated receptors | C0166418 | 3 | 3 | 1 | 0.333333333333333 |
| decarboxylase | C0007054 | 2 |  | 1 |  |
| NeuN | 287847 | 1 | 2 | 1 | 0.5 |
| Pituitary adenylate cyclase-activating polypeptide | C0071163 | 3 |  | 1 |  |
| Pituitary adenylate cyclase activating polypeptide | C0071163 | 3 |  | 1 |  |
| PACAP | C0071163 | 3 |  | 1 |  |
| glutamic acid decarboxylase | C0017785 | 3 | 2 | 1 | 0.5 |
| natriuretic peptide | C1144709 | 1 |  | 1 |  |
| MSN | 17698 | 3 | 1 | 1 | 1 |
| JNK | 116554 | 3 | 3 | 1 | 0.333333333333333 |
| Bad | 64639 | 1 |  | 1 |  |
| MAP2 | 4133 | 3 | 1 | 1 | 1 |
| MAP-2 | 4133 | 3 | 1 | 1 | 1 |
| amino acid transporters | C0949771 | 1 | 1 | 1 | 1 |
| EAATs | C0949771 | 1 | 1 | 1 | 1 |
| glycogen synthase kinase | C0206354 | 1 |  | 1 |  |
| Cre-recombinase | C1744318 | 1 | 4 | 1 | 0.25 |
| ESC | C1851476 |  | 4 | 1 | 0.25 |
| NG2)- | 1464 | 1 | 2 | 1 | 0.5 |
| bovine serum albumin | C0036774 | 1 | 2 | 1 | 0.5 |
| pyruvate dehydrogenase | C0034343 | 2 |  | 1 |  |
| alpha(1) | C1979844 | 1 | 4 | 1 | 0.25 |
| tCR | 6962 | 2 |  | 1 |  |
| NADH dehydrogenase | C0027293 | 2 | 4 | 1 | 0.25 |
| autoreceptors | C0206128 | 2 | 3 | 1 | 0.333333333333333 |
| Txnip | 10628 | 1 |  | 1 |  |
| swapping | 50618 | 2 | 2 | 1 | 0.5 |
| swap | 50618 | 2 | 2 | 1 | 0.5 |
| Small GTPases | C0751984 | 2 | 3 | 1 | 0.333333333333333 |
| small G-proteins | C0751984 | 2 | 3 | 1 | 0.333333333333333 |
| STS | 412 | 1 |  | 1 |  |
| poly-ubiquitin | C0084133 | 2 | 1 | 1 | 1 |
| NAP | 56259 | 1 |  | 1 |  |
| peroxidases | C0031179 | 2 |  | 1 |  |
| a D(2 | 27239 | 2 |  | 1 |  |
| CD1 | 111334 | 1 | 2 | 1 | 0.5 |
| beta-galactosidase | C0005220 | 1 |  | 1 |  |
| IGF2 | 3481 | 1 |  | 1 |  |
| gene cluster | C0017258 | 1 |  | 1 |  |
| transferrin receptor | 64678 | 1 |  | 1 |  |
| TfR | 64678 | 1 |  | 1 |  |
| Yap1 | 855005 | 1 |  | 1 |  |
| Yap1p | 855005 | 1 |  | 1 |  |
| MDC | 4185 | 2 |  | 1 |  |
| coil | 8161 | 2 | 3 | 1 | 0.333333333333333 |
| coilin | 8161 | 2 | 3 | 1 | 0.333333333333333 |
| CD11b | 25021 | 2 | 2 | 1 | 0.5 |
| CaMKII | 818 | 1 | 3 | 1 | 0.333333333333333 |
| NAC | 195046 | 1 | 1 | 1 | 1 |
| Cav | 763 | 1 | 1 | 1 | 1 |
| G(i/ | C0086706 | 1 | 1 | 1 | 1 |
| Cav1 | 857 | 1 |  | 1 |  |
| Angiotensin II type 1 receptors | C0529330 | 1 | 3 | 1 | 0.333333333333333 |
| PACAP | 116 | 2 | 1 | 1 | 1 |
| microtubule-associated protein tau | 17762 |  | 3 | 1 | 0.333333333333333 |
| cell- | 1056 | 2 | 1 | 1 | 1 |
| Rab1 | 5861 | 2 |  | 1 |  |
| Angiotensin II | 24179 | 2 | 2 | 1 | 0.5 |
| trap | 100187907 | 2 | 2 | 1 | 0.5 |
| c-Jun | 64663 | 2 | 3 | 1 | 0.333333333333333 |
| protein kinase C | 64663 | 2 | 3 | 1 | 0.333333333333333 |
| PKC | 64663 | 2 | 3 | 1 | 0.333333333333333 |
| noggin | 9241 | 2 |  | 1 |  |
| p47phox | 653361 | 2 | 2 | 1 | 0.5 |
| p47(phox) | 653361 | 2 | 2 | 1 | 0.5 |
| thrombin | 29251 | 1 |  | 1 |  |
| GWA | C0017428 | 1 | 3 | 1 | 0.333333333333333 |
| movers | 127262 |  |  | 1 |  |
| amyloid fibers | C0475316 | 2 | 2 | 1 | 0.5 |
| microtubule-associated protein Light Chain 3 | C1446946 | 1 | 3 | 1 | 0.333333333333333 |
| red fluorescent protein | C0960938 | 1 |  | 1 |  |
| beta-methylamino alanine | C0124981 | 2 | 1 | 1 | 1 |
| beta-N-methylamino-L-alanine | C0124981 | 2 | 1 | 1 | 1 |
| caspase-8 | 841 |  | 2 | 1 | 0.5 |
| Lmx1a | 110648 | 1 | 2 | 1 | 0.5 |
| high-density lipoprotein | C0023821 | 2 |  | 1 |  |
| HDL | C0023821 | 2 |  | 1 |  |
| sepiapterin reductase | 20751 | 1 |  | 1 |  |
| SPR | 20751 | 1 |  | 1 |  |
| SPR gene | C1335186 | 1 |  | 1 |  |
| plant origin | C0162728 | 1 | 2 | 1 | 0.5 |
| secreted exosomal proteins | C0597427 | 1 | 2 | 1 | 0.5 |
| cystatin | C0010646 | 1 |  | 1 |  |
| hERG | 2078 | 1 | 2 | 1 | 0.5 |
| dopamine beta hydroxylase | 1621 | 1 | 2 | 1 | 0.5 |
| Dopamine beta-hydroxylase | 1621 | 1 | 2 | 1 | 0.5 |
| DBH -1021C | 1621 | 1 | 2 | 1 | 0.5 |
| annexin V | C0059249 | 1 | 1 | 1 | 1 |
| firefly luciferase | C0311727 | 1 | 2 | 1 | 0.5 |
| deubiquitinating enzyme | C1333282 | 1 | 2 | 1 | 0.5 |
| serum protein | C0036825 | 1 | 2 | 1 | 0.5 |
| apamin | C0003521 | 1 | 2 | 1 | 0.5 |
| Mortalin | C0214599 | 1 |  | 1 |  |
| disease genes pink1 | 8825 | 1 | 2 | 1 | 0.5 |
| 3-NP | C1706141 | 2 |  | 1 |  |
| AChE | 11423 | 1 | 2 | 1 | 0.5 |
| OPA1 | 36578 | 3 |  | 1 |  |
| Optic atrophy 1 | 36578 | 3 |  | 1 |  |
| R1441 | 175683 | 1 |  | 1 |  |
| GABA(A) | 14405 | 1 |  | 1 |  |
| mature neuronal genes | C0017340 | 1 | 2 | 1 | 0.5 |
| regulatory ATPase gene | C0017362 |  | 2 | 1 | 0.5 |
| glycogen synthase kinase-3 | C0244989 | 1 | 2 | 1 | 0.5 |
| glycogen synthase kinase (GSK)-3 | C0244989 | 1 | 2 | 1 | 0.5 |
| GSK-3 | C0244989 | 1 | 2 | 1 | 0.5 |
| anti-apoptotic proteins | C1565114 | 1 | 3 | 1 | 0.333333333333333 |
| Superoxide dismutase | 39251 | 1 | 1 | 1 | 1 |
| MAP2 | 17756 | 1 |  | 1 |  |
| Neurofilament M | C2981622 | 1 |  | 1 |  |
| miRNA-433 | 574034 | 1 | 2 | 1 | 0.5 |
| microRNA-433 | 574034 | 1 | 2 | 1 | 0.5 |
| Mixed lineage kinase | 9175 | 1 | 2 | 1 | 0.5 |
| FMR1 | 2332 | 1 | 2 | 1 | 0.5 |
| FMR1 gene | 2332 | 1 | 2 | 1 | 0.5 |
| CagA | 6279 |  |  | 1 |  |
| dopamine beta-hydroxylase | C0013032 | 1 | 2 | 1 | 0.5 |
| cyclin D1 | 58919 | 2 |  | 1 |  |
| cyclin-D1 | 58919 | 2 |  | 1 |  |
| cytokine genes | C1333196 | 1 | 2 | 1 | 0.5 |
| conotoxin | C0304176 | 1 | 1 | 1 | 1 |
| conotoxins | C0304176 | 1 | 1 | 1 | 1 |
| interleukin-1 receptor antagonist | C1704264 | 1 | 1 | 1 | 1 |
| phosphoserine | C0031721 | 1 |  | 1 |  |
| adapter proteins | C0599697 | 1 | 2 | 1 | 0.5 |
| adapter protein | C0599697 | 1 | 2 | 1 | 0.5 |
| P-gp | 283871 | 3 | 1 | 1 | 1 |
| aldose reductase | C0002003 |  |  | 1 |  |
| IKAP | 8518 |  |  | 1 |  |
| SERT | 15567 | 1 | 1 | 1 | 1 |
| GLT1 | 29482 | 1 | 2 | 1 | 0.5 |
| EAAT2 | 29482 | 1 | 2 | 1 | 0.5 |
| leucine-rich repeat kinase 1 | 79705 | 3 | 1 | 1 | 1 |
| LRRK1 | 79705 | 3 | 1 | 1 | 1 |
| CAA | 10194 | 3 |  | 1 |  |
| galectin-1 | 3956 | 1 |  | 1 |  |
| sorcin | 6717 | 1 |  | 1 |  |
| septin 4 | 5414 | 1 | 2 | 1 | 0.5 |
| 4-Sep | 5414 | 1 | 2 | 1 | 0.5 |
| V-type ATPase | C0379099 | 1 | 2 | 1 | 0.5 |
| gene loci | C1708726 | 1 | 2 | 1 | 0.5 |
| yeast protein | C0872306 |  | 2 | 1 | 0.5 |
| vGluT1 | 57030 | 1 |  | 1 |  |
| monoamine oxidase A | C0026455 | 1 |  | 1 |  |
| monoamine oxidase -A | C0026455 | 1 |  | 1 |  |
| ANAs | C0003241 | 1 | 1 | 1 | 1 |
| tau-proteins | C0085401 |  | 2 | 1 | 0.5 |
| tau-protein | C0085401 |  | 2 | 1 | 0.5 |
| Engrailed-1 | 685360 | 1 | 1 | 1 | 1 |
| DSI | 5034 | 2 | 1 | 1 | 1 |
| Sox2 | 20674 | 1 |  | 1 |  |
| neurotrophin receptors | C0132173 | 1 |  | 1 |  |
| NT-4/5 | 4909 |  |  | 1 |  |
| cytoplasmic protein | C1333198 | 2 | 3 | 1 | 0.333333333333333 |
| histone H4 | C0019648 | 1 |  | 1 |  |
| phi | C0027851 | 1 |  | 1 |  |
| arms | 57498 | 2 |  | 1 |  |
| Tak | 1025 | 1 |  | 1 |  |
| neuropeptide Y | C0027893 |  | 1 | 1 | 1 |
| homologous protein | C1512488 |  | 2 | 1 | 0.5 |
| Asp | 434 | 1 | 4 | 1 | 0.25 |
| MrOS | 8011 | 1 |  | 1 |  |
| interferon-gamma | 25712 | 1 |  | 1 |  |
| xanthine oxidase | C0043317 |  |  | 1 |  |
| caspase-12 | C0911469 |  | 1 | 1 | 1 |
| ferroxidase | C0007841 | 3 | 1 | 1 | 1 |
| Ceruloplasmin | C0007841 | 3 | 1 | 1 | 1 |
| Cp | C0007841 | 3 | 1 | 1 | 1 |
| PUFA | 9933 | 1 | 1 | 1 | 1 |
| YWHAZ | 7534 | 1 |  | 1 |  |
| protein tau ( | 10971 | 1 | 1 | 1 | 1 |
| spot | C1427618 | 1 | 1 | 1 | 1 |
| phosphorylation-state-specific TH antibodies | C1257988 | 2 |  | 1 |  |
| phospho-specific antibodies | C1257988 | 2 |  | 1 |  |
| Ki67 | 4288 | 1 | 1 | 1 | 1 |
| Synaptobrevin | C0078208 |  |  | 1 |  |
| alpha-methyl tyrosine | C0051427 | 1 |  | 1 |  |
| alpha-MT | C0051427 | 1 |  | 1 |  |
| tyrosine hydroxylase inhibitor | C1519723 | 1 |  | 1 |  |
| tuberin | 7249 | 1 | 1 | 1 | 1 |
| PTEN | 19211 | 1 | 1 | 1 | 1 |
| choline acetyltransferase | 290567 | 1 |  | 1 |  |
| PARK11 | 26058 | 1 | 1 | 1 | 1 |
| GIGYF2 | 26058 | 1 | 1 | 1 | 1 |
| TNRC15 | 26058 | 1 | 1 | 1 | 1 |
| Grb10-Interacting GYF Protein-2 | 26058 | 1 | 1 | 1 | 1 |
| GIGYF2 gene | 26058 | 1 | 1 | 1 | 1 |
| PARK11 gene | 26058 | 1 | 1 | 1 | 1 |
| global protein | C0178663 | 1 | 1 | 1 | 1 |
| globular proteins | C0178663 | 1 | 1 | 1 | 1 |
| A2A | 28882 | 1 | 1 | 1 | 1 |
| Ndufs4 gene | 4724 | 1 | 1 | 1 | 1 |
| SCF | 4254 | 1 |  | 1 |  |
| phosphodiesterase IV | C0070829 | 1 |  | 1 |  |
| FGF2 | 54250 | 1 | 1 | 1 | 1 |
| Huntington disease gene | C0872189 | 1 | 1 | 1 | 1 |
| HD | C0872189 | 1 | 1 | 1 | 1 |
| HEK | 2042 | 1 |  | 1 |  |
| histamine H3 receptor | C0062739 | 1 |  | 1 |  |
| hsf-1 | 3297 | 3 |  | 1 |  |
| HSF1 | 3297 | 3 |  | 1 |  |
| heat shock factor 1 | 3297 | 3 |  | 1 |  |
| Heat shock transcription factor 1 | 3297 | 3 |  | 1 |  |
| synuclein family | C1336547 | 2 | 2 | 1 | 0.5 |
| guanosine triphosphatases | C3825040 | 1 |  | 1 |  |
| beta-actin | 728378 | 1 |  | 1 |  |
| dopamine D1/D5 receptor | C0114836 | 1 |  | 1 |  |
| leucinal | C0064781 | 2 |  | 1 |  |
| CPAP | 55835 |  |  | 1 |  |
| amino acid receptors | C0051610 |  | 1 | 1 | 1 |
| Calbindin 1 | 793 | 1 | 1 | 1 | 1 |
| CALB1 | 793 | 1 | 1 | 1 | 1 |
| cf ' | C3811819 |  |  | 1 |  |
| CMAP | 8530 | 1 |  | 1 |  |
| PDE | 501 | 1 | 1 | 1 | 1 |
| SWAN | 10137 | 1 |  | 1 |  |
| Ott | 64783 | 1 | 1 | 1 | 1 |
| neurotensin | C0027930 |  | 1 | 1 | 1 |
| insulin receptor substrate 2 | 29376 | 1 |  | 1 |  |
| IRS2 | 29376 | 1 |  | 1 |  |
| penumbra | 340348 |  |  | 1 |  |
| tumor necrosis factor alpha (TNF) gene | C1710304 | 2 |  | 1 |  |
| TNF gene | C1710304 | 2 |  | 1 |  |
| glucose transporter 4 | 6517 | 1 |  | 1 |  |
| ENT | 4907 | 1 |  | 1 |  |
| SIRT3 | 64384 |  | 1 | 1 | 1 |
| heat shock protein 90 | C0243044 | 4 | 1 | 1 | 1 |
| heat-shock protein 90 | C0243044 | 4 | 1 | 1 | 1 |
| B27 | 56246 | 1 | 1 | 1 | 1 |
| leukemia inhibitory factor | 3976 | 1 | 1 | 1 | 1 |
| LIF | 3976 | 1 | 1 | 1 | 1 |
| spinophilin | C0754682 | 1 |  | 1 |  |
| delta-aminolevulinate dehydratase | C0002564 | 1 |  | 1 |  |
| delta-ALA | C0002563 | 1 |  | 1 |  |
| protein phosphatase (PP) 1 | C1956003 | 1 |  | 1 |  |
| albumin | 213 | 1 | 1 | 1 | 1 |
| MUT | 4594 | 1 | 1 | 1 | 1 |
| IL-1 | C1522428 | 1 |  | 1 |  |
| MPO(+ | 4353 | 3 |  | 1 |  |
| myeloperoxidase | 4353 | 3 |  | 1 |  |
| MPO | 4353 | 3 |  | 1 |  |
| iMPO | 4353 | 3 |  | 1 |  |
| SUMO E3 ligase protein | C0534100 | 1 |  | 1 |  |
| C18 | 27241 | 2 |  | 1 |  |
| Grp58 | 29468 | 1 |  | 1 |  |
| Msx1 | 4487 | 2 | 1 | 1 | 1 |
| msh homeobox homolog 1 | 4487 | 2 | 1 | 1 | 1 |
| transcription factor genes | C0812327 | 3 |  | 1 |  |
| transcription factor gene | C0812327 | 3 |  | 1 |  |
| etanercept | C0717758 | 1 | 1 | 1 | 1 |
| protein (a | C0038164 | 1 |  | 1 |  |
| protein botulinum neurotoxin A | C0038164 | 1 |  | 1 |  |
| terminal deoxynucleotidyl transferase | 21673 | 1 |  | 1 |  |
| neutral protease | C0025250 | 1 |  | 1 |  |
| VIII | 1351 | 1 | 1 | 1 | 1 |
| Pompe disease | 2548 | 1 | 1 | 1 | 1 |
| mSv | 4342 | 1 |  | 1 |  |
| PARK2 | 56816 |  |  | 1 |  |
| pro-inflammatory cytokine tumor necrosis factor-alpha | C0378389 | 1 |  | 1 |  |
| LTB | 4050 | 1 |  | 1 |  |
| A20 | 7128 | 1 | 1 | 1 | 1 |
| MMP-3 | 17392 | 1 | 2 | 1 | 0.5 |
| HSP70 | 266759 | 1 | 2 | 1 | 0.5 |
| PSD-95 | 1742 | 1 | 1 | 1 | 1 |
| ADC | 113451 | 1 |  | 1 |  |
| formerly | C1415386 | 2 | 2 | 1 | 0.5 |
| Drs | 5411 | 1 |  | 1 |  |
| calretinin | C0054544 | 2 | 2 | 1 | 0.5 |
| CD68 | 968 | 1 |  | 1 |  |
| SCA-6 | 773 | 1 |  | 1 |  |
| kin | 22944 | 1 | 1 | 1 | 1 |
| transglutaminase | C0033679 |  | 1 | 1 | 1 |
| TG | C0033679 |  | 1 | 1 | 1 |
| Transglutaminases | C0033679 |  | 1 | 1 | 1 |
| PDK-2 | 5164 | 1 |  | 1 |  |
| secretases | C0171484 |  | 1 | 1 | 1 |
| endosulfine-alpha | 2029 | 1 |  | 1 |  |
| ENSA | 2029 | 1 |  | 1 |  |
| Hes-5 | 15208 | 1 | 1 | 1 | 1 |
| CK-BB | 1152 | 1 |  | 1 |  |
| vesicular glutamate transporter 1 | C0969320 | 1 |  | 1 |  |
| parvalbumin | 5816 | 1 | 3 | 1 | 0.333333333333333 |
| CCR2 | 12772 | 1 |  | 1 |  |
| amyloid beta protein fragment 25-35 | C0168307 | 1 | 1 | 1 | 1 |
| apolipoprotein (Apo)A-I | C0085201 | 1 | 1 | 1 | 1 |
| IRS | 3376 | 1 |  | 1 |  |
| PAR-3 | 8856 |  |  | 1 |  |
| PAR-1 | 2149 |  |  | 1 |  |
| NR2A | 2903 | 1 | 1 | 1 | 1 |
| PTMs | 5763 | 1 |  | 1 |  |
| acute-phase proteins | C0001347 | 1 |  | 1 |  |
| recombinant Tat-fusion protein | C1571593 | 1 |  | 1 |  |
| chemokine-treated VM neurospheres coexpressed Nurr1 | C0524914 | 1 | 1 | 1 | 1 |
| 5-HT(1B | 3351 |  | 1 | 1 | 1 |
| E-16 | 8140 | 1 |  | 1 |  |
| norepinephrine transporter | 6530 | 1 |  | 1 |  |
| BMP Receptor | C0289417 | 1 |  | 1 |  |
| MIS | 8449 | 1 |  | 1 |  |
| fibronectin | C0016055 | 1 |  | 1 |  |
| Methionine sulfoxide reductase A | 4482 | 1 |  | 1 |  |
| MsrA | 4482 | 1 |  | 1 |  |
| methionine sulfoxide | C0066124 | 1 |  | 1 |  |
| cytokine receptors | C0206552 |  |  | 1 |  |
| second most important disease causing gene | C1708995 | 1 |  | 1 |  |
| t = 3. | C0041014 | 1 | 2 | 1 | 0.5 |
| AII | C0003018 | 1 |  | 1 |  |
| DR5 | 8795 | 1 |  | 1 |  |
| Hdj2 | 3301 | 1 |  | 1 |  |
| DM2 | 7555 |  |  | 1 |  |
| Syt 4 | 64440 | 1 |  | 1 |  |
| neuroserpin | 5274 | 1 |  | 1 |  |
| galactosidase | C0016955 | 1 |  | 1 |  |
| AT1 | 9197 | 2 | 1 | 1 | 1 |
| AT(1 | 9197 | 2 | 1 | 1 | 1 |
| AII type-1 | C0003006 | 1 |  | 1 |  |
| POLG1 | 5428 |  | 3 | 1 | 0.333333333333333 |
| motor protein | C1179106 | 3 | 1 | 1 | 1 |
| Otx2 | 5015 | 2 |  | 1 |  |
| p65 | 65125 | 2 | 2 | 1 | 0.5 |
| CX3CR1 | 13051 | 1 |  | 1 |  |
| KIAA1267 | 284058 | 1 |  | 1 |  |
| acid alpha-glucosidase | C0015278 | 1 |  | 1 |  |
| MAO | 29253 | 1 | 1 | 1 | 1 |
| thrombin receptors | C0076552 |  |  | 1 |  |
| thrombin | 2147 |  |  | 1 |  |
| Atm | 472 | 1 |  | 1 |  |
| ataxia-telangiectasia mutated | 472 | 1 |  | 1 |  |
| 5-HT2 receptors | C0036757 | 1 |  | 1 |  |
| BAG5 | 9529 | 1 |  | 1 |  |
| sncb | 6620 | 1 |  | 1 |  |
| ND5 | 4540 |  |  | 1 |  |
| TrkA | 4914 |  | 1 | 1 | 1 |
| p52 | 2968 | 1 |  | 1 |  |
| aid | C3540469 | 3 |  | 1 |  |
| aided | C3540469 | 3 |  | 1 |  |
| CAS | 1434 | 1 |  | 1 |  |
| horseradish peroxidase | C0019941 | 1 |  | 1 |  |
| DMT1 | 4891 | 1 |  | 1 |  |
| Nramp2 | 4891 | 1 |  | 1 |  |
| Slc11a2 | 4891 | 1 |  | 1 |  |
| FECH | 2235 | 1 |  | 1 |  |
| ALAS2 | 212 | 1 |  | 1 |  |
| CYP3A | 1576 | 1 | 1 | 1 | 1 |
| WFS1 | 7466 |  |  | 1 |  |
| wolframin | 7466 |  |  | 1 |  |
| myotonic dystrophy | 1760 |  |  | 1 |  |
| CTX | 1593 | 1 |  | 1 |  |
| Q10 | 27161 | 1 | 2 | 1 | 0.5 |
| PD2- | 54623 | 1 |  | 1 |  |
| PD2 | 54623 | 1 |  | 1 |  |
| interleukin (IL)-2 | C0021756 | 1 | 1 | 1 | 1 |
| PSD | 23761 |  |  | 1 |  |
| zif-268 | 24330 | 1 |  | 1 |  |
| Zif268 | 24330 | 1 |  | 1 |  |
| alpha4 nAChR | 11438 | 1 |  | 1 |  |
| secretory phospholipase A2 | C2756983 | 1 |  | 1 |  |
| cyclic nucleotide phosphodiesterases | C0056696 | 1 |  | 1 |  |
| MEK2-CA | 5605 | 1 | 1 | 1 | 1 |
| beta-adrenergic receptors | C0034785 | 1 |  | 1 |  |
| beta-ARs | C0034785 | 1 |  | 1 |  |
| MEPs | C1707163 | 1 |  | 1 |  |
| interleukin 2 | 116562 | 1 |  | 1 |  |
| IL-2 | 116562 | 1 |  | 1 |  |
| stimulatory G-proteins | C0086903 | 1 |  | 1 |  |
| ZO-1 | 21872 | 1 |  | 1 |  |
| PKA inhibitors | C0243459 | 1 |  | 1 |  |
| DARPP-32 | 19049 | 2 | 2 | 1 | 0.5 |
| enzyme genetic | C1333402 | 1 |  | 1 |  |
| Kir2.3 | 3761 | 1 |  | 1 |  |
| Kir2.3's | 3761 | 1 |  | 1 |  |
| calcitonin gene-related peptide | C0006669 |  |  | 1 |  |
| low-affinity p75 neurotrophin receptor | 4804 | 2 |  | 1 |  |
| p75NTR | 4804 | 2 |  | 1 |  |
| BMPRII | 659 | 1 |  | 1 |  |
| BMPRIIDN | 659 | 1 |  | 1 |  |
| AAV-GAD | 17 |  | 1 | 1 | 1 |
| AAV-AADC | 17 |  | 1 | 1 | 1 |
| mGluR4 | 14802 | 1 | 2 | 1 | 0.5 |
| EP2 | 5732 |  |  | 1 |  |
| Trak1 | 22906 | 1 |  | 1 |  |
| Trak1 | 67095 | 1 |  | 1 |  |
| hole- | 80757 | 1 | 1 | 1 | 1 |
| Met1 | 3004 | 1 |  | 1 |  |
| mbt | 5657 | 1 |  | 1 |  |
| Nat | 6046 | 1 |  | 1 |  |
| Rao | 314 | 2 |  | 1 |  |
| tensin | 7145 | 1 | 3 | 1 | 0.333333333333333 |
| synapsin-1 | 24949 | 1 | 1 | 1 | 1 |
| DYRK1A | 1859 | 1 | 2 | 1 | 0.5 |
| Jun NH2-terminal kinase | C0248813 | 1 |  | 1 |  |
| somatostatin | 6750 |  | 1 | 1 | 1 |
| CYP1A2 | 1544 | 1 | 1 | 1 | 1 |
| ceruloplasmin | 12870 | 1 |  | 1 |  |
| PSEN2 | 5664 | 1 | 3 | 1 | 0.333333333333333 |
| MAC1 | 16409 | 1 | 1 | 1 | 1 |
| Bcl-x | 24888 | 1 | 3 | 1 | 0.333333333333333 |
| RNA binding protein | C0085177 | 1 |  | 1 |  |
| immunoglobulin | C0021027 | 1 | 2 | 1 | 0.5 |
| cathepsin D | 13033 | 1 | 1 | 1 | 1 |
| motor proteins | C1179106 | 3 | 1 | 1 | 1 |
| AAAD | C0002520 | 3 | 2 | 1 | 0.5 |
| amyloid precursor protein (APP)-processing secretases | C0171484 |  | 1 | 1 | 1 |
| htrA2 | 41756 | 2 | 1 | 1 | 1 |
| MELAS | C1417109 |  | 2 | 1 | 0.5 |
| torsinA | 1861 | 2 | 1 | 1 | 1 |
| PAR | 8856 |  |  | 1 |  |
| delta opioid receptors | C0140057 | 1 | 2 | 1 | 0.5 |
| delta receptors | C0140057 | 1 | 2 | 1 | 0.5 |
| delta opioid peptide (DOP) receptor | C0140057 | 1 | 2 | 1 | 0.5 |
| DOP receptor | C0140057 | 1 | 2 | 1 | 0.5 |
| DOP receptors | C0140057 | 1 | 2 | 1 | 0.5 |
| Msx-1 | 4487 | 2 | 1 | 1 | 1 |
| RTP801 | C3539835 | 1 | 1 | 1 | 1 |
| DAF-FM | 1604 | 1 | 1 | 1 | 1 |
| NGF | 4803 |  | 2 | 1 | 0.5 |
| heat shock protein-90 | C0243044 | 4 | 1 | 1 | 1 |
| PNN | 5411 | 1 |  | 1 |  |
| CD34 | 947 | 1 |  | 1 |  |
| amyloid fibres | C0475316 | 2 | 2 | 1 | 0.5 |
| HIRMAb | 3761 | 1 |  | 1 |  |
| IDUA | 3425 |  |  | 1 |  |
| nucleases | C0597094 | 1 |  | 1 |  |
| AAV-DDC | 17 |  | 1 | 1 | 1 |
| H. pylori antibody | C0369303 |  |  | 1 |  |
| H. pylori antibodies | C0369303 |  |  | 1 |  |
| cell's | 1056 | 2 | 1 | 1 | 1 |
| KMO | 8564 | 1 |  | 1 |  |
| CFI | 3426 | 1 |  | 1 |  |
| protein tau | 10971 | 1 | 1 | 1 | 1 |
| PDI | 5034 | 2 | 1 | 1 | 1 |
| eotaxin | C0250604 | 1 |  | 1 |  |
| PPARgamma | 25664 |  | 3 | 1 | 0.333333333333333 |
| mitogenic | C0018284 | 4 | 3 | 1 | 0.333333333333333 |
| microtubule-associated protein light chain-3 | C1446946 | 1 | 3 | 1 | 0.333333333333333 |
| extracellular matrix proteins | C0079323 |  |  | 1 |  |
| DBP | 1628 |  | 1 | 1 | 1 |
| PTP | 3690 |  | 2 | 1 | 0.5 |
| adrenergic receptors | C0034783 |  | 1 | 1 | 1 |
| adrenoceptors | C0034783 |  | 1 | 1 | 1 |
| ubiquitin C-terminal hydrolase | C0164005 | 3 | 4 | 1 | 0.25 |
| UCHL1 | 22223 | 3 | 5 | 1 | 0.2 |
| F-box protein | C1257945 |  | 1 | 1 | 1 |
| F-box proteins | C1257945 |  | 1 | 1 | 1 |
| immunoglobulins | C0021027 | 1 | 2 | 1 | 0.5 |
| SNpr | 4204 |  | 3 | 1 | 0.333333333333333 |
| mGlu(7) receptors | 4204 |  | 3 | 1 | 0.333333333333333 |
| liothyronine | C0041014 | 1 | 2 | 1 | 0.5 |
| RXR | C0140283 |  | 1 | 1 | 1 |
| Bcl-xl | 24888 | 1 | 3 | 1 | 0.333333333333333 |
| c-Myc | 24577 |  | 1 | 1 | 1 |
| protein kinase G | C0010536 |  | 1 | 1 | 1 |
| small G proteins | C0751984 | 2 | 3 | 1 | 0.333333333333333 |
| #NAME? | 51761 | 3 | 4 | 1 | 0.25 |
| mitoK(ATP) | 51761 | 3 | 4 | 1 | 0.25 |
| p44 | 50689 |  | 1 | 1 | 1 |
| miRNAs miR-181c | 406957 |  | 1 | 1 | 1 |
| Ile | C0022192 |  | 4 | 1 | 0.25 |
| histamine-N-methyltransferase | 3176 | 2 | 2 | 1 | 0.5 |
| G20 | 51161 |  | 1 | 1 | 1 |
| THP-1 | 2736 |  | 1 | 1 | 1 |
| stop | 4135 |  | 1 | 1 | 1 |
| neuropeptide Y | 4852 |  | 2 | 1 | 0.5 |
| NPY | 4852 |  | 2 | 1 | 0.5 |
| CTR | 799 |  | 2 | 1 | 0.5 |
| NRF-1 | C1565068 |  | 1 | 1 | 1 |
| growth/differentiation factor 5 | 8200 |  | 3 | 1 | 0.333333333333333 |
| GDF5 | 8200 |  | 3 | 1 | 0.333333333333333 |
| GDF5's | 8200 |  | 3 | 1 | 0.333333333333333 |
| BBS | 583 |  | 2 | 1 | 0.5 |
| CYP1A1 | 1543 | 1 | 1 | 1 | 1 |
| CYP1A2 genes | 1544 | 1 | 1 | 1 | 1 |
| neurofilaments 200 | C0068589 |  | 2 | 1 | 0.5 |
| Spino- | 84687 |  | 1 | 1 | 1 |
| G protein-coupled receptor (GPCR) kinases | C0872043 | 1 | 1 | 1 | 1 |
| ATP13A2 | 74772 |  | 2 | 1 | 0.5 |
| neutralizing antibodies | C0475463 |  | 3 | 1 | 0.333333333333333 |
| APP/PS/tau | 10971 | 1 | 1 | 1 | 1 |
| 5-hydroxytryptophan | C0000578 |  | 2 | 1 | 0.5 |
| interleukin-1alpha | 16175 |  | 1 | 1 | 1 |
| regucalcin | 9104 |  | 1 | 1 | 1 |
| L-tyrosine | C0041485 | 1 | 7 | 1 | 0.142857142857143 |
| calcium-dependent protein kinase | C0054490 |  | 1 | 1 | 1 |
| AAV-S129A | 17 |  | 1 | 1 | 1 |
| AAV-S129D | 17 |  | 1 | 1 | 1 |
| viral proton channel protein | C0042736 |  | 1 | 1 | 1 |
| calcium-dependent proteins | C2064889 |  | 1 | 1 | 1 |
| acetylcholinesterase | 11423 | 1 | 2 | 1 | 0.5 |
| Tcf4 protein | 6925 |  | 2 | 1 | 0.5 |
| PAG | 5052 |  | 2 | 1 | 0.5 |
| Alcohol dehydrogenases | C0001942 |  | 1 | 1 | 1 |
| ADH | 551 |  | 3 | 1 | 0.333333333333333 |
| ADH1C | 126 |  | 2 | 1 | 0.5 |
| ubiquitin-conjugating enzyme | C0084913 |  | 3 | 1 | 0.333333333333333 |
| Ub-conjugating enzyme | C0084913 |  | 3 | 1 | 0.333333333333333 |
| CCl(4) | 6351 |  | 1 | 1 | 1 |
| frataxin | 2395 |  | 2 | 1 | 0.5 |
| mGlu(4) | 2914 |  | 2 | 1 | 0.5 |
| iron-sulfur protein | C0022095 |  | 1 | 1 | 1 |
| p22(phox | 11331 | 1 | 1 | 1 | 1 |
| Rac1 | 5879 |  | 1 | 1 | 1 |
| ATF4 | 79255 |  | 1 | 1 | 1 |
| translation initiation factor | C0030943 |  | 1 | 1 | 1 |
| eIF2alpha | 502531 |  | 1 | 1 | 1 |
| NADPH oxidase 1 | 27035 |  | 2 | 1 | 0.5 |
| Nox1 | 27035 |  | 2 | 1 | 0.5 |
| neprilysin | C0025250 | 1 |  | 1 |  |
| polyubiquitin | C0084133 | 2 | 1 | 1 | 1 |
| HD gene | C0872189 | 1 | 1 | 1 | 1 |
| Optineurin | 10133 |  | 2 | 1 | 0.5 |
| Mash1 | 429 |  | 2 | 1 | 0.5 |
| virus proteins | C0042736 |  | 1 | 1 | 1 |
| serum proteins | C0036825 | 1 | 2 | 1 | 0.5 |
| PKR | 5313 |  |  | 1 |  |
| MnB | 1859 | 1 | 2 | 1 | 0.5 |
| aAT | 5265 |  | 2 | 1 | 0.5 |
| HMT | 3176 | 2 | 2 | 1 | 0.5 |
| fibrinogen | 2244 |  | 2 | 1 | 0.5 |
| Disease Rating Scale (UPDRS) part 1 | 8825 | 1 | 2 | 1 | 0.5 |
| LINGO-2 | 158038 |  | 1 | 1 | 1 |
| receptor-interacting protein-1 | 8737 |  | 1 | 1 | 1 |
| gamma2 | 7453 |  | 2 | 1 | 0.5 |
| ESCs | C1851476 |  | 4 | 1 | 0.25 |
| M1 receptors | C0531859 |  | 1 | 1 | 1 |
| M1 receptor | C0531859 |  | 1 | 1 | 1 |
| RTP801 | 74747 |  | 1 | 1 | 1 |
| KO) | 3856 |  | 2 | 1 | 0.5 |
| CCL5 | 6352 |  | 1 | 1 | 1 |
| B(12 | 4709 | 2 | 3 | 1 | 0.333333333333333 |
| RAP- | 4043 |  | 1 | 1 | 1 |
| OPTN | 10133 |  | 2 | 1 | 0.5 |
| cytochrome | C0010798 |  | 5 | 1 | 0.2 |
| CYP3A4 | 1576 | 1 | 1 | 1 | 1 |
| PDF | 64146 | 1 | 2 | 1 | 0.5 |
| mdNSCs | 7291 |  | 1 | 1 | 1 |
| hMSCs | 7291 |  | 1 | 1 | 1 |
| Abs | 51428 |  |  | 1 |  |
| MDN's | 56953 |  |  | 1 |  |
| protein(s | C0072393 |  | 1 | 1 | 1 |
| bone marrow stromal cell antigen 1 | C0255325 |  |  | 1 |  |
| GAK | 2580 |  | 6 | 1 | 0.166666666666667 |
| cyclin G associated kinase | 2580 |  | 6 | 1 | 0.166666666666667 |
| EIF4G1 | 1981 |  | 4 | 1 | 0.25 |
| complementarity determining regions | C0021024 |  | 2 | 1 | 0.5 |
| secreted CXCL10 protein | C0597427 | 1 | 2 | 1 | 0.5 |
| caspase-3/7 | C0537969 |  | 1 | 1 | 1 |
| KLF4 | 9314 |  | 4 | 1 | 0.25 |
| presenilin-1 | C0299212 |  | 1 | 1 | 1 |
| angiotensin type-1 receptor | C0529330 | 1 | 3 | 1 | 0.333333333333333 |
| PP1 | 5540 |  | 1 | 1 | 1 |
| neutralizing anti-GDNF antibody | C0475463 |  | 3 | 1 | 0.333333333333333 |
| gene promoters | C0314621 |  | 1 | 1 | 1 |
| glutathione reductase | 116686 |  | 3 | 1 | 0.333333333333333 |
| RTs | 4204 |  | 3 | 1 | 0.333333333333333 |
| PP2A | 5524 |  | 2 | 1 | 0.5 |
| CPu | 1361 |  | 4 | 1 | 0.25 |
| HSP70 | 15511 |  | 4 | 1 | 0.25 |
| TK | C0041485 | 1 | 7 | 1 | 0.142857142857143 |
| integrin alphaM | C1136310 |  | 1 | 1 | 1 |
| pyridoxal kinase | C0034265 |  |  | 1 |  |
| apoenzymes | C0003589 |  |  | 1 |  |
| L-type Ca | 5788 |  | 2 | 1 | 0.5 |
| A(2A) | 28882 | 1 | 1 | 1 | 1 |
| hA(2A) | 28882 | 1 | 1 | 1 | 1 |
| matrix metalloproteinase-9 | C0165519 |  | 1 | 1 | 1 |
| One hundred twenty-two | 64783 | 1 | 1 | 1 | 1 |
| FoxP3 | 50943 |  | 2 | 1 | 0.5 |
| vitamin D binding protein | 2638 |  | 1 | 1 | 1 |
| CDD | 4878 |  |  | 1 |  |
| class B member 2 gene | C0017349 |  | 1 | 1 | 1 |
| mannose-6-phosphate-independent receptor | 3482 |  | 1 | 1 | 1 |
| HOGG1 | 4968 |  | 1 | 1 | 1 |
| HSP-70 | 266759 | 1 | 2 | 1 | 0.5 |
| S100 protein | 6271 |  | 1 | 1 | 1 |
| TST | 7263 |  | 1 | 1 | 1 |
| Daf | 1604 | 1 | 1 | 1 | 1 |
| KYP-2047 | C2935972 |  | 1 | 1 | 1 |
| CB1 receptors | C0378126 |  | 1 | 1 | 1 |
| CB1 receptor | C0378126 |  | 1 | 1 | 1 |
| amino buturic acid (GABA) transporter | C0949771 | 1 | 1 | 1 | 1 |
| GTP cyclohydrolase I | 14528 |  | 1 | 1 | 1 |
| GTPCH | 14528 |  | 1 | 1 | 1 |
| GCH1 | 14528 |  | 1 | 1 | 1 |
| complex I equivalent NADH dehydrogenase | C0171406 | 4 | 3 | 1 | 0.333333333333333 |
| HLA-DRA | 3122 |  | 2 | 1 | 0.5 |
| Developmental origins | C0017340 | 1 | 2 | 1 | 0.5 |
| gene expression regulators | C0017362 |  | 2 | 1 | 0.5 |
| PLK3 | 1263 |  | 1 | 1 | 1 |
| CK2 | C0108555 | 1 | 1 | 1 | 1 |
| homologues proteins | C1512488 |  | 2 | 1 | 0.5 |
| Apoptosis signal-regulating kinase 1 | 26408 |  | 1 | 1 | 1 |
| ASK1 | 26408 |  | 1 | 1 | 1 |
| MAPT | 17762 |  | 3 | 1 | 0.333333333333333 |
| matrix metalloproteinase-3 | 17392 | 1 | 2 | 1 | 0.5 |
| vacuolar ATPase | C0379099 | 1 | 2 | 1 | 0.5 |
| proteolipid | C0033693 |  | 1 | 1 | 1 |
| vacuolar proton ATPase | C0379099 | 1 | 2 | 1 | 0.5 |
| synthetic enzymes | C0597548 | 2 | 1 | 1 | 1 |
| Spargel | 40562 |  | 1 | 1 | 1 |
| peroxisome proliferator-activated receptor gamma coactivator 1 | 40562 |  | 1 | 1 | 1 |
| PGC-1 | 40562 |  | 1 | 1 | 1 |
| r= -0.487 | C0220485 |  | 1 | 1 | 1 |
| iron regulatory protein | C0378503 |  | 1 | 1 | 1 |
| IRP2 | 3658 |  | 1 | 1 | 1 |
| ubiquitin-conjugating E2 enzyme | C0084913 |  | 3 | 1 | 0.333333333333333 |
| F12 | 2161 |  | 1 | 1 | 1 |
| XIAP | 331 |  | 2 | 1 | 0.5 |
| Atg16L1 | 55054 |  | 1 | 1 | 1 |
| E22 | 6925 |  | 2 | 1 | 0.5 |
| RGS4 | 19736 |  | 1 | 1 | 1 |
| GLAST | 6507 |  | 1 | 1 | 1 |
| MEK-Bcl-2 | 5605 | 1 | 1 | 1 | 1 |
| STM | 6818 |  | 1 | 1 | 1 |
| protomer | C1136161 |  | 1 | 1 | 1 |
| Chr | 1125 |  | 1 | 1 | 1 |
| FPN1 | 30061 |  | 2 | 1 | 0.5 |
| ferroportin 1 | 30061 |  | 2 | 1 | 0.5 |
| mGlu4 | 2914 |  | 2 | 1 | 0.5 |
| sICAM-1 | C0063695 |  | 1 | 1 | 1 |
| human MAO-A | C3657721 |  | 2 | 1 | 0.5 |
| TM5 | 7170 |  | 2 | 1 | 0.5 |
| V-type proton ATPase | C0379099 | 1 | 2 | 1 | 0.5 |
| hESC | C1851476 |  | 4 | 1 | 0.25 |
| Transthyretin | 7276 | 2 | 2 | 1 | 0.5 |
| ICAM | 25464 |  | 1 | 1 | 1 |
| ERG | 2078 | 1 | 2 | 1 | 0.5 |
| genetic regulators | C0017362 |  | 2 | 1 | 0.5 |
| overlapping genes | C0017359 |  | 1 | 1 | 1 |
| CYP | 9360 |  | 2 | 1 | 0.5 |
| autophagy protein 5 | C3537312 |  | 1 | 1 | 1 |
| MLK | 9175 | 1 | 2 | 1 | 0.5 |
| ubiquitin carboxyl-terminal hydrolase- | C0164005 | 3 | 4 | 1 | 0.25 |
| tensin induced homolog kinase 1 | 7145 | 1 | 3 | 1 | 0.333333333333333 |
| LPS receptor | C0108768 |  | 1 | 1 | 1 |
| SK3 | 54263 |  | 1 | 1 | 1 |
| growth differentiation factor 5 | 8200 |  | 3 | 1 | 0.333333333333333 |
| GDF5 | 252835 |  | 2 | 1 | 0.5 |
| ABCD1 | 11666 |  |  | 1 |  |
| dopamine-beta-hydroxylase | C0013032 | 1 | 2 | 1 | 0.5 |
| tensin homologue-induced kinase 1 | 7145 | 1 | 3 | 1 | 0.333333333333333 |
| presenilin | C0872078 |  | 1 | 1 | 1 |
| PARL | 55486 |  | 2 | 1 | 0.5 |
| ubiquitin carboxy-terminal hydrolase L1 | 22223 | 3 | 5 | 1 | 0.2 |
| angiotensin type 1 receptor | C0529330 | 1 | 3 | 1 | 0.333333333333333 |
| caspase 12 | 12364 |  | 1 | 1 | 1 |
| protein C | 64663 | 2 | 3 | 1 | 0.333333333333333 |
| Asp(2 | 23621 |  | 1 | 1 | 1 |
| anti-IgG | C0051979 |  | 1 | 1 | 1 |
| methionine sulfoxide reductases | C2713548 |  | 1 | 1 | 1 |
| dynorphin B | C0073381 |  | 1 | 1 | 1 |
| Voltage-gated sodium channels | C3494197 |  | 2 | 1 | 0.5 |
| alpha(1 | C1979844 | 1 | 4 | 1 | 0.25 |
| alpha4beta2 nicotinic receptors | C0911771 |  | 3 | 1 | 0.333333333333333 |
| activating transcription factor 6 | 226641 |  | 2 | 1 | 0.5 |
| ATF6 | 226641 |  | 2 | 1 | 0.5 |
| Aldh1a1 | 11668 |  | 1 | 1 | 1 |
| IL-1RA VNTR 2 | C0021756 | 1 | 1 | 1 | 1 |
| monoamine oxidase (MAO)-A | C0026455 | 1 |  | 1 |  |
| MAO-A | C0026455 | 1 |  | 1 |  |
| aminoacid | C0002520 | 3 | 2 | 1 | 0.5 |
| dynamin-related protein 1 | 74006 |  | 1 | 1 | 1 |
| Drp1 | 74006 |  | 1 | 1 | 1 |
| acetylcholine binding site | C0034792 |  | 2 | 1 | 0.5 |
| PPAR-gamma | 25664 |  | 3 | 1 | 0.333333333333333 |
| guanosine monophosphate (cGMP)-dependent kinase | 4842 | 3 | 3 | 1 | 0.333333333333333 |
| GPCRs | C3812695 |  | 1 | 1 | 1 |
| hMiro2 | 89941 |  | 2 | 1 | 0.5 |
| NADH-Q oxidoreductase | C0682753 |  | 2 | 1 | 0.5 |
| p105 | 1012 |  | 1 | 1 | 1 |
| alanine aminotransferase | C0001899 |  | 1 | 1 | 1 |
| aspartate aminotransferase | C0004002 |  | 1 | 1 | 1 |
| secretory proteins | C0597427 | 1 | 2 | 1 | 0.5 |
| protein disulfide isomerase | C0072354 |  | 2 | 1 | 0.5 |
| Ctrl | 1506 | 2 | 1 | 1 | 1 |
| COMT | 12846 |  | 1 | 1 | 1 |
| exchange factors | C0120465 |  | 1 | 1 | 1 |
| Girk2 | 3763 |  | 1 | 1 | 1 |
| myocyte enhancer factor 2 | C3658245 |  | 2 | 1 | 0.5 |
| glycogen storage disease type II | 2548 | 1 | 1 | 1 | 1 |
| methyltransferase | C0025831 |  | 2 | 1 | 0.5 |
| CAV-2 | 763 | 1 | 1 | 1 | 1 |
| FosB | 14282 |  | 1 | 1 | 1 |
| human GDF5 | 8200 |  | 3 | 1 | 0.333333333333333 |
| Growth/differentiation factor 5 | 252835 |  | 2 | 1 | 0.5 |
| SPL | 8879 |  | 2 | 1 | 0.5 |
| LC3 | 25291 |  | 1 | 1 | 1 |
| DGKQ | 1609 |  | 3 | 1 | 0.333333333333333 |
| LAMP3 | 27074 |  | 2 | 1 | 0.5 |
| TRPC1 | 22063 |  | 2 | 1 | 0.5 |
| stromal interaction molecule 1 | 20866 |  | 1 | 1 | 1 |
| STIM1 | 20866 |  | 1 | 1 | 1 |
| heat shock protein 70.1 | 15511 |  | 4 | 1 | 0.25 |
| Hsp70.1 | 15511 |  | 4 | 1 | 0.25 |
| Hspa1b | 15511 |  | 4 | 1 | 0.25 |
| GluA1 | 50592 |  | 2 | 1 | 0.5 |
| GluR1 | 50592 |  | 2 | 1 | 0.5 |
| GluR-A | 50592 |  | 2 | 1 | 0.5 |
| GWAS | C0017428 | 1 | 3 | 1 | 0.333333333333333 |
| Abs | C0003241 | 1 | 1 | 1 | 1 |
| calbindin D28k | 793 | 1 | 1 | 1 | 1 |
| PSEN-2 | 5664 | 1 | 3 | 1 | 0.333333333333333 |
| hemochromatosis | 3077 | 3 | 2 | 1 | 0.5 |
| Hes1 | 15205 |  | 1 | 1 | 1 |
| Hes5 | 15208 | 1 | 1 | 1 | 1 |
| vasopressin | 551 |  | 3 | 1 | 0.333333333333333 |
| AVP | 551 |  | 3 | 1 | 0.333333333333333 |
| breast cancer resistance protein | 9429 |  | 1 | 1 | 1 |
| swapped | 50618 | 2 | 2 | 1 | 0.5 |
| MUP | 100129193 |  | 1 | 1 | 1 |
| SCN9A gene | 6335 |  | 1 | 1 | 1 |
| Nav1.7 | 6335 |  | 1 | 1 | 1 |
| SCN9A | 6335 |  | 1 | 1 | 1 |
| TRPM2 | 294329 |  |  | 1 |  |
| TRPV1 | 83810 |  | 1 | 1 | 1 |
| GLT-1 | 29482 | 1 | 2 | 1 | 0.5 |
| GABA(B) receptor | C0051610 |  | 1 | 1 | 1 |
| amino butyric acid | C0002520 | 3 | 2 | 1 | 0.5 |
| PICALM | 8301 |  | 2 | 1 | 0.5 |
| cat-2 | 173411 |  | 1 | 1 | 1 |
| DT-diaphorase | 1728 | 3 | 1 | 1 | 1 |
| chondroitin sulfate proteoglycans | C0033689 |  | 2 | 1 | 0.5 |
| isoleucine | C0022192 |  | 4 | 1 | 0.25 |
| mGluR5s | 2915 | 3 | 1 | 1 | 1 |
| interleukin (IL)-1 receptor antagonist | C1704264 | 1 | 1 | 1 | 1 |
| IL-1 receptor antagonist | C1704264 | 1 | 1 | 1 | 1 |
| endonuclease PstI | 6690 |  | 1 | 1 | 1 |
| thioredoxin 1 | C1956394 |  | 1 | 1 | 1 |
| Trx1 | 22166 |  | 1 | 1 | 1 |
| Rho GTPases | C0174045 |  | 1 | 1 | 1 |
| MIRO2 | 89941 |  | 2 | 1 | 0.5 |
| Ras homolog gene family | C1335597 |  | 1 | 1 | 1 |
| RHOT2 | 89941 |  | 2 | 1 | 0.5 |
| PID | 9219 |  | 2 | 1 | 0.5 |
| EP4 | 19219 |  | 1 | 1 | 1 |
| GIGYF2 genes | 26058 | 1 | 1 | 1 | 1 |
| KLK6 | 5653 | 2 | 1 | 1 | 1 |
| Kallikrein-related peptidase 6 | 5653 | 2 | 1 | 1 | 1 |
| presenilin-associated rhomboid-like | 55486 |  | 2 | 1 | 0.5 |
| PARL-related rhomboid-7 gene | 55486 |  | 2 | 1 | 0.5 |
| Parl genes | 55486 |  | 2 | 1 | 0.5 |
| Hes1 | 3280 |  | 1 | 1 | 1 |
| Nedd4- | 4734 |  | 1 | 1 | 1 |
| NG2 | 1464 | 1 | 2 | 1 | 0.5 |
| CB1 | 25248 |  | 1 | 1 | 1 |
| NADH-cytochrome c reductase | C0027293 | 2 | 4 | 1 | 0.25 |
| Angiogenin | C0051844 |  |  | 1 |  |
| glutathione-S-transferase | 58962 |  | 1 | 1 | 1 |
| TRAF6 | 7189 |  | 3 | 1 | 0.333333333333333 |
| grail | 79589 |  | 1 | 1 | 1 |
| Bcl-X(L) | 24888 | 1 | 3 | 1 | 0.333333333333333 |
| Glutathione S-transferase pi | C0537086 |  | 3 | 1 | 0.333333333333333 |
| GSTP | C0537086 |  | 3 | 1 | 0.333333333333333 |
| Galpha(olf) | 14680 |  | 1 | 1 | 1 |
| Gnal gene | 2774 |  | 1 | 1 | 1 |
| Gnal | 14680 |  | 1 | 1 | 1 |
| PKCdelta | 18753 |  | 1 | 1 | 1 |
| NET-PD | C3853572 | 2 | 2 | 1 | 0.5 |
| glycogen phosphorylase | C0917785 |  | 1 | 1 | 1 |
| angiotensin type 1 receptors | C0529330 | 1 | 3 | 1 | 0.333333333333333 |
| RhoA | 11848 |  | 1 | 1 | 1 |
| Rho-Kinase | 19878 |  | 1 | 1 | 1 |
| ROCK II | 19878 |  | 1 | 1 | 1 |
| mtHsp70 | 3313 |  | 1 | 1 | 1 |
| ABC transporters | 51761 | 3 | 4 | 1 | 0.25 |
| ATP-binding cassette (ABC) transporters | 51761 | 3 | 4 | 1 | 0.25 |
| ABC transporter | 51761 | 3 | 4 | 1 | 0.25 |
| pigment epithelium-derived factor | C0213321 |  | 1 | 1 | 1 |
| alpha4beta2 nAChR | C0911771 |  | 3 | 1 | 0.333333333333333 |
| nAChR alpha4beta2 | C0911771 |  | 3 | 1 | 0.333333333333333 |
| heat shock protein (Hsp)90 | C0243044 | 4 | 1 | 1 | 1 |
| neurofilaments NF 200 | C0068589 |  | 2 | 1 | 0.5 |
| vesicular acetylcholine transporter | C0169582 |  | 1 | 1 | 1 |
| DNAJC6 | 9829 |  |  | 1 |  |
| auxilin | 9829 |  |  | 1 |  |
| alpha-adrenergic receptors | C0034784 |  |  | 1 |  |
| GTP-binding proteins | 92170 | 1 |  | 1 |  |
| SRY | 6736 |  | 1 | 1 | 1 |
| DBH | 1621 | 1 | 2 | 1 | 0.5 |
| RGS | 5308 |  | 3 | 1 | 0.333333333333333 |
| delta-opioid receptors | C0140057 | 1 | 2 | 1 | 0.5 |
| PCL | 2324 |  | 1 | 1 | 1 |
| SCA6 | 773 | 1 |  | 1 |  |
| M1s | 1128 |  | 1 | 1 | 1 |
| microRNA (miRNA) -433 | 574034 | 1 | 2 | 1 | 0.5 |
| UCH | C0164005 | 3 | 4 | 1 | 0.25 |
| deubiquitinating enzymes | C1333282 | 1 | 2 | 1 | 0.5 |
| dopamine D(4) receptor | 1815 |  | 1 | 1 | 1 |
| D(4) receptor | 1815 |  | 1 | 1 | 1 |
| tau/Abeta protein | C0085401 |  | 2 | 1 | 0.5 |
| RGS protein | 5308 |  | 3 | 1 | 0.333333333333333 |
| CB2 receptors | C0208757 |  | 1 | 1 | 1 |
| FER | 2241 |  | 1 | 1 | 1 |
| TAK1 | 6885 |  | 2 | 1 | 0.5 |
| Serum PGRN | C0036825 | 1 | 2 | 1 | 0.5 |
| apolipoprotein AII | 336 |  |  | 1 |  |
| AApoAII | 336 |  |  | 1 |  |
| ALP | 11691 |  | 1 | 1 | 1 |
| genes/loci | C1708726 | 1 | 2 | 1 | 0.5 |
| HPT | 3258 |  | 1 | 1 | 1 |
| AD-8 | 353128 |  | 1 | 1 | 1 |
| MEF2 | C3658245 |  | 2 | 1 | 0.5 |
| CA2 | 12349 |  | 1 | 1 | 1 |
| cystatin C | 25307 |  | 1 | 1 | 1 |
| MyD88 | 4615 |  | 2 | 1 | 0.5 |
| IRAK-1 | 3654 |  | 2 | 1 | 0.5 |
| TRAF-6 | 7189 |  | 3 | 1 | 0.333333333333333 |
| High Fe | 3077 | 3 | 2 | 1 | 0.5 |
| yeast prion protein | C0872306 |  | 2 | 1 | 0.5 |
| pantothenate kinase | C0070043 |  |  | 1 |  |
| PON2 | 5445 |  | 1 | 1 | 1 |
| EIF4G1 p | 1981 |  | 4 | 1 | 0.25 |
| mitogen-activated protein | C1979930 |  |  | 1 |  |
| MAP | C1979930 |  |  | 1 |  |
| CRE recombinase | C1744318 | 1 | 4 | 1 | 0.25 |
| GM1 | 210582 |  | 1 | 1 | 1 |
| aconitase | C0001156 |  | 1 | 1 | 1 |
| TOR1A | 1861 | 2 | 1 | 1 | 1 |
| synapsin I | 24949 | 1 | 1 | 1 | 1 |
| En1 | 685360 | 1 | 1 | 1 | 1 |
| TRIF | 140691 |  | 1 | 1 | 1 |
| annexin-V | C0059249 | 1 | 1 | 1 | 1 |
| UCP | 22227 |  | 1 | 1 | 1 |
| CD11b | 3684 |  | 1 | 1 | 1 |
| CD45 | 5788 |  | 2 | 1 | 0.5 |
| mannose receptor | C0065684 |  | 1 | 1 | 1 |
| SOD1 | 24786 |  | 1 | 1 | 1 |
| chemokine receptor | C0524914 | 1 | 1 | 1 | 1 |
| UBE2L3 | 7332 |  | 1 | 1 | 1 |
| UBCH7 | 7332 |  | 1 | 1 | 1 |
| ubiquitin-conjugating enzymes | C0084913 |  | 3 | 1 | 0.333333333333333 |
| SAH | 6296 |  | 1 | 1 | 1 |
| of nuclear receptor-related 1 protein | C0961411 |  | 1 | 1 | 1 |
| of Nurr1 | C0961411 |  | 1 | 1 | 1 |
| DM 1 | 1760 |  |  | 1 |  |
| MEF2D | 4209 |  | 2 | 1 | 0.5 |
| Lon | 9361 |  | 1 | 1 | 1 |
| cystathionase | C0010642 |  | 1 | 1 | 1 |
| CD44 | 960 |  | 1 | 1 | 1 |
| SOD-1 | 24786 |  | 1 | 1 | 1 |
| T10-8S | C2827388 |  | 2 | 1 | 0.5 |
| HSP-70 | 15511 |  | 4 | 1 | 0.25 |
| PK-KO | 3856 |  | 2 | 1 | 0.5 |
| LAMP-2A | 16784 |  | 1 | 1 | 1 |
| ARTS | 5414 | 1 | 2 | 1 | 0.5 |
| MAPKAPK 2 | 9261 |  | 1 | 1 | 1 |
| MK2 | 9261 |  | 1 | 1 | 1 |
| SIMS | 6493 |  |  | 1 |  |
| tau protein | C0085401 |  | 2 | 1 | 0.5 |
| DCTN1 | 1639 |  | 1 | 1 | 1 |
| EAG | 3756 |  | 1 | 1 | 1 |
| NOD2 | 64127 |  |  | 1 |  |
| FXN | 2395 |  | 2 | 1 | 0.5 |
| Wilson's disease | 540 |  | 1 | 1 | 1 |
| calcium/calmodulin-dependent protein kinase | C0769224 |  | 1 | 1 | 1 |
| Dag | 1605 |  |  | 1 |  |
| fibroblastic growth factor | C0016026 | 4 | 3 | 1 | 0.333333333333333 |
| GAD | 2571 | 1 | 1 | 1 | 1 |
| Human albumin | C0304925 |  | 1 | 1 | 1 |
| gene locus | C1708726 | 1 | 2 | 1 | 0.5 |
| C/EBPbeta | 1051 |  | 1 | 1 | 1 |
| CCAAT enhancer-binding protein (C/EBP)beta | C0209548 |  | 1 | 1 | 1 |
| lyase | C0024188 |  | 1 | 1 | 1 |
| lyases | C0024188 |  | 1 | 1 | 1 |
| histamine H(3) receptor | 11255 |  | 1 | 1 | 1 |
| Liver X receptor beta | 22260 |  | 1 | 1 | 1 |
| LXRbeta | 22260 |  | 1 | 1 | 1 |
| nuclear receptor super gene | C1335077 |  | 1 | 1 | 1 |
| LXR | 22260 |  | 1 | 1 | 1 |
| Protein disulfide isomerases | C0072354 |  | 2 | 1 | 0.5 |
| PDIs | C0072354 |  | 2 | 1 | 0.5 |
| SNCAIP | 9627 | 3 | 3 | 1 | 0.333333333333333 |
| Notch1 | 4851 |  | 1 | 1 | 1 |
| L-type voltage-dependent Ca | 5788 |  | 2 | 1 | 0.5 |
| AADC | C0002520 | 3 | 2 | 1 | 0.5 |
| PKCalpha | 24680 |  | 1 | 1 | 1 |
| peroxisome proliferator-activated receptor-gamma | 25664 |  | 3 | 1 | 0.333333333333333 |
| T10 | C2827388 |  | 2 | 1 | 0.5 |
| pol-II | 100616496 | 1 | 1 | 1 | 1 |
| SAP | 4068 |  | 1 | 1 | 1 |
| MOR1 | 4988 |  | 2 | 1 | 0.5 |
| Mfn2 | 170731 |  | 2 | 1 | 0.5 |
| fibroblast growth factor 2 | 54250 | 1 | 1 | 1 | 1 |
| secretase | C0171484 |  | 1 | 1 | 1 |
| miR-133b | 442890 |  | 1 | 1 | 1 |
| developmental genetic | C0017340 | 1 | 2 | 1 | 0.5 |
| developmental genes | C0017340 | 1 | 2 | 1 | 0.5 |
| Nanobodies | C3494234 |  |  | 1 |  |
| ASP( | 434 | 1 | 4 | 1 | 0.25 |
| ASP(+ | 434 | 1 | 4 | 1 | 0.25 |
| apolipoprotein A-1 | C0085201 | 1 | 1 | 1 | 1 |
| glutathione-S-transferase-Pi | C0537086 |  | 3 | 1 | 0.333333333333333 |
| LRP8 gene | C1335671 |  | 1 | 1 | 1 |
| myocyte enhancer factor 2D | 17261 |  | 1 | 1 | 1 |
| MEF2D | 17261 |  | 1 | 1 | 1 |
| cyclooxygenase type-2 | C0387583 |  |  | 1 |  |
| GCTA | C0017428 | 1 | 3 | 1 | 0.333333333333333 |
| SGK | 20393 |  | 1 | 1 | 1 |
| SNARE | 100170220 |  | 2 | 1 | 0.5 |
| DYT6 | 55145 |  |  | 1 |  |
| THAP1 | 55145 |  |  | 1 |  |
| Wnt co-receptor | 8321 |  | 1 | 1 | 1 |
| Dickkopf-1 | C2987134 |  | 1 | 1 | 1 |
| Dkk1 | 293897 |  | 1 | 1 | 1 |
| THAP1 gene | 55145 |  |  | 1 |  |
| aldehyde reductase | 126 |  | 2 | 1 | 0.5 |
| microtubule binding protein tau | C0085401 |  | 2 | 1 | 0.5 |
| IL-12 | C0123759 |  | 1 | 1 | 1 |
| mitogen- | C0018284 | 4 | 3 | 1 | 0.333333333333333 |
| Rhes | 23551 |  |  | 1 |  |
| mTORC1 | 382056 |  | 1 | 1 | 1 |
| HDM2 | 4193 |  | 1 | 1 | 1 |
| ATG32 | 854660 |  | 1 | 1 | 1 |
| c-Rel | 19696 |  | 1 | 1 | 1 |
| IL-12p40 | 16160 |  | 1 | 1 | 1 |
| IL-23 | 83430 |  | 1 | 1 | 1 |
| protein fragment | C1335533 |  | 1 | 1 | 1 |
| protein kinase B | 2185 |  | 2 | 1 | 0.5 |
| alpha-amyloid 1 | C1979844 | 1 | 4 | 1 | 0.25 |
| growth factor- | C0018284 | 4 | 3 | 1 | 0.333333333333333 |
| interleukin-1alpha | 24493 |  | 1 | 1 | 1 |
| IL-1alpha | 24493 |  | 1 | 1 | 1 |
| TrkA | 18211 |  | 1 | 1 | 1 |
| acetylcholine receptors | C0034792 |  | 2 | 1 | 0.5 |
| Twinkle | 56652 |  | 1 | 1 | 1 |
| mitochondrial UCPs | C0066604 |  | 2 | 1 | 0.5 |
| leucine-rich repeat kinases 1 | 79705 | 3 | 1 | 1 | 1 |
| CHK1 | 1111 |  | 1 | 1 | 1 |
| Raf-1 | 22882 |  | 1 | 1 | 1 |
| dup | 4437 |  | 1 | 1 | 1 |
| POLG | 5428 |  | 3 | 1 | 0.333333333333333 |
| SCA10 genes | 25814 |  |  | 1 |  |
| SCA10 | 25814 |  |  | 1 |  |
| MOR | 4988 |  | 2 | 1 | 0.5 |
| Hsf1 | 852806 |  | 1 | 1 | 1 |
| sphingomyelinases | C0037903 |  | 1 | 1 | 1 |
| SMases | C0037903 |  | 1 | 1 | 1 |
| TSC2 | 7249 | 1 | 1 | 1 | 1 |
| dj-1beta | 43652 |  | 1 | 1 | 1 |
| SKN | 55733 |  | 1 | 1 | 1 |
| oxytocin | C0030095 |  | 1 | 1 | 1 |
| OT | C0030095 |  | 1 | 1 | 1 |
| Human leucine-rich repeat kinase 1 | 79705 | 3 | 1 | 1 | 1 |
| ET | 24716 |  | 1 | 1 | 1 |
| rET | 24716 |  | 1 | 1 | 1 |
| miR-382 | 494331 |  | 1 | 1 | 1 |
| swaps | 50618 | 2 | 2 | 1 | 0.5 |
| Wilson disease | 540 |  | 1 | 1 | 1 |
| Star | 359787 |  | 1 | 1 | 1 |
| alpha4beta2 neuronal nicotinic receptors | C0911771 |  | 3 | 1 | 0.333333333333333 |
| G(i) - | C0086706 | 1 | 1 | 1 | 1 |
| calmodulin-dependent protein kinase II | 972 |  | 1 | 1 | 1 |
| CaMKII | 972 |  | 1 | 1 | 1 |
| dihydrofolate reductase | 1719 |  | 1 | 1 | 1 |
| cyclosomatostatin | 6750 |  | 1 | 1 | 1 |
| HLA-E | 3133 |  | 1 | 1 | 1 |
| MHC | 3133 |  | 1 | 1 | 1 |
| copper oxidase | C0007841 | 3 | 1 | 1 | 1 |
| Nramp | 6556 |  | 1 | 1 | 1 |
| Nramps | 6556 |  | 1 | 1 | 1 |
| ribonuclease | C0035542 |  | 1 | 1 | 1 |
| Keap1 | 50868 |  | 1 | 1 | 1 |
| CD31 | 5175 |  | 1 | 1 | 1 |
| Trk | 4914 |  | 1 | 1 | 1 |
| miR-34 | 407040 |  | 1 | 1 | 1 |
| alpha-Adrenoceptors | C0034784 |  |  | 1 |  |
| t (3 | C0041014 | 1 | 2 | 1 | 0.5 |
| CD200 | 17470 |  | 1 | 1 | 1 |
| CD11b | 16409 | 1 | 1 | 1 | 1 |
| lysozyme | 4069 |  | 1 | 1 | 1 |
| sIL- | 6490 |  | 1 | 1 | 1 |
| 4E-BP1 | 1978 |  | 1 | 1 | 1 |
| TRIF- | 148022 |  | 1 | 1 | 1 |
| NADH dehydrogenases | C0682753 |  | 2 | 1 | 0.5 |
| yeast prion proteins | C0872306 |  | 2 | 1 | 0.5 |
| Ca(V)1.2 | 775 |  | 1 | 1 | 1 |
| NG2- | 1464 | 1 | 2 | 1 | 0.5 |
| ATF6alpha | 226641 |  | 2 | 1 | 0.5 |
| ATF4 | 11911 |  | 1 | 1 | 1 |
| xCT | 26570 |  | 1 | 1 | 1 |
| histamine H3 receptors | C0062739 | 1 |  | 1 |  |
| Cend1 | 57754 |  | 1 | 1 | 1 |
| TTR)- | 7276 | 2 | 2 | 1 | 0.5 |
| TTR gene | 7276 | 2 | 2 | 1 | 0.5 |
| serpin A1 | 5265 |  | 2 | 1 | 0.5 |
| PS2 | 5664 | 1 | 3 | 1 | 0.333333333333333 |
| complementarity-determining regions | C0021024 |  | 2 | 1 | 0.5 |
| DNA-repair proteins | C1366764 |  | 1 | 1 | 1 |
| IL-13Ralpha1 | C0527729 |  | 1 | 1 | 1 |
| IL-13Ralpha1 | 16164 |  | 1 | 1 | 1 |
| Il13ra1 | 16164 |  | 1 | 1 | 1 |
| PARK12 | 677662 |  | 1 | 1 | 1 |
| RGS proteins | 5308 |  | 3 | 1 | 0.333333333333333 |
| R7-RGS | 5308 |  | 3 | 1 | 0.333333333333333 |
| mu-opioid receptor | C0066908 |  | 1 | 1 | 1 |
| RAGE | C3539760 |  | 1 | 1 | 1 |
| PDGF-BB | C0246681 |  | 1 | 1 | 1 |
| colony stimulating factor 1 receptor | 1436 |  | 1 | 1 | 1 |
| CSF1R | 1436 |  | 1 | 1 | 1 |
| CSF1R gene | 1436 |  | 1 | 1 | 1 |
| Snap25 | 20614 |  | 1 | 1 | 1 |
| SNAP-25 | 20614 |  | 1 | 1 | 1 |
| AK-7 | 78801 |  | 1 | 1 | 1 |
| polo-like kinase 2 | 20620 |  | 1 | 1 | 1 |
| Plk2 | 20620 |  | 1 | 1 | 1 |
| Mitochondrial neuronal uncoupling proteins | C0066604 |  | 2 | 1 | 0.5 |
| mitochondrial uncoupling proteins | C0066604 |  | 2 | 1 | 0.5 |
| leptin | C0299583 |  | 1 | 1 | 1 |
| Chrna6 | 8973 |  | 1 | 1 | 1 |
| Trx | 116484 |  | 1 | 1 | 1 |
| monoamine oxidase (A | C0026455 | 1 |  | 1 |  |
| disease duration 10.1 | 8825 | 1 | 2 | 1 | 0.5 |
| Tollip | 54472 |  | 1 | 1 | 1 |
| IRAK1 | 3654 |  | 2 | 1 | 0.5 |
| Toll-interacting protein | 54472 |  | 1 | 1 | 1 |
| TRAF6 | 22034 |  | 1 | 1 | 1 |
| transforming growth factor-beta activated kinase 1 | C0379816 |  | 1 | 1 | 1 |
| fusion genes | C1533585 |  | 1 | 1 | 1 |
| microtubule associated protein-light | C1446946 | 1 | 3 | 1 | 0.333333333333333 |
| L-5-hydroxytryptophan | C0000578 |  | 2 | 1 | 0.5 |
| tensin homolog-induced putative kinase protein 1 | 7145 | 1 | 3 | 1 | 0.333333333333333 |
| glycogen synthase kinase 3 | C0244989 | 1 | 2 | 1 | 0.5 |
| Guanine nucleotide exchange factor kalirin-7 | 8874 |  | 1 | 1 | 1 |
| SP 6 | 80320 |  | 1 | 1 | 1 |
| ERRalpha | 2101 |  | 1 | 1 | 1 |
| SMRT | 9612 |  | 1 | 1 | 1 |
| nuclear receptor NR4A2 | C2720177 |  |  | 1 |  |
| CD133(+) | 8842 |  |  | 1 |  |
| PAGAS | 5052 |  | 2 | 1 | 0.5 |
| Abeta25-35 | C0168307 | 1 | 1 | 1 | 1 |
| ATP-Binding Cassette membrane-associated transporter | 51761 | 3 | 4 | 1 | 0.25 |
| Sao | C1862323 |  |  | 1 |  |
| OnabotulinumtoxinA | C2719767 |  | 1 | 1 | 1 |
| hole | 80757 | 1 | 1 | 1 | 1 |
| OBs | 1009 |  | 1 | 1 | 1 |
| monoamine oxidase-A | 29253 | 1 | 1 | 1 | 1 |
| MAO-A | 29253 | 1 | 1 | 1 | 1 |
| dopamine-beta hydroxylase | C0013032 | 1 | 2 | 1 | 0.5 |
| PLI | 5345 |  | 1 | 1 | 1 |
| aminoacids | C0002520 | 3 | 2 | 1 | 0.5 |
| essential amino acid | C0002525 |  | 1 | 1 | 1 |
| Ba2 | 928 |  |  | 1 |  |
| Zhichan | 64663 | 2 | 3 | 1 | 0.333333333333333 |
| signal transduction protein | 64663 | 2 | 3 | 1 | 0.333333333333333 |
| NE-PAL | C3538705 |  |  | 1 |  |
| SPCS | 114131 |  |  | 1 |  |
| Pax6 gene | 5080 |  |  | 1 |  |
| Pax6 | 5080 |  |  | 1 |  |
| ACMSD-TMEM163 | 130013 |  |  | 1 |  |
| Hip | C3538851 |  |  | 1 |  |
| EPD | 501 | 1 | 1 | 1 | 1 |
| CGT | 7368 |  |  | 1 |  |
| ATP- | 51761 | 3 | 4 | 1 | 0.25 |
| CI = .20 | 374291 |  |  | 1 |  |
| FcgammaRI | 2209 |  |  | 1 |  |
| FcgammaRIIB | 2213 |  |  | 1 |  |
| antigen-antibody complexes | C0003313 |  |  | 1 |  |
| Cul1 | 8454 |  |  | 1 |  |
| F box protein | C1257945 |  | 1 | 1 | 1 |
| SCF) | 4254 | 1 |  | 1 |  |
| Vps35 | 853287 |  |  | 1 |  |
| dMyc | 31310 |  |  | 1 |  |
| proto-oncogene | C0033713 |  |  | 1 |  |
| NCAM | 4684 |  |  | 1 |  |
| H19 | 283120 |  |  | 1 |  |
| lincRNA-p21 | 102800311 |  |  | 1 |  |
| SNHG1 | 23642 |  |  | 1 |  |
| TncRNA | 283131 |  |  | 1 |  |
| Microtubule Actin Cross-linking Factor 1 | 23499 |  |  | 1 |  |
| MACF1 | 23499 |  |  | 1 |  |
| MACF1b | 23499 |  |  | 1 |  |
| MACF1a | 23499 |  |  | 1 |  |
| vab-10 | 173058 |  |  | 1 |  |
| MACF1's | 23499 |  |  | 1 |  |
| mGlu5) | 2915 | 3 | 1 | 1 | 1 |
| III | 972 |  | 1 | 1 | 1 |
| Tor1 | 853529 |  |  | 1 |  |
| IRS-1 | 25467 |  |  | 1 |  |
| IRS-2 | 29376 | 1 |  | 1 |  |
| BIRC5 | 332 |  |  | 1 |  |
| survivin | 332 |  |  | 1 |  |
| BIRC3 genes | 330 |  |  | 1 |  |
| homeodomain transcription factor 3 gene | C0017347 |  |  | 1 |  |
| DNA repair proteins | C1366764 |  | 1 | 1 | 1 |
| essential amino acids | C0002525 |  | 1 | 1 | 1 |
| alpha-methyl-p-tyrosine | C0051427 | 1 |  | 1 |  |
| alphaMpT | C0051427 | 1 |  | 1 |  |
| myrosinase | C0076469 |  |  | 1 |  |
| DURO-TAK | 1025 | 1 |  | 1 |  |
| Cav3.1 | 8913 |  |  | 1 |  |
| Cav3 | 859 |  |  | 1 |  |
| GC gene | 2638 |  | 1 | 1 | 1 |
| vancomycin | C0042313 |  |  | 1 |  |
| glucose transporter type 4 | C0166441 |  |  | 1 |  |
| GLUT4 | 6517 | 1 |  | 1 |  |
| SGLT1 | 6523 |  |  | 1 |  |
| STF-31 | C0599939 |  |  | 1 |  |
| Caspase-7 | 12369 |  |  | 1 |  |
| AIF | 26926 |  |  | 1 |  |
| IL-1ra | 16181 |  |  | 1 |  |
| H3 receptor | C0062739 | 1 |  | 1 |  |
| H3 receptors | C0062739 | 1 |  | 1 |  |
| H3-NMDA receptor | C0062739 | 1 |  | 1 |  |
| a 1.9-2. | 27239 | 2 |  | 1 |  |
| RARbeta | 218772 |  |  | 1 |  |
| retinoic acid receptors | C0140278 |  |  | 1 |  |
| RAR | 5914 |  |  | 1 |  |
| mGluR3 | 53623 |  |  | 1 |  |
| ARHGEF7 | 8874 |  | 1 | 1 | 1 |
| guanine nucleotide exchange factor | C0120465 |  | 1 | 1 | 1 |
| PCS | 8075 |  |  | 1 |  |
| AC-PC | C2350443 |  |  | 1 |  |
| 5-HT6 receptor | C1143376 |  |  | 1 |  |
| dopamine D1/D5 receptor- | C0114836 | 1 |  | 1 |  |
| CaMKIV | 814 |  |  | 1 |  |
| metabotropic glutamate receptor type 1 | C0535210 |  |  | 1 |  |
| dipeptidyl peptidase IV | 1803 |  |  | 1 |  |
| DPP IV | 1803 |  |  | 1 |  |
| Na+ /H+ Exchanger | C0074785 |  |  | 1 |  |
| NHE1 | 6548 |  |  | 1 |  |
| Na+ /H+ exchanger 1 | 20544 |  |  | 1 |  |
| NHE1 | 20544 |  |  | 1 |  |
| NHE | 285335 |  |  | 1 |  |
| NHE1 protein | 6548 |  |  | 1 |  |
| Lhx | 5862 |  |  | 1 |  |
| GATA | 55278 |  |  | 1 |  |
| Lmo3 | 55885 |  |  | 1 |  |
| NR1 | 2902 |  |  | 1 |  |
| pNR1 | 2902 |  |  | 1 |  |
| calmodulin-dependent protein kinase | C0769224 |  | 1 | 1 | 1 |
| GroES | 3336 |  |  | 1 |  |
| kynurenine aminotransferase | C0064450 |  |  | 1 |  |
| KAT-2 | 23923 |  |  | 1 |  |
| KAT-2 | 29416 |  |  | 1 |  |
| ERO | 23596 |  |  | 1 |  |
| cho-1 | 9493 |  |  | 1 |  |
| unc-50 | 25972 |  |  | 1 |  |
| ace-2 | 59272 |  |  | 1 |  |
| NALCN | 259232 |  |  | 1 |  |
| ng ml-1 | C0964553 |  |  | 1 |  |
| HEK- | 2042 | 1 |  | 1 |  |
| proteins tau | 10971 | 1 | 1 | 1 | 1 |
| Mage | C3812726 |  |  | 1 |  |
| ANO3 | 63982 |  |  | 1 |  |
| sphingosine-1-phosphate 1 receptor | C0390526 |  |  | 1 |  |
| S1PR1 | 13609 |  |  | 1 |  |
| sphingosine-1-phosphate receptor | C0390526 |  |  | 1 |  |
| S1PR | C0390526 |  |  | 1 |  |
| P = 0.165 | C1527371 |  |  | 1 |  |
| MDS 2 | 259283 |  |  | 1 |  |
| Parkinson's disease (PD)-related protein F-box only protein 7 | C3538910 |  |  | 1 |  |
| Cullin-F-box protein | C1257952 |  |  | 1 |  |
| VCAM-1 | 22329 |  |  | 1 |  |
| ICAM-1 | 15894 |  |  | 1 |  |
| CD20 | 931 |  |  | 1 |  |
| Epstein-Barr nuclear antigen 2 | C3641847 |  |  | 1 |  |
| viral protein | C0042736 |  | 1 | 1 | 1 |
| lysosome-associated membrane proteins | C1566771 |  |  | 1 |  |
| LAMP1 | 3916 |  |  | 1 |  |
| DC-LAMP | 27074 |  | 2 | 1 | 0.5 |
| heat shock factor-1 | 3297 | 3 |  | 1 |  |
| CF-PC | C3811819 |  |  | 1 |  |
| MIR886 | 100126299 |  |  | 1 |  |
| phosphodiesterase 4D | 5144 |  |  | 1 |  |
| PDE4D | 5144 |  |  | 1 |  |
| TRIM34 | 53840 |  |  | 1 |  |
| ND1 | 4535 |  |  | 1 |  |
| ND2 | 4536 |  |  | 1 |  |
| EP4 | 10406 |  |  | 1 |  |
| prostaglandin PGE2 receptor EP4 | 5734 |  |  | 1 |  |
| SBP | 25540 |  |  | 1 |  |
| MAG | C0129439 |  |  | 1 |  |
| myelin-associated glycoprotein | 4099 |  |  | 1 |  |
| MAG antibodies | C0443892 |  |  | 1 |  |
| HIF-1alpha | 15251 |  |  | 1 |  |
| VGluT1 proteins | 57030 | 1 |  | 1 |  |
| calcium handling proteins | C2064889 |  | 1 | 1 | 1 |
| galactocerebrosidase | C0016957 |  |  | 1 |  |
| alpha-glucosidase | C0002272 |  |  | 1 |  |
| sphingomyelinase | C0037903 |  | 1 | 1 | 1 |
| alpha-iduronidase | 3425 |  |  | 1 |  |
| GLA | 9027 |  |  | 1 |  |
| Peripheral Myelin | C0301710 |  |  | 1 |  |
| myelin proteins | C0026972 |  |  | 1 |  |
| histone deacetylase 3 | 8841 |  |  | 1 |  |
| guanosine triphosphatase | C3825040 | 1 |  | 1 |  |
| HDAC3 | 8841 |  |  | 1 |  |
| Lys-5 | 60496 |  |  | 1 |  |
| karyopherin subunit alpha2 | 3838 |  |  | 1 |  |
| myocyte-specific enhancer factor 2D | C0250373 |  |  | 1 |  |
| C11-BODIPY581 | C0061878 |  |  | 1 |  |
| DRG | 4733 |  |  | 1 |  |
| cyclooxygenase type 2 | C0387583 |  |  | 1 |  |
| Leu-Ala | C1173192 |  |  | 1 |  |
| TAK-1 | 26409 |  |  | 1 |  |
| Rip2 | 8767 |  |  | 1 |  |
| muramyl dipeptide | C0001060 |  |  | 1 |  |
| meso-diaminopimelic acid | C0011961 |  |  | 1 |  |
| Nod1/2 | 199713 |  |  | 1 |  |
| CD200R | 57781 |  |  | 1 |  |
| Toll-like receptor 2 | C0754728 |  |  | 1 |  |
| rabbit polyclonal antibody | C1979003 |  |  | 1 |  |
| LEP | 3952 | 2 | 1 | 1 | 1 |
| RAB39B | 116442 |  |  | 1 |  |
| protomers | C1136161 |  | 1 | 1 | 1 |
| Leu-Enk-Arg | 27 |  |  | 1 |  |
| phosphoramidon | C0070915 |  |  | 1 |  |
| dynorphin converting enzyme | C0058855 |  |  | 1 |  |
| opiorphin | C1871154 |  |  | 1 |  |
| aminopeptidase N | C0054943 |  |  | 1 |  |
| ADAMTS19 | 171019 |  |  | 1 |  |
| IAP | 39753 |  |  | 1 |  |
| keyhole limpet hemocyanin | C0064332 |  |  | 1 |  |
| Sirtuin 5 | 23408 |  |  | 1 |  |
| SIRT5 | 23408 |  |  | 1 |  |
| MCU | 768182 |  |  | 1 |  |
| VDAC1 | 334582 |  |  | 1 |  |
| micu1 | 561210 |  |  | 1 |  |
| G4-5 | 6901 |  |  | 1 |  |
| SCA36 | 10528 |  |  | 1 |  |
| SARA | 56681 |  |  | 1 |  |
| activity-dependent neuroprotective protein | 23394 |  |  | 1 |  |
| ADNP | 23394 |  |  | 1 |  |
| TREM2 | 54209 |  |  | 1 |  |
| GLP | 79813 |  |  | 1 |  |
| PLCbeta1 | 23236 |  |  | 1 |  |
| M1/2/4 receptors | C0531859 |  | 1 | 1 | 1 |
| MCP's | 822 | 1 |  | 1 |  |
| Decarboxylases | C0007054 | 2 |  | 1 |  |
| SP1058 | 931572 |  |  | 1 |  |
| MAF | 4094 |  |  | 1 |  |
| Nur77 | 3164 |  |  | 1 |  |
| Orphan nuclear receptor Nur77 | 3164 |  |  | 1 |  |
| Nur77 | 15370 |  |  | 1 |  |
| VAChT | 20508 |  |  | 1 |  |
| VAChTEn1 | 6572 |  |  | 1 |  |
| Fbxl18 | 80028 |  |  | 1 |  |
| Cullin1 | 8454 |  |  | 1 |  |
| CSF-PR 2 | 3918 |  |  | 1 |  |
| csf-pr-2 | 3918 |  |  | 1 |  |
| catechol-O-methyltransferase | 100412305 |  |  | 1 |  |
| COMT | 100412305 |  |  | 1 |  |
| VIPR1 | 7433 |  |  | 1 |  |
| VIPR2 | 7434 |  |  | 1 |  |
| apurinic/apyrimidinic endonuclease 1 | 79116 |  |  | 1 |  |
| PVRL2 | 5819 |  |  | 1 |  |
| DEGs | 58970 |  |  | 1 |  |
| TRPC3 | 22065 |  |  | 1 |  |
| ADAR2 | 104 |  |  | 1 |  |
| calpain inhibitor III | C1700414 |  |  | 1 |  |
| WASH | 653440 |  |  | 1 |  |
| ANKRD50 | 57182 |  |  | 1 |  |
| ankyrin-repeat-domain-containing protein 50 | 57182 |  |  | 1 |  |
| SNX27 | 81609 |  |  | 1 |  |
| torsin | 1861 | 2 | 1 | 1 | 1 |
| Transforming Growth Factor beta2 | 7042 |  |  | 1 |  |
| TGFbeta2 | 7042 |  |  | 1 |  |
| IDE | 3416 |  |  | 1 |  |
| pro-inflammatory cytokine TNFalpha | C0378389 | 1 |  | 1 |  |
| EPP | 8288 |  |  | 1 |  |
| Opa1 | 74143 |  |  | 1 |  |
| S15 | 6209 |  |  | 1 |  |
| SNO | 20622 |  |  | 1 |  |
| Dpr1 | 51339 |  |  | 1 |  |
| FBXO7 | 38443 |  |  | 1 |  |
| PI31 | 36277 |  |  | 1 |  |
| unc-54p | 259839 |  |  | 1 |  |
| 4-Dihydroxy-6-18F-fluoro-l-phenylalanine | C0030137 |  |  | 1 |  |
| IL-23 p19 | 51561 |  |  | 1 |  |
| Interleukin (IL)-23 | C1882965 |  |  | 1 |  |
| p19 | 83430 |  | 1 | 1 | 1 |
| IL-12 p40 | 16160 |  | 1 | 1 | 1 |
| Apo | 104237 |  |  | 1 |  |
| IL-23 p19 | 83430 |  | 1 | 1 | 1 |
| l-leucine | C0023401 | 2 | 7 | 1 | 0.142857142857143 |
| PD patients 1 | 8825 | 1 | 2 | 1 | 0.5 |
| 5-Mar | 54708 |  |  | 1 |  |
| GNE | 50798 |  |  | 1 |  |
| Interleukin (IL)-32 | 9235 |  |  | 1 |  |
| IL | C1527200 |  |  | 1 |  |
| AR | C0002003 |  |  | 1 |  |
| peroxiredoxin 2 | 21672 |  |  | 1 |  |
| Prx2 | 20204 |  |  | 1 |  |
| bGP | C0917785 |  | 1 | 1 | 1 |
| MT-ND4 | 4538 |  |  | 1 |  |
| MT-TL1 genes | 4567 |  |  | 1 |  |
| MT-TL1 | 4567 |  |  | 1 |  |
| MT-ND4 gene | 4538 |  |  | 1 |  |
| mitochondrially encoded NADH dehydrogenase 4 | 4538 |  |  | 1 |  |
| tRNA leucine 1 | 7207 |  |  | 1 |  |
| beta -4. | 10717 |  |  | 1 |  |
| eras | 3266 |  |  | 1 |  |
| NF-kappaB p52 | 2288 |  |  | 1 |  |
| NF-kappaB p52 subunit | 2288 |  |  | 1 |  |
| DNA methyltransferase 3a | C1743053 |  |  | 1 |  |
| ten-eleven translocation 1 | 80312 |  |  | 1 |  |
| TET1 | 80312 |  |  | 1 |  |
| Hsp31p | 852146 |  |  | 1 |  |
| HSP31 | 852146 |  |  | 1 |  |
| Cad1p | 852033 |  |  | 1 |  |
| Msn2p | 855053 |  |  | 1 |  |
| Msn4p | 853803 |  |  | 1 |  |
| Haa1p | 856117 |  |  | 1 |  |
| Hsf1p | 852806 |  | 1 | 1 | 1 |
| Hsp32p | 855849 |  |  | 1 |  |
| glo1 | 855009 |  |  | 1 |  |
| small ubiquitin-related modifier-1 | C0534100 | 1 |  | 1 |  |
| SUMO-1 | 22218 |  |  | 1 |  |
| CD163 | 93671 |  |  | 1 |  |
| CD204 | 4481 |  |  | 1 |  |
| fibroblast growth factor 8 | C0287829 |  |  | 1 |  |
| calreticulin | 811 |  |  | 1 |  |
| malate dehydrogenase | C0024544 |  |  | 1 |  |
| MDH | C0024544 |  |  | 1 |  |
| PRPH | 5630 |  |  | 1 |  |
| IRE1 | 26918 |  |  | 1 |  |
| Abcd2 | 26874 |  |  | 1 |  |
| NF-E2 | 18022 |  |  | 1 |  |
| interleukin 1 | C1522428 | 1 |  | 1 |  |
| bFGF | 54250 | 1 | 1 | 1 | 1 |
| basic fibroblast growth factor | 54250 | 1 | 1 | 1 | 1 |
| Slc17a6 | 84487 |  |  | 1 |  |
| vGluT2 | 84487 |  |  | 1 |  |
| NIPA1 | 123606 |  |  | 1 |  |
| MagT1 | 84061 |  |  | 1 |  |
| CNNM2 | 54805 |  |  | 1 |  |
| Parp | 3355109 |  |  | 1 |  |
| DLG2 | 1740 |  |  | 1 |  |
| MC1R | 17199 |  |  | 1 |  |
| lipases | C0023764 |  |  | 1 |  |
| Polylysine | C0032518 |  |  | 1 |  |
| viral gene | C0017376 |  |  | 1 |  |
| alpha-Syn lipoprotein | C0023821 | 2 |  | 1 |  |
| MRS | 1667 |  |  | 1 |  |
| pattern-recognition receptors | C1564907 |  |  | 1 |  |
| Type-I interferons | C0021743 |  |  | 1 |  |
| type-I IFNs | C0021743 |  |  | 1 |  |
| IFNAR1 | 15975 |  |  | 1 |  |
| IFNAR1 | 3454 |  |  | 1 |  |
| Cysteinyldopa | C0010665 |  |  | 1 |  |
| Cys-DOPA | C0010665 |  |  | 1 |  |
| ERK1 | 50689 |  | 1 | 1 | 1 |
| ERK2 | 116590 |  |  | 1 |  |
| TH enzyme inhibitor | C1519723 | 1 |  | 1 |  |
| aMT | 275 |  |  | 1 |  |
| dopamine-beta-hydroxylase | 574105 |  |  | 1 |  |
| had 1 | 22232 |  |  | 1 |  |
| HGF | 443075 |  |  | 1 |  |
| GHR | 443333 |  |  | 1 |  |
| IGF1R | 443515 |  |  | 1 |  |
| T=3. | C0041014 | 1 | 2 | 1 | 0.5 |
| PDI | C0072354 |  | 2 | 1 | 0.5 |
| CDK5 | C0249586 |  |  | 1 |  |
| p25 | 170496 |  |  | 1 |  |
| Sideroflexin 3 | 81855 |  |  | 1 |  |
| sideroflexin 3 | 94280 |  |  | 1 |  |
| SFXN3 | 94280 |  |  | 1 |  |
| Gadd45b | 4616 |  |  | 1 |  |
| Parp2 | 10038 |  |  | 1 |  |
| Parp3 | 10039 |  |  | 1 |  |
| Adam10 | 102 |  |  | 1 |  |
| Mmp11 | 4320 |  |  | 1 |  |
| sphingosine kinase 1 | C0897751 |  |  | 1 |  |
| Sphk1 | 8877 |  |  | 1 |  |
| Kv4.3 potassium channels | 3746 |  |  | 1 |  |
| Kv4.3 | 56543 |  |  | 1 |  |
| Mitofusin-2 | 170731 |  | 2 | 1 | 0.5 |
| p62 | 18412 |  |  | 1 |  |
| SQSTM1 | 18412 |  |  | 1 |  |
| ATG9 | 245860 |  |  | 1 |  |
| serotonin receptor 3A | 3359 |  |  | 1 |  |
| serotonin receptor 4 | 3360 |  |  | 1 |  |
| CD184 | 7852 |  |  | 1 |  |
| miR-15a | 406948 |  |  | 1 |  |
| miR-23a | 407010 |  |  | 1 |  |
| Cadherin | C0006631 |  |  | 1 |  |
| plasma albumin | C1260311 |  |  | 1 |  |
| adhesion molecule 1 | 23705 |  |  | 1 |  |
| TOM-40 | 53333 |  |  | 1 |  |
| TOM-20 | 67952 |  |  | 1 |  |
| TIM-23 | 53600 |  |  | 1 |  |
| COX-I | 17708 |  |  | 1 |  |
| COX-IV | 12857 |  |  | 1 |  |
| mtHSP70 | 15526 |  |  | 1 |  |
| nlp | 22981 |  |  | 1 |  |
| Prohibitin | 5245 |  |  | 1 |  |
| Prohibitin | 18673 |  |  | 1 |  |
| 14-3-3zeta | 7534 | 1 |  | 1 |  |
| Ndufs3 | 4722 |  |  | 1 |  |
| AM-404 | 3170950 |  |  | 1 |  |
| snail | 6615 |  |  | 1 |  |
| troponin I | C0077401 |  |  | 1 |  |
| FBP1 | 2203 |  |  | 1 |  |
| P80 | 57599 |  |  | 1 |  |
| P140 | 3812 |  |  | 1 |  |
| imprinting genes | C1708477 |  |  | 1 |  |
| insulin-like growth factor 2 | 3481 | 1 |  | 1 |  |
| thioredoxin-1 | 116484 |  | 1 | 1 | 1 |
| Trx-1 | 22166 |  | 1 | 1 | 1 |
| vascular cell adhesion molecule-1 | 25361 |  |  | 1 |  |
| VCAM-1 | 25361 |  |  | 1 |  |
| intercellular adhesion molecule-1 | 25464 |  | 1 | 1 | 1 |
| ICAM-1 | 25464 |  | 1 | 1 | 1 |
| 5-HT7 Receptor | C0535922 |  |  | 1 |  |
| 5-HT7 receptors | C0535922 |  |  | 1 |  |
| macrophage-derived chemokine | C1098076 |  |  | 1 |  |
| interleukin (IL)-4 | C0021758 |  |  | 1 |  |
| IL12P70 | C0123759 |  | 1 | 1 | 1 |
| RANTES | 6352 |  | 1 | 1 | 1 |
| ADCS | 113451 | 1 |  | 1 |  |
| MOB | 259230 |  |  | 1 |  |
| beta-gal | C0005220 | 1 |  | 1 |  |
| sex determining region Y- | 6736 |  | 1 | 1 | 1 |
| regulator gene | C0017362 |  | 2 | 1 | 0.5 |
| MEAN-SIM | 6493 |  |  | 1 |  |
| PIKE-L | 116986 |  |  | 1 |  |
| phosphoinositide-3 kinase enhancer | 116986 |  |  | 1 |  |
| PIKE | 216439 |  |  | 1 |  |
| pump protein | C2945682 |  |  | 1 |  |
| C1q | 712 |  |  | 1 |  |
| macrophage inflammatory protein 1-beta | 6351 |  | 1 | 1 | 1 |
| MIP-1-beta | 6351 |  | 1 | 1 | 1 |
| chaperonin CCT | C2717814 |  |  | 1 |  |
| chaperonin containing TCP-1 | 6690 |  | 1 | 1 | 1 |
| CCT | 907 |  |  | 1 |  |
| Axin2 | 12006 |  |  | 1 |  |
| Nesfatin-1 | 4925 |  |  | 1 |  |
| C-Raf | 22882 |  | 1 | 1 | 1 |
| nesfatin-1 | 53322 |  |  | 1 |  |
| C-Raf | 110157 |  |  | 1 |  |
| HY3 | 6085 |  |  | 1 |  |
| FIM | 7750 |  |  | 1 |  |
| complexin 1 | 10815 |  |  | 1 |  |
| CPLX1 | 10815 |  |  | 1 |  |
| GZMH | 2999 |  |  | 1 |  |
| PLTP | 5360 |  |  | 1 |  |
| alpha1 subunit-containing GABAA receptor | 2554 |  |  | 1 |  |
| alpha1 subunit-containing GABAA receptors | 2554 |  |  | 1 |  |
| SIRT1 | 797132 |  |  | 1 |  |
| M3 muscarinic receptor | 1131 |  |  | 1 |  |
| CI-M6PR | 3482 |  | 1 | 1 | 1 |
| cation-independent mannose 6-phosphate receptor | 3482 |  | 1 | 1 | 1 |
| RAD51B | 5890 |  |  | 1 |  |
| RAD51 | 5888 |  |  | 1 |  |
| iNSC | 233752 |  |  | 1 |  |
| GAD-67 | 2571 | 1 | 1 | 1 | 1 |
| Nicotinamide N-Methyltransferase | 4837 |  |  | 1 |  |
| NNMT | 4837 |  |  | 1 |  |
| SAM) | C0036002 | 5 | 2 | 1 | 0.5 |
| apoenzyme | C0003589 |  |  | 1 |  |
| SAM-dependent methyltransferase | 80745 |  |  | 1 |  |
| snap29 | 553460 |  |  | 1 |  |
| aifm3 | 100150876 |  |  | 1 |  |
| crkl | 556443 |  |  | 1 |  |
| CRKL | 1399 |  |  | 1 |  |
| Crkl | 12929 |  |  | 1 |  |
| SNAP29 | 9342 |  |  | 1 |  |
| AIFM3 | 150209 |  |  | 1 |  |
| ZNF746 | 69228 |  |  | 1 |  |
| Olfactory Marker Protein | 18378 |  |  | 1 |  |
| Olfactory Marker Protein | C0069416 |  |  | 1 |  |
| glucosidase | C0017764 |  |  | 1 |  |
| receptor (DR) gene | C1335671 |  | 1 | 1 | 1 |
| IL-1alpha | 16175 |  | 1 | 1 | 1 |
| interleukin 1alpha | C0600251 |  |  | 1 |  |
| uchl1 | 325119 |  |  | 1 |  |
| TiO2NPs | 594857 |  |  | 1 |  |
| PAR polymerase-1 | 2149 |  |  | 1 |  |
| EC-PAR | 8856 |  |  | 1 |  |
| Na+/H+ Exchanger 9 | 285195 |  |  | 1 |  |
| NHE9 | 285195 |  |  | 1 |  |
| HLA-DRB5 gene | 3122 |  | 2 | 1 | 0.5 |
| BTNL2 gene | 56244 |  |  | 1 |  |
| RAB38 | 23682 |  |  | 1 |  |
| CTSC gene | 1075 |  |  | 1 |  |
| A10 | 28870 |  |  | 1 |  |
| mesencephalic astrocyte-derived neurotrophic factor | 315989 |  |  | 1 |  |
| MANF | 315989 |  |  | 1 |  |
| PARK18 | 1981 |  | 4 | 1 | 0.25 |
| ascorbate peroxidase | C3178941 |  |  | 1 |  |
| miR | 22877 |  |  | 1 |  |
| SUMO1 | 301442 |  |  | 1 |  |
| SUMO2 | 690244 |  |  | 1 |  |
| Ubc9 | 7329 |  |  | 1 |  |
| aiding | C3540469 | 3 |  | 1 |  |
| GPATCH2L | 55668 |  |  | 1 |  |
| UHRF1BP1L | 23074 |  |  | 1 |  |
| PTPRH | 5794 |  |  | 1 |  |
| ARSB | 411 |  |  | 1 |  |
| STN beta | 85439 |  |  | 1 |  |
| perforin | C0070410 |  |  | 1 |  |
| Intracellular signaling proteins | C1449859 |  |  | 1 |  |
| fatty acid synthase | C0015683 |  |  | 1 |  |
| FASN | 14104 |  |  | 1 |  |
| FASN | 2194 |  |  | 1 |  |
| S100A10 | 6281 | 3 | 1 | 1 | 1 |
| GADD34 | 171071 |  |  | 1 |  |
| ment | 54964 |  |  | 1 |  |
| Auxilin | 40527 |  |  | 1 |  |
| G-associated kinase | C0010536 |  | 1 | 1 | 1 |
| GAK | C0010536 |  | 1 | 1 | 1 |
| aux | C0208529 |  |  | 1 |  |
| Yeast prions | C0872306 |  | 2 | 1 | 0.5 |
| ion channel proteins | C1334283 |  |  | 1 |  |
| stefin B | C0075212 |  |  | 1 |  |
| Nedd4-2 | 23327 |  |  | 1 |  |
| isocitrate dehydrogenase 2 | 3418 |  |  | 1 |  |
| IDH2 | 3418 |  |  | 1 |  |
| MICD | 4279 |  |  | 1 |  |
| caspase12 | 12364 |  | 1 | 1 | 1 |
| Mitochondrial ferritin | 94033 |  |  | 1 |  |
| FtMt | 94033 |  |  | 1 |  |
| Calbindin-D28k | 793 | 1 | 1 | 1 | 1 |
| Recombinant CD200 fusion protein | C1571593 | 1 |  | 1 |  |
| CD200 | 24560 |  |  | 1 |  |
| CD200R1 | 64357 |  |  | 1 |  |
| phycocyanin | C0031788 |  |  | 1 |  |
| C-PC | C0031788 |  |  | 1 |  |
| catalase | 40048 |  |  | 1 |  |
| BRIL | 387733 |  |  | 1 |  |
| apolipoprotein A1 | C0085201 | 1 | 1 | 1 | 1 |
| Notch1-IC protein | C0144507 |  |  | 1 |  |
| DA receptor D4 | 1815 |  | 1 | 1 | 1 |
| DRD4 | 1815 |  | 1 | 1 | 1 |
| serotonin receptor 1B | 3351 |  | 1 | 1 | 1 |
| HTR1B | 3351 |  | 1 | 1 | 1 |
| SLC6A2 | 6530 | 1 |  | 1 |  |
| cadherin 13 | 1012 |  | 1 | 1 | 1 |
| CDH13 | 1012 |  | 1 | 1 | 1 |
| CD25 | 3559 |  |  | 1 |  |
| PDK2 | 5164 | 1 |  | 1 |  |
| PACT | 5930 |  |  | 1 |  |
| heme oxygenase-2 | 3163 |  |  | 1 |  |
| HMOX2 | 3163 |  |  | 1 |  |
| miR-137 | 406928 |  |  | 1 |  |
| miR-184 | 406960 |  |  | 1 |  |
| Janus kinase 2 | 16452 |  |  | 1 |  |
| CD24 | 12484 |  |  | 1 |  |
| SCN1A | 6323 |  |  | 1 |  |
| guanine nucleotide exchange factors | C0120465 |  | 1 | 1 | 1 |
| GEFs | C0120465 |  | 1 | 1 | 1 |
| FBP | 2203 |  |  | 1 |  |
| FORE-FBP | 2203 |  |  | 1 |  |
| BMP2- | C0527443 |  |  | 1 |  |
| Bone morphogenetic protein 2 | 650 |  |  | 1 |  |
| BMP2 | 650 |  |  | 1 |  |
| BMP receptors | C0289417 | 1 |  | 1 |  |
| BMP | 649 |  |  | 1 |  |
| BMP2- | 650 |  |  | 1 |  |
| TREM2 gene | 54209 |  |  | 1 |  |
| translocator protein | 12257 |  |  | 1 |  |
| TSPO | 12257 |  |  | 1 |  |
| Galectin-3 | C0245382 |  |  | 1 |  |
| mis- | 8449 | 1 |  | 1 |  |
| RING2 | 7923 |  |  | 1 |  |
| RING2-REP | 7923 |  |  | 1 |  |
| nexin | C0030190 |  |  | 1 |  |
| aspartate transaminase | C0004002 |  | 1 | 1 | 1 |
| alanine transaminase | C0001899 |  | 1 | 1 | 1 |
| signal recognition particle | C0074512 |  |  | 1 |  |
| Orosomucoid-2 | 5005 |  |  | 1 |  |
| Orosomucoid | C0029297 |  |  | 1 |  |
| acute-phase protein | C0001347 | 1 |  | 1 |  |
| ORM | C0029297 |  |  | 1 |  |
| Orm2 | 18406 |  |  | 1 |  |
| Orm1 | 18405 |  |  | 1 |  |
| Orm3 | 18407 |  |  | 1 |  |
| ORM2 | 5005 |  |  | 1 |  |
| cytokine gene | C1333196 | 1 | 2 | 1 | 0.5 |
| CCL4 | 20303 |  |  | 1 |  |
| C-C chemokine receptor type 5 | C0387687 |  |  | 1 |  |
| orosomucoid-2 | 18406 |  |  | 1 |  |
| anti-T. gondii IgG | C0051979 |  | 1 | 1 | 1 |
| Anti-T. gondii IgG antibodies | C0312594 |  |  | 1 |  |
| anti-T. gondii IgM antibodies | C0312594 |  |  | 1 |  |
| TMEM175 | 305623 |  |  | 1 |  |
| glucocerebrosidase | 684536 |  |  | 1 |  |
| Cg) | C3540479 |  |  | 1 |  |
| GBA2 | 57704 |  |  | 1 |  |
| CD34 | 12490 |  |  | 1 |  |
| ANK2 | 287 |  |  | 1 |  |
| Ankyrin-B | 287 |  |  | 1 |  |
| KCNQ1 | 3784 |  |  | 1 |  |
| ankyrin-B p | 287 |  |  | 1 |  |
| Na/Ca exchanger | 6546 |  |  | 1 |  |
| B = -2.43 | C0605660 |  |  | 1 |  |
| Th2 | 15111 |  |  | 1 |  |
| GTP | 92170 | 1 |  | 1 |  |
| Cannabinoid Receptor 2 | 1269 |  |  | 1 |  |
| CB2) receptors | C0208757 |  | 1 | 1 | 1 |
| CB2 receptor | C0208757 |  | 1 | 1 | 1 |
| neurofilament 200 | C0068589 |  | 2 | 1 | 0.5 |
| dATF4 | 47767 |  |  | 1 |  |
| ATF4 | 47767 |  |  | 1 |  |
| SHMT2 | 31524 |  |  | 1 |  |
| NMDMC | 47895 |  |  | 1 |  |
| glycyl-l-histidyl-l-lysine | C0617032 |  |  | 1 |  |
| decorin | C0057252 |  |  | 1 |  |
| GJB2 | 2706 |  |  | 1 |  |
| Cx26 | 2706 |  |  | 1 |  |
| Cav 1 | 779 |  |  | 1 |  |
| P2Y6R | C0390418 |  |  | 1 |  |
| NTS | C0027930 |  | 1 | 1 | 1 |
| peroxisome proliferators-activated receptors | C0166418 | 3 | 3 | 1 | 0.333333333333333 |
| calcium-binding protein nuclebindin-1 | 9478 |  |  | 1 |  |
| macromolecular complexes | C0751282 |  |  | 1 |  |
| MAFF | 23764 |  |  | 1 |  |
| cysteinyl-glycine | C0056886 |  |  | 1 |  |
| miR-19b | 406980 |  |  | 1 |  |
| miR-195 | 406971 |  |  | 1 |  |
| glucagon-like peptide 1 | 24952 |  |  | 1 |  |
| GLP-1 | 24952 |  |  | 1 |  |
| glucose dependent insulinotropic polypeptide | 25040 |  |  | 1 |  |
| GIP | 25040 |  |  | 1 |  |
| Glucosylceramide synthase | 7357 |  |  | 1 |  |
| glucosylceramide synthase | 22234 |  |  | 1 |  |
| GCS | 7357 |  |  | 1 |  |
| mind bomb-2 | 76580 |  |  | 1 |  |
| Mib2 | 76580 |  |  | 1 |  |
| FoxO1 | 56458 |  |  | 1 |  |
| Ucp1 | 22227 |  | 1 | 1 | 1 |
| Uncoupling protein-1 | C0107264 |  |  | 1 |  |
| XIAP protein | 331 |  | 2 | 1 | 0.5 |
| complement C5a and C5a receptor | 727 |  |  | 1 |  |
| C5aR1 | 12273 |  |  | 1 |  |
| C5aR | 12273 |  |  | 1 |  |
| C5a | 727 |  |  | 1 |  |
| C5aR1 | 728 |  |  | 1 |  |
| F-actin | C1180307 |  |  | 1 |  |
| SMAD2 | 4087 |  |  | 1 |  |
| Sac | 55811 |  |  | 1 |  |
| PARK19 | 9829 |  |  | 1 |  |
| Mfn2 | 64476 |  |  | 1 |  |
| Mitofusin 2 | 64476 |  |  | 1 |  |
| angiotensin type-1 (AT1) receptors | C0529330 | 1 | 3 | 1 | 0.333333333333333 |
| angiotensin receptors | C0034787 |  |  | 1 |  |
| AT1 | C0003018 | 1 |  | 1 |  |
| CaV3.2 | 58226 |  |  | 1 |  |
| DHPs | 1725 |  |  | 1 |  |
| tight junction protein Zonula Occludens-1 | C3503764 |  |  | 1 |  |
| lipopolysaccharide specific inflammatory receptor | C0108768 |  | 1 | 1 | 1 |
| OR 1.3 | 128360 |  |  | 1 |  |
| STAT1 | 20846 |  |  | 1 |  |
| KuA | 387521 |  |  | 1 |  |
| Ifng | 25712 | 1 |  | 1 |  |
| beta-CD | 63857 |  |  | 1 |  |
| Galphaolf | 14680 |  | 1 | 1 | 1 |
| G-protein alpha subunit | C0887847 |  |  | 1 |  |
| Galphaolf | C0249519 |  |  | 1 |  |
| stimulatory GTP-binding protein | 92170 | 1 |  | 1 |  |
| BM88 | 57754 |  | 1 | 1 | 1 |
| CHRNB2 | 11444 |  |  | 1 |  |
| CEPA | C1413345 |  |  | 1 |  |
| GD3 | 117189 |  |  | 1 |  |
| GD3 synthase | 20449 |  |  | 1 |  |
| GD3S | 20449 |  |  | 1 |  |
| sialidase | C0027803 |  |  | 1 |  |
| CB2 | 1269 |  |  | 1 |  |
| Hp | C0018595 |  |  | 1 |  |
| beta-barrel protein | C2362547 |  |  | 1 |  |
| killers | 8795 | 1 |  | 1 |  |
| GluA2 | 29627 |  |  | 1 |  |
| cysteine protease | C0758959 |  |  | 1 |  |
| cullin-ring ligase (CRL) adaptor proteins | C1257952 |  |  | 1 |  |
| IkappaBalpha | C0126732 |  |  | 1 |  |
| Synaptic vesicle glycoprotein 2C | 22987 |  |  | 1 |  |
| SV2C | 22987 |  |  | 1 |  |
| SV2A | 64051 |  |  | 1 |  |
| SV2C | 75209 |  |  | 1 |  |
| DJ-1 (TgDJ-1) at 2 | 3301 | 1 |  | 1 |  |
| CDPK1 | C0054490 |  | 1 | 1 | 1 |
| calcium-dependent protein kinases | C0054490 |  | 1 | 1 | 1 |
| BP180 | 1308 |  |  | 1 |  |
| BPAG2 | 1308 |  |  | 1 |  |
| BP230 | C3539739 |  |  | 1 |  |
| adrenoceptor | C0034783 |  | 1 | 1 | 1 |
| resistin- | 56729 |  |  | 1 |  |
| Fizz1 | 84666 |  |  | 1 |  |
| Ym1 | 1128 |  | 1 | 1 | 1 |
| IL-13 | C0214743 |  |  | 1 |  |
| NLRP3 | 287362 |  |  | 1 |  |
| pyrin | C0665818 |  |  | 1 |  |
| Ac-YVAD-CMK | 4283 |  |  | 1 |  |
| glycogen synthase kinase-3beta | 478575 |  |  | 1 |  |
| Gsk3beta | 478575 |  |  | 1 |  |
| PVA | 1830 |  |  | 1 |  |
| Nec-1 | 5122 |  |  | 1 |  |
| receptor-interacting serine/threonine-protein kinase 1/ | 8737 |  | 1 | 1 | 1 |
| cystathionine-gamma-lyase | C0010642 |  | 1 | 1 | 1 |
| CSE | 1433 |  |  | 1 |  |
| SOS | 64132 |  |  | 1 |  |
| ERIC | 104355217 |  |  | 1 |  |
| SP6 | 80320 |  | 1 | 1 | 1 |
| LI4 | 643418 |  |  | 1 |  |
| PC6 | 5125 |  |  | 1 |  |
| synaptotagmin IV | 6860 |  |  | 1 |  |
| Syt IV | 64440 | 1 |  | 1 |  |
| Syt XI | 60568 |  |  | 1 |  |
| Syt IV | 20983 |  |  | 1 |  |
| reg- | 5967 |  |  | 1 |  |
| FOXA1 | 3169 |  |  | 1 |  |
| CEL | 1056 | 2 | 1 | 1 | 1 |
| gene/locus | C1708726 | 1 | 2 | 1 | 0.5 |
| MPHOSPH10 | 10199 |  |  | 1 |  |
| TAS2R19 | 259294 |  |  | 1 |  |
| SERPINA1 genes | 5265 |  | 2 | 1 | 0.5 |
| SERPINA1 | 5265 |  | 2 | 1 | 0.5 |
| Sirtuin 2 | 361532 |  |  | 1 |  |
| MT3 | 117038 |  |  | 1 |  |
| Eag1 | 3756 |  | 1 | 1 | 1 |
| miR-34a | 407040 |  | 1 | 1 | 1 |
| microRNA34a | 407040 |  | 1 | 1 | 1 |
| miR34a | 407040 |  | 1 | 1 | 1 |
| Eag1targeted | 3756 |  | 1 | 1 | 1 |
| EAG1hum | 3756 |  | 1 | 1 | 1 |
| Bone morphogenetic protein 6 | C0537467 |  |  | 1 |  |
| miR203 | 406986 |  |  | 1 |  |
| neuronal growth regulator 1 | 257194 |  |  | 1 |  |
| phosphatidic acid phosphatase | C0031607 |  |  | 1 |  |
| plateletderived growth factor receptor alpha | C3853694 |  |  | 1 |  |
| sortilin 1 | 6272 |  |  | 1 |  |
| miR495 | 574453 |  |  | 1 |  |
| miR543 | 100126335 |  |  | 1 |  |
| miR106a | 406899 |  |  | 1 |  |
| acylCoA synthetase longchain family member 1 | 65985 |  |  | 1 |  |
| repulsive guidance molecule family member B | 285704 |  |  | 1 |  |
| semaphorin | C1136340 |  |  | 1 |  |
| SEMA7A | 8482 |  |  | 1 |  |
| miR382 | 494331 |  | 1 | 1 | 1 |
| NEGR1 | 257194 |  |  | 1 |  |
| PDGFRA | C3853694 |  |  | 1 |  |
| SORT1 | 6272 |  |  | 1 |  |
| CRT | 799 |  | 2 | 1 | 0.5 |
| phosphodiesterase 10A | 10846 |  |  | 1 |  |
| v-ATPase | 242341 |  |  | 1 |  |
| CLN1 | 19063 |  |  | 1 |  |
| PPT1 | 19063 |  |  | 1 |  |
| LSD | 1776 |  |  | 1 |  |
| INCL | 19063 |  |  | 1 |  |
| adaptor protein- | C1135629 | 3 | 3 | 1 | 0.333333333333333 |
| AP-3 | 11774 |  |  | 1 |  |
| surface changes | C0487919 |  |  | 1 |  |
| SIRPA | 140885 |  |  | 1 |  |
| signal regulatory protein alpha | 140885 |  |  | 1 |  |
| Ghrelin Receptor | 2693 |  |  | 1 |  |
| ghrelin receptor | 208188 |  |  | 1 |  |
| Interferon-beta | C0015980 |  |  | 1 |  |
| TRIF | 148022 |  | 1 | 1 | 1 |
| ATG16L1 gene | 55054 |  | 1 | 1 | 1 |
| serotonin transporter | 574140 |  |  | 1 |  |
| DNAJB6 | 23950 |  |  | 1 |  |
| high mobility group box 1 protein | C0019796 |  |  | 1 |  |
| protein EndoA/Endophilin-A | C0038164 | 1 |  | 1 |  |
| p40phox | 4689 |  |  | 1 |  |
| p67phox | 4688 |  |  | 1 |  |
| CD4 | 24932 |  |  | 1 |  |
| BET | 92737 |  |  | 1 |  |
| transposon | C1257902 |  |  | 1 |  |
| GPR3 | 2827 |  |  | 1 |  |
| GPR6 | 2830 |  |  | 1 |  |
| GPR17 | 2840 |  |  | 1 |  |
| GPR55 | 9290 |  |  | 1 |  |
| GPR162 | 27239 | 2 |  | 1 |  |
| B max determination | C0033453 |  |  | 1 |  |
| MCPs | C3540820 |  |  | 1 |  |
| fLG | 2312 |  |  | 1 |  |
| macrophage-inflammatory protein-3alpha | 20297 |  |  | 1 |  |
| CCL20 | 20297 |  |  | 1 |  |
| ARX | 170302 |  |  | 1 |  |
| sphingosine 1-phosphate receptor | C3537343 |  |  | 1 |  |
| inhibitory G-protein | C0086706 | 1 | 1 | 1 | 1 |
| arrestin | C0104230 |  |  | 1 |  |
| S1P1 | 1901 |  |  | 1 |  |
| S1P2 receptor- | 9294 |  |  | 1 |  |
| S1P1 receptor | C0291188 |  |  | 1 |  |
| S1P1 receptor- | C0291188 |  |  | 1 |  |
| ATF6 | 304962 |  |  | 1 |  |
| cholesterol-25-hydroxylase | 9023 |  |  | 1 |  |
| CH25H | 9023 |  |  | 1 |  |
| Delta Opioid Receptor | C0140057 | 1 | 2 | 1 | 0.5 |
| DORs | C0140057 | 1 | 2 | 1 | 0.5 |
| DOR | 58476 |  |  | 1 |  |
| Leu5 | 10206 |  |  | 1 |  |
| DADLE | C0079286 |  |  | 1 |  |
| DADLE's | C0079286 |  |  | 1 |  |
| SP0990 | 931503 |  |  | 1 |  |
| Ass1 | 445 |  |  | 1 |  |
| RyRs | C0054493 |  |  | 1 |  |
| IP3Rs | 3708 |  |  | 1 |  |
| ryanodine receptors | C0054493 |  |  | 1 |  |
| HDx | 139324 |  |  | 1 |  |
| SLC6A15 gene | 55117 |  |  | 1 |  |
| BPRS | 138948 |  |  | 1 |  |
| GRIN2A | 2903 | 1 | 1 | 1 | 1 |
| Rpn10 | 5710 |  |  | 1 |  |
| chemokine receptors | C0524914 | 1 | 1 | 1 | 1 |
| CCR2-GFP | 729230 |  |  | 1 |  |
| 12-CCR2 | 729230 |  |  | 1 |  |
| CCL2-CCR2 | 729230 |  |  | 1 |  |
| heat shock protein 90 kDa | 3326 |  |  | 1 |  |
| reductase gene | C1335186 | 1 |  | 1 |  |
| neurotrophin-3 | 81737 |  |  | 1 |  |
| gene clusters | C0017258 | 1 |  | 1 |  |
| Pgp | 283871 | 3 | 1 | 1 | 1 |
| GAM | C0061057 |  |  | 1 |  |
| ALDH1 | 11668 |  | 1 | 1 | 1 |
| AD8 | 353128 |  | 1 | 1 | 1 |
| Ndufs4 | 4724 | 1 | 1 | 1 | 1 |
| SAC1 | 22908 |  |  | 1 |  |
| synaptojanin | C0385927 |  |  | 1 |  |
| Atg18a | 38913 |  |  | 1 |  |
| PIKfyve | 200576 |  |  | 1 |  |
| GIPR | 2696 |  |  | 1 |  |
| GIPR | 381853 |  |  | 1 |  |
| APPL | 31002 |  |  | 1 |  |
| APPL | 26060 |  |  | 1 |  |
| Amyloid Precursor Protein-Like | 31002 |  |  | 1 |  |
| FST | 10468 |  |  | 1 |  |
| ARNT | 25242 |  |  | 1 |  |
| Arginase 1 | C0003763 |  |  | 1 |  |
| vesicle-associated membrane protein- | C0078208 |  |  | 1 |  |
| bone marrow stromal cell antigen-1 | C0255325 |  |  | 1 |  |
| HSPA9 | 3313 |  | 1 | 1 | 1 |
| hype | 11153 |  |  | 1 |  |
| Alglucosidase alpha | C1695579 |  |  | 1 |  |
| GAA | 2548 | 1 | 1 | 1 | 1 |
| alglucosidase alfa | C1695579 |  |  | 1 |  |
| AA | C1695579 |  |  | 1 |  |
| ATR | 685055 |  |  | 1 |  |
| transcription factor Nuclear Receptor Related Factor 1 | C0214562 |  |  | 1 |  |
| FBXO2 | 26232 |  |  | 1 |  |
| FBXO6 | 26270 |  |  | 1 |  |
| FBXO12 | 285231 |  |  | 1 |  |
| FBXO41 | 150726 |  |  | 1 |  |
| G20-G21 | 51161 |  | 1 | 1 | 1 |
| lag 0.1 | 388372 |  |  | 1 |  |
| lags 1 | 388372 |  |  | 1 |  |
| lags 0.1 | 388372 |  |  | 1 |  |
| thrombin-PAR1 | C0076552 |  |  | 1 |  |
| matrix metalloproteinase (MMP)-9 | C0165519 |  | 1 | 1 | 1 |
| protease- | 100616101 |  |  | 1 |  |
| PAR1 | 100616101 |  |  | 1 |  |
| t(1205 | 1070623 |  |  | 1 |  |
| serotonin 5-HT2 receptors | C0036757 | 1 |  | 1 |  |
| Bcl-2-associated athanogene 5 | 9529 | 1 |  | 1 |  |
| DQA1 | 3117 |  |  | 1 |  |
| amino butyric acid (GABA)a receptor | C0051610 |  | 1 | 1 | 1 |
| GABAa | 14405 | 1 |  | 1 |  |
| Protein tyrosine phosphatase 1B | C0908145 |  |  | 1 |  |
| PTP1B | 19246 |  |  | 1 |  |
| PTP1B | 38160 |  |  | 1 |  |
| alpha-mannosidase | C0051350 |  |  | 1 |  |
| lysosomal alpha-mannosidase | C0283775 |  |  | 1 |  |
| transcription initiation factor | C0030943 |  | 1 | 1 | 1 |
| TIF- | 7301 |  |  | 1 |  |
| RRN3 | 106298 |  |  | 1 |  |
| Ki67 | 17345 |  |  | 1 |  |
| RLS | 192142 |  |  | 1 |  |
| C19orf12 | 83636 |  |  | 1 |  |
| C19orf12 neurodegeneration with brain iron accumulation (NBIA) causing gene | 83636 |  |  | 1 |  |
| Lon protease | 9361 |  | 1 | 1 | 1 |
| Lon | 74142 |  |  | 1 |  |
| alpha-ketoglutarate dehydrogenase | 18293 |  |  | 1 |  |
| SPA | 653509 |  |  | 1 |  |
| Pro3 | 5831 |  |  | 1 |  |
| homeodomain-interacting protein kinase 2 | 28996 |  |  | 1 |  |
| HIPK2 | 28996 |  |  | 1 |  |
| pyruvate carrier | C0072799 |  |  | 1 |  |
| EPO | 13856 |  |  | 1 |  |
| aryl sulfotransferase | 6783 |  |  | 1 |  |
| SULT1A3 | 6818 |  | 1 | 1 | 1 |
| SULT1A4 | 445329 |  |  | 1 |  |
| SULT1A3/4 genes | 6818 |  | 1 | 1 | 1 |
| protein kinase D1 | 18760 |  |  | 1 |  |
| PKD1 | 18763 |  |  | 1 |  |
| SLP-2 | 33608 |  |  | 1 |  |
| glutathione-s-transferase pi | C0537086 |  | 3 | 1 | 0.333333333333333 |
| GST-pi | C0537086 |  | 3 | 1 | 0.333333333333333 |
| IgLON5 | 402665 |  |  | 1 |  |
| immunoglobulin G (IgG) subclass | C1275917 |  |  | 1 |  |
| IgG subclass | C1275917 |  |  | 1 |  |
| IgG1 | C0020855 |  |  | 1 |  |
| multifunctional protein 2 | 3295 |  |  | 1 |  |
| CD133 | 8842 |  |  | 1 |  |
| TLR5 | 289337 |  |  | 1 |  |
| TLR1 | 7096 |  |  | 1 |  |
| intercellular adhesion molecule-1 | C0063695 |  | 1 | 1 | 1 |
| GPNMB | 10457 |  |  | 1 |  |
| KLHL7 | 55975 |  |  | 1 |  |
| NUPL2 | 11097 |  |  | 1 |  |
| KLHL7-AS1 | 100775104 |  |  | 1 |  |
| HY I | 81888 |  |  | 1 |  |
| serum S-100B protein | C0036825 | 1 | 2 | 1 | 0.5 |
| SE=0.058 | 1056906 |  |  | 1 |  |
| histamine H3 receptor | 11255 |  | 1 | 1 | 1 |
| ASCL1 | 429 |  | 2 | 1 | 0.5 |
| STX5 | 6811 |  |  | 1 |  |
| ALiX | 10015 |  |  | 1 |  |
| PDCD6IP | 10015 |  |  | 1 |  |
| Vps4 | 27183 |  |  | 1 |  |
| AAV2-GAD | 2571 | 1 | 1 | 1 | 1 |
| MHC-II genes | C1334545 |  |  | 1 |  |
| C-2 | 717 | 1 | 1 | 1 | 1 |
| sterol regulatory element binding protein | 7555 |  |  | 1 |  |
| sterol regulatory element-binding protein | 7555 |  |  | 1 |  |
| SREBP-2 | 6721 |  |  | 1 |  |
| synaptogyrin-3 | 9143 |  |  | 1 |  |
| PGC-1alpha | 40562 |  | 1 | 1 | 1 |
| AMPK | 43904 |  |  | 1 |  |
| MRP | 4363 |  |  | 1 |  |
| claudin-5 | 7122 |  |  | 1 |  |
| occludin | 100506658 |  |  | 1 |  |
| PECAM-1 | 5175 |  | 1 | 1 | 1 |
| VE-cadherin | 1003 |  |  | 1 |  |
| BTS | 1201 |  |  | 1 |  |
| MC RTs | 4204 |  | 3 | 1 | 0.333333333333333 |
| Abeta29-42 | C1743100 |  |  | 1 |  |
| MicroRNA-130b | 406920 |  |  | 1 |  |
| microRNA-130b | 100314028 |  |  | 1 |  |
| miR-130b | 100314028 |  |  | 1 |  |
| HDAC3 | 84578 |  |  | 1 |  |
| PON-2 | 5445 |  | 1 | 1 | 1 |
| KMOS | 8564 | 1 |  | 1 |  |
| adrenoceptor alpha | C0034784 |  |  | 1 |  |
| ADRA1D | 29413 |  |  | 1 |  |
| CACNA1S | 682930 |  |  | 1 |  |
| NDUFV2 | 81728 |  |  | 1 |  |
| prohibitin | C0084178 |  |  | 1 |  |
| oxytocin receptor | 5021 |  |  | 1 |  |
| OXTR | 25342 |  |  | 1 |  |
| collapsin response mediator protein 1 | 25415 |  |  | 1 |  |
| CRMP1 | 25415 |  |  | 1 |  |
| dihydropyrimidinase like 2 | 25416 |  |  | 1 |  |
| DPYSL2 | 25416 |  |  | 1 |  |
| PHB | C0084178 |  |  | 1 |  |
| BACE1 | 23621 |  | 1 | 1 | 1 |
| beta-site APP cleaving enzyme | 23621 |  | 1 | 1 | 1 |
| APC +2 | 10297 |  |  | 1 |  |
| sharpness | 23013 |  |  | 1 |  |
| human serum albumin | C0304925 |  | 1 | 1 | 1 |
| VAChT | 6572 |  |  | 1 |  |
| CBF.1 | 3516 |  |  | 1 |  |
| caveolin | C0887901 |  |  | 1 |  |
| alpha hemoglobin stabilizing protein | 51327 |  |  | 1 |  |
| solute carrier family 11 member 2 | 4891 | 1 |  | 1 |  |
| ferrochelatase | 2235 | 1 |  | 1 |  |
| EPB42 | 2038 |  |  | 1 |  |
| 5'-aminolevulinate synthase 2 | 212 | 1 |  | 1 |  |
| MCC | 4163 |  |  | 1 |  |
| interleukin-13 receptor alpha 1 | 16164 |  | 1 | 1 | 1 |
| cytokine receptor | C0206552 |  |  | 1 |  |
| JWA | 10550 |  |  | 1 |  |
| ARL6IP5 | 65106 |  |  | 1 |  |
| bacterial genomes | C0085238 |  |  | 1 |  |
| Smad3 | 25631 |  |  | 1 |  |
| Catechol-O-methyltransferase | 12846 |  | 1 | 1 | 1 |
| proliferating cell nuclear antigen | 5111 |  |  | 1 |  |
| PCNA | 5111 |  |  | 1 |  |
| NBS1 | 4683 | 1 |  | 1 |  |
| DNA polymerase eta | C0675096 |  |  | 1 |  |
| alpha-1 | C1979844 | 1 | 4 | 1 | 0.25 |
| CISD2 | 493856 |  |  | 1 |  |
| ALMS1 | 7840 |  |  | 1 |  |
| SLC19A2 | 10560 |  |  | 1 |  |
| WFS1 gene | 7466 |  |  | 1 |  |
| Msx1 genes | 4487 | 2 | 1 | 1 | 1 |
| IKBKAP | 230233 |  |  | 1 |  |
| ELP1 | 230233 |  |  | 1 |  |
| IKAP | 230233 |  |  | 1 |  |
| MALAT1 | 72289 |  |  | 1 |  |
| DMPK | 1760 |  |  | 1 |  |
| CR 16 | 644150 |  |  | 1 |  |
| GPR139 | 124274 |  |  | 1 |  |
| Trp | C0041249 |  |  | 1 |  |
| dos | 255057 |  |  | 1 |  |
| HOTAIR | 100124700 |  |  | 1 |  |
| Hox transcript antisense intergenic RNA | 100124700 |  |  | 1 |  |
| HOXC | 3220 |  |  | 1 |  |
| HOTAIR | 100503872 |  |  | 1 |  |
| Ang II | 24179 | 2 | 2 | 1 | 0.5 |
| AT1R | 24180 |  |  | 1 |  |
| eotaxin | 20292 |  |  | 1 |  |
| dipeptidyl peptidase 4 | 1803 |  |  | 1 |  |
| rpn1 | 177455 |  |  | 1 |  |
| egl-1 | 179943 |  |  | 1 |  |
| PAP | 5068 |  |  | 1 |  |
| Rab27a | 5873 |  |  | 1 |  |
| CD68 | 12514 |  |  | 1 |  |
| MetSO | C0066124 | 1 |  | 1 |  |
| Msr | 4552 |  |  | 1 |  |
| Msrs | C2713548 |  | 1 | 1 | 1 |
| ST) at 2 | 27111 |  |  | 1 |  |
| HCD | 1732 |  |  | 1 |  |
| DJ-1 | 43652 |  | 1 | 1 | 1 |
| IL-7 | 3574 |  |  | 1 |  |
| IL-7 receptor | C0083032 |  |  | 1 |  |
| IL-7R | 3575 |  |  | 1 |  |
| interleukin (IL)-17 | C0384648 |  |  | 1 |  |
| bone morphogenetic protein receptor type IB | 658 |  |  | 1 |  |
| Growth/differentiation factor-5 | 252835 |  | 2 | 1 | 0.5 |
| GDF-5 | 252835 |  | 2 | 1 | 0.5 |
| GDF-5 | 14563 |  |  | 1 |  |
| BMPRIB-CA | 658 |  |  | 1 |  |
| terminal deoxynucleotidyl transferase | C0012881 |  |  | 1 |  |
| TdT | 21673 | 1 |  | 1 |  |
| BMPRIB | 12167 |  |  | 1 |  |
| Humanin | C0966511 |  |  | 1 |  |
| HN | C0966511 |  |  | 1 |  |
| OGG1 | 4968 |  | 1 | 1 | 1 |
| neurotrophin receptor | C0132173 | 1 |  | 1 |  |
| neurotrophin receptor's | C0132173 | 1 |  | 1 |  |
| GHRP-6 | C0120348 |  |  | 1 |  |
| AR | 28882 | 1 | 1 | 1 | 1 |
| PAMP | 10730 |  |  | 1 |  |
| c-Abl | 11350 |  |  | 1 |  |
| high Fe( | 3077 | 3 | 2 | 1 | 0.5 |
| TLX | 7101 |  |  | 1 |  |
| EDA | 1896 |  |  | 1 |  |
| hsa-miR-148a | 406940 |  |  | 1 |  |
| Roralpha | 6095 |  |  | 1 |  |
| synaptophysin | 24804 |  |  | 1 |  |
| calcyon | C0912815 |  |  | 1 |  |
| LPS- | 3664 |  |  | 1 |  |
| MCM | 4594 | 1 | 1 | 1 | 1 |
| Nramp1 | 6556 |  | 1 | 1 | 1 |
| natural resistance-associated macrophage protein-1 | 6556 |  | 1 | 1 | 1 |
| Nramp1 | 18173 |  |  | 1 |  |
| inflammasomes | C2936529 |  |  | 1 |  |
| IL18 | 3606 |  |  | 1 |  |
| LTF | 4057 |  |  | 1 |  |
| PCD | 352909 |  |  | 1 |  |
| mal | C3540595 |  |  | 1 |  |
| Vps35 | 37536 |  |  | 1 |  |
| dVps35 | 37536 |  |  | 1 |  |
| Rab5 | 33418 |  |  | 1 |  |
| Rab11 | 42501 |  |  | 1 |  |
| NT3 | 4877 |  |  | 1 |  |
| alpha-Syn toxicity | C0031669 |  |  | 1 |  |
| G protein-coupled receptor kinase | C0872043 | 1 | 1 | 1 | 1 |
| GRK) | C0872383 | 1 | 1 | 1 | 1 |
| GRK5 | 14773 |  |  | 1 |  |
| GRK2 | 110355 |  |  | 1 |  |
| miR-135b | 442891 |  |  | 1 |  |
| rho-associated protein kinase 2 | C1740231 |  |  | 1 |  |
| PDE10A | 10846 |  |  | 1 |  |
| PSD95 | 13385 |  |  | 1 |  |
| PSD-95 | 13385 |  |  | 1 |  |
| ADSL | 158 |  |  | 1 |  |
| Adenylosuccinate Lyase | 158 |  |  | 1 |  |
| c.1387 | 1035088 |  |  | 1 |  |
| TYR | C0012524 | 2 | 1 | 1 | 1 |
| dopamine D4 receptor | 1815 |  | 1 | 1 | 1 |
| TrkA | 59109 |  |  | 1 |  |
| HBA | 6326 |  |  | 1 |  |
| v-SNARE | 10490 |  |  | 1 |  |
| vesicle SNARE | C1506158 |  |  | 1 |  |
| VAMP2 | 6844 |  |  | 1 |  |
| t-SNARE | C1506159 |  |  | 1 |  |
| synapsin I | 6853 |  |  | 1 |  |
| VAC14 genes | 55697 |  |  | 1 |  |
| TPH | 24848 |  |  | 1 |  |
| FGF18 | 29369 |  |  | 1 |  |
| fibroblast growth factor 18 | 29369 |  |  | 1 |  |
| Atg8 | 852200 |  |  | 1 |  |
| von Willebrand factor | C0042971 |  |  | 1 |  |
| immobilized proteins | C2350558 |  |  | 1 |  |
| FUS | 317385 |  |  | 1 |  |
| Npas2 | 316351 |  |  | 1 |  |
| Cry1 | 299691 |  |  | 1 |  |
| Per1 | 287422 |  |  | 1 |  |
| Rev-Erbalpha | 252917 |  |  | 1 |  |
| Nr1d1 | 252917 |  |  | 1 |  |
| pannexin 1 | 24145 |  |  | 1 |  |
| P2 purinergic receptors | C0206495 |  |  | 1 |  |
| ecto-ATPase | 51592 |  |  | 1 |  |
| EFCAB5 | 374786 |  |  | 1 |  |
| MARK1 | 4139 |  |  | 1 |  |
| Kv3.4 | 3749 |  |  | 1 |  |
| PARP4 | 143 |  |  | 1 |  |
| MTCL1 | 23255 |  |  | 1 |  |
| TRPM7 | 58800 |  |  | 1 |  |
| myeloid differentiation factor-88 | C0286648 |  |  | 1 |  |
| tumor necrosis factor receptor-associated factor 6 | C0530129 |  |  | 1 |  |
| transforming growth factor-beta-activated protein kinase 1 | C0379816 |  | 1 | 1 | 1 |
| GP78 | 267 |  |  | 1 |  |
| Dynamin-related protein 1 | 33445 | 3 | 1 | 1 | 1 |
| Elav | 31000 |  |  | 1 |  |
| hypoxia-inducible factor | 43580 |  |  | 1 |  |
| MAPK | C0018284 | 4 | 3 | 1 | 0.333333333333333 |
| TMT- | 25823 |  |  | 1 |  |
| neurotrophin-4/5 | 4909 |  |  | 1 |  |
| Zip14 | 213053 |  |  | 1 |  |
| Slc39a14 | 213053 |  |  | 1 |  |
| ZIP14 | 23516 |  |  | 1 |  |
| zinc transporter | C1608304 |  |  | 1 |  |
| ZIP | 1613 |  |  | 1 |  |
| Hen Egg White Lysozyme | C0964782 |  |  | 1 |  |
| HEWL | C0964782 |  |  | 1 |  |
| 70kd | 6200 |  |  | 1 |  |
| immediate early gene | C0206256 |  |  | 1 |  |
| sPLA2 | C2756983 | 1 |  | 1 |  |
| less | C1414864 |  |  | 1 |  |
| VRV-PLA2 | 5319 |  |  | 1 |  |
| NN-PLA2 | 5319 |  |  | 1 |  |
| PFAS | 5198 |  |  | 1 |  |
| Dicer | 23405 |  |  | 1 |  |
| RNAse Dicer | 23405 |  |  | 1 |  |
| recombinase | C0073020 |  |  | 1 |  |
| miR-124-3p | 100314155 |  |  | 1 |  |
| organic cation transporter 3 | 20519 |  |  | 1 |  |
| 3-Oct | 20519 |  |  | 1 |  |
| ASIF | C0051134 |  |  | 1 |  |
| Orai1 | 84876 |  |  | 1 |  |
| par | C1705885 |  |  | 1 |  |
| RCAN1 | 1827 |  |  | 1 |  |
| RCAN1-1S | 1827 |  |  | 1 |  |
| IRAK1-TRAF6 | 3654 |  | 2 | 1 | 0.5 |
| mouse myelin basic protein | C1450293 |  |  | 1 |  |
| PKA-mitochondrial scaffold dual-specificity A Kinase Anchoring Protein 1 ( | 8165 |  |  | 1 |  |
| D-AKAP1 | 8165 |  |  | 1 |  |
| RIIbeta | 5577 |  |  | 1 |  |
| Miro-2 | 89941 |  | 2 | 1 | 0.5 |
| TRAK2 | 66008 |  |  | 1 |  |
| Geminin | 57441 |  |  | 1 |  |
| Geminin's | 51053 |  |  | 1 |  |
| PDHA1 | 5160 |  |  | 1 |  |
| pyruvate dehydrogenase (PDH) complex | C0034344 |  |  | 1 |  |
| PDH complex | C0034344 |  |  | 1 |  |
| Bcl-2-associated X protein | C0219474 |  |  | 1 |  |
| r=0.487 | C0220485 |  | 1 | 1 | 1 |
| PKAN | C0070043 |  |  | 1 |  |
| PICALM Gene | 8301 |  | 2 | 1 | 0.5 |
| overlapping genetic | C0017359 |  | 1 | 1 | 1 |
| CR1 | 1378 |  |  | 1 |  |
| CYS C | 25307 |  | 1 | 1 | 1 |
| Cst3 | 25307 |  | 1 | 1 | 1 |
| CYS C | 13010 |  |  | 1 |  |
| PKC-alpha | 24680 |  | 1 | 1 | 1 |
| VDAC1 | 7416 |  |  | 1 |  |
| 14-3-3/phospho-Tau | 10971 | 1 | 1 | 1 | 1 |
| 14-3-3/pTau | 10971 | 1 | 1 | 1 | 1 |
| Microtubule actin crosslinking factor 1 | 23499 |  |  | 1 |  |
| human MACF1 | 23499 |  |  | 1 |  |
| PDCN | 170484 |  |  | 1 |  |
| Microtubule-actin crosslinking factor 1 | 23499 |  |  | 1 |  |
| ACF7 | 23499 |  |  | 1 |  |
| eukaryotic elongation factor 1A-2 | 13628 |  |  | 1 |  |
| thioredoxin-1 | C1956394 |  | 1 | 1 | 1 |
| direct thrombin inhibitor | C0003440 |  |  | 1 |  |
| Nurr1-controlled genes | C1335077 |  | 1 | 1 | 1 |
| ACT-R | 8202 |  |  | 1 |  |
| TRIM10 | 10107 |  |  | 1 |  |
| SETD1A | 9739 |  |  | 1 |  |
| WNT3 | 7473 |  |  | 1 |  |
| KANSL1 | 284058 | 1 |  | 1 |  |
| BOLA2 | 552900 |  |  | 1 |  |
| GUCY1A3 | 2982 |  |  | 1 |  |
| Beta-secretase 1 | 23621 |  | 1 | 1 | 1 |
| MICS1 | 27069 |  |  | 1 |  |
| AR | 10894 |  |  | 1 |  |
| hAR | 10894 |  |  | 1 |  |
| MYB | 4602 |  |  | 1 |  |
| Cav1.2 | 775 |  | 1 | 1 | 1 |
| HT7 | 12215 |  |  | 1 |  |
| DHCR7 | 1717 |  |  | 1 |  |
| CYP2R1 | 120227 |  |  | 1 |  |
| CYP24A1 | 1591 |  |  | 1 |  |
| Rab-interacting lysosomal protein | 83547 |  |  | 1 |  |
| RILP | 83547 |  |  | 1 |  |
| BPI | 671 |  |  | 1 |  |
| MGMT | 4255 |  |  | 1 |  |
| Nogo-A | 83765 |  |  | 1 |  |
| TRN | 3842 |  |  | 1 |  |
| Pdxk | 39066 |  |  | 1 |  |
| ribosomal protein S6 | 29304 |  |  | 1 |  |
| GLT-1 | 20511 |  |  | 1 |  |
| glutamate aspartate transporter | 20512 |  |  | 1 |  |
| GLAST | 20512 |  |  | 1 |  |
| ATF2 | 1386 |  |  | 1 |  |
| HAX-1 | 291202 |  |  | 1 |  |
| PGC-1alpha gene | 5225 |  |  | 1 |  |
| estrogen-related receptor alpha | 2101 |  | 1 | 1 | 1 |
| NRF-2 | 55922 |  |  | 1 |  |
| beta-methylamino-L-alanine | C0124981 | 2 | 1 | 1 | 1 |
| G41 | 55012 |  |  | 1 |  |
| STC-1 | 20855 |  |  | 1 |  |
| c-REL | 5966 |  |  | 1 |  |
| BIRC3 | 330 |  |  | 1 |  |
| epoxomicin | C0656383 |  |  | 1 |  |
| Amino Acid Supplementation | C0556082 |  |  | 1 |  |
| aryl hydrocarbon receptor | 11622 |  |  | 1 |  |
| N-phosphono-methylglycine | C0036228 |  |  | 1 |  |
| miRNA-19B1 | 406980 |  |  | 1 |  |
| miRNA-29A | 407021 |  |  | 1 |  |
| miRNA-15A | 406948 |  |  | 1 |  |
| TDP-43 | 37781 |  |  | 1 |  |
| dynactin | 39536 |  |  | 1 |  |
| p150Glued | 39536 |  |  | 1 |  |
| TBPH | 37781 |  |  | 1 |  |
| CPZ | 8532 |  |  | 1 |  |
| LPLI | 10434 |  |  | 1 |  |
| Gpx4 | 625249 |  |  | 1 |  |
| AgRP | 11604 |  |  | 1 |  |
| POMC | 18976 |  |  | 1 |  |
| nuclear receptor subfamily 4 group A member 2 | C2720177 |  |  | 1 |  |
| Hydroxytryptophan | C0000578 |  | 2 | 1 | 0.5 |
| CST | 106478911 |  |  | 1 |  |
| REC | 84515 |  |  | 1 |  |
| DNA polymerase-beta | C0525039 |  |  | 1 |  |
| pol- | 100616496 | 1 | 1 | 1 | 1 |
| DNA polymerase-beta inhibitor 2 | C0971133 |  |  | 1 |  |
| metabotropic glutamate receptor 5 antagonist 2-methyl-6 | C0756239 |  |  | 1 |  |
| TMP | 25314 |  |  | 1 |  |
| PDGF-beta | 18591 |  |  | 1 |  |
| small Rab GTPases | C0751984 | 2 | 3 | 1 | 0.333333333333333 |
| Rab7 | 19349 |  |  | 1 |  |
| Spinophilin | 84687 |  | 1 | 1 | 1 |
| protein phosphatase 1 | C1956003 | 1 |  | 1 |  |
| PP1 | C1956003 | 1 |  | 1 |  |
| ZFP42 | 100405209 |  |  | 1 |  |
| PODXL | 100414785 |  |  | 1 |  |
| DNMT3B | 100412936 |  |  | 1 |  |
| C-MYC | 100407754 |  |  | 1 |  |
| LIN28 | 79727 |  |  | 1 |  |
| KLF4 | 100407017 |  |  | 1 |  |
| NANOG | 100399586 |  |  | 1 |  |
| SOX2 | 100407856 |  |  | 1 |  |
| 4-Oct | 100384946 |  |  | 1 |  |
| FOXA2 | 100390359 |  |  | 1 |  |
| OTX2 | 100413469 |  |  | 1 |  |
| EN-1 | 100405008 |  |  | 1 |  |
| Apoptosis Inhibitors | C1332320 |  |  | 1 |  |
| C17-CO2H | 54360 |  |  | 1 |  |
| C-17 | 54360 |  |  | 1 |  |
| GSK3 | 173149 |  |  | 1 |  |
| FBXL5 | 26234 |  |  | 1 |  |
| Iron regulatory proteins | C0378503 |  | 1 | 1 | 1 |
| leucine-rich repeat protein | C0664702 |  |  | 1 |  |
| TM3 | 7170 |  | 2 | 1 | 0.5 |
| cyclooxygenases | C0033551 | 3 | 3 | 1 | 0.333333333333333 |
| Huntington's disease gene | C0872189 | 1 | 1 | 1 | 1 |
| MSH3 | 4437 |  | 1 | 1 | 1 |
| DHFR | 1719 |  | 1 | 1 | 1 |
| MTRNR2L2 | 100462981 |  |  | 1 |  |
| 10-4DHFR p=8.45 x 10-4MTRNR2L2 | 100462981 |  |  | 1 |  |
| Msh3 | 17686 |  |  | 1 |  |
| N22-P40 | 3578 |  |  | 1 |  |
| OR 2.52 | 26479 |  |  | 1 |  |
| REST | 5978 |  |  | 1 |  |
| paraoxonase-1 | 84024 |  |  | 1 |  |
| PON-1 | 84024 |  |  | 1 |  |
| BChE | 65036 |  |  | 1 |  |
| CADPS2 | 93664 |  |  | 1 |  |
| Ca2+-dependent activator protein for secretion 2 | 93664 |  |  | 1 |  |
| CADPS | 8618 |  |  | 1 |  |
| CADPS2 gene | 93664 |  |  | 1 |  |
| luciferase-based gene | C1334435 |  |  | 1 |  |
| PLK-2 | 20620 |  | 1 | 1 | 1 |
| solanezumab | C2935150 |  |  | 1 |  |
| sargramostim | C0216231 |  |  | 1 |  |
| CD16 | 2214 |  |  | 1 |  |
| phosphoglycerate kinase | C0031656 |  |  | 1 |  |
| lipid/lipoprotein | C0443602 |  |  | 1 |  |
| G/H/S | C0086903 | 1 |  | 1 |  |
| arrhythmogenic right ventricular dysplasia 3 | 424 |  |  | 1 |  |
| NGF receptors | C0132173 | 1 |  | 1 |  |
| SENS | 284252 |  |  | 1 |  |
| MMP12 | 4321 |  |  | 1 |  |
| MMP12 gene | 4321 |  |  | 1 |  |
| class III beta tubulin | C3537404 |  |  | 1 |  |
| developmental origin | C0017340 | 1 | 2 | 1 | 0.5 |
| CGRP | C0006669 |  |  | 1 |  |
| entire X-chromosome | C0450238 |  |  | 1 |  |
| entire X chromosome | C0450238 |  |  | 1 |  |
| Smad4 | 17128 |  |  | 1 |  |
| activin | C0050668 |  |  | 1 |  |
| ACMSD Gene | 130013 |  |  | 1 |  |
| ACMSD | 130013 |  |  | 1 |  |
| endopeptidase | C0030946 |  |  | 1 |  |
| FAC | 2176 |  |  | 1 |  |
| GSTA4 | 300850 |  |  | 1 |  |
| Glutathione S-transferase alpha 4 | 300850 |  |  | 1 |  |
| protein a | C0038164 | 1 |  | 1 |  |
| single-domain antibodies | C3494231 |  |  | 1 |  |
| vascular cell adhesion protein 1 | C0078056 |  |  | 1 |  |
| EPAS1 | 2034 |  |  | 1 |  |
| ADAM9 | 8754 |  |  | 1 |  |
| EGLN1 | 54583 |  |  | 1 |  |
| C-type Natriuretic Peptide | 4880 |  |  | 1 |  |
| PLP-alpha | 19110 |  |  | 1 |  |
| SNT | 10818 |  |  | 1 |  |
| SNT-V | 10818 |  |  | 1 |  |
| SAMe | C0036002 | 5 | 2 | 1 | 0.5 |
| s-adenosyl methionine | C0036002 | 5 | 2 | 1 | 0.5 |
| protein kinase inhibitors | C1449702 |  |  | 1 |  |
| meanon | C0650705 |  |  | 1 |  |
| Pax 6 | 5080 |  |  | 1 |  |
| immunoglobulin-heavy-chain-binding protein | C0063423 |  |  | 1 |  |
| Leu | C0064819 |  |  | 1 |  |
| H21-H6 | 9495 |  |  | 1 |  |
| SAR | 20223 |  |  | 1 |  |
| JMP | 5696 |  |  | 1 |  |
| SLC30A10 | 55532 |  |  | 1 |  |
| SLC30A10 | 289353 |  |  | 1 |  |
| PERK | 5313 |  |  | 1 |  |
| miR-433 | 574034 | 1 | 2 | 1 | 0.5 |
| miR-34b | 407041 |  |  | 1 |  |
| miR-34c | 407042 |  |  | 1 |  |
| ChAc | 23230 |  |  | 1 |  |
| VPS13A | 23230 |  |  | 1 |  |
| TRPC6 | 7225 |  |  | 1 |  |
| DVL1-3 | 1855 |  |  | 1 |  |
| LRRK2 | 445832 |  |  | 1 |  |
| PDZ domain-containing protein | C0872241 |  |  | 1 |  |
| GIPC1 | 67903 |  |  | 1 |  |
| ILK | 16202 |  |  | 1 |  |
| PRICKLE1 | 144165 |  |  | 1 |  |
| CELSR1 | 9620 |  |  | 1 |  |
| FLOTILLIN-2 | 2319 |  |  | 1 |  |
| CULLIN-3 | 8452 |  |  | 1 |  |
| Parkinson disease protein 7 | C3538910 |  |  | 1 |  |
| Two-Pore Channel 2 | 219931 |  |  | 1 |  |
| TPC2 | 219931 |  |  | 1 |  |
| Angiopoietin-1 | 284 |  |  | 1 |  |
| anti-acetylcholine receptor (AChR) antibody | C0236516 |  |  | 1 |  |
| Secretory carrier membrane protein 5 | 192683 |  |  | 1 |  |
| SCAMP5 | 192683 |  |  | 1 |  |
| secretory carrier membrane protein | C0597427 | 1 | 2 | 1 | 0.5 |
| orexin-A | 25723 |  |  | 1 |  |
| phosphodiesterase 4 | C0070829 | 1 |  | 1 |  |
| p = 0.748 | C0391093 |  |  | 1 |  |
| semax | C0141950 |  |  | 1 |  |
| selank | C1100112 |  |  | 1 |  |
| taftsin | C0041350 |  |  | 1 |  |
| E3s | 7805 |  |  | 1 |  |
| miR-29a | 407021 |  |  | 1 |  |
| miR-29c | 407026 |  |  | 1 |  |
| Cav2.1 | 858 |  |  | 1 |  |
| Talk | 659 | 1 |  | 1 |  |
| LAPTM4A | 9741 |  |  | 1 |  |
| ATP6V0D1 | 9114 |  |  | 1 |  |
| protease cathepsin D | C0072115 |  |  | 1 |  |
| Pep4 | 855949 |  |  | 1 |  |
| Pep1 | 852264 |  |  | 1 |  |
| oPD) | 2316 |  |  | 1 |  |
| oPD | 2316 |  |  | 1 |  |
| Ronin | 57215 |  |  | 1 |  |
| Thap11 | 57215 |  |  | 1 |  |
| Gtf2h4 | 2968 | 1 |  | 1 |  |
| Tyrosine hydroxylase | 38746 |  |  | 1 |  |
| Dopamine transporter | 36849 |  |  | 1 |  |
| plasma membrane protein | C1179841 |  |  | 1 |  |
| Prostaglandin EP2 Receptors | C0034835 |  |  | 1 |  |
| E2 receptor 2 | 6925 |  | 2 | 1 | 0.5 |
| HCP1 | C1612271 |  |  | 1 |  |
| heme carrier protein 1 | 303333 |  |  | 1 |  |
| Kruppel-like factor 4 | C0529085 |  |  | 1 |  |
| KLF4 | 114505 |  |  | 1 |  |
| secondary origins | C1708995 | 1 |  | 1 |  |
| protein-fragment | C1335533 |  | 1 | 1 | 1 |
| GGA3 | 23163 |  |  | 1 |  |
| HV1 | 84329 |  |  | 1 |  |
| O-GlcNAcase | 154968 |  |  | 1 |  |
| OGA | 154968 |  |  | 1 |  |
| Midnolin | 90007 |  |  | 1 |  |
| MIDN | 90007 |  |  | 1 |  |
| MIDN | 314623 |  |  | 1 |  |
| cAMP response element | C1511573 |  |  | 1 |  |
| Noxa | C1334155 |  |  | 1 |  |
| lactoferrin | C0022942 |  |  | 1 |  |
| CAR' | 846 |  |  | 1 |  |
| CAR-25 | 846 |  |  | 1 |  |
| CAR | 846 |  |  | 1 |  |
| mul-1 | 298576 |  |  | 1 |  |
| PPL | 5493 |  |  | 1 |  |
| lipase | C0023764 |  |  | 1 |  |
| NLRP1 | 22861 |  |  | 1 |  |
| microtubule-associated protein 2 | 4133 | 3 | 1 | 1 | 1 |
| BK channels | 3827 |  |  | 1 |  |
| IL-18 | 3606 |  |  | 1 |  |
| BK channel | 3827 |  |  | 1 |  |
| BK protein | 51760 |  |  | 1 |  |
| iberiotoxin | C0082965 |  |  | 1 |  |
| MIP-1alpha | C0128546 |  |  | 1 |  |
| LAMP2A | 16784 |  | 1 | 1 | 1 |
| 1-Dec | 50514 |  |  | 1 |  |
| 1-Dec | 20893 |  |  | 1 |  |
| PIK3CA | 18706 |  |  | 1 |  |
| aquaporin-4 | 11829 | 1 |  | 1 |  |
| Mhc2ta | 4261 |  |  | 1 |  |
| Mhc2ta gene | 4261 |  |  | 1 |  |
| Mhc2ta | 85483 |  |  | 1 |  |
| Sult | C1328027 |  |  | 1 |  |
| Brd2 | 14312 |  |  | 1 |  |
| OPRM1 | 4988 |  | 2 | 1 | 0.5 |
| LMX1B | 16917 |  |  | 1 |  |
| reelin | 5649 |  |  | 1 |  |
| NUCKS1 | 64710 |  |  | 1 |  |
| fatty acid binding protein 3 | 2170 | 1 |  | 1 |  |
| FABP3 | 2170 | 1 |  | 1 |  |
| AMPK | 78975 |  |  | 1 |  |
| apyrase | C0003652 |  |  | 1 |  |
| retinoid X receptor | C0140283 |  | 1 | 1 | 1 |
| IL-10 receptor | C0247771 |  |  | 1 |  |
| IL-10R gene | C1334205 |  |  | 1 |  |
| Thioredoxin-interacting protein | 10628 | 1 |  | 1 |  |
| Thioredoxin-interacting protein | C1450344 |  |  | 1 |  |
| mTORC | C3152110 |  |  | 1 |  |
| Cxcl13 | 55985 |  |  | 1 |  |
| pnp | 4860 |  |  | 1 |  |
| IL-23 | 51561 |  |  | 1 |  |
| LXRalpha | 22259 |  |  | 1 |  |
| ABCA1 | 11303 |  |  | 1 |  |
| GLA | 11605 |  |  | 1 |  |
| cystathionine-beta-synthase | 24250 |  |  | 1 |  |
| IPL | 7262 |  |  | 1 |  |
| 3phosphoinositide dependent protein kinase 1 | 5170 |  |  | 1 |  |
| PDPK1 | 5170 |  |  | 1 |  |
| PRKACB | 5567 |  |  | 1 |  |
| Seipin | 26580 |  |  | 1 |  |
| Srsf10 | 10772 |  |  | 1 |  |
| Creb3 | 10488 |  |  | 1 |  |
| Ifit3 | 3437 |  |  | 1 |  |
| Rsad2 | 91543 |  |  | 1 |  |
| MAVS | 57506 |  |  | 1 |  |
| DDX58 | 23586 |  |  | 1 |  |
| IFIT1 | 3434 |  |  | 1 |  |
| Fc gamma receptor III | 14131 |  |  | 1 |  |
| Fcgr3 | 14131 |  |  | 1 |  |
| CD16 | 14131 |  |  | 1 |  |
| NfH | 4744 |  |  | 1 |  |
| enzyme genes | C1333402 | 1 |  | 1 |  |
| MicroRNA-181c | 406957 |  | 1 | 1 | 1 |
| MiR-181c | 406957 |  | 1 | 1 | 1 |
| mir181c | 100314242 |  |  | 1 |  |
| miR-181c | 100314242 |  |  | 1 |  |
| BCL2L11 | 64547 |  |  | 1 |  |
| CBS | C0010641 |  |  | 1 |  |
| cystathionine beta-synthase | 12411 |  |  | 1 |  |
| Bydureon | C3257792 |  |  | 1 |  |
| TLR3 | 364594 |  |  | 1 |  |
| TLR3 receptors | 7098 |  |  | 1 |  |
| CHIP | 37837 |  |  | 1 |  |
| carnitine palmitoyltransferase 1 | C0007262 |  |  | 1 |  |
| LAD | 1738 | 1 |  | 1 |  |
| ERR | 6541 |  |  | 1 |  |
| Chrna4 | 11438 | 1 |  | 1 |  |
| Chrna6 genes | 8973 |  | 1 | 1 | 1 |
| apelin | 8862 |  |  | 1 |  |
| apelin receptor | 187 |  |  | 1 |  |
| potassium-chloride cotransporter-2 | 57468 |  |  | 1 |  |
| brain-type creatine kinase | 1152 | 1 |  | 1 |  |
| Atg12 | 39383 |  |  | 1 |  |
| Atg17 | 40700 |  |  | 1 |  |
| MicroRNA-4639 | 100616269 |  |  | 1 |  |
| gene regulators | C0017362 |  | 2 | 1 | 0.5 |
| hsa-miR-4639 | 100616269 |  |  | 1 |  |
| Growth Differentiation Factor 15 | 9518 |  |  | 1 |  |
| GDF15 | 9518 |  |  | 1 |  |
| MMP-9 | 81687 |  |  | 1 |  |
| occludin | 83497 |  |  | 1 |  |
| claudin-5 | 65131 |  |  | 1 |  |
| ZO-1 | 292994 |  |  | 1 |  |
| eIF2alpha | 1965 |  |  | 1 |  |
| glia maturation factor | C0061308 |  |  | 1 |  |
| L4-L5 | 6124 |  |  | 1 |  |
| CD19 | 930 |  |  | 1 |  |
| alpha-klotho protein | C0667394 |  |  | 1 |  |
| DCDC2 | 51473 |  |  | 1 |  |
| PHOX2B | 8929 |  |  | 1 |  |
| MYH8 | 4626 |  |  | 1 |  |
| MYH13 | 8735 |  |  | 1 |  |
| CDH4 | 1002 |  |  | 1 |  |
| ezrin | 7430 |  |  | 1 |  |
| radixin | 5962 |  |  | 1 |  |
| synaptopodin | 11346 |  |  | 1 |  |
| SYNPO | 11346 |  |  | 1 |  |
| Helicobacter pylori (Hp) antibody | C0369303 |  |  | 1 |  |
| flagellin | C0016194 |  |  | 1 |  |
| p54 | 1656 |  |  | 1 |  |
| p29 | 5657 | 1 |  | 1 |  |
| p67 | 10988 |  |  | 1 |  |
| FSH | 6046 | 1 |  | 1 |  |
| p120 | 1500 |  |  | 1 |  |
| p26 | 23423 |  |  | 1 |  |
| p17 | 54107 |  |  | 1 |  |
| ab | C0003241 | 1 | 1 | 1 | 1 |
| mixed lineage kinase 1 | 4293 |  |  | 1 |  |
| MLK1 | 4293 |  |  | 1 |  |
| QM protein | 6134 |  |  | 1 |  |
| GIT | 5034 | 2 | 1 | 1 | 1 |
| P85 | 5295 |  |  | 1 |  |
| MRP2 | C0525410 |  |  | 1 |  |
| multidrug resistance-associated protein 2 | C0525410 |  |  | 1 |  |
| cellular stress response | 51435 |  |  | 1 |  |
| WT-PINK1 | C2362085 |  |  | 1 |  |
| GPR55 | 227326 |  |  | 1 |  |
| myocyte enhancer factor 2D | 81518 |  |  | 1 |  |
| MEF2D | 81518 |  |  | 1 |  |
| heat shock factor (HSF)-1 | 3297 | 3 |  | 1 |  |
| HSF-1 | 79245 |  |  | 1 |  |
| RP-18 | 388552 |  |  | 1 |  |
| 168p | 28462 |  |  | 1 |  |
| PLD1 | 5337 |  |  | 1 |  |
| PLD2 | 5338 |  |  | 1 |  |
| slug | 6591 |  |  | 1 |  |
| LRRK1's | 79705 | 3 | 1 | 1 | 1 |
| Met-1 | 3004 | 1 |  | 1 |  |
| neutrophil gelatinase-associated lipocalin | 3934 | 1 | 2 | 1 | 0.5 |
| NGAL | 3934 | 1 | 2 | 1 | 0.5 |
| ULK1 | 360827 |  |  | 1 |  |
| apoptosis signal-regulating kinase 1 | 365057 |  |  | 1 |  |
| ASK1 | 365057 |  |  | 1 |  |
| PDIA1 | 5034 | 2 | 1 | 1 | 1 |
| PDIA3 | 29468 | 1 |  | 1 |  |
| human insulin | C0795635 |  |  | 1 |  |
| HI | C0795635 |  |  | 1 |  |
| DC50 | 81892 |  |  | 1 |  |
| ATF4 | C0101379 |  |  | 1 |  |
| peroxiredoxin1 | 5052 |  | 2 | 1 | 0.5 |
| Prx1 | 5052 |  | 2 | 1 | 0.5 |
| Rabphilin 3A | 22895 |  |  | 1 |  |
| Rabphilin 3A | 171039 |  |  | 1 |  |
| Rph3A | 171039 |  |  | 1 |  |
| MAGL | 23945 |  |  | 1 |  |
| monoacylglycerol lipase | 23945 |  |  | 1 |  |
| Mgll | 23945 |  |  | 1 |  |
| SP1037 | 931551 |  |  | 1 |  |
| PON2 | 296851 |  |  | 1 |  |
| G Protein-coupled Receptor 17 | 2840 |  |  | 1 |  |
| NADP+-dependent isocitrate dehydrogenase | C0027312 |  |  | 1 |  |
| IDH | 44291 |  |  | 1 |  |
| Granulins | C0168913 |  |  | 1 |  |
| GRNs | C0168913 |  |  | 1 |  |
| glutamate aspartate transporter | C0210502 |  |  | 1 |  |
| angiotensins | C0003018 | 1 |  | 1 |  |
| Glucose-6-phosphatase-alpha | 2538 |  |  | 1 |  |
| glucose-6-phosphatase | C0017755 |  |  | 1 |  |
| G6Pase | 2538 |  |  | 1 |  |
| G6PC | 2538 |  |  | 1 |  |
| Protein Phosphatase 2A Catalytic Subunit Activity in Cell Free Assays. alpha- | 5515 |  |  | 1 |  |
| PP2Ac | 5515 |  |  | 1 |  |
| laminin-511 | C0767106 |  |  | 1 |  |
| extracellular matrix protein | C0079323 |  |  | 1 |  |
| LM511 | C0767106 |  |  | 1 |  |
| integrin alpha3beta1 | C0246766 |  |  | 1 |  |
| microRNA miR-130a | 406919 |  |  | 1 |  |
| CD68 | 287435 |  |  | 1 |  |
| NG2 proteoglycan | 1464 | 1 | 2 | 1 | 0.5 |
| postsynaptic density 95 | 116681 |  |  | 1 |  |
| CBL | 500985 |  |  | 1 |  |
| tuberous sclerosis complex 2 | 7249 | 1 | 1 | 1 | 1 |
| TSC 2 | 7249 | 1 | 1 | 1 | 1 |
| VEGFR2 | 3791 |  |  | 1 |  |
| VEGFR2 | 25589 |  |  | 1 |  |
| MAP Kinase SWIP-13 | C1456416 |  |  | 1 |  |
| ERK8 | 225689 |  |  | 1 |  |
| Swip | 23325 |  |  | 1 |  |
| SWIP-13 | 23325 |  |  | 1 |  |
| Repulsive Guidance Molecule | 56963 |  |  | 1 |  |
| RGMa | 56963 |  |  | 1 |  |
| RGMa | 244058 |  |  | 1 |  |
| antibody- | C0021027 | 1 | 2 | 1 | 0.5 |
| A missense MT-ND5 | 4540 |  |  | 1 |  |
| DROSHA | 29102 |  |  | 1 |  |
| acid sphingomyelinase | C0037903 |  | 1 | 1 | 1 |
| CB2 | 57302 |  |  | 1 |  |
| Ass1 | 25698 |  |  | 1 |  |
| PDI | 25506 |  |  | 1 |  |
| Tumor necrosis factor alpha (TNFA) gene | C1710304 | 2 |  | 1 |  |
| TNFA gene | C1710304 | 2 |  | 1 |  |
| Hk1 | 3098 |  |  | 1 |  |
| MA1 | 9240 |  |  | 1 |  |
| AF-6 | 40620 |  |  | 1 |  |
| Afadin | C0667316 |  |  | 1 |  |
| AF-6 | 4301 |  |  | 1 |  |
| Colony Stimulating Factor Receptor | C0080095 |  |  | 1 |  |
| colony stimulating factor-1 receptor | 1436 |  | 1 | 1 | 1 |
| CSF-1R | 1436 |  | 1 | 1 | 1 |
| CD115 | 1436 |  | 1 | 1 | 1 |
| CSF-1 | 1435 |  |  | 1 |  |
| Myo | 9499 |  |  | 1 |  |
| NBP | 4682 |  |  | 1 |  |
| extracellular signalregulated kinase 1 | 26417 |  |  | 1 |  |
| PP2 | 4888 |  |  | 1 |  |
| fluorinase | C1565193 |  |  | 1 |  |
| LARS2 | 23395 |  |  | 1 |  |
| ABCB9 | 23457 |  |  | 1 |  |
| RAB39B gene | 116442 |  |  | 1 |  |
| Ucp1 | 24860 |  |  | 1 |  |
| PGIS | 5740 |  |  | 1 |  |
| syndecans | C0075691 |  |  | 1 |  |
| integrin beta3 | 3690 |  | 2 | 1 | 0.5 |
| syndecan-1 | C1609943 |  |  | 1 |  |
| syndecan | 6382 |  |  | 1 |  |
| PKD | 5587 |  |  | 1 |  |
| Glycoprotein NMB | 10457 |  |  | 1 |  |
| transmembrane glycoprotein | 10457 |  |  | 1 |  |
| synaptotagmin 7 | 54525 |  |  | 1 |  |
| Doc-2 | 13132 |  |  | 1 |  |
| synaptotagmin 2 | 20980 |  |  | 1 |  |
| Somatostatin- | 6750 |  | 1 | 1 | 1 |
| a beta2 | 15002 |  |  | 1 |  |
| beta2AR | 11555 |  |  | 1 |  |
| UCA1 | 652995 |  |  | 1 |  |
| CISS-15 | 9244 |  |  | 1 |  |
| Discoidin domain receptor | C0285094 |  |  | 1 |  |
| discoidin domain receptors | C0285094 |  |  | 1 |  |
| DDRs | C0285094 |  |  | 1 |  |
| DDR2 | 4921 |  |  | 1 |  |
| microtubule-associated protein (MAP) tau | C2700455 |  |  | 1 |  |
| MAP tau | C2700455 |  |  | 1 |  |
| cysteine lysosomal protease | C0758959 |  |  | 1 |  |
| STBD1 | 8987 |  |  | 1 |  |
| SPATA19 | 219938 |  |  | 1 |  |
| epsilon-sarcoglycan | C0669353 |  |  | 1 |  |
| insulin receptor | 24954 |  |  | 1 |  |
| Multidrug Resistance Protein 2 | C0763572 |  |  | 1 |  |
| MRP2 | 1244 |  |  | 1 |  |
| BCRP | 9429 |  | 1 | 1 | 1 |
| NRLS | 4901 |  |  | 1 |  |
| CBL | 12402 |  |  | 1 |  |
| hbeta4 | 27345 |  |  | 1 |  |
| C. tepidum Roco protein | C0055817 |  |  | 1 |  |
| LMN | 4001 |  |  | 1 |  |
| RER1 | 11079 |  |  | 1 |  |
| NEDD4 | 4734 |  | 1 | 1 | 1 |
| Perilipin-2 | 11520 |  |  | 1 |  |
| cytoplasmic lipid droplet (CLD) protein | C1333198 | 2 | 3 | 1 | 0.333333333333333 |
| Plin2 | 11520 |  |  | 1 |  |
| HRt1 | 291 |  |  | 1 |  |
| jasplakinolide | C0165117 |  |  | 1 |  |
| Jas | C0165117 |  |  | 1 |  |
| MitoNEET | 52637 |  |  | 1 |  |
| CISD1 | 52637 |  |  | 1 |  |
| iron-sulfur containing protein | C0022095 |  | 1 | 1 | 1 |
| peptide hormones | C0597192 | 2 |  | 1 |  |
| rHMGB1 | 25459 |  |  | 1 |  |
| high-density lipoproteins | C0023821 | 2 |  | 1 |  |
| lipoprotein (a) | C0065058 |  |  | 1 |  |
| Lp(a) | C0065058 |  |  | 1 |  |
| ATXN10 | 25814 |  |  | 1 |  |
| ATXN10 gene | 25814 |  |  | 1 |  |
| DNAJC12 | 56521 |  |  | 1 |  |
| STPS | C3813590 |  |  | 1 |  |
| Adenosine A1 Receptor | 29290 |  |  | 1 |  |
| myelin oligodendrocyte glycoprotein | 4340 |  |  | 1 |  |
| mTF-miRNA-gene | C0812327 | 3 |  | 1 |  |
| SMN1 | 20595 |  |  | 1 |  |
| ROCK2 | 19878 |  | 1 | 1 | 1 |
| LIM kinase | 10611 |  |  | 1 |  |
| LIMK | 16885 |  |  | 1 |  |
| cofilin | C0056080 |  |  | 1 |  |
| OCM | 654231 |  |  | 1 |  |
| HIF-1alpha inhibitor | 55662 |  |  | 1 |  |
| HIR | 3761 | 1 |  | 1 |  |
| protein kinase-B | 2185 |  | 2 | 1 | 0.5 |
| PDE1A | 18573 |  |  | 1 |  |
| PDE1C | 18575 |  |  | 1 |  |
| APRIL | 69583 |  |  | 1 |  |
| E14 | 114729 |  |  | 1 |  |
| BCMA | 21935 |  |  | 1 |  |
| TNFRSF13A | 21935 |  |  | 1 |  |
| TACI | 57916 |  |  | 1 |  |
| TNFRSF13B | 57916 |  |  | 1 |  |
| Presenilins | C0872078 |  | 1 | 1 | 1 |
| presenilins 1 | C0299212 |  | 1 | 1 | 1 |
| anti-apoptotic Bcl-2 proteins | C1565114 | 1 | 3 | 1 | 0.333333333333333 |
| ATP5A1 | 498 |  |  | 1 |  |
| VDAC3 | 7419 |  |  | 1 |  |
| Kcnn2 | 54262 |  |  | 1 |  |
| SK2 | 54262 |  |  | 1 |  |
| BCKDK | 10295 |  |  | 1 |  |
| STX1B | 112755 |  |  | 1 |  |
| PKM2 | 5315 |  |  | 1 |  |
| ZNF134 | 7693 |  |  | 1 |  |
| ZNF160 | 90338 |  |  | 1 |  |
| Pancreatic Polypeptide | 5539 |  |  | 1 |  |
| pancreatic polypeptide | C0030298 |  |  | 1 |  |
| PP | C0030298 |  |  | 1 |  |
| pancreatic elastase enzyme | C0920330 |  |  | 1 |  |
| ACR-ACNM | 49 |  |  | 1 |  |
| Fyn tyrosine kinase | C0072475 |  |  | 1 |  |
| CAMK2A | 815 |  |  | 1 |  |
| tied | 9358 |  |  | 1 |  |
| ATG5 | 494180 |  |  | 1 |  |
| dopamine transporter | 80787 |  |  | 1 |  |
| DAT | 80787 |  |  | 1 |  |
| decyl-3-hydroxy-2 | C0607379 |  |  | 1 |  |
| DAC | 13 |  |  | 1 |  |
| moon lighting proteins | C0382507 |  |  | 1 |  |
| t=14.58 | 1070896 |  |  | 1 |  |
| t=10.39 | 1070370 |  |  | 1 |  |
| MAP2K2 | 5605 | 1 | 1 | 1 | 1 |
| ribosomal protein S6 kinase | C0073337 |  |  | 1 |  |
| MEK2 | 5605 | 1 | 1 | 1 | 1 |
| mitogen-activated protein kinase kinase 2 | 5605 | 1 | 1 | 1 | 1 |
| SLC4A11 | 83959 |  |  | 1 |  |
| Vps35 | 65114 |  |  | 1 |  |
| vacuolar protein sorting 35 | 65114 |  |  | 1 |  |
| SLC4A11 | 269356 |  |  | 1 |  |
| NCEH-1 | 189866 |  |  | 1 |  |
| neutral cholesterol ester hydrolase 1 | 57552 |  |  | 1 |  |
| M1-M5 | 100507027 |  |  | 1 |  |
| M1-M4 | 100507027 |  |  | 1 |  |
| grasp | 160622 |  |  | 1 |  |
| GDX | 8266 |  |  | 1 |  |
| streptavidin | C0075278 |  |  | 1 |  |
| GDX+ | 8266 |  |  | 1 |  |
| nuclear factor kappaB (NF-kappaB) p65 | C0214222 |  |  | 1 |  |
| transketolase | 7086 |  |  | 1 |  |
| AAV-PARIS | 17 |  | 1 | 1 | 1 |
| transketolase | 21881 |  |  | 1 |  |
| TKT | 21881 |  |  | 1 |  |
| TKT | 7086 |  |  | 1 |  |
| tuna | 100507043 |  |  | 1 |  |
| prop-1 | 5626 |  |  | 1 |  |
| prop-1 | 266738 |  |  | 1 |  |
| CIB1 | 10519 |  |  | 1 |  |
| CIB1 | 23991 |  |  | 1 |  |
| integrin alphaIIb | C0032191 |  |  | 1 |  |
| CIB1 gene | 10519 |  |  | 1 |  |
| Smurf1 | 57154 |  |  | 1 |  |
| Smad ubiquitination regulatory factor 1 | 57154 |  |  | 1 |  |
| Smurf1 | 690516 |  |  | 1 |  |
| adenosine A2A receptor | 102132487 |  |  | 1 |  |
| NADPH oxidase1 | 27035 |  | 2 | 1 | 0.5 |
| BAG3 | 293524 |  |  | 1 |  |
| BAG3 | 29810 |  |  | 1 |  |
| sequestosome 1 | 113894 |  |  | 1 |  |
| Atg5 | 365601 |  |  | 1 |  |
| TCPS | C1709995 |  |  | 1 |  |
| IAP | C0220248 |  |  | 1 |  |
| FLASH | 6474 |  |  | 1 |  |
| ApoA2 | 336 |  |  | 1 |  |
| Twist1 | 7291 |  | 1 | 1 | 1 |
| TGDS | 23483 |  |  | 1 |  |
| SCA19/22 | 7550920 |  |  | 1 |  |
| KCND3 | 3752 |  |  | 1 |  |
| Huntington's disease genetic | C0872189 | 1 | 1 | 1 | 1 |
| Whey protein isolate | C1883555 |  |  | 1 |  |
| WPI | C1883555 |  |  | 1 |  |
| FRDA | 2395 |  | 2 | 1 | 0.5 |
| cytokine antibody | C2732647 |  |  | 1 |  |
| IL-12 p40/p70 | 84959 |  |  | 1 |  |
| MCP-5 | 822 | 1 |  | 1 |  |
| GMF | C0061308 |  |  | 1 |  |
| mouse mast cell protease-6 | C1567158 |  |  | 1 |  |
| MMCP-6 | 17229 |  |  | 1 |  |
| MMCP-7 | 100503895 |  |  | 1 |  |
| tryptase | C0147080 |  |  | 1 |  |
| brain-specific serine protease-4 | 64063 |  |  | 1 |  |
| BSSP-4 | 70835 |  |  | 1 |  |
| tryptase/BSSP-4 | C0147080 |  |  | 1 |  |
| CD40L | 21947 |  |  | 1 |  |
| bean | 361358 |  |  | 1 |  |
| EC 3.4 | C0024188 |  | 1 | 1 | 1 |
| asparaginyl endopeptidase | C0104398 |  |  | 1 |  |
| RREB1 | 68750 |  |  | 1 |  |
| annexin A2 | 12306 |  |  | 1 |  |
| cystatin E/ | 1474 |  |  | 1 |  |
| TLC | 3933 |  |  | 1 |  |
| GAS6 | 2621 |  |  | 1 |  |
| STC | 6781 |  |  | 1 |  |
| matrix metalloproteinase-8 | 17394 |  |  | 1 |  |
| MMP-8 | 17394 |  |  | 1 |  |
| CSF-1 | 12977 |  |  | 1 |  |
| IL-34 | 76527 |  |  | 1 |  |
| proton pump | C0018440 |  |  | 1 |  |
| ATP6V1B2 | 526 |  |  | 1 |  |
| N-ethylmaleimide-sensitive fusion protein | C0067966 |  |  | 1 |  |
| NSF | 4905 |  |  | 1 |  |
| homocarnosine | C0062927 |  |  | 1 |  |
| Methionine sulfoxide reductase | C0066125 |  |  | 1 |  |
| Msr | 290947 |  |  | 1 |  |
| MsrA | 29447 |  |  | 1 |  |
| hexokinase 1 | 15275 |  |  | 1 |  |
| PTEN | 50557 |  |  | 1 |  |
| beta-synuclein | 6620 | 1 |  | 1 |  |
| cytochrome P450 family 1 subfamily A member 1 | 1543 | 1 | 1 | 1 | 1 |
| neuronal ceroid lipofuscinosis | 76524 |  |  | 1 |  |
| Cln3 | 12752 |  |  | 1 |  |
| NCL | 17975 |  |  | 1 |  |
| CLN3 | 39981 |  |  | 1 |  |
| Tel- | 2120 |  |  | 1 |  |
| Pax-4 | 5078 |  |  | 1 |  |
| Evi- | 79971 |  |  | 1 |  |
| COX17 | 10063 |  |  | 1 |  |
| COX7B | 1349 |  |  | 1 |  |
| COX6A1 | 1337 |  |  | 1 |  |
| ATP5J | 522 |  |  | 1 |  |
| type I bovine collagen | C0041455 |  |  | 1 |  |
| 4E-BP1 | 1978 |  | 1 | 1 | 1 |
| Tctex1 | 6993 |  |  | 1 |  |
| nuclear respiratory factor 1 | C1565068 |  | 1 | 1 | 1 |
| CADM2 | 253559 |  |  | 1 |  |
| LINGO2 | 158038 |  | 1 | 1 | 1 |
| Mdm2 | 4193 |  | 1 | 1 | 1 |
| nuclease | C0597094 | 1 |  | 1 |  |
| cysteine protease gene | C1333187 |  |  | 1 |  |
| flower | 11094 |  |  | 1 |  |
| AM1241 | 3171117 |  |  | 1 |  |
| CR3 | 16409 | 1 | 1 | 1 | 1 |
| Src | 20779 |  |  | 1 |  |
| NRBF2 | 29982 |  |  | 1 |  |
| nuclear receptor binding factor 2 | 29982 |  |  | 1 |  |
| NRBF2 | 641340 |  |  | 1 |  |
| G (AG | C0369248 |  |  | 1 |  |
| miR-22-3p | 407008 |  |  | 1 |  |
| crisprTFs | 1434 | 1 |  | 1 |  |
| VasP | 7408 |  |  | 1 |  |
| ghrelin O-acyltransferase | 619373 |  |  | 1 |  |
| ferroportin 1 | 170840 |  |  | 1 |  |
| TfR1 | 64678 | 1 |  | 1 |  |
| Fpn1 | 170840 |  |  | 1 |  |
| Ft-L | 29292 |  |  | 1 |  |
| Ding | 6045 |  |  | 1 |  |
| asa | C3853627 |  |  | 1 |  |
| DNMT3b gene | C1707617 |  |  | 1 |  |
| Rab1a | 5861 | 2 |  | 1 |  |
| hnRNPA1 | 3178 |  |  | 1 |  |
| PABP1 | 26980 |  |  | 1 |  |
| RICTOR | 78757 |  |  | 1 |  |
| Slc40a1 | 53945 |  |  | 1 |  |
| Hamp | 84506 |  |  | 1 |  |
| LRP1 | 4035 |  |  | 1 |  |
| LRPAP1 | 4043 |  | 1 | 1 | 1 |
| Checkpoint Kinase 1 | 1111 |  | 1 | 1 | 1 |
| checkpoint kinase 1 | 12649 |  |  | 1 |  |
| CHK1 | 12649 |  |  | 1 |  |
| activated protein C | 64663 | 2 | 3 | 1 | 0.333333333333333 |
| Grp94 | 22027 |  |  | 1 |  |
| Gp96 | 22027 |  |  | 1 |  |
| cdd | 978 |  |  | 1 |  |
| Lamp1 | 25328 |  |  | 1 |  |
| Lamp2 | 24944 |  |  | 1 |  |
| S100beta | 25742 |  |  | 1 |  |
| ATFS-1 | 179922 |  |  | 1 |  |
| Vps26 | 31144 |  |  | 1 |  |
| LOQs | 6895 |  |  | 1 |  |
| phenylethanolamine N-methyltransferase | 5409 |  |  | 1 |  |
| PNMT | 5409 |  |  | 1 |  |
| alcohol dehydrogenase | C0001942 |  | 1 | 1 | 1 |
| LEs | 2525 |  |  | 1 |  |
| M1-LFP | 100507027 |  |  | 1 |  |
| halorhodopsin | C0062108 |  |  | 1 |  |
| MCU | 90550 |  |  | 1 |  |
| mitochondrial calcium uptake 1 | 10367 |  |  | 1 |  |
| MICU1 | 10367 |  |  | 1 |  |
| NCLX | 80024 |  |  | 1 |  |
| MAPK | C1979930 |  |  | 1 |  |
| homologous bacterial Roco protein | C1512488 |  | 2 | 1 | 0.5 |
| cytoplasmic proteins | C1333198 | 2 | 3 | 1 | 0.333333333333333 |
| Lipoprotein-associated phospholipase A2 | 7941 |  |  | 1 |  |
| Lp-PLA2 | 7941 |  |  | 1 |  |
| CCAAT/Enhancer binding protein beta | C0209548 |  | 1 | 1 | 1 |
| CCAAT/Enhancer binding protein beta | 1051 |  | 1 | 1 | 1 |
| GPCR | 227289 |  |  | 1 |  |
| CD-1 | 111334 | 1 | 2 | 1 | 0.5 |
| inhibitory PAS domain protein | 64344 |  |  | 1 |  |
| IPAS | 53417 |  |  | 1 |  |
| MAPK-activated protein kinase 2 | 9261 |  | 1 | 1 | 1 |
| MAPKAPK2 | 289014 |  |  | 1 |  |
| MK2 | 3737 |  |  | 1 |  |
| interleukin-2 | 16183 |  |  | 1 |  |
| mlp | 8048 |  |  | 1 |  |
| SYNJ1 | 104015 |  |  | 1 |  |
| synaptojanin1 | 104015 |  |  | 1 |  |
| synapsin I/II | C0087045 |  |  | 1 |  |
| Sri | 6717 | 1 |  | 1 |  |
| SCA1 | 6310 |  |  | 1 |  |
| ATXN1 | 6310 |  |  | 1 |  |
| PPP2R2B | 5521 |  |  | 1 |  |
| 5-HT1F Receptor- | C0534690 |  |  | 1 |  |
| 5-HT1F receptor | C0534690 |  |  | 1 |  |
| cytochrome P450 oxidoreductase | C0027310 |  |  | 1 |  |
| ATP7A | 538 |  |  | 1 |  |
| SLC45A4 | 57210 |  |  | 1 |  |
| LAT-1 | 8140 | 1 |  | 1 |  |
| large neutral amino acid transporter-1 | C0293237 |  |  | 1 |  |
| MK-0657 | 1476758 |  |  | 1 |  |
| DTW | 64856 |  |  | 1 |  |
| Warping | 64856 |  |  | 1 |  |
| copper-/zinc-binding proteins | C0056309 |  |  | 1 |  |
| MSD | 4434 |  |  | 1 |  |
| CCL-11 | 6356 |  |  | 1 |  |
| eotaxin-3 | 10344 |  |  | 1 |  |
| CCL-26 | 10344 |  |  | 1 |  |
| thymus and activation-regulated chemokine | 6361 |  |  | 1 |  |
| TARC | 6361 |  |  | 1 |  |
| CCL-17 | 6361 |  |  | 1 |  |
| CCL17 | 6361 |  |  | 1 |  |
| Pannexin1 | 24145 |  |  | 1 |  |
| Panx1 | 24145 |  |  | 1 |  |
| Cu/ZnSOD | 24786 |  | 1 | 1 | 1 |
| DKK-1 | 293897 |  | 1 | 1 | 1 |
| cyclinD1 | 58919 | 2 |  | 1 |  |
| 3T3-L1 | 28938 |  |  | 1 |  |
| adiponectin | C0389071 |  |  | 1 |  |
| resistin | C0963992 |  |  | 1 |  |
| myelin-associated oligodendrocyte basic protein | C0014063 |  |  | 1 |  |
| syntaxin 6 | 10228 |  |  | 1 |  |
| Pam3Cys | C0068258 |  |  | 1 |  |
| tensin homologue-induced putative kinase 1 | 7145 | 1 | 3 | 1 | 0.333333333333333 |
| hBest1 | 7439 |  |  | 1 |  |
| human bestrophin-1 (hBest1) - transmembrane Ca2+-activated chloride channel protein | 7439 |  |  | 1 |  |
| HYS | 4201 |  |  | 1 |  |
| Let-7 | 266952 |  |  | 1 |  |
| Let-7d | 406886 |  |  | 1 |  |
| hnRNP F | 3185 |  |  | 1 |  |
| hnRNP Q | 10492 |  |  | 1 |  |
| Angiotensin 1-7 | C0103306 |  |  | 1 |  |
| Ang II type 1 | C0003006 | 1 |  | 1 |  |
| phosphatidylinositol 3-kinases | C2936824 |  |  | 1 |  |
| TNT | 7138 |  |  | 1 |  |
| c)2017 | 1036093 |  |  | 1 |  |
| Shp-2 | 25622 |  |  | 1 |  |
| aspartame | C0003999 |  |  | 1 |  |
| l-phenylalanine methyl ester | C0070635 |  |  | 1 |  |
| casein kinase 2 | C0108555 | 1 | 1 | 1 | 1 |
| CK2 | 13000 |  |  | 1 |  |
| actomyosin | C0001291 |  |  | 1 |  |
| FAM134A | 79137 |  |  | 1 |  |
| myosin II | C0027103 |  |  | 1 |  |
| Mdn | 56953 |  |  | 1 |  |
| SEb | 26040 |  |  | 1 |  |
| catalase | 30068 |  |  | 1 |  |
| sod1 | 30553 |  |  | 1 |  |
| sod2 | 335799 |  |  | 1 |  |
| bcl2 | 570772 |  |  | 1 |  |
| bax | 58081 |  |  | 1 |  |
| GRF | 2691 |  |  | 1 |  |
| hD1 | 3065 |  |  | 1 |  |
| iPLA2gamma | 50640 |  |  | 1 |  |
| iPLA2 | 360426 |  |  | 1 |  |
| Sox9 | 6662 |  |  | 1 |  |
| Sox9 | 20682 |  |  | 1 |  |
| suppressor gene | C0017372 |  |  | 1 |  |
| klotho | 83504 |  |  | 1 |  |
| protein kinase A (PKA) inhibitor | C0243459 | 1 |  | 1 |  |
| gamma-glutamyltransferase | C0017040 |  |  | 1 |  |
| gamma glutamyltransferase | C0017040 |  |  | 1 |  |
| GGT | C0017040 |  |  | 1 |  |
| GTP-binding protein-coupled receptors | 92170 | 1 |  | 1 |  |
| serine hydrolases | C1335948 |  |  | 1 |  |
| Ah receptor | C0052441 |  |  | 1 |  |
| tauT | 6533 |  |  | 1 |  |
| BI-2 | 243423 |  |  | 1 |  |
| NT-3 | 4877 |  |  | 1 |  |
| NT-4 | 4909 |  |  | 1 |  |
| NT-5 | 4909 |  |  | 1 |  |
| CHCHD10 | 103172 |  |  | 1 |  |
| CHCHD2 | 14004 |  |  | 1 |  |
| p32 | 708 |  |  | 1 |  |
| GC1QR | 708 |  |  | 1 |  |
| dipeptidyl peptidase-4 | 1803 |  |  | 1 |  |
| RNA-binding proteins | C0085177 | 1 |  | 1 |  |
| PGR | 5241 |  |  | 1 |  |
| miR185 | 406961 |  |  | 1 |  |
| microRNA185 | 406961 |  |  | 1 |  |
| AMPK inhibitor | C1449702 |  |  | 1 |  |
| miR17 | 406952 |  |  | 1 |  |
| membrane metalloendopeptidase | 4311 |  |  | 1 |  |
| MME | 4311 |  |  | 1 |  |
| cyclic peptide | C0030957 |  |  | 1 |  |
| connective tissue growth factor | C0110610 |  |  | 1 |  |
| CTGF | 14219 |  |  | 1 |  |
| LETM1 | 3954 |  |  | 1 |  |
| LETM1-T192E | 3954 |  |  | 1 |  |
| MHC class II genes | C0017349 |  | 1 | 1 | 1 |
| TO-RGN | 9104 |  | 1 | 1 | 1 |
| Rab8A | 17274 |  |  | 1 |  |
| Rab10 | 19325 |  |  | 1 |  |
| Rab12 | 19328 |  |  | 1 |  |
| Rab29 | 226422 |  |  | 1 |  |
| Rab35 | 77407 |  |  | 1 |  |
| Rab43 | 69834 |  |  | 1 |  |
| RILPL1 | 75695 |  |  | 1 |  |
| RILPL2 | 80291 |  |  | 1 |  |
| primary cilia formation | 128344 |  |  | 1 |  |
| phospho-specific Rab protein antibodies | C1257988 | 2 |  | 1 |  |
| Rab35 | 11021 |  |  | 1 |  |
| Rab43 | 339122 |  |  | 1 |  |
| CTh | 23584 |  |  | 1 |  |
| pattern recognition receptors | C1564907 |  |  | 1 |  |
| HLH-30 | 177157 |  |  | 1 |  |
| DNA-damage-inducible transcript 4 | 54541 |  |  | 1 |  |
| Ddit4 | 54541 |  |  | 1 |  |
