# Supplementary material for: Menagerie: A text-mining tool to support animal-human translation in neurodegeneration research
Source: PLoS One. 2019 Dec 17;14(12):e0226176. doi: 10.1371/journal.pone.0226176 (PMC6917268; doi:10.1371/journal.pone.0226176)
Supplement: S4 Table — Genes are listed by text term and corresponding NCBI or UMLS identifier and are ranked in descending order. (DOCX) [file pone.0226176.s005.docx]

**S4 Table.** Genes extracted from entire abstract in papers in which L-DOPA or alpha-synuclein were extracted by the Interventions module. Genes are listed by text term and corresponding NCBI or UMLS identifier and are ranked in descending order.

| **L-DOPA , ID D007980, n= 353 PMIDs** | | |  |  |  |  |  | **Alpha-synuclein, ID D051844, n =516** | | |  |  |  |  |
| --- | --- | --- | --- | --- | --- | --- | --- | --- | --- | --- | --- | --- | --- | --- |
| **GENE TERM** | **TERM_ID** | **Source** | **2008** | **2012** | **2017** | **All** |  | **GENE TERM** | **TERM_ID** | **Source** | **2008** | **2012** | **2017** | **All** |
| *Total # PMIDS* |  |  | *92* | *121* | *140* | *353* |  | *Total # PMIDS* |  |  | 138 | 165 | 213 | 516 |
| *Gene not reported* |  |  | *32* | *38* | *60* | *130* |  | *Gene not reported* |  |  | 0 | 0 | 0 | 0 |
| dopamine receptor | C0034798 | UMLS | 3 | 5 | 5 | 13 |  | Alpha-synuclein | 6622 | NCBI | 138 | 165 | 213 | 516 |
| tyrosine hydroxylase | 7054 | NCBI | 4 | 6 | 3 | 13 |  | alpha-synuclein's | C0285890 | UMLS | 26 | 32 | 40 | 98 |
| COMT | 1312 | NCBI | 6 | 3 | 3 | 12 |  | alphaSYN | 20617 | NCBI | 9 | 14 | 15 | 38 |
| dopamine transporter | C0114838 | UMLS | 5 | 2 | 4 | 11 |  | amyloid | C0002716 | UMLS | 11 | 10 | 14 | 35 |
| Delta FosB | 2354 | NCBI | 4 | 3 | 4 | 11 |  | amyloid fibrils | C1449651 | UMLS | 6 | 10 | 12 | 28 |
| D2R | 1813 | NCBI | 2 | 3 | 5 | 10 |  | prion proteins | C0033164 | UMLS | 2 | 6 | 15 | 23 |
| NMDA receptor | C0080093 | UMLS | 0 | 4 | 4 | 8 |  | ubiquitin | C0041538 | UMLS | 7 | 7 | 3 | 17 |
| extracellular signal-regulated kinase | C0600388 | UMLS | 2 | 1 | 4 | 7 |  | tau | 4137 | NCBI | 7 | 4 | 5 | 16 |
| D2 receptors | C0058698 | UMLS | 3 | 0 | 4 | 7 |  | tyrosine hydroxylase | 7054 | NCBI | 4 | 6 | 6 | 16 |
| ERK1 | 5595 | NCBI | 0 | 4 | 3 | 7 |  | sHSP | C0033684 | UMLS | 2 | 9 | 5 | 16 |
| Homocysteine | C0019878 | UMLS | 1 | 3 | 2 | 6 |  | Synuclein | C0165073 | UMLS | 3 | 6 | 7 | 16 |
| alanine | C0001898 | UMLS | 1 | 3 | 2 | 6 |  | molecular chaperone | C0243041 | UMLS | 7 | 6 | 3 | 16 |
| Alpha-synuclein | 6622 | NCBI | 0 | 1 | 5 | 6 |  | SYN | 2534 | NCBI | 4 | 2 | 9 | 15 |
| 5-HT(1A) receptors | C0379900 | UMLS | 1 | 3 | 1 | 5 |  | proteasome | C0208355 | UMLS | 6 | 7 | 2 | 15 |
| NMDA | C0079883 | UMLS | 1 | 3 | 1 | 5 |  | LRRK2 | 120892 | NCBI | 3 | 6 | 5 | 14 |
| tyrosine hydroxylase | C0041491 | UMLS | 2 | 2 | 1 | 5 |  | protease K | C0059256 | UMLS | 3 | 2 | 7 | 12 |
| DARPP-32 | 360616 | NCBI | 1 | 2 | 2 | 5 |  | Amyloid-beta (Abeta) peptide | 351 | NCBI | 6 | 4 | 1 | 11 |
| DAT | 6531 | NCBI | 1 | 1 | 3 | 5 |  | Syn | 23336 | NCBI | 4 | 2 | 3 | 9 |
| AADC | 1644 | NCBI | 1 | 4 | 0 | 5 |  | lysine | C0024337 | UMLS | 4 | 2 | 3 | 9 |
| aromatic l-amino acid decarboxylase | C1412028 | UMLS | 0 | 2 | 2 | 4 |  | amyloid fibril forming protein | C1456454 | UMLS | 3 | 3 | 3 | 9 |
| 3-O-methyldopa | C0964304 | UMLS | 3 | 0 | 1 | 4 |  | Rare | C1514917 | UMLS | 5 | 4 | 0 | 9 |
| glutamate receptors | C0061465 | UMLS | 1 | 1 | 2 | 4 |  | Thy1 | 21838 | NCBI | 3 | 3 | 2 | 8 |
| DA D1 receptors | C0058697 | UMLS | 1 | 2 | 1 | 4 |  | green fluorescent protein | C0120285 | UMLS | 0 | 1 | 7 | 8 |
| alpha-methyldopa | C0025741 | UMLS | 3 | 1 | 0 | 4 |  | beta-synuclein | C0245467 | UMLS | 2 | 3 | 3 | 8 |
| cysteine | C0010654 | UMLS | 0 | 2 | 2 | 4 |  | glucocerebrosidase | 2629 | NCBI | 0 | 2 | 5 | 7 |
| dopa decarboxylase | C0003806 | UMLS | 0 | 3 | 1 | 4 |  | human Haptoglobin (Hp) protein | 4929 | NCBI | 1 | 4 | 2 | 7 |
| FosB | 1E+08 | NCBI | 2 | 0 | 2 | 4 |  | NAc | 7504 | NCBI | 2 | 3 | 2 | 7 |
| NR2B | 24410 | NCBI | 0 | 2 | 2 | 4 |  | proteome | C0751973 | UMLS | 6 | 1 | 0 | 7 |
| dopamine receptor D1 | 13488 | NCBI | 0 | 2 | 2 | 4 |  | natively unfolded proteins | C3658247 | UMLS | 4 | 2 | 1 | 7 |
| tau | 4137 | NCBI | 0 | 3 | 1 | 4 |  | top | 7064 | NCBI | 0 | 2 | 4 | 6 |
| 5-HT1A receptor | 3350 | NCBI | 1 | 3 | 0 | 4 |  | leucine-rich repeat kinase 2 | 66725 | NCBI | 1 | 3 | 2 | 6 |
| Adenosine A2A receptor | 135 | NCBI | 1 | 0 | 3 | 4 |  | recombinant proteins | C0034861 | UMLS | 3 | 0 | 3 | 6 |
| receptor- | C0597357 | UMLS | 0 | 1 | 2 | 3 |  | protein structure | C0175168 | UMLS | 2 | 3 | 1 | 6 |
| aspartate receptor | C0052536 | UMLS | 1 | 0 | 2 | 3 |  | Hsp70 | C0243043 | UMLS | 2 | 2 | 2 | 6 |
| TH | C0041485 | UMLS | 0 | 3 | 0 | 3 |  | protein or 'canonical' amino acids | C1874187 | UMLS | 1 | 4 | 1 | 6 |
| nicotinic acetylcholine receptor | C0034830 | UMLS | 1 | 1 | 1 | 3 |  | APOE | 348 | NCBI | 3 | 1 | 1 | 5 |
| TH | C0020364 | UMLS | 1 | 1 | 1 | 3 |  | Rep1 | 1121 | NCBI | 2 | 3 | 0 | 5 |
| glutamine | C0017797 | UMLS | 0 | 1 | 2 | 3 |  | Hsp70 | 3308 | NCBI | 4 | 0 | 1 | 5 |
| FOG | 161882 | NCBI | 1 | 1 | 1 | 3 |  | IFN-gamma | 3458 | NCBI | 1 | 1 | 3 | 5 |
| DAT | 24898 | NCBI | 1 | 1 | 1 | 3 |  | IL-1beta | 3553 | NCBI | 3 | 1 | 1 | 5 |
| NR2A | 24409 | NCBI | 0 | 1 | 2 | 3 |  | parkin | 5071 | NCBI | 1 | 2 | 2 | 5 |
| ERK | 24338 | NCBI | 0 | 2 | 1 | 3 |  | DAT | 6531 | NCBI | 2 | 1 | 2 | 5 |
| mGluR5 | 14805 | NCBI | 1 | 0 | 2 | 3 |  | Thy1 | 7070 | NCBI | 0 | 3 | 2 | 5 |
| CREB | 12912 | NCBI | 0 | 0 | 3 | 3 |  | TNF-alpha | 7124 | NCBI | 2 | 0 | 3 | 5 |
| monoamine oxidase type B | 4129 | NCBI | 2 | 1 | 0 | 3 |  | PLK-2 | 10769 | NCBI | 0 | 3 | 2 | 5 |
| metagenome | C2717745 | UMLS | 0 | 0 | 2 | 2 |  | DJ-1 | 11315 | NCBI | 0 | 1 | 4 | 5 |
| neuronal nitric oxide synthase | C0669368 | UMLS | 0 | 1 | 1 | 2 |  | PINK1 | 65018 | NCBI | 1 | 3 | 1 | 5 |
| metabotropic glutamate receptor 5 | C0530778 | UMLS | 0 | 0 | 2 | 2 |  | tyrosine hydroxylase | C0041491 | UMLS | 1 | 1 | 3 | 5 |
| nociceptin/orphanin FQ | C0382336 | UMLS | 1 | 1 | 0 | 2 |  | binding protein | C0242210 | UMLS | 2 | 0 | 3 | 5 |
| metabotropic glutamate receptors | C0206529 | UMLS | 1 | 1 | 0 | 2 |  | beta-strands | C1706935 | UMLS | 2 | 3 | 0 | 5 |
| green fluorescent protein | C0120285 | UMLS | 0 | 0 | 2 | 2 |  | AFM | 173 | NCBI | 1 | 1 | 2 | 4 |
| coenzyme Q-10 | C0077666 | UMLS | 1 | 1 | 0 | 2 |  | htt | 3064 | NCBI | 1 | 2 | 1 | 4 |
| cyclic-AMP response element binding protein | C0056695 | UMLS | 0 | 0 | 2 | 2 |  | PARK9 | 23400 | NCBI | 0 | 2 | 2 | 4 |
| tryptophan hydroxylase | C0041252 | UMLS | 1 | 1 | 0 | 2 |  | LC3 | 84557 | NCBI | 0 | 1 | 3 | 4 |
| phenylalanine | C0031453 | UMLS | 0 | 1 | 1 | 2 |  | Mol | 90527 | NCBI | 2 | 1 | 1 | 4 |
| methionine | C0025646 | UMLS | 0 | 1 | 1 | 2 |  | Ferritin | C0015879 | UMLS | 1 | 1 | 2 | 4 |
| histones | C0019652 | UMLS | 1 | 0 | 1 | 2 |  | luciferase | C0024075 | UMLS | 1 | 1 | 2 | 4 |
| glycine | C0017890 | UMLS | 0 | 1 | 1 | 2 |  | phosphatases | C0031678 | UMLS | 1 | 1 | 2 | 4 |
| GSH | C0017817 | UMLS | 0 | 2 | 0 | 2 |  | amyloid (beta-sheeted proteins | C0078939 | UMLS | 1 | 0 | 3 | 4 |
| enkephalin | C0014298 | UMLS | 1 | 0 | 1 | 2 |  | coding region | C0079941 | UMLS | 1 | 2 | 1 | 4 |
| dynorphin | C0013355 | UMLS | 1 | 1 | 0 | 2 |  | specific p53 antibody | C0443640 | UMLS | 2 | 1 | 1 | 4 |
| protein kinase A | C0010531 | UMLS | 0 | 2 | 0 | 2 |  | homology | C1334043 | UMLS | 1 | 0 | 3 | 4 |
| amyloid | C0002716 | UMLS | 0 | 0 | 2 | 2 |  | Abeta | C1705543 | UMLS | 1 | 2 | 1 | 4 |
| 5-hydroxytryptophan | C0000578 | UMLS | 0 | 1 | 1 | 2 |  | amyloid-beta | C3484390 | UMLS | 2 | 1 | 1 | 4 |
| cyclin-dependent kinase 5 | 140908 | NCBI | 1 | 1 | 0 | 2 |  | Membrane-bound | 19 | NCBI | 2 | 0 | 1 | 3 |
| GluA1 | 50592 | NCBI | 0 | 2 | 0 | 2 |  | Akt | 207 | NCBI | 1 | 0 | 2 | 3 |
| enkephalin | 29237 | NCBI | 0 | 2 | 0 | 2 |  | disease-associated genes | 925 | NCBI | 0 | 0 | 3 | 3 |
| D1-4 | 25802 | NCBI | 0 | 1 | 1 | 2 |  | EPR | 2069 | NCBI | 3 | 0 | 0 | 3 |
| caspase-3 | 25402 | NCBI | 1 | 1 | 0 | 2 |  | 3'UTR | 2837 | NCBI | 0 | 1 | 2 | 3 |
| adenosine A2A receptor | 25369 | NCBI | 0 | 0 | 2 | 2 |  | p62 | 2965 | NCBI | 0 | 0 | 3 | 3 |
| 5-HT1A | 24473 | NCBI | 1 | 1 | 0 | 2 |  | spin | 2969 | NCBI | 1 | 1 | 1 | 3 |
| adenosine A(2A) receptor | 11540 | NCBI | 0 | 0 | 2 | 2 |  | Hsc70 | 3312 | NCBI | 0 | 2 | 1 | 3 |
| tyrosinase | 7299 | NCBI | 0 | 1 | 1 | 2 |  | interleukin 6 | 3569 | NCBI | 1 | 0 | 2 | 3 |
| NR1A/2 | 7068 | NCBI | 2 | 0 | 0 | 2 |  | Smn-/- | 3925 | NCBI | 1 | 1 | 1 | 3 |
| Nociceptin | 5368 | NCBI | 1 | 1 | 0 | 2 |  | PSP | 4477 | NCBI | 0 | 3 | 0 | 3 |
| preproenkephalin | 5179 | NCBI | 1 | 1 | 0 | 2 |  | iNOS | 4843 | NCBI | 0 | 2 | 1 | 3 |
| parkin | 5071 | NCBI | 0 | 1 | 1 | 2 |  | Prolyl oligopeptidase | 5550 | NCBI | 1 | 1 | 1 | 3 |
| orphanin FQ receptor | 4987 | NCBI | 1 | 1 | 0 | 2 |  | PRNP | 5621 | NCBI | 0 | 0 | 3 | 3 |
| 3-OMD | 4958 | NCBI | 2 | 0 | 0 | 2 |  | tyrosinase | 7299 | NCBI | 2 | 1 | 0 | 3 |
| MTHFR | 4524 | NCBI | 0 | 1 | 1 | 2 |  | UCHL1 | 7345 | NCBI | 0 | 1 | 2 | 3 |
| GRK6 | 2870 | NCBI | 1 | 1 | 0 | 2 |  | IL-1beta | 16176 | NCBI | 0 | 2 | 1 | 3 |
| GCH1 | 2643 | NCBI | 0 | 0 | 2 | 2 |  | neuronal nitric oxide synthase | 18125 | NCBI | 0 | 2 | 1 | 3 |
| SYN(- | 2534 | NCBI | 0 | 0 | 2 | 2 |  | tumor necrosis factor alpha | 21926 | NCBI | 0 | 1 | 2 | 3 |
| dopamine D1 receptor | 1812 | NCBI | 0 | 1 | 1 | 2 |  | silent information regulator 2 | 22933 | NCBI | 0 | 1 | 2 | 3 |
| kit | C3853650 | UMLS | 1 | 0 | 0 | 1 |  | SNCA | 29219 | NCBI | 0 | 1 | 2 | 3 |
| GPCRs | C3812695 | UMLS | 0 | 1 | 0 | 1 |  | M83 | 58986 | NCBI | 0 | 1 | 2 | 3 |
| GP-EX | C3811116 | UMLS | 0 | 0 | 1 | 1 |  | actin | C0001271 | UMLS | 2 | 0 | 1 | 3 |
| aid | C3540469 | UMLS | 0 | 0 | 1 | 1 |  | cysteine | C0010654 | UMLS | 2 | 1 | 0 | 3 |
| amyloid-beta | C3484390 | UMLS | 0 | 0 | 1 | 1 |  | glial fibrillary acidic protein | C0017626 | UMLS | 0 | 1 | 2 | 3 |
| per 1 | C3273591 | UMLS | 0 | 1 | 0 | 1 |  | stress signaling protein | C0018850 | UMLS | 1 | 0 | 2 | 3 |
| mTORC | C3152110 | UMLS | 0 | 0 | 1 | 1 |  | DSR | C0036720 | UMLS | 0 | 1 | 2 | 3 |
| E3 ubiquitin ligase Parkin | C2983548 | UMLS | 0 | 1 | 0 | 1 |  | nitrotyrosine | C0047645 | UMLS | 2 | 1 | 0 | 3 |
| Receptor activity modifying proteins | C2936792 | UMLS | 1 | 0 | 0 | 1 |  | casein kinase | C0054846 | UMLS | 2 | 1 | 0 | 3 |
| ionotropic glutamate receptors | C2936399 | UMLS | 0 | 1 | 0 | 1 |  | dityrosine | C0058505 | UMLS | 2 | 1 | 0 | 3 |
| heat shock transcription factor-1 | C2348945 | UMLS | 1 | 0 | 0 | 1 |  | dopamine transporter | C0114838 | UMLS | 1 | 0 | 2 | 3 |
| calcium-dependent proteins | C2064889 | UMLS | 0 | 1 | 0 | 1 |  | vesicular monoamine transporter 2 | C0246932 | UMLS | 1 | 0 | 2 | 3 |
| protein or 'canonical' amino acids | C1874187 | UMLS | 0 | 1 | 0 | 1 |  | receptor- | C0597357 | UMLS | 1 | 0 | 2 | 3 |
| glutamate-glutamine | C1718801 | UMLS | 0 | 0 | 1 | 1 |  | secreted exosomal proteins | C0597427 | UMLS | 1 | 1 | 1 | 3 |
| protein phosphatase 2A | C1704708 | UMLS | 0 | 1 | 0 | 1 |  | disease susceptibility gene | C0919453 | UMLS | 2 | 1 | 0 | 3 |
| IL-1 | C1522428 | UMLS | 1 | 0 | 0 | 1 |  | mitochondria-specific proteins | C0949610 | UMLS | 0 | 2 | 1 | 3 |
| Rare | C1514917 | UMLS | 0 | 1 | 0 | 1 |  | ortholog | C1335144 | UMLS | 0 | 1 | 2 | 3 |
| signaling pathways. alpha-syn protein | C1335962 | UMLS | 0 | 0 | 1 | 1 |  | Iba1 | 199 | NCBI | 0 | 2 | 0 | 2 |
| LRP8 gene | C1335671 | UMLS | 0 | 0 | 1 | 1 |  | cAMP response element-binding protein | 820 | NCBI | 0 | 1 | 1 | 2 |
| functional component | C1179435 | UMLS | 0 | 0 | 1 | 1 |  | caspase-3 | 836 | NCBI | 0 | 0 | 2 | 2 |
| scaffolding protein | C1179132 | UMLS | 0 | 1 | 0 | 1 |  | CD4 | 920 | NCBI | 0 | 0 | 2 | 2 |
| adaptor protein | C1135629 | UMLS | 1 | 0 | 0 | 1 |  | CIs | 1154 | NCBI | 1 | 1 | 0 | 2 |
| G-protein alpha subunit | C0887847 | UMLS | 0 | 0 | 1 | 1 |  | Cathepsin D | 1509 | NCBI | 1 | 0 | 1 | 2 |
| GRK | C0872383 | UMLS | 1 | 0 | 0 | 1 |  | Fos protein | 2353 | NCBI | 0 | 1 | 1 | 2 |
| G protein-coupled receptor kinases | C0872043 | UMLS | 1 | 0 | 0 | 1 |  | Nrf2 | 2551 | NCBI | 0 | 1 | 1 | 2 |
| protein function | C0815043 | UMLS | 0 | 0 | 1 | 1 |  | hint | 3094 | NCBI | 0 | 0 | 2 | 2 |
| proteome | C0751973 | UMLS | 1 | 0 | 0 | 1 |  | Rab | 3267 | NCBI | 1 | 1 | 0 | 2 |
| G-protein coupled receptors | C0682972 | UMLS | 1 | 0 | 0 | 1 |  | human insulin receptor | 3643 | NCBI | 0 | 0 | 2 | 2 |
| mGluR agonist L-(+)-2-amino-4 | C0667286 | UMLS | 0 | 1 | 0 | 1 |  | polo-like kinase | 5347 | NCBI | 0 | 1 | 1 | 2 |
| arrestin3 | C0660239 | UMLS | 1 | 0 | 0 | 1 |  | Neurosin | 5653 | NCBI | 1 | 0 | 1 | 2 |
| synthetic enzyme | C0597548 | UMLS | 1 | 0 | 0 | 1 |  | cyclooxygenase-2 | 5743 | NCBI | 2 | 0 | 0 | 2 |
| 5-HT1B receptor | C0535025 | UMLS | 1 | 0 | 0 | 1 |  | Siah-1 | 6477 | NCBI | 2 | 0 | 0 | 2 |
| N/OFQ receptor | C0529964 | UMLS | 0 | 1 | 0 | 1 |  | VMAT2 | 6571 | NCBI | 1 | 0 | 1 | 2 |
| DPDPE | C0525856 | UMLS | 0 | 1 | 0 | 1 |  | MSA | 7173 | NCBI | 0 | 1 | 1 | 2 |
| specific p53 antibody | C0443640 | UMLS | 0 | 0 | 1 | 1 |  | Hsp | 7190 | NCBI | 0 | 0 | 2 | 2 |
| gamma-secretases | C0379528 | UMLS | 0 | 0 | 1 | 1 |  | coil | 8161 | NCBI | 1 | 1 | 0 | 2 |
| CB1 receptors | C0378126 | UMLS | 0 | 0 | 1 | 1 |  | UTR | 8170 | NCBI | 0 | 0 | 2 | 2 |
| arrestins | C0376570 | UMLS | 1 | 0 | 0 | 1 |  | light | 8740 | NCBI | 0 | 1 | 1 | 2 |
| aromatic amino acid | C0301713 | UMLS | 0 | 0 | 1 | 1 |  | A beta | 8803 | NCBI | 1 | 0 | 1 | 2 |
| Chlorotoxin | C0293227 | UMLS | 0 | 1 | 0 | 1 |  | wave | 8936 | NCBI | 1 | 0 | 1 | 2 |
| pace | C0287990 | UMLS | 0 | 0 | 1 | 1 |  | atg5 gene | 9474 | NCBI | 0 | 1 | 1 | 2 |
| synaptotagmin 4 | C0286878 | UMLS | 1 | 0 | 0 | 1 |  | PUFA | 9933 | NCBI | 1 | 0 | 1 | 2 |
| Src family tyrosine kinases | C0282625 | UMLS | 0 | 0 | 1 | 1 |  | dynamin-related protein 1 | 10059 | NCBI | 0 | 1 | 1 | 2 |
| RPTP-zeta/beta | C0253470 | UMLS | 1 | 0 | 0 | 1 |  | miR-7 | 10859 | NCBI | 0 | 0 | 2 | 2 |
| anaplastic lymphoma kinase | C0252409 | UMLS | 1 | 0 | 0 | 1 |  | p25alpha | 11076 | NCBI | 0 | 0 | 2 | 2 |
| Galphaolf | C0249519 | UMLS | 0 | 0 | 1 | 1 |  | cathepsin D | 13033 | NCBI | 1 | 0 | 1 | 2 |
| Gly-Pro-Glu | C0245891 | UMLS | 0 | 1 | 0 | 1 |  | Fyn | 14360 | NCBI | 0 | 0 | 2 | 2 |
| glycogen synthase kinase-3 | C0244989 | UMLS | 1 | 0 | 0 | 1 |  | GFAP | 14580 | NCBI | 0 | 1 | 1 | 2 |
| CB2 receptors | C0208757 | UMLS | 0 | 0 | 1 | 1 |  | NMDAR | 14810 | NCBI | 0 | 0 | 2 | 2 |
| proteasome | C0208355 | UMLS | 0 | 1 | 0 | 1 |  | HSP70 | 15511 | NCBI | 0 | 1 | 1 | 2 |
| immediate early gene | C0206256 | UMLS | 0 | 0 | 1 | 1 |  | Nrf2 | 18024 | NCBI | 0 | 2 | 0 | 2 |
| GTP cyclohydrolase I | C0205775 | UMLS | 0 | 1 | 0 | 1 |  | Nurr1 | 18227 | NCBI | 0 | 2 | 0 | 2 |
| serotonin transporter | C0170657 | UMLS | 0 | 1 | 0 | 1 |  | PrP(c) | 19122 | NCBI | 0 | 0 | 2 | 2 |
| extracellular signal-regulated kinases 1 and 2 | C0170168 | UMLS | 0 | 1 | 0 | 1 |  | polo-like kinase 2 | 20620 | NCBI | 0 | 1 | 1 | 2 |
| beta-arrestin | C0167464 | UMLS | 0 | 0 | 1 | 1 |  | VIP | 22353 | NCBI | 0 | 2 | 0 | 2 |
| N-syndecan | C0167183 | UMLS | 1 | 0 | 0 | 1 |  | SIR2 | 23411 | NCBI | 0 | 2 | 0 | 2 |
| MDL-28170 | C0165174 | UMLS | 0 | 1 | 0 | 1 |  | DAT | 24898 | NCBI | 1 | 0 | 1 | 2 |
| delta opioid receptors | C0140057 | UMLS | 0 | 1 | 0 | 1 |  | FGF20 | 26281 | NCBI | 2 | 0 | 0 | 2 |
| casein kinase II | C0108555 | UMLS | 0 | 0 | 1 | 1 |  | swapping | 50618 | NCBI | 0 | 1 | 1 | 2 |
| G-protein | C0086376 | UMLS | 0 | 0 | 1 | 1 |  | pH=4. | 54681 | NCBI | 1 | 0 | 1 | 2 |
| amyloid precursor protein | C0085151 | UMLS | 0 | 0 | 1 | 1 |  | VPS35 | 55737 | NCBI | 0 | 0 | 2 | 2 |
| synaptotagmins | C0084697 | UMLS | 1 | 0 | 0 | 1 |  | DJ-1 | 57320 | NCBI | 1 | 0 | 1 | 2 |
| signal-regulated kinase 1/ | C0082529 | UMLS | 1 | 0 | 0 | 1 |  | GST | 133482 | NCBI | 1 | 1 | 0 | 2 |
| dopamine D3 receptor | C0082341 | UMLS | 0 | 0 | 1 | 1 |  | NeuN | 146713 | NCBI | 0 | 1 | 1 | 2 |
| nuclear factor-kappaB | C0079904 | UMLS | 0 | 0 | 1 | 1 |  | tagging | 404663 | NCBI | 0 | 0 | 2 | 2 |
| ubiquitin protein ligases | C0077678 | UMLS | 0 | 1 | 0 | 1 |  | miRNA-433 | 574034 | NCBI | 1 | 1 | 0 | 2 |
| silk | C0074529 | UMLS | 1 | 0 | 0 | 1 |  | PS-129 | 2843875 | NCBI | 0 | 1 | 1 | 2 |
| senktide | C0074331 | UMLS | 1 | 0 | 0 | 1 |  | SNARE | 1E+08 | NCBI | 0 | 1 | 1 | 2 |
| AMPA receptor regulatory proteins | C0072899 | UMLS | 0 | 1 | 0 | 1 |  | alanine | C0001898 | UMLS | 0 | 1 | 1 | 2 |
| Neurokinin B/NK3 receptors | C0068603 | UMLS | 1 | 0 | 0 | 1 |  | mAb | C0003250 | UMLS | 0 | 0 | 2 | 2 |
| L-DOPA-methylester | C0064871 | UMLS | 0 | 1 | 0 | 1 |  | calmodulin | C0006772 | UMLS | 1 | 0 | 1 | 2 |
| kynurenine hydroxylase | C0064449 | UMLS | 0 | 1 | 0 | 1 |  | clathrin | C0008905 | UMLS | 1 | 0 | 1 | 2 |
| immunoglobulin-heavy-chain-binding protein | C0063423 | UMLS | 0 | 0 | 1 | 1 |  | caspase | C0010656 | UMLS | 1 | 0 | 1 | 2 |
| histamine H3 receptor | C0062739 | UMLS | 1 | 0 | 0 | 1 |  | tyrosinase | C0012524 | UMLS | 1 | 1 | 0 | 2 |
| glutamate transporter | C0061467 | UMLS | 0 | 0 | 1 | 1 |  | glutamine | C0017797 | UMLS | 1 | 0 | 1 | 2 |
| cysteinyl-glycine | C0056886 | UMLS | 0 | 0 | 1 | 1 |  | GTPase | C0018296 | UMLS | 1 | 1 | 0 | 2 |
| chloride channel | C0055363 | UMLS | 1 | 0 | 0 | 1 |  | histidine | C0019602 | UMLS | 1 | 0 | 1 | 2 |
| cannabinoid CB(1) receptors | C0054594 | UMLS | 0 | 0 | 1 | 1 |  | EC 1.6.5.3 | C0020289 | UMLS | 0 | 0 | 2 | 2 |
| calpain inhibitors | C0054533 | UMLS | 0 | 1 | 0 | 1 |  | ligases | C0023689 | UMLS | 1 | 0 | 1 | 2 |
| calcineurin | C0054450 | UMLS | 0 | 0 | 1 | 1 |  | neural protein | C0027759 | UMLS | 1 | 0 | 1 | 2 |
| arginine methyl ester | C0052335 | UMLS | 0 | 1 | 0 | 1 |  | proline | C0033382 | UMLS | 1 | 0 | 1 | 2 |
| 6-hydroxydopa | C0049608 | UMLS | 0 | 1 | 0 | 1 |  | protein kinase | C0033640 | UMLS | 0 | 0 | 2 | 2 |
| alpha-aminoisobutyric acid | C0045888 | UMLS | 0 | 1 | 0 | 1 |  | serum protein | C0036825 | UMLS | 1 | 1 | 0 | 2 |
| cresolase | C0041484 | UMLS | 0 | 1 | 0 | 1 |  | transforming growth factor beta | C0040690 | UMLS | 0 | 1 | 1 | 2 |
| transferase | C0040676 | UMLS | 0 | 1 | 0 | 1 |  | tubulin | C0041348 | UMLS | 0 | 0 | 2 | 2 |
| synaptic D2 receptors | C0039064 | UMLS | 0 | 1 | 0 | 1 |  | Tumor Necrosis Factor | C0041368 | UMLS | 1 | 1 | 0 | 2 |
| superoxide dismutase | C0038838 | UMLS | 1 | 0 | 0 | 1 |  | vasoactive intestinal peptide | C0042395 | UMLS | 0 | 1 | 1 | 2 |
| S-adenosylmethionine | C0036002 | UMLS | 0 | 1 | 0 | 1 |  | calcineurin | C0054450 | UMLS | 0 | 1 | 1 | 2 |
| serotonin receptors | C0034838 | UMLS | 1 | 0 | 0 | 1 |  | calretinin | C0054544 | UMLS | 1 | 0 | 1 | 2 |
| neurotransmitter receptor | C0034828 | UMLS | 0 | 1 | 0 | 1 |  | deoxy | C0069851 | UMLS | 1 | 0 | 1 | 2 |
| GABA(A) receptor | C0034807 | UMLS | 1 | 0 | 0 | 1 |  | prolyl oligopeptidase | C0072132 | UMLS | 0 | 1 | 1 | 2 |
| adrenergic receptors | C0034783 | UMLS | 0 | 1 | 0 | 1 |  | protein-serine kinase | C0072402 | UMLS | 1 | 1 | 0 | 2 |
| factor -P | C0033452 | UMLS | 1 | 0 | 0 | 1 |  | ubiquitin protein ligases | C0077678 | UMLS | 2 | 0 | 0 | 2 |
| potassium channels | C0032824 | UMLS | 0 | 1 | 0 | 1 |  | nuclear factor-kappaB | C0079904 | UMLS | 2 | 0 | 0 | 2 |
| blood plasma protein | C0032120 | UMLS | 1 | 0 | 0 | 1 |  | i) altered protein | C0087044 | UMLS | 0 | 2 | 0 | 2 |
| phosphatases | C0031678 | UMLS | 0 | 1 | 0 | 1 |  | nitric oxide synthase | C0132555 | UMLS | 2 | 0 | 0 | 2 |
| PDEs | C0031640 | UMLS | 1 | 0 | 0 | 1 |  | fusion protein | C0162768 | UMLS | 0 | 1 | 1 | 2 |
| reductase | C0030016 | UMLS | 0 | 1 | 0 | 1 |  | global protein | C0178663 | UMLS | 0 | 1 | 1 | 2 |
| Neurokinin B | C0027847 | UMLS | 1 | 0 | 0 | 1 |  | Ferrireductase | C0243839 | UMLS | 0 | 0 | 2 | 2 |
| monoamine oxidase (MAO) B | C0026456 | UMLS | 0 | 1 | 0 | 1 |  | 26 S proteasome | C0286330 | UMLS | 1 | 0 | 1 | 2 |
| monoamine oxidase | C0026454 | UMLS | 0 | 1 | 0 | 1 |  | SNARE | C0300824 | UMLS | 0 | 2 | 0 | 2 |
| Maps | C0026045 | UMLS | 0 | 0 | 1 | 1 |  | polyglutamine | C0384782 | UMLS | 1 | 0 | 1 | 2 |
| methyltransferase | C0025831 | UMLS | 0 | 1 | 0 | 1 |  | DAT | C0596902 | UMLS | 0 | 0 | 2 | 2 |
| lipases | C0023764 | UMLS | 0 | 0 | 1 | 1 |  | synthetic peptides | C0597551 | UMLS | 0 | 2 | 0 | 2 |
| Leu | C0023401 | UMLS | 0 | 1 | 0 | 1 |  | Toll-like receptors | C0670896 | UMLS | 0 | 2 | 0 | 2 |
| kynurenine | C0022818 | UMLS | 0 | 0 | 1 | 1 |  | mitogen-activated protein (MAP) kinase | C0752312 | UMLS | 1 | 1 | 0 | 2 |
| interleukin-1beta | C0021753 | UMLS | 1 | 0 | 0 | 1 |  | gamma-synuclein | C0753208 | UMLS | 1 | 0 | 1 | 2 |
| transmembrane accessory proteins | C0021699 | UMLS | 1 | 0 | 0 | 1 |  | adaptor protein | C1135629 | UMLS | 1 | 1 | 0 | 2 |
| IGF-1- | C0021665 | UMLS | 0 | 1 | 0 | 1 |  | functional component | C1179435 | UMLS | 0 | 1 | 1 | 2 |
| kappa-light-chain | C0021036 | UMLS | 0 | 0 | 1 | 1 |  | candidate genetic | C1332838 | UMLS | 0 | 1 | 1 | 2 |
| histone H4 | C0019648 | UMLS | 1 | 0 | 0 | 1 |  | cytoplasmic protein | C1333198 | UMLS | 1 | 1 | 0 | 2 |
| histone H3 | C0019647 | UMLS | 1 | 0 | 0 | 1 |  | protein fragment | C1335533 | UMLS | 0 | 1 | 1 | 2 |
| histidine | C0019602 | UMLS | 1 | 0 | 0 | 1 |  | anti-keratin monoclonal antibodies | C1624602 | UMLS | 0 | 1 | 1 | 2 |
| glutathione peroxidase | C0017822 | UMLS | 1 | 0 | 0 | 1 |  | alpha-Synuclein protein | C2362546 | UMLS | 0 | 0 | 2 | 2 |
| glutamic acid decarboxylase | C0017785 | UMLS | 1 | 0 | 0 | 1 |  | AAV-GAD | 17 | NCBI | 0 | 1 | 0 | 1 |
| glial fibrillary acidic protein | C0017626 | UMLS | 0 | 0 | 1 | 1 |  | PACAP | 116 | NCBI | 0 | 1 | 0 | 1 |
| galactosidase | C0016955 | UMLS | 0 | 0 | 1 | 1 |  | at 2 | 186 | NCBI | 0 | 0 | 1 | 1 |
| fibroin | C0016042 | UMLS | 1 | 0 | 0 | 1 |  | ALAS2 | 212 | NCBI | 1 | 0 | 0 | 1 |
| dihydroxyphenylalanine)- | C0013023 | UMLS | 0 | 0 | 1 | 1 |  | albumin | 213 | NCBI | 0 | 0 | 1 | 1 |
| tyrosinase | C0012524 | UMLS | 1 | 0 | 0 | 1 |  | mis | 268 | NCBI | 0 | 1 | 0 | 1 |
| complexes I-IV | C0010760 | UMLS | 1 | 0 | 0 | 1 |  | ApoA-I | 335 | NCBI | 0 | 0 | 1 | 1 |
| cytochrome c | C0010749 | UMLS | 1 | 0 | 0 | 1 |  | Apolipoprotein B-100 | 338 | NCBI | 0 | 0 | 1 | 1 |
| choline acetyltransferase | C0008407 | UMLS | 0 | 0 | 1 | 1 |  | CHIP | 358 | NCBI | 1 | 0 | 0 | 1 |
| decarboxylase | C0007054 | UMLS | 1 | 0 | 0 | 1 |  | 16a | 537 | NCBI | 0 | 1 | 0 | 1 |
| calpain | C0006784 | UMLS | 0 | 1 | 0 | 1 |  | ATR | 545 | NCBI | 0 | 1 | 0 | 1 |
| calmodulin kinase | C0006776 | UMLS | 0 | 0 | 1 | 1 |  | Axl | 558 | NCBI | 0 | 1 | 0 | 1 |
| adenosine receptor | C0001471 | UMLS | 0 | 0 | 1 | 1 |  | beta2-microglobulin | 567 | NCBI | 1 | 0 | 0 | 1 |
| MK-0657 | 1476758 | NCBI | 0 | 0 | 1 | 1 |  | Bcl-2 | 596 | NCBI | 1 | 0 | 0 | 1 |
| LB-100 | 1154026 | NCBI | 0 | 1 | 0 | 1 |  | brain-derived neurotrophic factor | 627 | NCBI | 0 | 0 | 1 | 1 |
| RP-18 | 388552 | NCBI | 0 | 0 | 1 | 1 |  | BLVRB | 645 | NCBI | 1 | 0 | 0 | 1 |
| Fos | 314322 | NCBI | 0 | 1 | 0 | 1 |  | CA3 | 761 | NCBI | 0 | 0 | 1 | 1 |
| NeuN | 287847 | NCBI | 0 | 1 | 0 | 1 |  | Cav | 763 | NCBI | 0 | 0 | 1 | 1 |
| ANKK1 genes | 255239 | NCBI | 0 | 1 | 0 | 1 |  | Calbindin 1 | 793 | NCBI | 1 | 0 | 0 | 1 |
| Rabphilin 3A | 171039 | NCBI | 0 | 0 | 1 | 1 |  | CaMKII | 818 | NCBI | 0 | 1 | 0 | 1 |
| SANS | 124590 | NCBI | 0 | 0 | 1 | 1 |  | caspase 9 | 842 | NCBI | 0 | 0 | 1 | 1 |
| JNK | 116554 | NCBI | 0 | 1 | 0 | 1 |  | catalase | 847 | NCBI | 0 | 1 | 0 | 1 |
| BET | 92737 | NCBI | 0 | 0 | 1 | 1 |  | CCT | 907 | NCBI | 0 | 0 | 1 | 1 |
| gamma-GTP | 92170 | NCBI | 0 | 0 | 1 | 1 |  | CD14 | 929 | NCBI | 1 | 0 | 0 | 1 |
| DARPP-32 | 84152 | NCBI | 0 | 0 | 1 | 1 |  | CD68 | 968 | NCBI | 1 | 0 | 0 | 1 |
| mix | 83881 | NCBI | 0 | 1 | 0 | 1 |  | Cdc5 | 988 | NCBI | 0 | 1 | 0 | 1 |
| c-Src | 83805 | NCBI | 0 | 0 | 1 | 1 |  | Cdc42 effector proteins | 998 | NCBI | 1 | 0 | 0 | 1 |
| norepinephrine transporter | 83511 | NCBI | 1 | 0 | 0 | 1 |  | P15 | 1030 | NCBI | 0 | 1 | 0 | 1 |
| RICTOR | 78757 | NCBI | 0 | 0 | 1 | 1 |  | ChAT | 1103 | NCBI | 0 | 0 | 1 | 1 |
| PINK1 | 68943 | NCBI | 0 | 1 | 0 | 1 |  | clusterin | 1191 | NCBI | 0 | 0 | 1 | 1 |
| PINK1 | 65018 | NCBI | 0 | 1 | 0 | 1 |  | PLK3 | 1263 | NCBI | 0 | 0 | 1 | 1 |
| Ott | 64783 | NCBI | 1 | 0 | 0 | 1 |  | TAT-PAX6 protein | 1385 | NCBI | 0 | 1 | 0 | 1 |
| Syt 4 | 64440 | NCBI | 1 | 0 | 0 | 1 |  | p38 MAP kinase | 1398 | NCBI | 1 | 0 | 0 | 1 |
| Per2 | 63840 | NCBI | 0 | 0 | 1 | 1 |  | Drp1 | 1400 | NCBI | 0 | 0 | 1 | 1 |
| interleukin-1 receptor antagonist | 60582 | NCBI | 1 | 0 | 0 | 1 |  | CAS | 1434 | NCBI | 0 | 0 | 1 | 1 |
| Syt 10 | 60567 | NCBI | 1 | 0 | 0 | 1 |  | capped | 1483 | NCBI | 0 | 0 | 1 | 1 |
| Ghrelin | 59301 | NCBI | 0 | 1 | 0 | 1 |  | chymotrypsin-like | 1506 | NCBI | 0 | 0 | 1 | 1 |
| Syt 7 | 59267 | NCBI | 1 | 0 | 0 | 1 |  | cathepsin B | 1508 | NCBI | 1 | 0 | 0 | 1 |
| G protein-coupled receptor kinase 6 | 59076 | NCBI | 0 | 1 | 0 | 1 |  | MEP | 1514 | NCBI | 0 | 0 | 1 | 1 |
| RAMP1 | 58965 | NCBI | 1 | 0 | 0 | 1 |  | CHOP | 1649 | NCBI | 0 | 1 | 0 | 1 |
| CB2 | 57302 | NCBI | 0 | 0 | 1 | 1 |  | ATPase | 1769 | NCBI | 1 | 0 | 0 | 1 |
| PDSS)-2 | 57107 | NCBI | 0 | 0 | 1 | 1 |  | dopamine D1 receptor | 1812 | NCBI | 0 | 0 | 1 | 1 |
| muM | 56925 | NCBI | 0 | 1 | 0 | 1 |  | DPI | 1832 | NCBI | 0 | 0 | 1 | 1 |
| GSK3beta | 56637 | NCBI | 0 | 1 | 0 | 1 |  | DYRK1A | 1859 | NCBI | 0 | 1 | 0 | 1 |
| DNAJC12 | 56521 | NCBI | 0 | 0 | 1 | 1 |  | eta | 1909 | NCBI | 0 | 1 | 0 | 1 |
| 3/5/2019 | 54708 | NCBI | 0 | 0 | 1 | 1 |  | ETB | 1910 | NCBI | 0 | 1 | 0 | 1 |
| pH=4. | 54681 | NCBI | 1 | 0 | 0 | 1 |  | EIF4G1 | 1981 | NCBI | 0 | 0 | 1 | 1 |
| Bmal1 | 29657 | NCBI | 0 | 0 | 1 | 1 |  | hub | 1993 | NCBI | 0 | 0 | 1 | 1 |
| PSD-95 | 29495 | NCBI | 0 | 1 | 0 | 1 |  | neuron-specific enolase | 2026 | NCBI | 0 | 1 | 0 | 1 |
| GLT1 | 29482 | NCBI | 0 | 0 | 1 | 1 |  | APAs | 2028 | NCBI | 1 | 0 | 0 | 1 |
| sigma-1 receptor | 29336 | NCBI | 1 | 0 | 0 | 1 |  | endosulfine-alpha | 2029 | NCBI | 0 | 0 | 1 | 1 |
| GCH1 | 29244 | NCBI | 0 | 1 | 0 | 1 |  | HEK | 2042 | NCBI | 1 | 0 | 0 | 1 |
| dopamine D-3 receptor | 29238 | NCBI | 1 | 0 | 0 | 1 |  | ERK)1/2 | 2048 | NCBI | 1 | 0 | 0 | 1 |
| preprodynorphin | 29190 | NCBI | 1 | 0 | 0 | 1 |  | eps-8 | 2059 | NCBI | 1 | 0 | 0 | 1 |
| NK(1 | 27087 | NCBI | 1 | 0 | 0 | 1 |  | inositol-requiring enzyme 1 | 2081 | NCBI | 0 | 0 | 1 | 1 |
| LAT | 27040 | NCBI | 0 | 1 | 0 | 1 |  | heart-fatty acid binding protein | 2170 | NCBI | 0 | 0 | 1 | 1 |
| G protein-coupled Receptor Kinase 6 | 26385 | NCBI | 0 | 1 | 0 | 1 |  | FcgammaRI | 2209 | NCBI | 0 | 0 | 1 | 1 |
| wise | 25928 | NCBI | 0 | 0 | 1 | 1 |  | FcgammaRIIB | 2213 | NCBI | 0 | 0 | 1 | 1 |
| MAO B | 25750 | NCBI | 0 | 0 | 1 | 1 |  | FECH | 2235 | NCBI | 1 | 0 | 0 | 1 |
| p65 | 25716 | NCBI | 1 | 0 | 0 | 1 |  | fibroblast growth factor 2 | 2247 | NCBI | 1 | 0 | 0 | 1 |
| Ass1 | 25698 | NCBI | 0 | 0 | 1 | 1 |  | FOXO3a | 2309 | NCBI | 0 | 0 | 1 | 1 |
| Shp-2 | 25622 | NCBI | 0 | 0 | 1 | 1 |  | mammalian target of rapamycin | 2475 | NCBI | 0 | 0 | 1 | 1 |
| Serotonin transporter | 25553 | NCBI | 0 | 1 | 0 | 1 |  | Hsc | 2523 | NCBI | 0 | 1 | 0 | 1 |
| SBP | 25540 | NCBI | 0 | 0 | 1 | 1 |  | GATA-1 | 2623 | NCBI | 1 | 0 | 0 | 1 |
| CB1 | 25248 | NCBI | 0 | 0 | 1 | 1 |  | GATA-2 | 2624 | NCBI | 1 | 0 | 0 | 1 |
| tyrosine hydroxylase | 25085 | NCBI | 1 | 0 | 0 | 1 |  | TOF | 2626 | NCBI | 1 | 0 | 0 | 1 |
| alpha(2A) adrenoceptor | 25083 | NCBI | 0 | 1 | 0 | 1 |  | GDNF | 2668 | NCBI | 0 | 1 | 0 | 1 |
| serotonin receptor 1B | 25075 | NCBI | 1 | 0 | 0 | 1 |  | GFAP | 2670 | NCBI | 0 | 1 | 0 | 1 |
| Pleiotrophin | 24924 | NCBI | 1 | 0 | 0 | 1 |  | GCL | 2729 | NCBI | 0 | 1 | 0 | 1 |
| NK(3) receptor | 24808 | NCBI | 1 | 0 | 0 | 1 |  | MER | 2852 | NCBI | 0 | 1 | 0 | 1 |
| Syt 2 | 24805 | NCBI | 1 | 0 | 0 | 1 |  | NR2B | 2904 | NCBI | 0 | 0 | 1 | 1 |
| neuronal NOS | 24598 | NCBI | 0 | 1 | 0 | 1 |  | GSK3beta | 2932 | NCBI | 1 | 0 | 0 | 1 |
| interleukin-1beta | 24494 | NCBI | 1 | 0 | 0 | 1 |  | H2AX | 3014 | NCBI | 0 | 0 | 1 | 1 |
| NR1 | 24408 | NCBI | 0 | 0 | 1 | 1 |  | sPD) | 3239 | NCBI | 0 | 0 | 1 | 1 |
| zif-268 | 24330 | NCBI | 0 | 0 | 1 | 1 |  | GroES | 3336 | NCBI | 0 | 0 | 1 | 1 |
| DRD2 | 24318 | NCBI | 0 | 1 | 0 | 1 |  | serotonin receptor 3A | 3359 | NCBI | 0 | 0 | 1 | 1 |
| DRD1 | 24316 | NCBI | 0 | 1 | 0 | 1 |  | serotonin receptor 4 | 3360 | NCBI | 0 | 0 | 1 | 1 |
| AADC | 24311 | NCBI | 0 | 0 | 1 | 1 |  | IAPP | 3375 | NCBI | 0 | 1 | 0 | 1 |
| DBP | 24309 | NCBI | 0 | 0 | 1 | 1 |  | IDP | 3417 | NCBI | 0 | 1 | 0 | 1 |
| COMT | 24267 | NCBI | 0 | 0 | 1 | 1 |  | IDUA | 3425 | NCBI | 0 | 0 | 1 | 1 |
| BDNF | 24225 | NCBI | 0 | 0 | 1 | 1 |  | IFN | 3439 | NCBI | 1 | 0 | 0 | 1 |
| Bcl-2 | 24224 | NCBI | 1 | 0 | 0 | 1 |  | ALS | 3483 | NCBI | 1 | 0 | 0 | 1 |
| Syn | 23336 | NCBI | 0 | 1 | 0 | 1 |  | IL-2 | 3558 | NCBI | 0 | 0 | 1 | 1 |
| Arc | 23237 | NCBI | 0 | 0 | 1 | 1 |  | IL-8 | 3576 | NCBI | 0 | 0 | 1 | 1 |
| 5-HT2A receptor | 22954 | NCBI | 1 | 0 | 0 | 1 |  | CXCL10 | 3627 | NCBI | 0 | 1 | 0 | 1 |
| Rabphilin 3A | 22895 | NCBI | 0 | 0 | 1 | 1 |  | CD11b | 3684 | NCBI | 0 | 0 | 1 | 1 |
| Tyrosine hydroxylase | 21823 | NCBI | 0 | 1 | 0 | 1 |  | c-Jun-N-terminal kinase | 3725 | NCBI | 1 | 0 | 0 | 1 |
| PHF-1 | 21652 | NCBI | 0 | 1 | 0 | 1 |  | Kv4.3 potassium channels | 3746 | NCBI | 0 | 0 | 1 | 1 |
| p11 | 20194 | NCBI | 1 | 0 | 0 | 1 |  | lysosome-associated membrane protein 2 | 3920 | NCBI | 0 | 0 | 1 | 1 |
| 6-pyruvoyl-tetrahydropterin synthase | 19286 | NCBI | 1 | 0 | 0 | 1 |  | TLC | 3933 | NCBI | 0 | 0 | 1 | 1 |
| Pitx3 | 18742 | NCBI | 0 | 1 | 0 | 1 |  | p25 | 3934 | NCBI | 0 | 0 | 1 | 1 |
| PEnk | 18619 | NCBI | 1 | 0 | 0 | 1 |  | LRP | 3949 | NCBI | 0 | 0 | 1 | 1 |
| 5-HT1B receptor | 15551 | NCBI | 1 | 0 | 0 | 1 |  | GAL4 | 3960 | NCBI | 0 | 1 | 0 | 1 |
| NR2B | 14812 | NCBI | 0 | 0 | 1 | 1 |  | LTB | 4050 | NCBI | 0 | 0 | 1 | 1 |
| mGluR2 | 14800 | NCBI | 1 | 0 | 0 | 1 |  | monoamine oxidase type B | 4129 | NCBI | 0 | 0 | 1 | 1 |
| Galpha(olf) | 14680 | NCBI | 0 | 0 | 1 | 1 |  | Mhc2ta | 4261 | NCBI | 0 | 0 | 1 | 1 |
| Gad2 | 14417 | NCBI | 0 | 0 | 1 | 1 |  | ataxin-3 | 4287 | NCBI | 0 | 0 | 1 | 1 |
| Fyn | 14360 | NCBI | 0 | 0 | 1 | 1 |  | Mmp2 | 4313 | NCBI | 0 | 0 | 1 | 1 |
| FosB | 14282 | NCBI | 0 | 0 | 1 | 1 |  | MPP( | 4359 | NCBI | 1 | 0 | 0 | 1 |
| dopamine D3 receptor | 13490 | NCBI | 0 | 0 | 1 | 1 |  | MS) | 4397 | NCBI | 1 | 0 | 0 | 1 |
| CK2 | 13000 | NCBI | 0 | 0 | 1 | 1 |  | Metallothionein-III | 4504 | NCBI | 0 | 0 | 1 | 1 |
| caspase-3 | 12367 | NCBI | 0 | 1 | 0 | 1 |  | Cox-2 | 4513 | NCBI | 1 | 0 | 0 | 1 |
| catalase | 12359 | NCBI | 0 | 0 | 1 | 1 |  | c-Myc | 4609 | NCBI | 0 | 0 | 1 | 1 |
| AKT | 11651 | NCBI | 0 | 1 | 0 | 1 |  | p95 | 4683 | NCBI | 1 | 0 | 0 | 1 |
| Receptor-activity modifying protein 1 | 10267 | NCBI | 1 | 0 | 0 | 1 |  | NCL | 4691 | NCBI | 0 | 0 | 1 | 1 |
| clock | 9575 | NCBI | 0 | 0 | 1 | 1 |  | NDUFV2 | 4729 | NCBI | 1 | 0 | 0 | 1 |
| Homer | 9456 | NCBI | 0 | 0 | 1 | 1 |  | Nedd4- | 4734 | NCBI | 0 | 0 | 1 | 1 |
| PERK | 9451 | NCBI | 0 | 0 | 1 | 1 |  | beta2 | 4760 | NCBI | 1 | 0 | 0 | 1 |
| grid | 9402 | NCBI | 0 | 1 | 0 | 1 |  | nuclear factor erythroid 2-related factor 2 | 4780 | NCBI | 0 | 1 | 0 | 1 |
| NOP | 8996 | NCBI | 1 | 0 | 0 | 1 |  | NGF | 4803 | NCBI | 0 | 1 | 0 | 1 |
| wave | 8936 | NCBI | 0 | 1 | 0 | 1 |  | TrkB | 4915 | NCBI | 0 | 0 | 1 | 1 |
| SPL | 8879 | NCBI | 0 | 1 | 0 | 1 |  | plasminogen activator inhibitor-1 | 5054 | NCBI | 0 | 1 | 0 | 1 |
| NAc | 7504 | NCBI | 0 | 0 | 1 | 1 |  | PHDs | 5132 | NCBI | 1 | 0 | 0 | 1 |
| XBP-1 | 7494 | NCBI | 0 | 0 | 1 | 1 |  | PDs | 5172 | NCBI | 0 | 0 | 1 | 1 |
| UCHL1 | 7345 | NCBI | 0 | 0 | 1 | 1 |  | plasmin | 5340 | NCBI | 0 | 1 | 0 | 1 |
| ubiquitin C | 7316 | NCBI | 0 | 0 | 1 | 1 |  | inhibitors 1 | 5502 | NCBI | 0 | 0 | 1 | 1 |
| MSA | 7173 | NCBI | 0 | 0 | 1 | 1 |  | AMPK | 5562 | NCBI | 0 | 0 | 1 | 1 |
| TNF-alpha | 7124 | NCBI | 0 | 0 | 1 | 1 |  | PKCdelta | 5580 | NCBI | 0 | 0 | 1 | 1 |
| top | 7064 | NCBI | 0 | 0 | 1 | 1 |  | ERK1 | 5595 | NCBI | 0 | 0 | 1 | 1 |
| Tat protein | 6898 | NCBI | 1 | 0 | 0 | 1 |  | PTEN | 5728 | NCBI | 0 | 0 | 1 | 1 |
| Src | 6714 | NCBI | 0 | 0 | 1 | 1 |  | lipocalin-type prostaglandin D synthase | 5730 | NCBI | 0 | 1 | 0 | 1 |
| SMD -1 | 6632 | NCBI | 0 | 1 | 0 | 1 |  | focal adhesion kinase | 5747 | NCBI | 0 | 1 | 0 | 1 |
| SNAP-25 | 6616 | NCBI | 0 | 0 | 1 | 1 |  | protein tyrosine phosphatase 1B | 5770 | NCBI | 0 | 1 | 0 | 1 |
| serotonin transporter | 6532 | NCBI | 0 | 1 | 0 | 1 |  | Rab1 | 5861 | NCBI | 0 | 0 | 1 | 1 |
| norepinephrine transporter | 6530 | NCBI | 1 | 0 | 0 | 1 |  | Rab5 | 5868 | NCBI | 0 | 1 | 0 | 1 |
| GLT-1 | 6506 | NCBI | 0 | 0 | 1 | 1 |  | Rab27a | 5873 | NCBI | 0 | 0 | 1 | 1 |
| type | 6445 | NCBI | 0 | 0 | 1 | 1 |  | ret | 5979 | NCBI | 0 | 1 | 0 | 1 |
| SAH | 6296 | NCBI | 0 | 1 | 0 | 1 |  | L38 | 6169 | NCBI | 1 | 0 | 0 | 1 |
| p11 | 6281 | NCBI | 1 | 0 | 0 | 1 |  | SCA2 | 6311 | NCBI | 0 | 0 | 1 | 1 |
| Roralpha | 6095 | NCBI | 0 | 0 | 1 | 1 |  | SIAH-2 | 6478 | NCBI | 1 | 0 | 0 | 1 |
| Pleiotrophin | 5764 | NCBI | 1 | 0 | 0 | 1 |  | glucose transporters isoform 1 | 6513 | NCBI | 0 | 0 | 1 | 1 |
| delta psi | 5694 | NCBI | 0 | 1 | 0 | 1 |  | glucose transporter 4 | 6517 | NCBI | 0 | 0 | 1 | 1 |
| PSG | 5673 | NCBI | 0 | 0 | 1 | 1 |  | SGLT1 | 6523 | NCBI | 0 | 0 | 1 | 1 |
| MEK | 5609 | NCBI | 1 | 0 | 0 | 1 |  | Nramp | 6556 | NCBI | 0 | 0 | 1 | 1 |
| JNKs | 5599 | NCBI | 0 | 1 | 0 | 1 |  | SNL-4 | 6624 | NCBI | 1 | 0 | 0 | 1 |
| PP2A | 5524 | NCBI | 0 | 1 | 0 | 1 |  | endonuclease PstI | 6690 | NCBI | 0 | 0 | 1 | 1 |
| PHDs | 5132 | NCBI | 0 | 0 | 1 | 1 |  | SPR | 6697 | NCBI | 0 | 0 | 1 | 1 |
| PAM | 5066 | NCBI | 0 | 1 | 0 | 1 |  | SPTBN1 | 6711 | NCBI | 0 | 1 | 0 | 1 |
| pnp | 4860 | NCBI | 0 | 0 | 1 | 1 |  | Ssa1p | 6737 | NCBI | 0 | 1 | 0 | 1 |
| nNOS | 4842 | NCBI | 0 | 0 | 1 | 1 |  | Hsp70-interacting protein | 6767 | NCBI | 1 | 0 | 0 | 1 |
| NF-kappa B | 4790 | NCBI | 0 | 1 | 0 | 1 |  | VAMP2 | 6844 | NCBI | 0 | 0 | 1 | 1 |
| B12 | 4709 | NCBI | 1 | 0 | 0 | 1 |  | synaptophysin | 6855 | NCBI | 1 | 0 | 0 | 1 |
| ND2 | 4536 | NCBI | 0 | 0 | 1 | 1 |  | transcription factor GATA-1 | 6927 | NCBI | 1 | 0 | 0 | 1 |
| ND1 | 4535 | NCBI | 0 | 0 | 1 | 1 |  | Tctex1 | 6993 | NCBI | 0 | 0 | 1 | 1 |
| PSP | 4477 | NCBI | 0 | 0 | 1 | 1 |  | TGF-beta1 | 7040 | NCBI | 0 | 0 | 1 | 1 |
| LTF | 4057 | NCBI | 0 | 0 | 1 | 1 |  | Tissue transglutaminase | 7052 | NCBI | 1 | 0 | 0 | 1 |
| DBS's | 4036 | NCBI | 0 | 1 | 0 | 1 |  | A20 | 7128 | NCBI | 1 | 0 | 0 | 1 |
| DDD | 3852 | NCBI | 0 | 1 | 0 | 1 |  | TNFalpha receptor 1 | 7132 | NCBI | 0 | 1 | 0 | 1 |
| N22-P40 | 3578 | NCBI | 0 | 0 | 1 | 1 |  | TNT | 7138 | NCBI | 0 | 0 | 1 | 1 |
| IL-1beta | 3553 | NCBI | 0 | 0 | 1 | 1 |  | Grp94 | 7184 | NCBI | 0 | 0 | 1 | 1 |
| IFN | 3439 | NCBI | 0 | 0 | 1 | 1 |  | Trp4 | 7223 | NCBI | 1 | 0 | 0 | 1 |
| HTR2A | 3356 | NCBI | 0 | 1 | 0 | 1 |  | THP-1 | 7369 | NCBI | 1 | 0 | 0 | 1 |
| 3-hydroxy-3-methylglutaryl-CoA reductase | 3156 | NCBI | 1 | 0 | 0 | 1 |  | VIP | 7432 | NCBI | 0 | 0 | 1 | 1 |
| mGlu(4) | 2914 | NCBI | 0 | 1 | 0 | 1 |  | gamma2 | 7453 | NCBI | 0 | 1 | 0 | 1 |
| NR2B | 2904 | NCBI | 0 | 0 | 1 | 1 |  | XBP-1 | 7494 | NCBI | 0 | 0 | 1 | 1 |
| GluR1 | 2890 | NCBI | 0 | 1 | 0 | 1 |  | 14-3-3 epsilon | 7531 | NCBI | 0 | 1 | 0 | 1 |
| GRK5 | 2869 | NCBI | 1 | 0 | 0 | 1 |  | TFEB | 7942 | NCBI | 0 | 0 | 1 | 1 |
| Gnal gene | 2774 | NCBI | 0 | 0 | 1 | 1 |  | USP9X | 8239 | NCBI | 0 | 1 | 0 | 1 |
| glucocerebrosidase | 2629 | NCBI | 0 | 1 | 0 | 1 |  | held | 8289 | NCBI | 0 | 0 | 1 | 1 |
| Nrf2 | 2551 | NCBI | 0 | 1 | 0 | 1 |  | ULK1 | 8408 | NCBI | 0 | 0 | 1 | 1 |
| mammalian target of rapamycin | 2475 | NCBI | 0 | 0 | 1 | 1 |  | CMAP | 8530 | NCBI | 0 | 0 | 1 | 1 |
| Fos protein | 2353 | NCBI | 0 | 0 | 1 | 1 |  | CADPS | 8618 | NCBI | 0 | 0 | 1 | 1 |
| ERK 1/2 | 2048 | NCBI | 0 | 0 | 1 | 1 |  | flip- | 8837 | NCBI | 0 | 1 | 0 | 1 |
| beta-adrenergic receptor | 1991 | NCBI | 0 | 1 | 0 | 1 |  | sequestosome 1 | 8878 | NCBI | 0 | 1 | 0 | 1 |
| dopamine D(4) receptor | 1815 | NCBI | 0 | 1 | 0 | 1 |  | GLA | 9027 | NCBI | 0 | 0 | 1 | 1 |
| DRD3 | 1814 | NCBI | 0 | 1 | 0 | 1 |  | ENS | 9053 | NCBI | 1 | 0 | 0 | 1 |
| PSD-95 | 1742 | NCBI | 0 | 1 | 0 | 1 |  | ubiquitin-conjugating enzyme UbcH8 | 9246 | NCBI | 1 | 0 | 0 | 1 |
| CHOP | 1649 | NCBI | 0 | 0 | 1 | 1 |  | Klotho | 9365 | NCBI | 0 | 0 | 1 | 1 |
| CTX | 1593 | NCBI | 0 | 0 | 1 | 1 |  | PERK | 9451 | NCBI | 0 | 1 | 0 | 1 |
| CPu | 1361 | NCBI | 0 | 1 | 0 | 1 |  | bis[ | 9531 | NCBI | 0 | 0 | 1 | 1 |
| CPA | 1357 | NCBI | 1 | 0 | 0 | 1 |  | syn(1 | 9623 | NCBI | 0 | 1 | 0 | 1 |
| VIII | 1351 | NCBI | 1 | 0 | 0 | 1 |  | the SAM | 9652 | NCBI | 0 | 0 | 1 | 1 |
| MED) | 1297 | NCBI | 1 | 0 | 0 | 1 |  | epsinR | 9685 | NCBI | 1 | 0 | 0 | 1 |
| cannabinoid receptor 1 | 1268 | NCBI | 0 | 0 | 1 | 1 |  | TRAP1 | 10131 | NCBI | 0 | 1 | 0 | 1 |
| BCH | 1124 | NCBI | 1 | 0 | 0 | 1 |  | CHIP | 10273 | NCBI | 1 | 0 | 0 | 1 |
| Cbl-interacting protein | 867 | NCBI | 1 | 0 | 0 | 1 |  | beta5 | 10382 | NCBI | 1 | 0 | 0 | 1 |
| cAMP response element-binding protein | 820 | NCBI | 0 | 0 | 1 | 1 |  | v-SNARE | 10490 | NCBI | 0 | 0 | 1 | 1 |
| CaMKII | 818 | NCBI | 1 | 0 | 0 | 1 |  | Txnip | 10628 | NCBI | 0 | 0 | 1 | 1 |
| CAMK2A | 815 | NCBI | 0 | 0 | 1 | 1 |  | PGC-1alpha | 10891 | NCBI | 0 | 1 | 0 | 1 |
| brain-derived neurotrophic factor | 627 | NCBI | 0 | 0 | 1 | 1 |  | small acidic protein | 10944 | NCBI | 1 | 0 | 0 | 1 |
| ADH | 551 | NCBI | 0 | 1 | 0 | 1 |  | RER1 | 11079 | NCBI | 0 | 0 | 1 | 1 |
| ATF4 | 468 | NCBI | 0 | 0 | 1 | 1 |  | apolipoprotein E | 11816 | NCBI | 1 | 0 | 0 | 1 |
| Asp | 434 | NCBI | 0 | 1 | 0 | 1 |  | CA2 | 12349 | NCBI | 0 | 0 | 1 | 1 |
| beta-arrestin2 | 409 | NCBI | 0 | 0 | 1 | 1 |  | CD4 | 12504 | NCBI | 0 | 1 | 0 | 1 |
| Amyloid-beta (Abeta) peptide | 351 | NCBI | 0 | 0 | 1 | 1 |  | choline acetyltransferase | 12647 | NCBI | 0 | 1 | 0 | 1 |
| Aldose reductase | 231 | NCBI | 0 | 0 | 1 | 1 |  | CREB | 12912 | NCBI | 0 | 1 | 0 | 1 |
| Akt | 207 | NCBI | 0 | 0 | 1 | 1 |  | corticotropin releasing factor | 12918 | NCBI | 1 | 0 | 0 | 1 |
| GRK3 | 157 | NCBI | 1 | 0 | 0 | 1 |  | alpha B-crystallin | 12955 | NCBI | 1 | 0 | 0 | 1 |
| GRK2 | 156 | NCBI | 1 | 0 | 0 | 1 |  | DAT | 13162 | NCBI | 1 | 0 | 0 | 1 |
| PARP | 142 | NCBI | 1 | 0 | 0 | 1 |  | Fos | 14281 | NCBI | 0 | 1 | 0 | 1 |
|  |  |  |  |  |  |  |  | GBA | 14466 | NCBI | 0 | 0 | 1 | 1 |
|  |  |  |  |  |  |  |  | glial cell line-derived neurotrophic factor | 14573 | NCBI | 0 | 1 | 0 | 1 |
|  |  |  |  |  |  |  |  | mGluR5 | 14805 | NCBI | 0 | 0 | 1 | 1 |
|  |  |  |  |  |  |  |  | NR2B | 14812 | NCBI | 0 | 0 | 1 | 1 |
|  |  |  |  |  |  |  |  | huntingtin | 15194 | NCBI | 1 | 0 | 0 | 1 |
|  |  |  |  |  |  |  |  | heme oxygenase-1 | 15368 | NCBI | 0 | 1 | 0 | 1 |
|  |  |  |  |  |  |  |  | HSF1 | 15499 | NCBI | 0 | 1 | 0 | 1 |
|  |  |  |  |  |  |  |  | SERT | 15567 | NCBI | 0 | 1 | 0 | 1 |
|  |  |  |  |  |  |  |  | IFNgamma | 15978 | NCBI | 0 | 0 | 1 | 1 |
|  |  |  |  |  |  |  |  | interleukin-1alpha | 16175 | NCBI | 0 | 0 | 1 | 1 |
|  |  |  |  |  |  |  |  | interleukin-6 | 16193 | NCBI | 0 | 1 | 0 | 1 |
|  |  |  |  |  |  |  |  | mBP | 17196 | NCBI | 0 | 0 | 1 | 1 |
|  |  |  |  |  |  |  |  | microtubule-associated protein tau | 17762 | NCBI | 0 | 0 | 1 | 1 |
|  |  |  |  |  |  |  |  | NQO1 | 18104 | NCBI | 0 | 1 | 0 | 1 |
|  |  |  |  |  |  |  |  | Nramp1 | 18173 | NCBI | 0 | 0 | 1 | 1 |
|  |  |  |  |  |  |  |  | PDE1A | 18573 | NCBI | 0 | 0 | 1 | 1 |
|  |  |  |  |  |  |  |  | PDE1C | 18575 | NCBI | 0 | 0 | 1 | 1 |
|  |  |  |  |  |  |  |  | DARPP-32 | 19049 | NCBI | 1 | 0 | 0 | 1 |
|  |  |  |  |  |  |  |  | PREP | 19072 | NCBI | 0 | 1 | 0 | 1 |
|  |  |  |  |  |  |  |  | Ret | 19713 | NCBI | 0 | 1 | 0 | 1 |
|  |  |  |  |  |  |  |  | Snap25 | 20614 | NCBI | 0 | 1 | 0 | 1 |
|  |  |  |  |  |  |  |  | STC-1 | 20855 | NCBI | 0 | 0 | 1 | 1 |
|  |  |  |  |  |  |  |  | Synaptophysin | 20977 | NCBI | 0 | 0 | 1 | 1 |
|  |  |  |  |  |  |  |  | TLR4 | 21898 | NCBI | 0 | 1 | 0 | 1 |
|  |  |  |  |  |  |  |  | mTOR | 21977 | NCBI | 0 | 0 | 1 | 1 |
|  |  |  |  |  |  |  |  | Grp94 | 22027 | NCBI | 0 | 0 | 1 | 1 |
|  |  |  |  |  |  |  |  | TRPC3 | 22065 | NCBI | 0 | 0 | 1 | 1 |
|  |  |  |  |  |  |  |  | SUMO-1 | 22218 | NCBI | 0 | 0 | 1 | 1 |
|  |  |  |  |  |  |  |  | UCH-L1 | 22223 | NCBI | 1 | 0 | 0 | 1 |
|  |  |  |  |  |  |  |  | 14-3-3 epsilon | 22627 | NCBI | 0 | 1 | 0 | 1 |
|  |  |  |  |  |  |  |  | ATF6 | 22926 | NCBI | 0 | 1 | 0 | 1 |
|  |  |  |  |  |  |  |  | 5-HT2A receptor | 22954 | NCBI | 0 | 0 | 1 | 1 |
|  |  |  |  |  |  |  |  | GGA3 | 23163 | NCBI | 0 | 0 | 1 | 1 |
|  |  |  |  |  |  |  |  | PLCbeta1 | 23236 | NCBI | 0 | 0 | 1 | 1 |
|  |  |  |  |  |  |  |  | SIRT3 | 23410 | NCBI | 0 | 0 | 1 | 1 |
|  |  |  |  |  |  |  |  | TDP-43 | 23435 | NCBI | 1 | 0 | 0 | 1 |
|  |  |  |  |  |  |  |  | ABCA5 | 23461 | NCBI | 0 | 1 | 0 | 1 |
|  |  |  |  |  |  |  |  | TLR2 | 24088 | NCBI | 0 | 1 | 0 | 1 |
|  |  |  |  |  |  |  |  | glial cell line-derived neurotrophic factor | 25453 | NCBI | 0 | 1 | 0 | 1 |
|  |  |  |  |  |  |  |  | Grp78 | 25617 | NCBI | 0 | 1 | 0 | 1 |
|  |  |  |  |  |  |  |  | D1-4 | 25802 | NCBI | 0 | 0 | 1 | 1 |
|  |  |  |  |  |  |  |  | genesis | 27022 | NCBI | 1 | 0 | 0 | 1 |
|  |  |  |  |  |  |  |  | beta3 | 27319 | NCBI | 1 | 0 | 0 | 1 |
|  |  |  |  |  |  |  |  | HTRA2 | 27429 | NCBI | 1 | 0 | 0 | 1 |
|  |  |  |  |  |  |  |  | A30 | 28937 | NCBI | 0 | 1 | 0 | 1 |
|  |  |  |  |  |  |  |  | BAG3 | 29810 | NCBI | 0 | 0 | 1 | 1 |
|  |  |  |  |  |  |  |  | PTEN-induced kinase 1 | 31607 | NCBI | 0 | 1 | 0 | 1 |
|  |  |  |  |  |  |  |  | troponin T | 32314 | NCBI | 1 | 0 | 0 | 1 |
|  |  |  |  |  |  |  |  | TRAP1 | 35559 | NCBI | 0 | 1 | 0 | 1 |
|  |  |  |  |  |  |  |  | manganese-superoxide dismutase | 36878 | NCBI | 1 | 0 | 0 | 1 |
|  |  |  |  |  |  |  |  | muscle LIM protein at 60A | 37853 | NCBI | 1 | 0 | 0 | 1 |
|  |  |  |  |  |  |  |  | fat body protein 1 | 39566 | NCBI | 1 | 0 | 0 | 1 |
|  |  |  |  |  |  |  |  | parkin | 40336 | NCBI | 0 | 1 | 0 | 1 |
|  |  |  |  |  |  |  |  | hypoxia-inducible factor | 43580 | NCBI | 0 | 0 | 1 | 1 |
|  |  |  |  |  |  |  |  | ATP synthase | 46069 | NCBI | 1 | 0 | 0 | 1 |
|  |  |  |  |  |  |  |  | PARKIN protein | 50873 | NCBI | 0 | 1 | 0 | 1 |
|  |  |  |  |  |  |  |  | retromer-complex protein | 51699 | NCBI | 0 | 0 | 1 | 1 |
|  |  |  |  |  |  |  |  | cytochrome C | 54205 | NCBI | 1 | 0 | 0 | 1 |
|  |  |  |  |  |  |  |  | G41 | 55012 | NCBI | 0 | 0 | 1 | 1 |
|  |  |  |  |  |  |  |  | cryptic | 55997 | NCBI | 1 | 0 | 0 | 1 |
|  |  |  |  |  |  |  |  | septin 6 | 56526 | NCBI | 1 | 0 | 0 | 1 |
|  |  |  |  |  |  |  |  | Kv4.3 | 56543 | NCBI | 0 | 0 | 1 | 1 |
|  |  |  |  |  |  |  |  | vGluT1 | 57030 | NCBI | 0 | 0 | 1 | 1 |
|  |  |  |  |  |  |  |  | arms | 57498 | NCBI | 1 | 0 | 0 | 1 |
|  |  |  |  |  |  |  |  | neutral cholesterol ester hydrolase 1 | 57552 | NCBI | 0 | 0 | 1 | 1 |
|  |  |  |  |  |  |  |  | sirtuin 2 | 64383 | NCBI | 0 | 0 | 1 | 1 |
|  |  |  |  |  |  |  |  | LC3 | 66734 | NCBI | 0 | 0 | 1 | 1 |
|  |  |  |  |  |  |  |  | Atg7 | 74244 | NCBI | 0 | 1 | 0 | 1 |
|  |  |  |  |  |  |  |  | Sideroflexin 3 | 81855 | NCBI | 0 | 0 | 1 | 1 |
|  |  |  |  |  |  |  |  | Mhc2ta | 85483 | NCBI | 0 | 0 | 1 | 1 |
|  |  |  |  |  |  |  |  | eri-1 | 90459 | NCBI | 1 | 0 | 0 | 1 |
|  |  |  |  |  |  |  |  | Soc | 91544 | NCBI | 0 | 0 | 1 | 1 |
|  |  |  |  |  |  |  |  | CADPS2 | 93664 | NCBI | 0 | 0 | 1 | 1 |
|  |  |  |  |  |  |  |  | SIRT1 | 93759 | NCBI | 0 | 1 | 0 | 1 |
|  |  |  |  |  |  |  |  | sideroflexin 3 | 94280 | NCBI | 0 | 0 | 1 | 1 |
|  |  |  |  |  |  |  |  | sequestosome 1 | 113894 | NCBI | 0 | 0 | 1 | 1 |
|  |  |  |  |  |  |  |  | PIKE-L | 116986 | NCBI | 0 | 0 | 1 | 1 |
|  |  |  |  |  |  |  |  | pSH1 | 121665 | NCBI | 1 | 0 | 0 | 1 |
|  |  |  |  |  |  |  |  | beta4 | 128408 | NCBI | 1 | 0 | 0 | 1 |
|  |  |  |  |  |  |  |  | APS 2 | 170685 | NCBI | 1 | 0 | 0 | 1 |
|  |  |  |  |  |  |  |  | TLR 8 | 170744 | NCBI | 0 | 1 | 0 | 1 |
|  |  |  |  |  |  |  |  | ADAMTS19 | 171019 | NCBI | 0 | 0 | 1 | 1 |
|  |  |  |  |  |  |  |  | fractalkine receptor | 171056 | NCBI | 0 | 1 | 0 | 1 |
|  |  |  |  |  |  |  |  | daf-16 | 172981 | NCBI | 0 | 0 | 1 | 1 |
|  |  |  |  |  |  |  |  | NCEH-1 | 189866 | NCBI | 0 | 0 | 1 | 1 |
|  |  |  |  |  |  |  |  | Secretory carrier membrane protein 5 | 192683 | NCBI | 0 | 0 | 1 | 1 |
|  |  |  |  |  |  |  |  | monoamine transporter | 214084 | NCBI | 1 | 0 | 0 | 1 |
|  |  |  |  |  |  |  |  | PIKE | 216439 | NCBI | 0 | 0 | 1 | 1 |
|  |  |  |  |  |  |  |  | SWT | 219793 | NCBI | 0 | 0 | 1 | 1 |
|  |  |  |  |  |  |  |  | Let-7 | 266952 | NCBI | 0 | 0 | 1 | 1 |
|  |  |  |  |  |  |  |  | NeuN | 287847 | NCBI | 0 | 1 | 0 | 1 |
|  |  |  |  |  |  |  |  | BAG3 | 293524 | NCBI | 0 | 0 | 1 | 1 |
|  |  |  |  |  |  |  |  | leucine-rich repeat kinase 2 | 300160 | NCBI | 1 | 0 | 0 | 1 |
|  |  |  |  |  |  |  |  | Sirtuin 2 | 361532 | NCBI | 0 | 0 | 1 | 1 |
|  |  |  |  |  |  |  |  | Atg5 | 365601 | NCBI | 0 | 0 | 1 | 1 |
|  |  |  |  |  |  |  |  | agrin | 375790 | NCBI | 1 | 0 | 0 | 1 |
|  |  |  |  |  |  |  |  | miR-132 | 387150 | NCBI | 1 | 0 | 0 | 1 |
|  |  |  |  |  |  |  |  | spot | 387357 | NCBI | 0 | 1 | 0 | 1 |
|  |  |  |  |  |  |  |  | sncb | 393944 | NCBI | 0 | 1 | 0 | 1 |
|  |  |  |  |  |  |  |  | sncg2 | 550229 | NCBI | 0 | 1 | 0 | 1 |
|  |  |  |  |  |  |  |  | sncg1 | 553679 | NCBI | 0 | 1 | 0 | 1 |
|  |  |  |  |  |  |  |  | scFv | 652070 | NCBI | 1 | 0 | 0 | 1 |
|  |  |  |  |  |  |  |  | miR-10a | 723893 | NCBI | 1 | 0 | 0 | 1 |
|  |  |  |  |  |  |  |  | Ssa1p | 851259 | NCBI | 0 | 1 | 0 | 1 |
|  |  |  |  |  |  |  |  | Pep1 | 852264 | NCBI | 0 | 0 | 1 | 1 |
|  |  |  |  |  |  |  |  | GAL1 | 852308 | NCBI | 0 | 1 | 0 | 1 |
|  |  |  |  |  |  |  |  | JEM1 | 853372 | NCBI | 1 | 0 | 0 | 1 |
|  |  |  |  |  |  |  |  | ARG2 | 853374 | NCBI | 1 | 0 | 0 | 1 |
|  |  |  |  |  |  |  |  | ENT3 | 853589 | NCBI | 1 | 0 | 0 | 1 |
|  |  |  |  |  |  |  |  | YCA1 | 854372 | NCBI | 1 | 0 | 0 | 1 |
|  |  |  |  |  |  |  |  | ATG32 | 854660 | NCBI | 0 | 1 | 0 | 1 |
|  |  |  |  |  |  |  |  | IDP3 | 855723 | NCBI | 1 | 0 | 0 | 1 |
|  |  |  |  |  |  |  |  | AIF1 | 855811 | NCBI | 1 | 0 | 0 | 1 |
|  |  |  |  |  |  |  |  | HSP82 | 855836 | NCBI | 1 | 0 | 0 | 1 |
|  |  |  |  |  |  |  |  | Pep4 | 855949 | NCBI | 0 | 0 | 1 | 1 |
|  |  |  |  |  |  |  |  | ATG11 | 856162 | NCBI | 0 | 1 | 0 | 1 |
|  |  |  |  |  |  |  |  | MG-132 | 875581 | NCBI | 0 | 1 | 0 | 1 |
|  |  |  |  |  |  |  |  | alpha-synuclein | 1.02E+08 | NCBI | 0 | 0 | 1 | 1 |
|  |  |  |  |  |  |  |  | N-acetylcysteine | C0001047 | UMLS | 0 | 0 | 1 | 1 |
|  |  |  |  |  |  |  |  | transacetylases | C0001068 | UMLS | 1 | 0 | 0 | 1 |
|  |  |  |  |  |  |  |  | aequorin | C0001694 | UMLS | 0 | 1 | 0 | 1 |
|  |  |  |  |  |  |  |  | albumin | C0001924 | UMLS | 0 | 1 | 0 | 1 |
|  |  |  |  |  |  |  |  | allelic | C0002085 | UMLS | 0 | 0 | 1 | 1 |
|  |  |  |  |  |  |  |  | alpha-galactosidase | C0002268 | UMLS | 0 | 0 | 1 | 1 |
|  |  |  |  |  |  |  |  | alpha-glucosidase | C0002272 | UMLS | 0 | 0 | 1 | 1 |
|  |  |  |  |  |  |  |  | alpha/beta-tubulin | C0002318 | UMLS | 1 | 0 | 0 | 1 |
|  |  |  |  |  |  |  |  | ANAs | C0003241 | UMLS | 0 | 1 | 0 | 1 |
|  |  |  |  |  |  |  |  | antigen-antibody complexes | C0003313 | UMLS | 0 | 0 | 1 | 1 |
|  |  |  |  |  |  |  |  | apolipoprotein | C0003591 | UMLS | 0 | 0 | 1 | 1 |
|  |  |  |  |  |  |  |  | arginase | C0003762 | UMLS | 1 | 0 | 0 | 1 |
|  |  |  |  |  |  |  |  | pentraxin 1 | C0006560 | UMLS | 0 | 0 | 1 | 1 |
|  |  |  |  |  |  |  |  | calcium-permeable ion channels | C0006685 | UMLS | 0 | 0 | 1 | 1 |
|  |  |  |  |  |  |  |  | calcium-binding protein | C0006732 | UMLS | 1 | 0 | 0 | 1 |
|  |  |  |  |  |  |  |  | SERT protein | C0007292 | UMLS | 0 | 0 | 1 | 1 |
|  |  |  |  |  |  |  |  | cathepsin | C0007428 | UMLS | 1 | 0 | 0 | 1 |
|  |  |  |  |  |  |  |  | choline acetyltransferase | C0008407 | UMLS | 0 | 1 | 0 | 1 |
|  |  |  |  |  |  |  |  | chromatin | C0008546 | UMLS | 0 | 0 | 1 | 1 |
|  |  |  |  |  |  |  |  | protein kinase G | C0010536 | UMLS | 0 | 1 | 0 | 1 |
|  |  |  |  |  |  |  |  | cystatin | C0010646 | UMLS | 1 | 0 | 0 | 1 |
|  |  |  |  |  |  |  |  | cytochrome c | C0010754 | UMLS | 1 | 0 | 0 | 1 |
|  |  |  |  |  |  |  |  | galactocerebrosidase | C0016957 | UMLS | 0 | 0 | 1 | 1 |
|  |  |  |  |  |  |  |  | genetic libraries | C0017272 | UMLS | 0 | 1 | 0 | 1 |
|  |  |  |  |  |  |  |  | lysosomal enzyme glucocerebrosidase | C0017768 | UMLS | 0 | 0 | 1 | 1 |
|  |  |  |  |  |  |  |  | glutathione S-transferase | C0017837 | UMLS | 0 | 1 | 0 | 1 |
|  |  |  |  |  |  |  |  | glycine | C0017890 | UMLS | 0 | 0 | 1 | 1 |
|  |  |  |  |  |  |  |  | hexokinase | C0019472 | UMLS | 0 | 0 | 1 | 1 |
|  |  |  |  |  |  |  |  | human leukocyte antigen | C0019629 | UMLS | 0 | 0 | 1 | 1 |
|  |  |  |  |  |  |  |  | major histocompatibility complex class II | C0019630 | UMLS | 1 | 0 | 0 | 1 |
|  |  |  |  |  |  |  |  | TH | C0020364 | UMLS | 1 | 0 | 0 | 1 |
|  |  |  |  |  |  |  |  | immunoglobulin | C0021027 | UMLS | 1 | 0 | 0 | 1 |
|  |  |  |  |  |  |  |  | insulin | C0021641 | UMLS | 0 | 0 | 1 | 1 |
|  |  |  |  |  |  |  |  | IGF-1- | C0021665 | UMLS | 0 | 0 | 1 | 1 |
|  |  |  |  |  |  |  |  | transmembrane accessory proteins | C0021699 | UMLS | 0 | 1 | 0 | 1 |
|  |  |  |  |  |  |  |  | integrins | C0021701 | UMLS | 0 | 0 | 1 | 1 |
|  |  |  |  |  |  |  |  | interferon-gamma | C0021740 | UMLS | 1 | 0 | 0 | 1 |
|  |  |  |  |  |  |  |  | interleukin-6 | C0021760 | UMLS | 0 | 1 | 0 | 1 |
|  |  |  |  |  |  |  |  | interleukin | C0021764 | UMLS | 0 | 0 | 1 | 1 |
|  |  |  |  |  |  |  |  | lectin | C0023206 | UMLS | 1 | 0 | 0 | 1 |
|  |  |  |  |  |  |  |  | lipoprotein | C0023820 | UMLS | 0 | 0 | 1 | 1 |
|  |  |  |  |  |  |  |  | high-density lipoprotein | C0023821 | UMLS | 0 | 0 | 1 | 1 |
|  |  |  |  |  |  |  |  | major histocompatibility complex | C0024518 | UMLS | 0 | 0 | 1 | 1 |
|  |  |  |  |  |  |  |  | membrane associated proteins | C0025252 | UMLS | 0 | 0 | 1 | 1 |
|  |  |  |  |  |  |  |  | metalloproteases | C0025543 | UMLS | 0 | 0 | 1 | 1 |
|  |  |  |  |  |  |  |  | methionine | C0025646 | UMLS | 0 | 0 | 1 | 1 |
|  |  |  |  |  |  |  |  | peroxidase | C0027021 | UMLS | 0 | 0 | 1 | 1 |
|  |  |  |  |  |  |  |  | nuclear protein | C0028589 | UMLS | 0 | 1 | 0 | 1 |
|  |  |  |  |  |  |  |  | PDEs | C0031640 | UMLS | 0 | 0 | 1 | 1 |
|  |  |  |  |  |  |  |  | alpha-Syn toxicity | C0031669 | UMLS | 0 | 0 | 1 | 1 |
|  |  |  |  |  |  |  |  | protein phosphatases | C0031686 | UMLS | 0 | 1 | 0 | 1 |
|  |  |  |  |  |  |  |  | cyclooxygenase | C0033551 | UMLS | 1 | 0 | 0 | 1 |
|  |  |  |  |  |  |  |  | muscarinic receptor | C0034826 | UMLS | 0 | 0 | 1 | 1 |
|  |  |  |  |  |  |  |  | nicotinic acetylcholine receptor | C0034830 | UMLS | 0 | 0 | 1 | 1 |
|  |  |  |  |  |  |  |  | ribosomal proteins | C0035552 | UMLS | 1 | 0 | 0 | 1 |
|  |  |  |  |  |  |  |  | serine proteases | C0036734 | UMLS | 1 | 0 | 0 | 1 |
|  |  |  |  |  |  |  |  | bovine serum albumin | C0036774 | UMLS | 0 | 0 | 1 | 1 |
|  |  |  |  |  |  |  |  | sodium channel | C0037492 | UMLS | 1 | 0 | 0 | 1 |
|  |  |  |  |  |  |  |  | spectrin | C0037799 | UMLS | 0 | 1 | 0 | 1 |
|  |  |  |  |  |  |  |  | sphingomyelinases | C0037903 | UMLS | 0 | 0 | 1 | 1 |
|  |  |  |  |  |  |  |  | superoxide dismutase | C0038838 | UMLS | 0 | 1 | 0 | 1 |
|  |  |  |  |  |  |  |  | SCOPA-MS | C0039676 | UMLS | 1 | 0 | 0 | 1 |
|  |  |  |  |  |  |  |  | Thioredoxin | C0039938 | UMLS | 0 | 0 | 1 | 1 |
|  |  |  |  |  |  |  |  | trans-activator | C0040627 | UMLS | 0 | 0 | 1 | 1 |
|  |  |  |  |  |  |  |  | valine | C0042285 | UMLS | 0 | 0 | 1 | 1 |
|  |  |  |  |  |  |  |  | K(d) approximately 4 | C0052012 | UMLS | 1 | 0 | 0 | 1 |
|  |  |  |  |  |  |  |  | calbindin | C0054444 | UMLS | 1 | 0 | 0 | 1 |
|  |  |  |  |  |  |  |  | D2 receptors | C0058698 | UMLS | 0 | 0 | 1 | 1 |
|  |  |  |  |  |  |  |  | glutamate transporter | C0061467 | UMLS | 0 | 0 | 1 | 1 |
|  |  |  |  |  |  |  |  | GTPase activating proteins | C0061928 | UMLS | 1 | 0 | 0 | 1 |
|  |  |  |  |  |  |  |  | halorhodopsin | C0062108 | UMLS | 0 | 0 | 1 | 1 |
|  |  |  |  |  |  |  |  | islet amyloid polypeptide | C0063684 | UMLS | 0 | 1 | 0 | 1 |
|  |  |  |  |  |  |  |  | keyhole limpet hemocyanin | C0064332 | UMLS | 0 | 0 | 1 | 1 |
|  |  |  |  |  |  |  |  | link protein | C0065011 | UMLS | 0 | 1 | 0 | 1 |
|  |  |  |  |  |  |  |  | lipoprotein (a) | C0065058 | UMLS | 0 | 0 | 1 | 1 |
|  |  |  |  |  |  |  |  | lysosome-related proteins | C0065400 | UMLS | 0 | 1 | 0 | 1 |
|  |  |  |  |  |  |  |  | porins | C0071728 | UMLS | 0 | 1 | 0 | 1 |
|  |  |  |  |  |  |  |  | protease cathepsin D | C0072115 | UMLS | 0 | 0 | 1 | 1 |
|  |  |  |  |  |  |  |  | Fyn tyrosine kinase | C0072475 | UMLS | 0 | 0 | 1 | 1 |
|  |  |  |  |  |  |  |  | PrP(res) | C0074204 | UMLS | 0 | 0 | 1 | 1 |
|  |  |  |  |  |  |  |  | synaptosomal-associated protein of 25 | C0075689 | UMLS | 0 | 1 | 0 | 1 |
|  |  |  |  |  |  |  |  | villin | C0078238 | UMLS | 1 | 0 | 0 | 1 |
|  |  |  |  |  |  |  |  | leucine zipper | C0079686 | UMLS | 1 | 0 | 0 | 1 |
|  |  |  |  |  |  |  |  | NMDA) receptor | C0080093 | UMLS | 0 | 0 | 1 | 1 |
|  |  |  |  |  |  |  |  | different disease-associated proteins | C0082213 | UMLS | 0 | 0 | 1 | 1 |
|  |  |  |  |  |  |  |  | trkB Receptors | C0084873 | UMLS | 0 | 0 | 1 | 1 |
|  |  |  |  |  |  |  |  | amyloid precursor protein | C0085151 | UMLS | 0 | 1 | 0 | 1 |
|  |  |  |  |  |  |  |  | intravenous immunoglobulin | C0085297 | UMLS | 0 | 1 | 0 | 1 |
|  |  |  |  |  |  |  |  | NAA | C0085845 | UMLS | 0 | 0 | 1 | 1 |
|  |  |  |  |  |  |  |  | Gammagard | C0086333 | UMLS | 0 | 1 | 0 | 1 |
|  |  |  |  |  |  |  |  | synapsin I/II | C0087045 | UMLS | 0 | 0 | 1 | 1 |
|  |  |  |  |  |  |  |  | 14-3-3 proteins | C0090388 | UMLS | 0 | 1 | 0 | 1 |
|  |  |  |  |  |  |  |  | CKI | C0108554 | UMLS | 0 | 1 | 0 | 1 |
|  |  |  |  |  |  |  |  | CCAAT/-enhancer-binding protein | C0108685 | UMLS | 0 | 1 | 0 | 1 |
|  |  |  |  |  |  |  |  | Fcgamma receptor | C0123263 | UMLS | 0 | 1 | 0 | 1 |
|  |  |  |  |  |  |  |  | transcription factor Nuclear factor erythroid-2 | C0126037 | UMLS | 0 | 1 | 0 | 1 |
|  |  |  |  |  |  |  |  | plant origin | C0162728 | UMLS | 0 | 1 | 0 | 1 |
|  |  |  |  |  |  |  |  | glucose transporter type 4 | C0166441 | UMLS | 0 | 0 | 1 | 1 |
|  |  |  |  |  |  |  |  | glial cell line-derived neurotrophic factor | C0207072 | UMLS | 0 | 1 | 0 | 1 |
|  |  |  |  |  |  |  |  | glycogen synthase kinase 3beta | C0244988 | UMLS | 1 | 0 | 0 | 1 |
|  |  |  |  |  |  |  |  | fat body protein 1 | C0255858 | UMLS | 1 | 0 | 0 | 1 |
|  |  |  |  |  |  |  |  | Src family tyrosine kinases | C0282625 | UMLS | 0 | 0 | 1 | 1 |
|  |  |  |  |  |  |  |  | Divalent metal transporter 1 | C0288148 | UMLS | 0 | 0 | 1 | 1 |
|  |  |  |  |  |  |  |  | presenilin-1 | C0299212 | UMLS | 0 | 1 | 0 | 1 |
|  |  |  |  |  |  |  |  | Human albumin | C0304925 | UMLS | 0 | 0 | 1 | 1 |
|  |  |  |  |  |  |  |  | human Spr gene | C0314604 | UMLS | 0 | 1 | 0 | 1 |
|  |  |  |  |  |  |  |  | nature killer cell receptor | C0382839 | UMLS | 1 | 0 | 0 | 1 |
|  |  |  |  |  |  |  |  | amyloid fibers | C0475316 | UMLS | 0 | 1 | 0 | 1 |
|  |  |  |  |  |  |  |  | tissue transglutaminase | C0529334 | UMLS | 1 | 0 | 0 | 1 |
|  |  |  |  |  |  |  |  | metabotropic glutamate receptor 5 | C0530778 | UMLS | 0 | 0 | 1 | 1 |
|  |  |  |  |  |  |  |  | Neurturin | C0531925 | UMLS | 0 | 1 | 0 | 1 |
|  |  |  |  |  |  |  |  | SUMO E3 ligase protein | C0534100 | UMLS | 0 | 0 | 1 | 1 |
|  |  |  |  |  |  |  |  | HO-1 | C0538674 | UMLS | 0 | 1 | 0 | 1 |
|  |  |  |  |  |  |  |  | enzyme digestion | C0544420 | UMLS | 1 | 0 | 0 | 1 |
|  |  |  |  |  |  |  |  | Amino Acid Supplementation | C0556082 | UMLS | 0 | 0 | 1 | 1 |
|  |  |  |  |  |  |  |  | diethyldithiocarbamate | C0556252 | UMLS | 0 | 0 | 1 | 1 |
|  |  |  |  |  |  |  |  | peptide analog | C0597191 | UMLS | 1 | 0 | 0 | 1 |
|  |  |  |  |  |  |  |  | Peptide hormone | C0597192 | UMLS | 0 | 0 | 1 | 1 |
|  |  |  |  |  |  |  |  | protein 1 isoform | C0597298 | UMLS | 1 | 0 | 0 | 1 |
|  |  |  |  |  |  |  |  | adapter proteins | C0599697 | UMLS | 1 | 0 | 0 | 1 |
|  |  |  |  |  |  |  |  | STF-31 | C0599939 | UMLS | 0 | 0 | 1 | 1 |
|  |  |  |  |  |  |  |  | interleukin 1alpha | C0600251 | UMLS | 0 | 0 | 1 | 1 |
|  |  |  |  |  |  |  |  | extracellular signal-regulated kinase | C0600388 | UMLS | 0 | 1 | 0 | 1 |
|  |  |  |  |  |  |  |  | essential yeast gene | C0600449 | UMLS | 0 | 1 | 0 | 1 |
|  |  |  |  |  |  |  |  | epoxomicin | C0656383 | UMLS | 0 | 0 | 1 | 1 |
|  |  |  |  |  |  |  |  | alpha-klotho protein | C0667394 | UMLS | 0 | 0 | 1 | 1 |
|  |  |  |  |  |  |  |  | mutant cDNA genes | C0678941 | UMLS | 0 | 1 | 0 | 1 |
|  |  |  |  |  |  |  |  | cathepsins B | C0699919 | UMLS | 1 | 0 | 0 | 1 |
|  |  |  |  |  |  |  |  | cDNA library | C0751608 | UMLS | 0 | 1 | 0 | 1 |
|  |  |  |  |  |  |  |  | Small GTPases | C0751984 | UMLS | 1 | 0 | 0 | 1 |
|  |  |  |  |  |  |  |  | apoptosis-inducing factor | C0763396 | UMLS | 1 | 0 | 0 | 1 |
|  |  |  |  |  |  |  |  | calcium/calmodulin-dependent protein kinase | C0769224 | UMLS | 0 | 1 | 0 | 1 |
|  |  |  |  |  |  |  |  | amino acids 2-4 | C0771098 | UMLS | 1 | 0 | 0 | 1 |
|  |  |  |  |  |  |  |  | voltage-activated calcium channels | C0814022 | UMLS | 0 | 0 | 1 | 1 |
|  |  |  |  |  |  |  |  | protein function | C0815043 | UMLS | 0 | 0 | 1 | 1 |
|  |  |  |  |  |  |  |  | toxic misfolded proteins | C0815048 | UMLS | 0 | 0 | 1 | 1 |
|  |  |  |  |  |  |  |  | caveolin | C0887901 | UMLS | 0 | 0 | 1 | 1 |
|  |  |  |  |  |  |  |  | dynamin-1 K44A | C0904071 | UMLS | 1 | 0 | 0 | 1 |
|  |  |  |  |  |  |  |  | ferroportin | C0915115 | UMLS | 0 | 0 | 1 | 1 |
|  |  |  |  |  |  |  |  | dsRED | C0960939 | UMLS | 0 | 0 | 1 | 1 |
|  |  |  |  |  |  |  |  | of nuclear receptor-related 1 protein | C0961411 | UMLS | 0 | 1 | 0 | 1 |
|  |  |  |  |  |  |  |  | toll-like receptors (TLRs) 1 | C0971363 | UMLS | 0 | 1 | 0 | 1 |
|  |  |  |  |  |  |  |  | sirtuins | C1136177 | UMLS | 0 | 1 | 0 | 1 |
|  |  |  |  |  |  |  |  | scaffolding protein | C1179132 | UMLS | 0 | 0 | 1 | 1 |
|  |  |  |  |  |  |  |  | F-actin | C1180307 | UMLS | 0 | 0 | 1 | 1 |
|  |  |  |  |  |  |  |  | protein complex | C1180347 | UMLS | 1 | 0 | 0 | 1 |
|  |  |  |  |  |  |  |  | phosphorylation-state-specific TH antibodies | C1257988 | UMLS | 1 | 0 | 0 | 1 |
|  |  |  |  |  |  |  |  | enhanced green fluorescent protein | C1258415 | UMLS | 1 | 0 | 0 | 1 |
|  |  |  |  |  |  |  |  | polypeptides | C1305923 | UMLS | 0 | 1 | 0 | 1 |
|  |  |  |  |  |  |  |  | deacetylases | C1333261 | UMLS | 0 | 1 | 0 | 1 |
|  |  |  |  |  |  |  |  | extracellular target proteins | C1333498 | UMLS | 0 | 1 | 0 | 1 |
|  |  |  |  |  |  |  |  | luciferase-based gene | C1334435 | UMLS | 0 | 0 | 1 | 1 |
|  |  |  |  |  |  |  |  | mouse protein | C1334805 | UMLS | 0 | 0 | 1 | 1 |
|  |  |  |  |  |  |  |  | signaling pathways. alpha-syn protein | C1335962 | UMLS | 0 | 0 | 1 | 1 |
|  |  |  |  |  |  |  |  | synuclein family | C1336547 | UMLS | 1 | 0 | 0 | 1 |
|  |  |  |  |  |  |  |  | transcriptional coactivator | C1336776 | UMLS | 0 | 1 | 0 | 1 |
|  |  |  |  |  |  |  |  | mouse myelin basic protein | C1450293 | UMLS | 0 | 0 | 1 | 1 |
|  |  |  |  |  |  |  |  | Thioredoxin-interacting protein | C1450344 | UMLS | 0 | 0 | 1 | 1 |
|  |  |  |  |  |  |  |  | vesicle SNARE | C1506158 | UMLS | 0 | 0 | 1 | 1 |
|  |  |  |  |  |  |  |  | t-SNARE | C1506159 | UMLS | 0 | 0 | 1 | 1 |
|  |  |  |  |  |  |  |  | tet | C1515333 | UMLS | 0 | 1 | 0 | 1 |
|  |  |  |  |  |  |  |  | DOPAL | C1517852 | UMLS | 0 | 0 | 1 | 1 |
|  |  |  |  |  |  |  |  | interleukins 4 | C1522540 | UMLS | 1 | 0 | 0 | 1 |
|  |  |  |  |  |  |  |  | vesicular monoamine transporters | C1529240 | UMLS | 1 | 0 | 0 | 1 |
|  |  |  |  |  |  |  |  | Transient receptor potential canonical (TRPC) channels | C1563722 | UMLS | 0 | 0 | 1 | 1 |
|  |  |  |  |  |  |  |  | mutant disease proteins | C1564139 | UMLS | 1 | 0 | 0 | 1 |
|  |  |  |  |  |  |  |  | GATA transcription factors | C1564871 | UMLS | 1 | 0 | 0 | 1 |
|  |  |  |  |  |  |  |  | programmed cell death proteins | C1564881 | UMLS | 0 | 1 | 0 | 1 |
|  |  |  |  |  |  |  |  | liver-X-receptors | C1612060 | UMLS | 1 | 0 | 0 | 1 |
|  |  |  |  |  |  |  |  | protein phosphatase 2A | C1704708 | UMLS | 0 | 1 | 0 | 1 |
|  |  |  |  |  |  |  |  | AMP-activated protein kinase | C2350345 | UMLS | 0 | 0 | 1 | 1 |
|  |  |  |  |  |  |  |  | glucose-regulated protein | C2599779 | UMLS | 0 | 1 | 0 | 1 |
|  |  |  |  |  |  |  |  | human IgG | C2702333 | UMLS | 0 | 1 | 0 | 1 |
|  |  |  |  |  |  |  |  | chaperonin CCT | C2717814 | UMLS | 0 | 0 | 1 | 1 |
|  |  |  |  |  |  |  |  | KYP-2047 | C2935972 | UMLS | 0 | 1 | 0 | 1 |
|  |  |  |  |  |  |  |  | modifier genes | C3178895 | UMLS | 1 | 0 | 0 | 1 |
|  |  |  |  |  |  |  |  | ascorbate peroxidase | C3178941 | UMLS | 0 | 0 | 1 | 1 |
|  |  |  |  |  |  |  |  | autophagy protein 5 | C3537312 | UMLS | 0 | 0 | 1 | 1 |
|  |  |  |  |  |  |  |  | Hip | C3538851 | UMLS | 0 | 0 | 1 | 1 |
|  |  |  |  |  |  |  |  | synphilin-1 | C3541909 | UMLS | 1 | 0 | 0 | 1 |
|  |  |  |  |  |  |  |  | APOE epsilon4 allele | C3642141 | UMLS | 1 | 0 | 0 | 1 |
|  |  |  |  |  |  |  |  | eQTLs | C3826857 | UMLS | 0 | 0 | 1 | 1 |
