# Supplementary material for: Menagerie: A text-mining tool to support animal-human translation in neurodegeneration research
Source: PLoS One. 2019 Dec 17;14(12):e0226176. doi: 10.1371/journal.pone.0226176 (PMC6917268; doi:10.1371/journal.pone.0226176)
Supplement: S5 Table — (DOCX) [file pone.0226176.s006.docx]

**S5 Table:** The most 15 significantly enriched pathways for L-DOPA and alpha-synuclein associated studies are shown

| **Intervention module: L-DOPA** | | | |
| --- | --- | --- | --- |
| **Pathway identifier** | **Pathway name** | **Entities ratio** | **Entities pValue** |
| R-HSA-390651 | Dopamine receptors | 4E-04 | 1E-06 |
| R-HSA-447038 | NrCAM interactions | 5E-04 | 9E-05 |
| R-HSA-9032500 | Activated NTRK2 signals through FYN | 8E-04 | 3E-04 |
| R-HSA-390666 | Serotonin receptors | 9E-04 | 5E-04 |
| R-HSA-8849932 | Synaptic adhesion-like molecules | 2E-03 | 1E-05 |
| R-HSA-442982 | Ras activation upon Ca2+ influx through NMDA receptor | 2E-03 | 2E-05 |
| R-HSA-389357 | CD28 dependent PI3K/Akt signaling | 2E-03 | 3E-04 |
| R-HSA-9617324 | Negative regulation of NMDA receptor-mediated neuronal transmission | 2E-03 | 2E-05 |
| R-HSA-438066 | Unblocking of NMDA receptors, glutamate binding and activation | 2E-03 | 1E-06 |
| R-HSA-9620244 | Long-term potentiation | 2E-03 | 2E-06 |
| R-HSA-5674400 | Constitutive Signaling by AKT1 E17K in Cancer | 2E-03 | 5E-05 |
| R-HSA-9006115 | Signaling by NTRK2 (TRKB) | 2E-03 | 5E-05 |
| R-HSA-442742 | CREB1 phosphorylation through NMDA receptor-mediated activation of RAS signaling | 3E-03 | 5E-07 |
| R-HSA-9609736 | Assembly and cell surface presentation of NMDA receptors | 3E-03 | 3E-04 |
| R-HSA-6794362 | Protein-protein interactions at synapses | 7E-03 | 1E-03 |
| **Intervention module: alpha-synuclein** | | | |
| **Pathway identifier** | **Pathway name** | **Entities ratio** | **Entities pValue** |
| R-HSA-111469 | SMAC, XIAP-regulated apoptotic response | 6E-04 | 2E-04 |
| R-HSA-9032500 | Activated NTRK2 signals through FYN | 8E-04 | 6E-04 |
| R-HSA-198693 | AKT phosphorylates targets in the nucleus | 8E-04 | 9E-04 |
| R-HSA-381183 | ATF6 (ATF6-alpha) activates chaperone genes | 1E-03 | 2E-04 |
| R-HSA-381033 | ATF6 (ATF6-alpha) activates chaperones | 1E-03 | 3E-04 |
| R-HSA-111471 | Apoptotic factor-mediated response | 1E-03 | 4E-04 |
| R-HSA-9634638 | Estrogen-dependent nuclear events downstream of ESR-membrane signaling | 2E-03 | 9E-06 |
| R-HSA-5205647 | Mitophagy | 2E-03 | 8E-04 |
| R-HSA-5674400 | Constitutive Signaling by AKT1 E17K in Cancer | 2E-03 | 1E-04 |
| R-HSA-442742 | CREB1 phosphorylation through NMDA receptor-mediated activation of RAS signaling | 3E-03 | 4E-04 |
| R-HSA-9615017 | FOXO-mediated transcription of oxidative stress, metabolic and neuronal genes | 3E-03 | 3E-04 |
| R-HSA-109606 | Intrinsic Pathway for Apoptosis | 4E-03 | 8E-06 |
| R-HSA-375165 | NCAM signaling for neurite out-growth | 5E-03 | 7E-04 |
| R-HSA-170834 | Signaling by TGF-beta Receptor Complex | 6E-03 | 8E-04 |
| R-HSA-977225 | Amyloid fiber formation | 6E-03 | 3E-06 |
