# Supplementary material for: Menagerie: A text-mining tool to support animal-human translation in neurodegeneration research
Source: PLoS One. 2019 Dec 17;14(12):e0226176. doi: 10.1371/journal.pone.0226176 (PMC6917268; doi:10.1371/journal.pone.0226176)
Supplement: S6 Table — The number of studies mentioning each gene by species is colored coded (green = least, red = most). (DOCX) [file pone.0226176.s007.docx]

**S6 Table:** Gene mentions across species in papers in which alpha synuclein has been identified using the Interventions module. The number of studies mentioning each gene by species is colored coded (green=least, red =most).

| **Gene term** | **Term ID** | **Human** | **Macaque** | **Marmoset** | **Dog** | **Mouse** | **Rat** | **Fish** | **Fly** | **Worm** | **Yeast** |
| --- | --- | --- | --- | --- | --- | --- | --- | --- | --- | --- | --- |
| SNCA | 6622 | 89 | 1 | 1 | 1 | 125 | 37 | 2 | 10 | 8 | 19 |
| PARK8/LRRK2 | 120892 | 5 |  |  |  | 5 | 1 |  |  |  | 1 |
| MAPT | 4137 | 5 |  |  |  | 2 |  |  |  |  |  |
| Snca | 20617 | 4 |  |  |  | 38 |  |  |  | 1 |  |
| GBA1 | 2629 | 3 |  |  |  | 1 |  |  |  |  |  |
| PARK9/ATP13A2 | 23400 | 2 |  |  |  | 1 |  |  |  | 1 | 2 |
| Syn | 23336 | 2 |  |  |  | 2 |  | 1 |  |  | 1 |
| TNFalpha | 7124 | 2 |  |  |  |  |  |  |  |  |  |
| Thy1 | 7070 | 2 |  |  |  | 5 |  |  |  |  |  |
| tyrosine hydroxylase | 7054 | 2 |  |  |  | 7 | 4 |  |  | 1 |  |
| PARK2 | 5071 | 2 |  |  |  | 1 |  |  |  |  | 1 |
| Nurr1 | 4929 | 2 |  |  |  | 3 | 1 |  |  | 1 |  |
| IFN-gamma | 3458 | 2 |  |  |  | 1 |  |  |  |  |  |
| syn | 2534 | 2 |  |  |  | 4 | 3 |  |  |  |  |
| Rep1 | 1121 | 2 |  |  |  |  |  |  |  |  |  |
| cAMP response element binding protein | 820 | 2 |  |  |  | 2 |  |  |  |  |  |
| SNARE | 100170220 | 1 |  |  |  | 1 |  |  |  |  |  |
| pS129 | 2843875 | 1 |  |  |  | 1 |  |  |  |  |  |
| miR-10a | 723893 | 1 |  |  |  | 1 |  |  |  |  |  |
| miRNA-433 | 574034 | 1 |  |  |  |  |  |  |  |  |  |
| miR-132 | 387150 | 1 |  |  |  | 1 |  |  |  |  |  |
| SCAMP5 | 192683 | 1 |  |  |  |  |  |  |  |  |  |
| NeuN | 146713 | 1 |  |  |  | 1 |  |  |  |  |  |
| CADPS2 | 93664 | 1 |  |  |  |  |  |  |  |  |  |
| Mhc2ta | 85483 | 1 |  |  |  |  | 1 |  |  |  |  |
| PARK6/PINK1 | 65018 | 1 |  |  |  |  | 1 |  | 2 |  | 1 |
| PARK17 | 55737 | 1 |  |  |  |  |  |  |  |  | 1 |
| VPS35 | 55737 | 1 |  |  |  |  |  |  |  |  | 1 |
| PARK2 | 50873 | 1 |  |  |  | 1 |  |  |  |  |  |
| GDNF | 25453 | 1 |  |  |  | 1 | 1 |  |  |  |  |
| TDP-43 | 23435 | 1 |  |  |  |  |  |  |  |  |  |
| VIP | 22353 | 1 |  |  |  | 2 |  |  |  |  |  |
| Thy-1 | 21838 | 1 |  |  |  | 8 |  |  |  |  |  |
| SNAP-25 | 20614 | 1 |  |  |  | 1 |  |  |  |  |  |
| Ret | 19713 | 1 |  |  |  | 1 | 1 |  |  |  |  |
| Nurr1 | 18227 | 1 |  |  |  | 2 | 1 |  |  |  |  |
| Nramp1 | 18173 | 1 |  |  |  | 1 |  |  |  |  |  |
| nNOS | 18125 | 1 |  |  |  | 3 |  |  |  |  |  |
| NQO1 | 18104 | 1 |  |  |  | 1 |  |  |  |  |  |
| Nrf2 | 18024 | 1 |  |  |  | 2 |  |  |  |  |  |
| IL-6 | 16193 | 1 |  |  |  | 1 |  |  |  |  |  |
| IL-1beta | 16176 | 1 |  |  |  | 3 |  |  |  |  |  |
| heme oxygenase-1 | 15368 | 1 |  |  |  | 1 |  |  |  |  |  |
| GDNF | 14573 | 1 |  |  |  | 1 | 1 |  |  |  |  |
| cathepsin D | 13033 | 1 |  |  |  | 2 |  |  |  | 1 |  |
| CREB | 12912 | 1 |  |  |  | 1 |  |  |  |  |  |
| PARK7/DJ-1 gene | 11315 | 1 |  |  |  | 2 |  |  |  |  |  |
| miR-7 | 10859 | 1 |  |  |  |  |  |  |  |  |  |
| GLA | 9027 | 1 |  |  |  |  |  |  |  |  |  |
| A beta | 8803 | 1 |  |  |  |  |  |  |  |  |  |
| CADPS | 8618 | 1 |  |  |  |  |  |  |  |  |  |
| UTR | 8170 | 1 |  |  |  |  |  |  |  |  |  |
| TFEB | 7942 | 1 |  |  |  |  |  |  |  |  |  |
| VIP | 7432 | 1 |  |  |  |  |  |  |  |  |  |
| PGP 9.5 | 7345 | 1 |  |  |  |  |  |  |  |  |  |
| MSA | 7173 | 1 |  |  |  | 1 | 1 |  |  |  |  |
| top | 7064 | 1 |  |  |  | 1 |  |  |  |  | 1 |
| Nramp1 | 6556 | 1 |  |  |  | 1 |  |  |  |  |  |
| ATXN2 | 6311 | 1 |  |  |  |  |  |  |  |  | 1 |
| Ret | 5979 | 1 |  |  |  | 1 | 1 |  |  |  |  |
| cyclooxygenase-2 | 5743 | 1 |  |  |  |  |  |  |  |  |  |
| beta-trace protein | 5730 | 1 |  |  |  | 1 |  |  |  |  |  |
| PREP | 5550 | 1 |  |  |  | 1 |  |  |  |  |  |
| PDC | 5132 | 1 |  |  |  |  |  |  |  |  |  |
| Inducible nitric oxide synthase | 4843 | 1 |  |  |  | 2 | 1 |  |  |  |  |
| iNOS | 4843 | 1 |  |  |  | 2 | 1 |  |  |  |  |
| NGF | 4803 | 1 |  |  |  | 1 |  |  |  |  |  |
| Nrf2 | 4780 | 1 |  |  |  | 1 |  |  |  |  |  |
| COX-2 | 4513 | 1 |  |  |  |  |  |  |  |  |  |
| Atx3 | 4287 | 1 |  |  |  |  |  |  |  |  |  |
| Mhc2ta | 4261 | 1 |  |  |  |  | 1 |  |  |  |  |
| CD11b | 3684 | 1 |  |  |  |  |  |  |  |  |  |
| Il-6 | 3569 | 1 |  |  |  |  |  |  |  |  |  |
| interleukin-2 | 3558 | 1 |  |  |  |  |  |  |  |  |  |
| ALS | 3483 | 1 |  |  |  |  |  |  |  |  |  |
| alpha-iduronidase | 3425 | 1 |  |  |  |  |  |  |  |  |  |
| serotonin receptor 4 | 3360 | 1 |  |  |  |  |  |  |  |  |  |
| serotonin receptor 3A | 3359 | 1 |  |  |  |  |  |  |  |  |  |
| sPD | 3239 | 1 |  |  |  |  |  |  |  |  |  |
| P62 | 2965 | 1 |  |  |  |  |  |  |  |  |  |
| Mer | 2852 | 1 |  |  |  | 1 |  |  |  |  |  |
| GDNF | 2668 | 1 |  |  |  | 1 | 1 |  |  |  |  |
| TOF | 2626 | 1 |  |  |  |  |  |  |  |  |  |
| Nrf2 | 2551 | 1 |  |  |  | 1 |  |  |  |  |  |
| cFos | 2353 | 1 |  |  |  | 2 |  |  |  |  |  |
| FABP3 | 2170 | 1 |  |  |  |  |  |  |  |  |  |
| ENSA | 2029 | 1 |  |  |  |  |  |  |  |  |  |
| neuron-specific enolase | 2026 | 1 |  |  |  | 1 |  |  |  |  |  |
| hub | 1993 | 1 |  |  |  |  |  |  |  |  | 1 |
| PARK18/EIF4G1 | 1981 | 1 |  |  |  |  |  |  |  |  | 1 |
| dopamine receptor D1 | 1812 | 1 |  |  |  |  |  |  |  |  |  |
| CAPS | 1483 | 1 |  |  |  |  |  |  |  |  |  |
| CREB | 1385 | 1 |  |  |  | 1 |  |  |  |  |  |
| cis-acting elements | 1154 | 1 |  |  |  | 1 |  |  |  |  |  |
| CD8 | 925 | 1 |  |  |  | 1 | 2 |  |  |  |  |
| CD4 | 920 | 1 |  |  |  |  | 1 |  |  |  |  |
| catalase | 847 | 1 |  |  |  |  |  |  |  |  |  |
| Axl | 558 | 1 |  |  |  | 1 |  |  |  |  |  |
| amyloid-beta peptide | 351 | 1 |  |  |  | 2 |  |  |  |  |  |
| APOE | 348 | 1 |  |  |  | 1 |  |  |  |  |  |
| PACAP | 116 | 1 |  |  |  | 1 |  |  |  |  |  |
| SNCA | 102119369 |  | 1 |  |  |  |  |  |  |  |  |
| MG-132 | 875581 |  |  |  |  |  |  |  |  |  | 1 |
| ATG11 | 856162 |  |  |  |  |  |  |  |  |  | 1 |
| Pep4 | 855949 |  |  |  |  |  |  |  |  |  | 1 |
| HSP82 | 855836 |  |  |  |  |  |  |  |  |  | 1 |
| AIF1 | 855811 |  |  |  |  |  |  |  |  |  | 1 |
| IDP3 | 855723 |  |  |  |  |  |  |  |  |  | 1 |
| ATG32 | 854660 |  |  |  |  |  |  |  |  |  | 1 |
| YCA1 | 854372 |  |  |  |  |  |  |  |  |  | 1 |
| ENT3 | 853589 |  |  |  |  |  |  |  |  |  | 1 |
| ARG2 | 853374 |  |  |  |  |  |  |  |  |  | 1 |
| JEM1 | 853372 |  |  |  |  |  |  |  |  |  | 1 |
| GAL1 | 852308 |  |  |  |  |  |  |  |  |  | 1 |
| Pep1 | 852264 |  |  |  |  |  |  |  |  |  | 1 |
| Ssa1p | 851259 |  |  |  |  |  |  |  |  |  | 1 |
| scFv | 652070 |  |  |  |  |  |  |  |  |  | 1 |
| sncg1 | 553679 |  |  |  |  |  |  | 1 |  |  |  |
| sncg2 | 550229 |  |  |  |  |  |  | 1 |  |  |  |
| sncb | 393944 |  |  |  |  |  |  | 1 |  |  |  |
| spot | 387357 |  |  |  |  | 1 |  |  |  |  |  |
| Atg5 | 365601 |  |  |  |  | 1 |  |  |  |  |  |
| Sirtuin 2 | 361532 |  |  |  |  |  | 1 |  |  |  |  |
| Lrrk2 | 300160 |  |  |  |  | 1 | 1 |  |  |  |  |
| BAG3 | 293524 |  |  |  |  | 1 |  |  |  |  |  |
| NeuN | 287847 |  |  |  |  |  | 1 |  |  |  |  |
| Let-7 | 266952 |  |  |  |  |  |  |  |  | 1 |  |
| TBSS | 219793 |  |  |  |  | 1 |  |  |  |  |  |
| PIKE | 216439 |  |  |  |  | 1 |  |  |  |  |  |
| VMAT2 | 214084 |  |  |  |  | 1 |  |  |  |  |  |
| NCEH-1 | 189866 |  |  |  |  |  |  |  |  | 1 |  |
| Daf-16 | 172981 |  |  |  |  |  |  |  |  | 1 |  |
| CX3CR1 | 171056 |  |  |  |  |  | 1 |  |  |  |  |
| TLR 8 | 170744 |  |  |  |  | 1 |  |  |  |  |  |
| aps-2 | 170685 |  |  |  |  |  |  |  |  | 1 |  |
| pSH1 | 121665 |  |  |  |  | 1 |  |  |  |  |  |
| PIKE-L | 116986 |  |  |  |  | 1 |  |  |  |  |  |
| sequestosome 1 | 113894 |  |  |  |  | 1 |  |  |  |  |  |
| SFXN3 | 94280 |  |  |  |  | 1 |  |  | 1 |  |  |
| SIRT1 | 93759 |  |  |  |  | 1 |  |  |  |  |  |
| eri-1 | 90459 |  |  |  |  |  |  |  |  | 1 |  |
| Sideroflexin 3 | 81855 |  |  |  |  | 1 |  |  | 1 |  |  |
| Atg7 | 74244 |  |  |  |  | 1 |  |  |  |  |  |
| LC3 | 66734 |  |  |  |  | 1 |  |  |  |  |  |
| LRRK2 | 66725 |  |  |  |  | 6 | 1 |  |  |  |  |
| Sirt2 | 64383 |  |  |  |  | 1 |  |  |  |  |  |
| M83 | 58986 |  |  |  |  | 3 |  |  |  |  |  |
| neutral cholesterol ester hydrolase 1 | 57552 |  |  |  |  |  |  |  |  | 1 |  |
| DJ-1 | 57320 |  |  |  |  | 2 | 1 |  |  |  |  |
| VGluT1 proteins | 57030 |  |  |  |  |  | 1 |  |  |  |  |
| Kv4.3 | 56543 |  |  |  |  | 1 |  |  |  |  |  |
| septin 6 | 56526 |  |  |  |  | 1 |  |  |  |  | 1 |
| ATP synthase | 46069 |  |  |  |  |  |  |  | 1 |  |  |
| hypoxia-inducible factor | 43580 |  |  |  |  | 1 |  |  | 1 |  |  |
| parkin | 40336 |  |  |  |  |  |  |  | 1 |  |  |
| fat body protein 1 | 39566 |  |  |  |  |  |  |  | 1 |  |  |
| muscle LIM protein at 60A | 37853 |  |  |  |  |  |  |  | 1 |  |  |
| manganese-superoxide dismutase | 36878 |  |  |  |  |  |  |  | 1 |  |  |
| TRAP1 | 35559 |  |  |  |  |  | 1 |  | 1 |  |  |
| troponin T | 32314 |  |  |  |  |  |  |  | 1 |  |  |
| Pink1 | 31607 |  |  |  |  |  |  |  | 1 |  |  |
| BAG3 | 29810 |  |  |  |  | 1 |  |  |  |  |  |
| Snca | 29219 |  |  |  |  | 1 | 2 |  |  |  |  |
| GRP78 | 25617 |  |  |  |  |  | 1 |  |  |  |  |
| DAT | 24898 |  |  |  |  |  | 1 |  |  |  |  |
| TLR 2 | 24088 |  |  |  |  | 1 |  |  |  |  |  |
| SIRT1 | 23411 |  |  |  |  | 1 |  |  |  |  | 1 |
| SIRT3 | 23410 |  |  |  |  |  | 1 |  |  |  |  |
| Sirt2 gene | 22933 |  |  |  |  | 1 | 1 |  |  |  | 1 |
| ATF6 | 22926 |  |  |  |  |  | 1 |  |  |  |  |
| 14-3-3 epsilon | 22627 |  |  |  |  | 1 |  |  |  |  |  |
| UCH-L1 | 22223 |  |  |  |  | 1 | 1 |  |  |  |  |
| SUMO-1 | 22218 |  |  |  |  | 1 |  |  |  |  |  |
| TRPC3 | 22065 |  |  |  |  | 1 |  |  |  |  |  |
| Grp94 | 22027 |  |  |  |  | 1 |  |  |  |  |  |
| mTOR | 21977 |  |  |  |  | 1 |  |  |  |  |  |
| TNFalpha | 21926 |  |  |  |  | 3 |  |  |  |  |  |
| TLR 4 | 21898 |  |  |  |  | 1 |  |  |  |  |  |
| synaptophysin | 20977 |  |  |  |  | 1 |  |  |  |  |  |
| STC-1 | 20855 |  |  |  |  | 1 |  |  |  |  |  |
| Plk2 | 20620 |  |  |  |  | 2 |  |  |  |  |  |
| PrPC | 19122 |  |  |  |  | 2 |  |  |  |  |  |
| PREP | 19072 |  |  |  |  | 1 |  |  |  |  |  |
| DARPP-32 | 19049 |  |  |  |  | 1 | 1 |  |  |  |  |
| PDE1C | 18575 |  |  |  |  | 1 |  |  |  |  |  |
| PDE1A | 18573 |  |  |  |  | 1 |  |  |  |  |  |
| Mapt | 17762 |  |  |  |  | 1 |  |  |  |  |  |
| MBP | 17196 |  |  |  |  | 1 |  |  |  |  |  |
| IL-1alpha | 16175 |  |  |  |  | 1 |  |  |  |  |  |
| IFNgamma | 15978 |  |  |  |  | 1 |  |  |  |  |  |
| SERT | 15567 |  |  |  |  | 1 |  |  |  |  |  |
| HSP70 | 15511 |  |  |  |  | 2 |  |  |  |  |  |
| HSF1 | 15499 |  |  |  |  | 1 |  |  |  |  |  |
| huntingtin | 15194 |  |  |  |  | 1 |  |  |  |  |  |
| GluN2B | 14812 |  |  |  |  | 1 |  |  |  |  |  |
| NMDAR | 14810 |  |  |  |  | 2 |  |  |  |  |  |
| mGluR5 | 14805 |  |  |  |  | 1 |  |  |  |  |  |
| GFAP | 14580 |  |  |  |  | 2 |  |  |  |  |  |
| Gba1 | 14466 |  |  |  |  | 1 |  |  |  |  |  |
| Fyn | 14360 |  |  |  |  | 2 |  |  |  |  |  |
| c-Fos | 14281 |  |  |  |  | 1 |  |  |  |  |  |
| DA transporter | 13162 |  |  |  |  | 1 |  |  |  |  |  |
| alphaB-crystallin | 12955 |  |  |  |  | 1 |  |  |  |  |  |
| corticotropin releasing factor | 12918 |  |  |  |  | 1 |  |  |  |  |  |
| choline acetyltransferase | 12647 |  |  |  |  | 1 |  |  |  |  |  |
| CD4 | 12504 |  |  |  |  | 1 |  |  |  |  |  |
| CA2 | 12349 |  |  |  |  | 1 |  |  |  |  |  |
| ApoE | 11816 |  |  |  |  | 1 |  |  |  |  |  |
| TPPP/p25 | 11076 |  |  |  |  | 1 |  |  |  |  |  |
| Plk2 | 10769 |  |  |  |  | 2 |  |  |  |  | 1 |
| TRAP1 | 10131 |  |  |  |  |  | 1 |  | 1 |  |  |
| DLP1 | 10059 |  |  |  |  |  | 1 |  |  |  |  |
| epsinR | 9685 |  |  |  |  |  |  |  |  |  | 1 |
| BAG3 | 9531 |  |  |  |  | 1 |  |  |  |  |  |
| ATG5 | 9474 |  |  |  |  | 1 |  |  |  |  |  |
| PERK | 9451 |  |  |  |  |  | 1 |  |  |  |  |
| Klotho | 9365 |  |  |  |  | 1 |  |  |  |  |  |
| ENS | 9053 |  |  |  |  |  | 1 |  |  |  |  |
| ULK1 | 8408 |  |  |  |  | 1 |  |  |  |  |  |
| 14-3-3 epsilon | 7531 |  |  |  |  | 1 |  |  |  |  |  |
| NAC32 | 7504 |  |  |  |  |  |  |  |  |  | 1 |
| gamma2 | 7453 |  |  |  |  |  |  | 1 |  |  |  |
| HSP | 7190 |  |  |  |  | 2 |  |  |  |  |  |
| Grp94 | 7184 |  |  |  |  | 1 |  |  |  |  |  |
| tumor necrosis factor receptor associated protein-1 | 7132 |  |  |  |  |  | 1 |  | 1 |  |  |
| A20 | 7128 |  |  |  |  | 1 |  |  |  |  |  |
| TGFbeta | 7040 |  |  |  |  | 1 |  |  |  |  |  |
| Ssa1p | 6737 |  |  |  |  |  |  |  |  |  | 1 |
| VMAT2 | 6571 |  |  |  |  | 1 | 1 |  |  |  |  |
| DA transporter | 6531 |  |  |  |  | 3 | 2 |  |  |  |  |
| SGLT1 | 6523 |  |  |  |  | 1 |  |  |  |  |  |
| GLUT4 | 6517 |  |  |  |  | 1 |  |  |  |  |  |
| GLUT | 6513 |  |  |  |  | 1 |  |  |  |  |  |
| Rab5 | 5868 |  |  |  |  | 1 |  |  |  |  | 1 |
| PrPC | 5621 |  |  |  |  | 2 |  |  |  |  |  |
| PLK | 5347 |  |  |  |  |  |  |  |  |  | 1 |
| myc | 4609 |  |  |  |  |  | 1 |  |  |  |  |
| LTB | 4050 |  |  |  |  | 1 |  |  |  |  |  |
| Gal4 | 3960 |  |  |  |  |  |  |  | 1 |  |  |
| LDLR | 3949 |  |  |  |  |  |  |  |  | 1 |  |
| Kv4.3 potassium channels | 3746 |  |  |  |  | 1 |  |  |  |  |  |
| Hsc70 | 3312 |  |  |  |  | 1 |  |  |  |  | 1 |
| rab | 3267 |  |  |  |  | 1 |  |  |  | 1 | 1 |
| huntingtin | 3064 |  |  |  |  | 2 |  |  | 1 |  |  |
| H2AX | 3014 |  |  |  |  | 1 | 1 |  |  |  |  |
| NMDAR2B | 2904 |  |  |  |  | 1 |  |  |  |  |  |
| GCL | 2729 |  |  |  |  | 1 |  |  |  |  |  |
| GFAP | 2670 |  |  |  |  | 1 |  |  |  |  |  |
| Hsc | 2523 |  |  |  |  |  |  |  |  |  | 1 |
| mTOR | 2475 |  |  |  |  | 1 |  |  |  |  |  |
| FcgammaRIIB | 2213 |  |  |  |  | 1 |  |  |  |  |  |
| FcgammaRI | 2209 |  |  |  |  | 1 |  |  |  |  |  |
| eps-8 | 2059 |  |  |  |  |  |  |  |  | 1 |  |
| eta | 1909 |  |  |  |  | 1 |  |  |  |  |  |
| dpi | 1832 |  |  |  |  | 1 |  |  |  |  |  |
| ATPase | 1769 |  |  |  |  | 1 |  |  |  |  |  |
| CHOP | 1649 |  |  |  |  |  | 1 |  |  |  |  |
| CatD | 1509 |  |  |  |  | 1 |  |  |  | 1 | 1 |
| crisprTFs | 1434 |  |  |  |  |  |  |  |  |  | 1 |
| Cdc42 | 998 |  |  |  |  | 1 |  |  |  |  | 1 |
| Cdc5 | 988 |  |  |  |  |  |  |  |  |  | 1 |
| CD68 | 968 |  |  |  |  | 1 |  |  |  |  |  |
| CaMKII | 818 |  |  |  |  | 1 |  |  |  |  |  |
| Cav | 763 |  |  |  |  | 1 |  |  |  |  |  |
| CA3 | 761 |  |  |  |  | 1 |  |  |  |  |  |
| Akt | 207 |  |  |  |  | 1 |  |  |  |  | 1 |
| Iba1 | 199 |  |  |  | 1 |  | 1 |  |  |  |  |
| AAV | 17 |  |  |  |  |  | 1 |  |  |  |  |
