# Supplementary material for: Menagerie: A text-mining tool to support animal-human translation in neurodegeneration research
Source: PLoS One. 2019 Dec 17;14(12):e0226176. doi: 10.1371/journal.pone.0226176 (PMC6917268; doi:10.1371/journal.pone.0226176)
Supplement: S7 Table — We manually classified entities extracted by the Interventions/disease modifiers module as Established or Experimental. The former list is presented below—all remaining Interventions were included in the Experimental category. (DOCX) [file pone.0226176.s008.docx]

**S7 Table**: Interventions classified as Established

We manually classified entities extracted by the Interventions/disease modifiers module as Established or Experimental. The former list is presented below – all remaining Interventions were included in the Experimental category.

| **ESTABLISHED** |  |
| --- | --- |
| INTERVENTION TERM | INTERVENTION ID |
| levodopa | D007980 |
| Deep-brain stimulation | D046690 |
| haloperidol | D006220 |
| Acupuncture | D026881 |
| Pramipexole | C061333 |
| exercise | D015444 |
| Dopamine transporter | D050483 |
| rasagiline | C031967 |
| monoamine oxidase | D008995 |
| riluzole | D019782 |
| reserpine | D012110 |
| cabergoline | C047047 |
| physical therapy | C0949766 |
| Selegiline | D012642 |
| Vesicular monoamine transporter | D050493 |
| ropinirole | C046649 |
| galantamine | D005702 |
| amantadine | D000547 |
| rotigotine | C047508 |
| dopamine agonist | D018491 |
| ablation | C0547070 |
| rolipram | D020889 |
| preladenant | C539997 |
| denervation | D003714 |
| dopaminergic mechanisms | C3825331 |
| duloxetine | C058218 |
| piribedil | D010891 |
| combined SSRI- antipsychotic treatment | C0009429 |
| Dopamine D3 receptor | D050637 |
| Anti-Parkinsonian Agents | C0040616 |
| Benserazide | D001545 |
| bromocriptine | D001971 |
| citalopram | D015283 |
| dopamine D2 receptor | D017448 |
| entacapone | C071192 |
| Buspirone | D002065 |
| Cholinesterase | D002802 |
| MAO-B Inhibitor | C0595265 |
| apomorphine | D001058 |
| levodopa methyl ester | C035420 |
| donepezil | C076946 |
| monoamine | C0599682 |
| Perampanel | C551441 |
| Piracetam | D010889 |
| band | C0175723 |
| dancing | D003614 |
| diazepam | D003975 |
| apraclonidine | C016986 |
| dopaminergic receptors | C0034798 |
| Citicoline | D003566 |
| dopamine transporters | C0114838 |
| DAT-SPECT | C0114838 |
| benzazepine | D001552 |
| Muscarinic receptors | D011976 |
| sildenafil | C101426 |
| pergolide | D010479 |
| D2 Receptors | C0058698 |
| dopamine D2/D3 receptors | C0058698 |
| Dopamine D1 Receptor | D017447 |
| task performance | D013647 |
